# Supplementary material for: Health checks for adults with intellectual disability and association with survival rates: a linked electronic records matched cohort study in Wales, UK
Source: BMJ Open. 2022 Apr 13;12(4):e049441. doi: 10.1136/bmjopen-2021-049441 (PMC9013997; doi:10.1136/bmjopen-2021-049441)
Supplement: Author's manuscript [file bmjopen-2021-049441.draft_revisions.pdf]

BMJ Open

BMJ Open is committed to open peer review. As part of this commitment we make the peer review history of every article we publish publicly available.

When an article is published we post the peer reviewers' comments and the authors' responses online. We also post the versions of the paper that were used during peer review. These are the versions that the peer review comments apply to.

The versions of the paper that follow are the versions that were submitted during the peer review process. They are not the versions of record or the final published versions. They should not be cited or distributed as the published version of this manuscript.

BMJ Open is an open access journal and the full, final, typeset and author-corrected version of record of the manuscript is available on our site with no access controls, subscription charges or pay-per-view fees (http://bmjopen.bmj.com).

If you have any questions on BMJ Open's open peer review process please email info.bmjopen@bmj.com

BMJ Open

Health checks for adults with intellectual disability are associated with improved survival rates for those with autism or Down's Syndrome – a linked electronic records matched cohort study in Wales, UK

Journal:	*BMJ Open*
Manuscript ID	bmjopen-2021-049441
Article Type:	Original research
Date Submitted by the Author:	20-Aug-2021
Complete List of Authors:	Kennedy, Natasha; Swansea University, National Centre for Population Health and Wellbeing<br>Kennedy, Jonathan; Swansea University; Swansea University<br>Kerr, Mike; Cardiff University School of Medicine<br>Dredge, Sam; Swansea University, School of Medicine<br>Brophy, Sinead; University of Swansea Institute of Health Research, School of Medicine
Keywords:	Quality in health care < HEALTH SERVICES ADMINISTRATION & MANAGEMENT, EPIDEMIOLOGY, GENERAL MEDICINE (see Internal Medicine)

SCHOLARONE™
Manuscripts

I, the Submitting Author has the right to grant and does grant on behalf of all authors of the Work (as defined in the below author licence), an exclusive licence and/or a non-exclusive licence for contributions from authors who are: i) UK Crown employees; ii) where BMJ has agreed a CC-BY licence shall apply, and/or iii) in accordance with the terms applicable for US Federal Government officers or employees acting as part of their official duties; on a worldwide, perpetual, irrevocable, royalty-free basis to BMJ Publishing Group Ltd ("BMJ") its licensees and where the relevant Journal is co-owned by BMJ to the co-owners of the Journal, to publish the Work in this journal and any other BMJ products and to exploit all rights, as set out in our licence.

The Submitting Author accepts and understands that any supply made under these terms is made by BMJ to the Submitting Author unless you are acting as an employee on behalf of your employer or a postgraduate student of an affiliated institution which is paying any applicable article publishing charge ("APC") for Open Access articles. Where the Submitting Author wishes to make the Work available on an Open Access basis (and intends to pay the relevant APC), the terms of reuse of such Open Access shall be governed by a Creative Commons licence – details of these licences and which Creative Commons licence will apply to this Work are set out in our licence referred to above.

Other than as permitted in any relevant BMJ Author's Self Archiving Policies, I confirm this Work has not been accepted for publication elsewhere, is not being considered for publication elsewhere and does not duplicate material already published. I confirm all authors consent to publication of this Work and authorise the granting of this licence.

For peer review only

Health checks for adults with intellectual disability are associated with improved survival rates for those with autism or Down's Syndrome – a linked electronic records matched cohort study in Wales, UK.

Natasha Kennedy PhD[a], Jonathan Kennedy EngD[b, c], Michael Kerr PhD[d], Sam Dredge PGCE[a], Sinead Brophy PhD[a]

a. Swansea University Medical School, Data Science Building, Swansea University, Swansea, United Kingdom
b. Swansea University Medical School, Data Science Building, Swansea University, Swansea, United Kingdom. Telephone: 01792 606321 Email: J.I.Kennedy@swansea.ac.uk
c. Health Data Research UK Wales and Northern Ireland, Swansea University Medical School, Data Science Building, Swansea University, Swansea, United Kingdom.
d. Institute of Psychological Medicine and Clinical Neurosciences, Cardiff University School of Medicine, Cardiff, UK

Corresponding author: Professor Sinead Brophy, National Centre for Population Health and Wellbeing Research, Data Science Building, School of Medicine, Swansea University. SA2 8PP. UK. Tel 01792602058, email: s.brophy@swansea.ac.uk.

For peer review only

Abstract

Objective

To examine if mortality rates are lower in people with intellectual disability who have had a health check compared to those who have not had health checks.

Setting

General practice records of 26,954 people with an intellectual disability in Wales between 2005-2017.

Primary outcome measure:

ONS mortality data. Cox Regression was used to examine time to death adjusted for co-morbidities and gender.

Results

Patients who had a health check were stratified by those who (a) had a confirmed health check i.e. Read Code for a health check (n = 7,650 (22.2 %)) and (b) had no evidence of receiving a health check in their medical record. Patients with a health check were matched for age at time of health check with two people who did not have a health check. The health check was associated with improved survival for those with autism or Down's Syndrome (Hazard Ratio (HR): 0·58 (95 % CI: 0·38 to 0·91) and HR: 0·76 (95 % CI: 0·70 to 0·91)), respectively). There was no evidence of improved survival for those diagnosed with diabetes or cancer. The people who had a health check were more likely to be older, have epilepsy and less likely to have autism or Down's Syndrome.

Conclusions

Health checks are likely to influence survival if started before a person is diagnosed with a chronic condition, especially for people with autism or Down's Syndrome.

For peer review only - http://bmjopen.bmj.com/site/about/guidelines.xhtml

Article summary: Strengths and limitations of the study:

- These findings are based on follow up of 26,954 people with intellectual disabilities
- Patients who have had a health check, but it was not recorded in their GP data, will be misclassified and so differences observed will likely be higher than those reported in this study.
- Patients who live longer are more likely to have a health check (survival basis) we have tried to account for this by matching with age matched controls. However, fewer controls were available to match for older people who had a health check, which means the controls were more often younger in the older age group patients (within 5 years matched age) and this may have influenced the findings for comparisons at older ages.

For peer review only

Introduction

People with an intellectual disability experience more health conditions such as; epilepsy, autism, and dental problems.[1] In addition, they are at a higher risk of leading sedentary lives and becoming overweight; resulting in developing diabetes, cardiovascular problems, and are more likely to be exposed to poverty, poor housing conditions, unemployment, and other social determinates of poor health.[2] Finally, communication and ability to act on health promotion information means access to health care provision will be reduced for people with intellectual disability. Inequalities in health are apparent with people with intellectual disabilities, health checks have been recommended as one component of international health policy to address the poorer health of people with intellectual disabilities.[3] Annual health checks for people with an intellectual disability and being present on the social services register were introduced into Wales in 2006 and in England in 2007. The Cardiff/Welsh annual health check for adults is aimed at early detection and treatment.[4] In 2010 the Improving Health and Lives – The Learning Disabilities Public Health Observatory (IHAL) review demonstrated that the health check improves detection of unmet, potentially treatable health needs, [5] additionally, a recent study indicated that General Practitioners (GP) practices with Enhanced Service (eg. incentivised in offering the health check) had more health action plans and secondary care referrals.[6] Previous studies [3, 7] identified that health checks lead to detection of unmet needs and targeted actions to address health needs. The health check has been observed to improve detection of less serious health conditions such as ear wax obscuring one or both eardrums, and dental problems which will greatly influence a person's quality of life.[3] They have been found to improve detection of serious conditions such as cancer, and may improve the health promotion [3, 4, 6]. Conversely, it is not known if this translates into health gain. Few studies have evaluated the extent to which providing health checks leads to long term health benefits. In fact, a new evaluation of the impact of the Directed Enhanced Services (DES) in England found no significant difference between health check and controls (no health check) in terms of intermediate outcomes such as control of blood pressure[8] and annual health checks where not found to reduce emergency admissions, but they did appear to reduce preventable emergency admissions (e.g. those for diabetes or COPD[9]). The lack of evidence of the long term benefits of health checks was one reason for practices not offering them, as without this evidence the time taken to organise and undertake assessments is a real barrier to GPs.[10] This study specifically examines if health checks are associated with better survival and lower rates of mortality compared to those who have no health check.

Methods

Study Design

The Secure Anonymised Information Linkage (SAIL) databank is a data repository which allows person-based data linkage across datasets. This databank includes Welsh GP data, hospital in- and out-patient records, as well as mortality data collected by the Office of National Statistics (ONS). SAIL comprises over a billion anonymised records. It utilises a split-file approach to ensure anonymisation, overcome issues of confidentiality, and disclosure in health-related data warehousing. Demographic data is sent to a partner organisation, NHS Wales Informatics Service, where identifiable information is removed.

For peer review only - http://bmjopen.bmj.com/site/about/guidelines.xhtml

Clinical data are sent directly to the SAIL databank where an individual is assigned an encrypted Anonymised Linking Field (ALF). The ALF is utilised to link anonymised individuals across datasets, facilitating longitudinal analysis of an individual's journey through multiple health, education, and social datasets. Data collected by GP's is captured via Read Codes (5-digit codes related to diagnosis, medication, and process of care codes). Hospital in- and out-patient data are collected in the Patient Episode Database for Wales, which contains clinical information regarding patients' hospital admissions, discharges, diagnoses, and operations employing the International Classification of Diseases (ICD-10) clinical coding system. The ONS Mortality dataset contains demographic data, place of death, and underlying cause of death (also ICD-10). Anonymised routine data analysis is not subject to ethical committee approval but is subject to Information Governance approval. This project has been approved by the IGRP (Information Governance Review Panel) which provides independent approval that the research is within the public interest and includes representatives from the National Research Ethics Service, British Medical Association and the public.

Patient and Public Involvement: This work was discussed with the Learning Disability Ministerial Advisory Group for Wales which includes people with learning disabilities and those caring for those with learning disability. Those with learning disability were not involved in the design of the study, or conduct of the study.

All data was selected from 2005 – 2017, patients were flagged for intellectual disability (See Supplementary file 1 and definition provided in [11]). Patients entered the study when they became eligible for a health check, at age 18 or in 2006 whichever is later. The eligible patients were stratified by (a) ever had a confirmed health check (Read Code for a health check recorded) at any time in the patients record, and (b) had no evidence of receiving a health check in their medical record.

Procedures

Statistical Analysis

STATA 15 was utilised for all analysis. Descriptive statistics for each category were generated. Patients with a health check (index case) were matched for age (+/- 5 years) at health check with 2 patients who did not have a health check forming a matched cohort (Figure 1). Cox regression was employed to examine time to mortality, from date of study entry. Analysis was adjusted for gender and co-morbidities (autism, Down's, diabetes, epilepsy, and cancer), the cluster command in STATA was used to account for matching design. The incidence of death (deaths over number of person years follow up) are presented for each co-morbidity separately. However, the comparison patient was not matched on co-morbidity in this sub-analysis. People were censored if they moved out of Wales or were lost to follow up. This dataset included everyone with an intellectual disability over the age of 18 since 2006.

Results

There were 26,954 people with intellectual disability between 2006 and 2017 in Wales (11-year period) and of these 7650 (22.2 %) have a GP record of ever having had a health check (see table 1). The

For peer review only - http://bmjopen.bmj.com/site/about/guidelines.xhtml

proportion of people who have a health check by year is between 1·69 % (in 2006 when health checks began) and 12·4 % in 2015 (see table 2 and Figure 2) This indicates that 77.8 % of people with intellectual disabilities have no record of having a health check in their electronic medical records and each year 87 % of eligible people do not have a health check.

The people who had a health check were more likely to be older (see table 1) (average age 40.5 years (health check compared to 31.3 years (No health check) (difference: 9.2 (95 % CI: 8.8 to 9.7)) and to have epilepsy (39·3 % (health check) compared to 28.7 % (no health check) difference 10.6 % (95 % CI: 9.3 % to 11·9 %)). Those with autism were less likely to have a health check (15% of those with autism have had a health check). There was no difference in socio-economic level of those who had a health check compared to those who did not (see Table 1).

In order to adjust for the older age of those having a health check, each index case was matched for the age (+/- 5 years) at health check with two patient who had no record of having had a health check (see Table 2). There was a trend for those who had a health check to have a lower rate of death compared to their matched comparisons (2.4 per 1000 per year fewer deaths, Figure 3). The health check showed better survival for those with autism (HR: 0.58 (95%CI: 0.38 to 0.91) and those with downs (HR: 0.76 (95% CI: 0.70.64 to 0.91) (Table 2). There was no evidence that survival was improved if a patient had diabetes, epilepsy or cancer. However, in nearly every case the diagnosis of these conditions happened before the date of the health check.

Screening for cancer was very low in both the health check and no health check groups (7.6% and 5.3%) and there was no evidence of lower death rates in those who had cancer and had a health check compared to those who do not have a health check. There was a slight trend to higher rate of death in those who have a health check and have cancer. However, it should be noted that the comparison matches would have been unlikely to also have cancer.

Discussion:

This study examined the medical records of 26,954 people with an intellectual disability and observed that having a health check was associated with reduced mortality for people with autism and those with Down's syndrome. There was little evidence that it reduced mortality rates for those diagnosed with conditions such as diabetes or epilepsy and there was no evidence that health check improved outcomes for people diagnosed with cancer.

This study only examined those who had a record of having at least one health check recorded in their medical notes through recording a READ code. If a person had a health check but this was not coded or recorded as such, we will miss-classify these individuals in the no health check group. Previous studies suggested that the number of people having health checks was 41% in 2008 in Wales[12] and this number is based on GP submissions to the Community, Primary Care and Health Services Policy Division of the Welsh Assembly Government. However, in this study it was observed that 22 % of people with intellectual disability had ever received a health check coded in their medical records. This means

For peer review only - http://bmjopen.bmj.com/site/about/guidelines.xhtml

only half of those who might have had a health check had it coded in their notes and so we may potentially be missing half the people who had health check. This means, the true difference between those who have health checks and those who do not may be larger than this study has detected due to miss-classification error. In addition, this study can only follow individuals who are registered with their GP and had a code in their medical record identifying that they had an intellectual disability. This study could not observe the level of intellectual disability. However, the results indicate that more people who received the health check, possessed a diagnosis of epilepsy. This would suggest it is those with more severe intellectual disability who are most likely to be given a health check and they are likely to have had a higher mortality rate. This assumption would be supported by previous research[9] which found those who have health checks are older, have higher levels of support needs and more likely to be in communal living. There were fewer controls available in the older age groups and this means the control was more often younger than the case (within 5 years), this may influence the finding for comparisons at older ages.

The number of eligible people having a health check recorded by the GP practice each year was approximately 10 - 12 %. This suggests that many people may have received one health check but then not undergone the health check annually and that the majority of people with intellectual disability never have a health check.

Health checks have been indicated to be valued by carers and people with an intellectual disability; they have been revealed to not increase costs, and do detect health requirements earlier[7,13]. This study shows that they also associated with a long-term gain in improving survival for people with an intellectual disability, especially for those with autism or down's syndrome. The findings of the study suggest that health checks may be most beneficial for prevention of morbidities, it did not find evidence that when a person has existing health conditions such as diabetes or cancer that it improves survival. This may be because once diagnosed, those with ID have the same care for their diabetes/epilepsy/cancer whether receiving health checks or not. Previous research has shown that emergency admission for these conditions is reduced[9] when a person has health checks. The health check may improve prevention and therefore be associated with improved survival for those without existing co-morbidities. There is the argument that the health check itself does not lead to improved survival but instead this relationship is confounded by health check being given by more engaged GP practices, more engaged family members and better overall care. However, the lack of association of the health check with enhanced survival when diagnosed with a chronic condition and especially those with cancer, would refute this argument of confounding.

Conclusions

Health checks are associated with a trend to improved survival for people with intellectual disabilities, especially for people with autism and Downs. Increasing the uptake of health checks could help with prevention of morbidities and improve survival for people who do not already have chronic disease. However, there was limited evidence from this work that survival is improved when a person has existing

For peer review only - http://bmjopen.bmj.com/site/about/guidelines.xhtml

morbidities. This study shows health outcome benefits of health checks in terms of lower rates of mortality for those with autism or Downs.

Contributorship statement:

The concept of the study and design was by MK, SB. The dataset was prepared by JK and analysis undertaken by NK with supervision from SB, MK, the findings and interpretation was after discussion with stakeholders a consultation which was undertaken by SD. All authors contributed to drafting the paper and all have given approval of the final submission.

Competing interests

None to declare.

Funding

This work was supported by Health and Care Research Wales (National Centre for Population Health and Well-being), and Health Data Research UK (NIWA1) which receives its funding from HDR UK Ltd. HDR UK Ltd is funded by the UK Medical Research Council, Engineering and Physical Sciences Research Council, Economic and Social Research Council, Department of Health and Social Care (England), Chief Scientist Office of the Scottish Government Health and Social Care Directorates, Health and Social Care Research and Development Division (Welsh Government), Public Health Agency (Northern Ireland), British Heart Foundation (BHF), and the Wellcome Trust.

Data sharing statement

The dataset used in this article is available via the Secure Anonymised Information Linkage (SAIL) databank, which is part of the national ehealth records infrastructure for Wales. 19. The data is accessible by applying to SAIL. For further information on the SAIL databank and enquiries in how to access the data, please visit the SAIL website (http://www.saildatabank.com).

Acknowledgements

This study uses anonymised data provided by patients, collected by the NHS as part of their care and support and held in the Secure Anonymised Information Linkage (SAIL) Databank. We would like to acknowledge all data providers that make anonymised data available for research. All data used can be accessed by contacting the SAIL gateway (https://saildatabank.com/).

For peer review only

Table 1: Demographics of those who have a health check compared to those with no health check (from time eligible for health check)

	No health check	Health check	Difference (95% CI)
**Demographic Characteristics**			
**Total (n)**	19,304 (56 %)	7650 (22.2 %)	
**Average number of years of follow-up**	5.74	3.24	
**Mean age (sdev)**	35 (15.6)	40.5 (16.4)	9.2 (8.8 to 9.7)
**Age at baseline**			
**18 to 50 years old**	15,904 (82%)	5387 (70.4%)	12% (10.8 to 13.1)
**More than 50 years old**	3400 (17.6 %)	2263 (29.6 %)	-12% (-13.1 to -10.8)
**Male**	11655 (60·0 %)	4401 (57·0 %)	2.9% (1.5 to 4.2)
**Autism**	6290 (32.2 %)	1180(15.4 %)	17.2% (16.1 to 18.2)
**Down's**	3364 (17.4 %)	915 (12 %)	5.5 % (4.6 to 6.4)
**Diabetes**	2332 (12 %)	1030 (13.5 %)	-1.38% (-2.3 to -0.5)
**Epilepsy**	5536 (28.7 %)	3004 (39.3 %)	-10.6% (-11.6 to -9.3)
**Cancer Screening**	1030 (5.3 %)	582 (7.6 %)	-2.27 % (-2.0 to -1.6)
**Cancer**	3095 (16 %)	1040 (13 %)	2.4% (1.5 to 3.4)
**Died**	2,527 (13 %)	751 (9·8 %)	3.3% (2.4 to 4.0)
**Townsend Scores**			
**1**	2515 (13 %)	1087 (14 %)	-1.18 (-2 to -0.3)
**2**	2978 (15.4%)	1137 (14.9%)	No difference
**3**	3317 (20%)	1535 (17 %)	2.8 (1.9 to 3.9)
**4**	3778 (22%)	1706 (19.6 %)	2.7 (1.7 to 3.8)
**5 (most deprived)**	4468 (23%)	1829 (23 %)	No difference
**N/A**	2248(11.6%)	356 (4.6%)	7 (6.3 to 7.6)

For peer review only - http://bmjopen.bmj.com/site/about/guidelines.xhtml

Table 2: Survival in those who have a health check compared to no health check group (2:1 matched (for age +/- 5 years).

	No health check (2 matches) N=12,152	Health check (Index case) N=7650
Survival (time to death)		
Total (deaths per 1000/year)	19.6 (95 % CI: 18.8 – 20.3)	17.1(95 % CI: 15.9 – 18.4)
Hazard ratio adjusted for co-morbidities and gender	1	0.94 (95 % CI: 0.89 – 1.04)
Males (deaths per 1000/year)	19 (95 % CI: 18.0 – 20.1)	16.9 (95 % CI: 15.3 – 18.6)
Hazard Ratio adjusted for co-morbidities and gender	1	0.93 (95 % CI: 0.83 – 1.03)
Females (deaths per 1000/year)	20.2 (95 % CI: 19.1 – 21.4)	17.4 (95 % CI: 15.6 – 19.4)
Hazard Ratio adjusted for co-morbidities and gender	1	0.94 (95 % CI: 0.84- 1.07)
For those with Autism	8.4 (95 % CI: 7.4 – 9.6)	3.55 (95 % CI: 2.3 – 5.3)
Crude Hazard Ratio	1	**0.58 (95 % CI: 0.37 - 0·91)**
Adjusted for co-morbidities And gender	1	**0.56 (95 % CI: 0·36 – 0·89)**
For those with Down's	35.6 (95 % CI: 32.8- 38.6)	27.2 (95 % CI: 23.2 to 31.9)

For peer review only - http://bmjopen.bmj.com/site/about/guidelines.xhtml

Crude Hazard Ratio	1	**0.76 (95 % CI: 0.63 – 0.91)**
Adjusted for co-morbidities And gender	1	**0·80 (9 % CI: 0·67 – 0.95)**
For those with Diabetes	31.7 (95 % CI: 29.4 – 34.2)	27.6 (95 % CI: 23.8 – 32.2)
Crude Hazard Ratio	1	0·97 (95 % CI: 0·81 – 1.15)
Adjusted for co-morbidities And gender	1	0·98 (95 % CI: 0·82 – 1.16)
For those with Epilepsy	24.3 (95 % CI: 22.9 – 25.7)	21.9 (95 % CI: 19.8 – 24.2)
Crude Hazard Ratio	1	1.03 (95 % CI: 0·91 - 1.16)
Adjusted for co-morbidities And gender	1	1.07 (95 % CI: 0.94-1.20)
For those with Cancer Screening	24.4 % CI: 22.6 – 28.6)	21.2 (95 % CI: 17.1 – 26.3)
Crude Hazard Ratio	1	1.0 (95 % CI: 0.77 – 1.30)
Adjusted for co-morbidities And gender	1	1.08 (95 % CI: 0.83 -1.40)
For those with Cancer	36.6 (95 % CI: 34.3 – 39.0)	38.5 (95 % CI: 33.7 – 43.9)
Crude Hazard Ratio	1	1.18 (95 % CI: 1.0 – 1.3)
Adjusted for co-morbidities And gender	1	1.18 (95 % CI: 1.0 -1.4)

For peer review only

References:

1 van Schrojenstein Lantman-de Valk HM, Walsh PN. Managing health problems in people with intellectual disabilities. *BMJ* 2008, **337**:a2507.

2 Turner S. Health Inequalities and People with Learning Disabilities in the UK: 2011. In.: Improving Health and Lives: Learning Disabilities Observatory; 2011.

3 Robertson J, Hatton C, Emerson E, Baines S. The impact of health checks for people with intellectual disabilities: an updated systematic review of evidence. *Res Dev Disabil* 2014, **35**(10):2450-2462.

4 Felce D, Baxter H, Lowe K, Dunstan F, Houston H, Jones G, Grey J, Felce J, Kerr M. The Impact of Checking the Health of Adults with Intellectual Disabilities on Primary Care Consultation Rates, Health Promotion and Contact with Specialists. In., vol. 21: Journal of Applied Research in Intellectual Disabilities; 2008: 597-602.

5 Ahmed Z, Fraser W, Kerr MP, Kiernan C, Emerson E, Robertson J, Felce D, Allen D, Baxter H, Thomas J. Reducing antipsychotic medication in people with a learning disability. *Br J Psychiatry* 2000, **176**:42-46.

6 Buszewicz M, Welch C, Horsfall L, *et al*. Assessment of an incentivised scheme to provide annual health checks in primary care for adults with intellectual disability: a longitudinal cohort study. *Lancet Psychiatry* 2014, **1**(7):522-530.

7 Robertson J, Roberts H, Emerson E: Health Checks for people with learning disabilities: a systematic review of evidence. In. Stockton-on-Tees: Improving Health and Lives: Learning Disabilities Observatory; 2010.

8 Chauhan U, Reeve J, Kontopantelis E, Hinder S, Nelson P, Doran T. Impact of the English directly enhanced service (DES) for learning disability. In. Manchester: Manchester University; 2012.

9. Carey IM, Hosking F, Harris T, DeWilde S, Beighton C, Shah S, Cook. J Epidemiol Community Health 2017: 71: 52-58.

10 Walmsley J. If annual health checks for people with learning disabilities can address failings identified in their health care, why are more GPs not doing them? In., vol. 338; 2009.

11 Brophy S, Kennedy J, Fernandez-Gutierrez F, et al. Characteristics of Children Prescribed Antipsychotics: Analysis of Routinely Collected Data. *J Child Adolesc Psychopharmacol* 2018, **28**(3):180-191.

12 Perry J, Kerr M, Felce D, Bartley S, Tomlinson J. Monitoring the public health impact of health checks for adults with learning disability in Wales. In.: Public Health Wales.; 2010.

13 Romeo R, Knapp M, Morrison J, Melville C, Allan L, Finlayson J, et al. Cost estimation of a healthcheck intervention for adults with intellectual disabilities in the UK. Journal of Intellectual Disability Research 2009;53:426-39.

For peer review only - http://bmjopen.bmj.com/site/about/guidelines.xhtml

Figure 1: Flow diagram of participant inclusion.

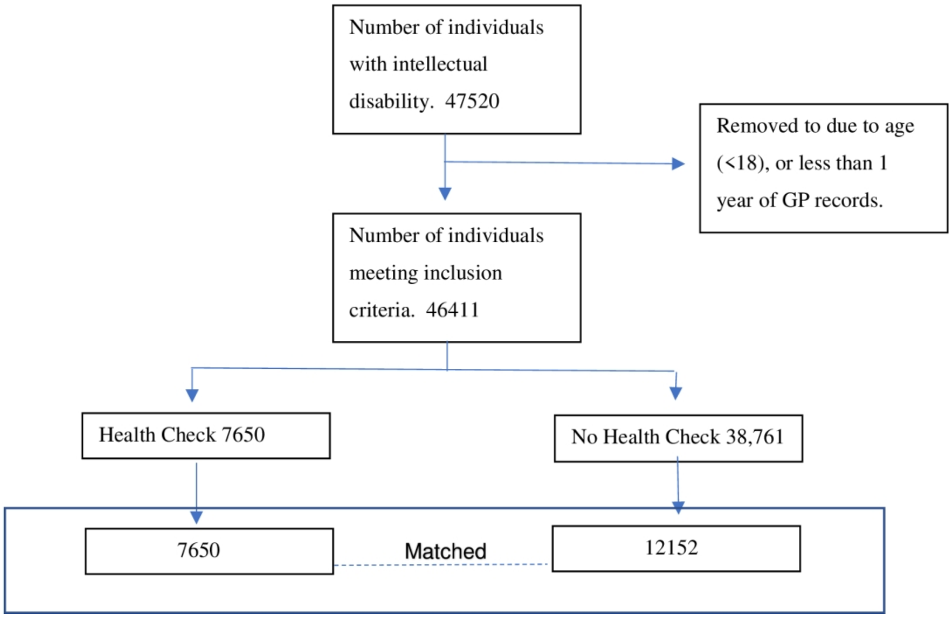

Flow diagram of participant inclusion

163x113mm (300 x 300 DPI)

Figure 2: Number of health checks per year.

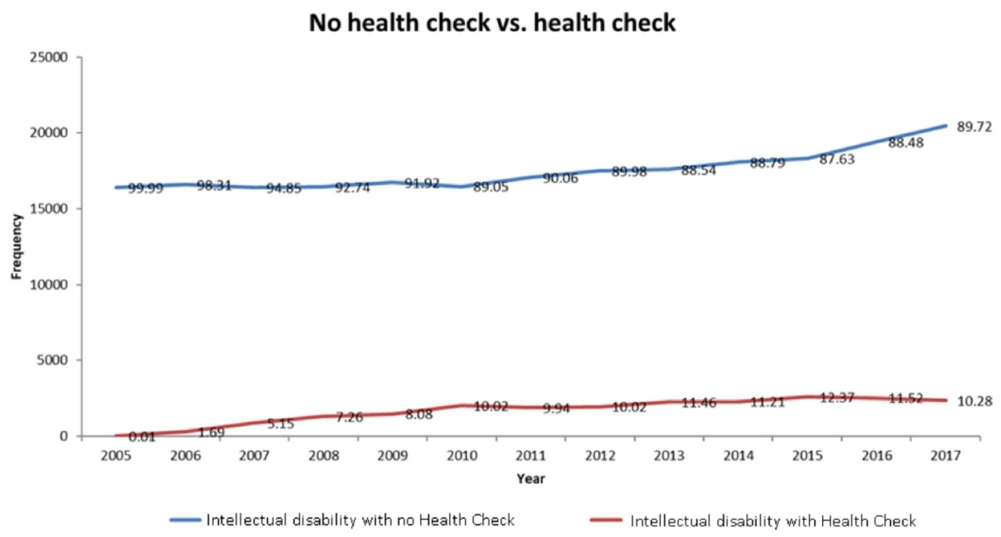

Number of health checks per year

190x124mm (300 x 300 DPI)

Figure 3: Survival in those who have a health check compared to no health check group (2:1 matched (for age +/- 5 years).

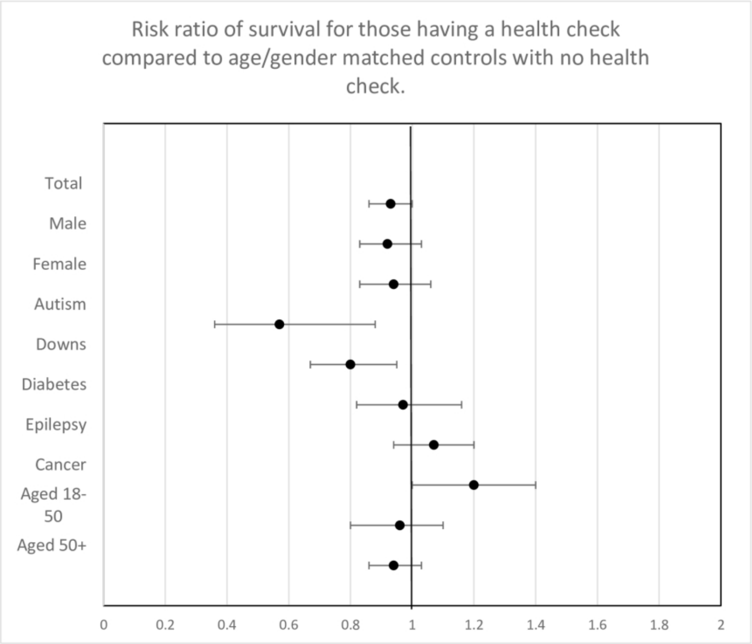

1

Survival in those who have a health check compared to no health check group (2:1 matched (for age +/- 5 years).

143x222mm (300 x 300 DPI)

Health Assessment

READ_CODE	READ_DESC
9HB5.	Learning disabilities annual health assessment

Downs (GP)

READ_CODE	READ_DESC
PJ0..	Down's syndrome - trisomy 21
PJ00.	Trisomy 21, meiotic nondisjunction
PJ0z.	Down's syndrome NOS
PJ02.	Trisomy 21, translocation
PJ01.	Trisomy 21, mosaicism

Hospital

ALT_CODE	DESCRIPTION
Q900	Trisomy 21, meiotic nondisjunction
Q901	Trisomy 21, mosaicism (mitotic nondisjunction)
Q902	Trisomy 21, translocation
Q909	Down syndrome, unspecified
Q909	Down's syndrome, unspecified

ALT_CODE	DESCRIPTION
Q90	Down syndrome

Autism (GP)

READ_CODE	READ_DESC
1J9..	Suspected autism
E1401	Residual infantile autism
E1400	Active infantile autism
E140.	Infantile autism
Eu841	[X]Atypical autism
E140z	Infantile autism NOS
Eu840	[X]Childhood autism

Hospital

ALT_CODE	DESCRIPTION
F840	Childhood autism
F841	Atypical autism

For peer review only

Learning disability (GP)

READ_CODE	READ_DESC
1J9..	Suspected autism
69DB.	Learning disability health examination
8Ce6.	Preferred place of care - learning disability unit
8H4f.	Referral to learning disabilities psychiatrist
8HHP.	Referral to learning disability team
8Hg2.	Discharge from learning disability team
918e.	On learning disability register
94Z9.	Preferred place of death: learning disability unit
9HB..	Learning disabilities administration status
9HB0.	Learning disabilities health action plan declined
9HB1.	Learning disabilities health action plan offered
9HB2.	Learning disabilities health action plan reviewed
9HB3.	Learning disabilities health assessment
9HB4.	Learning disabilities health action plan completed
9HB5.	Learning disabilities annual health assessment
9HB6.	Learning disabilities annual health assessment declined
9HB7.	Did not attend learning disabilities annual health assessment
9N0y.	Seen in learning disabilities clinic
9hL..	Exception reporting: learning disability quality indicators
9mA..	Learning disability annual health check invitation
9mA0.	Learning disability annual health check verbal invitation
9mA1.	Learning disability annual health check telephone invitation
9mA2.	Learning disability annual health check letter invitation
9mA20	Learning disability annual health check invitation first letter
9mA21	Learning disability annual health check invitation second letter
9mA22	Learning disability annual health check invitation third letter
E140.	Infantile autism
E1400	Active infantile autism
E1401	Residual infantile autism
E140z	Infantile autism NOS
E3...	Mental retardation
E30..	Mild mental retardation, IQ in range 50-70
E31..	Other specified mental retardation
E310.	Moderate mental retardation, IQ in range 35-49
E311.	Severe mental retardation, IQ in range 20-34
E312.	Profound mental retardation with IQ less than 20
E31z.	Other specified mental retardation NOS
E3y..	Other specified mental retardation
E3z..	Mental retardation NOS
Eu7..	[X]Mental retardation
Eu70.	[X]Mild mental retardation
Eu700	[X]Mild mental retardation with the statement of no, or minimal, impairment of behaviour
Eu701	[X]Mild mental retardation, significant impairment of behaviour requiring attention or treatment

For peer review only

Eu70y	[X]Mild mental retardation, other impairments of behaviour
Eu70z	[X]Mild mental retardation without mention of impairment of behaviour
Eu71.	[X]Moderate mental retardation
Eu710	[X]Moderate mental retardation with the statement of no, or minimal, impairment of behaviour
Eu711	[X]Moderate mental retardation, significant impairment of behaviour requiring attention or treatment
Eu71y	[X]Mod retard oth behav impair
Eu71z	[X]Moderate mental retardation without mention of impairment of behaviour
Eu72.	[X]Severe mental retardation
Eu720	[X]Severe mental retardation with the statement of no, or minimal, impairment of behaviour
Eu721	[X]Severe mental retardation, significant impairment of behaviour requiring attention or treatment
Eu72y	[X]Severe mental retardation, other impairments of behaviour
Eu72z	[X]Severe mental retardation without mention of impairment of behaviour
Eu73.	[X]Profound mental retardation
Eu730	[X]Profound mental retardation with the statement of no, or minimal, impairment of behaviour
Eu731	[X]Profound mental retardation, significant impairment of behaviour requiring attention or treatment
Eu73y	[X]Profound mental retardation, other impairments of behaviour
Eu73z	[X]Profound mental retardation without mention of impairment of behaviour
Eu7y.	[X]Other mental retardation
Eu7y0	[X]Other mental retardation with the statement of no, or minimal, impairment of behaviour
Eu7y1	[X]Other mental retardation, significant impairment of behaviour requiring attention or treatment
Eu7yy	[X]Other mental retardation, other impairments of behaviour
Eu7yz	[X]Other mental retardation without mention of impairment of behaviour
Eu7z.	[X]Unspecified mental retardation
Eu7z0	[X]Unspecified mental retardation with the statement of no, or minimal, impairment of behaviour
Eu7z1	[X]Unspecified mental retardation, significant impairment of behaviour requiring attention or treatment
Eu7zy	[X]Unspecified mental retardation, other impairments of behaviour
Eu7zz	[X]Unspecified mental retardation without mention of impairment of behaviour
Eu814	[X]Moderate learning disability
Eu815	[X]Severe learning disability
Eu816	[X]Mild learning disability
Eu817	[X]Profound learning disability
Eu818	[X]Specific learning disability
Eu840	[X]Childhood autism
Eu841	[X]Atypical autism
PJ0..	Down's syndrome - trisomy 21
PJ00.	Trisomy 21, meiotic nondisjunction
PJ01.	Trisomy 21, mosaicism
PJ02.	Trisomy 21, translocation
PJ0z.	Down's syndrome NOS

For peer review only

Hospital

ALT_CODE	DESCRIPTION
F79X	Unspecified mental retardation
Q18.9	Trisomy 21, meiotic nondisjunction
F84.0	Pervasive developmental disorders
Q90	Down syndrome
Q900	Trisomy 21, meiotic nondisjunction
Q901	Trisomy 21, mosaicism (mitotic nondisjunction)
Q902	Trisomy 21, translocation
Q909	Down syndrome, unspecified

Diabetes (GP)

READ_CODE	READ_DESC
C10..	Diabetes mellitus
C100.	Diabetes mellitus with no mention of complication
C1000	Diabetes mellitus, juvenile type, with no mention of complication
C1001	Diabetes mellitus, adult onset, with no mention of complication
C100z	Diabetes mellitus NOS with no mention of complication
C101.	Diabetes mellitus with ketoacidosis
C1010	Diabetes mellitus, juvenile type, with ketoacidosis
C1011	Diabetes mellitus, adult onset, with ketoacidosis
C101y	Other specified diabetes mellitus with ketoacidosis
C101z	Diabetes mellitus NOS with ketoacidosis
C102.	Diabetes mellitus with hyperosmolar coma
C1020	Diabetes mellitus, juvenile type, with hyperosmolar coma
C1021	Diabetes mellitus, adult onset, with hyperosmolar coma
C102z	Diabetes mellitus NOS with hyperosmolar coma
C103.	Diabetes mellitus with ketoacidotic coma
C1030	Diabetes mellitus, juvenile type, with ketoacidotic coma
C1031	Diabetes mellitus, adult onset, with ketoacidotic coma
C103y	Other specified diabetes mellitus with coma
C103z	Diabetes mellitus NOS with ketoacidotic coma
C104.	Diabetes mellitus with renal manifestation
C1040	Diabetes mellitus, juvenile type, with renal manifestation
C1041	Diabetes mellitus, adult onset, with renal manifestation
C104y	Other specified diabetes mellitus with renal complications
C104z	Diabetes mellitus with nephropathy NOS
C105.	Diabetes mellitus with ophthalmic manifestation
C1050	Diabetes mellitus, juvenile type, with ophthalmic manifestation
C1051	Diabetes mellitus, adult onset, with ophthalmic manifestation
C105y	Other specified diabetes mellitus with ophthalmic complications
C105z	Diabetes mellitus NOS with ophthalmic manifestation
C1060	Diabetes mellitus, juvenile type, with neurological manifestation
C1061	Diabetes mellitus, adult onset, with neurological manifestation

For peer review only

C107.	Diabetes mellitus with peripheral circulatory disorder
C1070	Diabetes mellitus, juvenile type, with peripheral circulatory disorder
C1071	Diabetes mellitus, adult onset, with peripheral circulatory disorder
C1072	Diabetes mellitus, adult with gangrene
C1073	IDDM with peripheral circulatory disorder
C1074	NIDDM with peripheral circulatory disorder
C107y	Other specified diabetes mellitus with peripheral circulatory complications
C107z	Diabetes mellitus NOS with peripheral circulatory disorder
C108.	Insulin dependent diabetes mellitus
C1080	Insulin-dependent diabetes mellitus with renal complications
C1081	Insulin-dependent diabetes mellitus with ophthalmic complications
C1082	Insulin-dependent diabetes mellitus with neurological complications
C1083	Insulin dependent diabetes mellitus with multiple complications
C1084	Unstable insulin dependent diabetes mellitus
C1085	Insulin dependent diabetes mellitus with ulcer
C1086	Insulin dependent diabetes mellitus with gangrene
C1087	Insulin dependent diabetes mellitus with retinopathy
C1088	Insulin dependent diabetes mellitus - poor control
C1089	Insulin dependent diabetes maturity onset
C108A	Insulin-dependent diabetes without complication
C108B	Insulin dependent diabetes mellitus with mononeuropathy
C108C	Insulin dependent diabetes mellitus with polyneuropathy
C108D	Insulin dependent diabetes mellitus with nephropathy
C108E	Insulin dependent diabetes mellitus with hypoglycaemic coma
C108F	Insulin dependent diabetes mellitus with diabetic cataract
C108G	Insulin dependent diabetes mellitus with peripheral angiopathy
C108H	Insulin dependent diabetes mellitus with arthropathy
C108J	Insulin dependent diabetes mellitus with neuropathic arthropathy
C108y	Other specified diabetes mellitus with multiple complications
C108z	Unspecified diabetes mellitus with multiple complications
C109.	Non-insulin dependent diabetes mellitus
C1090	Non-insulin-dependent diabetes mellitus with renal complications
C1091	Non-insulin-dependent diabetes mellitus with ophthalmic complications
C1092	Non-insulin-dependent diabetes mellitus with neurological complications
C1093	Non-insulin-dependent diabetes mellitus with multiple complications
C1094	Non-insulin dependent diabetes mellitus with ulcer
C1095	Non-insulin dependent diabetes mellitus with gangrene
C1096	Non-insulin-dependent diabetes mellitus with retinopathy
C1097	Non-insulin dependent diabetes mellitus - poor control
C1099	Non-insulin-dependent diabetes mellitus without complication
C109A	Non-insulin dependent diabetes mellitus with mononeuropathy
C109B	Non-insulin dependent diabetes mellitus with polyneuropathy
C109C	Non-insulin dependent diabetes mellitus with nephropathy
C109D	Non-insulin dependent diabetes mellitus with hypoglycaemic coma
C109E	Non-insulin dependent diabetes mellitus with diabetic cataract
C109F	Non-insulin-dependent diabetes mellitus with peripheral angiopathy
C109G	Non-insulin dependent diabetes mellitus with arthropathy
C109H	Non-insulin dependent diabetes mellitus with neuropathic arthropathy

For peer review only

C109J	Insulin treated Type 2 diabetes mellitus
C109K	Hyperosmolar non-ketotic state in type 2 diabetes mellitus
C10E.	Type 1 diabetes mellitus
C10E0	Type 1 diabetes mellitus with renal complications
C10E1	Type 1 diabetes mellitus with ophthalmic complications
C10E2	Type 1 diabetes mellitus with neurological complications
C10E3	Type 1 diabetes mellitus with multiple complications
C10E4	Unstable type 1 diabetes mellitus
C10E5	Type 1 diabetes mellitus with ulcer
C10E6	Type 1 diabetes mellitus with gangrene
C10E7	Type 1 diabetes mellitus with retinopathy
C10E8	Type 1 diabetes mellitus - poor control
C10E9	Type 1 diabetes mellitus maturity onset
C10EA	Type 1 diabetes mellitus without complication
C10EB	Type 1 diabetes mellitus with mononeuropathy
C10EC	Type 1 diabetes mellitus with polyneuropathy
C10ED	Type 1 diabetes mellitus with nephropathy
C10EE	Type 1 diabetes mellitus with hypoglycaemic coma
C10EF	Type 1 diabetes mellitus with diabetic cataract
C10EG	Type 1 diabetes mellitus with peripheral angiopathy
C10EH	Type 1 diabetes mellitus with arthropathy
C10EJ	Type 1 diabetes mellitus with neuropathic arthropathy
C10EK	Type 1 diabetes mellitus with persistent proteinuria
C10EL	Type 1 diabetes mellitus with persistent microalbuminuria
C10EM	Type 1 diabetes mellitus with ketoacidosis
C10EN	Type 1 diabetes mellitus with ketoacidotic coma
C10EP	Type 1 diabetes mellitus with exudative maculopathy
C10EQ	Type 1 diabetes mellitus with gastroparesis
C10ER	Latent autoimmune diabetes mellitus in adult
C10F.	Type 2 diabetes mellitus
C10F0	Type 2 diabetes mellitus with renal complications
C10F1	Type 2 diabetes mellitus with ophthalmic complications
C10F2	Type 2 diabetes mellitus with neurological complications
C10F3	Type 2 diabetes mellitus with multiple complications
C10F4	Type 2 diabetes mellitus with ulcer
C10F5	Type 2 diabetes mellitus with gangrene
C10F6	Type 2 diabetes mellitus with retinopathy
C10F7	Type 2 diabetes mellitus - poor control
C10F9	Type 2 diabetes mellitus without complication
C10FA	Type 2 diabetes mellitus with mononeuropathy
C10FB	Type 2 diabetes mellitus with polyneuropathy
C10FC	Type 2 diabetes mellitus with nephropathy
C10FD	Type 2 diabetes mellitus with hypoglycaemic coma
C10FE	Type 2 diabetes mellitus with diabetic cataract
C10FF	Type 2 diabetes mellitus with peripheral angiopathy
C10FG	Type 2 diabetes mellitus with arthropathy
C10FH	Type 2 diabetes mellitus with neuropathic arthropathy
C10FJ	Insulin treated Type 2 diabetes mellitus

For peer review only

C10FK	Hyperosmolar non-ketotic state in type 2 diabetes mellitus
C10FL	Type 2 diabetes mellitus with persistent proteinuria
C10FM	Type 2 diabetes mellitus with persistent microalbuminuria
C10FN	Type 2 diabetes mellitus with ketoacidosis
C10FQ	Type 2 diabetes mellitus with exudative maculopathy
C10FR	Type 2 diabetes mellitus with gastroparesis
C10y.	Diabetes mellitus with other specified manifestation
C10y0	Diabetes mellitus, juvenile type, with other specified manifestation
C10y1	Diabetes mellitus, adult onset, with other specified manifestation
C10yy	Other specified diabetes mellitus with other specified complications
C10yz	Diabetes mellitus NOS with other specified manifestation
C10z.	Diabetes mellitus with unspecified complication
C10z0	Diabetes mellitus, juvenile type, with unspecified complication
C10z1	Diabetes mellitus, adult onset, with unspecified complication
C10zy	Other specified diabetes mellitus with unspecified complications
C10zz	Diabetes mellitus NOS with unspecified complication
L180.	Diabetes mellitus during pregnancy, childbirth and the puerperium
L1800	Diabetes mellitus - unspecified whether during pregnancy or the puerperium
L1801	Diabetes mellitus during pregnancy - baby delivered
L1802	Diabetes mellitus in the puerperium - baby delivered during current episode of care
L1803	Diabetes mellitus during pregnancy - baby not yet delivered
L1804	Diabetes mellitus in the pueperium - baby delivered during previous episode of care
L1805	Pre-existing diabetes mellitus, insulin-dependent
L1806	Pre-existing diabetes mellitus, non-insulin-dependent
L1808	Diabetes mellitus arising in pregnancy
L1809	Gestational diabetes mellitus
L180X	Pre-existing diabetes mellitus, unspecified
L180z	Diabetes mellitus during pregnancy, childbirth or the puerperium NOS

Hospital

ALT_CODE	DESCRIPTION
E10	Insulin-dependent diabetes mellitus
E10	Type 1 diabetes mellitus
E100	Insulin-dependent diabetes mellitus
E100	Type 1 diabetes mellitus
E101	Insulin-dependent diabetes mellitus
E101	Type 1 diabetes mellitus
E102	Insulin-dependent diabetes mellitus
E102	Type 1 diabetes mellitus
E103	Insulin-dependent diabetes mellitus
E103	Type 1 diabetes mellitus
E104	Insulin-dependent diabetes mellitus
E104	Type 1 diabetes mellitus

For peer review only

E105	Insulin-dependent diabetes mellitus
E105	Type 1 diabetes mellitus
E106	Insulin-dependent diabetes mellitus
E106	Type 1 diabetes mellitus
E107	Insulin-dependent diabetes mellitus
E107	Type 1 diabetes mellitus
E108	Insulin-dependent diabetes mellitus
E108	Type 1 diabetes mellitus
E109	Insulin-dependent diabetes mellitus
E109	Type 1 diabetes mellitus
E11	Non-insulin-dependent diabetes mellitus
E11	Type 2 diabetes mellitus
E110	Non-insulin-dependent diabetes mellitus
E110	Type 2 diabetes mellitus
E111	Non-insulin-dependent diabetes mellitus
E111	Type 2 diabetes mellitus
E112	Non-insulin-dependent diabetes mellitus
E112	Type 2 diabetes mellitus
E113	Non-insulin-dependent diabetes mellitus
E113	Type 2 diabetes mellitus
E114	Non-insulin-dependent diabetes mellitus
E114	Type 2 diabetes mellitus
E115	Non-insulin-dependent diabetes mellitus
E115	Type 2 diabetes mellitus
E116	Non-insulin-dependent diabetes mellitus
E116	Type 2 diabetes mellitus
E117	Non-insulin-dependent diabetes mellitus
E117	Type 2 diabetes mellitus
E118	Non-insulin-dependent diabetes mellitus
E118	Type 2 diabetes mellitus
E119	Non-insulin-dependent diabetes mellitus
E119	Type 2 diabetes mellitus
E12	Malnutrition-related diabetes mellitus
E120	Malnutrition-related diabetes mellitus
E121	Malnutrition-related diabetes mellitus
E122	Malnutrition-related diabetes mellitus
E123	Malnutrition-related diabetes mellitus
E124	Malnutrition-related diabetes mellitus
E125	Malnutrition-related diabetes mellitus
E126	Malnutrition-related diabetes mellitus
E127	Malnutrition-related diabetes mellitus
E128	Malnutrition-related diabetes mellitus
E129	Malnutrition-related diabetes mellitus
E13	Other specified diabetes mellitus
E130	Other specified diabetes mellitus
E131	Other specified diabetes mellitus
E132	Other specified diabetes mellitus
E133	Other specified diabetes mellitus

For peer review only

E134	Other specified diabetes mellitus
E135	Other specified diabetes mellitus
E136	Other specified diabetes mellitus
E137	Other specified diabetes mellitus
E138	Other specified diabetes mellitus
E139	Other specified diabetes mellitus
E14	Unspecified diabetes mellitus
E140	Unspecified diabetes mellitus
E141	Unspecified diabetes mellitus
E142	Unspecified diabetes mellitus
E143	Unspecified diabetes mellitus
E144	Unspecified diabetes mellitus
E145	Unspecified diabetes mellitus
E146	Unspecified diabetes mellitus
E147	Unspecified diabetes mellitus
E148	Unspecified diabetes mellitus
E149	Unspecified diabetes mellitus
O240	Diabetes mellitus in pregnancy: Pre-existing diabetes mellitus, insulin-dependent
O240	Diabetes mellitus in pregnancy: Pre-existing type 1 diabetes mellitus
O241	Diabetes mellitus in pregnancy: Pre-existing diabetes mellitus, non-insulin-dependent
O241	Diabetes mellitus in pregnancy: Pre-existing type 2 diabetes mellitus
O242	Diabetes mellitus in pregnancy: Pre-existing malnutrition-related diabetes mellitus
O243	Diabetes mellitus in pregnancy: Pre-existing diabetes mellitus, unspecified
O249	Diabetes mellitus in pregnancy, unspecified

Arthritis

READ_CODE	READ_DESC
N....	Musculoskeletal and connective tissue diseases
N0...	Arthropathies and related disorders
N00..	Diffuse diseases of connective tissue
N000.	Systemic lupus erythematosus
N0000	Disseminated lupus erythematosus
N0001	Libman-Sacks disease
N0002	Drug-induced systemic lupus erythematosus
N0003	Systemic lupus erythematosus with organ or system involvement
N0004	Systemic lupus erythematosus with pericarditis
N0005	Neonatal lupus erythematosus
N0006	Cerebral lupus
N000z	Systemic lupus erythematosus NOS
N001.	Scleroderma
N0010	Progressive systemic sclerosis
N0011	CREST syndrome
N0012	Systemic sclerosis induced by drugs and chemicals
N002.	Sicca (Sjogren's) syndrome
N003.	Dermatomyositis

For peer review only

N0030	Juvenile dermatomyositis
N0031	Dermatopolymyositis in neoplastic disease
N003X	Dermatopolymyositis, unspecified
N004.	Polymyositis
N005.	Adult Still's Disease
N006.	Antiphospholipid syndrome
N00y.	Other specified diffuse collagen diseases
N00y0	Eosinophilic fasciitis
N00y1	Fibrosclerosis systemic
N00z.	Collagen disease NOS
N01..	Arthropathy associated with infections
N010.	Pyogenic arthritis
N0100	Pyogenic arthritis of unspecified site
N0101	Pyogenic arthritis of the shoulder region
N0102	Pyogenic arthritis of the upper arm
N0103	Pyogenic arthritis of the forearm
N0104	Pyogenic arthritis of the hand
N0105	Pyogenic arthritis of the pelvic region and thigh
N0106	Pyogenic arthritis of the lower leg
N0107	Pyogenic arthritis of the ankle and foot
N0108	Staphylococcal arthritis and polyarthritis
N0109	Pneumococcal arthritis and polyarthritis
N010A	Arthritis in Lyme disease
N010x	Pyogenic arthritis of multiple sites
N010y	Pyogenic arthritis of other specified sites
N010z	Pyogenic arthritis NOS
N011.	Sexually acquired reactive arthropathy
N0110	Sexually acquired reactive arthropathy of unspecified site
N0111	Sexually acquired reactive arthropathy of the shoulder region
N0112	Sexually acquired reactive arthropathy of the upper arm
N0113	Sexually acquired reactive arthropathy of the forearm
N0114	Sexually acquired reactive arthropathy of the hand
N0115	Sexually acquired reactive arthropathy of the pelvic region and thigh
N0116	Sexually acquired reactive arthropathy of the lower leg
N0117	Sexually acquired reactive arthropathy of the ankle and foot
N011x	Sexually acquired reactive arthropathy of multiple sites
N011y	Sexually acquired reactive arthropathy of other specified site
N011z	Sexually acquired reactive arthropathy NOS
N012.	Arthropathy in Behcet's syndrome
N0120	Arthropathy in Behcet's syndrome of unspecified site
N0121	Arthropathy in Behcet's syndrome of the shoulder region
N0122	Arthropathy in Behcet's syndrome of the upper arm
N0123	Arthropathy in Behcet's syndrome of the forearm
N0124	Arthropathy in Behcet's syndrome of the hand
N0125	Arthropathy in Behcet's syndrome of the pelvic region and thigh
N0126	Arthropathy in Behcet's syndrome of the lower leg
N0127	Arthropathy in Behcet's syndrome of the ankle and foot
N012x	Arthropathy in Behcet's syndrome of multiple sites

For peer review only

N012y	Arthropathy in Behcet's syndrome of other specified sites
N012z	Arthropathy in Behcet's syndrome NOS
N013.	Postdysenteric reactive arthropathy
N0130	Postdysenteric reactive arthropathy of unspecified site
N0131	Postdysenteric reactive arthropathy of the shoulder region
N0132	Postdysenteric reactive arthropathy of the upper arm
N0133	Postdysenteric reactive arthropathy of the forearm
N0134	Postdysenteric reactive arthropathy of the hand
N0135	Postdysenteric reactive arthropathy of the pelvic region and thigh
N0136	Postdysenteric reactive arthropathy of the lower leg
N0137	Postdysenteric reactive arthropathy of the ankle and foot
N013x	Postdysenteric reactive arthropathy of multiple sites
N013y	Postdysenteric reactive arthropathy of other specified sites
N013z	Postdysenteric reactive arthropathy NOS
N014.	Arthropathy associated with other bacterial diseases
N0140	Arthropathy associated with other bacterial disease, of unspecified site
N0141	Arthropathy associated with other bacterial disease, of the shoulder region
N0142	Arthropathy associated with other bacterial disease, of the upper arm
N0143	Arthropathy associated with other bacterial disease, of the forearm
N0144	Arthropathy associated with other bacterial disease, of the hand
N0145	Arthropathy associated with other bacterial disease, of the pelvic region and thigh
N0146	Arthropathy associated with other bacterial disease, of the lower leg
N0147	Arthropathy associated with other bacterial disease, of the ankle and foot
N0148	Arthropathy in Whipple's disease
N014x	Arthropathy associated with other bacterial disease, of multiple sites
N014y	Arthropathy associated with other bacterial disease, of other specified site
N014z	Arthropathy associated with other bacterial disease NOS
N015.	Arthropathy associated with other viral diseases
N0150	Arthropathy associated with other viral disease, of unspecified site
N0151	Arthropathy associated with other viral disease, of the shoulder region
N0152	Arthropathy associated with other viral disease, of the upper arm
N0153	Arthropathy associated with other viral disease, of the forearm
N0154	Arthropathy with other viral disease, of hand
N0155	Arthropathy associated with other viral disease, of the pelvic region and thigh
N0156	Arthropathy associated with other viral disease, of the lower leg
N0157	Arthropathy associated with other viral disease, of the ankle and foot
N015x	Arthropathy associated with other viral disease, of multiple sites
N015y	Arthropathy associated with other viral disease, of other specified site
N015z	Arthropathy associated with other viral disease NOS
N016.	Arthropathy associated with mycoses
N0160	Arthropathy associated with mycoses, of unspecified site
N0161	Arthropathy associated with mycoses, of the shoulder region
N0162	Arthropathy associated with mycoses, of the upper arm
N0163	Arthropathy associated with mycoses, of the forearm
N0164	Arthropathy associated with mycoses, of the hand
N0165	Arthropathy associated with mycoses, of the pelvic region and thigh
N0166	Arthropathy associated with mycoses, of the lower leg
N0167	Arthropathy associated with mycoses, of the ankle and foot

For peer review only

N016x	Arthropathy associated with mycoses, of multiple sites
N016y	Arthropathy associated with mycoses, of other specified site
N016z	Arthropathy associated with mycoses NOS
N017.	Helminthiasis with arthropathy
N0170	Helminthiasis with arthropathy of unspecified site
N0171	Helminthiasis with arthropathy of the shoulder region
N0172	Helminthiasis with arthropathy of the upper arm
N0173	Helminthiasis with arthropathy of the forearm
N0174	Helminthiasis with arthropathy of the hand
N0175	Helminthiasis with arthropathy of the pelvic region and thigh
N0176	Helminthiasis with arthropathy of the lower leg
N0177	Helminthiasis with arthropathy of the ankle and foot
N017x	Helminthiasis with arthropathy of multiple sites
N017y	Helminthiasis with arthropathy of other specified site
N017z	Helminthiasis with arthropathy NOS
N018.	Tuberculous arthritis
N01w.	Reactive arthropathy, unspecified
N01w0	Reactive arthropathy of shoulder
N01w1	Reactive arthropathy of sternoclavicular joint
N01w2	Reactive arthropathy of acromioclavicular joint
N01w3	Reactive arthropathy of elbow
N01w4	Reactive arthropathy of distal radio-ulnar joint
N01w5	Reactive arthropathy of wrist
N01w6	Reactive arthropathy of metacarpophalangeal joint
N01w7	Reactive arthropathy of proximal interphalangeal joint of finger
N01w8	Reactive arthropathy of distal interphalangeal joint of finger
N01w9	Reactive arthropathy of hip
N01wA	Reactive arthropathy of sacro-iliac joint
N01wB	Reactive arthropathy of knee
N01wC	Reactive arthropathy of tibio-fibular joint
N01wD	Reactive arthropathy of ankle
N01wE	Reactive arthropathy of subtalar joint
N01wF	Reactive arthropathy of talonavicular joint
N01wG	Reactive arthropathy of other tarsal joint
N01wH	Reactive arthropathy of 1st metatarsophalangeal joint
N01wJ	Reactive arthropathy of lesser metatarsophalangeal joint
N01wK	Reactive arthropathy of interphalangeal joint of toe
N01y.	Other infectious and parasitic diseases with arthropathy
N01y0	Other infectious and parasitic diseases with arthropathy of unspecified site
N01y1	Other infectious and parasitic diseases with arthropathy of the shoulder region
N01y2	Other infectious and parasitic diseases with arthropathy of the upper arm
N01y3	Other infectious and parasitic diseases with arthropathy of the forearm
N01y4	Other infectious and parasitic diseases with arthropathy of the hand
N01y5	Other infectious and parasitic diseases with arthropathy of the pelvic region and thigh
N01y6	Other infectious and parasitic diseases with arthropathy of the lower leg
N01y7	Other infectious and parasitic diseases with arthropathy of the ankle and foot
N01yx	Other infectious and parasitic diseases with arthropathy of multiple sites

For peer review only

N01yy	Other infectious and parasitic diseases with arthropathy of other specified sites
N01yz	Other infectious and parasitic diseases with arthropathy NOS
N01z.	Infective arthritis NOS
N01z0	Infective arthritis NOS, of unspecified site
N01z1	Infective arthritis NOS, of the shoulder region
N01z2	Infective arthritis NOS, of the upper arm
N01z3	Infective arthritis NOS, of the forearm
N01z4	Infective arthritis NOS, of the hand
N01z5	Infective arthritis NOS, of the pelvic region and thigh
N01z6	Infective arthritis NOS, of the lower leg
N01z7	Infective arthritis NOS, of the ankle and foot
N01z8	Infective arthritis NOS, of shoulder
N01z9	Infective arthritis NOS, of sternoclavicular joint
N01zA	Infective arthritis NOS, of acromioclavicular joint
N01zB	Infective arthritis NOS, of elbow
N01zC	Infective arthritis NOS, of distal radio-ulnar joint
N01zD	Infective arthritis NOS, of wrist
N01zE	Infective arthritis NOS, of metacarpophalangeal joint
N01zF	Infective arthritis NOS, of proximal interphalangeal joint of finger
N01zG	Infective arthritis NOS, of distal interphalangeal joint of finger
N01zH	Infective arthritis NOS, of hip
N01zJ	Infective arthritis NOS, of sacro-iliac joint
N01zK	Infective arthritis NOS, of knee
N01zL	Infective arthritis NOS, of tibio-fibular joint
N01zM	Infective arthritis NOS, of ankle
N01zN	Infective arthritis NOS, of subtalar joint
N01zP	Infective arthritis NOS, of talonavicular joint
N01zQ	Infective arthritis NOS, of other tarsal joint
N01zR	Infective arthritis NOS, of 1st metatarsophalangeal joint
N01zS	Infective arthritis NOS, of lesser metatarsophalangeal joint
N01zT	Infective arthritis NOS, of interphalangeal joint of toe
N01zx	Infective arthritis NOS, of multiple sites
N01zy	Infective arthritis NOS, of other specified site
N01zz	Infective arthritis NOS
N02..	Crystal arthropathies
N020.	Chondrocalcinosis due to dicalcium phosphate crystals
N0200	Chondrocalcinosis due to dicalcium phosphate crystals, of unspecified site
N0201	Chondrocalcinosis due to dicalcium phosphate crystals, of the shoulder region
N0202	Chondrocalcinosis due to dicalcium phosphate crystals, of the upper arm
N0203	Chondrocalcinosis due to dicalcium phosphate crystals, of the forearm
N0204	Chondrocalcinosis due to dicalcium phosphate crystals, of the hand
N0205	Chondrocalcinosis due to dicalcium phosphate crystals, of the pelvic region and thigh
N0206	Chondrocalcinosis due to dicalcium phosphate crystals, of the lower leg
N0207	Chondrocalcinosis due to dicalcium phosphate crystals, of the ankle and foot
N020x	Chondrocalcinosis due to dicalcium phosphate crystals, of multiple sites
N020y	Chondrocalcinosis due to dicalcium phosphate crystals, of other specified site
N020z	Chondrocalcinosis due to dicalcium phosphate crystals, NOS

For peer review only

N021.	Chondrocalcinosis due to pyrophosphate crystals
N0210	Chondrocalcinosis due to pyrophosphate crystals, of unspecified site
N0211	Chondrocalcinosis due to pyrophosphate crystals, of the shoulder region
N0212	Chondrocalcinosis due to pyrophosphate crystals, of the upper arm
N0213	Chondrocalcinosis due to pyrophosphate crystals, of the forearm
N0214	Chondrocalcinosis-pyrophosphate crystals, of the hand
N0215	Chondrocalcinosis due to pyrophosphate crystals, of the pelvic region and thigh
N0216	Chondrocalcinosis due to pyrophosphate crystals, of the lower leg
N0217	Chondrocalcinosis due to pyrophosphate crystals, of the ankle and foot
N021x	Chondrocalcinosis due to pyrophosphate crystals, of multiple sites
N021y	Chondrocalcinosis due to pyrophosphate crystals, of other specified site
N021z	Chondrocalcinosis due to pyrophosphate crystals, NOS
N022.	Chondrocalcinosis, unspecified
N0220	Chondrocalcinosis unspecified, of unspecified site
N0221	Chondrocalcinosis unspecified, of the shoulder region
N0222	Chondrocalcinosis unspecified, of the upper arm
N0223	Chondrocalcinosis unspecified, of the forearm
N0224	Chondrocalcinosis unspecified, of the hand
N0225	Chondrocalcinosis unspecified, of the pelvic region and thigh
N0226	Chondrocalcinosis unspecified, of the lower leg
N0227	Chondrocalcinosis unspecified, of the ankle and foot
N022x	Chondrocalcinosis unspecified, of multiple sites
N022y	Chondrocalcinosis unspecified, of other specified site
N022z	Chondrocalcinosis NOS
N023.	Gouty arthritis
N0230	Gouty arthritis of unspecified site
N0231	Gouty arthritis of the shoulder region
N0232	Gouty arthritis of the upper arm
N0233	Gouty arthritis of the forearm
N0234	Gouty arthritis of the hand
N0235	Gouty arthritis of the pelvic region and thigh
N0236	Gouty arthritis of the lower leg
N0237	Gouty arthritis of the ankle and foot
N0238	Gouty arthritis of toe
N023x	Gouty arthritis of multiple sites
N023y	Gouty arthritis of other specified site
N023z	Gouty arthritis NOS
N024.	Familial chondrocalcinosis
N02y.	Other crystal arthropathies
N02y0	Other crystal arthropathies of unspecified site
N02y1	Other crystal arthropathies of the shoulder
N02y2	Other crystal arthropathies of the upper arm
N02y3	Other crystal arthropathies of the forearm
N02y4	Other crystal arthropathies of the hand
N02y5	Other crystal arthropathies of the pelvic region and thigh
N02y6	Other crystal arthropathies of the lower leg
N02y7	Other crystal arthropathies of the ankle and foot
N02y8	Hydroxyapatite deposition disease

For peer review only

N02yx	Other crystal arthropathies of multiple sites
N02yy	Other crystal arthropathies of other specified sites
N02yz	Other crystal arthropathy NOS
N02z.	Crystal arthropathy NOS
N02z0	Crystal arthropathy NOS, site unspecified
N02z1	Crystal arthropathy NOS, of the shoulder region
N02z2	Crystal arthropathy NOS, of the upper arm
N02z3	Crystal arthropathy NOS, of the forearm
N02z4	Crystal arthropathy NOS, of the hand
N02z5	Crystal arthropathy NOS, of the pelvic region and thigh
N02z6	Crystal arthropathy NOS, of the lower leg
N02z7	Crystal arthropathy NOS, of the ankle and foot
N02z8	Crystal arthropathy NOS, of shoulder
N02z9	Crystal arthropathy NOS, of sternoclavicular joint
N02zA	Crystal arthropathy NOS, of acromioclavicular joint
N02zB	Crystal arthropathy NOS, of elbow
N02zC	Crystal arthropathy NOS, of distal radio-ulnar joint
N02zD	Crystal arthropathy NOS, of wrist
N02zE	Crystal arthropathy NOS, of metacarpophalangeal joint
N02zF	Crystal arthropathy NOS, of proximal interphalangeal joint of finger
N02zG	Crystal arthropathy NOS, of distal interphalangeal joint of finger
N02zH	Crystal arthropathy NOS, of hip
N02zJ	Crystal arthropathy NOS, of sacro-iliac joint
N02zK	Crystal arthropathy NOS, of knee
N02zL	Crystal arthropathy NOS, of tibio-fibular joint
N02zM	Crystal arthropathy NOS, of ankle
N02zN	Crystal arthropathy NOS, of subtalar joint
N02zP	Crystal arthropathy NOS, of talonavicular joint
N02zQ	Crystal arthropathy NOS, of other tarsal joint
N02zR	Crystal arthropathy NOS, of 1st metatarsophalangeal joint
N02zS	Crystal arthropathy NOS, of lesser metatarsophalangeal joint
N02zT	Crystal arthropathy NOS, of interphalangeal joint of toe
N02zx	Crystal arthropathy NOS, of multiple sites
N02zy	Crystal arthropathy NOS, of other specified site
N02zz	Crystal arthropathy NOS
N03..	Arthropathy associated with disorders EC
N030.	Arthropathy associated with endocrine and metabolic disorder
N0300	Diabetic cheiroarthropathy
N0301	Diabetic Charcot arthropathy
N0302	Arthropathy in amyloidosis
N031.	Arthropathy associated with non-infective gastrointestinal disorders
N0310	Arthropathy in ulcerative colitis
N0311	Arthropathy in Crohn's disease
N0312	Arthropathy in Whipple's disease
N0313	Arthropathy following intestinal bypass
N032.	Arthropathy associated with haematological disorders
N0320	Arthropathy due to haemophilia
N033.	Arthropathy associated with dermatological disorders

For peer review only

N034.	Arthropathy associated with respiratory disorders
N035.	Neuropathic arthropathy
N036.	Arthropathy due to hypersensitivity reaction
N037.	Postimmunization arthropathy
N038.	Reactive arthropathies
N0380	Postmeningococcal arthritis
N0381	Postinfective arthropathy in syphilis
N039.	Beta-2 microglobulin arthropathy
N03x.	Other general diseases with associated arthropathy
N03x0	Arthritis associated with other disease, shoulder
N03x1	Arthritis associated with other disease, sternoclavicular joint
N03x2	Arthritis associated with other disease, acromioclavicular joint
N03x3	Arthritis associated with other disease, elbow
N03x4	Arthritis associated with other disease, distal radio-ulnar joint
N03x5	Arthritis associated with other disease, wrist
N03x6	Arthritis associated with other disease, metacarpophalangeal joint
N03x7	Arthritis associated with other disease, proximal interphalangeal joint of finger
N03x8	Arthritis associated with other disease, distal interphalangeal joint of finger
N03x9	Arthritis associated with other disease, hip
N03xA	Arthritis associated with other disease, sacro-iliac joint
N03xB	Arthritis associated with other disease, knee
N03xC	Arthritis associated with other disease, tibio-fibular joint
N03xD	Arthritis associated with other disease, ankle
N03xE	Arthritis associated with other disease, subtalar joint
N03xF	Arthritis associated with other disease, talonavicular joint
N03xG	Arthritis associated with other disease, other tarsal joint
N03xH	Arthritis associated with other disease, 1st metatarsophalangeal joint
N03xJ	Arthritis associated with other disease, lesser metatarsophalangeal joint
N03xK	Arthritis associated with other disease, interphalangeal joint of toe
N03y.	Arthropathy associated with other conditions EC
N03z.	Arthropathy associated with disorders EC NOS
N04..	Rheumatoid arthritis and other inflammatory polyarthropathies
N040.	Rheumatoid arthritis
N0400	Rheumatoid arthritis of cervical spine
N0401	Other rheumatoid arthritis of spine
N0402	Rheumatoid arthritis of shoulder
N0403	Rheumatoid arthritis of sternoclavicular joint
N0404	Rheumatoid arthritis of acromioclavicular joint
N0405	Rheumatoid arthritis of elbow
N0406	Rheumatoid arthritis of distal radio-ulnar joint
N0407	Rheumatoid arthritis of wrist
N0408	Rheumatoid arthritis of metacarpophalangeal joint
N0409	Rheumatoid arthritis of proximal interphalangeal joint of finger
N040A	Rheumatoid arthritis of distal interphalangeal joint of finger
N040B	Rheumatoid arthritis of hip
N040C	Rheumatoid arthritis of sacro-iliac joint
N040D	Rheumatoid arthritis of knee
N040E	Rheumatoid arthritis of tibio-fibular joint

For peer review only

N040F	Rheumatoid arthritis of ankle
N040G	Rheumatoid arthritis of subtalar joint
N040H	Rheumatoid arthritis of talonavicular joint
N040J	Rheumatoid arthritis of other tarsal joint
N040K	Rheumatoid arthritis of 1st metatarsophalangeal joint
N040L	Rheumatoid arthritis of lesser metatarsophalangeal joint
N040M	Rheumatoid arthritis of interphalangeal joint of toe
N040N	Rheumatoid vasculitis
N040P	Seronegative rheumatoid arthritis
N040Q	Rheumatoid bursitis
N040R	Rheumatoid nodule
N040S	Rheumatoid arthritis - multiple joint
N040T	Flare of rheumatoid arthritis
N041.	Felty's syndrome
N042.	Other rheumatoid arthropathy with visceral or systemic involvement
N0420	Rheumatic carditis
N0421	Rheumatoid lung disease
N0422	Rheumatoid nodule
N042z	Rheumatoid arthropathy with visceral or systemic involvement NOS
N043.	Juvenile rheumatoid arthritis - Still's disease
N0430	Juvenile rheumatoid arthropathy unspecified
N0431	Acute polyarticular juvenile rheumatoid arthritis
N0432	Pauciarticular juvenile rheumatoid arthritis
N0433	Monarticular juvenile rheumatoid arthritis
N043z	Juvenile rheumatoid arthritis NOS
N044.	Chronic post-rheumatic arthropathy
N045.	Other juvenile arthritis
N0450	Juvenile ankylosing spondylitis
N0451	Juvenile seronegative polyarthritis
N0452	Juvenile arthritis in psoriasis
N0453	Juvenile arthritis in Crohn's disease
N0454	Juvenile arthritis in ulcerative colitis
N0455	Juvenile rheumatoid arthritis
N0456	Pauciarticular onset juvenile chronic arthritis
N047.	Seropositive errosive rheumatoid arthritis
N04X.	Seropositive rheumatoid arthritis, unspecified
N04y.	Other specified inflammatory polyarthropathy
N04y0	Rheumatoid lung
N04y1	Sero negative arthritis
N04y2	Adult-onset Still's disease
N04y3	Remitting seronegative symmetrical synovitis with pitting oedema
N04yz	Other specified inflammatory polyarthropathy NOS
N04z.	Inflammatory polyarthropathy NOS
N05..	Osteoarthritis and allied disorders
N050.	Generalised osteoarthritis - OA
N0500	Generalised osteoarthritis of unspecified site
N0501	Generalised osteoarthritis of the hand
N0502	Generalised osteoarthritis of multiple sites

For peer review only

N0503	Bouchard's nodes with arthropathy
N0504	Primary generalized osteoarthrosis
N0505	Secondary multiple arthrosis
N0506	Erosive osteoarthrosis
N0507	Heberden's nodes with arthropathy
N050z	Generalised osteoarthritis NOS
N051.	Localised, primary osteoarthritis
N0510	Localised, primary osteoarthritis of unspecified site
N0511	Localised, primary osteoarthritis of the shoulder region
N0512	Localised, primary osteoarthritis of the upper arm
N0513	Localised, primary osteoarthritis of the forearm
N0514	Localised, primary osteoarthritis of the hand
N0515	Localised, primary osteoarthritis of the pelvic region and thigh
N0516	Localised, primary osteoarthritis of the lower leg
N0517	Localised, primary osteoarthritis of the ankle and foot
N0518	Localised, primary osteoarthritis of other specified site
N0519	Primary coxarthrosis, bilateral
N051A	Coxarthrosis resulting from dysplasia, bilateral
N051B	Primary gonarthrosis, bilateral
N051C	Primary arthrosis of first carpometacarpal joints, bilateral
N051D	Localised, primary osteoarthritis of the wrist
N051E	Localised, primary osteoarthritis of toe
N051F	Localised, primary osteoarthritis of elbow
N051G	Osteoarthritis of spinal facet joint
N051z	Localised, primary osteoarthritis NOS
N052.	Localised, secondary osteoarthritis
N0520	Localised, secondary osteoarthritis of unspecified site
N0521	Localised, secondary osteoarthritis of the shoulder region
N0522	Localised, secondary osteoarthritis of the upper arm
N0523	Localised, secondary osteoarthritis of the forearm
N0524	Localised, secondary osteoarthritis of the hand
N0525	Localised, secondary osteoarthritis of the pelvic region and thigh
N0526	Localised, secondary osteoarthritis of the lower leg
N0527	Localised, secondary osteoarthritis of the ankle and foot
N0528	Localised, secondary osteoarthritis of other specified site
N0529	Post-traumatic coxarthrosis, bilateral
N052A	Post-traumatic gonarthrosis, bilateral
N052B	Post-traumatic arthrosis of first carpometacarpal joints, bilateral
N052C	Post-traumatic gonarthrosis, unilateral
N052z	Localised, secondary osteoarthritis NOS
N053.	Localised osteoarthritis, unspecified
N0530	Localised osteoarthritis, unspecified, of unspecified site
N0531	Localised osteoarthritis, unspecified, of the shoulder region
N0532	Localised osteoarthritis, unspecified, of the upper arm
N0533	Localised osteoarthritis, unspecified, of the forearm
N0534	Localised osteoarthritis, unspecified, of the hand
N0535	Localised osteoarthritis, unspecified, of the pelvic region and thigh
N0536	Localised osteoarthritis, unspecified, of the lower leg

For peer review only

N0537	Localised osteoarthritis, unspecified, of the ankle and foot
N0538	Localised osteoarthritis, unspecified, of other specified site
N0539	Arthrosis of first carpometacarpal joint, unspecified
N053z	Localised osteoarthritis, unspecified, NOS
N054.	Oligoarticular osteoarthritis, unspecified
N0540	Oligoarticular osteoarthritis, unspecified, of unspecified sites
N0541	Oligoarticular osteoarthritis, unspecified, of the shoulder region
N0542	Oligoarticular osteoarthritis, unspecified, of upper arm
N0543	Oligoarticular osteoarthritis, unspecified, of forearm
N0544	Oligoarticular osteoarthritis, unspecified, of hand
N0545	Oligoarticular osteoarthritis, unspecified, of the pelvic region and thigh
N0546	Oligoarticular osteoarthritis, unspecified, of lower leg
N0547	Oligoarticular osteoarthritis, unspecified, of ankle and foot
N0548	Oligoarticular osteoarthritis, unspecified, of other specified sites
N0549	Oligoarticular osteoarthritis, unspecified, of multiple sites
N054z	Osteoarthritis of more than one site, unspecified, NOS
N05z.	Osteoarthritis NOS
N05z0	Osteoarthritis NOS, of unspecified site
N05z1	Osteoarthritis NOS, of shoulder region
N05z2	Osteoarthritis NOS, of the upper arm
N05z3	Osteoarthritis NOS, of the forearm
N05z4	Osteoarthritis NOS, of the hand
N05z5	Osteoarthritis NOS, pelvic region/thigh
N05z6	Osteoarthritis NOS, of the lower leg
N05z7	Osteoarthritis NOS, of ankle and foot
N05z8	Osteoarthritis NOS, other specified site
N05z9	Osteoarthritis NOS, of shoulder
N05zA	Osteoarthritis NOS, of sternoclavicular joint
N05zB	Osteoarthritis NOS, of acromioclavicular joint
N05zC	Osteoarthritis NOS, of elbow
N05zD	Osteoarthritis NOS, of distal radio-ulnar joint
N05zE	Osteoarthritis NOS, of wrist
N05zF	Osteoarthritis NOS, of metacarpophalangeal joint
N05zG	Osteoarthritis NOS, of proximal interphalangeal joint of finger
N05zH	Osteoarthritis NOS, of distal interphalangeal joint of finger
N05zJ	Osteoarthritis NOS, of hip
N05zK	Osteoarthritis NOS, of sacro-iliac joint
N05zL	Osteoarthritis NOS, of knee
N05zM	Osteoarthritis NOS, of tibio-fibular joint
N05zN	Osteoarthritis NOS, of ankle
N05zP	Osteoarthritis NOS, of subtalar joint
N05zQ	Osteoarthritis NOS, of talonavicular joint
N05zR	Osteoarthritis NOS, of other tarsal joint
N05zS	Osteoarthritis NOS, of 1st metatarsophalangeal joint
N05zT	Osteoarthritis NOS, of lesser metatarsophalangeal joint
N05zU	Osteoarthritis NOS, of interphalangeal joint of toe
N05zz	Osteoarthritis NOS
N06..	Other and unspecified arthropathies

For peer review only

N060.	Kaschin - Beck disease
N0600	Kaschin-Beck disease of unspecified site
N0601	Kaschin-Beck disease of the shoulder region
N0602	Kaschin-Beck disease of the upper arm
N0603	Kaschin-Beck disease of the forearm
N0604	Kaschin-Beck disease of the hand
N0605	Kaschin-Beck disease of the pelvic region and thigh
N0606	Kaschin-Beck disease of the lower leg
N0607	Kaschin-Beck disease of the ankle and foot
N0608	Kaschin-Beck disease of other specified site
N0609	Kaschin-Beck disease of multiple sites
N060z	Kaschin-Beck disease NOS
N061.	Traumatic arthropathy
N0610	Traumatic arthropathy of unspecified site
N0611	Traumatic arthropathy of the shoulder region
N0612	Traumatic arthropathy of the upper arm
N0613	Traumatic arthropathy of the forearm
N0614	Traumatic arthropathy of the hand
N0615	Traumatic arthropathy of the pelvic region and thigh
N0616	Traumatic arthropathy of the lower leg
N0617	Traumatic arthropathy of the ankle and foot
N0618	Traumatic arthropathy of other specified site
N0619	Traumatic arthropathy of multiple sites
N061A	Traumatic arthropathy of shoulder
N061B	Traumatic arthropathy of sternoclavicular joint
N061C	Traumatic arthropathy of acromioclavicular joint
N061D	Traumatic arthropathy-elbow
N061E	Traumatic arthropathy of distal radio-ulnar joint
N061F	Traumatic arthropathy-wrist
N061G	Traumatic arthropathy of metacarpophalangeal joint
N061H	Traumatic arthropathy of proximal interphalangeal joint of finger
N061J	Traumatic arthropathy of distal interphalangeal joint of finger
N061K	Traumatic arthropathy-hip
N061L	Traumatic arthropathy of sacro-iliac joint
N061M	Traumatic arthropathy-knee
N061N	Traumatic arthropathy of tibio-fibular joint
N061P	Traumatic arthropathy-ankle
N061Q	Traumatic arthropathy of subtalar joint
N061R	Traumatic arthropathy of talonavicular joint
N061S	Traumatic arthropathy of other tarsal joint
N061T	Traumatic arthropathy of 1st metatarsophalangeal joint
N061U	Traumatic arthropathy of lesser metatarsophalangeal joint
N061V	Traumatic arthropathy of interphalangeal joint of toe
N061z	Traumatic arthropathy NOS
N062.	Allergic arthritis
N0620	Allergic arthritis of unspecified site
N0621	Allergic arthritis of the shoulder region
N0622	Allergic arthritis of the upper arm

For peer review only

N0623	Allergic arthritis of the forearm
N0624	Allergic arthritis of the hand
N0625	Allergic arthritis of the pelvic region and thigh
N0626	Allergic arthritis of the lower leg
N0627	Allergic arthritis of the ankle and foot
N0628	Allergic arthritis of other specified site
N0629	Allergic arthritis of multiple sites
N062z	Allergic arthritis NOS
N063.	Climacteric arthritis
N0630	Climacteric arthritis of unspecified site
N0631	Climacteric arthritis of the shoulder region
N0632	Climacteric arthritis of the upper arm
N0633	Climacteric arthritis of the forearm
N0634	Climacteric arthritis of the hand
N0635	Climacteric arthritis of the pelvic region and thigh
N0636	Climacteric arthritis of the lower leg
N0637	Climacteric arthritis of the ankle and foot
N0638	Climacteric arthritis of other specified site
N0639	Climacteric arthritis of multiple sites
N063z	Climacteric arthritis NOS
N064.	Transient arthropathy
N0640	Transient arthropathy of unspecified site
N0641	Transient arthropathy of the shoulder region
N0642	Transient arthropathy of the upper arm
N0643	Transient arthropathy of the forearm
N0644	Transient arthropathy of the hand
N0645	Transient arthropathy of the pelvic region and thigh
N0646	Transient arthropathy of the lower leg
N0647	Transient arthropathy of the ankle and foot
N0648	Transient arthropathy of other specified site
N0649	Transient arthropathy of multiple sites
N064A	Transient arthropathy of shoulder
N064B	Transient arthropathy of sternoclavicular joint
N064C	Transient arthropathy of acromioclavicular joint
N064D	Transient arthropathy-elbow
N064E	Transient arthropathy of distal radio-ulnar joint
N064F	Transient arthropathy-wrist
N064G	Transient arthropathy of metacarpophalangeal joint
N064H	Transient arthropathy of proximal interphalangeal joint of finger
N064J	Transient arthropathy of distal interphalangeal joint of finger
N064K	Transient arthropathy-hip
N064L	Transient arthropathy of sacro-iliac joint
N064M	Transient arthropathy-knee
N064N	Transient arthropathy of tibio-fibular joint
N064P	Transient arthropathy-ankle
N064Q	Transient arthropathy of subtalar joint
N064R	Transient arthropathy of talonavicular joint
N064S	Transient arthropathy of other tarsal joint

For peer review only

N064T	Transient arthropathy of 1st metatarsophalangeal joint
N064U	Transient arthropathy of lesser metatarsophalangeal joint
N064V	Transient arthropathy of interphalangeal joint of toe
N064z	Transient arthropathy NOS
N065.	Unspecified polyarthropathy or polyarthritis
N0650	Unspecified polyarthropathy of unspecified site
N0651	Unspecified polyarthropathy of the shoulder region
N0652	Unspecified polyarthropathy of the upper arm
N0653	Unspecified polyarthropathy of the forearm
N0654	Unspecified polyarthropathy of the hand
N0655	Unspecified polyarthropathy of the pelvic region and thigh
N0656	Unspecified polyarthropathy of the lower leg
N0657	Unspecified polyarthropathy of the ankle and foot
N0658	Unspecified polyarthropathy of other specified site
N0659	Unspecified polyarthropathy of multiple sites
N065A	Generalised arthritis
N065z	Unspecified polyarthropathy or polyarthritis NOS
N066.	Unspecified monoarthritis
N0660	Unspecified monoarthritis of unspecified site
N0661	Unspecified monoarthritis of the shoulder region
N0662	Unspecified monoarthritis of the upper arm
N0663	Unspecified monoarthritis of the forearm
N0664	Unspecified monoarthritis of the hand
N0665	Unspecified monoarthritis of the pelvic region and thigh
N0666	Unspecified monoarthritis of the lower leg
N0667	Unspecified monoarthritis of the ankle and foot
N0668	Unspecified monoarthritis of other specified site
N066z	Unspecified monoarthritis NOS
N067.	Ochronotic arthropathy
N068.	Haemophilic arthropathy
N069.	Arthropathy in neoplastic disease
N06y.	Other specified arthropathy
N06y0	Other specified arthropathy of unspecified site
N06y1	Other specified arthropathy of the shoulder region
N06y2	Other specified arthropathy of the upper arm
N06y3	Other specified arthropathy of the forearm
N06y4	Other specified arthropathy of the hand
N06y5	Other specified arthropathy of the pelvic region and thigh
N06y6	Other specified arthropathy of the lower leg
N06y7	Other specified arthropathy of the ankle and foot
N06y8	Other specified arthropathy of other specified site
N06y9	Other specified arthropathy of multiple sites
N06yz	Other specified arthropathy NOS
N06z.	Arthropathy NOS
N06z0	Arthropathy NOS, of unspecified site
N06z1	Arthropathy NOS, of the shoulder region
N06z2	Arthropathy NOS, of the upper arm
N06z3	Arthropathy NOS, of the forearm

For peer review only

N06z4	Arthropathy NOS, of the hand
N06z5	Arthropathy NOS, of the pelvic region and thigh
N06z6	Arthropathy NOS, of the lower leg
N06z7	Arthropathy NOS, of the ankle and foot
N06z8	Arthropathy NOS, of other specified site
N06z9	Arthropathy NOS, of multiple sites
N06zA	Acute arthritis
N06zB	Chronic arthritis
N06zz	Arthropathy NOS
N07..	Internal derangement of knee
N070.	Medial meniscus derangement
N0700	Medial meniscus derangement, unspecified
N0701	Old bucket handle tear of medial meniscus
N0702	Medial meniscus, anterior horn derangement
N0703	Medial meniscus, posterior horn derangement
N0704	Parrot beak tear of posterior horn of medial meniscus
N0705	Peripheral detachment of medial meniscus
N0706	Radial tear of medial meniscus
N0707	Horizontal cleavage tear of medial meniscus
N0708	Multiple tears of medial meniscus
N0709	Cyst of medial meniscus
N070A	Old tear of medial meniscus
N070B	Old tear of posterior horn of medial meniscus
N070z	Medial meniscus derangement NOS
N071.	Lateral meniscus derangement
N0710	Lateral meniscus derangement unspecified
N0711	Old bucket handle tear of lateral meniscus
N0712	Lateral meniscus, anterior horn derangement
N0713	Lateral meniscus, posterior horn derangement
N0714	Lateral meniscus derangement NOS
N0715	Parrot beak tear of posterior horn of lateral meniscus
N0716	Peripheral detachment of lateral meniscus
N0717	Radial tear of lateral meniscus
N0718	Horizontal cleavage tear of lateral meniscus
N0719	Multiple tears of lateral meniscus
N071A	Cyst of lateral meniscus
N071B	Discoid lateral meniscus
N071C	Old tear of lateral meniscus
N072.	Meniscus derangement NEC
N0720	Old torn meniscus of knee
N0721	Degenerative lesion of articular cartilage of knee
N0722	Cyst of semilunar cartilage
N073.	Loose body in knee
N074.	Chondromalacia patellae
N07y.	Other internal knee derangement
N07y0	Old lateral collateral ligament disruption
N07y1	Old medial collateral ligament disruption
N07y2	Old anterior cruciate ligament disruption

For peer review only

N07y3	Old posterior cruciate ligament disruption
N07y4	Old capsular knee ligament disruption
N07y5	Locked knee
N07y6	Patellofemoral maltracking
N07y7	Old partial tear lateral collateral ligament
N07y8	Old complete tear lateral collateral ligament
N07y9	Old posterolateral capsular complex tear
N07yA	Old partial tear medial collateral ligament
N07yB	Old complete tear medial collateral ligament
N07yC	Old medial capsular complex tear
N07yD	Old partial tear anterior cruciate ligament
N07yE	Old complete tear anterior cruciate ligament
N07yF	Old partial tear posterior cruciate ligament
N07yG	Old complete tear posterior cruciate ligament
N07yH	Locking knee
N07yJ	Adhesions of knee joint
N07yy	Other old knee ligament disruption
N07yz	Other internal knee derangement NOS
N07z.	Internal knee derangement NOS
N08..	Other derangement of joint
N080.	Articular cartilage disorder, excluding the knee
N0800	Articular cartilage disorder, of unspecified site
N0801	Articular cartilage disorder of the shoulder region
N0802	Articular cartilage disorder of the upper arm
N0803	Articular cartilage disorder of the forearm
N0804	Articular cartilage disorder of the hand
N0805	Articular cartilage disorder of the pelvic region and thigh
N0806	Articular cartilage disorder of the ankle and foot
N0807	Articular cartilage disorder of other specified site
N0808	Articular cartilage disorder of multiple sites
N0809	Hill-Sachs lesion
N080A	Reverse Hill-Sachs lesion
N080B	Articular cartilage disorder of other joints of the shoulder girdle
N080C	Chondrolysis of the femoral head
N080z	Articular cartilage disorder NOS
N081.	Loose body in joint, excluding the knee
N0810	Loose body in joint, unspecified joint
N0811	Loose body in joint of shoulder region
N0812	Loose body in joint of upper arm
N0813	Loose body in joint of forearm
N0814	Loose body in joint of hand
N0815	Loose body in joint of pelvic region and thigh
N0816	Loose body in joint of ankle and foot
N0817	Loose body in joint, other specified joint
N0818	Loose joint body in multiple joints
N0819	Loose body in shoulder joint
N081A	Loose body in other joint of shoulder girdle
N081B	Loose body in elbow joint

For peer review only

N081C	Loose body in wrist joint
N081D	Loose body in hip joint
N081E	Loose body in other joint of pelvic girdle
N081F	Loose body in ankle joint
N081G	Loose body in foot joint
N081z	Loose body in joint (excluding the knee) NOS
N082.	Pathological dislocation
N0820	Pathological dislocation of unspecified site
N0821	Pathological dislocation of the shoulder region
N0822	Pathological dislocation of the upper arm
N0823	Pathological dislocation of the forearm
N0824	Pathological dislocation of the hand
N0825	Pathological dislocation of the pelvic region and thigh
N0826	Pathological dislocation of the lower leg
N0827	Pathological dislocation of the ankle and foot
N0828	Pathological dislocation of other specified site
N0829	Pathological dislocation of multiple joints
N082A	Pathological dislocation of the shoulder joint
N082B	Pathological dislocation of other joint of shoulder girdle
N082C	Pathological dislocation of humero-ulnar joint
N082D	Pathological dislocation of superior radio-ulnar joint
N082E	Pathological dislocation of radial head
N082F	Pathological dislocation of inferior radio-ulnar joint
N082G	Pathological dislocation of wrist joint
N082H	Pathological dislocation of 1st carpometacarpal joint
N082J	Pathological dislocation of other carpometacarpal joint
N082K	Pathological dislocation of metacarpophalangeal joint
N082L	Pathological dislocation of proximal interphalangeal joint
N082M	Pathological dislocation of distal interphalangeal joint
N082N	Neuromuscular dislocation of the hip
N082P	Other acquired pathological dislocation of the hip
N082Q	Pathological dislocation of knee joint
N082R	Pathological dislocation of patellofemoral joint
N082S	Pathological dislocation of ankle joint
N082T	Pathological dislocation of subtalar joint
N082U	Pathological dislocation of midtarsal joint
N082V	Pathological dislocation of tarsometatarsal joint
N082W	Pathological dislocation of 1st metatarsophalangeal joint
N082X	Pathological dislocation of lesser metatarsophalangeal joint
N082Y	Pathological dislocation of toe interphalangeal joint
N082Z	Non-traumatic subluxation of acromioclavicular joint
N082z	Pathological dislocation NOS
N083.	Recurrent dislocation of joint
N0830	Recurrent joint dislocation, of unspecified site
N0831	Recurrent joint dislocation, of the shoulder region
N0832	Recurrent joint dislocation, of the upper arm
N0833	Recurrent joint dislocation, of the forearm
N0834	Recurrent joint dislocation, of the hand

For peer review only

N0835	Recurrent joint dislocation, of the pelvic region and thigh
N0836	Recurrent joint dislocation, of the lower leg
N0837	Recurrent joint dislocation, of the ankle and foot
N0838	Recurrent joint dislocation, of other specified site
N0839	Recurrent dislocation of multiple joints
N083A	Recurrent dislocation of shoulder - anterior
N083B	Recurrent dislocation of shoulder - posterior
N083C	Recurrent subluxation of shoulder - anterior
N083D	Recurrent subluxation of shoulder - posterior
N083E	Recurrent dislocation of shoulder - inferior
N083F	Recurrent subluxation of shoulder - inferior
N083G	Recurrent dislocation of shoulder - anterior
N083H	Recurrent subluxation of shoulder - anterior
N083J	Recurrent dislocation of shoulder - multidirectional
N083K	Recurrent subluxation of shoulder - multidirectional
N083L	Habitual dislocation of the shoulder
N083M	Habitual subluxation of the shoulder
N083N	Recurrent dislocation of elbow
N083P	Recurrent subluxation of elbow
N083Q	Recurrent dislocation of superior radio-ulnar joint
N083R	Recurrent subluxation of superior radio-ulnar joint
N083S	Recurrent dislocation of radial head
N083T	Recurrent subluxation of radial head
N083U	Recurrent dislocation of inferior radio-ulnar joint
N083V	Recurrent subluxation of inferior radio-ulnar joint
N083W	Recurrent dislocation of wrist
N083X	Carpal instability
N083Y	Recurrent subluxation of wrist
N083Z	Carpal instability, dorsal intercalated segment instability
N083a	Carpal instability, ventral intercalated segment instability
N083b	Carpal instability, ulnar translocation
N083c	Carpal instability, dorsal subluxation
N083d	Carpal instability, other
N083e	Recurrent dislocation of carpometacarpal joint
N083f	Recurrent subluxation of carpometacarpal joint
N083g	Recurrent dislocation of metacarpophalangeal joint
N083h	Recurrent subluxation of metacarpophalangeal joint
N083j	Recurrent dislocation of interphalangeal joint
N083k	Recurrent subluxation of interphalangeal joint
N083l	Recurrent dislocation of the hip
N083m	Recurrent dislocation of other pelvic joints
N083n	Recurrent dislocation of the knee
N083p	Recurrent dislocation of the patella
N083q	Recurrent subluxation of the patella
N083r	Habitual dislocation of the patella
N083s	Recurrent dislocation of the ankle
N083t	Recurrent subluxation of the ankle
N083u	Recurrent dislocation of foot joint

For peer review only

N083v	Recurrent subluxation of the subtalar joint
N083w	Recurrent subluxation of other foot joint
N083x	Recurrent subluxation of the hip joint
N083z	Recurrent joint dislocation NOS
N084.	Contracture of joint
N0840	Joint contracture of unspecified site
N0841	Joint contracture of the shoulder region
N0842	Joint contracture of the upper arm
N0843	Joint contracture of the forearm
N0844	Joint contracture of the hand
N0845	Joint contracture of the pelvic region and thigh
N0846	Joint contracture of the lower leg
N0847	Joint contracture of the ankle and foot
N0848	Joint contracture of other specified site
N0849	Contracture of multiple joints
N084A	Flexion contracture-shoulder
N084B	Extension contracture-shoulder
N084C	Abduction contracture-shoulder
N084D	Adduction contracture-shoulder
N084E	Internal rotation contracture-shoulder
N084F	External rotation contracture-shoulder
N084G	Flexion contracture - elbow
N084H	Extension contracture - elbow
N084J	Pronation contracture - forearm
N084K	Supination contracture - forearm
N084L	Flexion contracture - wrist
N084M	Extension contracture of the wrist
N084N	Ulnar deviation contracture of the wrist
N084P	Radial deviation contracture of the wrist
N084Q	Flexion contracture of metacarpophalangeal joint
N084R	Extension contracture of metacarpophalangeal joint
N084S	Flexion contracture of proximal interphalangeal joint
N084T	Flexion contracture of distal interphalangeal joint
N084U	Flexion contracture of hip
N084V	Extension contracture of hip
N084W	Abduction contracture of hip
N084X	Adduction contracture of hip
N084Y	Internal rotation contracture of hip
N084Z	External rotation contracture of hip
N084a	Flexion contracture of the knee
N084b	Equinus contracture of the ankle
N084c	Calcaneus contracture of the ankle
N084d	Flexion contracture of metatarsophalangeal joint
N084e	Extension contracture of metatarsophalangeal joint
N084f	Flexion contracture of toe interphalangeal joint
N084g	Extension contracture of toe interphalangeal joint
N084z	Contracture of joint NOS
N085.	Ankylosis of joint

For peer review only

N0850	Joint ankylosis of unspecified site
N0851	Joint ankylosis of the shoulder region
N0852	Joint ankylosis of the upper arm
N0853	Joint ankylosis of the forearm
N0854	Joint ankylosis of the hand
N0855	Joint ankylosis of the pelvic region and thigh
N0856	Joint ankylosis of the lower leg
N0857	Joint ankylosis of the ankle and foot
N0858	Joint ankylosis of other specified site
N0859	Ankylosis of multiple joints
N085A	Ankylosis of the shoulder joint
N085B	Ankylosis of other joint of the shoulder girdle
N085C	Ankylosis of the elbow joint
N085D	Ankylosis of the superior radio-ulnar joint
N085E	Ankylosis of the inferior radio-ulnar joint
N085F	Ankylosis of the wrist joint
N085G	Ankylosis of the 1st carpometacarpal joint
N085H	Ankylosis of other carpometacarpal joint
N085J	Ankylosis of metacarpophalangeal joint
N085K	Ankylosis of proximal interphalangeal joint
N085L	Ankylosis of distal interphalangeal joint
N085M	Ankylosis of the hip joint
N085N	Ankylosis of other pelvic joint
N085P	Ankylosis of the knee joint
N085Q	Ankylosis of the ankle joint
N085R	Ankylosis of the subtalar joint
N085S	Ankylosis of other tarsal joint
N085T	Ankylosis of metatarsophalangeal joint
N085U	Ankylosis of toe joint
N085z	Ankylosis of joint NOS
N086.	Unspecified intrapelvic protrusio acetabuli
N0860	Protrusio acetabuli of unspecified site
N0861	Protrusio acetabuli of the pelvic region and thigh
N086z	Protrusio acetabuli NOS
N087.	Fibrocartilage lesion of joint
N0870	Bankart lesion
N0871	Reverse Bankart lesion
N0872	Glenoid labrum detachment
N0873	Glenoid labrum tear
N0874	Triangular fibrocartilage tear
N0875	Triangular fibrocartilage detachment
N0876	Acetabular labrum detachment
N0877	Acetabular labrum tear
N0878	Snapping shoulder
N08y.	Other joint derangement NEC
N08y0	Other joint derangement NEC, of unspecified site
N08y1	Other joint derangement NEC, of the shoulder region
N08y2	Other joint derangement NEC, of the upper arm

For peer review only

N08y3	Other joint derangement NEC, of the forearm
N08y4	Other joint derangement NEC, of the hand
N08y5	Other joint derangement NEC, of the pelvic region and thigh
N08y6	Other joint derangement NEC, of the lower leg
N08y7	Other joint derangement NEC, of the ankle and foot
N08y8	Other joint derangement NEC, of other specified site
N08y9	Other joint derangement NEC, of multiple sites
N08yA	Flail joint
N08yB	Shoulder joint unstable
N08yz	Other joint derangement NEC, NOS
N08z.	Joint derangement NOS
N08z0	Joint derangement NOS, of unspecified site
N08z1	Joint derangement NOS, of the shoulder region
N08z2	Joint derangement NOS, of the upper arm
N08z3	Joint derangement NOS, of the forearm
N08z4	Joint derangement NOS, of the hand
N08z5	Joint derangement NOS, of the pelvic region and thigh
N08z6	Joint derangement NOS, of the ankle and foot
N08z7	Joint derangement NOS, of other specified site
N08z8	Joint derangement NOS, of multiple sites
N08zz	Joint derangement NOS
N09..	Other and unspecified joint disorders
N090.	Effusion of joint
N0900	Joint effusion of unspecified site
N0901	Joint effusion of the shoulder region
N0902	Joint effusion of the upper arm
N0903	Joint effusion of the forearm
N0904	Joint effusion of the hand
N0905	Joint effusion of the pelvic region and thigh
N0906	Joint effusion of the lower leg
N0907	Joint effusion of the ankle and foot
N0908	Joint effusion of other specified site
N0909	Effusion of multiple joints
N090A	Effusion of shoulder
N090B	Effusion of sternoclavicular joint
N090C	Effusion of acromioclavicular joint
N090D	Effusion of elbow
N090E	Effusion of distal radio-ulnar joint
N090F	Effusion of wrist
N090G	Effusion of metacarpophalangeal joint
N090H	Effusion of proximal interphalangeal joint of finger
N090J	Effusion of distal interphalangeal joint of finger
N090K	Effusion of hip
N090L	Effusion of sacro-iliac joint
N090M	Effusion of knee
N090N	Effusion of tibio-fibular joint
N090P	Effusion of ankle
N090Q	Effusion of subtalar joint

For peer review only

N090R	Effusion of talonavicular joint
N090S	Effusion of other tarsal joint
N090T	Effusion of 1st metatarsophalangeal joint
N090U	Effusion of lesser metatarsophalangeal joint
N090V	Effusion of interphalangeal joint of toe
N090W	Intermittent hydrarthrosis
N090X	Chronic joint effusion
N090Y	Acute joint effusion
N090z	Effusion of joint NOS
N091.	Haemarthrosis
N0910	Haemarthrosis of unspecified site
N0911	Haemarthrosis of the shoulder region
N0912	Haemarthrosis of the upper arm
N0913	Haemarthrosis of the forearm
N0914	Haemarthrosis of the hand
N0915	Haemarthrosis of the pelvic region and thigh
N0916	Haemarthrosis of the lower leg
N0917	Haemarthrosis of the ankle and foot
N0918	Haemarthrosis of other specified site
N0919	Haemarthrosis of multiple joints
N091A	Haemarthrosis of shoulder
N091B	Haemarthrosis of sternoclavicular joint
N091C	Haemarthrosis of acromioclavicular joint
N091D	Haemarthrosis of elbow
N091E	Haemarthrosis of distal radio-ulnar joint
N091F	Haemarthrosis of wrist
N091G	Haemarthrosis of MCP joint
N091H	Haemarthrosis of PIP joint of finger
N091J	Haemarthrosis of DIP joint of finger
N091K	Haemarthrosis of hip
N091L	Haemarthrosis of sacro-iliac joint
N091M	Haemarthrosis of knee
N091N	Haemarthrosis of tibio-fibular joint
N091P	Haemarthrosis of ankle
N091Q	Haemarthrosis of subtalar joint
N091R	Haemarthrosis of talonavicular joint
N091S	Haemarthrosis of other tarsal joint
N091T	Haemarthrosis-1st metatarsophalangeal joint
N091U	Haemarthrosis of lesser metatarsophalangeal joint
N091V	Haemarthrosis of interphalangeal joint of toe
N091z	Haemarthrosis NOS
N092.	Villonodular synovitis
N0920	Villonodular synovitis of unspecified site
N0921	Villonodular synovitis of the shoulder region
N0922	Villonodular synovitis of the upper arm
N0923	Villonodular synovitis of the forearm
N0924	Villonodular synovitis of the hand
N0925	Villonodular synovitis of the pelvic region and thigh

For peer review only

N0926	Villonodular synovitis of the lower leg
N0927	Villonodular synovitis of the ankle and foot
N0928	Villonodular synovitis of other specified site
N0929	Villonodular synovitis of multiple sites
N092A	Villonodular synovitis of shoulder joint
N092B	Villonodular synovitis of sternoclavicular joint
N092C	Villonodular synovitis of acromioclavicular joint
N092D	Villonodular synovitis of elbow
N092E	Villonodular synovitis of distal radio-ulnar joint
N092F	Villonodular synovitis of wrist
N092G	Villonodular synovitis of metacarpophalangeal joint
N092H	Villonodular synovitis of proximal interphalangeal joint of finger
N092J	Villonodular synovitis of distal interphalangeal joint of finger
N092K	Villonodular synovitis of hip
N092L	Villonodular synovitis of sacro-iliac joint
N092M	Villonodular synovitis of knee
N092N	Villonodular synovitis of tibio-fibular joint
N092P	Villonodular synovitis of ankle
N092Q	Villonodular synovitis of subtalar joint
N092R	Villonodular synovitis of talonavicular joint
N092S	Villonodular synovitis of other tarsal joint
N092T	Villonodular synovitis of 1st metatarsophalangeal joint
N092U	Villonodular synovitis of lesser metatarsophalangeal joint
N092V	Villonodular synovitis of interphalangeal joint of toe
N092z	Villonodular synovitis NOS
N093.	Palindromic rheumatism
N0930	Palindromic rheumatism of unspecified site
N0931	Palindromic rheumatism of the shoulder region
N0932	Palindromic rheumatism of the upper arm
N0933	Palindromic rheumatism of the forearm
N0934	Palindromic rheumatism of the hand
N0935	Palindromic rheumatism of the pelvic region and thigh
N0936	Palindromic rheumatism of the lower leg
N0937	Palindromic rheumatism of the ankle and foot
N0938	Palindromic rheumatism of other specified site
N0939	Palindromic rheumatism of multiple sites
N093z	Palindromic rheumatism NOS
N094.	Pain in joint - arthralgia
N0940	Arthralgia of unspecified site
N0941	Arthralgia of the shoulder region
N0942	Arthralgia of the upper arm
N0943	Arthralgia of the forearm
N0944	Arthralgia of the hand
N0945	Arthralgia of the pelvic region and thigh
N0946	Arthralgia of the lower leg
N0947	Arthralgia of the ankle and foot
N0948	Arthralgia of other specified site
N0949	Arthralgia of multiple joints

For peer review only

N094A	Arthralgia of shoulder
N094B	Arthralgia of sternoclavicular joint
N094C	Arthralgia of acromioclavicular joint
N094D	Arthralgia of elbow
N094E	Arthralgia of distal radio-ulnar joint
N094F	Arthralgia of wrist
N094G	Arthralgia of metacarpophalangeal joint
N094H	Arthralgia of proximal interphalangeal joint of finger
N094J	Arthralgia of distal interphalangeal joint of finger
N094K	Arthralgia of hip
N094L	Arthralgia of sacro-iliac joint
N094M	Arthralgia of knee
N094N	Arthralgia of tibio-fibular joint
N094P	Arthralgia of ankle
N094Q	Arthralgia of subtalar joint
N094R	Arthralgia of talonavicular joint
N094S	Arthralgia of other tarsal joint
N094T	Arthralgia of 1st metatarsophalangeal joint
N094U	Arthralgia of lesser metatarsophalangeal joint
N094V	Arthralgia of interphalangeal joint of toe
N094W	Anterior knee pain
N094z	Arthralgia NOS
N095.	Joint stiffness NEC
N0950	Stiff joint NEC, of unspecified site
N0951	Stiff joint NEC, of the shoulder region
N0952	Stiff joint NEC, of the upper arm
N0953	Stiff joint NEC, of the forearm
N0954	Stiff joint NEC, of the hand
N0955	Stiff joint NEC, of the pelvic region and thigh
N0956	Stiff joint NEC, of the lower leg
N0957	Stiff joint NEC, of the ankle and foot
N0958	Stiff joint NEC, of other specified site
N0959	Multiple stiff joints
N095A	Stiff shoulder NEC
N095B	Stiff sternoclavicular joint NEC
N095C	Stiff acromioclavicular joint NEC
N095D	Stiff elbow NEC
N095E	Stiff distal radio-ulnar joint NEC
N095F	Stiff wrist NEC
N095G	Stiff metacarpophalangeal joint NEC
N095H	Stiff proximal interphalangeal joint of finger NEC
N095J	Stiff distal interphalangeal joint of finger NEC
N095K	Stiff hip NEC
N095L	Stiff sacro-iliac joint NEC
N095M	Stiff knee NEC
N095N	Stiff tibio-fibular joint NEC
N095P	Stiff ankle NEC
N095Q	Stiff subtalar joint NEC

For peer review only

N095R	Stiff talonavicular joint NEC
N095S	Stiff other tarsal joint NEC
N095T	Stiff 1st metatarsophalangeal joint NEC
N095U	Stiff lesser metatarsophalangeal joint NEC
N095V	Stiff interphalangeal joint of toe NEC
N095W	Stiff finger
N095z	Joint stiffness NEC, NOS
N096.	Other joint symptoms
N0960	Other joint symptoms of unspecified site
N0961	Other joint symptoms of the shoulder region
N0962	Other joint symptoms of the upper arm
N0963	Other joint symptoms of the forearm
N0964	Other joint symptoms of the hand
N0965	Other joint symptoms of the pelvic region and thigh
N0966	Other joint symptoms of the lower leg
N0967	Other joint symptoms of the ankle and foot
N0968	Other joint symptoms of other specified site
N0969	Other joint symptoms of multiple sites
N096A	Other symptoms - shoulder
N096B	Other symptoms in sternoclavicular joint
N096C	Other symptoms in acromioclavicular joint
N096D	Other symptoms - elbow
N096E	Other symptoms in distal radio-ulnar joint
N096F	Other symptoms - wrist
N096G	Other symptoms - MCPJ
N096H	Other symptoms in proximal interphalangeal joint of finger
N096J	Other symptoms in distal interphalangeal joint of finger
N096K	Other symptoms - hip
N096L	Other symptoms in sacro-iliac joint
N096M	Other symptoms - knee
N096N	Other symptoms in tibio-fibular joint
N096P	Other symptoms - ankle
N096Q	Other symptoms in subtalar joint
N096R	Other symptoms in talonavicular joint
N096S	Other symptoms in other tarsal joint
N096T	Other symptoms in 1st metatarsophalangeal joint
N096U	Other symptoms in lesser metatarsophalangeal joint
N096V	Other symptoms in interphalangeal joint of toe
N096z	Other joint symptoms NOS
N097.	Difficulty in walking
N0970	Walking difficulty due to unspecified site
N0971	Walking difficulty due to pelvic region and thigh
N0972	Walking difficulty due to lower leg
N0973	Walking difficulty due to ankle and foot
N0974	Walking difficulty due to other specified site
N0975	Walking difficulty due to multiple sites
N097z	Difficulty in walking NOS
N098.	Synovial osteochondromatosis

For peer review only

N0980	Synovial osteochondromatosis of shoulder
N0981	Synovial osteochondromatosis of sternoclavicular joint
N0982	Synovial osteochondromatosis of acromioclavicular joint
N0983	Synovial osteochondromatosis of elbow
N0984	Synovial osteochondromatosis of distal radio-ulnar joint
N0985	Synovial osteochondromatosis of wrist
N0986	Synovial osteochondromatosis of metacarpophalangeal joint
N0987	Synovial osteochondromatosis of proximal interphalangeal joint of finger
N0988	Synovial osteochondromatosis of distal interphalangeal joint of finger
N0989	Synovial osteochondromatosis of hip
N098A	Synovial osteochondromatosis of sacro-iliac joint
N098B	Synovial osteochondromatosis of knee
N098C	Synovial osteochondromatosis of tibio-fibular joint
N098D	Synovial osteochondromatosis of ankle
N098E	Synovial osteochondromatosis of subtalar joint
N098F	Synovial osteochondromatosis of talonavicular joint
N098G	Synovial osteochondromatosis of other tarsal joint
N098H	Synovial osteochondromatosis of 1st metatarsophalangeal joint
N098J	Synovial osteochondromatosis of lesser metatarsophalangeal joint
N098K	Synovial osteochondromatosis of interphalangeal joint of toe
N099.	Clicking joint
N0990	Clicking shoulder
N0991	Clicking sternoclavicular joint
N0992	Clicking acromioclavicular joint
N0993	Clicking elbow
N0994	Clicking distal radio-ulnar joint
N0995	Clicking wrist
N0996	Clicking metacarpophalangeal joint
N0997	Clicking proximal interphalangeal joint of finger
N0998	Clicking distal interphalangeal joint of finger
N0999	Clicking hip
N099A	Multiple clicking joints
N099B	Clicking sacro-iliac joint
N099C	Clicking knee
N099D	Clicking tibio-fibular joint
N099E	Clicking ankle
N099F	Clicking subtalar joint
N099G	Clicking talonavicular joint
N099H	Clicking other tarsal joint
N099J	Clicking 1st metatarsophalangeal joint
N099K	Clicking lesser metatarsophalangeal joint
N099L	Clicking interphalangeal joint of toe
N09A.	Patellofemoral disorder
N09AX	Disorder of patella, unspecified
N09B.	Osteophyte
N09C.	Fistula of joint
N09y.	Other specified joint disorders
N09y0	Other specified joint disorders of unspecified site

For peer review only

N09y1	Other specified joint disorders of the shoulder region
N09y2	Other specified joint disorders of the upper arm
N09y3	Other specified joint disorders of the forearm
N09y4	Other specified joint disorders of the hand
N09y5	Other specified joint disorders of the pelvic region and thigh
N09y6	Other specified joint disorders of the lower leg
N09y7	Other specified joint disorders of the ankle and foot
N09y8	Other specified joint disorders of other specified site
N09y9	Other specified joint disorders of multiple sites
N09yz	Other specified joint disorders NOS
N09z.	Joint disorders NOS
N09z0	Joint disorder NOS, of unspecified site
N09z1	Joint disorder NOS, of shoulder region
N09z2	Joint disorder NOS, of the upper arm
N09z3	Joint disorder NOS, of the forearm
N09z4	Joint disorder NOS, of the hand
N09z5	Joint disorder NOS, of the pelvic region and thigh
N09z6	Joint disorder NOS, of the lower leg
N09z7	Joint disorder NOS, of ankle and foot
N09z8	Joint disorder NOS, of other specified site
N09z9	Joint disorder NOS, of multiple sites
N09zz	Joint disorders NOS
N0y..	Other specified arthropathies
N0z..	Arthropathies NOS
N1...	Vertebral column syndromes
N10..	Inflammatory spondylopathies
N100.	Ankylosing spondylitis
N101.	Spinal enthesopathy
N102.	Sacroiliitis NEC
N10y.	Other inflammatory spondylopathies
N10y0	Inflammatory spondylopathies in diseases EC
N10yz	Other inflammatory spondylopathies NOS
N10z.	Spondylitis NOS
N11..	Spondylosis and allied disorders
N110.	Cervical spondylosis without myelopathy
N1100	Single-level cervical spondylosis without myelopathy
N1101	Two-level cervical spondylosis without myelopathy
N1102	Multiple-level cervical spondylosis without myelopathy
N111.	Cervical spondylosis with myelopathy
N1110	Single-level cervical spondylosis with myelopathy
N1111	Two-level cervical spondylosis with myelopathy
N1112	Multiple-level cervical spondylosis with myelopathy
N1113	Cervical myelopathy
N112.	Thoracic spondylosis without myelopathy
N1120	Single-level thoracic spondylosis without myelopathy
N1121	Two-level thoracic spondylosis without myelopathy
N1122	Multiple-level thoracic spondylosis without myelopathy
N1123	Dorsal spondylosis without myelopathy

For peer review only

N113.	Thoracic spondylosis with myelopathy
N1130	Single-level thoracic spondylosis with myelopathy
N1131	Two-level thoracic spondylosis with myelopathy
N1132	Multiple-level thoracic spondylosis with myelopathy
N114.	Lumbosacral spondylosis without myelopathy
N1140	Single-level lumbosacral spondylosis without myelopathy
N1141	Two-level lumbosacral spondylosis without myelopathy
N1142	Multiple-level lumbosacral spondylosis without myelopathy
N115.	Lumbosacral spondylosis with myelopathy
N1150	Single-level lumbosacral spondylosis with myelopathy
N1151	Two-level lumbosacral spondylosis with myelopathy
N1152	Multiple-level lumbosacral spondylosis with myelopathy
N116.	Kissing spine
N117.	Ankylosing vertebral hyperostosis
N118.	Traumatic spondylopathy
N119.	Cervical spondylosis with radiculopathy
N1190	Single-level cervical spondylosis with radiculopathy
N1191	Two-level cervical spondylosis with radiculopathy
N1192	Multiple-level cervical spondylosis with radiculopathy
N11A.	Cervical spondylosis with vascular compression
N11B.	Thoracic spondylosis with radiculopathy
N11B0	Single-level thoracic spondylosis with radiculopathy
N11B1	Two-level thoracic spondylosis with radiculopathy
N11B2	Multiple-level thoracic spondylosis with radiculopathy
N11C.	Lumbosacral spondylosis with radiculopathy
N11C0	Single-level lumbosacral spondylosis with radiculopathy
N11C1	Two-level lumbosacral spondylosis with radiculopathy
N11C2	Multiple-level lumbosacral spondylosis with radiculopathy
N11D.	Osteoarthritis of spine
N11D0	Osteoarthritis of cervical spine
N11D1	Osteoarthritis of thoracic spine
N11D2	Osteoarthritis of lumbar spine
N11D3	Osteoarthritis of spine NOS
N11E.	Cervical spondylosis
N11F.	Axial spondyloarthritis
N11y.	Other spondyloses and allied disorders
N11y0	Brucella spondylitis
N11y1	Enterobacterial spondylitis
N11y2	Neuropathic spondylopathy
N11z.	Spondylosis NOS
N11z0	Spondylosis without myelopathy, NOS
N11z1	Spondylosis with myelopathy, NOS
N11zz	Spondylosis NOS
N12..	Intervertebral disc disorders
N120.	Cervical disc displacement without myelopathy
N121.	Thoracic disc displacement without myelopathy
N122.	Lumbar disc displacement
N123.	Disc displacement, site unspecified, without myelopathy

For peer review only

N124.	Schmorl's nodes
N1240	Schmorl's nodes of unspecified region
N1241	Schmorl's nodes of the thoracic region
N1242	Schmorl's nodes of the lumbar region
N124z	Schmorl's nodes, region NOS
N125.	Cervical disc degeneration
N126.	Thoracic disc degeneration
N127.	Lumbar disc degeneration
N128.	Degenerative disc disease NOS
N129.	Disc disorder with myelopathy
N1290	Unspecified disc disorder with myelopathy
N1291	Cervical disc disorder with myelopathy
N1292	Thoracic disc disorder with myelopathy
N1293	Lumbar disc disorder with myelopathy
N129z	Disc disorder with myelopathy NOS
N12A.	Postlaminectomy syndrome
N12A0	Postlaminectomy syndrome of unspecified site
N12A1	Cervical postlaminectomy syndrome
N12A2	Thoracic postlaminectomy syndrome
N12A3	Lumbar postlaminectomy syndrome
N12Az	Postlaminectomy syndrome NOS
N12B.	Disc prolapse with myelopathy
N12B0	Cervical disc prolapse with myelopathy
N12B1	Thoracic disc prolapse with myelopathy
N12B2	Lumbar disc prolapse with myelopathy
N12C.	Disc prolapse with radiculopathy
N12C0	Cervical disc prolapse with radiculopathy
N12C1	Thoracic disc prolapse with radiculopathy
N12C2	Lumbar disc prolapse with radiculopathy
N12C3	Lumbar disc prolapse with cauda equina compression
N12C4	Prolapsed lumbar intervertebral disc with sciatica
N12D.	Narrowing intervertebral disc space
N12z.	Other and unspecified disc disorders
N12z0	Other disc disorders of unspecified site
N12z1	Other cervical disc disorders
N12z2	Other thoracic disc disorders
N12z3	Other lumbar disc disorders
N12z4	Cervical discitis
N12z5	Annular tear of cervical disc
N12z6	Resorption of cervical disc
N12z7	Calcification of cervical disc
N12z8	Thoracic discitis
N12z9	Annular tear of thoracic disc
N12zA	Resorption of thoracic disc
N12zB	Calcification of thoracic disc
N12zC	Lumbar discitis
N12zD	Annular tear of lumbar disc
N12zE	Resorption of lumbar disc

For peer review only

N12zF	Calcification of lumbar disc
N12zG	Infection of intervertebral disc - pyogenic
N12zH	Cervical disc disorder with radiculopathy
N12zz	Disc disorders NOS
N13..	Other cervical disorders
N130.	Cervical spinal stenosis
N1300	Idiopathic cervical spinal stenosis
N1301	Degenerative cervical spinal stenosis
N1302	Iatrogenic cervical spinal stenosis
N1303	Cervical spinal stenosis secondary to other disease
N131.	Cervicalgia - pain in neck
N132.	Cervicocranial syndrome
N133.	Cervicobrachial syndrome
N134.	Brachial (cervical) neuritis
N135.	Torticollis unspecified
N1350	Intermittent torticollis
N1351	Rheumatic torticollis
N135z	Torticollis NOS
N136.	Panniculitis of neck
N137.	Cervical posterior longitudinal ligament ossification
N138.	Cervicalgia
N13y.	Other cervical syndromes
N13y0	Cervical syndrome NEC
N13y1	Klippel's disease
N13y2	Crick in neck
N13y3	Cervical root syndrome
N13yz	Other cervical syndromes NOS
N13z.	Cervical and neck disorders NOS
N14..	Other and unspecified back disorders
N140.	Spinal stenosis, excluding cervical region
N1400	Spinal stenosis of unspecified region
N1401	Thoracic spinal stenosis
N1402	Lumbar spinal stenosis
N1403	Idiopathic thoracic spinal stenosis
N1404	Degenerative thoracic spinal stenosis
N1405	Iatrogenic thoracic spinal stenosis
N1406	Thoracic spinal stenosis secondary to other disease
N1407	Idiopathic lumbar spinal stenosis
N1408	Degenerative lumbar spinal stenosis
N1409	Iatrogenic lumbar spinal stenosis
N140A	Lumbar spinal stenosis secondary to other disease
N140z	Spinal stenosis NOS
N141.	Pain in thoracic spine
N142.	Pain in lumbar spine
N1420	Lumbago with sciatica
N143.	Sciatica
N144.	Thoracic and lumbosacral neuritis
N1440	Thoracic neuritis, unspecified

For peer review only

N1441	Lumbosacral neuritis, unspecified
N144z	Thoracic and lumbosacral neuritis NOS
N145.	Backache, unspecified
N146.	Disorders of the sacrum
N1460	Lumbosacral ankylosis
N1461	Sacroiliac ankylosis
N1462	Sacral ankylosis NOS
N1463	Lumbosacral instability
N1464	Sacroiliac instability
N1465	Sacral instability NOS
N1466	Sacroiliac disorder
N146z	Disorders of the sacrum NOS
N147.	Disorders of the coccyx
N1470	Unspecified disorder of the coccyx
N1471	Hypermobility of the coccyx
N1472	Coccygodynia
N147z	Coccyx disorder NOS
N148.	Ankylosis or instability of cervical,thoracic or lumbar spine
N1480	Atlanto-occipital ankylosis
N1481	Atlanto-axial ankylosis
N1482	Cervical spine ankylosis
N1483	Cervico-thoracic ankylosis
N1484	Thoracic spine ankylosis
N1485	Thoraco-lumbar ankylosis
N1486	Lumbar spine ankylosis
N1487	Atlanto-occipital instability
N1488	Atlanto-axial instability
N1489	Cervical spine instability
N148A	Cervico-thoracic instability
N148B	Thoracic spine instability
N148C	Lumbar spine instability
N149.	Back stiffness
N14A.	Neurogenic claudication
N14X.	Sacrococcygeal disorders, not elsewhere classified
N14y.	Other back symptoms
N14z.	Back disorders NOS
N1y..	Other specified disorders of vertebral column
N1y0.	Recurrent atlantoaxial subluxation with myelopathy
N1y1.	Fatigue fracture of vertebra
N1y2.	Pars interarticularis stress fracture
N1z..	Vertebral column disorder NOS
N2...	Rheumatism, excluding the back
N20..	Polymyalgia rheumatica
N200.	Giant cell arteritis with polymyalgia rheumatica
N21..	Peripheral enthesopathies and allied syndromes
N210.	Adhesive capsulitis of the shoulder
N211.	Rotator cuff shoulder syndrome and allied disorders
N2110	Rotator cuff syndrome, unspecified

For peer review only

For peer review only - http://bmjopen.bmj.com/site/about/guidelines.xhtml

N2111	Calcifying tendinitis of the shoulder
N2112	Bicipital tenosynovitis
N2113	Supraspinatus tendinitis
N2114	Partial thickness rotator cuff tear
N2115	Full thickness rotator cuff tear
N2116	Subacromial bursitis
N2117	Subdeltoid bursitis
N2118	Bursitis of shoulder
N211z	Rotator cuff syndrome NOS
N212.	Other shoulder affections NEC
N2120	Periarthritis of shoulder
N2121	Scapulohumeral fibrositis
N2122	Subacromial impingement
N2123	Coracoid impingement
N2124	Impingement syndrome of shoulder
N2125	Shoulder tendonitis
N212z	Other shoulder affections NEC, NOS
N213.	Enthesopathy of the elbow region
N2130	Elbow enthesopathy unspecified
N2131	Medial epicondylitis of the elbow
N2132	Lateral epicondylitis of the elbow
N2133	Olecranon bursitis
N2134	Biceps tendinitis
N2135	Triceps tendinitis
N213z	Elbow enthesopathy NOS
N214.	Enthesopathy of the wrist and carpus
N2140	Bursitis of wrist
N2141	Bursitis of hand
N2142	Periarthritis of wrist
N2143	Carpometacarpal bossing
N214z	Wrist or carpus enthesopathy NOS
N215.	Enthesopathy of the hip region
N2150	Hip enthesopathy, unspecified
N2151	Bursitis of hip
N2152	Gluteal tendinitis
N2153	Iliac crest spur
N2154	Psoas tendinitis
N2155	Trochanteric tendinitis
N2156	Adductor tendinitis
N2157	Trochanteric bursitis
N2158	Snapping hip
N2159	Iliotibial band syndrome
N215A	Ischial bursitis
N215B	Femoroacetabular impingement
N215z	Hip enthesopathy NOS
N216.	Enthesopathy of the knee
N2160	Bursitis of the knee NOS
N2161	Pes anserinus tendinitis and bursitis

For peer review only

N2162	Tibial collateral ligament bursitis
N2163	Fibular collateral ligament bursitis
N2164	Patellar tendinitis
N2165	Prepatellar bursitis
N2166	Infrapatellar bursitis
N2167	Subpatellar bursitis
N2168	Biceps femoris tendinitis
N2169	Semimembranosus tendinitis
N216z	Knee enthesopathy NOS
N217.	Enthesopathy of the ankle and tarsus
N2170	Enthesopathy of the ankle unspecified
N2171	Enthesopathy of the tarsus unspecified
N2172	Metatarsalgia NOS
N2173	Achilles bursitis
N2174	Achilles tendinitis
N2175	Tibialis anterior tendinitis
N2176	Tibialis posterior tendinitis
N2177	Calcaneal spur
N2178	Peroneal tendinitis
N2179	Plantar fasciitis
N217A	Posterior calcaneal exostosis (pump bump)
N217B	Anterior ankle impingement
N217C	Fibular impingement
N217z	Ankle or tarsus enthesopathy NOS
N21y.	Other peripheral enthesopathies
N21y0	Anterior shin splints
N21y1	Posterior shin splints
N21z.	Enthesopathy NOS
N21z0	Capsulitis NOS
N21z1	Periarthritis NOS
N21z2	Tendinitis NOS
N21z3	Exostosis of unspecified site
N21z4	Subungual exostosis
N21z5	Subungual exostosis of great toe
N21z6	Subungual exostosis of lesser toe
N21z7	Exostosis
N21zz	Peripheral enthesopathy NOS
N22..	Other disorders of the synovium, tendon and bursa
N220.	Synovitis and tenosynovitis
N2200	Synovitis or tenosynovitis NOS
N2201	Synovitis and tenosynovitis with disorders EC
N2202	Tendon sheath giant cell tumor
N2203	Trigger finger - acquired
N2204	Radial styloid tenosynovitis
N2205	Other tenosynovitis of hand or wrist
N2206	Tenosynovitis of ankle
N2207	Tenosynovitis of foot
N2208	Villonodular synovitis of tendon sheath

For peer review only

N2209	Plant thorn synovitis
N220A	Flexor tenosynovitis of wrist
N220B	Flexor tenosynovitis of finger
N220C	Flexor tenosynovitis of thumb
N220D	Extensor tenosynovitis of wrist
N220E	Extensor tenosynovitis of finger
N220F	Extensor tenosynovitis of thumb
N220G	Acquired trigger thumb
N220H	Achilles tenosynovitis
N220J	Tibialis anterior tenosynovitis
N220K	Tibialis posterior tenosynovitis
N220L	Extensor hallucis longus tenosynovitis
N220M	Extensor digitorum longus tenosynovitis
N220N	Peroneus longus tenosynovitis
N220P	Peroneus brevis tenosynovitis
N220Q	Transient synovitis
N220R	Chronic crepitant synovitis of hand and wrist
N220S	Synovitis of hip
N220T	Synovitis NOS
N220V	Synovitis of knee
N220W	Synovitis of elbow
N220X	Synovitis of shoulder
N220Y	Irritable hip
N220z	Other synovitis and tenosynovitis
N221.	Bunion
N2210	Infected bunion
N222.	Specific bursitides
N2220	Beat elbow
N2221	Beat hand
N2222	Beat knee
N2223	Miners' elbow
N2224	Miners' knee
N2225	Housemaids' knee
N2226	Calcium deposit in bursa
N2227	Syphilitic bursitis
N222z	Specific bursitides NOS
N223.	Bursitis NOS
N224.	Ganglion and cyst of synovium, tendon and bursa
N2240	Synovial cyst unspecified
N2241	Ganglion of joint
N2242	Ganglion of tendon sheath
N2243	Ganglion unspecified
N2244	Cyst of bursa
N2245	Ganglion of wrist
N2246	Ganglion of knee
N2247	Ganglion of superior tibio-fibular joint
N2248	Ganglion of ankle
N2249	Ganglion of flexor tendon sheath of finger

For peer review only

N224A	Synovial cyst of popliteal space
N224B	Ganglion of hand
N224C	Ganglion of foot
N224D	Cyst of tendon sheath
N224E	Digital mucous cyst
N224z	Ganglion or cyst of synovium/tendon/bursa NOS
N225.	Rupture of synovium
N2250	Rupture of synovium, unspecified
N2251	Rupture of popliteal space synovial cyst
N225z	Rupture of synovium NOS
N226.	Nontraumatic tendon rupture
N2260	Nontraumatic tendon rupture, unspecified
N2261	Rotator cuff complete rupture
N2262	Biceps tendon rupture
N2263	Hand and wrist extensor tendon rupture
N2264	Hand and wrist flexor tendon rupture
N2265	Quadriceps tendon rupture
N2266	Nontraumatic rupture of patellar tendon
N2267	Nontraumatic rupture of Achilles tendon
N2268	Extensor digitorum communis rupture
N2269	Extensor pollicis longus rupture
N226A	Long head of biceps rupture
N226B	Subluxation of long head of biceps
N226C	Flexor digitorum sublimis tendon rupture
N226D	Flexor digitorum profundus tendon rupture
N226E	Flexor pollicis longus tendon rupture
N226F	Tibialis posterior rupture
N226G	Peroneus longus rupture
N226H	Subluxation of peroneal tendon
N226J	Subluxation of tendon, wrist or hand
N226K	Dislocation of tendon, wrist or hand
N226L	Bowstringing of tendon, wrist or hand
N226M	Spontaneous rupture of flexor tendons
N226N	Spontaneous rupture of extensor tendons
N226y	Other foot and ankle tendon rupture
N226z	Other nontraumatic tendon rupture
N227.	Posterior tibial tendon insufficiency
N228.	Tendinous xanthoma
N22y.	Other synovial, tendon and bursa disorders
N22y0	Contracture of tendon sheath
N22y1	Calcification of tendon NOS
N22y2	Abscess of tendon
N22y3	Abscess of bursa
N22y4	Synovial plica
N22y5	Short tendon
N22y6	Abscess of tendon-arm
N22y7	Abscess of tendon-forearm
N22y8	Abscess of tendon-hand

For peer review only

N22y9	Abscess of tendon-thigh
N22yA	Abscess of tendon-leg
N22yB	Abscess of tendon-foot
N22yC	Pyogenic infection of tendon sheath
N22yD	Tuberculous infection of tendon sheath
N22yE	Abscess of bursa-shoulder
N22yF	Abscess of bursa-elbow
N22yG	Abscess of bursa-wrist
N22yH	Abscess of bursa-hip
N22yJ	Abscess of bursa-knee
N22yK	Abscess of bursa-ankle
N22yL	Abscess of bursa-foot
N22yM	Short Achilles tendon - acquired
N22yN	Achilles degeneration
N22yz	Other synovial, tendon or bursa disorder NOS
N22z.	Synovial, tendon or bursa disorder NOS
N23..	Muscle, ligament and fascia disorders
N230.	Infective myositis
N2300	Infective myositis-neck
N2301	Infective myositis-back
N2302	Infective myositis-shoulder
N2303	Infective myositis-arm
N2304	Infective myositis-forearm
N2305	Infective myositis-hand
N2306	Infective myositis-pelvis
N2307	Infective myositis-thigh
N2308	Infective myositis-leg
N2309	Infective myositis-foot
N230A	Muscle abscess
N230B	Muscle abscess-neck
N230C	Muscle abscess-back
N230D	Muscle abscess-shoulder
N230E	Muscle abscess-arm
N230F	Muscle abscess-forearm
N230G	Muscle abscess-hand
N230H	Muscle abscess-pelvis
N230J	Muscle abscess-thigh
N230K	Muscle abscess-leg
N230L	Muscle abscess-foot
N231.	Muscle calcification and ossification
N2310	Muscular calcification and ossification, unspecified
N2311	Progressive myositis ossificans
N2312	Traumatic myositis ossificans
N2313	Post-operative heterotopic calcification
N2314	Polymyositis ossificans
N2315	Calcification and ossification of muscles associated with burns
N2316	Paralytic calcification and ossification of muscle
N231z	Muscle calcification or ossification NOS

For peer review only

N232.	Muscle wasting and disuse atrophy NEC
N2320	Amyotrophia NOS
N2321	Myofibrosis
N2322	Muscle wasting NEC
N232z	Muscle wasting/disuse atrophy NEC NOS
N233.	Other specific muscle disorder
N2330	Arthrogryposis
N2331	Immobility syndrome
N2332	Myositis in sarcoidosis
N2333	Rhabdomyolysis
N2334	Antisynthetase syndrome
N233z	Other specific muscle disorder NOS
N234.	Laxity of ligament
N235.	Hypermobility syndrome
N236.	Dupuytren's contracture
N2360	Dupuytren's disease of palm
N2361	Dupuytren's disease of palm, nodules with no contracture
N2362	Dupuytren's disease of palm, with contracture
N2363	Dupuytren's disease of finger(s)
N2364	Dupuytren's disease of finger(s), nodules with no contracture
N2365	Dupuytren's disease of finger(s), with contracture
N2366	Dupuytren's disease of palm and finger(s)
N2367	Dupuytren's disease of palm and finger(s), nodules with no contracture
N2368	Dupuytren's disease of palm and finger(s), with contracture
N237.	Other fibromatoses
N2370	Plantar fascial fibromatosis
N2371	Knuckle pads
N2372	Nodular fasciitis
N2373	Pseudosarcomatous fibromatosis
N237z	Other fibromatoses NOS
N238.	Muscle contracture
N2380	Contracture of pectoralis major
N2381	Contracture of triceps
N2382	Contracture of biceps
N2383	Contracture of wrist flexor(s)
N2384	Contracture of wrist extensor(s)
N2385	Contracture of flexor pollicis longus
N2386	Contracture of thumb extensor(s)
N2387	Contracture of flexor digitorum superficialis
N2388	Contracture of flexor digitorum profundus
N2389	Contracture of adductor pollicis
N238A	Contracture of other intrinsic muscle(s) of hand
N238B	Contracture of iliopsoas
N238C	Contracture of rectus femoris
N238D	Contracture of adductor muscle(s) of hip
N238E	Contracture of abductor muscle(s) of hip
N238F	Contracture of hamstring(s)
N238G	Contracture of quadriceps

For peer review only

N238H	Contracture of tendo achilles
N238J	Contracture of tibialis anterior
N238K	Contracture of tibialis posterior
N238L	Contracture of long toe flexor(s)
N238M	Contracture of long toe extensor(s)
N238N	Contracture of intrinsic muscle(s) of foot
N239.	Fibromyalgia
N23y.	Other muscle, ligament and fascia disorder
N23y0	Interstitial myositis
N23y1	Foreign body muscle granuloma
N23y2	Nontraumatic muscle rupture
N23y3	Diastasis recti abdominis
N23y4	Spasm of muscle
N23y5	Inappropriate firing of muscle
N23y6	Palmar space infection, thenar
N23y7	Palmar space infection, mid-palm
N23y8	Palmar space infection, hypo-thenar
N23y9	Calcific tendinitis
N23yA	Diastasis of muscle
N23yB	Ischaemic infarction of muscle
N23yC	Contracture of muscle
N23yD	Muscle strain
N23yE	Spasm of back muscles
N23yF	Muscle crush syndrome
N23yz	Other muscle, ligament or fascia disorder NOS
N23z.	Muscle, ligament or fascia disorder NOS
N24..	Other soft tissue disorders
N240.	Rheumatism and fibrositis unspecified
N2400	Rheumatism unspecified
N2401	Fibrositis unspecified
N2402	Muscular rheumatism
N2403	Rheumatic pain
N2405	Fibrositis of neck
N2406	Fibrositis arm
N2407	Hand rheumatism
N240z	Rheumatism or fibrositis NOS
N241.	Myalgia and myositis unspecified
N2410	Myalgia unspecified
N2411	Myositis unspecified
N2412	Fibromyositis NOS
N2413	Viral myalgia
N241z	Myalgia or myositis NOS
N242.	Neuralgia, neuritis and radiculitis unspecified
N2420	Neuralgia unspecified
N2421	Neuritis unspecified
N2422	Radiculitis unspecified
N2423	Neuropathic pain
N242z	Neuralgia, neuritis or radiculitis NOS

For peer review only

N243.	Panniculitis unspecified
N2430	Panniculitis of unspecified site
N2431	Hypertrophy of the knee fat pad
N2432	Weber - Christian disease
N243z	Panniculitis NOS
N244.	Fasciitis unspecified
N245.	Pain in limb
N2450	Hand pain
N2451	Foot pain
N2452	Pain in leg
N2453	Pain in arm
N2454	Calf pain
N2455	Axillary pain
N2456	Tender heel pad
N2457	Shoulder pain
N246.	Residual foreign body in soft tissue
N247.	Other musculoskeletal limb symptoms
N2470	Swelling of limb
N2471	Leg cramps
N2472	Cramp
N247z	Musculoskeletal limb symptoms NOS
N248.	Fibromyalgia
N2480	Myofascial pain syndrome
N2481	Piriformis syndrome
N24z.	Soft tissue disorders NOS
N25..	SAPHO syndrome (Synovitis Acne Pustulosis Hyperostosis Osteomyelitis)
N2y..	Other specified nonarticular rheumatism
N2z..	Nonarticular rheumatism NOS
N3...	Osteopathies, chondropathies and acquired musculoskeletal deformities
N30..	Osteomyelitis, periostitis and other infections affecting bone
N300.	Acute osteomyelitis
N3000	Acute osteomyelitis of unspecified site
N3001	Acute osteomyelitis of the shoulder region
N3002	Acute osteomyelitis of the upper arm
N3003	Acute osteomyelitis of the forearm
N3004	Acute osteomyelitis of the hand
N3005	Acute osteomyelitis of the pelvic region and thigh
N3006	Acute osteomyelitis of the lower leg
N3007	Acute osteomyelitis of the ankle and foot
N3008	Acute osteomyelitis of other specified site
N3009	Acute osteomyelitis of multiple sites
N300A	Acute osteomyelitis-cervical spine
N300B	Acute osteomyelitis-thoracic spine
N300C	Acute osteomyelitis-lumbar spine
N300D	Acute osteomyelitis-sacrum
N300E	Acute osteomyelitis-coccyx
N300F	Acute osteomyelitis-clavicle
N300G	Acute osteomyelitis-scapula

For peer review only

N300H	Acute osteomyelitis-humerus
N300J	Acute osteomyelitis-radius
N300K	Acute osteomyelitis-ulna
N300L	Acute osteomyelitis-carpal bone
N300M	Acute osteomyelitis-metacarpal
N300N	Acute osteomyelitis-phalanx of finger/thumb
N300P	Acute osteomyelitis-pelvis
N300Q	Acute osteomyelitis-femur
N300R	Acute osteomyelitis-patella
N300S	Acute osteomyelitis-tibia
N300T	Acute osteomyelitis-fibula
N300U	Acute osteomyelitis-calcaneum
N300V	Acute osteomyelitis-talus
N300W	Acute osteomyelitis-other tarsal bone
N300X	Acute osteomyelitis-metatarsal
N300Y	Acute osteomyelitis-phalanx of toe
N300Z	Acute haematogenous osteomyelitis
N300z	Acute osteomyelitis NOS
N301.	Chronic osteomyelitis
N3010	Chronic osteomyelitis of unspecified site
N3011	Chronic osteomyelitis of the shoulder region
N3012	Chronic osteomyelitis of the upper arm
N3013	Chronic osteomyelitis of the forearm
N3014	Chronic osteomyelitis of the hand
N3015	Chronic osteomyelitis of the pelvic region and thigh
N3016	Chronic osteomyelitis of the lower leg
N3017	Chronic osteomyelitis of the ankle and foot
N3018	Chronic osteomyelitis of other specified site
N3019	Chronic osteomyelitis of multiple sites
N301A	Chronic osteomyelitis-cervical spine
N301B	Chronic osteomyelitis-thoracic spine
N301C	Chronic osteomyelitis-lumbar spine
N301D	Chronic osteomyelitis-sacrum
N301E	Chronic osteomyelitis-coccyx
N301F	Brodie's abscess-cervical spine
N301G	Brodie's abscess-thoracic spine
N301H	Brodie's abscess-lumbar spine
N301J	Brodie's abscess-sacrum
N301K	Brodie's abscess-coccyx
N301L	Chronic multifocal osteomyelitis
N301M	Chronic osteomyelitis with draining sinus
N301z	Chronic osteomyelitis NOS
N302.	Unspecified osteomyelitis
N3020	Unspecified osteomyelitis of unspecified site
N3021	Unspecified osteomyelitis of the shoulder region
N3022	Unspecified osteomyelitis of the upper arm
N3023	Unspecified osteomyelitis of the forearm
N3024	Unspecified osteomyelitis of the hand

For peer review only

N3025	Unspecified osteomyelitis of the pelvic region and thigh
N3026	Unspecified osteomyelitis of the lower leg
N3027	Unspecified osteomyelitis of the ankle and foot
N3028	Unspecified osteomyelitis of other specified site
N3029	Unspecified osteomyelitis of multiple sites
N302A	Infection of cervical spine
N302B	Infection of thoracic spine
N302C	Infection of lumbar spine
N302D	Infection of sacrum
N302E	Infection of coccyx
N302F	Infection of clavicle
N302G	Infection of scapula
N302H	Infection of humerus
N302J	Infection of radius
N302K	Infection of ulna
N302L	Infection of carpal bone
N302M	Infection of metacarpal
N302N	Infection of phalanx of finger or thumb
N302P	Infection of pelvis
N302Q	Infection of femur
N302R	Infection of patella
N302S	Infection of tibia
N302T	Infection of fibula
N302U	Infection of calcaneum
N302V	Infection of talus
N302W	Infection of other tarsal bone
N302X	Infection of metatarsal
N302Y	Infection of phalanx of toe
N302Z	Infection of multiple bones
N302a	Osteomyelitis of vertebra
N302b	Brodie's abscess
N302z	Unspecified osteomyelitis NOS
N303.	Periostitis without osteomyelitis
N3030	Periostitis without mention of osteomyelitis, of unspecified site
N3031	Periostitis without mention of osteomyelitis, of the shoulder region
N3032	Periostitis without mention of osteomyelitis, of the upper arm
N3033	Periostitis without osteomyelitis, of the forearm
N3034	Periostitis without osteomyelitis, of the hand
N3035	Periostitis without osteomyelitis, of the pelvic region and thigh
N3036	Periostitis without mention of osteomyelitis, of the lower leg
N3037	Periostitis without mention of osteomyelitis, of the ankle and foot
N3038	Periostitis without mention of osteomyelitis, of other specified site
N3039	Periostitis without mention of osteomyelitis, of multiple sites
N303A	Periostitis without osteomyelitis-cervical spine
N303B	Periostitis without osteomyelitis-thoracic spine
N303C	Periostitis without osteomyelitis-lumbar spine
N303D	Periostitis without osteomyelitis-sacrum
N303E	Periostitis without osteomyelitis-coccyx

For peer review only

N303z	Periostitis without osteomyelitis NOS
N304.	Tuberculosis of spine (Pott's)
N3040	Tuberculosis of cervical spine
N3041	Tuberculosis of thoracic spine
N3042	Tuberculosis of lumbar spine
N3043	Tuberculosis of sacrum/coccyx
N305.	Tuberculosis of limb bones
N3050	Tuberculosis of unspecified limb bone
N3051	Tuberculosis of the upper arm bone
N3052	Tuberculosis of the forearm bone
N3053	Tuberculosis of the pelvic and thigh bones
N3054	Tuberculosis of the lower leg bone
N3055	Tuberculosis of other limb bones
N3056	Tuberculosis of multiple limb bones
N305z	Tuberculosis of limb bones NOS
N306.	Tuberculosis of other bones
N3060	Tuberculosis of bone, site unspecified
N3061	Tuberculosis of the bones of the shoulder region
N3062	Tuberculosis of the bones of the hand
N3063	Tuberculosis of the bones of the ankle and foot
N3064	Tuberculosis of the bones of other sites
N3065	Tuberculosis of the bones of multiple sites
N306z	Tuberculosis of bone NOS
N307.	Osteopathy from poliomyelitis
N3070	Poliomyelitis osteopathy of unspecified site
N3071	Poliomyelitis osteopathy of the shoulder region
N3072	Poliomyelitis osteopathy of the upper arm
N3073	Poliomyelitis osteopathy of the forearm
N3074	Poliomyelitis osteopathy of the hand
N3075	Poliomyelitis osteopathy of the pelvic region and thigh
N3076	Poliomyelitis osteopathy of the lower leg
N3077	Poliomyelitis osteopathy of the ankle and foot
N3078	Poliomyelitis osteopathy of other specified sites
N3079	Poliomyelitis osteopathy of multiple sites
N307z	Poliomyelitis osteopathy NOS
N308.	Subacute osteomyelitis
N3080	Subacute osteomyelitis-cervical spine
N3081	Subacute osteomyelitis-thoracic spine
N3082	Subacute osteomyelitis-lumbar spine
N3083	Subacute osteomyelitis-sacrum
N3084	Subacute osteomyelitis-coccyx
N309.	Subacute osteomyelitis
N30y.	Other infections involving bone
N30y0	Other infections involving bone, site unspecified
N30y1	Other infections involving bone, of the shoulder region
N30y2	Other infections involving bone, of the upper arm
N30y3	Other infections involving bone, of the forearm
N30y4	Other infections involving bone, of the hand

For peer review only

N30y5	Other infections involving bone, of the pelvic region and thigh
N30y6	Other infections involving bone, of the lower leg
N30y7	Other infections involving bone, of the ankle and foot
N30y8	Other infections involving bone, of other specified site
N30y9	Other infections involving bone, of multiple sites
N30yz	Other infections involving bone, NOS
N30z.	Bone infection NOS
N30z0	Bone infection NOS, of unspecified site
N30z1	Bone infection NOS, of the shoulder region
N30z2	Bone infection NOS, of the upper arm
N30z3	Bone infection NOS, of the forearm
N30z4	Bone infection NOS, of the hand
N30z5	Bone infection NOS, of the pelvic/thigh
N30z6	Bone infection NOS, of the lower leg
N30z7	Bone infection NOS, of ankle and foot
N30z8	Bone infection NOS, of other specified site
N30z9	Bone infection NOS, of multiple sites
N30zz	Bone infection NOS
N31..	Osteitis deformans and osteopathies associated with diseases EC
N310.	Osteitis deformans - Paget's disease of the bone
N3100	Paget's disease-cervical spine
N3101	Paget's disease-thoracic spine
N3102	Paget's disease-lumbar spine
N3103	Paget's disease-sacrum
N3104	Paget's disease-coccyx
N3105	Paget's disease-clavicle
N3106	Paget's disease-scapula
N3107	Paget's disease-humerus
N3108	Paget's disease-radius
N3109	Paget's disease-ulna
N310A	Paget's disease-carpal bone
N310B	Paget's disease-metacarpal
N310C	Paget's disease-phalanx of finger or thumb
N310D	Paget's disease-pelvis
N310E	Paget's disease-femur
N310F	Paget's disease-patella
N310G	Paget's disease-tibia
N310H	Paget's disease-fibula
N310J	Paget's disease-calcaneum
N310K	Paget's disease-talus
N310L	Paget's disease-other tarsal bone
N310M	Paget's disease-metatarsal
N310N	Paget's disease-phalanx of toe
N310P	Paget's disease-skull
N310x	Paget's disease-multiple sites
N310y	Paget's disease OS
N310z	Paget's disease NOS
N311.	Osteitis deformans associated with diseases EC

For peer review only

N3110	Osteitis deformans in neoplastic disease
N312.	Hypertrophic pulmonary osteoarthropathy
N31y.	Other bone involvement in diseases EC
N31z.	Bone involvement in diseases EC NOS
N32..	Osteochondropathies
N320.	Juvenile osteochondritis of the spine
N3200	Juvenile osteochondritis of the spine, unspecified
N3201	Scheuermann's disease
N3202	Calve's vertebral osteochondritis
N320z	Juvenile osteochondritis of the spine NOS
N321.	Juvenile osteochondritis of the hip and pelvis
N3210	Juvenile osteochondritis of the hip and pelvis, unspecified
N3211	Perthes' disease - osteochondritis of the femoral head
N3212	Ischiopubic synchondrosis
N3213	Juvenile osteochondritis of the acetabulum
N3214	Juvenile osteochondritis of the iliac crest
N3215	Juvenile osteochondritis of the symphysis pubis
N3216	Coxa plana
N3217	Pseudocoxalgia
N321z	Juvenile osteochondritis of the hip and pelvis NOS
N322.	Non traumatic slipped upper femoral epiphysis
N3220	Non traumatic acute slipped upper femoral epiphysis
N3221	Non traumatic acute-on-chronic slipped upper femoral epiphysis
N3222	Non traumatic chronic slipped upper femoral epiphysis
N323.	Juvenile osteochondritis of the arm and hand
N3230	Juvenile osteochondritis of the arm, unspecified
N3231	Juvenile osteochondritis of the hand, unspecified
N3232	Panner's disease - osteochondritis of capitulum of humerus
N3233	Kienbock's disease - osteochondritis of carpal lunate
N3234	Haas' disease - osteochondritis of head of humerus
N3235	Mauclaire's disease - osteochondritis of metacarpal heads
N3236	Burn's disease - osteochondritis of the lower ulna
N3237	Brailsford's disease - osteochondritis of the radial head
N323z	Juvenile osteochondritis of the arm and hand NOS
N324.	Juvenile osteochondrosis of the leg
N3240	Juvenile osteochondrosis of the leg, unspecified
N3241	Kohler's disease - osteochondrosis of primary patella centre
N3242	Blount's disease - osteochondrosis of proximal tibia
N3243	Juvenile osteochondrosis of the secondary patellar centre
N3244	Osgood-Schlatter's disease - osteochondrosis of tibial tubercle
N324z	Juvenile osteochondrosis of the leg, NOS
N325.	Juvenile osteochondrosis of the foot
N3250	Juvenile osteochondrosis of the foot, unspecified
N3251	Diaz's disease - osteochondrosis of astragalus
N3252	Sever's disease - osteochondrosis of calcaneum
N3253	Freiberg's disease - osteochondrosis of second metatarsal
N3254	Iselin's disease - osteochondrosis of fifth metatarsal
N3255	Haglund's disease - osteochondrosis of os tibiale externum

For peer review only

N3256	Kohler's disease - osteochondrosis of tarsal navicular
N325z	Juvenile osteochondrosis of the foot NOS
N326.	Other juvenile osteochondroses
N3260	Juvenile apophysitis NOS
N3261	Juvenile epiphysitis NOS
N3262	Juvenile osteochondritis NOS
N3263	Juvenile osteochondrosis NOS
N326z	Juvenile osteochondroses NOS
N327.	Osteochondritis dissecans
N3270	Osteochondritis dissecans of patella
N3271	Osteochondritis dissecans of lateral femoral condyle
N3272	Other osteochondritis dissecans of knee
N3273	Osteochondritis dissecans of the humeral head
N3274	Osteochondritis dissecans of the capitellum
N3275	Osteochondritis dissecans of the radial head
N3276	Other osteochondritis dissecans of the elbow
N3277	Osteochondritis dissecans of the wrist
N3278	Osteochondritis dissecans of the femoral head
N3279	Osteochondritis dissecans of the talus
N327y	Osteochondritis dissecans of other site
N328.	Juvenile osteochondrosis of spine
N32y.	Other specified forms of osteochondropathy
N32y0	Adult osteochondrosis of spine
N32y1	Kienbock's disease of adults
N32yz	Other specified osteochondropathy NOS
N32z.	Osteochondropathy NOS
N32z0	Apophysitis NOS
N32z1	Epiphysitis NOS
N32z2	Osteochondritis NOS
N32z3	Osteochondrosis NOS
N32zz	Osteochondropathy NOS
N33..	Other bone and cartilage disorders
N330.	Osteoporosis
N3300	Osteoporosis, unspecified
N3301	Senile osteoporosis
N3302	Postmenopausal osteoporosis
N3303	Idiopathic osteoporosis
N3304	Dissuse osteoporosis
N3305	Drug-induced osteoporosis
N3306	Postoophorectomy osteoporosis
N3307	Postsurgical malabsorption osteoporosis
N3308	Localized osteoporosis - Lequesne
N3309	Osteoporosis in multiple myelomatosis
N330A	Osteoporosis in endocrine disorders
N330B	Vertebral osteoporosis
N330C	Osteoporosis localized to spine
N330D	Osteoporosis due to corticosteroids
N330z	Osteoporosis NOS

For peer review only

N331.	Pathological fracture
N3310	Pathological fracture of thoracic vertebra
N3311	Pathological fracture of lumbar vertebra
N3312	Postoophorectomy osteoporosis with pathological fracture
N3313	Osteoporosis of disuse with pathological fracture
N3314	Postsurgical malabsorption osteoporosis with pathological fracture
N3315	Drug-induced osteoporosis with pathological fracture
N3316	Idiopathic osteoporosis with pathological fracture
N3317	Fracture of bone in neoplastic disease
N3318	Osteoporosis + pathological fracture lumbar vertebrae
N3319	Osteoporosis + pathological fracture thoracic vertebrae
N331A	Osteoporosis + pathological fracture cervical vertebrae
N331B	Postmenopausal osteoporosis with pathological fracture
N331C	Pathological fracture of cervical vertebra
N331D	Collapsed vertebra NOS
N331E	Collapse of cervical vertebra
N331F	Collapse of thoracic vertebra
N331G	Collapse of lumbar vertebra
N331H	Collapse of cervical vertebra due to osteoporosis
N331J	Collapse of lumbar vertebra due to osteoporosis
N331K	Collapse of thoracic vertebra due to osteoporosis
N331L	Collapse of vertebra due to osteoporosis NOS
N331M	Fragility fracture due to unspecified osteoporosis
N331N	Fragility fracture
N331y	Other specified pathological fracture
N331z	Pathological fracture NOS
N332.	Cyst of bone
N3320	Localised bone cyst, unspecified
N3321	Solitary bone cyst
N3322	Aneurysmal bone cyst
N3323	Monostotic fibrous dysplasia
N3324	Fibrous cortical defect
N3325	Brown tumour of hyperparathyroidism
N332z	Cyst of bone NOS
N333.	Hyperostosis of skull
N3330	Hyperostosis interna frontalis
N3331	Leontiasis ossium
N333z	Hyperostosis of skull NOS
N334.	Avascular necrosis of bone
N3340	Avascular necrosis of bone, site unspecified
N3341	Avascular necrosis of the head of humerus
N3342	Avascular necrosis of the head of femur
N3343	Avascular necrosis of the medial femoral condyle
N3344	Avascular necrosis of the talus
N3345	Avascular necrosis of capitellum
N3346	Avascular necrosis of lateral femoral condyle
N3347	Avascular necrosis of other bone
N3348	Idiopathic aseptic necrosis of bone

For peer review only

N3349	Osteonecrosis due to drugs
N334A	Osteonecrosis due to previous trauma
N334B	Osteonecrosis in caisson disease
N334C	Osteonecrosis due to haemoglobinopathy
N334z	Avascular bone necrosis NOS
N335.	Osteitis condensans
N3350	Osteitis condensans ilii
N336.	Tietze's disease
N337.	Algoneurodystrophy
N3370	Disuse atrophy of bone
N3371	Sudek's atrophy
N3372	Algodystrophy of hand
N3373	Algodystrophy of knee
N3374	Algodystrophy of foot
N337z	Algoneurodystrophy NOS
N338.	Malunion and nonunion of fracture
N3380	Malunion of fracture
N3381	Pseudoarthrosis - fracture nonunion
N3382	Hypertrophic non-union of fracture
N3383	Atrophic non-union of fracture
N3384	Angular mal-union of fracture
N3385	Rotational mal-union of fracture
N3386	Delayed union of fracture
N338z	Fracture malunion or nonunion NOS
N339.	Residual foreign body in bone
N33A.	Bone pain
N33A0	Bony pelvic pain
N33A1	Clavicle pain
N33B.	Osteoradionecrosis
N33C.	Complex regional pain syndrome type I
N33z.	Other and unspecified bone or cartilage disorders
N33z0	Bone and cartilage disorders, unspecified
N33z1	Epiphyseal arrest
N33z2	Chondromalacia NOS
N33z3	Diaphysitis
N33z4	Hypertrophy of bone
N33z5	Relapsing polychondritis
N33z6	Compensatory hypertrophy of bone
N33z7	Idiopathic hypertrophy of bone
N33z8	Complete epiphyseal arrest
N33z9	Partial epiphyseal arrest
N33zA	Skeletal fluorosis
N33zB	Chondrolysis
N33zC	Pseudarthrosis after fusion or arthrodesis
N33zD	Osteolysis
N33zE	Costochondritis
N33zF	Disorder of bone, unspecified
N33zG	Disorder of cartilage, unspecified

For peer review only

N33zH	Osteolytic lesion
N33zJ	Chondritis
N33zK	Hajdu-Cheney syndrome
N33zL	Osteitis of symphysis pubis
N33zM	Adynamic bone disease
N33zz	Bone or cartilage disorders NOS
N34..	Flat foot
N340.	Pes planus - acquired
N3400	Hypermobile flat foot
N3401	Rigid flat foot
N3402	Peroneal spastic flat foot
N341.	Talipes planus - acquired
N34z.	Flat foot NOS
N35..	Acquired deformities of toe
N350.	Hallux valgus - acquired
N351.	Hallux varus - acquired
N352.	Hallux rigidus - acquired
N353.	Acquired hammer deformity of great toe
N354.	Other hammer toe - acquired
N355.	Claw toe - acquired
N356.	Clawing of great toe
N357.	Crossover toe
N358.	Mallet toe
N359.	Bunionette
N35A.	Over-riding 5th toe
N35B.	Acquired varus deformity of toe
N35C.	Acquired hammer toe, other than great toe
N35y.	Other acquired toe deformity
N35z.	Acquired toe deformity NOS
N36..	Other acquired limb deformity
N360.	Acquired forearm deformity, excluding fingers
N3600	Acquired forearm deformity, unspecified
N3601	Cubitus valgus - acquired
N3602	Cubitus varus - acquired
N3603	Acquired valgus wrist deformity
N3604	Acquired varus wrist deformity
N3605	Wrist drop - acquired
N3606	Claw hand - acquired
N3607	Club hand - acquired
N360z	Acquired forearm deformity NOS
N361.	Mallet finger
N362.	Other acquired finger deformity
N3620	Acquired finger deformity, unspecified
N3621	Boutonniere finger deformity
N3622	Swan-neck finger deformity
N3623	Flexion deformity of finger
N3624	Extension deformity of finger
N3625	Deviation of finger

For peer review only

N3626	Rotational deformity of finger
N362z	Acquired finger deformity NOS
N363.	Acquired deformities of hip
N3630	Acquired hip deformity, unspecified
N3631	Coxa valga - acquired
N3632	Coxa vara - acquired
N3633	Acquired internal femoral torsion
N3634	Persistent femoral anteversion
N3635	Acquired external femoral torsion
N363z	Acquired hip deformity NOS
N364.	Acquired genu valgum and varum
N3640	Acquired genu valgum
N3641	Acquired genu varum
N364z	Acquired genu valgum or varum NOS
N365.	Genu recurvatum - acquired
N366.	Acquired knee deformity NOS
N3660	Flexion deformity of knee
N367.	Other acquired ankle and foot deformity
N3670	Acquired ankle or foot deformity, unspecified
N3671	Acquired equinovarus - clubfoot
N3672	Acquired equinus foot deformity
N3673	Aquired cavus foot deformity
N3674	Acquired claw foot
N3675	Acquired cavovarus foot deformity
N3676	Other acquired calcaneus deformity
N3677	Acquired talipes NEC
N3678	Acquired varus heel
N3679	Acquired valgus heel
N367A	Plantar flexion of the midtarsal joint
N367B	Plantar flexion contracture of tarsometatarsal joint
N367C	Flexion contracture of metatarsophalangeal joint
N367D	Extension contracture of metatarsophalangeal joint
N367E	Flexion contracture of toe joint
N367F	Acquired plantar-flexed forefoot
N367G	Acquired plantar-flexed first ray
N367H	Acquired plantar-flexed fifth ray
N367J	Acquired dorsiflexed forefoot
N367K	Acquired dorsiflexed first ray
N367L	Acquired supinated forefoot
N367M	Acquired pronated forefoot
N367N	Acquired forefoot adductus
N367P	Acquired forefoot abductus
N367Q	Serpentine foot
N367z	Acquired ankle or foot deformity NOS
N368.	Other knee deformity
N3680	Acquired internal tibial torsion
N3681	Acquired external tibial torsion
N3682	Chronic instability of knee

For peer review only

N369.	Flexion deformity
N36A.	Foot drop
N36y.	Acquired deformity of other limb parts
N36y0	Acquired unequal leg length
N36y1	Acquired unequal arm length
N36y2	Deformity of bone
N36y3	Deformity of clavicle
N36y4	Deformity of scapula
N36y5	Deformity of humerus
N36y6	Deformity of radius
N36y7	Deformity of ulna
N36y8	Deformity of carpal bone
N36y9	Deformity of metacarpal
N36yA	Deformity of phalanx of finger or thumb
N36yB	Deformity of pelvis
N36yC	Deformity of femur
N36yD	Deformity of patella
N36yE	Deformity of tibia
N36yF	Deformity of fibula
N36yG	Deformity of calcaneum
N36yH	Deformity of talus
N36yJ	Deformity of other tarsal bone
N36yK	Deformity of metatarsal
N36yL	Deformity of phalanx of toe
N36yM	Old amputee NOS
N36yN	Acquired radial deviation of hand
N36yz	Acquired limb deformity NEC
N36z.	Acquired limb deformity NOS
N37..	Curvature of spine
N370.	Adolescent postural kyphosis
N371.	Acquired kyphosis
N3710	Acquired postural kyphosis
N3711	Radiation kyphosis
N3712	Post-laminectomy kyphosis
N3713	Kyphosis secondary to other treatment
N371z	Acquired kyphosis NOS
N372.	Acquired lordosis
N3720	Acquired postural lordosis
N3721	Post-laminectomy lordosis
N3722	Other post-surgical lordosis
N372z	Acquired lordosis NOS
N373.	Kyphoscoliosis and scoliosis
N3730	Idiopathic scoliosis
N3731	Idiopathic kyphoscoliosis
N3732	Resolving infantile idiopathic scoliosis
N3733	Progressive infantile idiopathic scoliosis
N3734	Radiation scoliosis
N3735	Thoracogenic scoliosis

For peer review only

N3736	Postural scoliosis
N3737	Adolescent idiopathic scoliosis
N3738	Post-surgical scoliosis
N3739	Scoliosis secondary to other treatment
N373z	Kyphoscoliosis or scoliosis NOS
N374.	Curvature of spine associated with other conditions
N3740	Curvature of spine, unspecified
N3741	Kyphosis associated with other condition
N3742	Lordosis associated with other condition
N3743	Scoliosis associated with other condition
N3744	Kyphosis in skeletal dysplasia
N3745	Neuromuscular kyphosis
N3746	Osteoporotic kyphosis
N3747	Lordosis in skeletal dysplasia
N3748	Lordosis in hip disease
N3749	Neuromuscular lordosis
N374A	Scoliosis in skeletal dysplasia
N374B	Neuromuscular scoliosis
N374C	Scoliosis in neurofibromatosis
N374D	Scoliosis in connective tissue anomalies
N374E	Flatback syndrome
N374W	Lordosis, unspecified
N374X	Other and unspecified kyphosis
N374z	Curvature of spine associated with other conditions NOS
N37y.	Other curvatures of spine
N37z.	Curvature of spine NOS
N37z0	Acquired hunchback
N37zz	Curvature of spine NOS
N38..	Other acquired deformity
N380.	Acquired deformity of nose
N381.	Other acquired head deformity
N382.	Acquired deformity of neck
N383.	Acquired chest and rib deformity
N3830	Acquired chest deformity, unspecified
N3831	Acquired rib deformity, unspecified
N3832	Acquired pectus carinatum
N3833	Acquired pectus excavatum
N383z	Acquired chest or rib deformity NOS
N384.	Acquired spondylolisthesis
N3840	Dysplastic spondylolisthesis
N3841	Isthmic spondylolisthesis
N3842	Degenerative spondylolisthesis
N3843	Pedicular spondylolisthesis
N385.	Acquired deformity of spine NOS
N386.	Acquired deformity of pelvis
N387.	Cauliflower ear
N388.	Spondylolysis
N38y.	Other acquired deformity

For peer review only

N38y0	Acquired clavicle deformity
N38yz	Other acquired deformity NOS
N38z.	Acquired deformity NOS
N39..	Nonallopathic lesions, NEC
N390.	Nonallopathic lesion of the head region
N391.	Nonallopathic lesion of the cervical region
N392.	Nonallopathic lesion of the thoracic region
N393.	Nonallopathic lesion of the lumbar region
N394.	Nonallopathic lesion of the sacral region
N395.	Nonallopathic lesion of the pelvic region
N396.	Nonallopathic lesion of the legs
N397.	Nonallopathic lesion of the arms
N398.	Nonallopathic lesion of the rib cage
N399.	Nonallopathic lesion of the abdomen and other regions
N39z.	Nonallopathic lesion NEC NOS
N3y..	Other specified musculoskeletal disorders
N3y0.	Biomechanical lesions, not elsewhere classified
N3y00	Segmental and somatic dysfunction
N3y01	Subluxation complex (vertebral)
N3y02	Subluxation stenosis of neural canal
N3y03	Osseous stenosis of neural canal
N3y04	Connective tissue stenosis of neural canal
N3y05	Intervertebral disc stenosis of neural canal
N3y06	Osseous and subluxation stenosis of intervertebral foramina
N3y07	Connective tissue and disc stenosis of intervertebral foramina
N3z..	Other musculoskeletal disorder NOS
Ny...	Other specified diseases of musculoskeletal or connective tissue
Ny2..	Repetitive strain injury
Ny20.	Work related upper limb disorder
NyX..	Postprocedural musculoskeletal disorder, unspecified
Nyu..	[X]Additional musculoskeletal and connective tissue disease classification terms
Nyu0.	[X]Infectious arthropathies
Nyu00	[X]Other streptococcal arthritis and polyarthritis
Nyu01	[X]Arthritis and polyarthritis due to other specified bacterial agents
Nyu02	[X]Arthritis in other infectious and parasitic diseases classified elsewhere
Nyu03	[X]Other reactive arthropathies
Nyu04	[X]Other postinfectious arthropathies in diseases classified elsewhere
Nyu05	[X]Reactive arthropathy in other diseases classified elsewhere
Nyu1.	[X]Inflammatory polyarthropathies
Nyu10	[X]Rheumatoid arthritis with involvement of other organs or systems
Nyu11	[X]Other seropositive rheumatoid arthritis
Nyu12	[X]Other specified rheumatoid arthritis
Nyu13	[X]Other psoriatic arthropathies
Nyu14	[X]Other enteropathic arthropathies
Nyu15	[X]Other juvenile arthritis
Nyu16	[X]Juvenile arthritis in other diseases classified elsewhere
Nyu17	[X]Other secondary gout
Nyu18	[X]Other chondrocalcinosis

For peer review only

Nyu19	[X]Other specified crystal arthropathies
Nyu1A	[X]Other specific arthropathies, not elsewhere classified
Nyu1B	[X]Other specified arthritis
Nyu1C	[X]Gouty arthropathy due to enzyme defects and other inherited disorders classified elsewhere
Nyu1D	[X]Crystal arthropathy in other metabolic disorders classified elsewhere
Nyu1E	[X]Arthropathies in other endocrine, nutritional and metabolic disorders
Nyu1F	[X]Arthropathies in other specified diseases classified elsewhere
Nyu1G	[X]Seropositive rheumatoid arthritis, unspecified
Nyu2.	[X]Arthrosis
Nyu20	[X]Other polyarthrosis
Nyu21	[X]Other primary coxarthrosis
Nyu22	[X]Other dysplastic coxarthrosis
Nyu23	[X]Other post-traumatic coxarthrosis
Nyu24	[X]Other secondary coxarthrosis, bilateral
Nyu25	[X]Other primary gonarthrosis
Nyu26	[X]Other post-traumatic gonarthrosis
Nyu27	[X]Other secondary gonarthrosis, bilateral
Nyu28	[X]Other secondary gonarthrosis
Nyu29	[X]Other primary arthrosis of first carpometacarpal joint
Nyu2A	[X]Other post-traumatic arthrosis of first carpometacarpal joint
Nyu2B	[X]Other secondary arthrosis of first carpometacarpal joints, bilateral
Nyu2C	[X]Other secondary arthrosis of first carpometacarpal joint
Nyu2D	[X]Other specified arthrosis
Nyu2E	[X]Other secondary coxarthrosis
Nyu2F	[X]Post-traumatic arthrosis of other joints
Nyu3.	[X]Other joint disorders
Nyu30	[X]Other deformity of hallux (acquired)
Nyu31	[X]Other hammer toe(s) (acquired)
Nyu32	[X]Other deformities of toe(s) (acquired)
Nyu33	[X]Other acquired deformities of ankle and foot
Nyu34	[X]Other specified acquired deformities of limbs
Nyu35	[X]Other derangements of patella
Nyu36	[X]Other disorders of patella
Nyu37	[X]Other meniscus derangements
Nyu38	[X]Other spontaneous disruption of ligament(s) of knee
Nyu39	[X]Other internal derangements of knee
Nyu3A	[X]Other articular cartilage disorders
Nyu3B	[X]Other specific joint derangements, not elsewhere classified
Nyu3C	[X]Other instability of joint
Nyu3D	[X]Other specified joint disorders
Nyu3E	[X]Disorder of patella, unspecified
Nyu4.	[X]Systemic connective tissue disorders
Nyu40	[X]Other conditions related to polyarteritis nodosa
Nyu41	[X]Other giant cell arteritis
Nyu42	[X]Other specified necrotizing vasculopathies
Nyu43	[X]Other forms of systemic lupus erythematosus
Nyu44	[X]Other dermatomyositis

For peer review only

Nyu45	[X]Other forms of systemic sclerosis
Nyu46	[X]Other overlap syndromes
Nyu47	[X]Other systemic diseases of connective tissue
Nyu48	[X]Dermato(poly)myositis in neoplastic disease classified elsewhere
Nyu49	[X]Arthropathy in neoplastic disease classified elsewhere
Nyu4A	[X]Arthropathy in other blood disorders classified elsewhere
Nyu4B	[X]Arthropathy in hypersensitivity reactions classified elsewhere
Nyu4C	[X]Systemic disorders of connective tissue in other diseases classified elsewhere
Nyu4D	[X]Necrotising vasculopathy, unspecified
Nyu4E	[X]Dermatopolymyositis, unspecified
Nyu4F	[X]Mixed connective tissue disease
Nyu5.	[X]Deforming dorsopathies
Nyu50	[X]Other secondary kyphosis
Nyu51	[X]Other and unspecified kyphosis
Nyu52	[X]Other lordosis
Nyu53	[X]Other idiopathic scoliosis
Nyu54	[X]Other secondary scoliosis
Nyu55	[X]Other forms of scoliosis
Nyu56	[X]Other fusion of spine
Nyu57	[X]Other recurrent atlantoaxial subluxation
Nyu58	[X]Other recurrent vertebral subluxation
Nyu59	[X]Other specified deforming dorsopathies
Nyu5A	[X]Lordosis, unspecified
Nyu5B	[X]Spinal osteochondrosis, unspecified
Nyu6.	[X]Spondylopathies
Nyu60	[X]Other infective spondylopathies
Nyu61	[X]Other specified inflammatory spondylopathies
Nyu62	[X]Other spondylosis with myelopathy
Nyu63	[X]Other spondylosis with radiculopathy
Nyu64	[X]Other spondylosis
Nyu65	[X]Other specified spondylopathies
Nyu66	[X]Spondylopathy in other infectious and parasitic diseases classified elsewhere
Nyu67	[X]Collapsed vertebra in diseases classified elsewhere
Nyu68	[X]Spondylopathy in other diseases classified elsewhere
Nyu69	[X]Spondylopathy, unspecified
Nyu7.	[X]Other dorsopathies
Nyu70	[X]Other cervical disc displacement
Nyu71	[X]Other cervical disc degeneration
Nyu72	[X]Other cervical disc disorders
Nyu73	[X]Lumbar and other intervertebral disc disorders with myelopathy
Nyu74	[X]Lumbar and other intervertebral disc disorders with radiculopathy
Nyu75	[X]Other specified intervertebral disc displacement
Nyu76	[X]Other specified intervertebral disc degeneration
Nyu77	[X]Other specified intervertebral disc disorders
Nyu78	[X]Sacrococcygeal disorders, not elsewhere classified
Nyu79	[X]Other specified dorsopathies
Nyu7A	[X]Other dorsalgia
Nyu7B	[X]Cervical disc disorder, unspecified

For peer review only

Nyu8.	[X]Disorders of muscles
Nyu80	[X]Other myositis
Nyu81	[X]Other calcification of muscle
Nyu82	[X]Other ossification of muscle
Nyu83	[X]Other rupture of muscle (nontraumatic)
Nyu84	[X]Muscle wasting and atrophy, not elsewhere classified
Nyu85	[X]Other specified disorders of muscle
Nyu86	[X]Myositis in bacterial diseases classified elsewhere
Nyu87	[X]Myositis in protozoal and parasitic infections classified elsewhere
Nyu88	[X]Myositis in other infectious diseases classified elsewhere
Nyu89	[X]Myositis in sarcoidosis classified elsewhere
Nyu8A	[X]Other disorders of muscle in diseases classified elsewhere
Nyu8B	[X]Disorder of muscle, unspecified
Nyu9.	[X]Disorders of synovium and tendon
Nyu90	[X]Other infective (teno)synovitis
Nyu91	[X]Other synovitis and tenosynovitis
Nyu92	[X]Spontaneous rupture of other tendons
Nyu93	[X]Other contracture of tendon (sheath)
Nyu94	[X]Other specified disorders of synovium and tendon
Nyu95	[X]Synovitis and tenosynovitis in bacterial diseases classified elsewhere
Nyu96	[X]Other disorders of synovium and tendon in diseases classified elsewhere
Nyu97	[X]Synovial hypertrophy, not elsewhere classified
NyuA.	[X]Other soft tissue disorders
NyuA0	[X]Other bursitis of elbow
NyuA1	[X]Other bursitis of knee
NyuA2	[X]Other bursitis of hip
NyuA3	[X]Other soft tissue disorders related to use, overuse and pressure
NyuA4	[X]Other infective bursitis
NyuA5	[X]Other bursal cyst
NyuA6	[X]Other bursitis, not elsewhere classified
NyuA7	[X]Other specified bursopathies
NyuA8	[X]Fasciitis, not elsewhere classified
NyuA9	[X]Other fibroblastic disorders
NyuAA	[X]Other soft tissue disorders in other diseases classified elsewhere
NyuAB	[X]Other shoulder lesions
NyuAC	[X]Other enthesopathies of lower limb, excluding foot
NyuAD	[X]Other enthesopathy of foot
NyuAE	[X]Other enthesopathies, not elsewhere classified
NyuAF	[X]Other specified soft tissue disorders
NyuAG	[X]Unspecified soft tissue disorder related to use, overuse and pressure
NyuAH	[X]Fibroblastic disorder, unspecified
NyuAJ	[X]Enthesopathy of lower limb, unspecified
NyuB.	[X]Disorders of bone density and structure
NyuB0	[X]Other osteoporosis with pathological fracture
NyuB1	[X]Other osteoporosis
NyuB2	[X]Osteoporosis in other disorders classified elsewhere
NyuB3	[X]Other drug-induced osteomalacia in adults
NyuB4	[X]Other adult osteomalacia

For peer review only

NyuB5	[X]Other disorders of continuity of bone
NyuB6	[X]Other cyst of bone
NyuB7	[X]Other specified disorders of bone density and structure
NyuB8	[X]Unspecified osteoporosis with pathological fracture
NyuB9	[X]Adult osteomalacia, unspecified
NyuBA	[X]Disorder of bone density and structure, unspecified
NyuBB	[X]Erosion of bone
NyuBC	[X]Osteopenia
NyuC.	[X]Other osteopathies
NyuC0	[X]Other acute osteomyelitis
NyuC1	[X]Other chronic haematogenous osteomyelitis
NyuC2	[X]Other chronic osteomyelitis
NyuC3	[X]Other osteomyelitis
NyuC4	[X]Other secondary osteonecrosis
NyuC5	[X]Other osteonecrosis
NyuC6	[X]Other disorders of bone development and growth
NyuC7	[X]Other hypertrophic osteoarthropathy
NyuC8	[X]Other specified disorders of bone
NyuC9	[X]Periostitis in other infectious diseases classified elsewhere
NyuCA	[X]Osteopathy in other infectious diseases classified elsewhere
NyuCB	[X]Osteonecrosis due to haemoglobinopathy classified elsewhere
NyuCC	[X]Osteonecrosis in other diseases classified elsewhere
NyuCD	[X]Osteitis deformans in neoplastic diseases classified elsewhere
NyuCE	[X]Fracture of bone in neoplastic diseases classified elsewhere
NyuCF	[X]Osteopathy in other diseases classified elsewhere
NyuD.	[X]Chondropathies
NyuD0	[X]Other juvenile osteochondrosis of hip and pelvis
NyuD1	[X]Other juvenile osteochondrosis of upper limb
NyuD2	[X]Other specified juvenile osteochondrosis
NyuD3	[X]Other specified osteochondropathies
NyuD4	[X]Other specified disorders of cartilage
NyuDE	[X]Disorder of cartilage, unspecified
NyuE.	[X]Other disorders of the musculoskeletal system and connective tissue
NyuE0	[X]Other specified acquired deformities of musculoskeletal system
NyuE1	[X]Fracture of bone following insertion of orthopaedic implant, joint prosthesis, or bone plate
NyuE2	[X]Other postprocedural musculoskeletal disorders
NyuE3	[X]Other biomechanical lesions
NyuE4	[X]Postprocedural musculoskeletal disorder, unspecified
Nz...	Musculoskeletal and connective tissue diseases NOS

Hospital

ALT_CODE	DESCRIPTION
M00	Pyogenic arthritis
M000	Staphylococcal arthritis and polyarthritis
M0000	Staphylococcal arthritis and polyarthritis

For peer review only

M0001	Staphylococcal arthritis and polyarthritis
M0002	Staphylococcal arthritis and polyarthritis
M0003	Staphylococcal arthritis and polyarthritis
M0004	Staphylococcal arthritis and polyarthritis
M0005	Staphylococcal arthritis and polyarthritis
M0006	Staphylococcal arthritis and polyarthritis
M0007	Staphylococcal arthritis and polyarthritis
M0008	Staphylococcal arthritis and polyarthritis
M0009	Staphylococcal arthritis and polyarthritis
M001	Pneumococcal arthritis and polyarthritis
M0010	Pneumococcal arthritis and polyarthritis
M0011	Pneumococcal arthritis and polyarthritis
M0012	Pneumococcal arthritis and polyarthritis
M0013	Pneumococcal arthritis and polyarthritis
M0014	Pneumococcal arthritis and polyarthritis
M0015	Pneumococcal arthritis and polyarthritis
M0016	Pneumococcal arthritis and polyarthritis
M0017	Pneumococcal arthritis and polyarthritis
M0018	Pneumococcal arthritis and polyarthritis
M0019	Pneumococcal arthritis and polyarthritis
M002	Other streptococcal arthritis and polyarthritis
M0020	Other streptococcal arthritis and polyarthritis
M0021	Other streptococcal arthritis and polyarthritis
M0022	Other streptococcal arthritis and polyarthritis
M0023	Other streptococcal arthritis and polyarthritis
M0024	Other streptococcal arthritis and polyarthritis
M0025	Other streptococcal arthritis and polyarthritis
M0026	Other streptococcal arthritis and polyarthritis
M0027	Other streptococcal arthritis and polyarthritis
M0028	Other streptococcal arthritis and polyarthritis
M0029	Other streptococcal arthritis and polyarthritis
M008	Arthritis and polyarthritis due to other specified bacterial agents
M0080	Arthritis and polyarthritis due to other specified bacterial agents
M0081	Arthritis and polyarthritis due to other specified bacterial agents
M0082	Arthritis and polyarthritis due to other specified bacterial agents
M0083	Arthritis and polyarthritis due to other specified bacterial agents
M0084	Arthritis and polyarthritis due to other specified bacterial agents
M0085	Arthritis and polyarthritis due to other specified bacterial agents
M0086	Arthritis and polyarthritis due to other specified bacterial agents
M0087	Arthritis and polyarthritis due to other specified bacterial agents
M0088	Arthritis and polyarthritis due to other specified bacterial agents
M0089	Arthritis and polyarthritis due to other specified bacterial agents
M009	Pyogenic arthritis, unspecified
M0090	Pyogenic arthritis, unspecified
M0091	Pyogenic arthritis, unspecified
M0092	Pyogenic arthritis, unspecified
M0093	Pyogenic arthritis, unspecified
M0094	Pyogenic arthritis, unspecified

For peer review only

M0095	Pyogenic arthritis, unspecified
M0096	Pyogenic arthritis, unspecified
M0097	Pyogenic arthritis, unspecified
M0098	Pyogenic arthritis, unspecified
M0099	Pyogenic arthritis, unspecified
M01	Direct infections of joint in infectious and parasitic diseases classified elsewhere
M010	Meningococcal arthritis
M0100	Meningococcal arthritis
M0101	Meningococcal arthritis
M0102	Meningococcal arthritis
M0103	Meningococcal arthritis
M0104	Meningococcal arthritis
M0105	Meningococcal arthritis
M0106	Meningococcal arthritis
M0107	Meningococcal arthritis
M0108	Meningococcal arthritis
M0109	Meningococcal arthritis
M011	Tuberculous arthritis
M0110	Tuberculous arthritis
M0111	Tuberculous arthritis
M0112	Tuberculous arthritis
M0113	Tuberculous arthritis
M0114	Tuberculous arthritis
M0115	Tuberculous arthritis
M0116	Tuberculous arthritis
M0117	Tuberculous arthritis
M0118	Tuberculous arthritis
M0119	Tuberculous arthritis
M012	Arthritis in Lyme disease
M0120	Arthritis in Lyme disease
M0121	Arthritis in Lyme disease
M0122	Arthritis in Lyme disease
M0123	Arthritis in Lyme disease
M0124	Arthritis in Lyme disease
M0125	Arthritis in Lyme disease
M0126	Arthritis in Lyme disease
M0127	Arthritis in Lyme disease
M0128	Arthritis in Lyme disease
M0129	Arthritis in Lyme disease
M013	Arthritis in other bacterial diseases classified elsewhere
M0130	Arthritis in other bacterial diseases classified elsewhere
M0131	Arthritis in other bacterial diseases classified elsewhere
M0132	Arthritis in other bacterial diseases classified elsewhere
M0133	Arthritis in other bacterial diseases classified elsewhere
M0134	Arthritis in other bacterial diseases classified elsewhere
M0135	Arthritis in other bacterial diseases classified elsewhere
M0136	Arthritis in other bacterial diseases classified elsewhere
M0137	Arthritis in other bacterial diseases classified elsewhere

For peer review only

M0138	Arthritis in other bacterial diseases classified elsewhere
M0139	Arthritis in other bacterial diseases classified elsewhere
M014	Rubella arthritis
M0140	Rubella arthritis
M0141	Rubella arthritis
M0142	Rubella arthritis
M0143	Rubella arthritis
M0144	Rubella arthritis
M0145	Rubella arthritis
M0146	Rubella arthritis
M0147	Rubella arthritis
M0148	Rubella arthritis
M0149	Rubella arthritis
M015	Arthritis in other viral diseases classified elsewhere
M0150	Arthritis in other viral diseases classified elsewhere
M0151	Arthritis in other viral diseases classified elsewhere
M0152	Arthritis in other viral diseases classified elsewhere
M0153	Arthritis in other viral diseases classified elsewhere
M0154	Arthritis in other viral diseases classified elsewhere
M0155	Arthritis in other viral diseases classified elsewhere
M0156	Arthritis in other viral diseases classified elsewhere
M0157	Arthritis in other viral diseases classified elsewhere
M0158	Arthritis in other viral diseases classified elsewhere
M0159	Arthritis in other viral diseases classified elsewhere
M016	Arthritis in mycoses
M0160	Arthritis in mycoses
M0161	Arthritis in mycoses
M0162	Arthritis in mycoses
M0163	Arthritis in mycoses
M0164	Arthritis in mycoses
M0165	Arthritis in mycoses
M0166	Arthritis in mycoses
M0167	Arthritis in mycoses
M0168	Arthritis in mycoses
M0169	Arthritis in mycoses
M018	Arthritis in other infectious and parasitic diseases classified elsewhere
M0180	Arthritis in other infectious and parasitic diseases classified elsewhere
M0181	Arthritis in other infectious and parasitic diseases classified elsewhere
M0182	Arthritis in other infectious and parasitic diseases classified elsewhere
M0183	Arthritis in other infectious and parasitic diseases classified elsewhere
M0184	Arthritis in other infectious and parasitic diseases classified elsewhere
M0185	Arthritis in other infectious and parasitic diseases classified elsewhere
M0186	Arthritis in other infectious and parasitic diseases classified elsewhere
M0187	Arthritis in other infectious and parasitic diseases classified elsewhere
M0188	Arthritis in other infectious and parasitic diseases classified elsewhere
M0189	Arthritis in other infectious and parasitic diseases classified elsewhere
M02	Reactive arthropathies
M020	Arthropathy following intestinal bypass

For peer review only

M0200	Arthropathy following intestinal bypass
M0201	Arthropathy following intestinal bypass
M0202	Arthropathy following intestinal bypass
M0203	Arthropathy following intestinal bypass
M0204	Arthropathy following intestinal bypass
M0205	Arthropathy following intestinal bypass
M0206	Arthropathy following intestinal bypass
M0207	Arthropathy following intestinal bypass
M0208	Arthropathy following intestinal bypass
M0209	Arthropathy following intestinal bypass
M021	Postdysenteric arthropathy
M0210	Postdysenteric arthropathy
M0211	Postdysenteric arthropathy
M0212	Postdysenteric arthropathy
M0213	Postdysenteric arthropathy
M0214	Postdysenteric arthropathy
M0215	Postdysenteric arthropathy
M0216	Postdysenteric arthropathy
M0217	Postdysenteric arthropathy
M0218	Postdysenteric arthropathy
M0219	Postdysenteric arthropathy
M022	Postimmunization arthropathy
M0220	Postimmunization arthropathy
M0221	Postimmunization arthropathy
M0222	Postimmunization arthropathy
M0223	Postimmunization arthropathy
M0224	Postimmunization arthropathy
M0225	Postimmunization arthropathy
M0226	Postimmunization arthropathy
M0227	Postimmunization arthropathy
M0228	Postimmunization arthropathy
M0229	Postimmunization arthropathy
M023	Reiter disease
M023	Reiter's disease
M0230	Reiter disease
M0230	Reiter's disease
M0231	Reiter disease
M0231	Reiter's disease
M0232	Reiter disease
M0232	Reiter's disease
M0233	Reiter disease
M0233	Reiter's disease
M0234	Reiter disease
M0234	Reiter's disease
M0235	Reiter disease
M0235	Reiter's disease
M0236	Reiter disease
M0236	Reiter's disease

For peer review only

M0237	Reiter disease
M0237	Reiter's disease
M0238	Reiter disease
M0238	Reiter's disease
M0239	Reiter disease
M0239	Reiter's disease
M028	Other reactive arthropathies
M0280	Other reactive arthropathies
M0281	Other reactive arthropathies
M0282	Other reactive arthropathies
M0283	Other reactive arthropathies
M0284	Other reactive arthropathies
M0285	Other reactive arthropathies
M0286	Other reactive arthropathies
M0287	Other reactive arthropathies
M0288	Other reactive arthropathies
M0289	Other reactive arthropathies
M029	Reactive arthropathy, unspecified
M0290	Reactive arthropathy, unspecified
M0291	Reactive arthropathy, unspecified
M0292	Reactive arthropathy, unspecified
M0293	Reactive arthropathy, unspecified
M0294	Reactive arthropathy, unspecified
M0295	Reactive arthropathy, unspecified
M0296	Reactive arthropathy, unspecified
M0297	Reactive arthropathy, unspecified
M0298	Reactive arthropathy, unspecified
M0299	Reactive arthropathy, unspecified
M03	Postinfective and reactive arthropathies in diseases classified elsewhere
M030	Postmeningococcal arthritis
M0300	Postmeningococcal arthritis
M0301	Postmeningococcal arthritis
M0302	Postmeningococcal arthritis
M0303	Postmeningococcal arthritis
M0304	Postmeningococcal arthritis
M0305	Postmeningococcal arthritis
M0306	Postmeningococcal arthritis
M0307	Postmeningococcal arthritis
M0308	Postmeningococcal arthritis
M0309	Postmeningococcal arthritis
M031	Postinfective arthropathy in syphilis
M0310	Postinfective arthropathy in syphilis
M0311	Postinfective arthropathy in syphilis
M0312	Postinfective arthropathy in syphilis
M0313	Postinfective arthropathy in syphilis
M0314	Postinfective arthropathy in syphilis
M0315	Postinfective arthropathy in syphilis
M0316	Postinfective arthropathy in syphilis

For peer review only

M0317	Postinfective arthropathy in syphilis
M0318	Postinfective arthropathy in syphilis
M0319	Postinfective arthropathy in syphilis
M032	Other postinfectious arthropathies in diseases classified elsewhere
M0320	Other postinfectious arthropathies in diseases classified elsewhere
M0321	Other postinfectious arthropathies in diseases classified elsewhere
M0322	Other postinfectious arthropathies in diseases classified elsewhere
M0323	Other postinfectious arthropathies in diseases classified elsewhere
M0324	Other postinfectious arthropathies in diseases classified elsewhere
M0325	Other postinfectious arthropathies in diseases classified elsewhere
M0326	Other postinfectious arthropathies in diseases classified elsewhere
M0327	Other postinfectious arthropathies in diseases classified elsewhere
M0328	Other postinfectious arthropathies in diseases classified elsewhere
M0329	Other postinfectious arthropathies in diseases classified elsewhere
M036	Reactive arthropathy in other diseases classified elsewhere
M0360	Reactive arthropathy in other diseases classified elsewhere
M0361	Reactive arthropathy in other diseases classified elsewhere
M0362	Reactive arthropathy in other diseases classified elsewhere
M0363	Reactive arthropathy in other diseases classified elsewhere
M0364	Reactive arthropathy in other diseases classified elsewhere
M0365	Reactive arthropathy in other diseases classified elsewhere
M0366	Reactive arthropathy in other diseases classified elsewhere
M0367	Reactive arthropathy in other diseases classified elsewhere
M0368	Reactive arthropathy in other diseases classified elsewhere
M0369	Reactive arthropathy in other diseases classified elsewhere
M05	Seropositive rheumatoid arthritis
M050	Felty syndrome
M050	Felty's syndrome
M0500	Felty syndrome
M0500	Felty's syndrome
M0501	Felty syndrome
M0501	Felty's syndrome
M0502	Felty syndrome
M0502	Felty's syndrome
M0503	Felty syndrome
M0503	Felty's syndrome
M0504	Felty syndrome
M0504	Felty's syndrome
M0505	Felty syndrome
M0505	Felty's syndrome
M0506	Felty syndrome
M0506	Felty's syndrome
M0507	Felty syndrome
M0507	Felty's syndrome
M0508	Felty syndrome
M0508	Felty's syndrome
M0509	Felty syndrome
M0509	Felty's syndrome

For peer review only

M051	Rheumatoid lung disease
M0510	Rheumatoid lung disease
M0511	Rheumatoid lung disease
M0512	Rheumatoid lung disease
M0513	Rheumatoid lung disease
M0514	Rheumatoid lung disease
M0515	Rheumatoid lung disease
M0516	Rheumatoid lung disease
M0517	Rheumatoid lung disease
M0518	Rheumatoid lung disease
M0519	Rheumatoid lung disease
M052	Rheumatoid vasculitis
M0520	Rheumatoid vasculitis
M0521	Rheumatoid vasculitis
M0522	Rheumatoid vasculitis
M0523	Rheumatoid vasculitis
M0524	Rheumatoid vasculitis
M0525	Rheumatoid vasculitis
M0526	Rheumatoid vasculitis
M0527	Rheumatoid vasculitis
M0528	Rheumatoid vasculitis
M0529	Rheumatoid vasculitis
M053	Rheumatoid arthritis with involvement of other organs and systems
M0530	Rheumatoid arthritis with involvement of other organs and systems
M0531	Rheumatoid arthritis with involvement of other organs and systems
M0532	Rheumatoid arthritis with involvement of other organs and systems
M0533	Rheumatoid arthritis with involvement of other organs and systems
M0534	Rheumatoid arthritis with involvement of other organs and systems
M0535	Rheumatoid arthritis with involvement of other organs and systems
M0536	Rheumatoid arthritis with involvement of other organs and systems
M0537	Rheumatoid arthritis with involvement of other organs and systems
M0538	Rheumatoid arthritis with involvement of other organs and systems
M0539	Rheumatoid arthritis with involvement of other organs and systems
M058	Other seropositive rheumatoid arthritis
M0580	Other seropositive rheumatoid arthritis
M0581	Other seropositive rheumatoid arthritis
M0582	Other seropositive rheumatoid arthritis
M0583	Other seropositive rheumatoid arthritis
M0584	Other seropositive rheumatoid arthritis
M0585	Other seropositive rheumatoid arthritis
M0586	Other seropositive rheumatoid arthritis
M0587	Other seropositive rheumatoid arthritis
M0588	Other seropositive rheumatoid arthritis
M0589	Other seropositive rheumatoid arthritis
M059	Seropositive rheumatoid arthritis, unspecified
M0590	Seropositive rheumatoid arthritis, unspecified
M0591	Seropositive rheumatoid arthritis, unspecified
M0592	Seropositive rheumatoid arthritis, unspecified

For peer review only

M0593	Seropositive rheumatoid arthritis, unspecified
M0594	Seropositive rheumatoid arthritis, unspecified
M0595	Seropositive rheumatoid arthritis, unspecified
M0596	Seropositive rheumatoid arthritis, unspecified
M0597	Seropositive rheumatoid arthritis, unspecified
M0598	Seropositive rheumatoid arthritis, unspecified
M0599	Seropositive rheumatoid arthritis, unspecified
M06	Other rheumatoid arthritis
M060	Seronegative rheumatoid arthritis
M0600	Seronegative rheumatoid arthritis
M0601	Seronegative rheumatoid arthritis
M0602	Seronegative rheumatoid arthritis
M0603	Seronegative rheumatoid arthritis
M0604	Seronegative rheumatoid arthritis
M0605	Seronegative rheumatoid arthritis
M0606	Seronegative rheumatoid arthritis
M0607	Seronegative rheumatoid arthritis
M0608	Seronegative rheumatoid arthritis
M0609	Seronegative rheumatoid arthritis
M061	Adult-onset Still disease
M061	Adult-onset Still's disease
M0610	Adult-onset Still disease
M0610	Adult-onset Still's disease
M0611	Adult-onset Still disease
M0611	Adult-onset Still's disease
M0612	Adult-onset Still disease
M0612	Adult-onset Still's disease
M0613	Adult-onset Still disease
M0613	Adult-onset Still's disease
M0614	Adult-onset Still disease
M0614	Adult-onset Still's disease
M0615	Adult-onset Still disease
M0615	Adult-onset Still's disease
M0616	Adult-onset Still disease
M0616	Adult-onset Still's disease
M0617	Adult-onset Still disease
M0617	Adult-onset Still's disease
M0618	Adult-onset Still disease
M0618	Adult-onset Still's disease
M0619	Adult-onset Still disease
M0619	Adult-onset Still's disease
M062	Rheumatoid bursitis
M0620	Rheumatoid bursitis
M0621	Rheumatoid bursitis
M0622	Rheumatoid bursitis
M0623	Rheumatoid bursitis
M0624	Rheumatoid bursitis
M0625	Rheumatoid bursitis

For peer review only

M0626	Rheumatoid bursitis
M0627	Rheumatoid bursitis
M0628	Rheumatoid bursitis
M0629	Rheumatoid bursitis
M063	Rheumatoid nodule
M0630	Rheumatoid nodule
M0631	Rheumatoid nodule
M0632	Rheumatoid nodule
M0633	Rheumatoid nodule
M0634	Rheumatoid nodule
M0635	Rheumatoid nodule
M0636	Rheumatoid nodule
M0637	Rheumatoid nodule
M0638	Rheumatoid nodule
M0639	Rheumatoid nodule
M064	Inflammatory polyarthropathy
M0640	Inflammatory polyarthropathy
M0641	Inflammatory polyarthropathy
M0642	Inflammatory polyarthropathy
M0643	Inflammatory polyarthropathy
M0644	Inflammatory polyarthropathy
M0645	Inflammatory polyarthropathy
M0646	Inflammatory polyarthropathy
M0647	Inflammatory polyarthropathy
M0648	Inflammatory polyarthropathy
M0649	Inflammatory polyarthropathy
M068	Other specified rheumatoid arthritis
M0680	Other specified rheumatoid arthritis
M0681	Other specified rheumatoid arthritis
M0682	Other specified rheumatoid arthritis
M0683	Other specified rheumatoid arthritis
M0684	Other specified rheumatoid arthritis
M0685	Other specified rheumatoid arthritis
M0686	Other specified rheumatoid arthritis
M0687	Other specified rheumatoid arthritis
M0688	Other specified rheumatoid arthritis
M0689	Other specified rheumatoid arthritis
M069	Rheumatoid arthritis, unspecified
M0690	Rheumatoid arthritis, unspecified
M0691	Rheumatoid arthritis, unspecified
M0692	Rheumatoid arthritis, unspecified
M0693	Rheumatoid arthritis, unspecified
M0694	Rheumatoid arthritis, unspecified
M0695	Rheumatoid arthritis, unspecified
M0696	Rheumatoid arthritis, unspecified
M0697	Rheumatoid arthritis, unspecified
M0698	Rheumatoid arthritis, unspecified
M0699	Rheumatoid arthritis, unspecified

For peer review only

M07	Psoriatic and enteropathic arthropathies
M070	Distal interphalangeal psoriatic arthropathy
M0700	Distal interphalangeal psoriatic arthropathy
M0704	Distal interphalangeal psoriatic arthropathy
M0707	Distal interphalangeal psoriatic arthropathy
M0709	Distal interphalangeal psoriatic arthropathy
M071	Arthritis mutilans
M0710	Arthritis mutilans
M0711	Arthritis mutilans
M0712	Arthritis mutilans
M0713	Arthritis mutilans
M0714	Arthritis mutilans
M0715	Arthritis mutilans
M0716	Arthritis mutilans
M0717	Arthritis mutilans
M0718	Arthritis mutilans
M0719	Arthritis mutilans
M072	Psoriatic spondylitis
M0720	Psoriatic spondylitis
M0721	Psoriatic spondylitis
M0722	Psoriatic spondylitis
M0723	Psoriatic spondylitis
M0724	Psoriatic spondylitis
M0725	Psoriatic spondylitis
M0726	Psoriatic spondylitis
M0727	Psoriatic spondylitis
M0728	Psoriatic spondylitis
M0729	Psoriatic spondylitis
M073	Other psoriatic arthropathies
M0730	Other psoriatic arthropathies
M0731	Other psoriatic arthropathies
M0732	Other psoriatic arthropathies
M0733	Other psoriatic arthropathies
M0734	Other psoriatic arthropathies
M0735	Other psoriatic arthropathies
M0736	Other psoriatic arthropathies
M0737	Other psoriatic arthropathies
M0738	Other psoriatic arthropathies
M0739	Other psoriatic arthropathies
M074	Arthropathy in Crohn disease [regional enteritis]
M074	Arthropathy in Crohn's disease [regional enteritis]
M0740	Arthropathy in Crohn disease [regional enteritis]
M0740	Arthropathy in Crohn's disease [regional enteritis]
M0741	Arthropathy in Crohn disease [regional enteritis]
M0741	Arthropathy in Crohn's disease [regional enteritis]
M0742	Arthropathy in Crohn disease [regional enteritis]
M0742	Arthropathy in Crohn's disease [regional enteritis]
M0743	Arthropathy in Crohn disease [regional enteritis]

For peer review only

M0743	Arthropathy in Crohn's disease [regional enteritis]
M0744	Arthropathy in Crohn disease [regional enteritis]
M0744	Arthropathy in Crohn's disease [regional enteritis]
M0745	Arthropathy in Crohn disease [regional enteritis]
M0745	Arthropathy in Crohn's disease [regional enteritis]
M0746	Arthropathy in Crohn disease [regional enteritis]
M0746	Arthropathy in Crohn's disease [regional enteritis]
M0747	Arthropathy in Crohn disease [regional enteritis]
M0747	Arthropathy in Crohn's disease [regional enteritis]
M0748	Arthropathy in Crohn disease [regional enteritis]
M0748	Arthropathy in Crohn's disease [regional enteritis]
M0749	Arthropathy in Crohn disease [regional enteritis]
M0749	Arthropathy in Crohn's disease [regional enteritis]
M075	Arthropathy in ulcerative colitis
M0750	Arthropathy in ulcerative colitis
M0751	Arthropathy in ulcerative colitis
M0752	Arthropathy in ulcerative colitis
M0753	Arthropathy in ulcerative colitis
M0754	Arthropathy in ulcerative colitis
M0755	Arthropathy in ulcerative colitis
M0756	Arthropathy in ulcerative colitis
M0757	Arthropathy in ulcerative colitis
M0758	Arthropathy in ulcerative colitis
M0759	Arthropathy in ulcerative colitis
M076	Other enteropathic arthropathies
M0760	Other enteropathic arthropathies
M0761	Other enteropathic arthropathies
M0762	Other enteropathic arthropathies
M0763	Other enteropathic arthropathies
M0764	Other enteropathic arthropathies
M0765	Other enteropathic arthropathies
M0766	Other enteropathic arthropathies
M0767	Other enteropathic arthropathies
M0768	Other enteropathic arthropathies
M0769	Other enteropathic arthropathies
M08	Juvenile arthritis
M080	Juvenile rheumatoid arthritis
M0800	Juvenile rheumatoid arthritis
M0801	Juvenile rheumatoid arthritis
M0802	Juvenile rheumatoid arthritis
M0803	Juvenile rheumatoid arthritis
M0804	Juvenile rheumatoid arthritis
M0805	Juvenile rheumatoid arthritis
M0806	Juvenile rheumatoid arthritis
M0807	Juvenile rheumatoid arthritis
M0808	Juvenile rheumatoid arthritis
M0809	Juvenile rheumatoid arthritis
M081	Juvenile ankylosing spondylitis

For peer review only

M0810	Juvenile ankylosing spondylitis
M0811	Juvenile ankylosing spondylitis
M0812	Juvenile ankylosing spondylitis
M0813	Juvenile ankylosing spondylitis
M0814	Juvenile ankylosing spondylitis
M0815	Juvenile ankylosing spondylitis
M0816	Juvenile ankylosing spondylitis
M0817	Juvenile ankylosing spondylitis
M0818	Juvenile ankylosing spondylitis
M0819	Juvenile ankylosing spondylitis
M082	Juvenile arthritis with systemic onset
M0820	Juvenile arthritis with systemic onset
M0821	Juvenile arthritis with systemic onset
M0822	Juvenile arthritis with systemic onset
M0823	Juvenile arthritis with systemic onset
M0824	Juvenile arthritis with systemic onset
M0825	Juvenile arthritis with systemic onset
M0826	Juvenile arthritis with systemic onset
M0827	Juvenile arthritis with systemic onset
M0828	Juvenile arthritis with systemic onset
M0829	Juvenile arthritis with systemic onset
M083	Juvenile polyarthritis (seronegative)
M0830	Juvenile polyarthritis (seronegative)
M0831	Juvenile polyarthritis (seronegative)
M0832	Juvenile polyarthritis (seronegative)
M0833	Juvenile polyarthritis (seronegative)
M0834	Juvenile polyarthritis (seronegative)
M0835	Juvenile polyarthritis (seronegative)
M0836	Juvenile polyarthritis (seronegative)
M0837	Juvenile polyarthritis (seronegative)
M0838	Juvenile polyarthritis (seronegative)
M0839	Juvenile polyarthritis (seronegative)
M084	Pauciarticular juvenile arthritis
M0840	Pauciarticular juvenile arthritis
M0841	Pauciarticular juvenile arthritis
M0842	Pauciarticular juvenile arthritis
M0843	Pauciarticular juvenile arthritis
M0844	Pauciarticular juvenile arthritis
M0845	Pauciarticular juvenile arthritis
M0846	Pauciarticular juvenile arthritis
M0847	Pauciarticular juvenile arthritis
M0848	Pauciarticular juvenile arthritis
M0849	Pauciarticular juvenile arthritis
M088	Other juvenile arthritis
M0880	Other juvenile arthritis
M0881	Other juvenile arthritis
M0882	Other juvenile arthritis
M0883	Other juvenile arthritis

For peer review only

M0884	Other juvenile arthritis
M0885	Other juvenile arthritis
M0886	Other juvenile arthritis
M0887	Other juvenile arthritis
M0888	Other juvenile arthritis
M0889	Other juvenile arthritis
M089	Juvenile arthritis, unspecified
M0890	Juvenile arthritis, unspecified
M0891	Juvenile arthritis, unspecified
M0892	Juvenile arthritis, unspecified
M0893	Juvenile arthritis, unspecified
M0894	Juvenile arthritis, unspecified
M0895	Juvenile arthritis, unspecified
M0896	Juvenile arthritis, unspecified
M0897	Juvenile arthritis, unspecified
M0898	Juvenile arthritis, unspecified
M0899	Juvenile arthritis, unspecified
M09	Juvenile arthritis in diseases classified elsewhere
M090	Juvenile arthritis in psoriasis
M0900	Juvenile arthritis in psoriasis
M0901	Juvenile arthritis in psoriasis
M0902	Juvenile arthritis in psoriasis
M0903	Juvenile arthritis in psoriasis
M0904	Juvenile arthritis in psoriasis
M0905	Juvenile arthritis in psoriasis
M0906	Juvenile arthritis in psoriasis
M0907	Juvenile arthritis in psoriasis
M0908	Juvenile arthritis in psoriasis
M0909	Juvenile arthritis in psoriasis
M091	Juvenile arthritis in Crohn disease [regional enteritis]
M091	Juvenile arthritis in Crohn's disease [regional enteritis]
M0910	Juvenile arthritis in Crohn disease [regional enteritis]
M0910	Juvenile arthritis in Crohn's disease [regional enteritis]
M0911	Juvenile arthritis in Crohn disease [regional enteritis]
M0911	Juvenile arthritis in Crohn's disease [regional enteritis]
M0912	Juvenile arthritis in Crohn disease [regional enteritis]
M0912	Juvenile arthritis in Crohn's disease [regional enteritis]
M0913	Juvenile arthritis in Crohn disease [regional enteritis]
M0913	Juvenile arthritis in Crohn's disease [regional enteritis]
M0914	Juvenile arthritis in Crohn disease [regional enteritis]
M0914	Juvenile arthritis in Crohn's disease [regional enteritis]
M0915	Juvenile arthritis in Crohn disease [regional enteritis]
M0915	Juvenile arthritis in Crohn's disease [regional enteritis]
M0916	Juvenile arthritis in Crohn disease [regional enteritis]
M0916	Juvenile arthritis in Crohn's disease [regional enteritis]
M0917	Juvenile arthritis in Crohn disease [regional enteritis]
M0917	Juvenile arthritis in Crohn's disease [regional enteritis]
M0918	Juvenile arthritis in Crohn disease [regional enteritis]

For peer review only

M0918	Juvenile arthritis in Crohn's disease [regional enteritis]
M0919	Juvenile arthritis in Crohn disease [regional enteritis]
M0919	Juvenile arthritis in Crohn's disease [regional enteritis]
M092	Juvenile arthritis in ulcerative colitis
M0920	Juvenile arthritis in ulcerative colitis
M0921	Juvenile arthritis in ulcerative colitis
M0922	Juvenile arthritis in ulcerative colitis
M0923	Juvenile arthritis in ulcerative colitis
M0924	Juvenile arthritis in ulcerative colitis
M0925	Juvenile arthritis in ulcerative colitis
M0926	Juvenile arthritis in ulcerative colitis
M0927	Juvenile arthritis in ulcerative colitis
M0928	Juvenile arthritis in ulcerative colitis
M0929	Juvenile arthritis in ulcerative colitis
M098	Juvenile arthritis in other diseases classified elsewhere
M0980	Juvenile arthritis in other diseases classified elsewhere
M0981	Juvenile arthritis in other diseases classified elsewhere
M0982	Juvenile arthritis in other diseases classified elsewhere
M0983	Juvenile arthritis in other diseases classified elsewhere
M0984	Juvenile arthritis in other diseases classified elsewhere
M0985	Juvenile arthritis in other diseases classified elsewhere
M0986	Juvenile arthritis in other diseases classified elsewhere
M0987	Juvenile arthritis in other diseases classified elsewhere
M0988	Juvenile arthritis in other diseases classified elsewhere
M0989	Juvenile arthritis in other diseases classified elsewhere
M10	Gout
M100	Idiopathic gout
M1000	Idiopathic gout
M1001	Idiopathic gout
M1002	Idiopathic gout
M1003	Idiopathic gout
M1004	Idiopathic gout
M1005	Idiopathic gout
M1006	Idiopathic gout
M1007	Idiopathic gout
M1008	Idiopathic gout
M1009	Idiopathic gout
M101	Lead-induced gout
M1010	Lead-induced gout
M1011	Lead-induced gout
M1012	Lead-induced gout
M1013	Lead-induced gout
M1014	Lead-induced gout
M1015	Lead-induced gout
M1016	Lead-induced gout
M1017	Lead-induced gout
M1018	Lead-induced gout
M1019	Lead-induced gout

For peer review only

M102	Drug-induced gout
M1020	Drug-induced gout
M1021	Drug-induced gout
M1022	Drug-induced gout
M1023	Drug-induced gout
M1024	Drug-induced gout
M1025	Drug-induced gout
M1026	Drug-induced gout
M1027	Drug-induced gout
M1028	Drug-induced gout
M1029	Drug-induced gout
M103	Gout due to impairment of renal function
M1030	Gout due to impairment of renal function
M1031	Gout due to impairment of renal function
M1032	Gout due to impairment of renal function
M1033	Gout due to impairment of renal function
M1034	Gout due to impairment of renal function
M1035	Gout due to impairment of renal function
M1036	Gout due to impairment of renal function
M1037	Gout due to impairment of renal function
M1038	Gout due to impairment of renal function
M1039	Gout due to impairment of renal function
M104	Other secondary gout
M1040	Other secondary gout
M1041	Other secondary gout
M1042	Other secondary gout
M1043	Other secondary gout
M1044	Other secondary gout
M1045	Other secondary gout
M1046	Other secondary gout
M1047	Other secondary gout
M1048	Other secondary gout
M1049	Other secondary gout
M109	Gout, unspecified
M1090	Gout, unspecified
M1091	Gout, unspecified
M1092	Gout, unspecified
M1093	Gout, unspecified
M1094	Gout, unspecified
M1095	Gout, unspecified
M1096	Gout, unspecified
M1097	Gout, unspecified
M1098	Gout, unspecified
M1099	Gout, unspecified
M11	Other crystal arthropathies
M110	Hydroxyapatite deposition disease
M1100	Hydroxyapatite deposition disease
M1101	Hydroxyapatite deposition disease

For peer review only

M1102	Hydroxyapatite deposition disease
M1103	Hydroxyapatite deposition disease
M1104	Hydroxyapatite deposition disease
M1105	Hydroxyapatite deposition disease
M1106	Hydroxyapatite deposition disease
M1107	Hydroxyapatite deposition disease
M1108	Hydroxyapatite deposition disease
M1109	Hydroxyapatite deposition disease
M111	Familial chondrocalcinosis
M1110	Familial chondrocalcinosis
M1111	Familial chondrocalcinosis
M1112	Familial chondrocalcinosis
M1113	Familial chondrocalcinosis
M1114	Familial chondrocalcinosis
M1115	Familial chondrocalcinosis
M1116	Familial chondrocalcinosis
M1117	Familial chondrocalcinosis
M1118	Familial chondrocalcinosis
M1119	Familial chondrocalcinosis
M112	Other chondrocalcinosis
M1120	Other chondrocalcinosis
M1121	Other chondrocalcinosis
M1122	Other chondrocalcinosis
M1123	Other chondrocalcinosis
M1124	Other chondrocalcinosis
M1125	Other chondrocalcinosis
M1126	Other chondrocalcinosis
M1127	Other chondrocalcinosis
M1128	Other chondrocalcinosis
M1129	Other chondrocalcinosis
M118	Other specified crystal arthropathies
M1180	Other specified crystal arthropathies
M1181	Other specified crystal arthropathies
M1182	Other specified crystal arthropathies
M1183	Other specified crystal arthropathies
M1184	Other specified crystal arthropathies
M1185	Other specified crystal arthropathies
M1186	Other specified crystal arthropathies
M1187	Other specified crystal arthropathies
M1188	Other specified crystal arthropathies
M1189	Other specified crystal arthropathies
M119	Crystal arthropathy, unspecified
M1190	Crystal arthropathy, unspecified
M1191	Crystal arthropathy, unspecified
M1192	Crystal arthropathy, unspecified
M1193	Crystal arthropathy, unspecified
M1194	Crystal arthropathy, unspecified
M1195	Crystal arthropathy, unspecified

For peer review only

M1196	Crystal arthropathy, unspecified
M1197	Crystal arthropathy, unspecified
M1198	Crystal arthropathy, unspecified
M1199	Crystal arthropathy, unspecified
M12	Other specific arthropathies
M120	Chronic postrheumatic arthropathy [Jaccoud]
M1200	Chronic postrheumatic arthropathy [Jaccoud]
M1201	Chronic postrheumatic arthropathy [Jaccoud]
M1202	Chronic postrheumatic arthropathy [Jaccoud]
M1203	Chronic postrheumatic arthropathy [Jaccoud]
M1204	Chronic postrheumatic arthropathy [Jaccoud]
M1205	Chronic postrheumatic arthropathy [Jaccoud]
M1206	Chronic postrheumatic arthropathy [Jaccoud]
M1207	Chronic postrheumatic arthropathy [Jaccoud]
M1208	Chronic postrheumatic arthropathy [Jaccoud]
M1209	Chronic postrheumatic arthropathy [Jaccoud]
M121	Kaschin-Beck disease
M1210	Kaschin-Beck disease
M1211	Kaschin-Beck disease
M1212	Kaschin-Beck disease
M1213	Kaschin-Beck disease
M1214	Kaschin-Beck disease
M1215	Kaschin-Beck disease
M1216	Kaschin-Beck disease
M1217	Kaschin-Beck disease
M1218	Kaschin-Beck disease
M1219	Kaschin-Beck disease
M122	Villonodular synovitis (pigmented)
M1220	Villonodular synovitis (pigmented)
M1221	Villonodular synovitis (pigmented)
M1222	Villonodular synovitis (pigmented)
M1223	Villonodular synovitis (pigmented)
M1224	Villonodular synovitis (pigmented)
M1225	Villonodular synovitis (pigmented)
M1226	Villonodular synovitis (pigmented)
M1227	Villonodular synovitis (pigmented)
M1228	Villonodular synovitis (pigmented)
M1229	Villonodular synovitis (pigmented)
M123	Palindromic rheumatism
M1230	Palindromic rheumatism
M1231	Palindromic rheumatism
M1232	Palindromic rheumatism
M1233	Palindromic rheumatism
M1234	Palindromic rheumatism
M1235	Palindromic rheumatism
M1236	Palindromic rheumatism
M1237	Palindromic rheumatism
M1238	Palindromic rheumatism

For peer review only

M1239	Palindromic rheumatism
M124	Intermittent hydrarthrosis
M1240	Intermittent hydrarthrosis
M1241	Intermittent hydrarthrosis
M1242	Intermittent hydrarthrosis
M1243	Intermittent hydrarthrosis
M1244	Intermittent hydrarthrosis
M1245	Intermittent hydrarthrosis
M1246	Intermittent hydrarthrosis
M1247	Intermittent hydrarthrosis
M1248	Intermittent hydrarthrosis
M1249	Intermittent hydrarthrosis
M125	Traumatic arthropathy
M1250	Traumatic arthropathy
M1251	Traumatic arthropathy
M1252	Traumatic arthropathy
M1253	Traumatic arthropathy
M1254	Traumatic arthropathy
M1255	Traumatic arthropathy
M1256	Traumatic arthropathy
M1257	Traumatic arthropathy
M1258	Traumatic arthropathy
M1259	Traumatic arthropathy
M128	Other specific arthropathies, not elsewhere classified
M1280	Other specific arthropathies, not elsewhere classified
M1281	Other specific arthropathies, not elsewhere classified
M1282	Other specific arthropathies, not elsewhere classified
M1283	Other specific arthropathies, not elsewhere classified
M1284	Other specific arthropathies, not elsewhere classified
M1285	Other specific arthropathies, not elsewhere classified
M1286	Other specific arthropathies, not elsewhere classified
M1287	Other specific arthropathies, not elsewhere classified
M1288	Other specific arthropathies, not elsewhere classified
M1289	Other specific arthropathies, not elsewhere classified
M13	Other arthritis
M130	Polyarthritis, unspecified
M1300	Polyarthritis, unspecified
M1301	Polyarthritis, unspecified
M1302	Polyarthritis, unspecified
M1303	Polyarthritis, unspecified
M1304	Polyarthritis, unspecified
M1305	Polyarthritis, unspecified
M1306	Polyarthritis, unspecified
M1307	Polyarthritis, unspecified
M1308	Polyarthritis, unspecified
M1309	Polyarthritis, unspecified
M131	Monoarthritis, not elsewhere classified
M1310	Monoarthritis, not elsewhere classified

For peer review only

M1311	Monoarthritis, not elsewhere classified
M1312	Monoarthritis, not elsewhere classified
M1313	Monoarthritis, not elsewhere classified
M1314	Monoarthritis, not elsewhere classified
M1315	Monoarthritis, not elsewhere classified
M1316	Monoarthritis, not elsewhere classified
M1317	Monoarthritis, not elsewhere classified
M1318	Monoarthritis, not elsewhere classified
M1319	Monoarthritis, not elsewhere classified
M138	Other specified arthritis
M1380	Other specified arthritis
M1381	Other specified arthritis
M1382	Other specified arthritis
M1383	Other specified arthritis
M1384	Other specified arthritis
M1385	Other specified arthritis
M1386	Other specified arthritis
M1387	Other specified arthritis
M1388	Other specified arthritis
M1389	Other specified arthritis
M139	Arthritis, unspecified
M1390	Arthritis, unspecified
M1391	Arthritis, unspecified
M1392	Arthritis, unspecified
M1393	Arthritis, unspecified
M1394	Arthritis, unspecified
M1395	Arthritis, unspecified
M1396	Arthritis, unspecified
M1397	Arthritis, unspecified
M1398	Arthritis, unspecified
M1399	Arthritis, unspecified
M14	Arthropathies in other diseases classified elsewhere
M140	Gouty arthropathy due to enzyme defects and other inherited disorders
M141	Crystal arthropathy in other metabolic disorders
M142	Diabetic arthropathy
M143	Lipoid dermatoarthritis
M144	Arthropathy in amyloidosis
M145	Arthropathies in other endocrine, nutritional and metabolic disorders
M146	Neuropathic arthropathy
M148	Arthropathies in other specified diseases classified elsewhere
M15	Polyarthrosis
M150	Primary generalized (osteo)arthrosis
M151	Heberden nodes (with arthropathy)
M151	Heberden's nodes (with arthropathy)
M152	Bouchard nodes (with arthropathy)
M152	Bouchard's nodes (with arthropathy)
M153	Secondary multiple arthrosis
M154	Erosive (osteo)arthrosis

For peer review only

M158	Other polyarthrosis
M159	Polyarthrosis, unspecified
M16	Coxarthrosis [arthrosis of hip]
M160	Primary coxarthrosis, bilateral
M161	Other primary coxarthrosis
M162	Coxarthrosis resulting from dysplasia, bilateral
M163	Other dysplastic coxarthrosis
M164	Post-traumatic coxarthrosis, bilateral
M165	Other post-traumatic coxarthrosis
M166	Other secondary coxarthrosis, bilateral
M167	Other secondary coxarthrosis
M169	Coxarthrosis, unspecified
M17	Gonarthrosis [arthrosis of knee]
M170	Primary gonarthrosis, bilateral
M171	Other primary gonarthrosis
M172	Post-traumatic gonarthrosis, bilateral
M173	Other post-traumatic gonarthrosis
M174	Other secondary gonarthrosis, bilateral
M175	Other secondary gonarthrosis
M179	Gonarthrosis, unspecified
M18	Arthrosis of first carpometacarpal joint
M180	Primary arthrosis of first carpometacarpal joints, bilateral
M181	Other primary arthrosis of first carpometacarpal joint
M182	Post-traumatic arthrosis of first carpometacarpal joints, bilateral
M183	Other post-traumatic arthrosis of first carpometacarpal joint
M184	Other secondary arthrosis of first carpometacarpal joints, bilateral
M185	Other secondary arthrosis of first carpometacarpal joint
M189	Arthrosis of first carpometacarpal joint, unspecified
M19	Other arthrosis
M190	Primary arthrosis of other joints
M1900	Primary arthrosis of other joints
M1901	Primary arthrosis of other joints
M1902	Primary arthrosis of other joints
M1903	Primary arthrosis of other joints
M1904	Primary arthrosis of other joints
M1905	Primary arthrosis of other joints
M1907	Primary arthrosis of other joints
M1908	Primary arthrosis of other joints
M1909	Primary arthrosis of other joints
M191	Post-traumatic arthrosis of other joints
M1910	Post-traumatic arthrosis of other joints
M1911	Post-traumatic arthrosis of other joints
M1912	Post-traumatic arthrosis of other joints
M1913	Post-traumatic arthrosis of other joints
M1914	Post-traumatic arthrosis of other joints
M1915	Post-traumatic arthrosis of other joints
M1917	Post-traumatic arthrosis of other joints
M1918	Post-traumatic arthrosis of other joints

For peer review only

M1919	Post-traumatic arthrosis of other joints
M192	Other secondary arthrosis
M1920	Other secondary arthrosis
M1921	Other secondary arthrosis
M1922	Other secondary arthrosis
M1923	Other secondary arthrosis
M1924	Other secondary arthrosis
M1925	Other secondary arthrosis
M1927	Other secondary arthrosis
M1928	Other secondary arthrosis
M1929	Other secondary arthrosis
M198	Other specified arthrosis
M1980	Other specified arthrosis
M1981	Other specified arthrosis
M1982	Other specified arthrosis
M1983	Other specified arthrosis
M1984	Other specified arthrosis
M1985	Other specified arthrosis
M1986	Other specified arthrosis
M1987	Other specified arthrosis
M1988	Other specified arthrosis
M1989	Other specified arthrosis
M199	Arthrosis, unspecified
M1990	Arthrosis, unspecified
M1991	Arthrosis, unspecified
M1992	Arthrosis, unspecified
M1993	Arthrosis, unspecified
M1994	Arthrosis, unspecified
M1995	Arthrosis, unspecified
M1997	Arthrosis, unspecified
M1998	Arthrosis, unspecified
M1999	Arthrosis, unspecified
M20	Acquired deformities of fingers and toes
M200	Deformity of finger(s)
M201	Hallux valgus (acquired)
M202	Hallux rigidus
M203	Other deformity of hallux (acquired)
M204	Other hammer toe(s) (acquired)
M205	Other deformities of toe(s) (acquired)
M206	Acquired deformity of toe(s), unspecified
M21	Other acquired deformities of limbs
M210	Valgus deformity, not elsewhere classified
M2100	Valgus deformity, not elsewhere classified
M2101	Valgus deformity, not elsewhere classified
M2102	Valgus deformity, not elsewhere classified
M2103	Valgus deformity, not elsewhere classified
M2104	Valgus deformity, not elsewhere classified
M2105	Valgus deformity, not elsewhere classified

For peer review only

M2106	Valgus deformity, not elsewhere classified
M2107	Valgus deformity, not elsewhere classified
M2108	Valgus deformity, not elsewhere classified
M2109	Valgus deformity, not elsewhere classified
M211	Varus deformity, not elsewhere classified
M2110	Varus deformity, not elsewhere classified
M2111	Varus deformity, not elsewhere classified
M2112	Varus deformity, not elsewhere classified
M2113	Varus deformity, not elsewhere classified
M2114	Varus deformity, not elsewhere classified
M2115	Varus deformity, not elsewhere classified
M2116	Varus deformity, not elsewhere classified
M2117	Varus deformity, not elsewhere classified
M2118	Varus deformity, not elsewhere classified
M2119	Varus deformity, not elsewhere classified
M212	Flexion deformity
M2120	Flexion deformity
M2121	Flexion deformity
M2122	Flexion deformity
M2123	Flexion deformity
M2124	Flexion deformity
M2125	Flexion deformity
M2126	Flexion deformity
M2127	Flexion deformity
M2128	Flexion deformity
M2129	Flexion deformity
M213	Wrist or foot drop (acquired)
M2130	Wrist or foot drop (acquired)
M2133	Wrist or foot drop (acquired)
M2134	Wrist or foot drop (acquired)
M2137	Wrist or foot drop (acquired)
M214	Flat foot [pes planus] (acquired)
M215	Acquired clawhand, clubhand, clawfoot and clubfoot
M2150	Acquired clawhand, clubhand, clawfoot and clubfoot
M2154	Acquired clawhand, clubhand, clawfoot and clubfoot
M2157	Acquired clawhand, clubhand, clawfoot and clubfoot
M216	Other acquired deformities of ankle and foot
M217	Unequal limb length (acquired)
M2170	Unequal limb length (acquired)
M2172	Unequal limb length (acquired)
M2173	Unequal limb length (acquired)
M2174	Unequal limb length (acquired)
M2175	Unequal limb length (acquired)
M2176	Unequal limb length (acquired)
M2177	Unequal limb length (acquired)
M2179	Unequal limb length (acquired)
M218	Other specified acquired deformities of limbs
M2180	Other specified acquired deformities of limbs

For peer review only

M2181	Other specified acquired deformities of limbs
M2182	Other specified acquired deformities of limbs
M2183	Other specified acquired deformities of limbs
M2184	Other specified acquired deformities of limbs
M2185	Other specified acquired deformities of limbs
M2186	Other specified acquired deformities of limbs
M2187	Other specified acquired deformities of limbs
M2189	Other specified acquired deformities of limbs
M219	Acquired deformity of limb, unspecified
M2190	Acquired deformity of limb, unspecified
M2191	Acquired deformity of limb, unspecified
M2192	Acquired deformity of limb, unspecified
M2193	Acquired deformity of limb, unspecified
M2194	Acquired deformity of limb, unspecified
M2195	Acquired deformity of limb, unspecified
M2196	Acquired deformity of limb, unspecified
M2197	Acquired deformity of limb, unspecified
M2199	Acquired deformity of limb, unspecified
M22	Disorders of patella
M220	Recurrent dislocation of patella
M221	Recurrent subluxation of patella
M222	Patellofemoral disorders
M223	Other derangements of patella
M224	Chondromalacia patellae
M228	Other disorders of patella
M229	Disorder of patella, unspecified
M23	Internal derangement of knee
M230	Cystic meniscus
M2300	Cystic meniscus
M2301	Cystic meniscus
M2302	Cystic meniscus
M2303	Cystic meniscus
M2304	Cystic meniscus
M2305	Cystic meniscus
M2306	Cystic meniscus
M2309	Cystic meniscus
M231	Discoid meniscus (congenital)
M2310	Discoid meniscus (congenital)
M2311	Discoid meniscus (congenital)
M2312	Discoid meniscus (congenital)
M2313	Discoid meniscus (congenital)
M2314	Discoid meniscus (congenital)
M2315	Discoid meniscus (congenital)
M2316	Discoid meniscus (congenital)
M2319	Discoid meniscus (congenital)
M232	Derangement of meniscus due to old tear or injury
M2320	Derangement of meniscus due to old tear or injury
M2321	Derangement of meniscus due to old tear or injury

For peer review only

M2322	Derangement of meniscus due to old tear or injury
M2323	Derangement of meniscus due to old tear or injury
M2324	Derangement of meniscus due to old tear or injury
M2325	Derangement of meniscus due to old tear or injury
M2326	Derangement of meniscus due to old tear or injury
M2329	Derangement of meniscus due to old tear or injury
M233	Other meniscus derangements
M2330	Other meniscus derangements
M2331	Other meniscus derangements
M2332	Other meniscus derangements
M2333	Other meniscus derangements
M2334	Other meniscus derangements
M2335	Other meniscus derangements
M2336	Other meniscus derangements
M2339	Other meniscus derangements
M234	Loose body in knee
M235	Chronic instability of knee
M2350	Chronic instability of knee
M2351	Chronic instability of knee
M2352	Chronic instability of knee
M2353	Chronic instability of knee
M2354	Chronic instability of knee
M2355	Chronic instability of knee
M2356	Chronic instability of knee
M2357	Chronic instability of knee
M2359	Chronic instability of knee
M236	Other spontaneous disruption of ligament(s) of knee
M2360	Other spontaneous disruption of ligament(s) of knee
M2361	Other spontaneous disruption of ligament(s) of knee
M2362	Other spontaneous disruption of ligament(s) of knee
M2363	Other spontaneous disruption of ligament(s) of knee
M2364	Other spontaneous disruption of ligament(s) of knee
M2367	Other spontaneous disruption of ligament(s) of knee
M2369	Other spontaneous disruption of ligament(s) of knee
M238	Other internal derangements of knee
M2380	Other internal derangements of knee
M2381	Other internal derangements of knee
M2382	Other internal derangements of knee
M2383	Other internal derangements of knee
M2384	Other internal derangements of knee
M2385	Other internal derangements of knee
M2386	Other internal derangements of knee
M2387	Other internal derangements of knee
M2389	Other internal derangements of knee
M239	Internal derangement of knee, unspecified
M2390	Internal derangement of knee, unspecified
M2391	Internal derangement of knee, unspecified
M2392	Internal derangement of knee, unspecified

For peer review only

M2393	Internal derangement of knee, unspecified
M2394	Internal derangement of knee, unspecified
M2395	Internal derangement of knee, unspecified
M2396	Internal derangement of knee, unspecified
M2397	Internal derangement of knee, unspecified
M2399	Internal derangement of knee, unspecified
M24	Other specific joint derangements
M240	Loose body in joint
M2400	Loose body in joint
M2401	Loose body in joint
M2402	Loose body in joint
M2403	Loose body in joint
M2404	Loose body in joint
M2405	Loose body in joint
M2407	Loose body in joint
M2408	Loose body in joint
M2409	Loose body in joint
M241	Other articular cartilage disorders
M2410	Other articular cartilage disorders
M2411	Other articular cartilage disorders
M2412	Other articular cartilage disorders
M2413	Other articular cartilage disorders
M2414	Other articular cartilage disorders
M2415	Other articular cartilage disorders
M2416	Other articular cartilage disorders
M2417	Other articular cartilage disorders
M2418	Other articular cartilage disorders
M2419	Other articular cartilage disorders
M242	Disorder of ligament
M2420	Disorder of ligament
M2421	Disorder of ligament
M2422	Disorder of ligament
M2423	Disorder of ligament
M2424	Disorder of ligament
M2425	Disorder of ligament
M2426	Disorder of ligament
M2427	Disorder of ligament
M2428	Disorder of ligament
M2429	Disorder of ligament
M243	Pathological dislocation and subluxation of joint, not elsewhere classified
M2430	Pathological dislocation and subluxation of joint, not elsewhere classified
M2431	Pathological dislocation and subluxation of joint, not elsewhere classified
M2432	Pathological dislocation and subluxation of joint, not elsewhere classified
M2433	Pathological dislocation and subluxation of joint, not elsewhere classified
M2434	Pathological dislocation and subluxation of joint, not elsewhere classified
M2435	Pathological dislocation and subluxation of joint, not elsewhere classified
M2436	Pathological dislocation and subluxation of joint, not elsewhere classified
M2437	Pathological dislocation and subluxation of joint, not elsewhere classified

For peer review only

M2438	Pathological dislocation and subluxation of joint, not elsewhere classified
M2439	Pathological dislocation and subluxation of joint, not elsewhere classified
M244	Recurrent dislocation and subluxation of joint
M2440	Recurrent dislocation and subluxation of joint
M2441	Recurrent dislocation and subluxation of joint
M2442	Recurrent dislocation and subluxation of joint
M2443	Recurrent dislocation and subluxation of joint
M2444	Recurrent dislocation and subluxation of joint
M2445	Recurrent dislocation and subluxation of joint
M2446	Recurrent dislocation and subluxation of joint
M2447	Recurrent dislocation and subluxation of joint
M2448	Recurrent dislocation and subluxation of joint
M2449	Recurrent dislocation and subluxation of joint
M245	Contracture of joint
M2450	Contracture of joint
M2451	Contracture of joint
M2452	Contracture of joint
M2453	Contracture of joint
M2454	Contracture of joint
M2455	Contracture of joint
M2456	Contracture of joint
M2457	Contracture of joint
M2458	Contracture of joint
M2459	Contracture of joint
M246	Ankylosis of joint
M2460	Ankylosis of joint
M2461	Ankylosis of joint
M2462	Ankylosis of joint
M2463	Ankylosis of joint
M2464	Ankylosis of joint
M2465	Ankylosis of joint
M2466	Ankylosis of joint
M2467	Ankylosis of joint
M2468	Ankylosis of joint
M2469	Ankylosis of joint
M247	Protrusio acetabuli
M248	Other specific joint derangements, not elsewhere classified
M2480	Other specific joint derangements, not elsewhere classified
M2481	Other specific joint derangements, not elsewhere classified
M2482	Other specific joint derangements, not elsewhere classified
M2483	Other specific joint derangements, not elsewhere classified
M2484	Other specific joint derangements, not elsewhere classified
M2485	Other specific joint derangements, not elsewhere classified
M2486	Other specific joint derangements, not elsewhere classified
M2487	Other specific joint derangements, not elsewhere classified
M2488	Other specific joint derangements, not elsewhere classified
M2489	Other specific joint derangements, not elsewhere classified
M249	Joint derangement, unspecified

For peer review only

M2490	Joint derangement, unspecified
M2491	Joint derangement, unspecified
M2492	Joint derangement, unspecified
M2493	Joint derangement, unspecified
M2494	Joint derangement, unspecified
M2495	Joint derangement, unspecified
M2496	Joint derangement, unspecified
M2497	Joint derangement, unspecified
M2498	Joint derangement, unspecified
M2499	Joint derangement, unspecified
M25	Other joint disorders, not elsewhere classified
M250	Haemarthrosis
M2500	Haemarthrosis
M2501	Haemarthrosis
M2502	Haemarthrosis
M2503	Haemarthrosis
M2504	Haemarthrosis
M2505	Haemarthrosis
M2506	Haemarthrosis
M2507	Haemarthrosis
M2508	Haemarthrosis
M2509	Haemarthrosis
M251	Fistula of joint
M2510	Fistula of joint
M2511	Fistula of joint
M2512	Fistula of joint
M2513	Fistula of joint
M2514	Fistula of joint
M2515	Fistula of joint
M2516	Fistula of joint
M2517	Fistula of joint
M2518	Fistula of joint
M2519	Fistula of joint
M252	Flail joint
M2520	Flail joint
M2521	Flail joint
M2522	Flail joint
M2523	Flail joint
M2524	Flail joint
M2525	Flail joint
M2526	Flail joint
M2527	Flail joint
M2528	Flail joint
M2529	Flail joint
M253	Other instability of joint
M2530	Other instability of joint
M2531	Other instability of joint
M2532	Other instability of joint

For peer review only

M2533	Other instability of joint
M2534	Other instability of joint
M2535	Other instability of joint
M2536	Other instability of joint
M2537	Other instability of joint
M2538	Other instability of joint
M2539	Other instability of joint
M254	Effusion of joint
M2540	Effusion of joint
M2541	Effusion of joint
M2542	Effusion of joint
M2543	Effusion of joint
M2544	Effusion of joint
M2545	Effusion of joint
M2546	Effusion of joint
M2547	Effusion of joint
M2548	Effusion of joint
M2549	Effusion of joint
M255	Pain in joint
M2550	Pain in joint
M2551	Pain in joint
M2552	Pain in joint
M2553	Pain in joint
M2554	Pain in joint
M2555	Pain in joint
M2556	Pain in joint
M2557	Pain in joint
M2558	Pain in joint
M2559	Pain in joint
M256	Stiffness of joint, not elsewhere classified
M2560	Stiffness of joint, not elsewhere classified
M2561	Stiffness of joint, not elsewhere classified
M2562	Stiffness of joint, not elsewhere classified
M2563	Stiffness of joint, not elsewhere classified
M2564	Stiffness of joint, not elsewhere classified
M2565	Stiffness of joint, not elsewhere classified
M2566	Stiffness of joint, not elsewhere classified
M2567	Stiffness of joint, not elsewhere classified
M2568	Stiffness of joint, not elsewhere classified
M2569	Stiffness of joint, not elsewhere classified
M257	Osteophyte
M2570	Osteophyte
M2571	Osteophyte
M2572	Osteophyte
M2573	Osteophyte
M2574	Osteophyte
M2575	Osteophyte
M2576	Osteophyte

For peer review only

M2577	Osteophyte
M2578	Osteophyte
M2579	Osteophyte
M258	Other specified joint disorders
M2580	Other specified joint disorders
M2581	Other specified joint disorders
M2582	Other specified joint disorders
M2583	Other specified joint disorders
M2584	Other specified joint disorders
M2585	Other specified joint disorders
M2586	Other specified joint disorders
M2587	Other specified joint disorders
M2588	Other specified joint disorders
M2589	Other specified joint disorders
M259	Joint disorder, unspecified
M2590	Joint disorder, unspecified
M2591	Joint disorder, unspecified
M2592	Joint disorder, unspecified
M2593	Joint disorder, unspecified
M2594	Joint disorder, unspecified
M2595	Joint disorder, unspecified
M2596	Joint disorder, unspecified
M2597	Joint disorder, unspecified
M2598	Joint disorder, unspecified
M2599	Joint disorder, unspecified
M30	Polyarteritis nodosa and related conditions
M300	Polyarteritis nodosa
M301	Polyarteritis with lung involvement [Churg-Strauss]
M302	Juvenile polyarteritis
M303	Mucocutaneous lymph node syndrome [Kawasaki]
M308	Other conditions related to polyarteritis nodosa
M31	Other necrotizing vasculopathies
M310	Hypersensitivity angiitis
M311	Thrombotic microangiopathy
M312	Lethal midline granuloma
M313	Wegener granulomatosis
M313	Wegener's granulomatosis
M314	Aortic arch syndrome [Takayasu]
M315	Giant cell arteritis with polymyalgia rheumatica
M316	Other giant cell arteritis
M317	Microscopic polyangiitis
M318	Other specified necrotizing vasculopathies
M319	Necrotizing vasculopathy, unspecified
M32	Systemic lupus erythematosus
M320	Drug-induced systemic lupus erythematosus
M321	Systemic lupus erythematosus with organ or system involvement
M328	Other forms of systemic lupus erythematosus
M329	Systemic lupus erythematosus, unspecified

For peer review only

M33	Dermatopolymyositis
M330	Juvenile dermatomyositis
M331	Other dermatomyositis
M332	Polymyositis
M339	Dermatopolymyositis, unspecified
M34	Systemic sclerosis
M340	Progressive systemic sclerosis
M341	CR(E)ST syndrome
M342	Systemic sclerosis induced by drugs and chemicals
M348	Other forms of systemic sclerosis
M349	Systemic sclerosis, unspecified
M35	Other systemic involvement of connective tissue
M350	Sicca syndrome [Sj”gren]
M351	Other overlap syndromes
M352	Beh‡et disease
M352	Beh‡et's disease
M353	Polymyalgia rheumatica
M354	Diffuse (eosinophilic) fasciitis
M355	Multifocal fibrosclerosis
M356	Relapsing panniculitis [Weber-Christian]
M357	Hypermobility syndrome
M358	Other specified systemic involvement of connective tissue
M359	Systemic involvement of connective tissue, unspecified
M36	Systemic disorders of connective tissue in diseases classified elsewhere
M360	Dermato(poly)myositis in neoplastic disease
M361	Arthropathy in neoplastic disease
M362	Haemophilic arthropathy
M363	Arthropathy in other blood disorders
M364	Arthropathy in hypersensitivity reactions classified elsewhere
M368	Systemic disorders of connective tissue in other diseases classified elsewhere
M40	Kyphosis and lordosis
M400	Postural kyphosis
M4000	Postural kyphosis
M4001	Postural kyphosis
M4002	Postural kyphosis
M4003	Postural kyphosis
M4004	Postural kyphosis
M4005	Postural kyphosis
M4006	Postural kyphosis
M4007	Postural kyphosis
M4008	Postural kyphosis
M4009	Postural kyphosis
M401	Other secondary kyphosis
M4010	Other secondary kyphosis
M4011	Other secondary kyphosis
M4012	Other secondary kyphosis
M4013	Other secondary kyphosis
M4014	Other secondary kyphosis

For peer review only

M4015	Other secondary kyphosis
M4016	Other secondary kyphosis
M4017	Other secondary kyphosis
M4018	Other secondary kyphosis
M4019	Other secondary kyphosis
M402	Other and unspecified kyphosis
M4020	Other and unspecified kyphosis
M4021	Other and unspecified kyphosis
M4022	Other and unspecified kyphosis
M4023	Other and unspecified kyphosis
M4024	Other and unspecified kyphosis
M4025	Other and unspecified kyphosis
M4026	Other and unspecified kyphosis
M4027	Other and unspecified kyphosis
M4028	Other and unspecified kyphosis
M4029	Other and unspecified kyphosis
M403	Flatback syndrome
M4030	Flatback syndrome
M4031	Flatback syndrome
M4032	Flatback syndrome
M4033	Flatback syndrome
M4034	Flatback syndrome
M4035	Flatback syndrome
M4036	Flatback syndrome
M4037	Flatback syndrome
M4038	Flatback syndrome
M4039	Flatback syndrome
M404	Other lordosis
M4040	Other lordosis
M4041	Other lordosis
M4042	Other lordosis
M4043	Other lordosis
M4044	Other lordosis
M4045	Other lordosis
M4046	Other lordosis
M4047	Other lordosis
M4048	Other lordosis
M4049	Other lordosis
M405	Lordosis, unspecified
M4050	Lordosis, unspecified
M4051	Lordosis, unspecified
M4052	Lordosis, unspecified
M4053	Lordosis, unspecified
M4054	Lordosis, unspecified
M4055	Lordosis, unspecified
M4056	Lordosis, unspecified
M4057	Lordosis, unspecified
M4058	Lordosis, unspecified

For peer review only

M4059	Lordosis, unspecified
M41	Scoliosis
M410	Infantile idiopathic scoliosis
M4100	Infantile idiopathic scoliosis
M4101	Infantile idiopathic scoliosis
M4102	Infantile idiopathic scoliosis
M4103	Infantile idiopathic scoliosis
M4104	Infantile idiopathic scoliosis
M4105	Infantile idiopathic scoliosis
M4106	Infantile idiopathic scoliosis
M4107	Infantile idiopathic scoliosis
M4108	Infantile idiopathic scoliosis
M4109	Infantile idiopathic scoliosis
M411	Juvenile idiopathic scoliosis
M4110	Juvenile idiopathic scoliosis
M4111	Juvenile idiopathic scoliosis
M4112	Juvenile idiopathic scoliosis
M4113	Juvenile idiopathic scoliosis
M4114	Juvenile idiopathic scoliosis
M4115	Juvenile idiopathic scoliosis
M4116	Juvenile idiopathic scoliosis
M4117	Juvenile idiopathic scoliosis
M4118	Juvenile idiopathic scoliosis
M4119	Juvenile idiopathic scoliosis
M412	Other idiopathic scoliosis
M4120	Other idiopathic scoliosis
M4121	Other idiopathic scoliosis
M4122	Other idiopathic scoliosis
M4123	Other idiopathic scoliosis
M4124	Other idiopathic scoliosis
M4125	Other idiopathic scoliosis
M4126	Other idiopathic scoliosis
M4127	Other idiopathic scoliosis
M4128	Other idiopathic scoliosis
M4129	Other idiopathic scoliosis
M413	Thoracogenic scoliosis
M4130	Thoracogenic scoliosis
M4133	Thoracogenic scoliosis
M4134	Thoracogenic scoliosis
M4135	Thoracogenic scoliosis
M414	Neuromuscular scoliosis
M4140	Neuromuscular scoliosis
M4141	Neuromuscular scoliosis
M4142	Neuromuscular scoliosis
M4143	Neuromuscular scoliosis
M4144	Neuromuscular scoliosis
M4145	Neuromuscular scoliosis
M4146	Neuromuscular scoliosis

For peer review only

M4147	Neuromuscular scoliosis
M4148	Neuromuscular scoliosis
M4149	Neuromuscular scoliosis
M415	Other secondary scoliosis
M4150	Other secondary scoliosis
M4151	Other secondary scoliosis
M4152	Other secondary scoliosis
M4153	Other secondary scoliosis
M4154	Other secondary scoliosis
M4155	Other secondary scoliosis
M4156	Other secondary scoliosis
M4157	Other secondary scoliosis
M4158	Other secondary scoliosis
M4159	Other secondary scoliosis
M418	Other forms of scoliosis
M4180	Other forms of scoliosis
M4181	Other forms of scoliosis
M4182	Other forms of scoliosis
M4183	Other forms of scoliosis
M4184	Other forms of scoliosis
M4185	Other forms of scoliosis
M4186	Other forms of scoliosis
M4187	Other forms of scoliosis
M4188	Other forms of scoliosis
M4189	Other forms of scoliosis
M419	Scoliosis, unspecified
M4190	Scoliosis, unspecified
M4191	Scoliosis, unspecified
M4192	Scoliosis, unspecified
M4193	Scoliosis, unspecified
M4194	Scoliosis, unspecified
M4195	Scoliosis, unspecified
M4196	Scoliosis, unspecified
M4197	Scoliosis, unspecified
M4198	Scoliosis, unspecified
M4199	Scoliosis, unspecified
M42	Spinal osteochondrosis
M420	Juvenile osteochondrosis of spine
M4200	Juvenile osteochondrosis of spine
M4201	Juvenile osteochondrosis of spine
M4202	Juvenile osteochondrosis of spine
M4203	Juvenile osteochondrosis of spine
M4204	Juvenile osteochondrosis of spine
M4205	Juvenile osteochondrosis of spine
M4206	Juvenile osteochondrosis of spine
M4207	Juvenile osteochondrosis of spine
M4208	Juvenile osteochondrosis of spine
M4209	Juvenile osteochondrosis of spine

For peer review only

M421	Adult osteochondrosis of spine
M4210	Adult osteochondrosis of spine
M4211	Adult osteochondrosis of spine
M4212	Adult osteochondrosis of spine
M4213	Adult osteochondrosis of spine
M4214	Adult osteochondrosis of spine
M4215	Adult osteochondrosis of spine
M4216	Adult osteochondrosis of spine
M4217	Adult osteochondrosis of spine
M4218	Adult osteochondrosis of spine
M4219	Adult osteochondrosis of spine
M429	Spinal osteochondrosis, unspecified
M4290	Spinal osteochondrosis, unspecified
M4291	Spinal osteochondrosis, unspecified
M4292	Spinal osteochondrosis, unspecified
M4293	Spinal osteochondrosis, unspecified
M4294	Spinal osteochondrosis, unspecified
M4295	Spinal osteochondrosis, unspecified
M4296	Spinal osteochondrosis, unspecified
M4297	Spinal osteochondrosis, unspecified
M4298	Spinal osteochondrosis, unspecified
M4299	Spinal osteochondrosis, unspecified
M43	Other deforming dorsopathies
M430	Spondylolysis
M4300	Spondylolysis
M4301	Spondylolysis
M4302	Spondylolysis
M4303	Spondylolysis
M4304	Spondylolysis
M4305	Spondylolysis
M4306	Spondylolysis
M4307	Spondylolysis
M4308	Spondylolysis
M4309	Spondylolysis
M431	Spondylolisthesis
M4310	Spondylolisthesis
M4311	Spondylolisthesis
M4312	Spondylolisthesis
M4313	Spondylolisthesis
M4314	Spondylolisthesis
M4315	Spondylolisthesis
M4316	Spondylolisthesis
M4317	Spondylolisthesis
M4318	Spondylolisthesis
M4319	Spondylolisthesis
M432	Other fusion of spine
M4320	Other fusion of spine
M4321	Other fusion of spine

For peer review only

M4322	Other fusion of spine
M4323	Other fusion of spine
M4324	Other fusion of spine
M4325	Other fusion of spine
M4326	Other fusion of spine
M4327	Other fusion of spine
M4328	Other fusion of spine
M4329	Other fusion of spine
M433	Recurrent atlantoaxial subluxation with myelopathy
M4330	Recurrent atlantoaxial subluxation with myelopathy
M4331	Recurrent atlantoaxial subluxation with myelopathy
M4332	Recurrent atlantoaxial subluxation with myelopathy
M4333	Recurrent atlantoaxial subluxation with myelopathy
M4334	Recurrent atlantoaxial subluxation with myelopathy
M4335	Recurrent atlantoaxial subluxation with myelopathy
M4336	Recurrent atlantoaxial subluxation with myelopathy
M4337	Recurrent atlantoaxial subluxation with myelopathy
M4338	Recurrent atlantoaxial subluxation with myelopathy
M4339	Recurrent atlantoaxial subluxation with myelopathy
M434	Other recurrent atlantoaxial subluxation
M435	Other recurrent vertebral subluxation
M4350	Other recurrent vertebral subluxation
M4352	Other recurrent vertebral subluxation
M4353	Other recurrent vertebral subluxation
M4354	Other recurrent vertebral subluxation
M4355	Other recurrent vertebral subluxation
M4356	Other recurrent vertebral subluxation
M4357	Other recurrent vertebral subluxation
M4358	Other recurrent vertebral subluxation
M4359	Other recurrent vertebral subluxation
M436	Torticollis
M438	Other specified deforming dorsopathies
M4380	Other specified deforming dorsopathies
M4381	Other specified deforming dorsopathies
M4382	Other specified deforming dorsopathies
M4383	Other specified deforming dorsopathies
M4384	Other specified deforming dorsopathies
M4385	Other specified deforming dorsopathies
M4386	Other specified deforming dorsopathies
M4387	Other specified deforming dorsopathies
M4388	Other specified deforming dorsopathies
M4389	Other specified deforming dorsopathies
M439	Deforming dorsopathy, unspecified
M4390	Deforming dorsopathy, unspecified
M4391	Deforming dorsopathy, unspecified
M4392	Deforming dorsopathy, unspecified
M4393	Deforming dorsopathy, unspecified
M4394	Deforming dorsopathy, unspecified

For peer review only

M4395	Deforming dorsopathy, unspecified
M4396	Deforming dorsopathy, unspecified
M4397	Deforming dorsopathy, unspecified
M4398	Deforming dorsopathy, unspecified
M4399	Deforming dorsopathy, unspecified
M45X	Ankylosing spondylitis
M45X0	Ankylosing spondylitis
M45X1	Ankylosing spondylitis
M45X2	Ankylosing spondylitis
M45X3	Ankylosing spondylitis
M45X4	Ankylosing spondylitis
M45X5	Ankylosing spondylitis
M45X6	Ankylosing spondylitis
M45X7	Ankylosing spondylitis
M45X8	Ankylosing spondylitis
M45X9	Ankylosing spondylitis
M46	Other inflammatory spondylopathies
M460	Spinal enthesopathy
M4600	Spinal enthesopathy
M4601	Spinal enthesopathy
M4602	Spinal enthesopathy
M4603	Spinal enthesopathy
M4604	Spinal enthesopathy
M4605	Spinal enthesopathy
M4606	Spinal enthesopathy
M4607	Spinal enthesopathy
M4608	Spinal enthesopathy
M4609	Spinal enthesopathy
M461	Sacroiliitis, not elsewhere classified
M462	Osteomyelitis of vertebra
M4620	Osteomyelitis of vertebra
M4621	Osteomyelitis of vertebra
M4622	Osteomyelitis of vertebra
M4623	Osteomyelitis of vertebra
M4624	Osteomyelitis of vertebra
M4625	Osteomyelitis of vertebra
M4626	Osteomyelitis of vertebra
M4627	Osteomyelitis of vertebra
M4628	Osteomyelitis of vertebra
M4629	Osteomyelitis of vertebra
M463	Infection of intervertebral disc (pyogenic)
M4630	Infection of intervertebral disc (pyogenic)
M4631	Infection of intervertebral disc (pyogenic)
M4632	Infection of intervertebral disc (pyogenic)
M4633	Infection of intervertebral disc (pyogenic)
M4634	Infection of intervertebral disc (pyogenic)
M4635	Infection of intervertebral disc (pyogenic)
M4636	Infection of intervertebral disc (pyogenic)

For peer review only

M4637	Infection of intervertebral disc (pyogenic)
M4638	Infection of intervertebral disc (pyogenic)
M4639	Infection of intervertebral disc (pyogenic)
M464	Discitis, unspecified
M4640	Discitis, unspecified
M4641	Discitis, unspecified
M4642	Discitis, unspecified
M4643	Discitis, unspecified
M4644	Discitis, unspecified
M4645	Discitis, unspecified
M4646	Discitis, unspecified
M4647	Discitis, unspecified
M4648	Discitis, unspecified
M4649	Discitis, unspecified
M465	Other infective spondylopathies
M4650	Other infective spondylopathies
M4651	Other infective spondylopathies
M4652	Other infective spondylopathies
M4653	Other infective spondylopathies
M4654	Other infective spondylopathies
M4655	Other infective spondylopathies
M4656	Other infective spondylopathies
M4657	Other infective spondylopathies
M4658	Other infective spondylopathies
M4659	Other infective spondylopathies
M468	Other specified inflammatory spondylopathies
M4680	Other specified inflammatory spondylopathies
M4681	Other specified inflammatory spondylopathies
M4682	Other specified inflammatory spondylopathies
M4683	Other specified inflammatory spondylopathies
M4684	Other specified inflammatory spondylopathies
M4685	Other specified inflammatory spondylopathies
M4686	Other specified inflammatory spondylopathies
M4687	Other specified inflammatory spondylopathies
M4688	Other specified inflammatory spondylopathies
M4689	Other specified inflammatory spondylopathies
M469	Inflammatory spondylopathy, unspecified
M4690	Inflammatory spondylopathy, unspecified
M4691	Inflammatory spondylopathy, unspecified
M4692	Inflammatory spondylopathy, unspecified
M4693	Inflammatory spondylopathy, unspecified
M4694	Inflammatory spondylopathy, unspecified
M4695	Inflammatory spondylopathy, unspecified
M4696	Inflammatory spondylopathy, unspecified
M4697	Inflammatory spondylopathy, unspecified
M4698	Inflammatory spondylopathy, unspecified
M4699	Inflammatory spondylopathy, unspecified
M47	Spondylosis

For peer review only

M470	Anterior spinal and vertebral artery compression syndromes
M4700	Anterior spinal and vertebral artery compression syndromes
M4701	Anterior spinal and vertebral artery compression syndromes
M4702	Anterior spinal and vertebral artery compression syndromes
M4703	Anterior spinal and vertebral artery compression syndromes
M4704	Anterior spinal and vertebral artery compression syndromes
M4705	Anterior spinal and vertebral artery compression syndromes
M4706	Anterior spinal and vertebral artery compression syndromes
M4707	Anterior spinal and vertebral artery compression syndromes
M4708	Anterior spinal and vertebral artery compression syndromes
M4709	Anterior spinal and vertebral artery compression syndromes
M471	Other spondylosis with myelopathy
M4710	Other spondylosis with myelopathy
M4711	Other spondylosis with myelopathy
M4712	Other spondylosis with myelopathy
M4713	Other spondylosis with myelopathy
M4714	Other spondylosis with myelopathy
M4715	Other spondylosis with myelopathy
M4716	Other spondylosis with myelopathy
M4717	Other spondylosis with myelopathy
M4718	Other spondylosis with myelopathy
M4719	Other spondylosis with myelopathy
M472	Other spondylosis with radiculopathy
M4720	Other spondylosis with radiculopathy
M4721	Other spondylosis with radiculopathy
M4722	Other spondylosis with radiculopathy
M4723	Other spondylosis with radiculopathy
M4724	Other spondylosis with radiculopathy
M4725	Other spondylosis with radiculopathy
M4726	Other spondylosis with radiculopathy
M4727	Other spondylosis with radiculopathy
M4728	Other spondylosis with radiculopathy
M4729	Other spondylosis with radiculopathy
M478	Other spondylosis
M4780	Other spondylosis
M4781	Other spondylosis
M4782	Other spondylosis
M4783	Other spondylosis
M4784	Other spondylosis
M4785	Other spondylosis
M4786	Other spondylosis
M4787	Other spondylosis
M4788	Other spondylosis
M4789	Other spondylosis
M479	Spondylosis, unspecified
M4790	Spondylosis, unspecified
M4791	Spondylosis, unspecified
M4792	Spondylosis, unspecified

For peer review only

M4793	Spondylosis, unspecified
M4794	Spondylosis, unspecified
M4795	Spondylosis, unspecified
M4796	Spondylosis, unspecified
M4797	Spondylosis, unspecified
M4798	Spondylosis, unspecified
M4799	Spondylosis, unspecified
M48	Other spondylopathies
M480	Spinal stenosis
M4800	Spinal stenosis
M4801	Spinal stenosis
M4802	Spinal stenosis
M4803	Spinal stenosis
M4804	Spinal stenosis
M4805	Spinal stenosis
M4806	Spinal stenosis
M4807	Spinal stenosis
M4808	Spinal stenosis
M4809	Spinal stenosis
M481	Ankylosing hyperostosis [Forestier]
M4810	Ankylosing hyperostosis [Forestier]
M4811	Ankylosing hyperostosis [Forestier]
M4812	Ankylosing hyperostosis [Forestier]
M4813	Ankylosing hyperostosis [Forestier]
M4814	Ankylosing hyperostosis [Forestier]
M4815	Ankylosing hyperostosis [Forestier]
M4816	Ankylosing hyperostosis [Forestier]
M4817	Ankylosing hyperostosis [Forestier]
M4818	Ankylosing hyperostosis [Forestier]
M4819	Ankylosing hyperostosis [Forestier]
M482	Kissing spine
M4820	Kissing spine
M4821	Kissing spine
M4822	Kissing spine
M4823	Kissing spine
M4824	Kissing spine
M4825	Kissing spine
M4826	Kissing spine
M4827	Kissing spine
M4828	Kissing spine
M4829	Kissing spine
M483	Traumatic spondylopathy
M4830	Traumatic spondylopathy
M4831	Traumatic spondylopathy
M4832	Traumatic spondylopathy
M4833	Traumatic spondylopathy
M4834	Traumatic spondylopathy
M4835	Traumatic spondylopathy

For peer review only

M4836	Traumatic spondylopathy
M4837	Traumatic spondylopathy
M4838	Traumatic spondylopathy
M4839	Traumatic spondylopathy
M484	Fatigue fracture of vertebra
M4840	Fatigue fracture of vertebra
M4841	Fatigue fracture of vertebra
M4842	Fatigue fracture of vertebra
M4843	Fatigue fracture of vertebra
M4844	Fatigue fracture of vertebra
M4845	Fatigue fracture of vertebra
M4846	Fatigue fracture of vertebra
M4847	Fatigue fracture of vertebra
M4848	Fatigue fracture of vertebra
M4849	Fatigue fracture of vertebra
M485	Collapsed vertebra, not elsewhere classified
M4850	Collapsed vertebra, not elsewhere classified
M4851	Collapsed vertebra, not elsewhere classified
M4852	Collapsed vertebra, not elsewhere classified
M4853	Collapsed vertebra, not elsewhere classified
M4854	Collapsed vertebra, not elsewhere classified
M4855	Collapsed vertebra, not elsewhere classified
M4856	Collapsed vertebra, not elsewhere classified
M4857	Collapsed vertebra, not elsewhere classified
M4858	Collapsed vertebra, not elsewhere classified
M4859	Collapsed vertebra, not elsewhere classified
M488	Other specified spondylopathies
M4880	Other specified spondylopathies
M4881	Other specified spondylopathies
M4882	Other specified spondylopathies
M4883	Other specified spondylopathies
M4884	Other specified spondylopathies
M4885	Other specified spondylopathies
M4886	Other specified spondylopathies
M4887	Other specified spondylopathies
M4888	Other specified spondylopathies
M4889	Other specified spondylopathies
M489	Spondylopathy, unspecified
M4890	Spondylopathy, unspecified
M4891	Spondylopathy, unspecified
M4892	Spondylopathy, unspecified
M4893	Spondylopathy, unspecified
M4894	Spondylopathy, unspecified
M4895	Spondylopathy, unspecified
M4896	Spondylopathy, unspecified
M4897	Spondylopathy, unspecified
M4898	Spondylopathy, unspecified
M4899	Spondylopathy, unspecified

For peer review only

M49	Spondylopathies in diseases classified elsewhere
M490	Tuberculosis of spine
M4900	Tuberculosis of spine
M4901	Tuberculosis of spine
M4902	Tuberculosis of spine
M4903	Tuberculosis of spine
M4904	Tuberculosis of spine
M4905	Tuberculosis of spine
M4906	Tuberculosis of spine
M4907	Tuberculosis of spine
M4908	Tuberculosis of spine
M4909	Tuberculosis of spine
M491	Brucella spondylitis
M4910	Brucella spondylitis
M4911	Brucella spondylitis
M4912	Brucella spondylitis
M4913	Brucella spondylitis
M4914	Brucella spondylitis
M4915	Brucella spondylitis
M4916	Brucella spondylitis
M4917	Brucella spondylitis
M4918	Brucella spondylitis
M4919	Brucella spondylitis
M492	Enterobacterial spondylitis
M4920	Enterobacterial spondylitis
M4921	Enterobacterial spondylitis
M4922	Enterobacterial spondylitis
M4923	Enterobacterial spondylitis
M4924	Enterobacterial spondylitis
M4925	Enterobacterial spondylitis
M4926	Enterobacterial spondylitis
M4927	Enterobacterial spondylitis
M4928	Enterobacterial spondylitis
M4929	Enterobacterial spondylitis
M493	Spondylopathy in other infectious and parasitic diseases classified elsewhere
M4930	Spondylopathy in other infectious and parasitic diseases classified elsewhere
M4931	Spondylopathy in other infectious and parasitic diseases classified elsewhere
M4932	Spondylopathy in other infectious and parasitic diseases classified elsewhere
M4933	Spondylopathy in other infectious and parasitic diseases classified elsewhere
M4934	Spondylopathy in other infectious and parasitic diseases classified elsewhere
M4935	Spondylopathy in other infectious and parasitic diseases classified elsewhere
M4936	Spondylopathy in other infectious and parasitic diseases classified elsewhere
M4937	Spondylopathy in other infectious and parasitic diseases classified elsewhere
M4938	Spondylopathy in other infectious and parasitic diseases classified elsewhere
M4939	Spondylopathy in other infectious and parasitic diseases classified elsewhere
M494	Neuropathic spondylopathy
M4940	Neuropathic spondylopathy
M4941	Neuropathic spondylopathy

For peer review only

M4942	Neuropathic spondylopathy
M4943	Neuropathic spondylopathy
M4944	Neuropathic spondylopathy
M4945	Neuropathic spondylopathy
M4946	Neuropathic spondylopathy
M4947	Neuropathic spondylopathy
M4948	Neuropathic spondylopathy
M4949	Neuropathic spondylopathy
M495	Collapsed vertebra in diseases classified elsewhere
M4950	Collapsed vertebra in diseases classified elsewhere
M4951	Collapsed vertebra in diseases classified elsewhere
M4952	Collapsed vertebra in diseases classified elsewhere
M4953	Collapsed vertebra in diseases classified elsewhere
M4954	Collapsed vertebra in diseases classified elsewhere
M4955	Collapsed vertebra in diseases classified elsewhere
M4956	Collapsed vertebra in diseases classified elsewhere
M4957	Collapsed vertebra in diseases classified elsewhere
M4958	Collapsed vertebra in diseases classified elsewhere
M4959	Collapsed vertebra in diseases classified elsewhere
M498	Spondylopathy in other diseases classified elsewhere
M4980	Spondylopathy in other diseases classified elsewhere
M4981	Spondylopathy in other diseases classified elsewhere
M4982	Spondylopathy in other diseases classified elsewhere
M4983	Spondylopathy in other diseases classified elsewhere
M4984	Spondylopathy in other diseases classified elsewhere
M4985	Spondylopathy in other diseases classified elsewhere
M4986	Spondylopathy in other diseases classified elsewhere
M4987	Spondylopathy in other diseases classified elsewhere
M4988	Spondylopathy in other diseases classified elsewhere
M4989	Spondylopathy in other diseases classified elsewhere
M50	Cervical disc disorders
M500	Cervical disc disorder with myelopathy
M501	Cervical disc disorder with radiculopathy
M502	Other cervical disc displacement
M503	Other cervical disc degeneration
M508	Other cervical disc disorders
M509	Cervical disc disorder, unspecified
M51	Other intervertebral disc disorders
M510	Lumbar and other intervertebral disc disorders with myelopathy
M511	Lumbar and other intervertebral disc disorders with radiculopathy
M512	Other specified intervertebral disc displacement
M513	Other specified intervertebral disc degeneration
M514	Schmorl nodes
M514	Schmorl's nodes
M518	Other specified intervertebral disc disorders
M519	Intervertebral disc disorder, unspecified
M53	Other dorsopathies, not elsewhere classified
M530	Cervicocranial syndrome

For peer review only

M5300	Cervicocranial syndrome
M5301	Cervicocranial syndrome
M5302	Cervicocranial syndrome
M5303	Cervicocranial syndrome
M531	Cervicobrachial syndrome
M5310	Cervicobrachial syndrome
M5311	Cervicobrachial syndrome
M5312	Cervicobrachial syndrome
M5313	Cervicobrachial syndrome
M532	Spinal instabilities
M5320	Spinal instabilities
M5321	Spinal instabilities
M5322	Spinal instabilities
M5323	Spinal instabilities
M5324	Spinal instabilities
M5325	Spinal instabilities
M5326	Spinal instabilities
M5327	Spinal instabilities
M5328	Spinal instabilities
M5329	Spinal instabilities
M533	Sacrococcygeal disorders, not elsewhere classified
M538	Other specified dorsopathies
M5380	Other specified dorsopathies
M5381	Other specified dorsopathies
M5382	Other specified dorsopathies
M5383	Other specified dorsopathies
M5384	Other specified dorsopathies
M5385	Other specified dorsopathies
M5386	Other specified dorsopathies
M5387	Other specified dorsopathies
M5388	Other specified dorsopathies
M5389	Other specified dorsopathies
M539	Dorsopathy, unspecified
M5390	Dorsopathy, unspecified
M5391	Dorsopathy, unspecified
M5392	Dorsopathy, unspecified
M5393	Dorsopathy, unspecified
M5394	Dorsopathy, unspecified
M5395	Dorsopathy, unspecified
M5396	Dorsopathy, unspecified
M5397	Dorsopathy, unspecified
M5398	Dorsopathy, unspecified
M5399	Dorsopathy, unspecified
M54	Dorsalgia
M540	Panniculitis affecting regions of neck and back
M5400	Panniculitis affecting regions of neck and back
M5401	Panniculitis affecting regions of neck and back
M5402	Panniculitis affecting regions of neck and back

For peer review only

M5403	Panniculitis affecting regions of neck and back
M5404	Panniculitis affecting regions of neck and back
M5405	Panniculitis affecting regions of neck and back
M5406	Panniculitis affecting regions of neck and back
M5407	Panniculitis affecting regions of neck and back
M5408	Panniculitis affecting regions of neck and back
M5409	Panniculitis affecting regions of neck and back
M541	Radiculopathy
M5410	Radiculopathy
M5411	Radiculopathy
M5412	Radiculopathy
M5413	Radiculopathy
M5414	Radiculopathy
M5415	Radiculopathy
M5416	Radiculopathy
M5417	Radiculopathy
M5418	Radiculopathy
M5419	Radiculopathy
M542	Cervicalgia
M5421	Cervicalgia
M5422	Cervicalgia
M5423	Cervicalgia
M543	Sciatica
M5430	Sciatica
M5435	Sciatica
M5436	Sciatica
M5437	Sciatica
M5438	Sciatica
M5439	Sciatica
M544	Lumbago with sciatica
M5440	Lumbago with sciatica
M5445	Lumbago with sciatica
M5446	Lumbago with sciatica
M5447	Lumbago with sciatica
M5448	Lumbago with sciatica
M5449	Lumbago with sciatica
M545	Low back pain
M5450	Low back pain
M5455	Low back pain
M5456	Low back pain
M5457	Low back pain
M5458	Low back pain
M5459	Low back pain
M546	Pain in thoracic spine
M5460	Pain in thoracic spine
M5463	Pain in thoracic spine
M5464	Pain in thoracic spine
M5465	Pain in thoracic spine

For peer review only

M548	Other dorsalgia
M5480	Other dorsalgia
M5481	Other dorsalgia
M5482	Other dorsalgia
M5483	Other dorsalgia
M5484	Other dorsalgia
M5485	Other dorsalgia
M5486	Other dorsalgia
M5487	Other dorsalgia
M5488	Other dorsalgia
M5489	Other dorsalgia
M549	Dorsalgia, unspecified
M5490	Dorsalgia, unspecified
M5491	Dorsalgia, unspecified
M5492	Dorsalgia, unspecified
M5493	Dorsalgia, unspecified
M5494	Dorsalgia, unspecified
M5495	Dorsalgia, unspecified
M5496	Dorsalgia, unspecified
M5497	Dorsalgia, unspecified
M5498	Dorsalgia, unspecified
M5499	Dorsalgia, unspecified
M60	Myositis
M600	Infective myositis
M6000	Infective myositis
M6001	Infective myositis
M6002	Infective myositis
M6003	Infective myositis
M6004	Infective myositis
M6005	Infective myositis
M6006	Infective myositis
M6007	Infective myositis
M6008	Infective myositis
M6009	Infective myositis
M601	Interstitial myositis
M6010	Interstitial myositis
M6011	Interstitial myositis
M6012	Interstitial myositis
M6013	Interstitial myositis
M6014	Interstitial myositis
M6015	Interstitial myositis
M6016	Interstitial myositis
M6017	Interstitial myositis
M6018	Interstitial myositis
M6019	Interstitial myositis
M602	Foreign body granuloma of soft tissue, not elsewhere classified
M6020	Foreign body granuloma of soft tissue, not elsewhere classified
M6021	Foreign body granuloma of soft tissue, not elsewhere classified

For peer review only

M6022	Foreign body granuloma of soft tissue, not elsewhere classified
M6023	Foreign body granuloma of soft tissue, not elsewhere classified
M6024	Foreign body granuloma of soft tissue, not elsewhere classified
M6025	Foreign body granuloma of soft tissue, not elsewhere classified
M6026	Foreign body granuloma of soft tissue, not elsewhere classified
M6027	Foreign body granuloma of soft tissue, not elsewhere classified
M6028	Foreign body granuloma of soft tissue, not elsewhere classified
M6029	Foreign body granuloma of soft tissue, not elsewhere classified
M608	Other myositis
M6080	Other myositis
M6081	Other myositis
M6082	Other myositis
M6083	Other myositis
M6084	Other myositis
M6085	Other myositis
M6086	Other myositis
M6087	Other myositis
M6088	Other myositis
M6089	Other myositis
M609	Myositis, unspecified
M6090	Myositis, unspecified
M6091	Myositis, unspecified
M6092	Myositis, unspecified
M6093	Myositis, unspecified
M6094	Myositis, unspecified
M6095	Myositis, unspecified
M6096	Myositis, unspecified
M6097	Myositis, unspecified
M6098	Myositis, unspecified
M6099	Myositis, unspecified
M61	Calcification and ossification of muscle
M610	Myositis ossificans traumatica
M6100	Myositis ossificans traumatica
M6101	Myositis ossificans traumatica
M6102	Myositis ossificans traumatica
M6103	Myositis ossificans traumatica
M6104	Myositis ossificans traumatica
M6105	Myositis ossificans traumatica
M6106	Myositis ossificans traumatica
M6107	Myositis ossificans traumatica
M6108	Myositis ossificans traumatica
M6109	Myositis ossificans traumatica
M611	Myositis ossificans progressiva
M6110	Myositis ossificans progressiva
M6111	Myositis ossificans progressiva
M6112	Myositis ossificans progressiva
M6113	Myositis ossificans progressiva
M6114	Myositis ossificans progressiva

For peer review only

M6115	Myositis ossificans progressiva
M6116	Myositis ossificans progressiva
M6117	Myositis ossificans progressiva
M6118	Myositis ossificans progressiva
M6119	Myositis ossificans progressiva
M612	Paralytic calcification and ossification of muscle
M6120	Paralytic calcification and ossification of muscle
M6121	Paralytic calcification and ossification of muscle
M6122	Paralytic calcification and ossification of muscle
M6123	Paralytic calcification and ossification of muscle
M6124	Paralytic calcification and ossification of muscle
M6125	Paralytic calcification and ossification of muscle
M6126	Paralytic calcification and ossification of muscle
M6127	Paralytic calcification and ossification of muscle
M6128	Paralytic calcification and ossification of muscle
M6129	Paralytic calcification and ossification of muscle
M613	Calcification and ossification of muscles associated with burns
M6130	Calcification and ossification of muscles associated with burns
M6131	Calcification and ossification of muscles associated with burns
M6132	Calcification and ossification of muscles associated with burns
M6133	Calcification and ossification of muscles associated with burns
M6134	Calcification and ossification of muscles associated with burns
M6135	Calcification and ossification of muscles associated with burns
M6136	Calcification and ossification of muscles associated with burns
M6137	Calcification and ossification of muscles associated with burns
M6138	Calcification and ossification of muscles associated with burns
M6139	Calcification and ossification of muscles associated with burns
M614	Other calcification of muscle
M6140	Other calcification of muscle
M6141	Other calcification of muscle
M6142	Other calcification of muscle
M6143	Other calcification of muscle
M6144	Other calcification of muscle
M6145	Other calcification of muscle
M6146	Other calcification of muscle
M6147	Other calcification of muscle
M6148	Other calcification of muscle
M6149	Other calcification of muscle
M615	Other ossification of muscle
M6150	Other ossification of muscle
M6151	Other ossification of muscle
M6152	Other ossification of muscle
M6153	Other ossification of muscle
M6154	Other ossification of muscle
M6155	Other ossification of muscle
M6156	Other ossification of muscle
M6157	Other ossification of muscle
M6158	Other ossification of muscle

For peer review only

M6159	Other ossification of muscle
M619	Calcification and ossification of muscle, unspecified
M6190	Calcification and ossification of muscle, unspecified
M6191	Calcification and ossification of muscle, unspecified
M6192	Calcification and ossification of muscle, unspecified
M6193	Calcification and ossification of muscle, unspecified
M6194	Calcification and ossification of muscle, unspecified
M6195	Calcification and ossification of muscle, unspecified
M6196	Calcification and ossification of muscle, unspecified
M6197	Calcification and ossification of muscle, unspecified
M6198	Calcification and ossification of muscle, unspecified
M6199	Calcification and ossification of muscle, unspecified
M62	Other disorders of muscle
M620	Diastasis of muscle
M6200	Diastasis of muscle
M6201	Diastasis of muscle
M6202	Diastasis of muscle
M6203	Diastasis of muscle
M6204	Diastasis of muscle
M6205	Diastasis of muscle
M6206	Diastasis of muscle
M6207	Diastasis of muscle
M6208	Diastasis of muscle
M6209	Diastasis of muscle
M621	Other rupture of muscle (nontraumatic)
M6210	Other rupture of muscle (nontraumatic)
M6211	Other rupture of muscle (nontraumatic)
M6212	Other rupture of muscle (nontraumatic)
M6213	Other rupture of muscle (nontraumatic)
M6214	Other rupture of muscle (nontraumatic)
M6215	Other rupture of muscle (nontraumatic)
M6216	Other rupture of muscle (nontraumatic)
M6217	Other rupture of muscle (nontraumatic)
M6218	Other rupture of muscle (nontraumatic)
M6219	Other rupture of muscle (nontraumatic)
M622	Ischaemic infarction of muscle
M6220	Ischaemic infarction of muscle
M6221	Ischaemic infarction of muscle
M6222	Ischaemic infarction of muscle
M6223	Ischaemic infarction of muscle
M6224	Ischaemic infarction of muscle
M6225	Ischaemic infarction of muscle
M6226	Ischaemic infarction of muscle
M6227	Ischaemic infarction of muscle
M6228	Ischaemic infarction of muscle
M6229	Ischaemic infarction of muscle
M623	Immobility syndrome (paraplegic)
M6230	Immobility syndrome (paraplegic)

For peer review only

M6231	Immobility syndrome (paraplegic)
M6232	Immobility syndrome (paraplegic)
M6233	Immobility syndrome (paraplegic)
M6234	Immobility syndrome (paraplegic)
M6235	Immobility syndrome (paraplegic)
M6236	Immobility syndrome (paraplegic)
M6237	Immobility syndrome (paraplegic)
M6238	Immobility syndrome (paraplegic)
M6239	Immobility syndrome (paraplegic)
M624	Contracture of muscle
M6240	Contracture of muscle
M6241	Contracture of muscle
M6242	Contracture of muscle
M6243	Contracture of muscle
M6244	Contracture of muscle
M6245	Contracture of muscle
M6246	Contracture of muscle
M6247	Contracture of muscle
M6248	Contracture of muscle
M6249	Contracture of muscle
M625	Muscle wasting and atrophy, not elsewhere classified
M6250	Muscle wasting and atrophy, not elsewhere classified
M6251	Muscle wasting and atrophy, not elsewhere classified
M6252	Muscle wasting and atrophy, not elsewhere classified
M6253	Muscle wasting and atrophy, not elsewhere classified
M6254	Muscle wasting and atrophy, not elsewhere classified
M6255	Muscle wasting and atrophy, not elsewhere classified
M6256	Muscle wasting and atrophy, not elsewhere classified
M6257	Muscle wasting and atrophy, not elsewhere classified
M6258	Muscle wasting and atrophy, not elsewhere classified
M6259	Muscle wasting and atrophy, not elsewhere classified
M626	Muscle strain
M6260	Muscle strain
M6261	Muscle strain
M6262	Muscle strain
M6263	Muscle strain
M6264	Muscle strain
M6265	Muscle strain
M6266	Muscle strain
M6267	Muscle strain
M6268	Muscle strain
M6269	Muscle strain
M628	Other specified disorders of muscle
M6280	Other specified disorders of muscle
M6281	Other specified disorders of muscle
M6282	Other specified disorders of muscle
M6283	Other specified disorders of muscle
M6284	Other specified disorders of muscle

For peer review only

M6285	Other specified disorders of muscle
M6286	Other specified disorders of muscle
M6287	Other specified disorders of muscle
M6288	Other specified disorders of muscle
M6289	Other specified disorders of muscle
M629	Disorder of muscle, unspecified
M6290	Disorder of muscle, unspecified
M6291	Disorder of muscle, unspecified
M6292	Disorder of muscle, unspecified
M6293	Disorder of muscle, unspecified
M6294	Disorder of muscle, unspecified
M6295	Disorder of muscle, unspecified
M6296	Disorder of muscle, unspecified
M6297	Disorder of muscle, unspecified
M6298	Disorder of muscle, unspecified
M6299	Disorder of muscle, unspecified
M63	Disorders of muscle in diseases classified elsewhere
M630	Myositis in bacterial diseases classified elsewhere
M631	Myositis in protozoal and parasitic infections classified elsewhere
M632	Myositis in other infectious diseases classified elsewhere
M633	Myositis in sarcoidosis
M638	Other disorders of muscle in diseases classified elsewhere
M65	Synovitis and tenosynovitis
M650	Abscess of tendon sheath
M6500	Abscess of tendon sheath
M6501	Abscess of tendon sheath
M6502	Abscess of tendon sheath
M6503	Abscess of tendon sheath
M6504	Abscess of tendon sheath
M6505	Abscess of tendon sheath
M6506	Abscess of tendon sheath
M6507	Abscess of tendon sheath
M6508	Abscess of tendon sheath
M6509	Abscess of tendon sheath
M651	Other infective (teno)synovitis
M6510	Other infective (teno)synovitis
M6511	Other infective (teno)synovitis
M6512	Other infective (teno)synovitis
M6513	Other infective (teno)synovitis
M6514	Other infective (teno)synovitis
M6515	Other infective (teno)synovitis
M6516	Other infective (teno)synovitis
M6517	Other infective (teno)synovitis
M6518	Other infective (teno)synovitis
M6519	Other infective (teno)synovitis
M652	Calcific tendinitis
M6520	Calcific tendinitis
M6521	Calcific tendinitis

For peer review only

M6522	Calcific tendinitis
M6523	Calcific tendinitis
M6524	Calcific tendinitis
M6525	Calcific tendinitis
M6526	Calcific tendinitis
M6527	Calcific tendinitis
M6528	Calcific tendinitis
M6529	Calcific tendinitis
M653	Trigger finger
M654	Radial styloid tenosynovitis [de Quervain]
M6540	Radial styloid tenosynovitis [de Quervain]
M6543	Radial styloid tenosynovitis [de Quervain]
M6544	Radial styloid tenosynovitis [de Quervain]
M658	Other synovitis and tenosynovitis
M6580	Other synovitis and tenosynovitis
M6581	Other synovitis and tenosynovitis
M6582	Other synovitis and tenosynovitis
M6583	Other synovitis and tenosynovitis
M6584	Other synovitis and tenosynovitis
M6585	Other synovitis and tenosynovitis
M6586	Other synovitis and tenosynovitis
M6587	Other synovitis and tenosynovitis
M6588	Other synovitis and tenosynovitis
M6589	Other synovitis and tenosynovitis
M659	Synovitis and tenosynovitis, unspecified
M6590	Synovitis and tenosynovitis, unspecified
M6591	Synovitis and tenosynovitis, unspecified
M6592	Synovitis and tenosynovitis, unspecified
M6593	Synovitis and tenosynovitis, unspecified
M6594	Synovitis and tenosynovitis, unspecified
M6595	Synovitis and tenosynovitis, unspecified
M6596	Synovitis and tenosynovitis, unspecified
M6597	Synovitis and tenosynovitis, unspecified
M6598	Synovitis and tenosynovitis, unspecified
M6599	Synovitis and tenosynovitis, unspecified
M66	Spontaneous rupture of synovium and tendon
M660	Rupture of popliteal cyst
M661	Rupture of synovium
M6610	Rupture of synovium
M6611	Rupture of synovium
M6612	Rupture of synovium
M6613	Rupture of synovium
M6614	Rupture of synovium
M6615	Rupture of synovium
M6616	Rupture of synovium
M6617	Rupture of synovium
M6618	Rupture of synovium
M6619	Rupture of synovium

For peer review only

M662	Spontaneous rupture of extensor tendons
M6620	Spontaneous rupture of extensor tendons
M6621	Spontaneous rupture of extensor tendons
M6622	Spontaneous rupture of extensor tendons
M6623	Spontaneous rupture of extensor tendons
M6624	Spontaneous rupture of extensor tendons
M6625	Spontaneous rupture of extensor tendons
M6626	Spontaneous rupture of extensor tendons
M6627	Spontaneous rupture of extensor tendons
M6629	Spontaneous rupture of extensor tendons
M663	Spontaneous rupture of flexor tendons
M6630	Spontaneous rupture of flexor tendons
M6631	Spontaneous rupture of flexor tendons
M6632	Spontaneous rupture of flexor tendons
M6633	Spontaneous rupture of flexor tendons
M6634	Spontaneous rupture of flexor tendons
M6635	Spontaneous rupture of flexor tendons
M6636	Spontaneous rupture of flexor tendons
M6637	Spontaneous rupture of flexor tendons
M6638	Spontaneous rupture of flexor tendons
M6639	Spontaneous rupture of flexor tendons
M664	Spontaneous rupture of other tendons
M6640	Spontaneous rupture of other tendons
M6641	Spontaneous rupture of other tendons
M6642	Spontaneous rupture of other tendons
M6643	Spontaneous rupture of other tendons
M6644	Spontaneous rupture of other tendons
M6645	Spontaneous rupture of other tendons
M6646	Spontaneous rupture of other tendons
M6647	Spontaneous rupture of other tendons
M6648	Spontaneous rupture of other tendons
M6649	Spontaneous rupture of other tendons
M665	Spontaneous rupture of unspecified tendon
M6650	Spontaneous rupture of unspecified tendon
M6651	Spontaneous rupture of unspecified tendon
M6652	Spontaneous rupture of unspecified tendon
M6653	Spontaneous rupture of unspecified tendon
M6654	Spontaneous rupture of unspecified tendon
M6655	Spontaneous rupture of unspecified tendon
M6656	Spontaneous rupture of unspecified tendon
M6657	Spontaneous rupture of unspecified tendon
M6658	Spontaneous rupture of unspecified tendon
M6659	Spontaneous rupture of unspecified tendon
M67	Other disorders of synovium and tendon
M670	Short Achilles tendon (acquired)
M671	Other contracture of tendon (sheath)
M672	Synovial hypertrophy, not elsewhere classified
M673	Transient synovitis

M674	Ganglion
M678	Other specified disorders of synovium and tendon
M679	Disorder of synovium and tendon, unspecified
M68	Disorders of synovium and tendon in diseases classified elsewhere
M680	Synovitis and tenosynovitis in bacterial diseases classified elsewhere
M688	Other disorders of synovium and tendon in diseases classified elsewhere
M70	Soft tissue disorders related to use, overuse and pressure
M700	Chronic crepitant synovitis of hand and wrist
M7000	Chronic crepitant synovitis of hand and wrist
M7003	Chronic crepitant synovitis of hand and wrist
M7004	Chronic crepitant synovitis of hand and wrist
M701	Bursitis of hand
M702	Olecranon bursitis
M703	Other bursitis of elbow
M704	Prepatellar bursitis
M705	Other bursitis of knee
M706	Trochanteric bursitis
M707	Other bursitis of hip
M708	Other soft tissue disorders related to use, overuse and pressure
M7080	Other soft tissue disorders related to use, overuse and pressure
M7081	Other soft tissue disorders related to use, overuse and pressure
M7082	Other soft tissue disorders related to use, overuse and pressure
M7083	Other soft tissue disorders related to use, overuse and pressure
M7084	Other soft tissue disorders related to use, overuse and pressure
M7085	Other soft tissue disorders related to use, overuse and pressure
M7086	Other soft tissue disorders related to use, overuse and pressure
M7087	Other soft tissue disorders related to use, overuse and pressure
M7088	Other soft tissue disorders related to use, overuse and pressure
M7089	Other soft tissue disorders related to use, overuse and pressure
M709	Unspecified soft tissue disorder related to use, overuse and pressure
M7090	Unspecified soft tissue disorder related to use, overuse and pressure
M7091	Unspecified soft tissue disorder related to use, overuse and pressure
M7092	Unspecified soft tissue disorder related to use, overuse and pressure
M7093	Unspecified soft tissue disorder related to use, overuse and pressure
M7094	Unspecified soft tissue disorder related to use, overuse and pressure
M7095	Unspecified soft tissue disorder related to use, overuse and pressure
M7096	Unspecified soft tissue disorder related to use, overuse and pressure
M7097	Unspecified soft tissue disorder related to use, overuse and pressure
M7098	Unspecified soft tissue disorder related to use, overuse and pressure
M7099	Unspecified soft tissue disorder related to use, overuse and pressure
M71	Other bursopathies
M710	Abscess of bursa
M7100	Abscess of bursa
M7101	Abscess of bursa
M7102	Abscess of bursa
M7103	Abscess of bursa
M7104	Abscess of bursa
M7105	Abscess of bursa

For peer review only

M7106	Abscess of bursa
M7107	Abscess of bursa
M7108	Abscess of bursa
M7109	Abscess of bursa
M711	Other infective bursitis
M7110	Other infective bursitis
M7111	Other infective bursitis
M7112	Other infective bursitis
M7113	Other infective bursitis
M7114	Other infective bursitis
M7115	Other infective bursitis
M7116	Other infective bursitis
M7117	Other infective bursitis
M7118	Other infective bursitis
M7119	Other infective bursitis
M712	Synovial cyst of popliteal space [Baker]
M713	Other bursal cyst
M7130	Other bursal cyst
M7131	Other bursal cyst
M7132	Other bursal cyst
M7133	Other bursal cyst
M7134	Other bursal cyst
M7135	Other bursal cyst
M7136	Other bursal cyst
M7137	Other bursal cyst
M7138	Other bursal cyst
M7139	Other bursal cyst
M714	Calcium deposit in bursa
M7140	Calcium deposit in bursa
M7141	Calcium deposit in bursa
M7142	Calcium deposit in bursa
M7143	Calcium deposit in bursa
M7144	Calcium deposit in bursa
M7145	Calcium deposit in bursa
M7146	Calcium deposit in bursa
M7147	Calcium deposit in bursa
M7148	Calcium deposit in bursa
M7149	Calcium deposit in bursa
M715	Other bursitis, not elsewhere classified
M7150	Other bursitis, not elsewhere classified
M7151	Other bursitis, not elsewhere classified
M7152	Other bursitis, not elsewhere classified
M7153	Other bursitis, not elsewhere classified
M7154	Other bursitis, not elsewhere classified
M7155	Other bursitis, not elsewhere classified
M7156	Other bursitis, not elsewhere classified
M7157	Other bursitis, not elsewhere classified
M7158	Other bursitis, not elsewhere classified

For peer review only

M7159	Other bursitis, not elsewhere classified
M718	Other specified bursopathies
M7180	Other specified bursopathies
M7181	Other specified bursopathies
M7182	Other specified bursopathies
M7183	Other specified bursopathies
M7184	Other specified bursopathies
M7185	Other specified bursopathies
M7186	Other specified bursopathies
M7187	Other specified bursopathies
M7188	Other specified bursopathies
M7189	Other specified bursopathies
M719	Bursopathy, unspecified
M7190	Bursopathy, unspecified
M7191	Bursopathy, unspecified
M7192	Bursopathy, unspecified
M7193	Bursopathy, unspecified
M7194	Bursopathy, unspecified
M7195	Bursopathy, unspecified
M7196	Bursopathy, unspecified
M7197	Bursopathy, unspecified
M7198	Bursopathy, unspecified
M7199	Bursopathy, unspecified
M72	Fibroblastic disorders
M720	Palmar fascial fibromatosis [Dupuytren]
M721	Knuckle pads
M7210	Knuckle pads
M7214	Knuckle pads
M7217	Knuckle pads
M7219	Knuckle pads
M722	Plantar fascial fibromatosis
M723	Nodular fasciitis
M7230	Nodular fasciitis
M7231	Nodular fasciitis
M7232	Nodular fasciitis
M7233	Nodular fasciitis
M7234	Nodular fasciitis
M7235	Nodular fasciitis
M7236	Nodular fasciitis
M7237	Nodular fasciitis
M7238	Nodular fasciitis
M724	Pseudosarcomatous fibromatosis
M7240	Pseudosarcomatous fibromatosis
M7241	Pseudosarcomatous fibromatosis
M7242	Pseudosarcomatous fibromatosis
M7243	Pseudosarcomatous fibromatosis
M7244	Pseudosarcomatous fibromatosis
M7245	Pseudosarcomatous fibromatosis

For peer review only

M7246	Pseudosarcomatous fibromatosis
M7247	Pseudosarcomatous fibromatosis
M7248	Pseudosarcomatous fibromatosis
M7249	Pseudosarcomatous fibromatosis
M725	Fasciitis, not elsewhere classified
M7250	Fasciitis, not elsewhere classified
M7251	Fasciitis, not elsewhere classified
M7252	Fasciitis, not elsewhere classified
M7253	Fasciitis, not elsewhere classified
M7254	Fasciitis, not elsewhere classified
M7255	Fasciitis, not elsewhere classified
M7256	Fasciitis, not elsewhere classified
M7257	Fasciitis, not elsewhere classified
M7258	Fasciitis, not elsewhere classified
M7259	Fasciitis, not elsewhere classified
M726	Necrotizing fasciitis
M7260	Necrotizing fasciitis
M7261	Necrotizing fasciitis
M7262	Necrotizing fasciitis
M7263	Necrotizing fasciitis
M7264	Necrotizing fasciitis
M7265	Necrotizing fasciitis
M7266	Necrotizing fasciitis
M7267	Necrotizing fasciitis
M7268	Necrotizing fasciitis
M7269	Necrotizing fasciitis
M728	Other fibroblastic disorders
M7280	Other fibroblastic disorders
M7281	Other fibroblastic disorders
M7282	Other fibroblastic disorders
M7283	Other fibroblastic disorders
M7284	Other fibroblastic disorders
M7285	Other fibroblastic disorders
M7286	Other fibroblastic disorders
M7287	Other fibroblastic disorders
M7288	Other fibroblastic disorders
M7289	Other fibroblastic disorders
M729	Fibroblastic disorder, unspecified
M7290	Fibroblastic disorder, unspecified
M7291	Fibroblastic disorder, unspecified
M7292	Fibroblastic disorder, unspecified
M7293	Fibroblastic disorder, unspecified
M7294	Fibroblastic disorder, unspecified
M7295	Fibroblastic disorder, unspecified
M7296	Fibroblastic disorder, unspecified
M7297	Fibroblastic disorder, unspecified
M7298	Fibroblastic disorder, unspecified
M7299	Fibroblastic disorder, unspecified

For peer review only

M73	Soft tissue disorders in diseases classified elsewhere
M730	Gonococcal bursitis
M7300	Gonococcal bursitis
M7301	Gonococcal bursitis
M7302	Gonococcal bursitis
M7303	Gonococcal bursitis
M7304	Gonococcal bursitis
M7305	Gonococcal bursitis
M7306	Gonococcal bursitis
M7307	Gonococcal bursitis
M7308	Gonococcal bursitis
M7309	Gonococcal bursitis
M731	Syphilitic bursitis
M7310	Syphilitic bursitis
M7311	Syphilitic bursitis
M7312	Syphilitic bursitis
M7313	Syphilitic bursitis
M7314	Syphilitic bursitis
M7315	Syphilitic bursitis
M7316	Syphilitic bursitis
M7317	Syphilitic bursitis
M7318	Syphilitic bursitis
M7319	Syphilitic bursitis
M738	Other soft tissue disorders in diseases classified elsewhere
M7380	Other soft tissue disorders in diseases classified elsewhere
M7381	Other soft tissue disorders in diseases classified elsewhere
M7382	Other soft tissue disorders in diseases classified elsewhere
M7383	Other soft tissue disorders in diseases classified elsewhere
M7384	Other soft tissue disorders in diseases classified elsewhere
M7385	Other soft tissue disorders in diseases classified elsewhere
M7386	Other soft tissue disorders in diseases classified elsewhere
M7387	Other soft tissue disorders in diseases classified elsewhere
M7388	Other soft tissue disorders in diseases classified elsewhere
M7389	Other soft tissue disorders in diseases classified elsewhere
M75	Shoulder lesions
M750	Adhesive capsulitis of shoulder
M751	Rotator cuff syndrome
M752	Bicipital tendinitis
M753	Calcific tendinitis of shoulder
M754	Impingement syndrome of shoulder
M755	Bursitis of shoulder
M758	Other shoulder lesions
M759	Shoulder lesion, unspecified
M76	Enthesopathies of lower limb, excluding foot
M760	Gluteal tendinitis
M7600	Gluteal tendinitis
M7605	Gluteal tendinitis
M761	Psoas tendinitis

For peer review only

M7610	Psoas tendinitis
M7615	Psoas tendinitis
M762	Iliac crest spur
M763	Iliotibial band syndrome
M7630	Iliotibial band syndrome
M7635	Iliotibial band syndrome
M7636	Iliotibial band syndrome
M764	Tibial collateral bursitis [Pellegrini-Stieda]
M765	Patellar tendinitis
M766	Achilles tendinitis
M7660	Achilles tendinitis
M7666	Achilles tendinitis
M7667	Achilles tendinitis
M767	Peroneal tendinitis
M7670	Peroneal tendinitis
M7676	Peroneal tendinitis
M7677	Peroneal tendinitis
M768	Other enthesopathies of lower limb, excluding foot
M7680	Other enthesopathies of lower limb, excluding foot
M7685	Other enthesopathies of lower limb, excluding foot
M7686	Other enthesopathies of lower limb, excluding foot
M7687	Other enthesopathies of lower limb, excluding foot
M769	Enthesopathy of lower limb, unspecified
M7690	Enthesopathy of lower limb, unspecified
M7695	Enthesopathy of lower limb, unspecified
M7696	Enthesopathy of lower limb, unspecified
M7697	Enthesopathy of lower limb, unspecified
M7698	Enthesopathy of lower limb, unspecified
M7699	Enthesopathy of lower limb, unspecified
M77	Other enthesopathies
M770	Medial epicondylitis
M7700	Medial epicondylitis
M7702	Medial epicondylitis
M7705	Medial epicondylitis
M7706	Medial epicondylitis
M7709	Medial epicondylitis
M771	Lateral epicondylitis
M7710	Lateral epicondylitis
M7712	Lateral epicondylitis
M7715	Lateral epicondylitis
M7716	Lateral epicondylitis
M7719	Lateral epicondylitis
M772	Periarthritis of wrist
M773	Calcaneal spur
M774	Metatarsalgia
M775	Other enthesopathy of foot
M778	Other enthesopathies, not elsewhere classified
M7780	Other enthesopathies, not elsewhere classified

For peer review only

M7781	Other enthesopathies, not elsewhere classified
M7782	Other enthesopathies, not elsewhere classified
M7783	Other enthesopathies, not elsewhere classified
M7784	Other enthesopathies, not elsewhere classified
M7785	Other enthesopathies, not elsewhere classified
M7786	Other enthesopathies, not elsewhere classified
M7787	Other enthesopathies, not elsewhere classified
M7788	Other enthesopathies, not elsewhere classified
M7789	Other enthesopathies, not elsewhere classified
M779	Enthesopathy, unspecified
M7790	Enthesopathy, unspecified
M7791	Enthesopathy, unspecified
M7792	Enthesopathy, unspecified
M7793	Enthesopathy, unspecified
M7794	Enthesopathy, unspecified
M7795	Enthesopathy, unspecified
M7796	Enthesopathy, unspecified
M7797	Enthesopathy, unspecified
M7798	Enthesopathy, unspecified
M7799	Enthesopathy, unspecified
M79	Other soft tissue disorders, not elsewhere classified
M790	Rheumatism, unspecified
M7900	Rheumatism, unspecified
M7901	Rheumatism, unspecified
M7902	Rheumatism, unspecified
M7903	Rheumatism, unspecified
M7904	Rheumatism, unspecified
M7905	Rheumatism, unspecified
M7906	Rheumatism, unspecified
M7907	Rheumatism, unspecified
M7908	Rheumatism, unspecified
M7909	Rheumatism, unspecified
M791	Myalgia
M7910	Myalgia
M7911	Myalgia
M7912	Myalgia
M7913	Myalgia
M7914	Myalgia
M7915	Myalgia
M7916	Myalgia
M7917	Myalgia
M7918	Myalgia
M7919	Myalgia
M792	Neuralgia and neuritis, unspecified
M7920	Neuralgia and neuritis, unspecified
M7921	Neuralgia and neuritis, unspecified
M7922	Neuralgia and neuritis, unspecified
M7923	Neuralgia and neuritis, unspecified

For peer review only

M7924	Neuralgia and neuritis, unspecified
M7925	Neuralgia and neuritis, unspecified
M7926	Neuralgia and neuritis, unspecified
M7927	Neuralgia and neuritis, unspecified
M7928	Neuralgia and neuritis, unspecified
M7929	Neuralgia and neuritis, unspecified
M793	Panniculitis, unspecified
M7930	Panniculitis, unspecified
M7931	Panniculitis, unspecified
M7932	Panniculitis, unspecified
M7933	Panniculitis, unspecified
M7934	Panniculitis, unspecified
M7935	Panniculitis, unspecified
M7936	Panniculitis, unspecified
M7937	Panniculitis, unspecified
M7938	Panniculitis, unspecified
M7939	Panniculitis, unspecified
M794	Hypertrophy of (infrapatellar) fat pad
M7940	Hypertrophy of (infrapatellar) fat pad
M7941	Hypertrophy of (infrapatellar) fat pad
M7942	Hypertrophy of (infrapatellar) fat pad
M7943	Hypertrophy of (infrapatellar) fat pad
M7944	Hypertrophy of (infrapatellar) fat pad
M7945	Hypertrophy of (infrapatellar) fat pad
M7946	Hypertrophy of (infrapatellar) fat pad
M7947	Hypertrophy of (infrapatellar) fat pad
M7948	Hypertrophy of (infrapatellar) fat pad
M795	Residual foreign body in soft tissue
M7950	Residual foreign body in soft tissue
M7951	Residual foreign body in soft tissue
M7952	Residual foreign body in soft tissue
M7953	Residual foreign body in soft tissue
M7954	Residual foreign body in soft tissue
M7955	Residual foreign body in soft tissue
M7956	Residual foreign body in soft tissue
M7957	Residual foreign body in soft tissue
M7958	Residual foreign body in soft tissue
M7959	Residual foreign body in soft tissue
M796	Pain in limb
M7960	Pain in limb
M7961	Pain in limb
M7962	Pain in limb
M7963	Pain in limb
M7964	Pain in limb
M7965	Pain in limb
M7966	Pain in limb
M7967	Pain in limb
M7968	Pain in limb

For peer review only

M7969	Pain in limb
M797	Fibromyalgia
M7970	Fibromyalgia
M7971	Fibromyalgia
M7972	Fibromyalgia
M7973	Fibromyalgia
M7974	Fibromyalgia
M7975	Fibromyalgia
M7976	Fibromyalgia
M7977	Fibromyalgia
M7978	Fibromyalgia
M7979	Fibromyalgia
M798	Other specified soft tissue disorders
M7980	Other specified soft tissue disorders
M7981	Other specified soft tissue disorders
M7982	Other specified soft tissue disorders
M7983	Other specified soft tissue disorders
M7984	Other specified soft tissue disorders
M7985	Other specified soft tissue disorders
M7986	Other specified soft tissue disorders
M7987	Other specified soft tissue disorders
M7988	Other specified soft tissue disorders
M7989	Other specified soft tissue disorders
M799	Soft tissue disorder, unspecified
M7990	Soft tissue disorder, unspecified
M7991	Soft tissue disorder, unspecified
M7992	Soft tissue disorder, unspecified
M7993	Soft tissue disorder, unspecified
M7994	Soft tissue disorder, unspecified
M7995	Soft tissue disorder, unspecified
M7996	Soft tissue disorder, unspecified
M7997	Soft tissue disorder, unspecified
M7998	Soft tissue disorder, unspecified
M7999	Soft tissue disorder, unspecified
M80	Osteoporosis with pathological fracture
M800	Postmenopausal osteoporosis with pathological fracture
M8000	Postmenopausal osteoporosis with pathological fracture
M8001	Postmenopausal osteoporosis with pathological fracture
M8002	Postmenopausal osteoporosis with pathological fracture
M8003	Postmenopausal osteoporosis with pathological fracture
M8004	Postmenopausal osteoporosis with pathological fracture
M8005	Postmenopausal osteoporosis with pathological fracture
M8006	Postmenopausal osteoporosis with pathological fracture
M8007	Postmenopausal osteoporosis with pathological fracture
M8008	Postmenopausal osteoporosis with pathological fracture
M8009	Postmenopausal osteoporosis with pathological fracture
M801	Postoophorectomy osteoporosis with pathological fracture
M8010	Postoophorectomy osteoporosis with pathological fracture

For peer review only

M8011	Postoophorectomy osteoporosis with pathological fracture
M8012	Postoophorectomy osteoporosis with pathological fracture
M8013	Postoophorectomy osteoporosis with pathological fracture
M8014	Postoophorectomy osteoporosis with pathological fracture
M8015	Postoophorectomy osteoporosis with pathological fracture
M8016	Postoophorectomy osteoporosis with pathological fracture
M8017	Postoophorectomy osteoporosis with pathological fracture
M8018	Postoophorectomy osteoporosis with pathological fracture
M8019	Postoophorectomy osteoporosis with pathological fracture
M802	Osteoporosis of disuse with pathological fracture
M8020	Osteoporosis of disuse with pathological fracture
M8021	Osteoporosis of disuse with pathological fracture
M8022	Osteoporosis of disuse with pathological fracture
M8023	Osteoporosis of disuse with pathological fracture
M8024	Osteoporosis of disuse with pathological fracture
M8025	Osteoporosis of disuse with pathological fracture
M8026	Osteoporosis of disuse with pathological fracture
M8027	Osteoporosis of disuse with pathological fracture
M8028	Osteoporosis of disuse with pathological fracture
M8029	Osteoporosis of disuse with pathological fracture
M803	Postsurgical malabsorption osteoporosis with pathological fracture
M8030	Postsurgical malabsorption osteoporosis with pathological fracture
M8031	Postsurgical malabsorption osteoporosis with pathological fracture
M8032	Postsurgical malabsorption osteoporosis with pathological fracture
M8033	Postsurgical malabsorption osteoporosis with pathological fracture
M8034	Postsurgical malabsorption osteoporosis with pathological fracture
M8035	Postsurgical malabsorption osteoporosis with pathological fracture
M8036	Postsurgical malabsorption osteoporosis with pathological fracture
M8037	Postsurgical malabsorption osteoporosis with pathological fracture
M8038	Postsurgical malabsorption osteoporosis with pathological fracture
M8039	Postsurgical malabsorption osteoporosis with pathological fracture
M804	Drug-induced osteoporosis with pathological fracture
M8040	Drug-induced osteoporosis with pathological fracture
M8041	Drug-induced osteoporosis with pathological fracture
M8042	Drug-induced osteoporosis with pathological fracture
M8043	Drug-induced osteoporosis with pathological fracture
M8044	Drug-induced osteoporosis with pathological fracture
M8045	Drug-induced osteoporosis with pathological fracture
M8046	Drug-induced osteoporosis with pathological fracture
M8047	Drug-induced osteoporosis with pathological fracture
M8048	Drug-induced osteoporosis with pathological fracture
M8049	Drug-induced osteoporosis with pathological fracture
M805	Idiopathic osteoporosis with pathological fracture
M8050	Idiopathic osteoporosis with pathological fracture
M8051	Idiopathic osteoporosis with pathological fracture
M8052	Idiopathic osteoporosis with pathological fracture
M8053	Idiopathic osteoporosis with pathological fracture
M8054	Idiopathic osteoporosis with pathological fracture

For peer review only

M8055	Idiopathic osteoporosis with pathological fracture
M8056	Idiopathic osteoporosis with pathological fracture
M8057	Idiopathic osteoporosis with pathological fracture
M8058	Idiopathic osteoporosis with pathological fracture
M8059	Idiopathic osteoporosis with pathological fracture
M808	Other osteoporosis with pathological fracture
M8080	Other osteoporosis with pathological fracture
M8081	Other osteoporosis with pathological fracture
M8082	Other osteoporosis with pathological fracture
M8083	Other osteoporosis with pathological fracture
M8084	Other osteoporosis with pathological fracture
M8085	Other osteoporosis with pathological fracture
M8086	Other osteoporosis with pathological fracture
M8087	Other osteoporosis with pathological fracture
M8088	Other osteoporosis with pathological fracture
M8089	Other osteoporosis with pathological fracture
M809	Unspecified osteoporosis with pathological fracture
M8090	Unspecified osteoporosis with pathological fracture
M8091	Unspecified osteoporosis with pathological fracture
M8092	Unspecified osteoporosis with pathological fracture
M8093	Unspecified osteoporosis with pathological fracture
M8094	Unspecified osteoporosis with pathological fracture
M8095	Unspecified osteoporosis with pathological fracture
M8096	Unspecified osteoporosis with pathological fracture
M8097	Unspecified osteoporosis with pathological fracture
M8098	Unspecified osteoporosis with pathological fracture
M8099	Unspecified osteoporosis with pathological fracture
M81	Osteoporosis without pathological fracture
M810	Postmenopausal osteoporosis
M8100	Postmenopausal osteoporosis
M8101	Postmenopausal osteoporosis
M8102	Postmenopausal osteoporosis
M8103	Postmenopausal osteoporosis
M8104	Postmenopausal osteoporosis
M8105	Postmenopausal osteoporosis
M8106	Postmenopausal osteoporosis
M8107	Postmenopausal osteoporosis
M8108	Postmenopausal osteoporosis
M8109	Postmenopausal osteoporosis
M811	Postoophorectomy osteoporosis
M8110	Postoophorectomy osteoporosis
M8111	Postoophorectomy osteoporosis
M8112	Postoophorectomy osteoporosis
M8113	Postoophorectomy osteoporosis
M8114	Postoophorectomy osteoporosis
M8115	Postoophorectomy osteoporosis
M8116	Postoophorectomy osteoporosis
M8117	Postoophorectomy osteoporosis

For peer review only

M8118	Postoophorectomy osteoporosis
M8119	Postoophorectomy osteoporosis
M812	Osteoporosis of disuse
M8120	Osteoporosis of disuse
M8121	Osteoporosis of disuse
M8122	Osteoporosis of disuse
M8123	Osteoporosis of disuse
M8124	Osteoporosis of disuse
M8125	Osteoporosis of disuse
M8126	Osteoporosis of disuse
M8127	Osteoporosis of disuse
M8128	Osteoporosis of disuse
M8129	Osteoporosis of disuse
M813	Postsurgical malabsorption osteoporosis
M8130	Postsurgical malabsorption osteoporosis
M8131	Postsurgical malabsorption osteoporosis
M8132	Postsurgical malabsorption osteoporosis
M8133	Postsurgical malabsorption osteoporosis
M8134	Postsurgical malabsorption osteoporosis
M8135	Postsurgical malabsorption osteoporosis
M8136	Postsurgical malabsorption osteoporosis
M8137	Postsurgical malabsorption osteoporosis
M8138	Postsurgical malabsorption osteoporosis
M8139	Postsurgical malabsorption osteoporosis
M814	Drug-induced osteoporosis
M8140	Drug-induced osteoporosis
M8141	Drug-induced osteoporosis
M8142	Drug-induced osteoporosis
M8143	Drug-induced osteoporosis
M8144	Drug-induced osteoporosis
M8145	Drug-induced osteoporosis
M8146	Drug-induced osteoporosis
M8147	Drug-induced osteoporosis
M8148	Drug-induced osteoporosis
M8149	Drug-induced osteoporosis
M815	Idiopathic osteoporosis
M8150	Idiopathic osteoporosis
M8151	Idiopathic osteoporosis
M8152	Idiopathic osteoporosis
M8153	Idiopathic osteoporosis
M8154	Idiopathic osteoporosis
M8155	Idiopathic osteoporosis
M8156	Idiopathic osteoporosis
M8157	Idiopathic osteoporosis
M8158	Idiopathic osteoporosis
M8159	Idiopathic osteoporosis
M816	Localized osteoporosis [Lequesne]
M8160	Localized osteoporosis [Lequesne]

For peer review only

M8161	Localized osteoporosis [Lequesne]
M8162	Localized osteoporosis [Lequesne]
M8163	Localized osteoporosis [Lequesne]
M8164	Localized osteoporosis [Lequesne]
M8165	Localized osteoporosis [Lequesne]
M8166	Localized osteoporosis [Lequesne]
M8167	Localized osteoporosis [Lequesne]
M8168	Localized osteoporosis [Lequesne]
M8169	Localized osteoporosis [Lequesne]
M818	Other osteoporosis
M8180	Other osteoporosis
M8181	Other osteoporosis
M8182	Other osteoporosis
M8183	Other osteoporosis
M8184	Other osteoporosis
M8185	Other osteoporosis
M8186	Other osteoporosis
M8187	Other osteoporosis
M8188	Other osteoporosis
M8189	Other osteoporosis
M819	Osteoporosis, unspecified
M8190	Osteoporosis, unspecified
M8191	Osteoporosis, unspecified
M8192	Osteoporosis, unspecified
M8193	Osteoporosis, unspecified
M8194	Osteoporosis, unspecified
M8195	Osteoporosis, unspecified
M8196	Osteoporosis, unspecified
M8197	Osteoporosis, unspecified
M8198	Osteoporosis, unspecified
M8199	Osteoporosis, unspecified
M82	Osteoporosis in diseases classified elsewhere
M820	Osteoporosis in multiple myelomatosis
M8200	Osteoporosis in multiple myelomatosis
M8201	Osteoporosis in multiple myelomatosis
M8202	Osteoporosis in multiple myelomatosis
M8203	Osteoporosis in multiple myelomatosis
M8204	Osteoporosis in multiple myelomatosis
M8205	Osteoporosis in multiple myelomatosis
M8206	Osteoporosis in multiple myelomatosis
M8207	Osteoporosis in multiple myelomatosis
M8208	Osteoporosis in multiple myelomatosis
M8209	Osteoporosis in multiple myelomatosis
M821	Osteoporosis in endocrine disorders
M8210	Osteoporosis in endocrine disorders
M8211	Osteoporosis in endocrine disorders
M8212	Osteoporosis in endocrine disorders
M8213	Osteoporosis in endocrine disorders

For peer review only

M8214	Osteoporosis in endocrine disorders
M8215	Osteoporosis in endocrine disorders
M8216	Osteoporosis in endocrine disorders
M8217	Osteoporosis in endocrine disorders
M8218	Osteoporosis in endocrine disorders
M8219	Osteoporosis in endocrine disorders
M828	Osteoporosis in other diseases classified elsewhere
M8280	Osteoporosis in other diseases classified elsewhere
M8281	Osteoporosis in other diseases classified elsewhere
M8282	Osteoporosis in other diseases classified elsewhere
M8283	Osteoporosis in other diseases classified elsewhere
M8284	Osteoporosis in other diseases classified elsewhere
M8285	Osteoporosis in other diseases classified elsewhere
M8286	Osteoporosis in other diseases classified elsewhere
M8287	Osteoporosis in other diseases classified elsewhere
M8288	Osteoporosis in other diseases classified elsewhere
M8289	Osteoporosis in other diseases classified elsewhere
M83	Adult osteomalacia
M830	Puerperal osteomalacia
M8300	Puerperal osteomalacia
M8301	Puerperal osteomalacia
M8302	Puerperal osteomalacia
M8303	Puerperal osteomalacia
M8304	Puerperal osteomalacia
M8305	Puerperal osteomalacia
M8306	Puerperal osteomalacia
M8307	Puerperal osteomalacia
M8308	Puerperal osteomalacia
M8309	Puerperal osteomalacia
M831	Senile osteomalacia
M8310	Senile osteomalacia
M8311	Senile osteomalacia
M8312	Senile osteomalacia
M8313	Senile osteomalacia
M8314	Senile osteomalacia
M8315	Senile osteomalacia
M8316	Senile osteomalacia
M8317	Senile osteomalacia
M8318	Senile osteomalacia
M8319	Senile osteomalacia
M832	Adult osteomalacia due to malabsorption
M8320	Adult osteomalacia due to malabsorption
M8321	Adult osteomalacia due to malabsorption
M8322	Adult osteomalacia due to malabsorption
M8323	Adult osteomalacia due to malabsorption
M8324	Adult osteomalacia due to malabsorption
M8325	Adult osteomalacia due to malabsorption
M8326	Adult osteomalacia due to malabsorption

For peer review only

M8327	Adult osteomalacia due to malabsorption
M8328	Adult osteomalacia due to malabsorption
M8329	Adult osteomalacia due to malabsorption
M833	Adult osteomalacia due to malnutrition
M8330	Adult osteomalacia due to malnutrition
M8331	Adult osteomalacia due to malnutrition
M8332	Adult osteomalacia due to malnutrition
M8333	Adult osteomalacia due to malnutrition
M8334	Adult osteomalacia due to malnutrition
M8335	Adult osteomalacia due to malnutrition
M8336	Adult osteomalacia due to malnutrition
M8337	Adult osteomalacia due to malnutrition
M8338	Adult osteomalacia due to malnutrition
M8339	Adult osteomalacia due to malnutrition
M834	Aluminium bone disease
M8340	Aluminium bone disease
M8341	Aluminium bone disease
M8342	Aluminium bone disease
M8343	Aluminium bone disease
M8344	Aluminium bone disease
M8345	Aluminium bone disease
M8346	Aluminium bone disease
M8347	Aluminium bone disease
M8348	Aluminium bone disease
M8349	Aluminium bone disease
M835	Other drug-induced osteomalacia in adults
M8350	Other drug-induced osteomalacia in adults
M8351	Other drug-induced osteomalacia in adults
M8352	Other drug-induced osteomalacia in adults
M8353	Other drug-induced osteomalacia in adults
M8354	Other drug-induced osteomalacia in adults
M8355	Other drug-induced osteomalacia in adults
M8356	Other drug-induced osteomalacia in adults
M8357	Other drug-induced osteomalacia in adults
M8358	Other drug-induced osteomalacia in adults
M8359	Other drug-induced osteomalacia in adults
M838	Other adult osteomalacia
M8380	Other adult osteomalacia
M8381	Other adult osteomalacia
M8382	Other adult osteomalacia
M8383	Other adult osteomalacia
M8384	Other adult osteomalacia
M8385	Other adult osteomalacia
M8386	Other adult osteomalacia
M8387	Other adult osteomalacia
M8388	Other adult osteomalacia
M8389	Other adult osteomalacia
M839	Adult osteomalacia, unspecified

For peer review only

M8390	Adult osteomalacia, unspecified
M8391	Adult osteomalacia, unspecified
M8392	Adult osteomalacia, unspecified
M8393	Adult osteomalacia, unspecified
M8394	Adult osteomalacia, unspecified
M8395	Adult osteomalacia, unspecified
M8396	Adult osteomalacia, unspecified
M8397	Adult osteomalacia, unspecified
M8398	Adult osteomalacia, unspecified
M8399	Adult osteomalacia, unspecified
M84	Disorders of continuity of bone
M840	Malunion of fracture
M8400	Malunion of fracture
M8401	Malunion of fracture
M8402	Malunion of fracture
M8403	Malunion of fracture
M8404	Malunion of fracture
M8405	Malunion of fracture
M8406	Malunion of fracture
M8407	Malunion of fracture
M8408	Malunion of fracture
M8409	Malunion of fracture
M841	Nonunion of fracture [pseudarthrosis]
M8410	Nonunion of fracture [pseudarthrosis]
M8411	Nonunion of fracture [pseudarthrosis]
M8412	Nonunion of fracture [pseudarthrosis]
M8413	Nonunion of fracture [pseudarthrosis]
M8414	Nonunion of fracture [pseudarthrosis]
M8415	Nonunion of fracture [pseudarthrosis]
M8416	Nonunion of fracture [pseudarthrosis]
M8417	Nonunion of fracture [pseudarthrosis]
M8418	Nonunion of fracture [pseudarthrosis]
M8419	Nonunion of fracture [pseudarthrosis]
M842	Delayed union of fracture
M8420	Delayed union of fracture
M8421	Delayed union of fracture
M8422	Delayed union of fracture
M8423	Delayed union of fracture
M8424	Delayed union of fracture
M8425	Delayed union of fracture
M8426	Delayed union of fracture
M8427	Delayed union of fracture
M8428	Delayed union of fracture
M8429	Delayed union of fracture
M843	Stress fracture, not elsewhere classified
M8430	Stress fracture, not elsewhere classified
M8431	Stress fracture, not elsewhere classified
M8432	Stress fracture, not elsewhere classified

For peer review only

M8433	Stress fracture, not elsewhere classified
M8434	Stress fracture, not elsewhere classified
M8435	Stress fracture, not elsewhere classified
M8436	Stress fracture, not elsewhere classified
M8437	Stress fracture, not elsewhere classified
M8438	Stress fracture, not elsewhere classified
M8439	Stress fracture, not elsewhere classified
M844	Pathological fracture, not elsewhere classified
M8440	Pathological fracture, not elsewhere classified
M8441	Pathological fracture, not elsewhere classified
M8442	Pathological fracture, not elsewhere classified
M8443	Pathological fracture, not elsewhere classified
M8444	Pathological fracture, not elsewhere classified
M8445	Pathological fracture, not elsewhere classified
M8446	Pathological fracture, not elsewhere classified
M8447	Pathological fracture, not elsewhere classified
M8448	Pathological fracture, not elsewhere classified
M8449	Pathological fracture, not elsewhere classified
M848	Other disorders of continuity of bone
M8480	Other disorders of continuity of bone
M8481	Other disorders of continuity of bone
M8482	Other disorders of continuity of bone
M8483	Other disorders of continuity of bone
M8484	Other disorders of continuity of bone
M8485	Other disorders of continuity of bone
M8486	Other disorders of continuity of bone
M8487	Other disorders of continuity of bone
M8488	Other disorders of continuity of bone
M8489	Other disorders of continuity of bone
M849	Disorder of continuity of bone, unspecified
M8490	Disorder of continuity of bone, unspecified
M8491	Disorder of continuity of bone, unspecified
M8492	Disorder of continuity of bone, unspecified
M8493	Disorder of continuity of bone, unspecified
M8494	Disorder of continuity of bone, unspecified
M8495	Disorder of continuity of bone, unspecified
M8496	Disorder of continuity of bone, unspecified
M8497	Disorder of continuity of bone, unspecified
M8498	Disorder of continuity of bone, unspecified
M8499	Disorder of continuity of bone, unspecified
M85	Other disorders of bone density and structure
M850	Fibrous dysplasia (monostotic)
M8500	Fibrous dysplasia (monostotic)
M8501	Fibrous dysplasia (monostotic)
M8502	Fibrous dysplasia (monostotic)
M8503	Fibrous dysplasia (monostotic)
M8504	Fibrous dysplasia (monostotic)
M8505	Fibrous dysplasia (monostotic)

For peer review only

M8506	Fibrous dysplasia (monostotic)
M8507	Fibrous dysplasia (monostotic)
M8508	Fibrous dysplasia (monostotic)
M8509	Fibrous dysplasia (monostotic)
M851	Skeletal fluorosis
M8510	Skeletal fluorosis
M8511	Skeletal fluorosis
M8512	Skeletal fluorosis
M8513	Skeletal fluorosis
M8514	Skeletal fluorosis
M8515	Skeletal fluorosis
M8516	Skeletal fluorosis
M8517	Skeletal fluorosis
M8518	Skeletal fluorosis
M8519	Skeletal fluorosis
M852	Hyperostosis of skull
M853	Osteitis condensans
M8530	Osteitis condensans
M8531	Osteitis condensans
M8532	Osteitis condensans
M8533	Osteitis condensans
M8534	Osteitis condensans
M8535	Osteitis condensans
M8536	Osteitis condensans
M8537	Osteitis condensans
M8538	Osteitis condensans
M8539	Osteitis condensans
M854	Solitary bone cyst
M8540	Solitary bone cyst
M8541	Solitary bone cyst
M8542	Solitary bone cyst
M8543	Solitary bone cyst
M8544	Solitary bone cyst
M8545	Solitary bone cyst
M8546	Solitary bone cyst
M8547	Solitary bone cyst
M8548	Solitary bone cyst
M8549	Solitary bone cyst
M855	Aneurysmal bone cyst
M8550	Aneurysmal bone cyst
M8551	Aneurysmal bone cyst
M8552	Aneurysmal bone cyst
M8553	Aneurysmal bone cyst
M8554	Aneurysmal bone cyst
M8555	Aneurysmal bone cyst
M8556	Aneurysmal bone cyst
M8557	Aneurysmal bone cyst
M8558	Aneurysmal bone cyst

For peer review only

M8559	Aneurysmal bone cyst
M856	Other cyst of bone
M8560	Other cyst of bone
M8561	Other cyst of bone
M8562	Other cyst of bone
M8563	Other cyst of bone
M8564	Other cyst of bone
M8565	Other cyst of bone
M8566	Other cyst of bone
M8567	Other cyst of bone
M8568	Other cyst of bone
M8569	Other cyst of bone
M858	Other specified disorders of bone density and structure
M8580	Other specified disorders of bone density and structure
M8581	Other specified disorders of bone density and structure
M8582	Other specified disorders of bone density and structure
M8583	Other specified disorders of bone density and structure
M8584	Other specified disorders of bone density and structure
M8585	Other specified disorders of bone density and structure
M8586	Other specified disorders of bone density and structure
M8587	Other specified disorders of bone density and structure
M8588	Other specified disorders of bone density and structure
M8589	Other specified disorders of bone density and structure
M859	Disorder of bone density and structure, unspecified
M8590	Disorder of bone density and structure, unspecified
M8591	Disorder of bone density and structure, unspecified
M8592	Disorder of bone density and structure, unspecified
M8593	Disorder of bone density and structure, unspecified
M8594	Disorder of bone density and structure, unspecified
M8595	Disorder of bone density and structure, unspecified
M8596	Disorder of bone density and structure, unspecified
M8597	Disorder of bone density and structure, unspecified
M8598	Disorder of bone density and structure, unspecified
M8599	Disorder of bone density and structure, unspecified
M86	Osteomyelitis
M860	Acute haematogenous osteomyelitis
M8600	Acute haematogenous osteomyelitis
M8601	Acute haematogenous osteomyelitis
M8602	Acute haematogenous osteomyelitis
M8603	Acute haematogenous osteomyelitis
M8604	Acute haematogenous osteomyelitis
M8605	Acute haematogenous osteomyelitis
M8606	Acute haematogenous osteomyelitis
M8607	Acute haematogenous osteomyelitis
M8608	Acute haematogenous osteomyelitis
M8609	Acute haematogenous osteomyelitis
M861	Other acute osteomyelitis
M8610	Other acute osteomyelitis

For peer review only

M8611	Other acute osteomyelitis
M8612	Other acute osteomyelitis
M8613	Other acute osteomyelitis
M8614	Other acute osteomyelitis
M8615	Other acute osteomyelitis
M8616	Other acute osteomyelitis
M8617	Other acute osteomyelitis
M8618	Other acute osteomyelitis
M8619	Other acute osteomyelitis
M862	Subacute osteomyelitis
M8620	Subacute osteomyelitis
M8621	Subacute osteomyelitis
M8622	Subacute osteomyelitis
M8623	Subacute osteomyelitis
M8624	Subacute osteomyelitis
M8625	Subacute osteomyelitis
M8626	Subacute osteomyelitis
M8627	Subacute osteomyelitis
M8628	Subacute osteomyelitis
M8629	Subacute osteomyelitis
M863	Chronic multifocal osteomyelitis
M8630	Chronic multifocal osteomyelitis
M8631	Chronic multifocal osteomyelitis
M8632	Chronic multifocal osteomyelitis
M8633	Chronic multifocal osteomyelitis
M8634	Chronic multifocal osteomyelitis
M8635	Chronic multifocal osteomyelitis
M8636	Chronic multifocal osteomyelitis
M8637	Chronic multifocal osteomyelitis
M8638	Chronic multifocal osteomyelitis
M8639	Chronic multifocal osteomyelitis
M864	Chronic osteomyelitis with draining sinus
M8640	Chronic osteomyelitis with draining sinus
M8641	Chronic osteomyelitis with draining sinus
M8642	Chronic osteomyelitis with draining sinus
M8643	Chronic osteomyelitis with draining sinus
M8644	Chronic osteomyelitis with draining sinus
M8645	Chronic osteomyelitis with draining sinus
M8646	Chronic osteomyelitis with draining sinus
M8647	Chronic osteomyelitis with draining sinus
M8648	Chronic osteomyelitis with draining sinus
M8649	Chronic osteomyelitis with draining sinus
M865	Other chronic haematogenous osteomyelitis
M8650	Other chronic haematogenous osteomyelitis
M8651	Other chronic haematogenous osteomyelitis
M8652	Other chronic haematogenous osteomyelitis
M8653	Other chronic haematogenous osteomyelitis
M8654	Other chronic haematogenous osteomyelitis

For peer review only

M8655	Other chronic haematogenous osteomyelitis
M8656	Other chronic haematogenous osteomyelitis
M8657	Other chronic haematogenous osteomyelitis
M8658	Other chronic haematogenous osteomyelitis
M8659	Other chronic haematogenous osteomyelitis
M866	Other chronic osteomyelitis
M8660	Other chronic osteomyelitis
M8661	Other chronic osteomyelitis
M8662	Other chronic osteomyelitis
M8663	Other chronic osteomyelitis
M8664	Other chronic osteomyelitis
M8665	Other chronic osteomyelitis
M8666	Other chronic osteomyelitis
M8667	Other chronic osteomyelitis
M8668	Other chronic osteomyelitis
M8669	Other chronic osteomyelitis
M868	Other osteomyelitis
M8680	Other osteomyelitis
M8681	Other osteomyelitis
M8682	Other osteomyelitis
M8683	Other osteomyelitis
M8684	Other osteomyelitis
M8685	Other osteomyelitis
M8686	Other osteomyelitis
M8687	Other osteomyelitis
M8688	Other osteomyelitis
M8689	Other osteomyelitis
M869	Osteomyelitis, unspecified
M8690	Osteomyelitis, unspecified
M8691	Osteomyelitis, unspecified
M8692	Osteomyelitis, unspecified
M8693	Osteomyelitis, unspecified
M8694	Osteomyelitis, unspecified
M8695	Osteomyelitis, unspecified
M8696	Osteomyelitis, unspecified
M8697	Osteomyelitis, unspecified
M8698	Osteomyelitis, unspecified
M8699	Osteomyelitis, unspecified
M87	Osteonecrosis
M870	Idiopathic aseptic necrosis of bone
M8700	Idiopathic aseptic necrosis of bone
M8701	Idiopathic aseptic necrosis of bone
M8702	Idiopathic aseptic necrosis of bone
M8703	Idiopathic aseptic necrosis of bone
M8704	Idiopathic aseptic necrosis of bone
M8705	Idiopathic aseptic necrosis of bone
M8706	Idiopathic aseptic necrosis of bone
M8707	Idiopathic aseptic necrosis of bone

For peer review only

M8708	Idiopathic aseptic necrosis of bone
M8709	Idiopathic aseptic necrosis of bone
M871	Osteonecrosis due to drugs
M8710	Osteonecrosis due to drugs
M8711	Osteonecrosis due to drugs
M8712	Osteonecrosis due to drugs
M8713	Osteonecrosis due to drugs
M8714	Osteonecrosis due to drugs
M8715	Osteonecrosis due to drugs
M8716	Osteonecrosis due to drugs
M8717	Osteonecrosis due to drugs
M8718	Osteonecrosis due to drugs
M8719	Osteonecrosis due to drugs
M872	Osteonecrosis due to previous trauma
M8720	Osteonecrosis due to previous trauma
M8721	Osteonecrosis due to previous trauma
M8722	Osteonecrosis due to previous trauma
M8723	Osteonecrosis due to previous trauma
M8724	Osteonecrosis due to previous trauma
M8725	Osteonecrosis due to previous trauma
M8726	Osteonecrosis due to previous trauma
M8727	Osteonecrosis due to previous trauma
M8728	Osteonecrosis due to previous trauma
M8729	Osteonecrosis due to previous trauma
M873	Other secondary osteonecrosis
M8730	Other secondary osteonecrosis
M8731	Other secondary osteonecrosis
M8732	Other secondary osteonecrosis
M8733	Other secondary osteonecrosis
M8734	Other secondary osteonecrosis
M8735	Other secondary osteonecrosis
M8736	Other secondary osteonecrosis
M8737	Other secondary osteonecrosis
M8738	Other secondary osteonecrosis
M8739	Other secondary osteonecrosis
M878	Other osteonecrosis
M8780	Other osteonecrosis
M8781	Other osteonecrosis
M8782	Other osteonecrosis
M8783	Other osteonecrosis
M8784	Other osteonecrosis
M8785	Other osteonecrosis
M8786	Other osteonecrosis
M8787	Other osteonecrosis
M8788	Other osteonecrosis
M8789	Other osteonecrosis
M879	Osteonecrosis, unspecified
M8790	Osteonecrosis, unspecified

For peer review only

M8791	Osteonecrosis, unspecified
M8792	Osteonecrosis, unspecified
M8793	Osteonecrosis, unspecified
M8794	Osteonecrosis, unspecified
M8795	Osteonecrosis, unspecified
M8796	Osteonecrosis, unspecified
M8797	Osteonecrosis, unspecified
M8798	Osteonecrosis, unspecified
M8799	Osteonecrosis, unspecified
M88	Paget disease of bone [osteitis deformans]
M88	Paget's disease of bone [osteitis deformans]
M880	Paget disease of skull
M880	Paget's disease of skull
M888	Paget disease of other bones
M888	Paget's disease of other bones
M8880	Paget disease of other bones
M8880	Paget's disease of other bones
M8881	Paget disease of other bones
M8881	Paget's disease of other bones
M8882	Paget disease of other bones
M8882	Paget's disease of other bones
M8883	Paget disease of other bones
M8883	Paget's disease of other bones
M8884	Paget disease of other bones
M8884	Paget's disease of other bones
M8885	Paget disease of other bones
M8885	Paget's disease of other bones
M8886	Paget disease of other bones
M8886	Paget's disease of other bones
M8887	Paget disease of other bones
M8887	Paget's disease of other bones
M8888	Paget disease of other bones
M8888	Paget's disease of other bones
M8889	Paget disease of other bones
M8889	Paget's disease of other bones
M889	Paget disease of bone, unspecified
M889	Paget's disease of bone, unspecified
M8890	Paget disease of bone, unspecified
M8890	Paget's disease of bone, unspecified
M8891	Paget disease of bone, unspecified
M8891	Paget's disease of bone, unspecified
M8892	Paget disease of bone, unspecified
M8892	Paget's disease of bone, unspecified
M8893	Paget disease of bone, unspecified
M8893	Paget's disease of bone, unspecified
M8894	Paget disease of bone, unspecified
M8894	Paget's disease of bone, unspecified
M8895	Paget disease of bone, unspecified

For peer review only

M8895	Paget's disease of bone, unspecified
M8896	Paget disease of bone, unspecified
M8896	Paget's disease of bone, unspecified
M8897	Paget disease of bone, unspecified
M8897	Paget's disease of bone, unspecified
M8898	Paget disease of bone, unspecified
M8898	Paget's disease of bone, unspecified
M8899	Paget disease of bone, unspecified
M8899	Paget's disease of bone, unspecified
M89	Other disorders of bone
M890	Algoneurodystrophy
M8900	Algoneurodystrophy
M8901	Algoneurodystrophy
M8902	Algoneurodystrophy
M8903	Algoneurodystrophy
M8904	Algoneurodystrophy
M8905	Algoneurodystrophy
M8906	Algoneurodystrophy
M8907	Algoneurodystrophy
M8908	Algoneurodystrophy
M8909	Algoneurodystrophy
M891	Epiphyseal arrest
M8910	Epiphyseal arrest
M8911	Epiphyseal arrest
M8912	Epiphyseal arrest
M8913	Epiphyseal arrest
M8914	Epiphyseal arrest
M8915	Epiphyseal arrest
M8916	Epiphyseal arrest
M8917	Epiphyseal arrest
M8918	Epiphyseal arrest
M8919	Epiphyseal arrest
M892	Other disorders of bone development and growth
M8920	Other disorders of bone development and growth
M8921	Other disorders of bone development and growth
M8922	Other disorders of bone development and growth
M8923	Other disorders of bone development and growth
M8924	Other disorders of bone development and growth
M8925	Other disorders of bone development and growth
M8926	Other disorders of bone development and growth
M8927	Other disorders of bone development and growth
M8928	Other disorders of bone development and growth
M8929	Other disorders of bone development and growth
M893	Hypertrophy of bone
M8930	Hypertrophy of bone
M8931	Hypertrophy of bone
M8932	Hypertrophy of bone
M8933	Hypertrophy of bone

For peer review only

M8934	Hypertrophy of bone
M8935	Hypertrophy of bone
M8936	Hypertrophy of bone
M8937	Hypertrophy of bone
M8938	Hypertrophy of bone
M8939	Hypertrophy of bone
M894	Other hypertrophic osteoarthropathy
M8940	Other hypertrophic osteoarthropathy
M8941	Other hypertrophic osteoarthropathy
M8942	Other hypertrophic osteoarthropathy
M8943	Other hypertrophic osteoarthropathy
M8944	Other hypertrophic osteoarthropathy
M8945	Other hypertrophic osteoarthropathy
M8946	Other hypertrophic osteoarthropathy
M8947	Other hypertrophic osteoarthropathy
M8948	Other hypertrophic osteoarthropathy
M8949	Other hypertrophic osteoarthropathy
M895	Osteolysis
M8950	Osteolysis
M8951	Osteolysis
M8952	Osteolysis
M8953	Osteolysis
M8954	Osteolysis
M8955	Osteolysis
M8956	Osteolysis
M8957	Osteolysis
M8958	Osteolysis
M8959	Osteolysis
M896	Osteopathy after poliomyelitis
M8960	Osteopathy after poliomyelitis
M8961	Osteopathy after poliomyelitis
M8962	Osteopathy after poliomyelitis
M8963	Osteopathy after poliomyelitis
M8964	Osteopathy after poliomyelitis
M8965	Osteopathy after poliomyelitis
M8966	Osteopathy after poliomyelitis
M8967	Osteopathy after poliomyelitis
M8968	Osteopathy after poliomyelitis
M8969	Osteopathy after poliomyelitis
M898	Other specified disorders of bone
M8980	Other specified disorders of bone
M8981	Other specified disorders of bone
M8982	Other specified disorders of bone
M8983	Other specified disorders of bone
M8984	Other specified disorders of bone
M8985	Other specified disorders of bone
M8986	Other specified disorders of bone
M8987	Other specified disorders of bone

For peer review only

M8988	Other specified disorders of bone
M8989	Other specified disorders of bone
M899	Disorder of bone, unspecified
M8990	Disorder of bone, unspecified
M8991	Disorder of bone, unspecified
M8992	Disorder of bone, unspecified
M8993	Disorder of bone, unspecified
M8994	Disorder of bone, unspecified
M8995	Disorder of bone, unspecified
M8996	Disorder of bone, unspecified
M8997	Disorder of bone, unspecified
M8998	Disorder of bone, unspecified
M8999	Disorder of bone, unspecified
M90	Osteopathies in diseases classified elsewhere
M900	Tuberculosis of bone
M9000	Tuberculosis of bone
M9001	Tuberculosis of bone
M9002	Tuberculosis of bone
M9003	Tuberculosis of bone
M9004	Tuberculosis of bone
M9005	Tuberculosis of bone
M9006	Tuberculosis of bone
M9007	Tuberculosis of bone
M9008	Tuberculosis of bone
M9009	Tuberculosis of bone
M901	Periostitis in other infectious diseases classified elsewhere
M9010	Periostitis in other infectious diseases classified elsewhere
M9011	Periostitis in other infectious diseases classified elsewhere
M9012	Periostitis in other infectious diseases classified elsewhere
M9013	Periostitis in other infectious diseases classified elsewhere
M9014	Periostitis in other infectious diseases classified elsewhere
M9015	Periostitis in other infectious diseases classified elsewhere
M9016	Periostitis in other infectious diseases classified elsewhere
M9017	Periostitis in other infectious diseases classified elsewhere
M9018	Periostitis in other infectious diseases classified elsewhere
M9019	Periostitis in other infectious diseases classified elsewhere
M902	Osteopathy in other infectious diseases classified elsewhere
M9020	Osteopathy in other infectious diseases classified elsewhere
M9021	Osteopathy in other infectious diseases classified elsewhere
M9022	Osteopathy in other infectious diseases classified elsewhere
M9023	Osteopathy in other infectious diseases classified elsewhere
M9024	Osteopathy in other infectious diseases classified elsewhere
M9025	Osteopathy in other infectious diseases classified elsewhere
M9026	Osteopathy in other infectious diseases classified elsewhere
M9027	Osteopathy in other infectious diseases classified elsewhere
M9028	Osteopathy in other infectious diseases classified elsewhere
M9029	Osteopathy in other infectious diseases classified elsewhere
M903	Osteonecrosis in caisson disease

For peer review only

M9030	Osteonecrosis in caisson disease
M9031	Osteonecrosis in caisson disease
M9032	Osteonecrosis in caisson disease
M9033	Osteonecrosis in caisson disease
M9034	Osteonecrosis in caisson disease
M9035	Osteonecrosis in caisson disease
M9036	Osteonecrosis in caisson disease
M9037	Osteonecrosis in caisson disease
M9038	Osteonecrosis in caisson disease
M9039	Osteonecrosis in caisson disease
M904	Osteonecrosis due to haemoglobinopathy
M9040	Osteonecrosis due to haemoglobinopathy
M9041	Osteonecrosis due to haemoglobinopathy
M9042	Osteonecrosis due to haemoglobinopathy
M9043	Osteonecrosis due to haemoglobinopathy
M9044	Osteonecrosis due to haemoglobinopathy
M9045	Osteonecrosis due to haemoglobinopathy
M9046	Osteonecrosis due to haemoglobinopathy
M9047	Osteonecrosis due to haemoglobinopathy
M9048	Osteonecrosis due to haemoglobinopathy
M9049	Osteonecrosis due to haemoglobinopathy
M905	Osteonecrosis in other diseases classified elsewhere
M9050	Osteonecrosis in other diseases classified elsewhere
M9051	Osteonecrosis in other diseases classified elsewhere
M9052	Osteonecrosis in other diseases classified elsewhere
M9053	Osteonecrosis in other diseases classified elsewhere
M9054	Osteonecrosis in other diseases classified elsewhere
M9055	Osteonecrosis in other diseases classified elsewhere
M9056	Osteonecrosis in other diseases classified elsewhere
M9057	Osteonecrosis in other diseases classified elsewhere
M9058	Osteonecrosis in other diseases classified elsewhere
M9059	Osteonecrosis in other diseases classified elsewhere
M906	Osteitis deformans in neoplastic disease
M9060	Osteitis deformans in neoplastic disease
M9061	Osteitis deformans in neoplastic disease
M9062	Osteitis deformans in neoplastic disease
M9063	Osteitis deformans in neoplastic disease
M9064	Osteitis deformans in neoplastic disease
M9065	Osteitis deformans in neoplastic disease
M9066	Osteitis deformans in neoplastic disease
M9067	Osteitis deformans in neoplastic disease
M9068	Osteitis deformans in neoplastic disease
M9069	Osteitis deformans in neoplastic disease
M907	Fracture of bone in neoplastic disease
M9070	Fracture of bone in neoplastic disease
M9071	Fracture of bone in neoplastic disease
M9072	Fracture of bone in neoplastic disease
M9073	Fracture of bone in neoplastic disease

For peer review only

M9074	Fracture of bone in neoplastic disease
M9075	Fracture of bone in neoplastic disease
M9076	Fracture of bone in neoplastic disease
M9077	Fracture of bone in neoplastic disease
M9078	Fracture of bone in neoplastic disease
M9079	Fracture of bone in neoplastic disease
M908	Osteopathy in other diseases classified elsewhere
M9080	Osteopathy in other diseases classified elsewhere
M9081	Osteopathy in other diseases classified elsewhere
M9082	Osteopathy in other diseases classified elsewhere
M9083	Osteopathy in other diseases classified elsewhere
M9084	Osteopathy in other diseases classified elsewhere
M9085	Osteopathy in other diseases classified elsewhere
M9086	Osteopathy in other diseases classified elsewhere
M9087	Osteopathy in other diseases classified elsewhere
M9088	Osteopathy in other diseases classified elsewhere
M9089	Osteopathy in other diseases classified elsewhere
M91	Juvenile osteochondrosis of hip and pelvis
M910	Juvenile osteochondrosis of pelvis
M911	Juvenile osteochondrosis of head of femur [Legg-Calv,-Perthes]
M912	Coxa plana
M913	Pseudocoxalgia
M918	Other juvenile osteochondrosis of hip and pelvis
M9180	Other juvenile osteochondrosis of hip and pelvis
M919	Juvenile osteochondrosis of hip and pelvis, unspecified
M9190	Juvenile osteochondrosis of hip and pelvis, unspecified
M92	Other juvenile osteochondrosis
M920	Juvenile osteochondrosis of humerus
M921	Juvenile osteochondrosis of radius and ulna
M922	Juvenile osteochondrosis of hand
M923	Other juvenile osteochondrosis of upper limb
M924	Juvenile osteochondrosis of patella
M925	Juvenile osteochondrosis of tibia and fibula
M926	Juvenile osteochondrosis of tarsus
M927	Juvenile osteochondrosis of metatarsus
M928	Other specified juvenile osteochondrosis
M929	Juvenile osteochondrosis, unspecified
M93	Other osteochondropathies
M930	Slipped upper femoral epiphysis (nontraumatic)
M931	Kienb”ck disease of adults
M931	Kienb”ck's disease of adults
M932	Osteochondritis dissecans
M938	Other specified osteochondropathies
M939	Osteochondropathy, unspecified
M94	Other disorders of cartilage
M940	Chondrocostal junction syndrome [Tietze]
M941	Relapsing polychondritis
M9410	Relapsing polychondritis

For peer review only

M9411	Relapsing polychondritis
M9412	Relapsing polychondritis
M9413	Relapsing polychondritis
M9414	Relapsing polychondritis
M9415	Relapsing polychondritis
M9416	Relapsing polychondritis
M9417	Relapsing polychondritis
M9418	Relapsing polychondritis
M9419	Relapsing polychondritis
M942	Chondromalacia
M9420	Chondromalacia
M9421	Chondromalacia
M9422	Chondromalacia
M9423	Chondromalacia
M9424	Chondromalacia
M9425	Chondromalacia
M9426	Chondromalacia
M9427	Chondromalacia
M9428	Chondromalacia
M9429	Chondromalacia
M943	Chondrolysis
M9430	Chondrolysis
M9431	Chondrolysis
M9432	Chondrolysis
M9433	Chondrolysis
M9434	Chondrolysis
M9435	Chondrolysis
M9436	Chondrolysis
M9437	Chondrolysis
M9438	Chondrolysis
M9439	Chondrolysis
M948	Other specified disorders of cartilage
M9480	Other specified disorders of cartilage
M9481	Other specified disorders of cartilage
M9482	Other specified disorders of cartilage
M9483	Other specified disorders of cartilage
M9484	Other specified disorders of cartilage
M9485	Other specified disorders of cartilage
M9486	Other specified disorders of cartilage
M9487	Other specified disorders of cartilage
M9488	Other specified disorders of cartilage
M9489	Other specified disorders of cartilage
M949	Disorder of cartilage, unspecified
M9490	Disorder of cartilage, unspecified
M9491	Disorder of cartilage, unspecified
M9492	Disorder of cartilage, unspecified
M9493	Disorder of cartilage, unspecified
M9494	Disorder of cartilage, unspecified

For peer review only

M9495	Disorder of cartilage, unspecified
M9496	Disorder of cartilage, unspecified
M9497	Disorder of cartilage, unspecified
M9498	Disorder of cartilage, unspecified
M9499	Disorder of cartilage, unspecified
M95	Other acquired deformities of musculoskeletal system and connective tissue
M950	Acquired deformity of nose
M951	Cauliflower ear
M952	Other acquired deformity of head
M953	Acquired deformity of neck
M954	Acquired deformity of chest and rib
M955	Acquired deformity of pelvis
M958	Other specified acquired deformities of musculoskeletal system
M959	Acquired deformity of musculoskeletal system, unspecified
M96	Postprocedural musculoskeletal disorders, not elsewhere classified
M960	Pseudarthrosis after fusion or arthrodesis
M961	Postlaminectomy syndrome, not elsewhere classified
M962	Postradiation kyphosis
M963	Postlaminectomy kyphosis
M964	Postsurgical lordosis
M965	Postradiation scoliosis
M966	Fracture of bone following insertion of orthopaedic implant, joint prosthesis, or bone plate
M968	Other postprocedural musculoskeletal disorders
M969	Postprocedural musculoskeletal disorder, unspecified
M99	Biomechanical lesions, not elsewhere classified
M990	Segmental and somatic dysfunction
M9900	Segmental and somatic dysfunction
M9901	Segmental and somatic dysfunction
M9902	Segmental and somatic dysfunction
M9903	Segmental and somatic dysfunction
M9904	Segmental and somatic dysfunction
M9905	Segmental and somatic dysfunction
M9906	Segmental and somatic dysfunction
M9907	Segmental and somatic dysfunction
M9908	Segmental and somatic dysfunction
M9909	Segmental and somatic dysfunction
M991	Subluxation complex (vertebral)
M9910	Subluxation complex (vertebral)
M9911	Subluxation complex (vertebral)
M9912	Subluxation complex (vertebral)
M9913	Subluxation complex (vertebral)
M9914	Subluxation complex (vertebral)
M9915	Subluxation complex (vertebral)
M9916	Subluxation complex (vertebral)
M9917	Subluxation complex (vertebral)
M9918	Subluxation complex (vertebral)
M9919	Subluxation complex (vertebral)

For peer review only

M992	Subluxation stenosis of neural canal
M9920	Subluxation stenosis of neural canal
M9921	Subluxation stenosis of neural canal
M9922	Subluxation stenosis of neural canal
M9923	Subluxation stenosis of neural canal
M9924	Subluxation stenosis of neural canal
M9925	Subluxation stenosis of neural canal
M993	Osseous stenosis of neural canal
M9930	Osseous stenosis of neural canal
M9931	Osseous stenosis of neural canal
M9932	Osseous stenosis of neural canal
M9933	Osseous stenosis of neural canal
M9934	Osseous stenosis of neural canal
M9935	Osseous stenosis of neural canal
M994	Connective tissue stenosis of neural canal
M9940	Connective tissue stenosis of neural canal
M9941	Connective tissue stenosis of neural canal
M9942	Connective tissue stenosis of neural canal
M9943	Connective tissue stenosis of neural canal
M9944	Connective tissue stenosis of neural canal
M9945	Connective tissue stenosis of neural canal
M995	Intervertebral disc stenosis of neural canal
M9950	Intervertebral disc stenosis of neural canal
M9951	Intervertebral disc stenosis of neural canal
M9952	Intervertebral disc stenosis of neural canal
M9953	Intervertebral disc stenosis of neural canal
M9954	Intervertebral disc stenosis of neural canal
M9955	Intervertebral disc stenosis of neural canal
M996	Osseous and subluxation stenosis of intervertebral foramina
M9960	Osseous and subluxation stenosis of intervertebral foramina
M9961	Osseous and subluxation stenosis of intervertebral foramina
M9962	Osseous and subluxation stenosis of intervertebral foramina
M9963	Osseous and subluxation stenosis of intervertebral foramina
M9964	Osseous and subluxation stenosis of intervertebral foramina
M9965	Osseous and subluxation stenosis of intervertebral foramina
M997	Connective tissue and disc stenosis of intervertebral foramina
M9970	Connective tissue and disc stenosis of intervertebral foramina
M9971	Connective tissue and disc stenosis of intervertebral foramina
M9972	Connective tissue and disc stenosis of intervertebral foramina
M9973	Connective tissue and disc stenosis of intervertebral foramina
M9974	Connective tissue and disc stenosis of intervertebral foramina
M9975	Connective tissue and disc stenosis of intervertebral foramina
M998	Other biomechanical lesions
M9980	Other biomechanical lesions
M9981	Other biomechanical lesions
M9982	Other biomechanical lesions
M9983	Other biomechanical lesions
M9984	Other biomechanical lesions

For peer review only

M9985	Other biomechanical lesions
M9986	Other biomechanical lesions
M9987	Other biomechanical lesions
M9988	Other biomechanical lesions
M9989	Other biomechanical lesions
M999	Biomechanical lesion, unspecified
M9990	Biomechanical lesion, unspecified
M9991	Biomechanical lesion, unspecified
M9992	Biomechanical lesion, unspecified
M9993	Biomechanical lesion, unspecified
M9994	Biomechanical lesion, unspecified
M9995	Biomechanical lesion, unspecified
M9996	Biomechanical lesion, unspecified
M9997	Biomechanical lesion, unspecified
M9998	Biomechanical lesion, unspecified
M9999	Biomechanical lesion, unspecified

Epilepsy (GP)

READ_CODE	READ_DESC
1O30.	Epilepsy confirmed
F25..	Epilepsy
F250.	Generalised nonconvulsive epilepsy
F2500	Petit mal (minor) epilepsy
F2501	Pykno-epilepsy
F2502	Epileptic seizures - atonic
F2503	Epileptic seizures - akinetic
F2504	Juvenile absence epilepsy
F2505	Lennox-Gastaut syndrome
F250y	Other specified generalised nonconvulsive epilepsy
F250z	Generalised nonconvulsive epilepsy NOS
F251.	Generalised convulsive epilepsy
F2510	Grand mal (major) epilepsy
F2511	Neonatal myoclonic epilepsy
F2512	Epileptic seizures - clonic
F2513	Epileptic seizures - myoclonic
F2514	Epileptic seizures - tonic
F2515	Tonic-clonic epilepsy
F2516	Grand mal seizure
F251y	Other specified generalised convulsive epilepsy
F251z	Generalised convulsive epilepsy NOS
F252.	Petit mal status
F253.	Grand mal status
F254.	Partial epilepsy with impairment of consciousness
F2540	Temporal lobe epilepsy
F2541	Psychomotor epilepsy

For peer review only

F2542	Psychosensory epilepsy
F2543	Limbic system epilepsy
F2544	Epileptic automatism
F2545	Complex partial epileptic seizure
F254z	Partial epilepsy with impairment of consciousness NOS
F255.	Partial epilepsy without mention of impairment of consciousness
F2550	Jacksonian, focal or motor epilepsy
F2551	Sensory induced epilepsy
F2552	Somatosensory epilepsy
F2553	Visceral reflex epilepsy
F2554	Visual reflex epilepsy
F2555	Unilateral epilepsy
F2556	Simple partial epileptic seizure
F255y	Other specified partial epilepsy without mention of impairment of consciousness
F255z	Partial epilepsy without mention of impairment of consciousness NOS
F256.	Infantile spasms
F2560	Hypsarrhythmia
F2561	Salaam attacks
F256z	Infantile spasms NOS
F257.	Kojevnikov's epilepsy
F258.	Post-ictal state
F259.	Early infantile epileptic encephalopathy with suppression bursts
F25A.	Juvenile myoclonic epilepsy
F25B.	Alcohol-induced epilepsy
F25C.	Drug-induced epilepsy
F25D.	Menstrual epilepsy
F25E.	Stress-induced epilepsy
F25F.	Photosensitive epilepsy
F25G.	Severe myoclonic epilepsy in infancy
F25H.	Generalised seizure
F25X.	Status epilepticus, unspecified
F25y.	Other forms of epilepsy
F25y0	Cursive (running) epilepsy
F25y1	Gelastic epilepsy
F25y2	Localization-related(focal)(partial)idiopathic epilepsy and epileptic syndromes with seizures of localised onset
F25y3	Complex partial status epilepticus
F25y4	Benign Rolandic epilepsy
F25y5	Panayiotopoulos syndrome
F25yz	Other forms of epilepsy NOS
F25z.	Epilepsy NOS
dn...	CONTROL OF EPILEPSY
dn1..	ACETAZOLAMIDE [EPILEPSY]
dn11.	DIAMOX [EP] 500mg m/r capsules
dn12.	DIAMOX [EP] 250mg tablets
dn13.	DIAMOX [EP] 500mg injection
dn1x.	ACETAZOLAMIDE [EP] 500mg m/r capsules
dn1y.	ACETAZOLAMIDE [EP] 250mg tablets

For peer review only

dn1z.	ACETAZOLAMIDE [EP] 500mg injection
dn2..	*BECLAMIDE
dn21.	*NYDRANE 500mg tablets
dn2z.	*BECLAMIDE 500mg tablets
dn3..	CARBAMAZEPINE
dn31.	CARBAMAZEPINE 100mg tablets
dn32.	CARBAMAZEPINE 200mg tablets
dn33.	CARBAMAZEPINE 400mg tablets
dn34.	TEGRETOL 100mg tablets
dn35.	TEGRETOL 200mg tablets
dn36.	TEGRETOL 400mg tablets
dn37.	TEGRETOL 100mg/5mL sugar free liquid
dn38.	TEGRETOL RETARD 200mg m/r tabs
dn39.	TEGRETOL RETARD 400mg m/r tabs
dn3A.	*EPIMAZ 100mg tablets
dn3B.	*EPIMAZ 200mg tablets
dn3C.	*EPIMAZ 400mg tablets
dn3D.	TEGRETOL 125mg suppositories
dn3E.	TEGRETOL 250mg suppositories
dn3F.	TIMONIL RETARD 200mg m/r tablets
dn3G.	TIMONIL RETARD 400mg m/r tablets
dn3H.	*TERIL CR 200mg m/r tablets
dn3I.	*TERIL CR 400mg m/r tablets
dn3J.	CARBAGEN SR 200mg m/r tablets
dn3K.	CARBAGEN SR 400mg m/r tablets
dn3a.	CARBAMAZEPINE 200mg m/r tabs
dn3b.	CARBAMAZEPINE 400mg m/r tabs
dn3c.	TEGRETOL 100mg chewable tablets
dn3c.	TEGRETOL 100mg chewable tablets
dn3d.	TEGRETOL 200mg chewable tablets
dn3d.	TEGRETOL 200mg chewable tablets
dn3e.	*ARBIL MR 200mg m/r tablets
dn3f.	*ARBIL MR 400mg m/r tablets
dn3v.	CARBAMAZEPINE 125mg suppositories
dn3w.	CARBAMAZEPINE 250mg suppositories
dn3x.	CARBAMAZEPINE 200mg chewable tablets
dn3x.	CARBAMAZEPINE 200mg chewable tablets
dn3y.	CARBAMAZEPINE 100mg chewable tablets
dn3y.	CARBAMAZEPINE 100mg chewable tablets
dn3z.	CARBAMAZEPINE 100mg/5mL sugar free liquid
dn4..	CLONAZEPAM [EPILEPSY CONTROL]
dn41.	*RIVOTRIL 500mcg tablets
dn41.	RIVOTRIL 500micrograms tablets
dn42.	RIVOTRIL 2mg tablets
dn42.	*RIVOTRIL 2mg tablets
dn4w.	CLONAZEPAM 0.5mg/5mL sugar free oral solution
dn4x.	CLONAZEPAM 2mg/5mL sugar free oral solution
dn4y.	CLONAZEPAM 500microgram tablets

For peer review only

dn4z.	CLONAZEPAM 2mg tablets
dn5..	ETHOSUXIMIDE
dn51.	*ETHOSUXIMIDE 250mg capsules
dn52.	*ETHOSUXIMIDE 250mg/5mL elixir
dn53.	*EMESIDE 250mg capsules
dn54.	EMESIDE 250mg/5mL syrup
dn55.	*ZARONTIN 250mg capsules
dn56.	ZARONTIN 250mg/5mL syrup
dn5x.	ETHOSUXIMIDE 250mg capsules
dn5y.	*ETHOSUXIMIDE 250mg/5mL elixir
dn5z.	ETHOSUXIMIDE 250mg/5mL syrup
dn6..	METHYLPHENOBARBITAL
dn61.	*PROMINAL 30mg tablets
dn62.	*PROMINAL 60mg tablets
dn63.	*PROMINAL 200mg tablets
dn6x.	METHYLPHENOBARBITONE 30mg tablets
dn6y.	METHYLPHENOBARBITONE 60mg tablets
dn6z.	METHYLPHENOBARBITONE 200mg tablets
dn7..	PHENOBARBITAL
dn71.	PHENOBARBITAL 15mg tablets
dn72.	PHENOBARBITAL 30mg tablets
dn73.	PHENOBARBITAL 60mg tablets
dn74.	PHENOBARBITAL 100mg tablets
dn75.	PHENOBARBITONE SODIUM 30mg tablets
dn76.	PHENOBARBITONE SODIUM 60mg tablets
dn77.	PHENOBARBITONE 15mg/10mL elixir
dn78.	PHENOBARBITAL 200mg/1mL injection
dn79.	*GARDENAL 200mg/1mL injection
dn7a.	*LUMINAL 15mg tablets
dn7b.	*LUMINAL 30mg tablets
dn7c.	*LUMINAL 60mg tablets
dn7d.	PHENOBARBITAL 15mg/5mL elixir
dn8..	PHENYTOIN
dn81.	EPANUTIN 30mg/5mL suspension
dn82.	EPANUTIN 50mg Infatabs
dn83.	PHENYTOIN 50mg chewable tablets
dn8y.	PHENYTOIN 30mg/5mL suspension
dn8z.	PHENYTOIN 90mg/5mL sugar free suspension
dn9..	PHENYTOIN SODIUM
dn91.	PHENYTOIN 50mg tablets
dn92.	PHENYTOIN 100mg tablets
dn93.	EPANUTIN 25mg capsules
dn93.	*EPANUTIN 25mg capsules
dn94.	EPANUTIN 50mg capsules
dn94.	*EPANUTIN 50mg capsules
dn95.	EPANUTIN 100mg capsules
dn95.	*EPANUTIN 100mg capsules
dn96.	*EPANUTIN 300mg capsules

For peer review only

dn96.	EPANUTIN 300mg capsules
dn97.	*PENTRAN 50mg tablets
dn98.	*PENTRAN 100mg tablets
dn9w.	PHENYTOIN SODIUM 300mg capsules
dn9x.	PHENYTOIN SODIUM 25mg caps
dn9y.	PHENYTOIN SODIUM 50mg capsules
dn9z.	PHENYTOIN SODIUM 100mg capsules
dna..	PRIMIDONE
dna1.	MYSOLINE 250mg tablets
dna1.	*MYSOLINE 250mg tablets
dna2.	MYSOLINE 250mg/5mL oral suspension
dna3.	*MYSOLINE 50mg tablets
dna3.	MYSOLINE 50mg tablets
dnax.	PRIMIDONE 50mg tablets
dnay.	PRIMIDONE 250mg tablets
dnaz.	PRIMIDONE 250mg/5mL oral suspension
dnb..	SODIUM VALPROATE
dnb1.	EPILIM 100mg crushable tablets
dnb2.	EPILIM 200mg e/c tablets
dnb3.	EPILIM 500mg e/c tablets
dnb4.	EPILIM 200mg/5mL sugar free liquid
dnb5.	EPILIM 200mg/5mL syrup
dnb6.	EPILIM IV 400mg/4mL injection
dnb7.	SODIUM VALPROATE 200mg e/c tablets
dnb8.	SODIUM VALPROATE 500mg e/c tablets
dnb9.	ORLEPT STARTER PACK 200mg e/c tablets x10
dnbA.	ORLEPT 200mg/5mL sugar free liquid
dnbB.	EPIVAL CR 300mg m/r tablets
dnbC.	EPIVAL CR 500mg m/r tablets
dnbD.	EPISENTA 300mg/3mL solution for injection
dnbE.	SODIUM VALPROATE 300mg/3mL solution for injection
dnbF.	EPISENTA 150mg m/r capsules
dnbG.	EPISENTA 300mg m/r capsules
dnbH.	EPISENTA 500mg/sachet m/r granules
dnbI.	EPISENTA 1g/sachet m/r granules
dnbJ.	SODIUM VALPROATE 150mg m/r capsules
dnbK.	SODIUM VALPROATE 300mg m/r capsules
dnbL.	SODIUM VALPROATE 500mg/sachet m/r granules
dnbM.	SODIUM VALPROATE 1g/sachet m/r granules
dnbN.	SODIUM VALPROATE 1g/10mL solution for injection
dnbO.	EPISENTA 1g/10mL solution for injection
dnbP.	EPILIM CHRONOSPHERE 50mg/sachet m/r granules
dnbQ.	EPILIM CHRONOSPHERE 100mg/sachet m/r granules
dnbR.	EPILIM CHRONOSPHERE 250mg/sachet m/r granules
dnbS.	EPILIM CHRONOSPHERE 500mg/sachet m/r granules
dnbT.	EPILIM CHRONOSPHERE 750mg/sachet m/r granules
dnbU.	EPILIM CHRONOSPHERE 1g/sachet m/r granules
dnba.	*ORLEPT 200mg e/c tablets

For peer review only

dnbb.	*ORLEPT 500mg e/c tablets
dnbc.	EPILIM CHRONO 200 m/r tablets
dnbd.	EPILIM CHRONO 300 m/r tablets
dnbe.	EPILIM CHRONO 500 m/r tablets
dnbn.	SODIUM VALPROATE 50mg/sachet m/r granules
dnbo.	SODIUM VALPROATE 100mg/sachet m/r granules
dnbp.	SODIUM VALPROATE 250mg/sachet m/r granules
dnbq.	SODIUM VALPROATE 750mg/sachet m/r granules
dnbr.	SODIUM VALPROATE 200mg m/r tablets
dnbs.	SODIUM VALPROATE 300mg m/r tablets
dnbt.	SODIUM VALPROATE 500mg m/r tablets
dnbu.	SODIUM VALPROATE 400mg/4mL injection
dnbv.	SODIUM VALPROATE 100mg crushable tablets
dnbw.	SODIUM VALPROATE 200mg crushable tablets
dnbx.	SODIUM VALPROATE 500mg tablets
dnby.	SODIUM VALPROATE 200mg/5mL sugar free liquid
dnbz.	SODIUM VALPROATE 200mg/5mL syrup
dnc..	CLOBAZAM [EPILEPSY ONLY]
dnc1.	*CLOBAZAM SLS 10mg capsules
dne..	VIGABATRIN
dne1.	VIGABATRIN 500mg tablets
dne2.	SABRIL 500mg tablets
dne3.	VIGABATRIN 500mg powder sachets
dne4.	SABRIL 500mg powder sachets
dnf..	LAMOTRIGINE
dnf1.	LAMOTRIGINE 50mg tablets
dnf2.	LAMOTRIGINE 100mg tablets
dnf3.	LAMICTAL 50mg tablets
dnf4.	LAMICTAL 100mg tablets
dnf5.	LAMOTRIGINE 25mg tablets
dnf6.	LAMICTAL 25mg tablets
dnf7.	LAMICTAL 5mg dispersible tablets
dnf8.	LAMICTAL 25mg dispersible tablets
dnf9.	LAMICTAL 100mg dispersible tablets
dnfA.	LAMOTRIGINE 5mg dispersible tablets
dnfB.	LAMOTRIGINE 25mg dispersible tablets
dnfC.	LAMOTRIGINE 100mg dispersible tablets
dnfD.	LAMICTAL 200mg tablets
dnfE.	LAMOTRIGINE 200mg tablets
dnfF.	LAMICTAL MONOTHERAPY 25mg starter pack
dnfG.	LAMICTAL VALPROATE ADD-ON 25mg starter pack
dnfH.	LAMICTAL NON-VALPROATE ADD-ON 50mg starter pack
dnfJ.	LAMICTAL 2mg dispersible tablets
dnfz.	LAMOTRIGINE 2mg dispersible tablets
dng..	PIRACETAM
dng1.	NOOTROPIL 800mg tablets
dng2.	NOOTROPIL 1.2g tablets
dng3.	NOOTROPIL 33% oral solution

For peer review only

dng4.	PIRACETAM 800mg tablets
dng5.	PIRACETAM 1.2g tablets
dng6.	PIRACETAM 333.3mg/mL oral solution
dnh..	VALPROIC ACID
dnh1.	CONVULEX 150mg e/c capsules
dnh2.	CONVULEX 300mg e/c capsules
dnh3.	CONVULEX 500mg e/c capsules
dnh4.	VALPROIC ACID 150mg e/c capsules
dnh5.	VALPROIC ACID 300mg e/c capsules
dnh6.	VALPROIC ACID 500mg e/c capsules
dnh7.	DEPAKOTE 250mg e/c tablets
dnh8.	DEPAKOTE 500mg e/c tablets
dnhy.	VALPROIC ACID 500mg e/c tablets
dnhz.	VALPROIC ACID 250mg e/c tablets
dni..	FOSPHENYTOIN SODIUM
dni1.	FOSPHENYTOIN SODIUM 750mg/10mL injection concentrate
dni2.	PRO-EPANUTIN 750mg/10mL injection concentrate
dnj..	GABAPENTIN
dnj1.	GABAPENTIN 100mg capsules
dnj2.	GABAPENTIN 300mg capsules
dnj3.	GABAPENTIN 400mg capsules
dnj4.	NEURONTIN 100mg capsules
dnj5.	NEURONTIN 300mg capsules
dnj6.	NEURONTIN 400mg capsules
dnj7.	NEURONTIN 600mg tablets
dnj8.	NEURONTIN 800mg tablets
dnj9.	NEURONTIN 300mg capsules/600mg tablets titration pack
dnjA.	GABAPENTIN 50mg/mL oral solution
dnjx.	GABAPENTIN 300mg capsules/600mg tablets titration pack
dnjy.	GABAPENTIN 600mg tablets
dnjz.	GABAPENTIN 800mg tablets
dnk..	TOPIRAMATE
dnk1.	TOPIRAMATE 50mg tablets
dnk2.	TOPIRAMATE 100mg tablets
dnk3.	TOPIRAMATE 200mg tablets
dnk4.	TOPAMAX 50mg tablets
dnk5.	TOPAMAX 100mg tablets
dnk6.	TOPAMAX 200mg tablets
dnk7.	TOPIRAMATE 25mg tablets
dnk8.	TOPAMAX 25mg tablets
dnk9.	TOPIRAMATE 15mg beads in capsules
dnkA.	TOPIRAMATE 25mg beads in capsules
dnkB.	TOPAMAX SPRINKLE 15mg capsules
dnkC.	TOPAMAX SPRINKLE 25mg capsules
dnkD.	TOPIRAMATE 50mg beads in capsules
dnkE.	TOPAMAX SPRINKLE 50mg capsules
dnl..	TIAGABINE
dnl1.	TIAGABINE 5mg tablets

For peer review only

dnl2.	TIAGABINE 10mg tablets
dnl3.	TIAGABINE 15mg tablets
dnl4.	GABITRIL 5mg tablets
dnl5.	GABITRIL 10mg tablets
dnl6.	GABITRIL 15mg tablets
dnm..	OXCARBAZEPINE
dnm1.	TRILEPTAL 150 tablets
dnm2.	TRILEPTAL 300 tablets
dnm3.	TRILEPTAL 600 tablets
dnm4.	TRILEPTAL 60mg/mL sugar free oral suspension
dnmw.	OXCARBAZEPINE 60mg/mL sugar free oral suspension
dnmx.	OXCARBAZEPINE 150mg tablets
dnmy.	OXCARBAZEPINE 300mg tablets
dnmz.	OXCARBAZEPINE 600mg tablets
dno..	LEVETIRACETAM
dno1.	KEPPRA 250mg tablets
dno2.	KEPPRA 500mg tablets
dno3.	KEPPRA 1g tablets
dno4.	KEPPRA 750mg tablets
dno5.	KEPPRA 100mg/mL s/f oral solution
dno6.	KEPPRA 500mg/5mL solution for injection
dno7.	DESITREND 250mg/sachet granules
dno8.	DESITREND 500mg/sachet granules
dno9.	DESITREND 1g/sachet granules
dnoA.	DESITREND 100mg/mL oral solution
dnoB.	DESITREND 500mg/5mL concentrate for solution for infusion
dnor.	LEVETIRACETAM 1g/sachet granules
dnos.	LEVETIRACETAM 500mg/sachet granules
dnot.	LEVETIRACETAM 250mg/sachet granules
dnou.	LEVETIRACETAM 500mg/5mL solution for injection
dnov.	LEVETIRACETAM 100mg/mL s/f oral solution
dnow.	LEVETIRACETAM 750mg tablets
dnox.	LEVETIRACETAM 1g tablets
dnoy.	LEVETIRACETAM 500mg tablets
dnoz.	LEVETIRACETAM 250mg tablets
dnp..	PREGABALIN
dnp1.	LYRICA 25mg capsules
dnp2.	LYRICA 50mg capsules
dnp3.	LYRICA 75mg capsules
dnp4.	LYRICA 100mg capsules
dnp5.	LYRICA 150mg capsules
dnp6.	LYRICA 200mg capsules
dnp7.	LYRICA 300mg capsules
dnp8.	LYRICA 225mg capsules
dnp9.	LYRICA 20mg/1mL oral solution
dnpA.	LECAENT 25mg capsules
dnpB.	LECAENT 50mg capsules
dnpC.	LECAENT 75mg capsules

For peer review only

dnpD.	LECAENT 100mg capsules
dnpE.	LECAENT 150mg capsules
dnpF.	LECAENT 200mg capsules
dnpG.	LECAENT 225mg capsules
dnpH.	LECAENT 300mg capsules
dnpI.	REWISCA 25mg capsules
dnpJ.	REWISCA 50mg capsules
dnpK.	REWISCA 75mg capsules
dnpL.	REWISCA 100mg capsules
dnpM.	REWISCA 150mg capsules
dnpN.	REWISCA 200mg capsules
dnpO.	REWISCA 225mg capsules
dnpP.	REWISCA 300mg capsules
dnpr.	PREGABALIN 20mg/1mL oral solution
dnps.	PREGABALIN 225mg capsules
dnpt.	PREGABALIN 300mg capsules
dnpu.	PREGABALIN 200mg capsules
dnpv.	PREGABALIN 100mg capsules
dnpw.	PREGABALIN 150mg capsules
dnpx.	PREGABALIN 75mg capsules
dnpy.	PREGABALIN 50mg capsules
dnpz.	PREGABALIN 25mg capsules
dnq..	ZONISAMIDE
dnq1.	ZONISAMIDE 25mg capsules
dnq2.	ZONISAMIDE 50mg capsules
dnq3.	ZONISAMIDE 100mg capsules
dnq4.	ZONEGRAN 25mg capsules
dnq5.	ZONEGRAN 50mg capsules
dnq6.	ZONEGRAN 100mg capsules
dnr..	RUFINAMIDE
dnr1.	INOVELON 100mg tablets
dnr2.	INOVELON 200mg tablets
dnr3.	INOVELON 400mg tablets
dnr4.	INOVELON 40mg/mL oral suspension
dnrw.	RUFINAMIDE 40mg/mL oral suspension
dnrx.	RUFINAMIDE 400mg tablets
dnry.	RUFINAMIDE 200mg tablets
dnrz.	RUFINAMIDE 100mg tablets
dns..	STIRIPENTOL
dns1.	DIACOMIT 250mg capsules
dns2.	DIACOMIT 500mg capsules
dns3.	DIACOMIT 250mg/sachet powder for oral suspension
dns4.	DIACOMIT 500mg/sachet powder for oral suspension
dnsw.	STIRIPENDOL 500mg/sachet powder for oral suspension
dnsx.	STIRIPENTOL 250mg/sachet powder for oral suspension
dnsy.	STIRIPENTOL 500mg capsules
dnsz.	STIRIPENTOL 250mg capsules
dnt..	LACOSAMIDE

For peer review only

dnt1.	VIMPAT 200mg/20mL solution for injection
dnt2.	VIMPAT 15mg/1mL sugar free liquid
dnt3.	VIMPAT 50mg tablets
dnt4.	VIMPAT 100mg tablets
dnt5.	VIMPAT 150mg tablets
dnt6.	VIMPAT 200mg tablets
dnt7.	LACOSAMIDE 200mg/20mL solution for injection
dnt8.	LACOSAMIDE 15mg/1mL sugar free liquid
dnt9.	LACOSAMIDE 50mg tablets
dntA.	LACOSAMIDE 100mg tablets
dntB.	LACOSAMIDE 150mg tablets
dntC.	LACOSAMIDE 200mg tablets
dntD.	VIMPAT 10mg/1mL syrup
dntE.	LACOSAMIDE 10mg/1mL sugar free liquid
dnu..	ESLICARBAZEPINE
dnu1.	ZEBINIX 800mg tablets
dnu2.	ESLICARBAZEPINE ACETATE 800mg tablets
dnv..	RETIGABINE
dnv1.	TROBALT 50mg tablets
dnv2.	TROBALT 100mg tablets
dnv3.	TROBALT 200mg tablets
dnv4.	TROBALT 300mg tablets
dnv5.	TROBALT 400mg tablets
dnv6.	TROBALT tablets initiation pack
dnv7.	RETIGABINE 50mg tablets
dnv8.	RETIGABINE 100mg tablets
dnv9.	RETIGABINE 200mg tablets
dnvA.	RETIGABINE 300mg tablets
dnvB.	RETIGABINE 400mg tablets
dnvC.	RETIGABINE 50mg+100mg tablets initiation pack
dnw..	PERAMPANEL
dnw1.	FYCOMPA 2mg tablets
dnw2.	FYCOMPA 4mg tablets
dnw3.	FYCOMPA 6mg tablets
dnw4.	FYCOMPA 8mg tablets
dnw5.	FYCOMPA 10mg tablets
dnw6.	FYCOMPA 12mg tablets
dnwu.	PERAMPANEL 12mg tablets
dnwv.	PERAMPANEL 10mg tablets
dnww.	PERAMPANEL 8mg tablets
dnwx.	PERAMPANEL 6mg tablets
dnwy.	PERAMPANEL 4mg tablets
dnwz.	PERAMPANEL 2mg tablets
do...	STATUS EPILEPTICUS DRUGS
do1..	DIAZEPAM [EPILEPSY USE]
do11.	DIAZEMULS [EP] 10mg/2mL injection
do12.	STESOLID [EP] 10mg/2mL injection
do13.	*STESOLID 20mg/4mL injection

For peer review only

do14.	STESOLID 5mg/2.5mL rectal solution
do15.	STESOLID 10mg/2.5mL rectal solution
do16.	VALIUM [EP] 10mg/2mL injection
do17.	VALIUM [EP] 20mg/4mL injection
do18.	DIAZEPAM 5mg/2.5mL RecTubes
do19.	DIAZEPAM 10mg/2.5mL RecTubes
do1A.	DIAZEPAM 2.5mg/1.25mL RecTubes
do1B.	*DIAZEPAM 20mg/5mL RecTubes
do1t.	DIAZEPAM 2.5mg/1.25mL rectal solution
do1u.	DIAZEPAM 20mg/5mL rectal solution
do1v.	DIAZEPAM 10mg/2mL emulsion injection
do1w.	DIAZEPAM 10mg/2mL injection
do1x.	DIAZEPAM 5mg/2.5mL rectal solution
do1y.	DIAZEPAM 10mg/2.5mL rectal solution
do1z.	*DIAZEPAM 20mg/4mL injection
do2..	CLONAZEPAM [STATUS EPILEPSY]
do21.	*RIVOTRIL 1mg/1mL injection
do21.	RIVOTRIL 1mg/1mL injection
do2z.	CLONAZEPAM 1mg/1mL injection
do2z.	*CLONAZEPAM 1mg/1mL injection
do3..	CLOMETHIAZOLE EDISYLATE [CENTRAL NERVOUS SYSTEM USE]
do31.	HEMINEVRIN [CNS] 8mg/mL intravenous infusion
do3z.	CLOMETHIAZOLE EDISYLATE 8mg/mL intravenous infusion
do4..	LORAZEPAM [EPILEPSY]
do41.	ATIVAN [EP] 4mg/mL injection
do5..	PARALDEHYDE
do51.	*PARALDEHYDE injection 5mL
do52.	*PARALDEHYDE injection 10mL
do6..	PHENYTOIN SODIUM [STATUS EPILEPSY]
do61.	EPANUTIN [EP] 250mg/5mL injection
do6z.	PHENYTOIN SODIUM 250mg/5mL injection

ALT_CODE	DESCRIPTION
G40	Epilepsy
G400	Localization-related (focal)(partial) idiopathic epilepsy and epileptic syndromes with seizures of localized onset
G401	Localization-related (focal)(partial) symptomatic epilepsy and epileptic syndromes with simple partial seizures
G402	Localization-related (focal)(partial) symptomatic epilepsy and epileptic syndromes with complex partial seizures
G403	Generalized idiopathic epilepsy and epileptic syndromes
G404	Other generalized epilepsy and epileptic syndromes
G405	Special epileptic syndromes
G406	Grand mal seizures, unspecified (with or without petit mal)
G407	Petit mal, unspecified, without grand mal seizures
G408	Other epilepsy
G409	Epilepsy, unspecified
G41	Status epilepticus

For peer review only

G410	Grand mal status epilepticus
G411	Petit mal status epilepticus
G412	Complex partial status epilepticus
G418	Other status epilepticus
G419	Status epilepticus, unspecified

For peer review only

The RECORD statement – checklist of items, extended from the STROBE statement, that should be reported in observational studies using routinely collected health data.

	Item No.	STROBE items	Location in manuscript where items are reported	RECORD items	Location in manuscript where items are reported
**Title and abstract**					
	1	(a) Indicate the study's design with a commonly used term in the title or the abstract (b) Provide in the abstract an informative and balanced summary of what was done and what was found		RECORD 1.1: The type of data used should be specified in the title or abstract. When possible, the name of the databases used should be included.<br><br>RECORD 1.2: If applicable, the geographic region and timeframe within which the study took place should be reported in the title or abstract.<br><br>RECORD 1.3: If linkage between databases was conducted for the study, this should be clearly stated in the title or abstract.	Page 1<br><br>Page 1<br><br>Page 1
**Introduction**					
Background rationale	2	Explain the scientific background and rationale for the investigation being reported			Page 3
Objectives	3	State specific objectives, including any prespecified hypotheses			Page 3
**Methods**					
Study Design	4	Present key elements of study design early in the paper			Pages 3-4
Setting	5	Describe the setting, locations, and relevant dates, including periods of recruitment, exposure, follow-up, and data collection			Page 4

For peer review only

Participants	6	*(a) Cohort study* - Give the eligibility criteria, and the sources and methods of selection of participants. Describe methods of follow-up<br>*Case-control study* - Give the eligibility criteria, and the sources and methods of case ascertainment and control selection. Give the rationale for the choice of cases and controls<br>*Cross-sectional study* - Give the eligibility criteria, and the sources and methods of selection of participants<br><br>*(b) Cohort study* - For matched studies, give matching criteria and number of exposed and unexposed<br>*Case-control study* - For matched studies, give matching criteria and the number of controls per case		RECORD 6.1: The methods of study population selection (such as codes or algorithms used to identify subjects) should be listed in detail. If this is not possible, an explanation should be provided.<br><br>RECORD 6.2: Any validation studies of the codes or algorithms used to select the population should be referenced. If validation was conducted for this study and not published elsewhere, detailed methods and results should be provided.<br><br>RECORD 6.3: If the study involved linkage of databases, consider use of a flow diagram or other graphical display to demonstrate the data linkage process, including the number of individuals with linked data at each stage.	Page 4<br><br>See flow diagram (Figure 1)
Variables	7	Clearly define all outcomes, exposures, predictors, potential confounders, and effect modifiers. Give diagnostic criteria, if applicable.		RECORD 7.1: A complete list of codes and algorithms used to classify exposures, outcomes, confounders, and effect modifiers should be provided. If these cannot be reported, an explanation should be provided.	Page 4 Supplementary file 1
Data sources/ measurement	8	For each variable of interest, give sources of data and details of methods of assessment (measurement).<br>Describe comparability of assessment methods if there is more than one group			Page 4

For peer review only

Bias	9	Describe any efforts to address potential sources of bias			Adjusted for confounders Page 4
Study size	10	Explain how the study size was arrived at			See flow diagram (Figure 1)
Quantitative variables	11	Explain how quantitative variables were handled in the analyses. If applicable, describe which groupings were chosen, and why			Page 4
Statistical methods	12	(a) Describe all statistical methods, including those used to control for confounding<br>(b) Describe any methods used to examine subgroups and interactions<br>(c) Explain how missing data were addressed<br>(d) *Cohort study* - If applicable, explain how loss to follow-up was addressed<br>*Case-control study* - If applicable, explain how matching of cases and controls was addressed<br>*Cross-sectional study* - If applicable, describe analytical methods taking account of sampling strategy<br>(e) Describe any sensitivity analyses			Page 4
Data access and cleaning methods		..		RECORD 12.1: Authors should describe the extent to which the investigators had access to the database population used to create the study population.	Page 3

				RECORD 12.2: Authors should provide information on the data cleaning methods used in the study.	
Linkage		..		RECORD 12.3: State whether the study included person-level, institutional-level, or other data linkage across two or more databases. The methods of linkage and methods of linkage quality evaluation should be provided.	Page 4
**Results**					
Participants	13	(a) Report the numbers of individuals at each stage of the study (*e.g.*, numbers potentially eligible, examined for eligibility, confirmed eligible, included in the study, completing follow-up, and analysed)<br>(b) Give reasons for non-participation at each stage.<br>(c) Consider use of a flow diagram		RECORD 13.1: Describe in detail the selection of the persons included in the study (*i.e.,* study population selection) including filtering based on data quality, data availability and linkage. The selection of included persons can be described in the text and/or by means of the study flow diagram.	Flow diagram (Figure 1)
Descriptive data	14	(a) Give characteristics of study participants (*e.g.*, demographic, clinical, social) and information on exposures and potential confounders<br>(b) Indicate the number of participants with missing data for each variable of interest<br>(c) *Cohort study* - summarise follow-up time (*e.g.*, average and total amount)			Page 7
Outcome data	15	*Cohort study* - Report numbers of outcome events or summary measures over time<br>*Case-control study* - Report numbers in each exposure			Page 8-10

For peer review only

		category, or summary measures of exposure<br>*Cross-sectional study* - Report numbers of outcome events or summary measures			
Main results	16	(a) Give unadjusted estimates and, if applicable, confounder-adjusted estimates and their precision (e.g., 95% confidence interval). Make clear which confounders were adjusted for and why they were included<br>(b) Report category boundaries when continuous variables were categorized<br>(c) If relevant, consider translating estimates of relative risk into absolute risk for a meaningful time period			Page 9, Page 4 &5
Other analyses	17	Report other analyses done—e.g., analyses of subgroups and interactions, and sensitivity analyses			Page 5
**Discussion**					
Key results	18	Summarise key results with reference to study objectives			Page 5
Limitations	19	Discuss limitations of the study, taking into account sources of potential bias or imprecision. Discuss both direction and magnitude of any potential bias		RECORD 19.1: Discuss the implications of using data that were not created or collected to answer the specific research question(s). Include discussion of misclassification bias, unmeasured confounding, missing data, and changing eligibility over time, as they pertain to the study being reported.	Page 5
Interpretation	20	Give a cautious overall interpretation of results considering objectives,			Page 5

For peer review only

		limitations, multiplicity of analyses, results from similar studies, and other relevant evidence			
Generalisability	21	Discuss the generalisability (external validity) of the study results			Page 6
**Other Information**					
Funding	22	Give the source of funding and the role of the funders for the present study and, if applicable, for the original study on which the present article is based			Page 6
Accessibility of protocol, raw data, and programming code		..		RECORD 22.1: Authors should provide information on how to access any supplemental information such as the study protocol, raw data, or programming code.	Page 6

*Reference: Benchimol EI, Smeeth L, Guttmann A, Harron K, Moher D, Petersen I, Sørensen HT, von Elm E, Langan SM, the RECORD Working Committee. The REporting of studies Conducted using Observational Routinely-collected health Data (RECORD) Statement. *PLoS Medicine* 2015; in press.

*Checklist is protected under Creative Commons Attribution (CC BY) license.

For peer review only

BMJ Open

Health checks for adults with intellectual disability and association with survival rates – a linked electronic records matched cohort study in Wales, UK.

Journal:	*BMJ Open*
Manuscript ID	bmjopen-2021-049441.R1
Article Type:	Original research
Date Submitted by the Author:	22-Jan-2022

Complete List of Authors:	Kennedy, Natasha; Swansea University, National Centre for Population Health and Wellbeing<br>Kennedy, Jonathan; Swansea University; Swansea University<br>Kerr, Mike; Cardiff University School of Medicine<br>Dredge, Sam; Swansea University, School of Medicine<br>Brophy, Sinead; University of Swansea Institute of Health Research, School of Medicine

**Primary Subject Heading**:	Epidemiology
Secondary Subject Heading:	Public health, Health services research
Keywords:	Quality in health care < HEALTH SERVICES ADMINISTRATION & MANAGEMENT, EPIDEMIOLOGY, GENERAL MEDICINE (see Internal Medicine)

SCHOLARONE™
Manuscripts

I, the Submitting Author has the right to grant and does grant on behalf of all authors of the Work (as defined in the below author licence), an exclusive licence and/or a non-exclusive licence for contributions from authors who are: i) UK Crown employees; ii) where BMJ has agreed a CC-BY licence shall apply, and/or iii) in accordance with the terms applicable for US Federal Government officers or employees acting as part of their official duties; on a worldwide, perpetual, irrevocable, royalty-free basis to BMJ Publishing Group Ltd ("BMJ") its licensees and where the relevant Journal is co-owned by BMJ to the co-owners of the Journal, to publish the Work in this journal and any other BMJ products and to exploit all rights, as set out in our licence.

The Submitting Author accepts and understands that any supply made under these terms is made by BMJ to the Submitting Author unless you are acting as an employee on behalf of your employer or a postgraduate student of an affiliated institution which is paying any applicable article publishing charge ("APC") for Open Access articles. Where the Submitting Author wishes to make the Work available on an Open Access basis (and intends to pay the relevant APC), the terms of reuse of such Open Access shall be governed by a Creative Commons licence – details of these licences and which Creative Commons licence will apply to this Work are set out in our licence referred to above.

Other than as permitted in any relevant BMJ Author's Self Archiving Policies, I confirm this Work has not been accepted for publication elsewhere, is not being considered for publication elsewhere and does not duplicate material already published. I confirm all authors consent to publication of this Work and authorise the granting of this licence.

For peer review only

Health checks for adults with intellectual disability and association with survival rates – a linked electronic records matched cohort study in Wales, UK.

Natasha Kennedy PhD[a], Jonathan Kennedy EngD[a,b,] , Michael Kerr FRCPSYCH[c], Sam Dredge PGCE[a], Sinead Brophy PhD[a]

a. Swansea University Medical School, Data Science Building, Swansea University, Swansea, United Kingdom
b. Health Data Research UK Wales and Northern Ireland, Swansea University Medical School, Data Science Building, Swansea University, Swansea, United Kingdom.
c. Institute of Psychological Medicine and Clinical Neurosciences, Cardiff University School of Medicine, Cardiff, UK

Corresponding author: Professor Sinead Brophy, National Centre for Population Health and Wellbeing Research, Data Science Building, School of Medicine, Swansea University. SA2 8PP. UK. Tel 01792602058, email: s.brophy@swansea.ac.uk.

For peer review only

For peer review only - http://bmjopen.bmj.com/site/about/guidelines.xhtml

Abstract

Objective

To examine if mortality rates are lower in people with intellectual disability who have had a health check compared to those who have not had health checks.

Setting

General practice records of 26,954 people with an intellectual disability in Wales between 2005-2017, of which 7,650 (28.4%) with a health check were matched 1:2 with those without a health check.

Primary outcome measure:

ONS mortality data; a Cox Regression was utilised to examine time to death adjusted for co-morbidities and gender.

Results

Patients who had a health check were stratified by those who (a) had a confirmed health check i.e. Read Code for a health check (n = 7,650 (28.4 %)), and (b) had no evidence of receiving a health check in their medical record. Patients with a health check were matched for age at time of health check with two people who did not have a health check. The health check was associated with improved survival for those with autism or Down's Syndrome (Hazard Ratio (HR): 0.58 (95 % CI: 0.37 to 0.91) and HR: 0.76 (95 % CI: 0.64 to 0.91), respectively). There was no evidence of improved survival for those diagnosed with diabetes or cancer. The people who had a health check were more likely to be older, have epilepsy, and less likely to have autism or Down's Syndrome.

Conclusions

Health checks are likely to influence survival if started before a person is diagnosed with a chronic condition, especially for people with autism or Down's Syndrome.

For peer review only

Article summary: Strengths and limitations of the study:

- These findings are based on a total population cohort in one country of people with intellectual disabilities.
- Patients who have had a health check with no record in their GP data will be misclassified, consequently, the differences observed will likely be higher than those reported in this study.
- Patients who live longer are more likely to have a health check (survival bias); to account for this the cohort has been paired with age matched controls.
- However, fewer controls were available to match for older people who had a health check, this could have influenced the findings for comparisons at older ages.

peer review only

For peer review only - http://bmjopen.bmj.com/site/about/guidelines.xhtml

Introduction

People with an intellectual disability experience more health conditions such as; epilepsy[1], autism, and dental problems [2][3]. In addition, prior research [4] observed that people with an intellectual disability are at a higher risk of leading sedentary lives and becoming overweight; subsequently developing diabetes, cardiovascular disease, and respiratory disease [5]. Furthermore, this research has indicated that they are more likely to be exposed to poverty, poor housing conditions, unemployment, and other social determinates of poor health [6]. Finally, communication and ability to act on health promotion information means access to health care provision will be reduced for people with intellectual disability. Inequalities in health are apparent for people with intellectual disabilities [7]; health checks have been recommended as one component of international health policy to address the poorer health of people with intellectual disabilities [8]. Annual health checks for people with an intellectual disability and being present on the social services register were introduced into Wales in 2006 and in England in 2007. The Cardiff/Welsh annual health check for adults is aimed at early detection and treatment [9]. The 2010 Improving Health and Lives – The Learning Disabilities Public Health Observatory (IHAL) review demonstrated that the health check improves detection of unmet, potentially treatable health needs [10]. Previous studies [8] further identified that health checks lead to detection of unmet needs and targeted actions to address health needs. Additionally, a recent study indicated that General Practitioners (GP) practices with Enhanced Service (e.g.incentivised in offering the health check) had more health action plans and secondary care referrals [11]. The health check has been observed to improve detection of less serious health conditions such as ear wax obscuring one or both eardrums, and dental problems which will greatly influence a person's quality of life [8]. They have been found to improve detection of serious conditions such as cancer, and may improve the health promotion [8,9,12]. Conversely, it is not known if this translates into health gain. Few studies have evaluated the extent to which providing health checks leads to long term health benefits. In fact, a new evaluation of the impact of the Directed Enhanced Services (DES) in England found no significant difference between health check and controls (no health check) in terms of intermediate outcomes such as control of blood pressure [13]. In addition, annual health checks where not found to reduce emergency admissions, but they did appear to reduce preventable emergency admissions (e.g. those for diabetes or COPD [13]). The lack of evidence of the long-term benefits of health checks was a justification for practices not offering them, as without this evidence the time taken to organise and undertake assessments is a substantial barrier for GPs [14]. This study specifically examines if health checks are associated with better survival and lower rates of mortality compared to those who have no health check.

Methods

Study Design

The Secure Anonymised Information Linkage (SAIL) databank is a data repository which allows person-based data linkage across datasets. This databank includes Welsh GP data, hospital in- and out-patient records, as well as mortality data collected by the Office of National Statistics (ONS). SAIL comprises of over a billion anonymised records. It utilises a split-file approach to ensure

For peer review only - http://bmjopen.bmj.com/site/about/guidelines.xhtml

anonymisation, overcome issues of confidentiality, and disclosure in health-related data warehousing. Demographic data is sent to a partner organisation, NHS Wales Informatics Service, where identifiable information is removed. Clinical data are sent directly to the SAIL databank where an individual is assigned an encrypted Anonymised Linking Field (ALF). The ALF is utilised to link anonymised individuals across datasets, facilitating longitudinal analysis of an individual's journey through multiple health, education, and social datasets. Data collected by GP's are captured via Read Codes (5-digit codes related to diagnosis, medication, and process of care codes). Hospital in- and out-patient data are collected in the Patient Episode Database for Wales; this employs the International Classification of Diseases (ICD-10) clinical coding system to record clinical information regarding patients' hospital admissions, discharges, diagnoses, and operations. The ONS Mortality dataset encompasses demographic data, place of death, and underlying cause of death (also ICD-10). Anonymised routine data analysis is not subject to ethical committee approval; it is subject to Information Governance approval. This project has been approved by the IGRP (Information Governance Review Panel) which provides independent approval that the research is within the public interest and includes representatives from the National Research Ethics Service, British Medical Association, and the public.

Patient and Public Involvement: This work was discussed with the Learning Disability Ministerial Advisory Group for Wales which includes people with intellectual disabilities, and those caring for those with intellectual disabilities. Those with an intellectual disability were not involved in the design of the study or conduct of the study.

All data was selected from 2005 – 2017, patients were flagged for intellectual disability (See Supplementary file 1 and definition provided in [15]). Patients entered the study when they became eligible for a health check; at age 18 or in 2006 whichever is later. The eligible patients were stratified by (a) ever had a confirmed health check (Read Code for a health check recorded) at any time in the patients record, and (b) had no evidence of receiving a health check in their medical record.

Procedures

Statistical Analysis

STATA 15 was utilised for all analysis. Descriptive statistics for each category were generated. Patients with a health check (index case) were matched for age (+/- 5 years) at health check with 2 patients who did not have a health check, thus forming a matched cohort (Figure 1). Cox regression was employed to examine time to mortality, from date of study entry. Analysis was adjusted for gender and comorbidities (autism (with intellectual disability), Down's Syndrome, diabetes, epilepsy, and cancer) as these were identified as confounding variables; the cluster command in STATA was utilised to account for matching design. The incidence of death (deaths over number of person years follow up) are presented for each co-morbidity separately. However, the comparison patient was not matched on co-morbidity in this sub-analysis. People were censored if they moved out of Wales or were lost to follow up. This dataset included everyone with an intellectual disability over the age of 18 since 2006.

For peer review only - http://bmjopen.bmj.com/site/about/guidelines.xhtml

Results

There were 26,954 people with an intellectual disability between 2006 and 2017 in Wales (11-year period), of these 7650 (28.4 %) have a GP record of ever having had a health check (see Table 1). Consequently, this indicates that 71.6 % of people with intellectual disabilities have no record of having a health check in their electronic medical records (e.g., 19304/26954). The proportion of people who have a health check by year is between 1.69 % (in 2006 when health checks began) and 12.4 % in 2015 (see Figure 2); subsequently, each year approximately 87.6 % (e.g., if at most 12.4% have a health check) of eligible people do not have a health check.

Patients who received a health check were more likely to be older (see Table 1); the average cohort age was 40.5 years (health check) compared to 31.3 years (No health check), resulting in a difference of 9.2 years (95 % CI: 8.8 to 9.7). Additionally, the health check cohort were more likely to have epilepsy; 39.3 % in comparison to 28.7 % for the control cohort (difference 10.6 % (95 % CI: 9.3 % to 11.9 %)). Those with autism & ID were less likely to undergo a health check (15 % of those with autism have had a health check). There was no difference in socio-economic level of those who had a health check compared to those who did not (see Table 1).

Those receiving a health check were on average older than those not having health checks, in order to adjust for this survival bias, each index case was matched for the age (+/- 5 years) at health check with two patients who had no record of undergoing a health check (see Table 2). There was a trend for those who had a health check to have a lower rate of death compared to their matched comparisons (2.5 per 1000 per year fewer deaths, Figure 3). Results also indicated that those with autism (HR: 0.58 (95 % CI: 0.37 to 0.91)) and those with Down's Syndrome (HR: 0.76 (95 % CI: 0.64 to 0.91)) attained better survival rates when receiving a health check (Table 2). Conversely, analysis indicated no significant increase in survival rate if a patient had diabetes, epilepsy, or cancer. However, in nearly every case the diagnosis of these conditions happened before the date of the health check.

Screening for cancer was very low in both the health check and no health check groups (7.6 % and 5.3 % respectively); there was no evidence of lower death rates in those who had cancer and received a health check compared to those who did not have a health check. There was a slight trend to higher rate of death in those who have a health check and have cancer. However, it should be noted that the comparison matches would have been unlikely to also have cancer.

Discussion:

This study examined the medical records of 26,954 people with an intellectual disability and observed that having a health check was associated with reduced mortality for people with autism and those with Down's syndrome. Minimal evidence of reduced mortality rates was observed for those

For peer review only - http://bmjopen.bmj.com/site/about/guidelines.xhtml

diagnosed with conditions such as diabetes or epilepsy; furthermore, no evidence was obtained to indicate that health check improved outcomes for people diagnosed with cancer.

This study only examined those who had a record of having at least one health check recorded in their medical notes with a READ code. If a person received a health check but this was not coded or recorded as such, these individuals would have been misclassified and categorised into the no health check group. Previous studies indicated that the number of people having health checks was 41 % in 2008 in Wales [16]; this number was established on GP submissions to the Community, Primary Care, and the Health Services Policy Division of the Welsh Assembly Government. However, this study observed that 28.4 % of people with an intellectual disability had ever received a health check coded in their medical records. This difference signifies that approximately 69 % of those who might have undergone a health check had it coded in their notes, thus, the health check cohort in this study could be missing a third of the people who received a health check. Consequently, the true difference between those who have health checks and those who do not may be larger than this study has detected due to a misclassification error. In addition, this study exclusively followed individuals who are registered with their GP and had a code in their medical record identifying that they had an intellectual disability. This study could not observe the level of intellectual disability. However, the results indicated that more people who received the health check also possessed a diagnosis of epilepsy. This would suggest that those with a more severe intellectual disability are most likely to be given a health check; thus are likely to have had a higher mortality rate. This assumption is supported by prior research [13] which found those who have health checks are older, have higher levels of support needs, and are more likely to be in communal living. There were fewer controls available in the older age groups, and this means the controls were often younger than the case (within 5 years), this could influence the finding for comparisons at older ages.

The number of eligible people having a health check recorded by the GP practice each year was approximately 10 - 12 %. This indicates that many people may have received one health check but not undergone the health check annually, and that the majority of people with an intellectual disability never have a health check. The study identified a lower than expected uptake of health checks in the adult population in Wales, with 71.6 % having no record of a health check. Whilst the methodology cannot identify the reason for this, this indication from the data is concerning. It appears likely that adults are not being offered health checks; either as they are not recognised as being eligible for a health check, or there is a barrier to accessing checks when offered. Further exploration of this disparity in delivery is required.

Health checks have been indicated to be valued by carers and people with an intellectual disability; they have been revealed to not increase costs, and detect health requirements earlier[7,13]. This study demonstrates that they are also associated with a long-term gain in improving survival for people with an intellectual disability, especially for those with autism or Down's syndrome. The findings of the study suggest that health checks may be most beneficial for prevention of morbidities; it did not find evidence that a health check improves survival when a person has existing health conditions, such as diabetes or cancer. It is likely that once diagnosed, those with ID have the same care for their

For peer review only - http://bmjopen.bmj.com/site/about/guidelines.xhtml

diabetes/epilepsy/cancer whether receiving health checks or not. Previous research has demonstrated that emergency admission for these conditions is reduced [13] when a person undergoes health checks. The health check may improve prevention and therefore be associated with improved survival for those without existing co-morbidities. There is the argument that the health check itself is not associated with improved survival; instead, this relationship is confounded by the health check being provided by more engaged GP practices [9], more engaged family members, those with paid carers, and better overall care. However, the lack of association of the health check with enhanced survival when diagnosed with a chronic condition, especially those with cancer, and a lack of association with socioeconomic deprivation found in this study, would refute this argument of confounding. Barriers and facilitators for health checks for cardiometabolic disease was reported to be more associated with the individual rather than home environment/socio economics/engagement [17]. Prior research concluded that factors such as feeling healthy, and practical issues such as the type of invitation, as well as availability of an easy appointment acted as barriers; consequently, an argument that engaged families rather than a health check leads to benefit does not appear to be supported by the existing evidence [17].

Conclusions

Health checks are associated with a trend to improved survival for people with intellectual disabilities, especially for people with autism (with intellectual disability) and Down's Syndrome. Increasing the uptake of health checks could help with prevention of morbidities and improve survival for people who do not already have chronic disease. However, there was limited evidence from this work that survival is improved when a person has existing morbidities. This study indicates benefits associated with health checks, in terms of lower rates of mortality for those with autism or Down's Syndrome.

Contributorship statement:

The concept of the study and design was by MK, SB. The dataset was prepared by JK and analysis undertaken by NK with supervision from SB and MK. The findings and interpretation was after discussion with stakeholders in a consultation which was undertaken by SD. All authors contributed to drafting the paper and all have given approval of the final submission.

Competing interests

None to declare.

Funding

This work was supported by Health and Care Research Wales (National Centre for Population Health and Well-being), and Health Data Research UK (NIWA1) which receives its funding from HDR UK Ltd. HDR UK Ltd is funded by the UK Medical Research Council, Engineering and Physical Sciences Research Council, Economic and Social Research Council, Department of Health and Social Care (England), Chief Scientist Office of the Scottish Government Health and Social Care Directorates,

For peer review only - http://bmjopen.bmj.com/site/about/guidelines.xhtml

Health and Social Care Research and Development Division (Welsh Government), Public Health Agency (Northern Ireland), British Heart Foundation (BHF), and the Wellcome Trust.

Data sharing statement

The dataset used in this article is available via the Secure Anonymised Information Linkage (SAIL) databank, which is part of the national ehealth records infrastructure for Wales. 19. The data is accessible by applying to SAIL. For further information on the SAIL databank and enquiries in how to access the data, please visit the SAIL website (http://www.saildatabank.com).

Acknowledgements

This study uses anonymised data provided by patients, collected by the NHS as part of their care and support and held in the Secure Anonymised Information Linkage (SAIL) Databank. We would like to acknowledge all data providers that make anonymised data available for research. All data used can be accessed by contacting the SAIL gateway (https://saildatabank.com/).

For peer review only

Figures:

Figure 1: Flow diagram of participant inclusion.

Figure 2: Number of health checks per year.

Figure 3: Survival in those who have a health check compared to no health check group (2:1 matched (for age +/- 5 years).

For peer review only - http://bmjopen.bmj.com/site/about/guidelines.xhtml

Table 1: Demographics of those who have a health check compared to those with no health check (from time eligible for health check)

	No health check	Health check	Difference (95% CI)
**Demographic Characteristics**			
**Total (n)**	19304 (71.6 %)	7650 (28.4 %)	
**Average number of years of follow-up**	8.37	3.85	
**Mean age (sdev)**	31.3 (17.7)	40.5 (16.4)	-9.2 (-9.7 to -8.8)
**Age at baseline**			
**18 to 50 years old**	16070 (83.2%)	5539 (72.4%)	10.8% (9.7 to 12.0)
**More than 50 years old**	3234 (16.8 %)	2111 (27.6 %)	-10.8% (-12.0 to -9.7)
**Male**	11655 (60.4 %)	4401 (57.5 %)	2.8% (1.5 to 4.2)
**Autism**	6290 (32.6 %)	1180 (15.4 %)	17.2% (16.1 to 18.2)
**Down's Syndrome**	3364 (17.4 %)	915 (12.0 %)	5.5 % (4.6 to 6.4)
**Diabetes**	2332 (12.1 %)	1030 (13.5 %)	-1.4% (-2.3 to -0.5)
**Epilepsy**	5536 (28.7 %)	3004 (39.3 %)	-10.6% (-11.9 to -9.3)
**Cancer Screening**	1030 (5.3 %)	582 (7.6 %)	-2.3 % (-3.0 to -1.6)
**Cancer**	3095 (16.0 %)	1040 (13.6 %)	2.4% (1.5 to 3.4)
**Died**	2527 (13.1 %)	751 (9.8 %)	3.3% (2.4 to 4.0)
**Townsend Scores**			
**1**	2515 (13.0 %)	1087 (14.2 %)	-1.2 (-2.1 to -0.3)
**2**	2978 (15.4%)	1137 (14.9%)	0.6 (-0.4 to 1.5)
**3**	3317 (17.2%)	1535 (20.1 %)	-2.9 (-3.9 to -1.9)
**4**	3778 (19.6%)	1706 (22.3 %)	-2.7 (-3.8 to -1.7)
**5 (most deprived)**	4468 (23.1%)	1829 (23.9 %)	-0.8(-1.9 to 0.4)
**N/A**	2248 (11.6%)	356 (4.7%)	7.0 (6.3 to 7.6)

For peer review only

For peer review only - http://bmjopen.bmj.com/site/about/guidelines.xhtml

Table 2: Survival in those who have a health check compared to no health check group (2:1 matched (for age +/- 5 years).

	No health check (2 matches) N=12152	Health check (Index case) N=7650
Average Number of Years of follow-up	10.39	5.74
Survival (time to death)		
Total (deaths per 1000/year)	19.6 (95 % CI: 18.8 – 20.3)	17.1 (95 % CI: 15.9 – 18.4)
Hazard ratio adjusted for co-morbidities and gender	1	0.94 (95 % CI: 0.87 – 1.01)
Males (deaths per 1000/year)	19.0 (95 % CI: 18.0 – 20.1)	16.9 (95 % CI: 15.3 – 18.6)
Hazard Ratio adjusted for co-morbidities and gender	1	0.93 (95 % CI: 0.83 – 1.03)
Females (deaths per 1000/year)	20.2 (95 % CI: 19.1 – 21.4)	17.4 (95 % CI: 15.6 – 19.4)
Hazard Ratio adjusted for co-morbidities and gender	1	0.94 (95 % CI: 0.84 - 1.07)
For those with Autism	8.4 (95 % CI: 7.4 – 9.6)	3.55 (95 % CI: 2.3 – 5.4)
Crude Hazard Ratio	1	**0.58 (95 % CI: 0.37 - 0.91)**
Adjusted for co-morbidities and gender	1	**0.56 (95 % CI: 0.36 – 0.89)**

For peer review only - http://bmjopen.bmj.com/site/about/guidelines.xhtml

For those with Down's Syndrome	35.6 (95 % CI: 32.8- 38.6)	27.2 (95 % CI: 23.2 - 31.9)
Crude Hazard Ratio	1	**0.76 (95 % CI: 0.64 – 0.91)**
Adjusted for co-morbidities and gender	1	**0.80 (95 % CI: 0.67 – 0.96)**
For those with Diabetes	31.7 (95 % CI: 29.4 – 34.2)	27.6 (95 % CI: 23.8 – 32.2)
Crude Hazard Ratio	1	0.97 (95 % CI: 0.81 – 1.15)
Adjusted for co-morbidities and gender	1	0.98 (95 % CI: 0.82 – 1.16)
For those with Epilepsy	24.3 (95 % CI: 22.9 – 25.7)	21.9 (95 % CI: 19.8 – 24.2)
Crude Hazard Ratio	1	1.03 (95 % CI: 0.91 - 1.16)
Adjusted for co-morbidities and gender	1	1.07 (95 % CI: 0.95 -1.20)
For those with Cancer Screening	25.4 (95 % CI: 22.6 – 28.6)	21.2 (95 % CI: 17.1 – 26.3)
Crude Hazard Ratio	1	1.0 (95 % CI: 0.77 – 1.30)
Adjusted for co-morbidities and gender	1	1.08 (95 % CI: 0.83 -1.40)
For those with Cancer	36.6 (95 % CI: 34.3 – 39.0)	38.5 (95 % CI: 33.7 – 43.9)
Crude Hazard Ratio	1	1.18 (95 % CI: 1.0 – 1.4)
Adjusted for co-morbidities and gender	1	1.18 (95 % CI: 1.0 - 1.4)

For peer review only

References:

[1] Kinnear D, Morrison J, Allan L, Henderson A, Smiley E, Cooper SA. Prevalence of physical conditions and multimorbidity in a cohort of adults with intellectual disabilities with and without Down syndrome: cross-sectional study. BMJ Open. 2018 Feb 5;8(2):e018292. doi: 10.1136/bmjopen-2017-018292. PMID: 29431619; PMCID: PMC5829598.

[2] van Schrojenstein Lantman-de Valk HM, Walsh PN. Managing health problems in people with intellectual disabilities. BMJ. 2008 Dec 8;337:a2507. doi: 10.1136/bmj.a2507. PMID: 19064601.

[3] Ward LM, Cooper SA, Hughes-McCormack L, Macpherson L, Kinnear D. Oral health of adults with intellectual disabilities: a systematic review. J Intellect Disabil Res. 2019 Nov;63(11):1359-1378. doi: 10.1111/jir.12632. Epub 2019 May 23. PMID: 31119825.

[4] Oeseburg, B. , Dijkstra, G. J., Groothoff, J. W., Reijneveld, S. A. , & Jansen, D. E.M.C. (2011). Prevalence of Chronic Health Conditions in Children With Intellectual Disability: A Systematic Literature Review. *Intellectual and Developmental Disabilities*, *49(2),*59-85. https://doi.org/10.1352/1934-9556-49.2.59

[5] Lhatoo SD, Sander JW. The epidemiology of epilepsy and learning disability. Epilepsia. 2001;42 Suppl 1:6-9; discussion 19-20. doi: 10.1046/j.1528-1157.2001.00502.x. PMID: 11422344.

[6] Turner S. Health Inequalities and people with learning disabilities in the UK: 2011. In: Improving Health and Lives. Learning Disabilities Oberatory;2021.

[7] Kinnear D, Morrison J, Allan L, et al. Prevalence of physical conditions and multimorbidity in a cohort of adults with intellectual disabilities with and without Down syndrome: cross-sectional study. BMJ Open. 2018 Feb;8(2):e018292. DOI: 10.1136/bmjopen-2017-018292. PMID: 29431619; PMCID: PMC5829598.

[8] Robertson J, Hatton C, Emerson E, Baines S. The impact of health checks for people with intellectual disabilities: an updated systematic review of evidence. Res Dev Disabil. 2014 Oct;35(10):2450-62. doi: 10.1016/j.ridd.2014.06.007. Epub 2014 Jun 28. PMID: 24984052.

[9] Lowe, K., Felce, D., Baxter, H., Dunstan, F., Houston, H., Jones, G., Grey, J., Felce, J., & Kerr, M. (2008). The Impact of Checking the Health of Adults with Intellectual Disabilities on Primary Care Consultation Rates, Health Promotion and Contact with Specialists. Journal of Applied Research in Intellectual Disabilities, 21(6), 597 - 602. https://doi.org/10.1111/j.1468-3148.2008.00432.x

[10] Robertson, J., Roberts, H., & Emerson, E. (2010). Health checks for people with learning disabilities: A systematic review of evidence. Improving Health & Lives: Learning Disabilities Observatory.

[11] Buszewicz M, Welch C, Horsfall L, Nazareth I, Osborn D, Hassiotis A, Glover G, Chauhan U, Hoghton M, Cooper SA, Moulster G, Hithersay R, Hunter R, Heslop P, Courtenay K, Strydom A. Assessment of an incentivised scheme to provide annual health checks in primary care for adults with intellectual disability: a longitudinal cohort study. Lancet Psychiatry. 2014 Dec;1(7):522-30. doi: 10.1016/S2215-0366(14)00079-0. Epub 2014 Dec 3. PMID: 26361311.

[12] Chauhan U, Reeve J, Kontopantelis E, Hinder S, Nelson P, Doran T. Impact of the English directly enhanced service (DES) for learning disability. In. Manchester: Manchester University; 2012.

For peer review only - http://bmjopen.bmj.com/site/about/guidelines.xhtml

[13] Carey, I. M., Hosking, F. J., Harris, T., DeWilde, S., Beighton, C., Shah, S. M., & Cook, D. G. (2017). Do health checks for adults with intellectual disability reduce emergency hospital admissions? Evaluation of a natural experiment. Journal of Epidemiology and Community Health, 71(1), 52-58. https://doi.org/10.1136/jech-2016-207557

[14] Walmsley J. If annual health checks for people with learning disabilities can address failings identified in their health care, why are more GPs not doing them? BMJ 2009;338:b1261.

[15] Brophy S, Kennedy J, Fernandez-Gutierrez F, John A, Potter R, Linehan C, Kerr M. Characteristics of Children Prescribed Antipsychotics: Analysis of Routinely Collected Data. J Child Adolesc Psychopharmacol. 2018 Apr;28(3):180-191. Doi: 10.1089/cap.2017.0003. Epub 2018 Feb 27. PMID: 29486137; PMCID: PMC5905863.

[16] Perry J, Kerr M, Felce D, Bartley S, Tomlinson J. Monitoring the public health impact of health checks for adults with learning disability in Wales. In.: Public Health Wales.; 2010

[17] de Waard AM, Wändell PE, Holzmann MJ, Korevaar JC, Hollander M, Gornitzki C, de Wit NJ, Schellevis FG, Lionis C, Søndergaard J, Seifert B, Carlsson AC; SPIMEU Research Group. Barriers and facilitators to participation in a health check for cardiometabolic diseases in primary care: A systematic review. Eur J Prev Cardiol. 2018 Aug;25(12):1326-1340. doi: 10.1177/2047487318780751. Epub 2018 Jun 19. PMID: 29916723; PMCID: PMC6097107.

For peer review only - http://bmjopen.bmj.com/site/about/guidelines.xhtml

Figure 1: Flow diagram of participant inclusion.

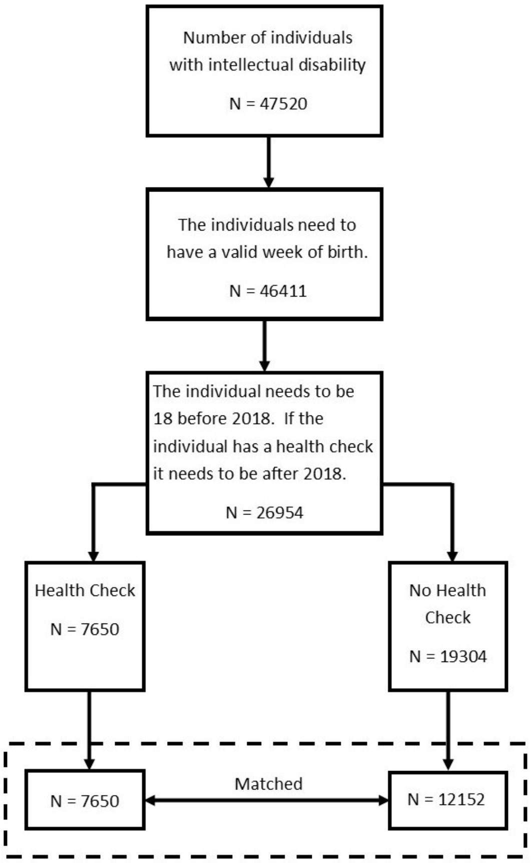

Figure 1: Flow diagram of participant inclusion

134x224mm (150 x 150 DPI)

Figure 2: Number of health checks per year.

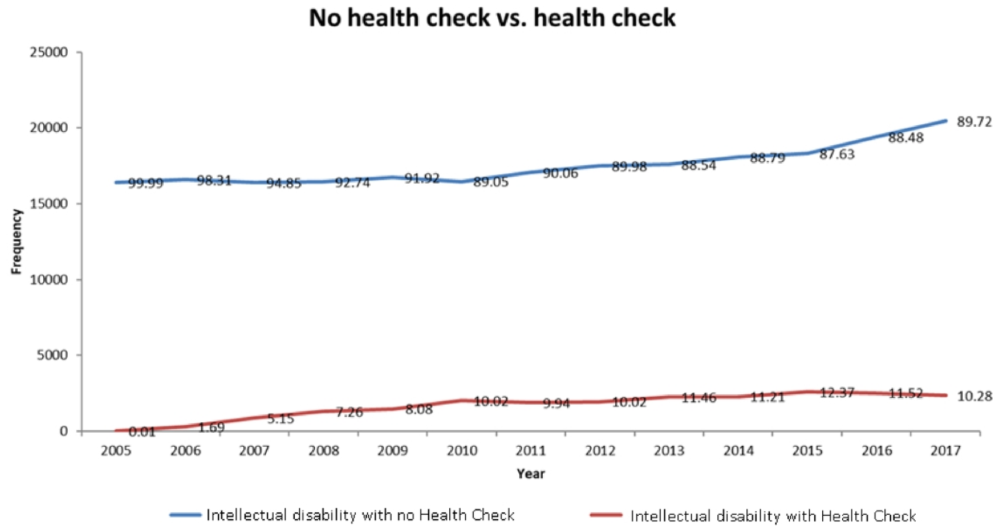

Number of health checks per year

190x124mm (300 x 300 DPI)

Figure 3: Survival in those who have a health check compared to no health check group (2:1 matched (for age +/- 5 years).

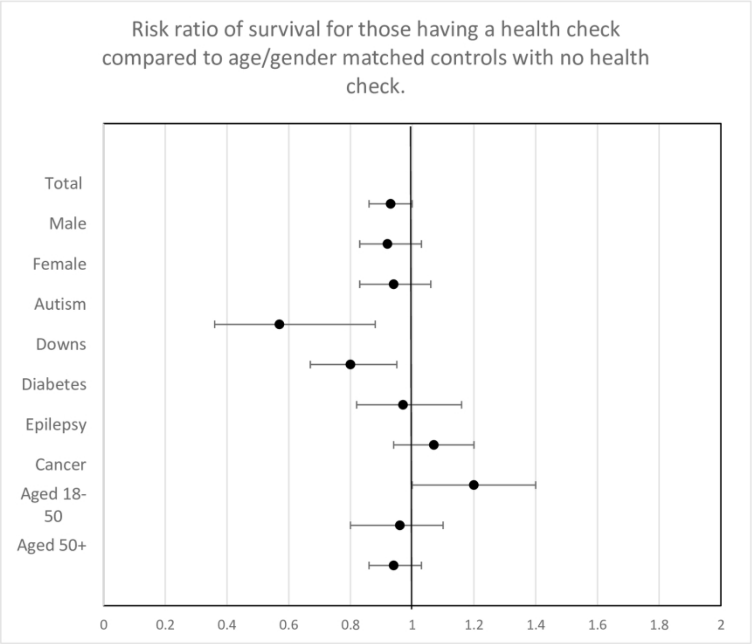

1

Survival in those who have a health check compared to no health check group (2:1 matched (for age +/- 5 years).

143x222mm (300 x 300 DPI)

Health Assessment

READ_CODE	READ_DESC
9HB5.	Learning disabilities annual health assessment

Downs (GP)

READ_CODE	READ_DESC
PJ0..	Down's syndrome - trisomy 21
PJ00.	Trisomy 21, meiotic nondisjunction
PJ0z.	Down's syndrome NOS
PJ02.	Trisomy 21, translocation
PJ01.	Trisomy 21, mosaicism

Hospital

ALT_CODE	DESCRIPTION
Q900	Trisomy 21, meiotic nondisjunction
Q901	Trisomy 21, mosaicism (mitotic nondisjunction)
Q902	Trisomy 21, translocation
Q909	Down syndrome, unspecified
Q909	Down's syndrome, unspecified

ALT_CODE	DESCRIPTION
Q90	Down syndrome

Autism (GP)

READ_CODE	READ_DESC
1J9..	Suspected autism
E1401	Residual infantile autism
E1400	Active infantile autism
E140.	Infantile autism
Eu841	[X]Atypical autism
E140z	Infantile autism NOS
Eu840	[X]Childhood autism

Hospital

ALT_CODE	DESCRIPTION
F840	Childhood autism
F841	Atypical autism

For peer review only

Learning disability (GP)

READ_CODE	READ_DESC
1J9..	Suspected autism
69DB.	Learning disability health examination
8Ce6.	Preferred place of care - learning disability unit
8H4f.	Referral to learning disabilities psychiatrist
8HHP.	Referral to learning disability team
8Hg2.	Discharge from learning disability team
918e.	On learning disability register
94Z9.	Preferred place of death: learning disability unit
9HB..	Learning disabilities administration status
9HB0.	Learning disabilities health action plan declined
9HB1.	Learning disabilities health action plan offered
9HB2.	Learning disabilities health action plan reviewed
9HB3.	Learning disabilities health assessment
9HB4.	Learning disabilities health action plan completed
9HB5.	Learning disabilities annual health assessment
9HB6.	Learning disabilities annual health assessment declined
9HB7.	Did not attend learning disabilities annual health assessment
9N0y.	Seen in learning disabilities clinic
9hL..	Exception reporting: learning disability quality indicators
9mA..	Learning disability annual health check invitation
9mA0.	Learning disability annual health check verbal invitation
9mA1.	Learning disability annual health check telephone invitation
9mA2.	Learning disability annual health check letter invitation
9mA20	Learning disability annual health check invitation first letter
9mA21	Learning disability annual health check invitation second letter
9mA22	Learning disability annual health check invitation third letter
E140.	Infantile autism
E1400	Active infantile autism
E1401	Residual infantile autism
E140z	Infantile autism NOS
E3...	Mental retardation
E30..	Mild mental retardation, IQ in range 50-70
E31..	Other specified mental retardation
E310.	Moderate mental retardation, IQ in range 35-49
E311.	Severe mental retardation, IQ in range 20-34
E312.	Profound mental retardation with IQ less than 20
E31z.	Other specified mental retardation NOS
E3y..	Other specified mental retardation
E3z..	Mental retardation NOS
Eu7..	[X]Mental retardation
Eu70.	[X]Mild mental retardation
Eu700	[X]Mild mental retardation with the statement of no, or minimal, impairment of behaviour
Eu701	[X]Mild mental retardation, significant impairment of behaviour requiring attention or treatment

For peer review only

Eu70y	[X]Mild mental retardation, other impairments of behaviour
Eu70z	[X]Mild mental retardation without mention of impairment of behaviour
Eu71.	[X]Moderate mental retardation
Eu710	[X]Moderate mental retardation with the statement of no, or minimal, impairment of behaviour
Eu711	[X]Moderate mental retardation, significant impairment of behaviour requiring attention or treatment
Eu71y	[X]Mod retard oth behav impair
Eu71z	[X]Moderate mental retardation without mention of impairment of behaviour
Eu72.	[X]Severe mental retardation
Eu720	[X]Severe mental retardation with the statement of no, or minimal, impairment of behaviour
Eu721	[X]Severe mental retardation, significant impairment of behaviour requiring attention or treatment
Eu72y	[X]Severe mental retardation, other impairments of behaviour
Eu72z	[X]Severe mental retardation without mention of impairment of behaviour
Eu73.	[X]Profound mental retardation
Eu730	[X]Profound mental retardation with the statement of no, or minimal, impairment of behaviour
Eu731	[X]Profound mental retardation, significant impairment of behaviour requiring attention or treatment
Eu73y	[X]Profound mental retardation, other impairments of behaviour
Eu73z	[X]Profound mental retardation without mention of impairment of behaviour
Eu7y.	[X]Other mental retardation
Eu7y0	[X]Other mental retardation with the statement of no, or minimal, impairment of behaviour
Eu7y1	[X]Other mental retardation, significant impairment of behaviour requiring attention or treatment
Eu7yy	[X]Other mental retardation, other impairments of behaviour
Eu7yz	[X]Other mental retardation without mention of impairment of behaviour
Eu7z.	[X]Unspecified mental retardation
Eu7z0	[X]Unspecified mental retardation with the statement of no, or minimal, impairment of behaviour
Eu7z1	[X]Unspecified mental retardation, significant impairment of behaviour requiring attention or treatment
Eu7zy	[X]Unspecified mental retardation, other impairments of behaviour
Eu7zz	[X]Unspecified mental retardation without mention of impairment of behaviour
Eu814	[X]Moderate learning disability
Eu815	[X]Severe learning disability
Eu816	[X]Mild learning disability
Eu817	[X]Profound learning disability
Eu818	[X]Specific learning disability
Eu840	[X]Childhood autism
Eu841	[X]Atypical autism
PJ0..	Down's syndrome - trisomy 21
PJ00.	Trisomy 21, meiotic nondisjunction
PJ01.	Trisomy 21, mosaicism
PJ02.	Trisomy 21, translocation
PJ0z.	Down's syndrome NOS

For peer review only

Hospital

ALT_CODE	DESCRIPTION
F79X	Unspecified mental retardation
Q18.9	Trisomy 21, meiotic nondisjunction
F84.0	Pervasive developmental disorders
Q90	Down syndrome
Q900	Trisomy 21, meiotic nondisjunction
Q901	Trisomy 21, mosaicism (mitotic nondisjunction)
Q902	Trisomy 21, translocation
Q909	Down syndrome, unspecified

Diabetes (GP)

READ_CODE	READ_DESC
C10..	Diabetes mellitus
C100.	Diabetes mellitus with no mention of complication
C1000	Diabetes mellitus, juvenile type, with no mention of complication
C1001	Diabetes mellitus, adult onset, with no mention of complication
C100z	Diabetes mellitus NOS with no mention of complication
C101.	Diabetes mellitus with ketoacidosis
C1010	Diabetes mellitus, juvenile type, with ketoacidosis
C1011	Diabetes mellitus, adult onset, with ketoacidosis
C101y	Other specified diabetes mellitus with ketoacidosis
C101z	Diabetes mellitus NOS with ketoacidosis
C102.	Diabetes mellitus with hyperosmolar coma
C1020	Diabetes mellitus, juvenile type, with hyperosmolar coma
C1021	Diabetes mellitus, adult onset, with hyperosmolar coma
C102z	Diabetes mellitus NOS with hyperosmolar coma
C103.	Diabetes mellitus with ketoacidotic coma
C1030	Diabetes mellitus, juvenile type, with ketoacidotic coma
C1031	Diabetes mellitus, adult onset, with ketoacidotic coma
C103y	Other specified diabetes mellitus with coma
C103z	Diabetes mellitus NOS with ketoacidotic coma
C104.	Diabetes mellitus with renal manifestation
C1040	Diabetes mellitus, juvenile type, with renal manifestation
C1041	Diabetes mellitus, adult onset, with renal manifestation
C104y	Other specified diabetes mellitus with renal complications
C104z	Diabetes mellitus with nephropathy NOS
C105.	Diabetes mellitus with ophthalmic manifestation
C1050	Diabetes mellitus, juvenile type, with ophthalmic manifestation
C1051	Diabetes mellitus, adult onset, with ophthalmic manifestation
C105y	Other specified diabetes mellitus with ophthalmic complications
C105z	Diabetes mellitus NOS with ophthalmic manifestation
C1060	Diabetes mellitus, juvenile type, with neurological manifestation
C1061	Diabetes mellitus, adult onset, with neurological manifestation

For peer review only

C107.	Diabetes mellitus with peripheral circulatory disorder
C1070	Diabetes mellitus, juvenile type, with peripheral circulatory disorder
C1071	Diabetes mellitus, adult onset, with peripheral circulatory disorder
C1072	Diabetes mellitus, adult with gangrene
C1073	IDDM with peripheral circulatory disorder
C1074	NIDDM with peripheral circulatory disorder
C107y	Other specified diabetes mellitus with peripheral circulatory complications
C107z	Diabetes mellitus NOS with peripheral circulatory disorder
C108.	Insulin dependent diabetes mellitus
C1080	Insulin-dependent diabetes mellitus with renal complications
C1081	Insulin-dependent diabetes mellitus with ophthalmic complications
C1082	Insulin-dependent diabetes mellitus with neurological complications
C1083	Insulin dependent diabetes mellitus with multiple complications
C1084	Unstable insulin dependent diabetes mellitus
C1085	Insulin dependent diabetes mellitus with ulcer
C1086	Insulin dependent diabetes mellitus with gangrene
C1087	Insulin dependent diabetes mellitus with retinopathy
C1088	Insulin dependent diabetes mellitus - poor control
C1089	Insulin dependent diabetes maturity onset
C108A	Insulin-dependent diabetes without complication
C108B	Insulin dependent diabetes mellitus with mononeuropathy
C108C	Insulin dependent diabetes mellitus with polyneuropathy
C108D	Insulin dependent diabetes mellitus with nephropathy
C108E	Insulin dependent diabetes mellitus with hypoglycaemic coma
C108F	Insulin dependent diabetes mellitus with diabetic cataract
C108G	Insulin dependent diabetes mellitus with peripheral angiopathy
C108H	Insulin dependent diabetes mellitus with arthropathy
C108J	Insulin dependent diabetes mellitus with neuropathic arthropathy
C108y	Other specified diabetes mellitus with multiple complications
C108z	Unspecified diabetes mellitus with multiple complications
C109.	Non-insulin dependent diabetes mellitus
C1090	Non-insulin-dependent diabetes mellitus with renal complications
C1091	Non-insulin-dependent diabetes mellitus with ophthalmic complications
C1092	Non-insulin-dependent diabetes mellitus with neurological complications
C1093	Non-insulin-dependent diabetes mellitus with multiple complications
C1094	Non-insulin dependent diabetes mellitus with ulcer
C1095	Non-insulin dependent diabetes mellitus with gangrene
C1096	Non-insulin-dependent diabetes mellitus with retinopathy
C1097	Non-insulin dependent diabetes mellitus - poor control
C1099	Non-insulin-dependent diabetes mellitus without complication
C109A	Non-insulin dependent diabetes mellitus with mononeuropathy
C109B	Non-insulin dependent diabetes mellitus with polyneuropathy
C109C	Non-insulin dependent diabetes mellitus with nephropathy
C109D	Non-insulin dependent diabetes mellitus with hypoglycaemic coma
C109E	Non-insulin dependent diabetes mellitus with diabetic cataract
C109F	Non-insulin-dependent diabetes mellitus with peripheral angiopathy
C109G	Non-insulin dependent diabetes mellitus with arthropathy
C109H	Non-insulin dependent diabetes mellitus with neuropathic arthropathy

For peer review only

C109J	Insulin treated Type 2 diabetes mellitus
C109K	Hyperosmolar non-ketotic state in type 2 diabetes mellitus
C10E.	Type 1 diabetes mellitus
C10E0	Type 1 diabetes mellitus with renal complications
C10E1	Type 1 diabetes mellitus with ophthalmic complications
C10E2	Type 1 diabetes mellitus with neurological complications
C10E3	Type 1 diabetes mellitus with multiple complications
C10E4	Unstable type 1 diabetes mellitus
C10E5	Type 1 diabetes mellitus with ulcer
C10E6	Type 1 diabetes mellitus with gangrene
C10E7	Type 1 diabetes mellitus with retinopathy
C10E8	Type 1 diabetes mellitus - poor control
C10E9	Type 1 diabetes mellitus maturity onset
C10EA	Type 1 diabetes mellitus without complication
C10EB	Type 1 diabetes mellitus with mononeuropathy
C10EC	Type 1 diabetes mellitus with polyneuropathy
C10ED	Type 1 diabetes mellitus with nephropathy
C10EE	Type 1 diabetes mellitus with hypoglycaemic coma
C10EF	Type 1 diabetes mellitus with diabetic cataract
C10EG	Type 1 diabetes mellitus with peripheral angiopathy
C10EH	Type 1 diabetes mellitus with arthropathy
C10EJ	Type 1 diabetes mellitus with neuropathic arthropathy
C10EK	Type 1 diabetes mellitus with persistent proteinuria
C10EL	Type 1 diabetes mellitus with persistent microalbuminuria
C10EM	Type 1 diabetes mellitus with ketoacidosis
C10EN	Type 1 diabetes mellitus with ketoacidotic coma
C10EP	Type 1 diabetes mellitus with exudative maculopathy
C10EQ	Type 1 diabetes mellitus with gastroparesis
C10ER	Latent autoimmune diabetes mellitus in adult
C10F.	Type 2 diabetes mellitus
C10F0	Type 2 diabetes mellitus with renal complications
C10F1	Type 2 diabetes mellitus with ophthalmic complications
C10F2	Type 2 diabetes mellitus with neurological complications
C10F3	Type 2 diabetes mellitus with multiple complications
C10F4	Type 2 diabetes mellitus with ulcer
C10F5	Type 2 diabetes mellitus with gangrene
C10F6	Type 2 diabetes mellitus with retinopathy
C10F7	Type 2 diabetes mellitus - poor control
C10F9	Type 2 diabetes mellitus without complication
C10FA	Type 2 diabetes mellitus with mononeuropathy
C10FB	Type 2 diabetes mellitus with polyneuropathy
C10FC	Type 2 diabetes mellitus with nephropathy
C10FD	Type 2 diabetes mellitus with hypoglycaemic coma
C10FE	Type 2 diabetes mellitus with diabetic cataract
C10FF	Type 2 diabetes mellitus with peripheral angiopathy
C10FG	Type 2 diabetes mellitus with arthropathy
C10FH	Type 2 diabetes mellitus with neuropathic arthropathy
C10FJ	Insulin treated Type 2 diabetes mellitus

For peer review only

C10FK	Hyperosmolar non-ketotic state in type 2 diabetes mellitus
C10FL	Type 2 diabetes mellitus with persistent proteinuria
C10FM	Type 2 diabetes mellitus with persistent microalbuminuria
C10FN	Type 2 diabetes mellitus with ketoacidosis
C10FQ	Type 2 diabetes mellitus with exudative maculopathy
C10FR	Type 2 diabetes mellitus with gastroparesis
C10y.	Diabetes mellitus with other specified manifestation
C10y0	Diabetes mellitus, juvenile type, with other specified manifestation
C10y1	Diabetes mellitus, adult onset, with other specified manifestation
C10yy	Other specified diabetes mellitus with other specified complications
C10yz	Diabetes mellitus NOS with other specified manifestation
C10z.	Diabetes mellitus with unspecified complication
C10z0	Diabetes mellitus, juvenile type, with unspecified complication
C10z1	Diabetes mellitus, adult onset, with unspecified complication
C10zy	Other specified diabetes mellitus with unspecified complications
C10zz	Diabetes mellitus NOS with unspecified complication
L180.	Diabetes mellitus during pregnancy, childbirth and the puerperium
L1800	Diabetes mellitus - unspecified whether during pregnancy or the puerperium
L1801	Diabetes mellitus during pregnancy - baby delivered
L1802	Diabetes mellitus in the puerperium - baby delivered during current episode of care
L1803	Diabetes mellitus during pregnancy - baby not yet delivered
L1804	Diabetes mellitus in the pueperium - baby delivered during previous episode of care
L1805	Pre-existing diabetes mellitus, insulin-dependent
L1806	Pre-existing diabetes mellitus, non-insulin-dependent
L1808	Diabetes mellitus arising in pregnancy
L1809	Gestational diabetes mellitus
L180X	Pre-existing diabetes mellitus, unspecified
L180z	Diabetes mellitus during pregnancy, childbirth or the puerperium NOS

Hospital

ALT_CODE	DESCRIPTION
E10	Insulin-dependent diabetes mellitus
E10	Type 1 diabetes mellitus
E100	Insulin-dependent diabetes mellitus
E100	Type 1 diabetes mellitus
E101	Insulin-dependent diabetes mellitus
E101	Type 1 diabetes mellitus
E102	Insulin-dependent diabetes mellitus
E102	Type 1 diabetes mellitus
E103	Insulin-dependent diabetes mellitus
E103	Type 1 diabetes mellitus
E104	Insulin-dependent diabetes mellitus
E104	Type 1 diabetes mellitus

For peer review only

E105	Insulin-dependent diabetes mellitus
E105	Type 1 diabetes mellitus
E106	Insulin-dependent diabetes mellitus
E106	Type 1 diabetes mellitus
E107	Insulin-dependent diabetes mellitus
E107	Type 1 diabetes mellitus
E108	Insulin-dependent diabetes mellitus
E108	Type 1 diabetes mellitus
E109	Insulin-dependent diabetes mellitus
E109	Type 1 diabetes mellitus
E11	Non-insulin-dependent diabetes mellitus
E11	Type 2 diabetes mellitus
E110	Non-insulin-dependent diabetes mellitus
E110	Type 2 diabetes mellitus
E111	Non-insulin-dependent diabetes mellitus
E111	Type 2 diabetes mellitus
E112	Non-insulin-dependent diabetes mellitus
E112	Type 2 diabetes mellitus
E113	Non-insulin-dependent diabetes mellitus
E113	Type 2 diabetes mellitus
E114	Non-insulin-dependent diabetes mellitus
E114	Type 2 diabetes mellitus
E115	Non-insulin-dependent diabetes mellitus
E115	Type 2 diabetes mellitus
E116	Non-insulin-dependent diabetes mellitus
E116	Type 2 diabetes mellitus
E117	Non-insulin-dependent diabetes mellitus
E117	Type 2 diabetes mellitus
E118	Non-insulin-dependent diabetes mellitus
E118	Type 2 diabetes mellitus
E119	Non-insulin-dependent diabetes mellitus
E119	Type 2 diabetes mellitus
E12	Malnutrition-related diabetes mellitus
E120	Malnutrition-related diabetes mellitus
E121	Malnutrition-related diabetes mellitus
E122	Malnutrition-related diabetes mellitus
E123	Malnutrition-related diabetes mellitus
E124	Malnutrition-related diabetes mellitus
E125	Malnutrition-related diabetes mellitus
E126	Malnutrition-related diabetes mellitus
E127	Malnutrition-related diabetes mellitus
E128	Malnutrition-related diabetes mellitus
E129	Malnutrition-related diabetes mellitus
E13	Other specified diabetes mellitus
E130	Other specified diabetes mellitus
E131	Other specified diabetes mellitus
E132	Other specified diabetes mellitus
E133	Other specified diabetes mellitus

For peer review only

E134	Other specified diabetes mellitus
E135	Other specified diabetes mellitus
E136	Other specified diabetes mellitus
E137	Other specified diabetes mellitus
E138	Other specified diabetes mellitus
E139	Other specified diabetes mellitus
E14	Unspecified diabetes mellitus
E140	Unspecified diabetes mellitus
E141	Unspecified diabetes mellitus
E142	Unspecified diabetes mellitus
E143	Unspecified diabetes mellitus
E144	Unspecified diabetes mellitus
E145	Unspecified diabetes mellitus
E146	Unspecified diabetes mellitus
E147	Unspecified diabetes mellitus
E148	Unspecified diabetes mellitus
E149	Unspecified diabetes mellitus
O240	Diabetes mellitus in pregnancy: Pre-existing diabetes mellitus, insulin-dependent
O240	Diabetes mellitus in pregnancy: Pre-existing type 1 diabetes mellitus
O241	Diabetes mellitus in pregnancy: Pre-existing diabetes mellitus, non-insulin-dependent
O241	Diabetes mellitus in pregnancy: Pre-existing type 2 diabetes mellitus
O242	Diabetes mellitus in pregnancy: Pre-existing malnutrition-related diabetes mellitus
O243	Diabetes mellitus in pregnancy: Pre-existing diabetes mellitus, unspecified
O249	Diabetes mellitus in pregnancy, unspecified

Arthritis

READ_CODE	READ_DESC
N....	Musculoskeletal and connective tissue diseases
N0...	Arthropathies and related disorders
N00..	Diffuse diseases of connective tissue
N000.	Systemic lupus erythematosus
N0000	Disseminated lupus erythematosus
N0001	Libman-Sacks disease
N0002	Drug-induced systemic lupus erythematosus
N0003	Systemic lupus erythematosus with organ or system involvement
N0004	Systemic lupus erythematosus with pericarditis
N0005	Neonatal lupus erythematosus
N0006	Cerebral lupus
N000z	Systemic lupus erythematosus NOS
N001.	Scleroderma
N0010	Progressive systemic sclerosis
N0011	CREST syndrome
N0012	Systemic sclerosis induced by drugs and chemicals
N002.	Sicca (Sjogren's) syndrome
N003.	Dermatomyositis

For peer review only

N0030	Juvenile dermatomyositis
N0031	Dermatopolymyositis in neoplastic disease
N003X	Dermatopolymyositis, unspecified
N004.	Polymyositis
N005.	Adult Still's Disease
N006.	Antiphospholipid syndrome
N00y.	Other specified diffuse collagen diseases
N00y0	Eosinophilic fasciitis
N00y1	Fibrosclerosis systemic
N00z.	Collagen disease NOS
N01..	Arthropathy associated with infections
N010.	Pyogenic arthritis
N0100	Pyogenic arthritis of unspecified site
N0101	Pyogenic arthritis of the shoulder region
N0102	Pyogenic arthritis of the upper arm
N0103	Pyogenic arthritis of the forearm
N0104	Pyogenic arthritis of the hand
N0105	Pyogenic arthritis of the pelvic region and thigh
N0106	Pyogenic arthritis of the lower leg
N0107	Pyogenic arthritis of the ankle and foot
N0108	Staphylococcal arthritis and polyarthritis
N0109	Pneumococcal arthritis and polyarthritis
N010A	Arthritis in Lyme disease
N010x	Pyogenic arthritis of multiple sites
N010y	Pyogenic arthritis of other specified sites
N010z	Pyogenic arthritis NOS
N011.	Sexually acquired reactive arthropathy
N0110	Sexually acquired reactive arthropathy of unspecified site
N0111	Sexually acquired reactive arthropathy of the shoulder region
N0112	Sexually acquired reactive arthropathy of the upper arm
N0113	Sexually acquired reactive arthropathy of the forearm
N0114	Sexually acquired reactive arthropathy of the hand
N0115	Sexually acquired reactive arthropathy of the pelvic region and thigh
N0116	Sexually acquired reactive arthropathy of the lower leg
N0117	Sexually acquired reactive arthropathy of the ankle and foot
N011x	Sexually acquired reactive arthropathy of multiple sites
N011y	Sexually acquired reactive arthropathy of other specified site
N011z	Sexually acquired reactive arthropathy NOS
N012.	Arthropathy in Behcet's syndrome
N0120	Arthropathy in Behcet's syndrome of unspecified site
N0121	Arthropathy in Behcet's syndrome of the shoulder region
N0122	Arthropathy in Behcet's syndrome of the upper arm
N0123	Arthropathy in Behcet's syndrome of the forearm
N0124	Arthropathy in Behcet's syndrome of the hand
N0125	Arthropathy in Behcet's syndrome of the pelvic region and thigh
N0126	Arthropathy in Behcet's syndrome of the lower leg
N0127	Arthropathy in Behcet's syndrome of the ankle and foot
N012x	Arthropathy in Behcet's syndrome of multiple sites

N012y	Arthropathy in Behcet's syndrome of other specified sites
N012z	Arthropathy in Behcet's syndrome NOS
N013.	Postdysenteric reactive arthropathy
N0130	Postdysenteric reactive arthropathy of unspecified site
N0131	Postdysenteric reactive arthropathy of the shoulder region
N0132	Postdysenteric reactive arthropathy of the upper arm
N0133	Postdysenteric reactive arthropathy of the forearm
N0134	Postdysenteric reactive arthropathy of the hand
N0135	Postdysenteric reactive arthropathy of the pelvic region and thigh
N0136	Postdysenteric reactive arthropathy of the lower leg
N0137	Postdysenteric reactive arthropathy of the ankle and foot
N013x	Postdysenteric reactive arthropathy of multiple sites
N013y	Postdysenteric reactive arthropathy of other specified sites
N013z	Postdysenteric reactive arthropathy NOS
N014.	Arthropathy associated with other bacterial diseases
N0140	Arthropathy associated with other bacterial disease, of unspecified site
N0141	Arthropathy associated with other bacterial disease, of the shoulder region
N0142	Arthropathy associated with other bacterial disease, of the upper arm
N0143	Arthropathy associated with other bacterial disease, of the forearm
N0144	Arthropathy associated with other bacterial disease, of the hand
N0145	Arthropathy associated with other bacterial disease, of the pelvic region and thigh
N0146	Arthropathy associated with other bacterial disease, of the lower leg
N0147	Arthropathy associated with other bacterial disease, of the ankle and foot
N0148	Arthropathy in Whipple's disease
N014x	Arthropathy associated with other bacterial disease, of multiple sites
N014y	Arthropathy associated with other bacterial disease, of other specified site
N014z	Arthropathy associated with other bacterial disease NOS
N015.	Arthropathy associated with other viral diseases
N0150	Arthropathy associated with other viral disease, of unspecified site
N0151	Arthropathy associated with other viral disease, of the shoulder region
N0152	Arthropathy associated with other viral disease, of the upper arm
N0153	Arthropathy associated with other viral disease, of the forearm
N0154	Arthropathy with other viral disease, of hand
N0155	Arthropathy associated with other viral disease, of the pelvic region and thigh
N0156	Arthropathy associated with other viral disease, of the lower leg
N0157	Arthropathy associated with other viral disease, of the ankle and foot
N015x	Arthropathy associated with other viral disease, of multiple sites
N015y	Arthropathy associated with other viral disease, of other specified site
N015z	Arthropathy associated with other viral disease NOS
N016.	Arthropathy associated with mycoses
N0160	Arthropathy associated with mycoses, of unspecified site
N0161	Arthropathy associated with mycoses, of the shoulder region
N0162	Arthropathy associated with mycoses, of the upper arm
N0163	Arthropathy associated with mycoses, of the forearm
N0164	Arthropathy associated with mycoses, of the hand
N0165	Arthropathy associated with mycoses, of the pelvic region and thigh
N0166	Arthropathy associated with mycoses, of the lower leg
N0167	Arthropathy associated with mycoses, of the ankle and foot

For peer review only

N016x	Arthropathy associated with mycoses, of multiple sites
N016y	Arthropathy associated with mycoses, of other specified site
N016z	Arthropathy associated with mycoses NOS
N017.	Helminthiasis with arthropathy
N0170	Helminthiasis with arthropathy of unspecified site
N0171	Helminthiasis with arthropathy of the shoulder region
N0172	Helminthiasis with arthropathy of the upper arm
N0173	Helminthiasis with arthropathy of the forearm
N0174	Helminthiasis with arthropathy of the hand
N0175	Helminthiasis with arthropathy of the pelvic region and thigh
N0176	Helminthiasis with arthropathy of the lower leg
N0177	Helminthiasis with arthropathy of the ankle and foot
N017x	Helminthiasis with arthropathy of multiple sites
N017y	Helminthiasis with arthropathy of other specified site
N017z	Helminthiasis with arthropathy NOS
N018.	Tuberculous arthritis
N01w.	Reactive arthropathy, unspecified
N01w0	Reactive arthropathy of shoulder
N01w1	Reactive arthropathy of sternoclavicular joint
N01w2	Reactive arthropathy of acromioclavicular joint
N01w3	Reactive arthropathy of elbow
N01w4	Reactive arthropathy of distal radio-ulnar joint
N01w5	Reactive arthropathy of wrist
N01w6	Reactive arthropathy of metacarpophalangeal joint
N01w7	Reactive arthropathy of proximal interphalangeal joint of finger
N01w8	Reactive arthropathy of distal interphalangeal joint of finger
N01w9	Reactive arthropathy of hip
N01wA	Reactive arthropathy of sacro-iliac joint
N01wB	Reactive arthropathy of knee
N01wC	Reactive arthropathy of tibio-fibular joint
N01wD	Reactive arthropathy of ankle
N01wE	Reactive arthropathy of subtalar joint
N01wF	Reactive arthropathy of talonavicular joint
N01wG	Reactive arthropathy of other tarsal joint
N01wH	Reactive arthropathy of 1st metatarsophalangeal joint
N01wJ	Reactive arthropathy of lesser metatarsophalangeal joint
N01wK	Reactive arthropathy of interphalangeal joint of toe
N01y.	Other infectious and parasitic diseases with arthropathy
N01y0	Other infectious and parasitic diseases with arthropathy of unspecified site
N01y1	Other infectious and parasitic diseases with arthropathy of the shoulder region
N01y2	Other infectious and parasitic diseases with arthropathy of the upper arm
N01y3	Other infectious and parasitic diseases with arthropathy of the forearm
N01y4	Other infectious and parasitic diseases with arthropathy of the hand
N01y5	Other infectious and parasitic diseases with arthropathy of the pelvic region and thigh
N01y6	Other infectious and parasitic diseases with arthropathy of the lower leg
N01y7	Other infectious and parasitic diseases with arthropathy of the ankle and foot
N01yx	Other infectious and parasitic diseases with arthropathy of multiple sites

For peer review only

N01yy	Other infectious and parasitic diseases with arthropathy of other specified sites
N01yz	Other infectious and parasitic diseases with arthropathy NOS
N01z.	Infective arthritis NOS
N01z0	Infective arthritis NOS, of unspecified site
N01z1	Infective arthritis NOS, of the shoulder region
N01z2	Infective arthritis NOS, of the upper arm
N01z3	Infective arthritis NOS, of the forearm
N01z4	Infective arthritis NOS, of the hand
N01z5	Infective arthritis NOS, of the pelvic region and thigh
N01z6	Infective arthritis NOS, of the lower leg
N01z7	Infective arthritis NOS, of the ankle and foot
N01z8	Infective arthritis NOS, of shoulder
N01z9	Infective arthritis NOS, of sternoclavicular joint
N01zA	Infective arthritis NOS, of acromioclavicular joint
N01zB	Infective arthritis NOS, of elbow
N01zC	Infective arthritis NOS, of distal radio-ulnar joint
N01zD	Infective arthritis NOS, of wrist
N01zE	Infective arthritis NOS, of metacarpophalangeal joint
N01zF	Infective arthritis NOS, of proximal interphalangeal joint of finger
N01zG	Infective arthritis NOS, of distal interphalangeal joint of finger
N01zH	Infective arthritis NOS, of hip
N01zJ	Infective arthritis NOS, of sacro-iliac joint
N01zK	Infective arthritis NOS, of knee
N01zL	Infective arthritis NOS, of tibio-fibular joint
N01zM	Infective arthritis NOS, of ankle
N01zN	Infective arthritis NOS, of subtalar joint
N01zP	Infective arthritis NOS, of talonavicular joint
N01zQ	Infective arthritis NOS, of other tarsal joint
N01zR	Infective arthritis NOS, of 1st metatarsophalangeal joint
N01zS	Infective arthritis NOS, of lesser metatarsophalangeal joint
N01zT	Infective arthritis NOS, of interphalangeal joint of toe
N01zx	Infective arthritis NOS, of multiple sites
N01zy	Infective arthritis NOS, of other specified site
N01zz	Infective arthritis NOS
N02..	Crystal arthropathies
N020.	Chondrocalcinosis due to dicalcium phosphate crystals
N0200	Chondrocalcinosis due to dicalcium phosphate crystals, of unspecified site
N0201	Chondrocalcinosis due to dicalcium phosphate crystals, of the shoulder region
N0202	Chondrocalcinosis due to dicalcium phosphate crystals, of the upper arm
N0203	Chondrocalcinosis due to dicalcium phosphate crystals, of the forearm
N0204	Chondrocalcinosis due to dicalcium phosphate crystals, of the hand
N0205	Chondrocalcinosis due to dicalcium phosphate crystals, of the pelvic region and thigh
N0206	Chondrocalcinosis due to dicalcium phosphate crystals, of the lower leg
N0207	Chondrocalcinosis due to dicalcium phosphate crystals, of the ankle and foot
N020x	Chondrocalcinosis due to dicalcium phosphate crystals, of multiple sites
N020y	Chondrocalcinosis due to dicalcium phosphate crystals, of other specified site
N020z	Chondrocalcinosis due to dicalcium phosphate crystals, NOS

For peer review only

N021.	Chondrocalcinosis due to pyrophosphate crystals
N0210	Chondrocalcinosis due to pyrophosphate crystals, of unspecified site
N0211	Chondrocalcinosis due to pyrophosphate crystals, of the shoulder region
N0212	Chondrocalcinosis due to pyrophosphate crystals, of the upper arm
N0213	Chondrocalcinosis due to pyrophosphate crystals, of the forearm
N0214	Chondrocalcinosis-pyrophosphate crystals, of the hand
N0215	Chondrocalcinosis due to pyrophosphate crystals, of the pelvic region and thigh
N0216	Chondrocalcinosis due to pyrophosphate crystals, of the lower leg
N0217	Chondrocalcinosis due to pyrophosphate crystals, of the ankle and foot
N021x	Chondrocalcinosis due to pyrophosphate crystals, of multiple sites
N021y	Chondrocalcinosis due to pyrophosphate crystals, of other specified site
N021z	Chondrocalcinosis due to pyrophosphate crystals, NOS
N022.	Chondrocalcinosis, unspecified
N0220	Chondrocalcinosis unspecified, of unspecified site
N0221	Chondrocalcinosis unspecified, of the shoulder region
N0222	Chondrocalcinosis unspecified, of the upper arm
N0223	Chondrocalcinosis unspecified, of the forearm
N0224	Chondrocalcinosis unspecified, of the hand
N0225	Chondrocalcinosis unspecified, of the pelvic region and thigh
N0226	Chondrocalcinosis unspecified, of the lower leg
N0227	Chondrocalcinosis unspecified, of the ankle and foot
N022x	Chondrocalcinosis unspecified, of multiple sites
N022y	Chondrocalcinosis unspecified, of other specified site
N022z	Chondrocalcinosis NOS
N023.	Gouty arthritis
N0230	Gouty arthritis of unspecified site
N0231	Gouty arthritis of the shoulder region
N0232	Gouty arthritis of the upper arm
N0233	Gouty arthritis of the forearm
N0234	Gouty arthritis of the hand
N0235	Gouty arthritis of the pelvic region and thigh
N0236	Gouty arthritis of the lower leg
N0237	Gouty arthritis of the ankle and foot
N0238	Gouty arthritis of toe
N023x	Gouty arthritis of multiple sites
N023y	Gouty arthritis of other specified site
N023z	Gouty arthritis NOS
N024.	Familial chondrocalcinosis
N02y.	Other crystal arthropathies
N02y0	Other crystal arthropathies of unspecified site
N02y1	Other crystal arthropathies of the shoulder
N02y2	Other crystal arthropathies of the upper arm
N02y3	Other crystal arthropathies of the forearm
N02y4	Other crystal arthropathies of the hand
N02y5	Other crystal arthropathies of the pelvic region and thigh
N02y6	Other crystal arthropathies of the lower leg
N02y7	Other crystal arthropathies of the ankle and foot
N02y8	Hydroxyapatite deposition disease

For peer review only

N02yx	Other crystal arthropathies of multiple sites
N02yy	Other crystal arthropathies of other specified sites
N02yz	Other crystal arthropathy NOS
N02z.	Crystal arthropathy NOS
N02z0	Crystal arthropathy NOS, site unspecified
N02z1	Crystal arthropathy NOS, of the shoulder region
N02z2	Crystal arthropathy NOS, of the upper arm
N02z3	Crystal arthropathy NOS, of the forearm
N02z4	Crystal arthropathy NOS, of the hand
N02z5	Crystal arthropathy NOS, of the pelvic region and thigh
N02z6	Crystal arthropathy NOS, of the lower leg
N02z7	Crystal arthropathy NOS, of the ankle and foot
N02z8	Crystal arthropathy NOS, of shoulder
N02z9	Crystal arthropathy NOS, of sternoclavicular joint
N02zA	Crystal arthropathy NOS, of acromioclavicular joint
N02zB	Crystal arthropathy NOS, of elbow
N02zC	Crystal arthropathy NOS, of distal radio-ulnar joint
N02zD	Crystal arthropathy NOS, of wrist
N02zE	Crystal arthropathy NOS, of metacarpophalangeal joint
N02zF	Crystal arthropathy NOS, of proximal interphalangeal joint of finger
N02zG	Crystal arthropathy NOS, of distal interphalangeal joint of finger
N02zH	Crystal arthropathy NOS, of hip
N02zJ	Crystal arthropathy NOS, of sacro-iliac joint
N02zK	Crystal arthropathy NOS, of knee
N02zL	Crystal arthropathy NOS, of tibio-fibular joint
N02zM	Crystal arthropathy NOS, of ankle
N02zN	Crystal arthropathy NOS, of subtalar joint
N02zP	Crystal arthropathy NOS, of talonavicular joint
N02zQ	Crystal arthropathy NOS, of other tarsal joint
N02zR	Crystal arthropathy NOS, of 1st metatarsophalangeal joint
N02zS	Crystal arthropathy NOS, of lesser metatarsophalangeal joint
N02zT	Crystal arthropathy NOS, of interphalangeal joint of toe
N02zx	Crystal arthropathy NOS, of multiple sites
N02zy	Crystal arthropathy NOS, of other specified site
N02zz	Crystal arthropathy NOS
N03..	Arthropathy associated with disorders EC
N030.	Arthropathy associated with endocrine and metabolic disorder
N0300	Diabetic cheiroarthropathy
N0301	Diabetic Charcot arthropathy
N0302	Arthropathy in amyloidosis
N031.	Arthropathy associated with non-infective gastrointestinal disorders
N0310	Arthropathy in ulcerative colitis
N0311	Arthropathy in Crohn's disease
N0312	Arthropathy in Whipple's disease
N0313	Arthropathy following intestinal bypass
N032.	Arthropathy associated with haematological disorders
N0320	Arthropathy due to haemophilia
N033.	Arthropathy associated with dermatological disorders

For peer review only

N034.	Arthropathy associated with respiratory disorders
N035.	Neuropathic arthropathy
N036.	Arthropathy due to hypersensitivity reaction
N037.	Postimmunization arthropathy
N038.	Reactive arthropathies
N0380	Postmeningococcal arthritis
N0381	Postinfective arthropathy in syphilis
N039.	Beta-2 microglobulin arthropathy
N03x.	Other general diseases with associated arthropathy
N03x0	Arthritis associated with other disease, shoulder
N03x1	Arthritis associated with other disease, sternoclavicular joint
N03x2	Arthritis associated with other disease, acromioclavicular joint
N03x3	Arthritis associated with other disease, elbow
N03x4	Arthritis associated with other disease, distal radio-ulnar joint
N03x5	Arthritis associated with other disease, wrist
N03x6	Arthritis associated with other disease, metacarpophalangeal joint
N03x7	Arthritis associated with other disease, proximal interphalangeal joint of finger
N03x8	Arthritis associated with other disease, distal interphalangeal joint of finger
N03x9	Arthritis associated with other disease, hip
N03xA	Arthritis associated with other disease, sacro-iliac joint
N03xB	Arthritis associated with other disease, knee
N03xC	Arthritis associated with other disease, tibio-fibular joint
N03xD	Arthritis associated with other disease, ankle
N03xE	Arthritis associated with other disease, subtalar joint
N03xF	Arthritis associated with other disease, talonavicular joint
N03xG	Arthritis associated with other disease, other tarsal joint
N03xH	Arthritis associated with other disease, 1st metatarsophalangeal joint
N03xJ	Arthritis associated with other disease, lesser metatarsophalangeal joint
N03xK	Arthritis associated with other disease, interphalangeal joint of toe
N03y.	Arthropathy associated with other conditions EC
N03z.	Arthropathy associated with disorders EC NOS
N04..	Rheumatoid arthritis and other inflammatory polyarthropathies
N040.	Rheumatoid arthritis
N0400	Rheumatoid arthritis of cervical spine
N0401	Other rheumatoid arthritis of spine
N0402	Rheumatoid arthritis of shoulder
N0403	Rheumatoid arthritis of sternoclavicular joint
N0404	Rheumatoid arthritis of acromioclavicular joint
N0405	Rheumatoid arthritis of elbow
N0406	Rheumatoid arthritis of distal radio-ulnar joint
N0407	Rheumatoid arthritis of wrist
N0408	Rheumatoid arthritis of metacarpophalangeal joint
N0409	Rheumatoid arthritis of proximal interphalangeal joint of finger
N040A	Rheumatoid arthritis of distal interphalangeal joint of finger
N040B	Rheumatoid arthritis of hip
N040C	Rheumatoid arthritis of sacro-iliac joint
N040D	Rheumatoid arthritis of knee
N040E	Rheumatoid arthritis of tibio-fibular joint

For peer review only

N040F	Rheumatoid arthritis of ankle
N040G	Rheumatoid arthritis of subtalar joint
N040H	Rheumatoid arthritis of talonavicular joint
N040J	Rheumatoid arthritis of other tarsal joint
N040K	Rheumatoid arthritis of 1st metatarsophalangeal joint
N040L	Rheumatoid arthritis of lesser metatarsophalangeal joint
N040M	Rheumatoid arthritis of interphalangeal joint of toe
N040N	Rheumatoid vasculitis
N040P	Seronegative rheumatoid arthritis
N040Q	Rheumatoid bursitis
N040R	Rheumatoid nodule
N040S	Rheumatoid arthritis - multiple joint
N040T	Flare of rheumatoid arthritis
N041.	Felty's syndrome
N042.	Other rheumatoid arthropathy with visceral or systemic involvement
N0420	Rheumatic carditis
N0421	Rheumatoid lung disease
N0422	Rheumatoid nodule
N042z	Rheumatoid arthropathy with visceral or systemic involvement NOS
N043.	Juvenile rheumatoid arthritis - Still's disease
N0430	Juvenile rheumatoid arthropathy unspecified
N0431	Acute polyarticular juvenile rheumatoid arthritis
N0432	Pauciarticular juvenile rheumatoid arthritis
N0433	Monarticular juvenile rheumatoid arthritis
N043z	Juvenile rheumatoid arthritis NOS
N044.	Chronic post-rheumatic arthropathy
N045.	Other juvenile arthritis
N0450	Juvenile ankylosing spondylitis
N0451	Juvenile seronegative polyarthritis
N0452	Juvenile arthritis in psoriasis
N0453	Juvenile arthritis in Crohn's disease
N0454	Juvenile arthritis in ulcerative colitis
N0455	Juvenile rheumatoid arthritis
N0456	Pauciarticular onset juvenile chronic arthritis
N047.	Seropositive errosive rheumatoid arthritis
N04X.	Seropositive rheumatoid arthritis, unspecified
N04y.	Other specified inflammatory polyarthropathy
N04y0	Rheumatoid lung
N04y1	Sero negative arthritis
N04y2	Adult-onset Still's disease
N04y3	Remitting seronegative symmetrical synovitis with pitting oedema
N04yz	Other specified inflammatory polyarthropathy NOS
N04z.	Inflammatory polyarthropathy NOS
N05..	Osteoarthritis and allied disorders
N050.	Generalised osteoarthritis - OA
N0500	Generalised osteoarthritis of unspecified site
N0501	Generalised osteoarthritis of the hand
N0502	Generalised osteoarthritis of multiple sites

For peer review only

N0503	Bouchard's nodes with arthropathy
N0504	Primary generalized osteoarthrosis
N0505	Secondary multiple arthrosis
N0506	Erosive osteoarthrosis
N0507	Heberden's nodes with arthropathy
N050z	Generalised osteoarthritis NOS
N051.	Localised, primary osteoarthritis
N0510	Localised, primary osteoarthritis of unspecified site
N0511	Localised, primary osteoarthritis of the shoulder region
N0512	Localised, primary osteoarthritis of the upper arm
N0513	Localised, primary osteoarthritis of the forearm
N0514	Localised, primary osteoarthritis of the hand
N0515	Localised, primary osteoarthritis of the pelvic region and thigh
N0516	Localised, primary osteoarthritis of the lower leg
N0517	Localised, primary osteoarthritis of the ankle and foot
N0518	Localised, primary osteoarthritis of other specified site
N0519	Primary coxarthrosis, bilateral
N051A	Coxarthrosis resulting from dysplasia, bilateral
N051B	Primary gonarthrosis, bilateral
N051C	Primary arthrosis of first carpometacarpal joints, bilateral
N051D	Localised, primary osteoarthritis of the wrist
N051E	Localised, primary osteoarthritis of toe
N051F	Localised, primary osteoarthritis of elbow
N051G	Osteoarthritis of spinal facet joint
N051z	Localised, primary osteoarthritis NOS
N052.	Localised, secondary osteoarthritis
N0520	Localised, secondary osteoarthritis of unspecified site
N0521	Localised, secondary osteoarthritis of the shoulder region
N0522	Localised, secondary osteoarthritis of the upper arm
N0523	Localised, secondary osteoarthritis of the forearm
N0524	Localised, secondary osteoarthritis of the hand
N0525	Localised, secondary osteoarthritis of the pelvic region and thigh
N0526	Localised, secondary osteoarthritis of the lower leg
N0527	Localised, secondary osteoarthritis of the ankle and foot
N0528	Localised, secondary osteoarthritis of other specified site
N0529	Post-traumatic coxarthrosis, bilateral
N052A	Post-traumatic gonarthrosis, bilateral
N052B	Post-traumatic arthrosis of first carpometacarpal joints, bilateral
N052C	Post-traumatic gonarthrosis, unilateral
N052z	Localised, secondary osteoarthritis NOS
N053.	Localised osteoarthritis, unspecified
N0530	Localised osteoarthritis, unspecified, of unspecified site
N0531	Localised osteoarthritis, unspecified, of the shoulder region
N0532	Localised osteoarthritis, unspecified, of the upper arm
N0533	Localised osteoarthritis, unspecified, of the forearm
N0534	Localised osteoarthritis, unspecified, of the hand
N0535	Localised osteoarthritis, unspecified, of the pelvic region and thigh
N0536	Localised osteoarthritis, unspecified, of the lower leg

For peer review only

N0537	Localised osteoarthritis, unspecified, of the ankle and foot
N0538	Localised osteoarthritis, unspecified, of other specified site
N0539	Arthrosis of first carpometacarpal joint, unspecified
N053z	Localised osteoarthritis, unspecified, NOS
N054.	Oligoarticular osteoarthritis, unspecified
N0540	Oligoarticular osteoarthritis, unspecified, of unspecified sites
N0541	Oligoarticular osteoarthritis, unspecified, of the shoulder region
N0542	Oligoarticular osteoarthritis, unspecified, of upper arm
N0543	Oligoarticular osteoarthritis, unspecified, of forearm
N0544	Oligoarticular osteoarthritis, unspecified, of hand
N0545	Oligoarticular osteoarthritis, unspecified, of the pelvic region and thigh
N0546	Oligoarticular osteoarthritis, unspecified, of lower leg
N0547	Oligoarticular osteoarthritis, unspecified, of ankle and foot
N0548	Oligoarticular osteoarthritis, unspecified, of other specified sites
N0549	Oligoarticular osteoarthritis, unspecified, of multiple sites
N054z	Osteoarthritis of more than one site, unspecified, NOS
N05z.	Osteoarthritis NOS
N05z0	Osteoarthritis NOS, of unspecified site
N05z1	Osteoarthritis NOS, of shoulder region
N05z2	Osteoarthritis NOS, of the upper arm
N05z3	Osteoarthritis NOS, of the forearm
N05z4	Osteoarthritis NOS, of the hand
N05z5	Osteoarthritis NOS, pelvic region/thigh
N05z6	Osteoarthritis NOS, of the lower leg
N05z7	Osteoarthritis NOS, of ankle and foot
N05z8	Osteoarthritis NOS, other specified site
N05z9	Osteoarthritis NOS, of shoulder
N05zA	Osteoarthritis NOS, of sternoclavicular joint
N05zB	Osteoarthritis NOS, of acromioclavicular joint
N05zC	Osteoarthritis NOS, of elbow
N05zD	Osteoarthritis NOS, of distal radio-ulnar joint
N05zE	Osteoarthritis NOS, of wrist
N05zF	Osteoarthritis NOS, of metacarpophalangeal joint
N05zG	Osteoarthritis NOS, of proximal interphalangeal joint of finger
N05zH	Osteoarthritis NOS, of distal interphalangeal joint of finger
N05zJ	Osteoarthritis NOS, of hip
N05zK	Osteoarthritis NOS, of sacro-iliac joint
N05zL	Osteoarthritis NOS, of knee
N05zM	Osteoarthritis NOS, of tibio-fibular joint
N05zN	Osteoarthritis NOS, of ankle
N05zP	Osteoarthritis NOS, of subtalar joint
N05zQ	Osteoarthritis NOS, of talonavicular joint
N05zR	Osteoarthritis NOS, of other tarsal joint
N05zS	Osteoarthritis NOS, of 1st metatarsophalangeal joint
N05zT	Osteoarthritis NOS, of lesser metatarsophalangeal joint
N05zU	Osteoarthritis NOS, of interphalangeal joint of toe
N05zz	Osteoarthritis NOS
N06..	Other and unspecified arthropathies

N060.	Kaschin - Beck disease
N0600	Kaschin-Beck disease of unspecified site
N0601	Kaschin-Beck disease of the shoulder region
N0602	Kaschin-Beck disease of the upper arm
N0603	Kaschin-Beck disease of the forearm
N0604	Kaschin-Beck disease of the hand
N0605	Kaschin-Beck disease of the pelvic region and thigh
N0606	Kaschin-Beck disease of the lower leg
N0607	Kaschin-Beck disease of the ankle and foot
N0608	Kaschin-Beck disease of other specified site
N0609	Kaschin-Beck disease of multiple sites
N060z	Kaschin-Beck disease NOS
N061.	Traumatic arthropathy
N0610	Traumatic arthropathy of unspecified site
N0611	Traumatic arthropathy of the shoulder region
N0612	Traumatic arthropathy of the upper arm
N0613	Traumatic arthropathy of the forearm
N0614	Traumatic arthropathy of the hand
N0615	Traumatic arthropathy of the pelvic region and thigh
N0616	Traumatic arthropathy of the lower leg
N0617	Traumatic arthropathy of the ankle and foot
N0618	Traumatic arthropathy of other specified site
N0619	Traumatic arthropathy of multiple sites
N061A	Traumatic arthropathy of shoulder
N061B	Traumatic arthropathy of sternoclavicular joint
N061C	Traumatic arthropathy of acromioclavicular joint
N061D	Traumatic arthropathy-elbow
N061E	Traumatic arthropathy of distal radio-ulnar joint
N061F	Traumatic arthropathy-wrist
N061G	Traumatic arthropathy of metacarpophalangeal joint
N061H	Traumatic arthropathy of proximal interphalangeal joint of finger
N061J	Traumatic arthropathy of distal interphalangeal joint of finger
N061K	Traumatic arthropathy-hip
N061L	Traumatic arthropathy of sacro-iliac joint
N061M	Traumatic arthropathy-knee
N061N	Traumatic arthropathy of tibio-fibular joint
N061P	Traumatic arthropathy-ankle
N061Q	Traumatic arthropathy of subtalar joint
N061R	Traumatic arthropathy of talonavicular joint
N061S	Traumatic arthropathy of other tarsal joint
N061T	Traumatic arthropathy of 1st metatarsophalangeal joint
N061U	Traumatic arthropathy of lesser metatarsophalangeal joint
N061V	Traumatic arthropathy of interphalangeal joint of toe
N061z	Traumatic arthropathy NOS
N062.	Allergic arthritis
N0620	Allergic arthritis of unspecified site
N0621	Allergic arthritis of the shoulder region
N0622	Allergic arthritis of the upper arm

For peer review only

N0623	Allergic arthritis of the forearm
N0624	Allergic arthritis of the hand
N0625	Allergic arthritis of the pelvic region and thigh
N0626	Allergic arthritis of the lower leg
N0627	Allergic arthritis of the ankle and foot
N0628	Allergic arthritis of other specified site
N0629	Allergic arthritis of multiple sites
N062z	Allergic arthritis NOS
N063.	Climacteric arthritis
N0630	Climacteric arthritis of unspecified site
N0631	Climacteric arthritis of the shoulder region
N0632	Climacteric arthritis of the upper arm
N0633	Climacteric arthritis of the forearm
N0634	Climacteric arthritis of the hand
N0635	Climacteric arthritis of the pelvic region and thigh
N0636	Climacteric arthritis of the lower leg
N0637	Climacteric arthritis of the ankle and foot
N0638	Climacteric arthritis of other specified site
N0639	Climacteric arthritis of multiple sites
N063z	Climacteric arthritis NOS
N064.	Transient arthropathy
N0640	Transient arthropathy of unspecified site
N0641	Transient arthropathy of the shoulder region
N0642	Transient arthropathy of the upper arm
N0643	Transient arthropathy of the forearm
N0644	Transient arthropathy of the hand
N0645	Transient arthropathy of the pelvic region and thigh
N0646	Transient arthropathy of the lower leg
N0647	Transient arthropathy of the ankle and foot
N0648	Transient arthropathy of other specified site
N0649	Transient arthropathy of multiple sites
N064A	Transient arthropathy of shoulder
N064B	Transient arthropathy of sternoclavicular joint
N064C	Transient arthropathy of acromioclavicular joint
N064D	Transient arthropathy-elbow
N064E	Transient arthropathy of distal radio-ulnar joint
N064F	Transient arthropathy-wrist
N064G	Transient arthropathy of metacarpophalangeal joint
N064H	Transient arthropathy of proximal interphalangeal joint of finger
N064J	Transient arthropathy of distal interphalangeal joint of finger
N064K	Transient arthropathy-hip
N064L	Transient arthropathy of sacro-iliac joint
N064M	Transient arthropathy-knee
N064N	Transient arthropathy of tibio-fibular joint
N064P	Transient arthropathy-ankle
N064Q	Transient arthropathy of subtalar joint
N064R	Transient arthropathy of talonavicular joint
N064S	Transient arthropathy of other tarsal joint

For peer review only

N064T	Transient arthropathy of 1st metatarsophalangeal joint
N064U	Transient arthropathy of lesser metatarsophalangeal joint
N064V	Transient arthropathy of interphalangeal joint of toe
N064z	Transient arthropathy NOS
N065.	Unspecified polyarthropathy or polyarthritis
N0650	Unspecified polyarthropathy of unspecified site
N0651	Unspecified polyarthropathy of the shoulder region
N0652	Unspecified polyarthropathy of the upper arm
N0653	Unspecified polyarthropathy of the forearm
N0654	Unspecified polyarthropathy of the hand
N0655	Unspecified polyarthropathy of the pelvic region and thigh
N0656	Unspecified polyarthropathy of the lower leg
N0657	Unspecified polyarthropathy of the ankle and foot
N0658	Unspecified polyarthropathy of other specified site
N0659	Unspecified polyarthropathy of multiple sites
N065A	Generalised arthritis
N065z	Unspecified polyarthropathy or polyarthritis NOS
N066.	Unspecified monoarthritis
N0660	Unspecified monoarthritis of unspecified site
N0661	Unspecified monoarthritis of the shoulder region
N0662	Unspecified monoarthritis of the upper arm
N0663	Unspecified monoarthritis of the forearm
N0664	Unspecified monoarthritis of the hand
N0665	Unspecified monoarthritis of the pelvic region and thigh
N0666	Unspecified monoarthritis of the lower leg
N0667	Unspecified monoarthritis of the ankle and foot
N0668	Unspecified monoarthritis of other specified site
N066z	Unspecified monoarthritis NOS
N067.	Ochronotic arthropathy
N068.	Haemophilic arthropathy
N069.	Arthropathy in neoplastic disease
N06y.	Other specified arthropathy
N06y0	Other specified arthropathy of unspecified site
N06y1	Other specified arthropathy of the shoulder region
N06y2	Other specified arthropathy of the upper arm
N06y3	Other specified arthropathy of the forearm
N06y4	Other specified arthropathy of the hand
N06y5	Other specified arthropathy of the pelvic region and thigh
N06y6	Other specified arthropathy of the lower leg
N06y7	Other specified arthropathy of the ankle and foot
N06y8	Other specified arthropathy of other specified site
N06y9	Other specified arthropathy of multiple sites
N06yz	Other specified arthropathy NOS
N06z.	Arthropathy NOS
N06z0	Arthropathy NOS, of unspecified site
N06z1	Arthropathy NOS, of the shoulder region
N06z2	Arthropathy NOS, of the upper arm
N06z3	Arthropathy NOS, of the forearm

For peer review only

N06z4	Arthropathy NOS, of the hand
N06z5	Arthropathy NOS, of the pelvic region and thigh
N06z6	Arthropathy NOS, of the lower leg
N06z7	Arthropathy NOS, of the ankle and foot
N06z8	Arthropathy NOS, of other specified site
N06z9	Arthropathy NOS, of multiple sites
N06zA	Acute arthritis
N06zB	Chronic arthritis
N06zz	Arthropathy NOS
N07..	Internal derangement of knee
N070.	Medial meniscus derangement
N0700	Medial meniscus derangement, unspecified
N0701	Old bucket handle tear of medial meniscus
N0702	Medial meniscus, anterior horn derangement
N0703	Medial meniscus, posterior horn derangement
N0704	Parrot beak tear of posterior horn of medial meniscus
N0705	Peripheral detachment of medial meniscus
N0706	Radial tear of medial meniscus
N0707	Horizontal cleavage tear of medial meniscus
N0708	Multiple tears of medial meniscus
N0709	Cyst of medial meniscus
N070A	Old tear of medial meniscus
N070B	Old tear of posterior horn of medial meniscus
N070z	Medial meniscus derangement NOS
N071.	Lateral meniscus derangement
N0710	Lateral meniscus derangement unspecified
N0711	Old bucket handle tear of lateral meniscus
N0712	Lateral meniscus, anterior horn derangement
N0713	Lateral meniscus, posterior horn derangement
N0714	Lateral meniscus derangement NOS
N0715	Parrot beak tear of posterior horn of lateral meniscus
N0716	Peripheral detachment of lateral meniscus
N0717	Radial tear of lateral meniscus
N0718	Horizontal cleavage tear of lateral meniscus
N0719	Multiple tears of lateral meniscus
N071A	Cyst of lateral meniscus
N071B	Discoid lateral meniscus
N071C	Old tear of lateral meniscus
N072.	Meniscus derangement NEC
N0720	Old torn meniscus of knee
N0721	Degenerative lesion of articular cartilage of knee
N0722	Cyst of semilunar cartilage
N073.	Loose body in knee
N074.	Chondromalacia patellae
N07y.	Other internal knee derangement
N07y0	Old lateral collateral ligament disruption
N07y1	Old medial collateral ligament disruption
N07y2	Old anterior cruciate ligament disruption

For peer review only

N07y3	Old posterior cruciate ligament disruption
N07y4	Old capsular knee ligament disruption
N07y5	Locked knee
N07y6	Patellofemoral maltracking
N07y7	Old partial tear lateral collateral ligament
N07y8	Old complete tear lateral collateral ligament
N07y9	Old posterolateral capsular complex tear
N07yA	Old partial tear medial collateral ligament
N07yB	Old complete tear medial collateral ligament
N07yC	Old medial capsular complex tear
N07yD	Old partial tear anterior cruciate ligament
N07yE	Old complete tear anterior cruciate ligament
N07yF	Old partial tear posterior cruciate ligament
N07yG	Old complete tear posterior cruciate ligament
N07yH	Locking knee
N07yJ	Adhesions of knee joint
N07yy	Other old knee ligament disruption
N07yz	Other internal knee derangement NOS
N07z.	Internal knee derangement NOS
N08..	Other derangement of joint
N080.	Articular cartilage disorder, excluding the knee
N0800	Articular cartilage disorder, of unspecified site
N0801	Articular cartilage disorder of the shoulder region
N0802	Articular cartilage disorder of the upper arm
N0803	Articular cartilage disorder of the forearm
N0804	Articular cartilage disorder of the hand
N0805	Articular cartilage disorder of the pelvic region and thigh
N0806	Articular cartilage disorder of the ankle and foot
N0807	Articular cartilage disorder of other specified site
N0808	Articular cartilage disorder of multiple sites
N0809	Hill-Sachs lesion
N080A	Reverse Hill-Sachs lesion
N080B	Articular cartilage disorder of other joints of the shoulder girdle
N080C	Chondrolysis of the femoral head
N080z	Articular cartilage disorder NOS
N081.	Loose body in joint, excluding the knee
N0810	Loose body in joint, unspecified joint
N0811	Loose body in joint of shoulder region
N0812	Loose body in joint of upper arm
N0813	Loose body in joint of forearm
N0814	Loose body in joint of hand
N0815	Loose body in joint of pelvic region and thigh
N0816	Loose body in joint of ankle and foot
N0817	Loose body in joint, other specified joint
N0818	Loose joint body in multiple joints
N0819	Loose body in shoulder joint
N081A	Loose body in other joint of shoulder girdle
N081B	Loose body in elbow joint

For peer review only

N081C	Loose body in wrist joint
N081D	Loose body in hip joint
N081E	Loose body in other joint of pelvic girdle
N081F	Loose body in ankle joint
N081G	Loose body in foot joint
N081z	Loose body in joint (excluding the knee) NOS
N082.	Pathological dislocation
N0820	Pathological dislocation of unspecified site
N0821	Pathological dislocation of the shoulder region
N0822	Pathological dislocation of the upper arm
N0823	Pathological dislocation of the forearm
N0824	Pathological dislocation of the hand
N0825	Pathological dislocation of the pelvic region and thigh
N0826	Pathological dislocation of the lower leg
N0827	Pathological dislocation of the ankle and foot
N0828	Pathological dislocation of other specified site
N0829	Pathological dislocation of multiple joints
N082A	Pathological dislocation of the shoulder joint
N082B	Pathological dislocation of other joint of shoulder girdle
N082C	Pathological dislocation of humero-ulnar joint
N082D	Pathological dislocation of superior radio-ulnar joint
N082E	Pathological dislocation of radial head
N082F	Pathological dislocation of inferior radio-ulnar joint
N082G	Pathological dislocation of wrist joint
N082H	Pathological dislocation of 1st carpometacarpal joint
N082J	Pathological dislocation of other carpometacarpal joint
N082K	Pathological dislocation of metacarpophalangeal joint
N082L	Pathological dislocation of proximal interphalangeal joint
N082M	Pathological dislocation of distal interphalangeal joint
N082N	Neuromuscular dislocation of the hip
N082P	Other acquired pathological dislocation of the hip
N082Q	Pathological dislocation of knee joint
N082R	Pathological dislocation of patellofemoral joint
N082S	Pathological dislocation of ankle joint
N082T	Pathological dislocation of subtalar joint
N082U	Pathological dislocation of midtarsal joint
N082V	Pathological dislocation of tarsometatarsal joint
N082W	Pathological dislocation of 1st metatarsophalangeal joint
N082X	Pathological dislocation of lesser metatarsophalangeal joint
N082Y	Pathological dislocation of toe interphalangeal joint
N082Z	Non-traumatic subluxation of acromioclavicular joint
N082z	Pathological dislocation NOS
N083.	Recurrent dislocation of joint
N0830	Recurrent joint dislocation, of unspecified site
N0831	Recurrent joint dislocation, of the shoulder region
N0832	Recurrent joint dislocation, of the upper arm
N0833	Recurrent joint dislocation, of the forearm
N0834	Recurrent joint dislocation, of the hand

For peer review only

N0835	Recurrent joint dislocation, of the pelvic region and thigh
N0836	Recurrent joint dislocation, of the lower leg
N0837	Recurrent joint dislocation, of the ankle and foot
N0838	Recurrent joint dislocation, of other specified site
N0839	Recurrent dislocation of multiple joints
N083A	Recurrent dislocation of shoulder - anterior
N083B	Recurrent dislocation of shoulder - posterior
N083C	Recurrent subluxation of shoulder - anterior
N083D	Recurrent subluxation of shoulder - posterior
N083E	Recurrent dislocation of shoulder - inferior
N083F	Recurrent subluxation of shoulder - inferior
N083G	Recurrent dislocation of shoulder - anterior
N083H	Recurrent subluxation of shoulder - anterior
N083J	Recurrent dislocation of shoulder - multidirectional
N083K	Recurrent subluxation of shoulder - multidirectional
N083L	Habitual dislocation of the shoulder
N083M	Habitual subluxation of the shoulder
N083N	Recurrent dislocation of elbow
N083P	Recurrent subluxation of elbow
N083Q	Recurrent dislocation of superior radio-ulnar joint
N083R	Recurrent subluxation of superior radio-ulnar joint
N083S	Recurrent dislocation of radial head
N083T	Recurrent subluxation of radial head
N083U	Recurrent dislocation of inferior radio-ulnar joint
N083V	Recurrent subluxation of inferior radio-ulnar joint
N083W	Recurrent dislocation of wrist
N083X	Carpal instability
N083Y	Recurrent subluxation of wrist
N083Z	Carpal instability, dorsal intercalated segment instability
N083a	Carpal instability, ventral intercalated segment instability
N083b	Carpal instability, ulnar translocation
N083c	Carpal instability, dorsal subluxation
N083d	Carpal instability, other
N083e	Recurrent dislocation of carpometacarpal joint
N083f	Recurrent subluxation of carpometacarpal joint
N083g	Recurrent dislocation of metacarpophalangeal joint
N083h	Recurrent subluxation of metacarpophalangeal joint
N083j	Recurrent dislocation of interphalangeal joint
N083k	Recurrent subluxation of interphalangeal joint
N083l	Recurrent dislocation of the hip
N083m	Recurrent dislocation of other pelvic joints
N083n	Recurrent dislocation of the knee
N083p	Recurrent dislocation of the patella
N083q	Recurrent subluxation of the patella
N083r	Habitual dislocation of the patella
N083s	Recurrent dislocation of the ankle
N083t	Recurrent subluxation of the ankle
N083u	Recurrent dislocation of foot joint

For peer review only

N083v	Recurrent subluxation of the subtalar joint
N083w	Recurrent subluxation of other foot joint
N083x	Recurrent subluxation of the hip joint
N083z	Recurrent joint dislocation NOS
N084.	Contracture of joint
N0840	Joint contracture of unspecified site
N0841	Joint contracture of the shoulder region
N0842	Joint contracture of the upper arm
N0843	Joint contracture of the forearm
N0844	Joint contracture of the hand
N0845	Joint contracture of the pelvic region and thigh
N0846	Joint contracture of the lower leg
N0847	Joint contracture of the ankle and foot
N0848	Joint contracture of other specified site
N0849	Contracture of multiple joints
N084A	Flexion contracture-shoulder
N084B	Extension contracture-shoulder
N084C	Abduction contracture-shoulder
N084D	Adduction contracture-shoulder
N084E	Internal rotation contracture-shoulder
N084F	External rotation contracture-shoulder
N084G	Flexion contracture - elbow
N084H	Extension contracture - elbow
N084J	Pronation contracture - forearm
N084K	Supination contracture - forearm
N084L	Flexion contracture - wrist
N084M	Extension contracture of the wrist
N084N	Ulnar deviation contracture of the wrist
N084P	Radial deviation contracture of the wrist
N084Q	Flexion contracture of metacarpophalangeal joint
N084R	Extension contracture of metacarpophalangeal joint
N084S	Flexion contracture of proximal interphalangeal joint
N084T	Flexion contracture of distal interphalangeal joint
N084U	Flexion contracture of hip
N084V	Extension contracture of hip
N084W	Abduction contracture of hip
N084X	Adduction contracture of hip
N084Y	Internal rotation contracture of hip
N084Z	External rotation contracture of hip
N084a	Flexion contracture of the knee
N084b	Equinus contracture of the ankle
N084c	Calcaneus contracture of the ankle
N084d	Flexion contracture of metatarsophalangeal joint
N084e	Extension contracture of metatarsophalangeal joint
N084f	Flexion contracture of toe interphalangeal joint
N084g	Extension contracture of toe interphalangeal joint
N084z	Contracture of joint NOS
N085.	Ankylosis of joint

For peer review only

N0850	Joint ankylosis of unspecified site
N0851	Joint ankylosis of the shoulder region
N0852	Joint ankylosis of the upper arm
N0853	Joint ankylosis of the forearm
N0854	Joint ankylosis of the hand
N0855	Joint ankylosis of the pelvic region and thigh
N0856	Joint ankylosis of the lower leg
N0857	Joint ankylosis of the ankle and foot
N0858	Joint ankylosis of other specified site
N0859	Ankylosis of multiple joints
N085A	Ankylosis of the shoulder joint
N085B	Ankylosis of other joint of the shoulder girdle
N085C	Ankylosis of the elbow joint
N085D	Ankylosis of the superior radio-ulnar joint
N085E	Ankylosis of the inferior radio-ulnar joint
N085F	Ankylosis of the wrist joint
N085G	Ankylosis of the 1st carpometacarpal joint
N085H	Ankylosis of other carpometacarpal joint
N085J	Ankylosis of metacarpophalangeal joint
N085K	Ankylosis of proximal interphalangeal joint
N085L	Ankylosis of distal interphalangeal joint
N085M	Ankylosis of the hip joint
N085N	Ankylosis of other pelvic joint
N085P	Ankylosis of the knee joint
N085Q	Ankylosis of the ankle joint
N085R	Ankylosis of the subtalar joint
N085S	Ankylosis of other tarsal joint
N085T	Ankylosis of metatarsophalangeal joint
N085U	Ankylosis of toe joint
N085z	Ankylosis of joint NOS
N086.	Unspecified intrapelvic protrusio acetabuli
N0860	Protrusio acetabuli of unspecified site
N0861	Protrusio acetabuli of the pelvic region and thigh
N086z	Protrusio acetabuli NOS
N087.	Fibrocartilage lesion of joint
N0870	Bankart lesion
N0871	Reverse Bankart lesion
N0872	Glenoid labrum detachment
N0873	Glenoid labrum tear
N0874	Triangular fibrocartilage tear
N0875	Triangular fibrocartilage detachment
N0876	Acetabular labrum detachment
N0877	Acetabular labrum tear
N0878	Snapping shoulder
N08y.	Other joint derangement NEC
N08y0	Other joint derangement NEC, of unspecified site
N08y1	Other joint derangement NEC, of the shoulder region
N08y2	Other joint derangement NEC, of the upper arm

For peer review only

N08y3	Other joint derangement NEC, of the forearm
N08y4	Other joint derangement NEC, of the hand
N08y5	Other joint derangement NEC, of the pelvic region and thigh
N08y6	Other joint derangement NEC, of the lower leg
N08y7	Other joint derangement NEC, of the ankle and foot
N08y8	Other joint derangement NEC, of other specified site
N08y9	Other joint derangement NEC, of multiple sites
N08yA	Flail joint
N08yB	Shoulder joint unstable
N08yz	Other joint derangement NEC, NOS
N08z.	Joint derangement NOS
N08z0	Joint derangement NOS, of unspecified site
N08z1	Joint derangement NOS, of the shoulder region
N08z2	Joint derangement NOS, of the upper arm
N08z3	Joint derangement NOS, of the forearm
N08z4	Joint derangement NOS, of the hand
N08z5	Joint derangement NOS, of the pelvic region and thigh
N08z6	Joint derangement NOS, of the ankle and foot
N08z7	Joint derangement NOS, of other specified site
N08z8	Joint derangement NOS, of multiple sites
N08zz	Joint derangement NOS
N09..	Other and unspecified joint disorders
N090.	Effusion of joint
N0900	Joint effusion of unspecified site
N0901	Joint effusion of the shoulder region
N0902	Joint effusion of the upper arm
N0903	Joint effusion of the forearm
N0904	Joint effusion of the hand
N0905	Joint effusion of the pelvic region and thigh
N0906	Joint effusion of the lower leg
N0907	Joint effusion of the ankle and foot
N0908	Joint effusion of other specified site
N0909	Effusion of multiple joints
N090A	Effusion of shoulder
N090B	Effusion of sternoclavicular joint
N090C	Effusion of acromioclavicular joint
N090D	Effusion of elbow
N090E	Effusion of distal radio-ulnar joint
N090F	Effusion of wrist
N090G	Effusion of metacarpophalangeal joint
N090H	Effusion of proximal interphalangeal joint of finger
N090J	Effusion of distal interphalangeal joint of finger
N090K	Effusion of hip
N090L	Effusion of sacro-iliac joint
N090M	Effusion of knee
N090N	Effusion of tibio-fibular joint
N090P	Effusion of ankle
N090Q	Effusion of subtalar joint

For peer review only

N090R	Effusion of talonavicular joint
N090S	Effusion of other tarsal joint
N090T	Effusion of 1st metatarsophalangeal joint
N090U	Effusion of lesser metatarsophalangeal joint
N090V	Effusion of interphalangeal joint of toe
N090W	Intermittent hydrarthrosis
N090X	Chronic joint effusion
N090Y	Acute joint effusion
N090z	Effusion of joint NOS
N091.	Haemarthrosis
N0910	Haemarthrosis of unspecified site
N0911	Haemarthrosis of the shoulder region
N0912	Haemarthrosis of the upper arm
N0913	Haemarthrosis of the forearm
N0914	Haemarthrosis of the hand
N0915	Haemarthrosis of the pelvic region and thigh
N0916	Haemarthrosis of the lower leg
N0917	Haemarthrosis of the ankle and foot
N0918	Haemarthrosis of other specified site
N0919	Haemarthrosis of multiple joints
N091A	Haemarthrosis of shoulder
N091B	Haemarthrosis of sternoclavicular joint
N091C	Haemarthrosis of acromioclavicular joint
N091D	Haemarthrosis of elbow
N091E	Haemarthrosis of distal radio-ulnar joint
N091F	Haemarthrosis of wrist
N091G	Haemarthrosis of MCP joint
N091H	Haemarthrosis of PIP joint of finger
N091J	Haemarthrosis of DIP joint of finger
N091K	Haemarthrosis of hip
N091L	Haemarthrosis of sacro-iliac joint
N091M	Haemarthrosis of knee
N091N	Haemarthrosis of tibio-fibular joint
N091P	Haemarthrosis of ankle
N091Q	Haemarthrosis of subtalar joint
N091R	Haemarthrosis of talonavicular joint
N091S	Haemarthrosis of other tarsal joint
N091T	Haemarthrosis-1st metatarsophalangeal joint
N091U	Haemarthrosis of lesser metatarsophalangeal joint
N091V	Haemarthrosis of interphalangeal joint of toe
N091z	Haemarthrosis NOS
N092.	Villonodular synovitis
N0920	Villonodular synovitis of unspecified site
N0921	Villonodular synovitis of the shoulder region
N0922	Villonodular synovitis of the upper arm
N0923	Villonodular synovitis of the forearm
N0924	Villonodular synovitis of the hand
N0925	Villonodular synovitis of the pelvic region and thigh

For peer review only

N0926	Villonodular synovitis of the lower leg
N0927	Villonodular synovitis of the ankle and foot
N0928	Villonodular synovitis of other specified site
N0929	Villonodular synovitis of multiple sites
N092A	Villonodular synovitis of shoulder joint
N092B	Villonodular synovitis of sternoclavicular joint
N092C	Villonodular synovitis of acromioclavicular joint
N092D	Villonodular synovitis of elbow
N092E	Villonodular synovitis of distal radio-ulnar joint
N092F	Villonodular synovitis of wrist
N092G	Villonodular synovitis of metacarpophalangeal joint
N092H	Villonodular synovitis of proximal interphalangeal joint of finger
N092J	Villonodular synovitis of distal interphalangeal joint of finger
N092K	Villonodular synovitis of hip
N092L	Villonodular synovitis of sacro-iliac joint
N092M	Villonodular synovitis of knee
N092N	Villonodular synovitis of tibio-fibular joint
N092P	Villonodular synovitis of ankle
N092Q	Villonodular synovitis of subtalar joint
N092R	Villonodular synovitis of talonavicular joint
N092S	Villonodular synovitis of other tarsal joint
N092T	Villonodular synovitis of 1st metatarsophalangeal joint
N092U	Villonodular synovitis of lesser metatarsophalangeal joint
N092V	Villonodular synovitis of interphalangeal joint of toe
N092z	Villonodular synovitis NOS
N093.	Palindromic rheumatism
N0930	Palindromic rheumatism of unspecified site
N0931	Palindromic rheumatism of the shoulder region
N0932	Palindromic rheumatism of the upper arm
N0933	Palindromic rheumatism of the forearm
N0934	Palindromic rheumatism of the hand
N0935	Palindromic rheumatism of the pelvic region and thigh
N0936	Palindromic rheumatism of the lower leg
N0937	Palindromic rheumatism of the ankle and foot
N0938	Palindromic rheumatism of other specified site
N0939	Palindromic rheumatism of multiple sites
N093z	Palindromic rheumatism NOS
N094.	Pain in joint - arthralgia
N0940	Arthralgia of unspecified site
N0941	Arthralgia of the shoulder region
N0942	Arthralgia of the upper arm
N0943	Arthralgia of the forearm
N0944	Arthralgia of the hand
N0945	Arthralgia of the pelvic region and thigh
N0946	Arthralgia of the lower leg
N0947	Arthralgia of the ankle and foot
N0948	Arthralgia of other specified site
N0949	Arthralgia of multiple joints

For peer review only

N094A	Arthralgia of shoulder
N094B	Arthralgia of sternoclavicular joint
N094C	Arthralgia of acromioclavicular joint
N094D	Arthralgia of elbow
N094E	Arthralgia of distal radio-ulnar joint
N094F	Arthralgia of wrist
N094G	Arthralgia of metacarpophalangeal joint
N094H	Arthralgia of proximal interphalangeal joint of finger
N094J	Arthralgia of distal interphalangeal joint of finger
N094K	Arthralgia of hip
N094L	Arthralgia of sacro-iliac joint
N094M	Arthralgia of knee
N094N	Arthralgia of tibio-fibular joint
N094P	Arthralgia of ankle
N094Q	Arthralgia of subtalar joint
N094R	Arthralgia of talonavicular joint
N094S	Arthralgia of other tarsal joint
N094T	Arthralgia of 1st metatarsophalangeal joint
N094U	Arthralgia of lesser metatarsophalangeal joint
N094V	Arthralgia of interphalangeal joint of toe
N094W	Anterior knee pain
N094z	Arthralgia NOS
N095.	Joint stiffness NEC
N0950	Stiff joint NEC, of unspecified site
N0951	Stiff joint NEC, of the shoulder region
N0952	Stiff joint NEC, of the upper arm
N0953	Stiff joint NEC, of the forearm
N0954	Stiff joint NEC, of the hand
N0955	Stiff joint NEC, of the pelvic region and thigh
N0956	Stiff joint NEC, of the lower leg
N0957	Stiff joint NEC, of the ankle and foot
N0958	Stiff joint NEC, of other specified site
N0959	Multiple stiff joints
N095A	Stiff shoulder NEC
N095B	Stiff sternoclavicular joint NEC
N095C	Stiff acromioclavicular joint NEC
N095D	Stiff elbow NEC
N095E	Stiff distal radio-ulnar joint NEC
N095F	Stiff wrist NEC
N095G	Stiff metacarpophalangeal joint NEC
N095H	Stiff proximal interphalangeal joint of finger NEC
N095J	Stiff distal interphalangeal joint of finger NEC
N095K	Stiff hip NEC
N095L	Stiff sacro-iliac joint NEC
N095M	Stiff knee NEC
N095N	Stiff tibio-fibular joint NEC
N095P	Stiff ankle NEC
N095Q	Stiff subtalar joint NEC

For peer review only

N095R	Stiff talonavicular joint NEC
N095S	Stiff other tarsal joint NEC
N095T	Stiff 1st metatarsophalangeal joint NEC
N095U	Stiff lesser metatarsophalangeal joint NEC
N095V	Stiff interphalangeal joint of toe NEC
N095W	Stiff finger
N095z	Joint stiffness NEC, NOS
N096.	Other joint symptoms
N0960	Other joint symptoms of unspecified site
N0961	Other joint symptoms of the shoulder region
N0962	Other joint symptoms of the upper arm
N0963	Other joint symptoms of the forearm
N0964	Other joint symptoms of the hand
N0965	Other joint symptoms of the pelvic region and thigh
N0966	Other joint symptoms of the lower leg
N0967	Other joint symptoms of the ankle and foot
N0968	Other joint symptoms of other specified site
N0969	Other joint symptoms of multiple sites
N096A	Other symptoms - shoulder
N096B	Other symptoms in sternoclavicular joint
N096C	Other symptoms in acromioclavicular joint
N096D	Other symptoms - elbow
N096E	Other symptoms in distal radio-ulnar joint
N096F	Other symptoms - wrist
N096G	Other symptoms - MCPJ
N096H	Other symptoms in proximal interphalangeal joint of finger
N096J	Other symptoms in distal interphalangeal joint of finger
N096K	Other symptoms - hip
N096L	Other symptoms in sacro-iliac joint
N096M	Other symptoms - knee
N096N	Other symptoms in tibio-fibular joint
N096P	Other symptoms - ankle
N096Q	Other symptoms in subtalar joint
N096R	Other symptoms in talonavicular joint
N096S	Other symptoms in other tarsal joint
N096T	Other symptoms in 1st metatarsophalangeal joint
N096U	Other symptoms in lesser metatarsophalangeal joint
N096V	Other symptoms in interphalangeal joint of toe
N096z	Other joint symptoms NOS
N097.	Difficulty in walking
N0970	Walking difficulty due to unspecified site
N0971	Walking difficulty due to pelvic region and thigh
N0972	Walking difficulty due to lower leg
N0973	Walking difficulty due to ankle and foot
N0974	Walking difficulty due to other specified site
N0975	Walking difficulty due to multiple sites
N097z	Difficulty in walking NOS
N098.	Synovial osteochondromatosis

For peer review only

N0980	Synovial osteochondromatosis of shoulder
N0981	Synovial osteochondromatosis of sternoclavicular joint
N0982	Synovial osteochondromatosis of acromioclavicular joint
N0983	Synovial osteochondromatosis of elbow
N0984	Synovial osteochondromatosis of distal radio-ulnar joint
N0985	Synovial osteochondromatosis of wrist
N0986	Synovial osteochondromatosis of metacarpophalangeal joint
N0987	Synovial osteochondromatosis of proximal interphalangeal joint of finger
N0988	Synovial osteochondromatosis of distal interphalangeal joint of finger
N0989	Synovial osteochondromatosis of hip
N098A	Synovial osteochondromatosis of sacro-iliac joint
N098B	Synovial osteochondromatosis of knee
N098C	Synovial osteochondromatosis of tibio-fibular joint
N098D	Synovial osteochondromatosis of ankle
N098E	Synovial osteochondromatosis of subtalar joint
N098F	Synovial osteochondromatosis of talonavicular joint
N098G	Synovial osteochondromatosis of other tarsal joint
N098H	Synovial osteochondromatosis of 1st metatarsophalangeal joint
N098J	Synovial osteochondromatosis of lesser metatarsophalangeal joint
N098K	Synovial osteochondromatosis of interphalangeal joint of toe
N099.	Clicking joint
N0990	Clicking shoulder
N0991	Clicking sternoclavicular joint
N0992	Clicking acromioclavicular joint
N0993	Clicking elbow
N0994	Clicking distal radio-ulnar joint
N0995	Clicking wrist
N0996	Clicking metacarpophalangeal joint
N0997	Clicking proximal interphalangeal joint of finger
N0998	Clicking distal interphalangeal joint of finger
N0999	Clicking hip
N099A	Multiple clicking joints
N099B	Clicking sacro-iliac joint
N099C	Clicking knee
N099D	Clicking tibio-fibular joint
N099E	Clicking ankle
N099F	Clicking subtalar joint
N099G	Clicking talonavicular joint
N099H	Clicking other tarsal joint
N099J	Clicking 1st metatarsophalangeal joint
N099K	Clicking lesser metatarsophalangeal joint
N099L	Clicking interphalangeal joint of toe
N09A.	Patellofemoral disorder
N09AX	Disorder of patella, unspecified
N09B.	Osteophyte
N09C.	Fistula of joint
N09y.	Other specified joint disorders
N09y0	Other specified joint disorders of unspecified site

For peer review only

N09y1	Other specified joint disorders of the shoulder region
N09y2	Other specified joint disorders of the upper arm
N09y3	Other specified joint disorders of the forearm
N09y4	Other specified joint disorders of the hand
N09y5	Other specified joint disorders of the pelvic region and thigh
N09y6	Other specified joint disorders of the lower leg
N09y7	Other specified joint disorders of the ankle and foot
N09y8	Other specified joint disorders of other specified site
N09y9	Other specified joint disorders of multiple sites
N09yz	Other specified joint disorders NOS
N09z.	Joint disorders NOS
N09z0	Joint disorder NOS, of unspecified site
N09z1	Joint disorder NOS, of shoulder region
N09z2	Joint disorder NOS, of the upper arm
N09z3	Joint disorder NOS, of the forearm
N09z4	Joint disorder NOS, of the hand
N09z5	Joint disorder NOS, of the pelvic region and thigh
N09z6	Joint disorder NOS, of the lower leg
N09z7	Joint disorder NOS, of ankle and foot
N09z8	Joint disorder NOS, of other specified site
N09z9	Joint disorder NOS, of multiple sites
N09zz	Joint disorders NOS
N0y..	Other specified arthropathies
N0z..	Arthropathies NOS
N1...	Vertebral column syndromes
N10..	Inflammatory spondylopathies
N100.	Ankylosing spondylitis
N101.	Spinal enthesopathy
N102.	Sacroiliitis NEC
N10y.	Other inflammatory spondylopathies
N10y0	Inflammatory spondylopathies in diseases EC
N10yz	Other inflammatory spondylopathies NOS
N10z.	Spondylitis NOS
N11..	Spondylosis and allied disorders
N110.	Cervical spondylosis without myelopathy
N1100	Single-level cervical spondylosis without myelopathy
N1101	Two-level cervical spondylosis without myelopathy
N1102	Multiple-level cervical spondylosis without myelopathy
N111.	Cervical spondylosis with myelopathy
N1110	Single-level cervical spondylosis with myelopathy
N1111	Two-level cervical spondylosis with myelopathy
N1112	Multiple-level cervical spondylosis with myelopathy
N1113	Cervical myelopathy
N112.	Thoracic spondylosis without myelopathy
N1120	Single-level thoracic spondylosis without myelopathy
N1121	Two-level thoracic spondylosis without myelopathy
N1122	Multiple-level thoracic spondylosis without myelopathy
N1123	Dorsal spondylosis without myelopathy

For peer review only

N113.	Thoracic spondylosis with myelopathy
N1130	Single-level thoracic spondylosis with myelopathy
N1131	Two-level thoracic spondylosis with myelopathy
N1132	Multiple-level thoracic spondylosis with myelopathy
N114.	Lumbosacral spondylosis without myelopathy
N1140	Single-level lumbosacral spondylosis without myelopathy
N1141	Two-level lumbosacral spondylosis without myelopathy
N1142	Multiple-level lumbosacral spondylosis without myelopathy
N115.	Lumbosacral spondylosis with myelopathy
N1150	Single-level lumbosacral spondylosis with myelopathy
N1151	Two-level lumbosacral spondylosis with myelopathy
N1152	Multiple-level lumbosacral spondylosis with myelopathy
N116.	Kissing spine
N117.	Ankylosing vertebral hyperostosis
N118.	Traumatic spondylopathy
N119.	Cervical spondylosis with radiculopathy
N1190	Single-level cervical spondylosis with radiculopathy
N1191	Two-level cervical spondylosis with radiculopathy
N1192	Multiple-level cervical spondylosis with radiculopathy
N11A.	Cervical spondylosis with vascular compression
N11B.	Thoracic spondylosis with radiculopathy
N11B0	Single-level thoracic spondylosis with radiculopathy
N11B1	Two-level thoracic spondylosis with radiculopathy
N11B2	Multiple-level thoracic spondylosis with radiculopathy
N11C.	Lumbosacral spondylosis with radiculopathy
N11C0	Single-level lumbosacral spondylosis with radiculopathy
N11C1	Two-level lumbosacral spondylosis with radiculopathy
N11C2	Multiple-level lumbosacral spondylosis with radiculopathy
N11D.	Osteoarthritis of spine
N11D0	Osteoarthritis of cervical spine
N11D1	Osteoarthritis of thoracic spine
N11D2	Osteoarthritis of lumbar spine
N11D3	Osteoarthritis of spine NOS
N11E.	Cervical spondylosis
N11F.	Axial spondyloarthritis
N11y.	Other spondyloses and allied disorders
N11y0	Brucella spondylitis
N11y1	Enterobacterial spondylitis
N11y2	Neuropathic spondylopathy
N11z.	Spondylosis NOS
N11z0	Spondylosis without myelopathy, NOS
N11z1	Spondylosis with myelopathy, NOS
N11zz	Spondylosis NOS
N12..	Intervertebral disc disorders
N120.	Cervical disc displacement without myelopathy
N121.	Thoracic disc displacement without myelopathy
N122.	Lumbar disc displacement
N123.	Disc displacement, site unspecified, without myelopathy

For peer review only

N124.	Schmorl's nodes
N1240	Schmorl's nodes of unspecified region
N1241	Schmorl's nodes of the thoracic region
N1242	Schmorl's nodes of the lumbar region
N124z	Schmorl's nodes, region NOS
N125.	Cervical disc degeneration
N126.	Thoracic disc degeneration
N127.	Lumbar disc degeneration
N128.	Degenerative disc disease NOS
N129.	Disc disorder with myelopathy
N1290	Unspecified disc disorder with myelopathy
N1291	Cervical disc disorder with myelopathy
N1292	Thoracic disc disorder with myelopathy
N1293	Lumbar disc disorder with myelopathy
N129z	Disc disorder with myelopathy NOS
N12A.	Postlaminectomy syndrome
N12A0	Postlaminectomy syndrome of unspecified site
N12A1	Cervical postlaminectomy syndrome
N12A2	Thoracic postlaminectomy syndrome
N12A3	Lumbar postlaminectomy syndrome
N12Az	Postlaminectomy syndrome NOS
N12B.	Disc prolapse with myelopathy
N12B0	Cervical disc prolapse with myelopathy
N12B1	Thoracic disc prolapse with myelopathy
N12B2	Lumbar disc prolapse with myelopathy
N12C.	Disc prolapse with radiculopathy
N12C0	Cervical disc prolapse with radiculopathy
N12C1	Thoracic disc prolapse with radiculopathy
N12C2	Lumbar disc prolapse with radiculopathy
N12C3	Lumbar disc prolapse with cauda equina compression
N12C4	Prolapsed lumbar intervertebral disc with sciatica
N12D.	Narrowing intervertebral disc space
N12z.	Other and unspecified disc disorders
N12z0	Other disc disorders of unspecified site
N12z1	Other cervical disc disorders
N12z2	Other thoracic disc disorders
N12z3	Other lumbar disc disorders
N12z4	Cervical discitis
N12z5	Annular tear of cervical disc
N12z6	Resorption of cervical disc
N12z7	Calcification of cervical disc
N12z8	Thoracic discitis
N12z9	Annular tear of thoracic disc
N12zA	Resorption of thoracic disc
N12zB	Calcification of thoracic disc
N12zC	Lumbar discitis
N12zD	Annular tear of lumbar disc
N12zE	Resorption of lumbar disc

For peer review only

N12zF	Calcification of lumbar disc
N12zG	Infection of intervertebral disc - pyogenic
N12zH	Cervical disc disorder with radiculopathy
N12zz	Disc disorders NOS
N13..	Other cervical disorders
N130.	Cervical spinal stenosis
N1300	Idiopathic cervical spinal stenosis
N1301	Degenerative cervical spinal stenosis
N1302	Iatrogenic cervical spinal stenosis
N1303	Cervical spinal stenosis secondary to other disease
N131.	Cervicalgia - pain in neck
N132.	Cervicocranial syndrome
N133.	Cervicobrachial syndrome
N134.	Brachial (cervical) neuritis
N135.	Torticollis unspecified
N1350	Intermittent torticollis
N1351	Rheumatic torticollis
N135z	Torticollis NOS
N136.	Panniculitis of neck
N137.	Cervical posterior longitudinal ligament ossification
N138.	Cervicalgia
N13y.	Other cervical syndromes
N13y0	Cervical syndrome NEC
N13y1	Klippel's disease
N13y2	Crick in neck
N13y3	Cervical root syndrome
N13yz	Other cervical syndromes NOS
N13z.	Cervical and neck disorders NOS
N14..	Other and unspecified back disorders
N140.	Spinal stenosis, excluding cervical region
N1400	Spinal stenosis of unspecified region
N1401	Thoracic spinal stenosis
N1402	Lumbar spinal stenosis
N1403	Idiopathic thoracic spinal stenosis
N1404	Degenerative thoracic spinal stenosis
N1405	Iatrogenic thoracic spinal stenosis
N1406	Thoracic spinal stenosis secondary to other disease
N1407	Idiopathic lumbar spinal stenosis
N1408	Degenerative lumbar spinal stenosis
N1409	Iatrogenic lumbar spinal stenosis
N140A	Lumbar spinal stenosis secondary to other disease
N140z	Spinal stenosis NOS
N141.	Pain in thoracic spine
N142.	Pain in lumbar spine
N1420	Lumbago with sciatica
N143.	Sciatica
N144.	Thoracic and lumbosacral neuritis
N1440	Thoracic neuritis, unspecified

For peer review only

N1441	Lumbosacral neuritis, unspecified
N144z	Thoracic and lumbosacral neuritis NOS
N145.	Backache, unspecified
N146.	Disorders of the sacrum
N1460	Lumbosacral ankylosis
N1461	Sacroiliac ankylosis
N1462	Sacral ankylosis NOS
N1463	Lumbosacral instability
N1464	Sacroiliac instability
N1465	Sacral instability NOS
N1466	Sacroiliac disorder
N146z	Disorders of the sacrum NOS
N147.	Disorders of the coccyx
N1470	Unspecified disorder of the coccyx
N1471	Hypermobility of the coccyx
N1472	Coccygodynia
N147z	Coccyx disorder NOS
N148.	Ankylosis or instability of cervical,thoracic or lumbar spine
N1480	Atlanto-occipital ankylosis
N1481	Atlanto-axial ankylosis
N1482	Cervical spine ankylosis
N1483	Cervico-thoracic ankylosis
N1484	Thoracic spine ankylosis
N1485	Thoraco-lumbar ankylosis
N1486	Lumbar spine ankylosis
N1487	Atlanto-occipital instability
N1488	Atlanto-axial instability
N1489	Cervical spine instability
N148A	Cervico-thoracic instability
N148B	Thoracic spine instability
N148C	Lumbar spine instability
N149.	Back stiffness
N14A.	Neurogenic claudication
N14X.	Sacrococcygeal disorders, not elsewhere classified
N14y.	Other back symptoms
N14z.	Back disorders NOS
N1y..	Other specified disorders of vertebral column
N1y0.	Recurrent atlantoaxial subluxation with myelopathy
N1y1.	Fatigue fracture of vertebra
N1y2.	Pars interarticularis stress fracture
N1z..	Vertebral column disorder NOS
N2...	Rheumatism, excluding the back
N20..	Polymyalgia rheumatica
N200.	Giant cell arteritis with polymyalgia rheumatica
N21..	Peripheral enthesopathies and allied syndromes
N210.	Adhesive capsulitis of the shoulder
N211.	Rotator cuff shoulder syndrome and allied disorders
N2110	Rotator cuff syndrome, unspecified

For peer review only

N2111	Calcifying tendinitis of the shoulder
N2112	Bicipital tenosynovitis
N2113	Supraspinatus tendinitis
N2114	Partial thickness rotator cuff tear
N2115	Full thickness rotator cuff tear
N2116	Subacromial bursitis
N2117	Subdeltoid bursitis
N2118	Bursitis of shoulder
N211z	Rotator cuff syndrome NOS
N212.	Other shoulder affections NEC
N2120	Periarthritis of shoulder
N2121	Scapulohumeral fibrositis
N2122	Subacromial impingement
N2123	Coracoid impingement
N2124	Impingement syndrome of shoulder
N2125	Shoulder tendonitis
N212z	Other shoulder affections NEC, NOS
N213.	Enthesopathy of the elbow region
N2130	Elbow enthesopathy unspecified
N2131	Medial epicondylitis of the elbow
N2132	Lateral epicondylitis of the elbow
N2133	Olecranon bursitis
N2134	Biceps tendinitis
N2135	Triceps tendinitis
N213z	Elbow enthesopathy NOS
N214.	Enthesopathy of the wrist and carpus
N2140	Bursitis of wrist
N2141	Bursitis of hand
N2142	Periarthritis of wrist
N2143	Carpometacarpal bossing
N214z	Wrist or carpus enthesopathy NOS
N215.	Enthesopathy of the hip region
N2150	Hip enthesopathy, unspecified
N2151	Bursitis of hip
N2152	Gluteal tendinitis
N2153	Iliac crest spur
N2154	Psoas tendinitis
N2155	Trochanteric tendinitis
N2156	Adductor tendinitis
N2157	Trochanteric bursitis
N2158	Snapping hip
N2159	Iliotibial band syndrome
N215A	Ischial bursitis
N215B	Femoroacetabular impingement
N215z	Hip enthesopathy NOS
N216.	Enthesopathy of the knee
N2160	Bursitis of the knee NOS
N2161	Pes anserinus tendinitis and bursitis

For peer review only

N2162	Tibial collateral ligament bursitis
N2163	Fibular collateral ligament bursitis
N2164	Patellar tendinitis
N2165	Prepatellar bursitis
N2166	Infrapatellar bursitis
N2167	Subpatellar bursitis
N2168	Biceps femoris tendinitis
N2169	Semimembranosus tendinitis
N216z	Knee enthesopathy NOS
N217.	Enthesopathy of the ankle and tarsus
N2170	Enthesopathy of the ankle unspecified
N2171	Enthesopathy of the tarsus unspecified
N2172	Metatarsalgia NOS
N2173	Achilles bursitis
N2174	Achilles tendinitis
N2175	Tibialis anterior tendinitis
N2176	Tibialis posterior tendinitis
N2177	Calcaneal spur
N2178	Peroneal tendinitis
N2179	Plantar fasciitis
N217A	Posterior calcaneal exostosis (pump bump)
N217B	Anterior ankle impingement
N217C	Fibular impingement
N217z	Ankle or tarsus enthesopathy NOS
N21y.	Other peripheral enthesopathies
N21y0	Anterior shin splints
N21y1	Posterior shin splints
N21z.	Enthesopathy NOS
N21z0	Capsulitis NOS
N21z1	Periarthritis NOS
N21z2	Tendinitis NOS
N21z3	Exostosis of unspecified site
N21z4	Subungual exostosis
N21z5	Subungual exostosis of great toe
N21z6	Subungual exostosis of lesser toe
N21z7	Exostosis
N21zz	Peripheral enthesopathy NOS
N22..	Other disorders of the synovium, tendon and bursa
N220.	Synovitis and tenosynovitis
N2200	Synovitis or tenosynovitis NOS
N2201	Synovitis and tenosynovitis with disorders EC
N2202	Tendon sheath giant cell tumor
N2203	Trigger finger - acquired
N2204	Radial styloid tenosynovitis
N2205	Other tenosynovitis of hand or wrist
N2206	Tenosynovitis of ankle
N2207	Tenosynovitis of foot
N2208	Villonodular synovitis of tendon sheath

For peer review only

N2209	Plant thorn synovitis
N220A	Flexor tenosynovitis of wrist
N220B	Flexor tenosynovitis of finger
N220C	Flexor tenosynovitis of thumb
N220D	Extensor tenosynovitis of wrist
N220E	Extensor tenosynovitis of finger
N220F	Extensor tenosynovitis of thumb
N220G	Acquired trigger thumb
N220H	Achilles tenosynovitis
N220J	Tibialis anterior tenosynovitis
N220K	Tibialis posterior tenosynovitis
N220L	Extensor hallucis longus tenosynovitis
N220M	Extensor digitorum longus tenosynovitis
N220N	Peroneus longus tenosynovitis
N220P	Peroneus brevis tenosynovitis
N220Q	Transient synovitis
N220R	Chronic crepitant synovitis of hand and wrist
N220S	Synovitis of hip
N220T	Synovitis NOS
N220V	Synovitis of knee
N220W	Synovitis of elbow
N220X	Synovitis of shoulder
N220Y	Irritable hip
N220z	Other synovitis and tenosynovitis
N221.	Bunion
N2210	Infected bunion
N222.	Specific bursitides
N2220	Beat elbow
N2221	Beat hand
N2222	Beat knee
N2223	Miners' elbow
N2224	Miners' knee
N2225	Housemaids' knee
N2226	Calcium deposit in bursa
N2227	Syphilitic bursitis
N222z	Specific bursitides NOS
N223.	Bursitis NOS
N224.	Ganglion and cyst of synovium, tendon and bursa
N2240	Synovial cyst unspecified
N2241	Ganglion of joint
N2242	Ganglion of tendon sheath
N2243	Ganglion unspecified
N2244	Cyst of bursa
N2245	Ganglion of wrist
N2246	Ganglion of knee
N2247	Ganglion of superior tibio-fibular joint
N2248	Ganglion of ankle
N2249	Ganglion of flexor tendon sheath of finger

For peer review only

N224A	Synovial cyst of popliteal space
N224B	Ganglion of hand
N224C	Ganglion of foot
N224D	Cyst of tendon sheath
N224E	Digital mucous cyst
N224z	Ganglion or cyst of synovium/tendon/bursa NOS
N225.	Rupture of synovium
N2250	Rupture of synovium, unspecified
N2251	Rupture of popliteal space synovial cyst
N225z	Rupture of synovium NOS
N226.	Nontraumatic tendon rupture
N2260	Nontraumatic tendon rupture, unspecified
N2261	Rotator cuff complete rupture
N2262	Biceps tendon rupture
N2263	Hand and wrist extensor tendon rupture
N2264	Hand and wrist flexor tendon rupture
N2265	Quadriceps tendon rupture
N2266	Nontraumatic rupture of patellar tendon
N2267	Nontraumatic rupture of Achilles tendon
N2268	Extensor digitorum communis rupture
N2269	Extensor pollicis longus rupture
N226A	Long head of biceps rupture
N226B	Subluxation of long head of biceps
N226C	Flexor digitorum sublimis tendon rupture
N226D	Flexor digitorum profundus tendon rupture
N226E	Flexor pollicis longus tendon rupture
N226F	Tibialis posterior rupture
N226G	Peroneus longus rupture
N226H	Subluxation of peroneal tendon
N226J	Subluxation of tendon, wrist or hand
N226K	Dislocation of tendon, wrist or hand
N226L	Bowstringing of tendon, wrist or hand
N226M	Spontaneous rupture of flexor tendons
N226N	Spontaneous rupture of extensor tendons
N226y	Other foot and ankle tendon rupture
N226z	Other nontraumatic tendon rupture
N227.	Posterior tibial tendon insufficiency
N228.	Tendinous xanthoma
N22y.	Other synovial, tendon and bursa disorders
N22y0	Contracture of tendon sheath
N22y1	Calcification of tendon NOS
N22y2	Abscess of tendon
N22y3	Abscess of bursa
N22y4	Synovial plica
N22y5	Short tendon
N22y6	Abscess of tendon-arm
N22y7	Abscess of tendon-forearm
N22y8	Abscess of tendon-hand

For peer review only

N22y9	Abscess of tendon-thigh
N22yA	Abscess of tendon-leg
N22yB	Abscess of tendon-foot
N22yC	Pyogenic infection of tendon sheath
N22yD	Tuberculous infection of tendon sheath
N22yE	Abscess of bursa-shoulder
N22yF	Abscess of bursa-elbow
N22yG	Abscess of bursa-wrist
N22yH	Abscess of bursa-hip
N22yJ	Abscess of bursa-knee
N22yK	Abscess of bursa-ankle
N22yL	Abscess of bursa-foot
N22yM	Short Achilles tendon - acquired
N22yN	Achilles degeneration
N22yz	Other synovial, tendon or bursa disorder NOS
N22z.	Synovial, tendon or bursa disorder NOS
N23..	Muscle, ligament and fascia disorders
N230.	Infective myositis
N2300	Infective myositis-neck
N2301	Infective myositis-back
N2302	Infective myositis-shoulder
N2303	Infective myositis-arm
N2304	Infective myositis-forearm
N2305	Infective myositis-hand
N2306	Infective myositis-pelvis
N2307	Infective myositis-thigh
N2308	Infective myositis-leg
N2309	Infective myositis-foot
N230A	Muscle abscess
N230B	Muscle abscess-neck
N230C	Muscle abscess-back
N230D	Muscle abscess-shoulder
N230E	Muscle abscess-arm
N230F	Muscle abscess-forearm
N230G	Muscle abscess-hand
N230H	Muscle abscess-pelvis
N230J	Muscle abscess-thigh
N230K	Muscle abscess-leg
N230L	Muscle abscess-foot
N231.	Muscle calcification and ossification
N2310	Muscular calcification and ossification, unspecified
N2311	Progressive myositis ossificans
N2312	Traumatic myositis ossificans
N2313	Post-operative heterotopic calcification
N2314	Polymyositis ossificans
N2315	Calcification and ossification of muscles associated with burns
N2316	Paralytic calcification and ossification of muscle
N231z	Muscle calcification or ossification NOS

For peer review only

N232.	Muscle wasting and disuse atrophy NEC
N2320	Amyotrophia NOS
N2321	Myofibrosis
N2322	Muscle wasting NEC
N232z	Muscle wasting/disuse atrophy NEC NOS
N233.	Other specific muscle disorder
N2330	Arthrogryposis
N2331	Immobility syndrome
N2332	Myositis in sarcoidosis
N2333	Rhabdomyolysis
N2334	Antisynthetase syndrome
N233z	Other specific muscle disorder NOS
N234.	Laxity of ligament
N235.	Hypermobility syndrome
N236.	Dupuytren's contracture
N2360	Dupuytren's disease of palm
N2361	Dupuytren's disease of palm, nodules with no contracture
N2362	Dupuytren's disease of palm, with contracture
N2363	Dupuytren's disease of finger(s)
N2364	Dupuytren's disease of finger(s), nodules with no contracture
N2365	Dupuytren's disease of finger(s), with contracture
N2366	Dupuytren's disease of palm and finger(s)
N2367	Dupuytren's disease of palm and finger(s), nodules with no contracture
N2368	Dupuytren's disease of palm and finger(s), with contracture
N237.	Other fibromatoses
N2370	Plantar fascial fibromatosis
N2371	Knuckle pads
N2372	Nodular fasciitis
N2373	Pseudosarcomatous fibromatosis
N237z	Other fibromatoses NOS
N238.	Muscle contracture
N2380	Contracture of pectoralis major
N2381	Contracture of triceps
N2382	Contracture of biceps
N2383	Contracture of wrist flexor(s)
N2384	Contracture of wrist extensor(s)
N2385	Contracture of flexor pollicis longus
N2386	Contracture of thumb extensor(s)
N2387	Contracture of flexor digitorum superficialis
N2388	Contracture of flexor digitorum profundus
N2389	Contracture of adductor pollicis
N238A	Contracture of other intrinsic muscle(s) of hand
N238B	Contracture of iliopsoas
N238C	Contracture of rectus femoris
N238D	Contracture of adductor muscle(s) of hip
N238E	Contracture of abductor muscle(s) of hip
N238F	Contracture of hamstring(s)
N238G	Contracture of quadriceps

For peer review only

N238H	Contracture of tendo achilles
N238J	Contracture of tibialis anterior
N238K	Contracture of tibialis posterior
N238L	Contracture of long toe flexor(s)
N238M	Contracture of long toe extensor(s)
N238N	Contracture of intrinsic muscle(s) of foot
N239.	Fibromyalgia
N23y.	Other muscle, ligament and fascia disorder
N23y0	Interstitial myositis
N23y1	Foreign body muscle granuloma
N23y2	Nontraumatic muscle rupture
N23y3	Diastasis recti abdominis
N23y4	Spasm of muscle
N23y5	Inappropriate firing of muscle
N23y6	Palmar space infection, thenar
N23y7	Palmar space infection, mid-palm
N23y8	Palmar space infection, hypo-thenar
N23y9	Calcific tendinitis
N23yA	Diastasis of muscle
N23yB	Ischaemic infarction of muscle
N23yC	Contracture of muscle
N23yD	Muscle strain
N23yE	Spasm of back muscles
N23yF	Muscle crush syndrome
N23yz	Other muscle, ligament or fascia disorder NOS
N23z.	Muscle, ligament or fascia disorder NOS
N24..	Other soft tissue disorders
N240.	Rheumatism and fibrositis unspecified
N2400	Rheumatism unspecified
N2401	Fibrositis unspecified
N2402	Muscular rheumatism
N2403	Rheumatic pain
N2405	Fibrositis of neck
N2406	Fibrositis arm
N2407	Hand rheumatism
N240z	Rheumatism or fibrositis NOS
N241.	Myalgia and myositis unspecified
N2410	Myalgia unspecified
N2411	Myositis unspecified
N2412	Fibromyositis NOS
N2413	Viral myalgia
N241z	Myalgia or myositis NOS
N242.	Neuralgia, neuritis and radiculitis unspecified
N2420	Neuralgia unspecified
N2421	Neuritis unspecified
N2422	Radiculitis unspecified
N2423	Neuropathic pain
N242z	Neuralgia, neuritis or radiculitis NOS

For peer review only

N243.	Panniculitis unspecified
N2430	Panniculitis of unspecified site
N2431	Hypertrophy of the knee fat pad
N2432	Weber - Christian disease
N243z	Panniculitis NOS
N244.	Fasciitis unspecified
N245.	Pain in limb
N2450	Hand pain
N2451	Foot pain
N2452	Pain in leg
N2453	Pain in arm
N2454	Calf pain
N2455	Axillary pain
N2456	Tender heel pad
N2457	Shoulder pain
N246.	Residual foreign body in soft tissue
N247.	Other musculoskeletal limb symptoms
N2470	Swelling of limb
N2471	Leg cramps
N2472	Cramp
N247z	Musculoskeletal limb symptoms NOS
N248.	Fibromyalgia
N2480	Myofascial pain syndrome
N2481	Piriformis syndrome
N24z.	Soft tissue disorders NOS
N25..	SAPHO syndrome (Synovitis Acne Pustulosis Hyperostosis Osteomyelitis)
N2y..	Other specified nonarticular rheumatism
N2z..	Nonarticular rheumatism NOS
N3...	Osteopathies, chondropathies and acquired musculoskeletal deformities
N30..	Osteomyelitis, periostitis and other infections affecting bone
N300.	Acute osteomyelitis
N3000	Acute osteomyelitis of unspecified site
N3001	Acute osteomyelitis of the shoulder region
N3002	Acute osteomyelitis of the upper arm
N3003	Acute osteomyelitis of the forearm
N3004	Acute osteomyelitis of the hand
N3005	Acute osteomyelitis of the pelvic region and thigh
N3006	Acute osteomyelitis of the lower leg
N3007	Acute osteomyelitis of the ankle and foot
N3008	Acute osteomyelitis of other specified site
N3009	Acute osteomyelitis of multiple sites
N300A	Acute osteomyelitis-cervical spine
N300B	Acute osteomyelitis-thoracic spine
N300C	Acute osteomyelitis-lumbar spine
N300D	Acute osteomyelitis-sacrum
N300E	Acute osteomyelitis-coccyx
N300F	Acute osteomyelitis-clavicle
N300G	Acute osteomyelitis-scapula

For peer review only

N300H	Acute osteomyelitis-humerus
N300J	Acute osteomyelitis-radius
N300K	Acute osteomyelitis-ulna
N300L	Acute osteomyelitis-carpal bone
N300M	Acute osteomyelitis-metacarpal
N300N	Acute osteomyelitis-phalanx of finger/thumb
N300P	Acute osteomyelitis-pelvis
N300Q	Acute osteomyelitis-femur
N300R	Acute osteomyelitis-patella
N300S	Acute osteomyelitis-tibia
N300T	Acute osteomyelitis-fibula
N300U	Acute osteomyelitis-calcaneum
N300V	Acute osteomyelitis-talus
N300W	Acute osteomyelitis-other tarsal bone
N300X	Acute osteomyelitis-metatarsal
N300Y	Acute osteomyelitis-phalanx of toe
N300Z	Acute haematogenous osteomyelitis
N300z	Acute osteomyelitis NOS
N301.	Chronic osteomyelitis
N3010	Chronic osteomyelitis of unspecified site
N3011	Chronic osteomyelitis of the shoulder region
N3012	Chronic osteomyelitis of the upper arm
N3013	Chronic osteomyelitis of the forearm
N3014	Chronic osteomyelitis of the hand
N3015	Chronic osteomyelitis of the pelvic region and thigh
N3016	Chronic osteomyelitis of the lower leg
N3017	Chronic osteomyelitis of the ankle and foot
N3018	Chronic osteomyelitis of other specified site
N3019	Chronic osteomyelitis of multiple sites
N301A	Chronic osteomyelitis-cervical spine
N301B	Chronic osteomyelitis-thoracic spine
N301C	Chronic osteomyelitis-lumbar spine
N301D	Chronic osteomyelitis-sacrum
N301E	Chronic osteomyelitis-coccyx
N301F	Brodie's abscess-cervical spine
N301G	Brodie's abscess-thoracic spine
N301H	Brodie's abscess-lumbar spine
N301J	Brodie's abscess-sacrum
N301K	Brodie's abscess-coccyx
N301L	Chronic multifocal osteomyelitis
N301M	Chronic osteomyelitis with draining sinus
N301z	Chronic osteomyelitis NOS
N302.	Unspecified osteomyelitis
N3020	Unspecified osteomyelitis of unspecified site
N3021	Unspecified osteomyelitis of the shoulder region
N3022	Unspecified osteomyelitis of the upper arm
N3023	Unspecified osteomyelitis of the forearm
N3024	Unspecified osteomyelitis of the hand

For peer review only

N3025	Unspecified osteomyelitis of the pelvic region and thigh
N3026	Unspecified osteomyelitis of the lower leg
N3027	Unspecified osteomyelitis of the ankle and foot
N3028	Unspecified osteomyelitis of other specified site
N3029	Unspecified osteomyelitis of multiple sites
N302A	Infection of cervical spine
N302B	Infection of thoracic spine
N302C	Infection of lumbar spine
N302D	Infection of sacrum
N302E	Infection of coccyx
N302F	Infection of clavicle
N302G	Infection of scapula
N302H	Infection of humerus
N302J	Infection of radius
N302K	Infection of ulna
N302L	Infection of carpal bone
N302M	Infection of metacarpal
N302N	Infection of phalanx of finger or thumb
N302P	Infection of pelvis
N302Q	Infection of femur
N302R	Infection of patella
N302S	Infection of tibia
N302T	Infection of fibula
N302U	Infection of calcaneum
N302V	Infection of talus
N302W	Infection of other tarsal bone
N302X	Infection of metatarsal
N302Y	Infection of phalanx of toe
N302Z	Infection of multiple bones
N302a	Osteomyelitis of vertebra
N302b	Brodie's abscess
N302z	Unspecified osteomyelitis NOS
N303.	Periostitis without osteomyelitis
N3030	Periostitis without mention of osteomyelitis, of unspecified site
N3031	Periostitis without mention of osteomyelitis, of the shoulder region
N3032	Periostitis without mention of osteomyelitis, of the upper arm
N3033	Periostitis without osteomyelitis, of the forearm
N3034	Periostitis without osteomyelitis, of the hand
N3035	Periostitis without osteomyelitis, of the pelvic region and thigh
N3036	Periostitis without mention of osteomyelitis, of the lower leg
N3037	Periostitis without mention of osteomyelitis, of the ankle and foot
N3038	Periostitis without mention of osteomyelitis, of other specified site
N3039	Periostitis without mention of osteomyelitis, of multiple sites
N303A	Periostitis without osteomyelitis-cervical spine
N303B	Periostitis without osteomyelitis-thoracic spine
N303C	Periostitis without osteomyelitis-lumbar spine
N303D	Periostitis without osteomyelitis-sacrum
N303E	Periostitis without osteomyelitis-coccyx

For peer review only

N303z	Periostitis without osteomyelitis NOS
N304.	Tuberculosis of spine (Pott's)
N3040	Tuberculosis of cervical spine
N3041	Tuberculosis of thoracic spine
N3042	Tuberculosis of lumbar spine
N3043	Tuberculosis of sacrum/coccyx
N305.	Tuberculosis of limb bones
N3050	Tuberculosis of unspecified limb bone
N3051	Tuberculosis of the upper arm bone
N3052	Tuberculosis of the forearm bone
N3053	Tuberculosis of the pelvic and thigh bones
N3054	Tuberculosis of the lower leg bone
N3055	Tuberculosis of other limb bones
N3056	Tuberculosis of multiple limb bones
N305z	Tuberculosis of limb bones NOS
N306.	Tuberculosis of other bones
N3060	Tuberculosis of bone, site unspecified
N3061	Tuberculosis of the bones of the shoulder region
N3062	Tuberculosis of the bones of the hand
N3063	Tuberculosis of the bones of the ankle and foot
N3064	Tuberculosis of the bones of other sites
N3065	Tuberculosis of the bones of multiple sites
N306z	Tuberculosis of bone NOS
N307.	Osteopathy from poliomyelitis
N3070	Poliomyelitis osteopathy of unspecified site
N3071	Poliomyelitis osteopathy of the shoulder region
N3072	Poliomyelitis osteopathy of the upper arm
N3073	Poliomyelitis osteopathy of the forearm
N3074	Poliomyelitis osteopathy of the hand
N3075	Poliomyelitis osteopathy of the pelvic region and thigh
N3076	Poliomyelitis osteopathy of the lower leg
N3077	Poliomyelitis osteopathy of the ankle and foot
N3078	Poliomyelitis osteopathy of other specified sites
N3079	Poliomyelitis osteopathy of multiple sites
N307z	Poliomyelitis osteopathy NOS
N308.	Subacute osteomyelitis
N3080	Subacute osteomyelitis-cervical spine
N3081	Subacute osteomyelitis-thoracic spine
N3082	Subacute osteomyelitis-lumbar spine
N3083	Subacute osteomyelitis-sacrum
N3084	Subacute osteomyelitis-coccyx
N309.	Subacute osteomyelitis
N30y.	Other infections involving bone
N30y0	Other infections involving bone, site unspecified
N30y1	Other infections involving bone, of the shoulder region
N30y2	Other infections involving bone, of the upper arm
N30y3	Other infections involving bone, of the forearm
N30y4	Other infections involving bone, of the hand

For peer review only

N30y5	Other infections involving bone, of the pelvic region and thigh
N30y6	Other infections involving bone, of the lower leg
N30y7	Other infections involving bone, of the ankle and foot
N30y8	Other infections involving bone, of other specified site
N30y9	Other infections involving bone, of multiple sites
N30yz	Other infections involving bone, NOS
N30z.	Bone infection NOS
N30z0	Bone infection NOS, of unspecified site
N30z1	Bone infection NOS, of the shoulder region
N30z2	Bone infection NOS, of the upper arm
N30z3	Bone infection NOS, of the forearm
N30z4	Bone infection NOS, of the hand
N30z5	Bone infection NOS, of the pelvic/thigh
N30z6	Bone infection NOS, of the lower leg
N30z7	Bone infection NOS, of ankle and foot
N30z8	Bone infection NOS, of other specified site
N30z9	Bone infection NOS, of multiple sites
N30zz	Bone infection NOS
N31..	Osteitis deformans and osteopathies associated with diseases EC
N310.	Osteitis deformans - Paget's disease of the bone
N3100	Paget's disease-cervical spine
N3101	Paget's disease-thoracic spine
N3102	Paget's disease-lumbar spine
N3103	Paget's disease-sacrum
N3104	Paget's disease-coccyx
N3105	Paget's disease-clavicle
N3106	Paget's disease-scapula
N3107	Paget's disease-humerus
N3108	Paget's disease-radius
N3109	Paget's disease-ulna
N310A	Paget's disease-carpal bone
N310B	Paget's disease-metacarpal
N310C	Paget's disease-phalanx of finger or thumb
N310D	Paget's disease-pelvis
N310E	Paget's disease-femur
N310F	Paget's disease-patella
N310G	Paget's disease-tibia
N310H	Paget's disease-fibula
N310J	Paget's disease-calcaneum
N310K	Paget's disease-talus
N310L	Paget's disease-other tarsal bone
N310M	Paget's disease-metatarsal
N310N	Paget's disease-phalanx of toe
N310P	Paget's disease-skull
N310x	Paget's disease-multiple sites
N310y	Paget's disease OS
N310z	Paget's disease NOS
N311.	Osteitis deformans associated with diseases EC

For peer review only

N3110	Osteitis deformans in neoplastic disease
N312.	Hypertrophic pulmonary osteoarthropathy
N31y.	Other bone involvement in diseases EC
N31z.	Bone involvement in diseases EC NOS
N32..	Osteochondropathies
N320.	Juvenile osteochondritis of the spine
N3200	Juvenile osteochondritis of the spine, unspecified
N3201	Scheuermann's disease
N3202	Calve's vertebral osteochondritis
N320z	Juvenile osteochondritis of the spine NOS
N321.	Juvenile osteochondritis of the hip and pelvis
N3210	Juvenile osteochondritis of the hip and pelvis, unspecified
N3211	Perthes' disease - osteochondritis of the femoral head
N3212	Ischiopubic synchondrosis
N3213	Juvenile osteochondritis of the acetabulum
N3214	Juvenile osteochondritis of the iliac crest
N3215	Juvenile osteochondritis of the symphysis pubis
N3216	Coxa plana
N3217	Pseudocoxalgia
N321z	Juvenile osteochondritis of the hip and pelvis NOS
N322.	Non traumatic slipped upper femoral epiphysis
N3220	Non traumatic acute slipped upper femoral epiphysis
N3221	Non traumatic acute-on-chronic slipped upper femoral epiphysis
N3222	Non traumatic chronic slipped upper femoral epiphysis
N323.	Juvenile osteochondritis of the arm and hand
N3230	Juvenile osteochondritis of the arm, unspecified
N3231	Juvenile osteochondritis of the hand, unspecified
N3232	Panner's disease - osteochondritis of capitulum of humerus
N3233	Kienbock's disease - osteochondritis of carpal lunate
N3234	Haas' disease - osteochondritis of head of humerus
N3235	Mauclaire's disease - osteochondritis of metacarpal heads
N3236	Burn's disease - osteochondritis of the lower ulna
N3237	Brailsford's disease - osteochondritis of the radial head
N323z	Juvenile osteochondritis of the arm and hand NOS
N324.	Juvenile osteochondrosis of the leg
N3240	Juvenile osteochondrosis of the leg, unspecified
N3241	Kohler's disease - osteochondrosis of primary patella centre
N3242	Blount's disease - osteochondrosis of proximal tibia
N3243	Juvenile osteochondrosis of the secondary patellar centre
N3244	Osgood-Schlatter's disease - osteochondrosis of tibial tubercle
N324z	Juvenile osteochondrosis of the leg, NOS
N325.	Juvenile osteochondrosis of the foot
N3250	Juvenile osteochondrosis of the foot, unspecified
N3251	Diaz's disease - osteochondrosis of astragalus
N3252	Sever's disease - osteochondrosis of calcaneum
N3253	Freiberg's disease - osteochondrosis of second metatarsal
N3254	Iselin's disease - osteochondrosis of fifth metatarsal
N3255	Haglund's disease - osteochondrosis of os tibiale externum

For peer review only

N3256	Kohler's disease - osteochondrosis of tarsal navicular
N325z	Juvenile osteochondrosis of the foot NOS
N326.	Other juvenile osteochondroses
N3260	Juvenile apophysitis NOS
N3261	Juvenile epiphysitis NOS
N3262	Juvenile osteochondritis NOS
N3263	Juvenile osteochondrosis NOS
N326z	Juvenile osteochondroses NOS
N327.	Osteochondritis dissecans
N3270	Osteochondritis dissecans of patella
N3271	Osteochondritis dissecans of lateral femoral condyle
N3272	Other osteochondritis dissecans of knee
N3273	Osteochondritis dissecans of the humeral head
N3274	Osteochondritis dissecans of the capitellum
N3275	Osteochondritis dissecans of the radial head
N3276	Other osteochondritis dissecans of the elbow
N3277	Osteochondritis dissecans of the wrist
N3278	Osteochondritis dissecans of the femoral head
N3279	Osteochondritis dissecans of the talus
N327y	Osteochondritis dissecans of other site
N328.	Juvenile osteochondrosis of spine
N32y.	Other specified forms of osteochondropathy
N32y0	Adult osteochondrosis of spine
N32y1	Kienbock's disease of adults
N32yz	Other specified osteochondropathy NOS
N32z.	Osteochondropathy NOS
N32z0	Apophysitis NOS
N32z1	Epiphysitis NOS
N32z2	Osteochondritis NOS
N32z3	Osteochondrosis NOS
N32zz	Osteochondropathy NOS
N33..	Other bone and cartilage disorders
N330.	Osteoporosis
N3300	Osteoporosis, unspecified
N3301	Senile osteoporosis
N3302	Postmenopausal osteoporosis
N3303	Idiopathic osteoporosis
N3304	Dissuse osteoporosis
N3305	Drug-induced osteoporosis
N3306	Postoophorectomy osteoporosis
N3307	Postsurgical malabsorption osteoporosis
N3308	Localized osteoporosis - Lequesne
N3309	Osteoporosis in multiple myelomatosis
N330A	Osteoporosis in endocrine disorders
N330B	Vertebral osteoporosis
N330C	Osteoporosis localized to spine
N330D	Osteoporosis due to corticosteroids
N330z	Osteoporosis NOS

For peer review only

N331.	Pathological fracture
N3310	Pathological fracture of thoracic vertebra
N3311	Pathological fracture of lumbar vertebra
N3312	Postoophorectomy osteoporosis with pathological fracture
N3313	Osteoporosis of disuse with pathological fracture
N3314	Postsurgical malabsorption osteoporosis with pathological fracture
N3315	Drug-induced osteoporosis with pathological fracture
N3316	Idiopathic osteoporosis with pathological fracture
N3317	Fracture of bone in neoplastic disease
N3318	Osteoporosis + pathological fracture lumbar vertebrae
N3319	Osteoporosis + pathological fracture thoracic vertebrae
N331A	Osteoporosis + pathological fracture cervical vertebrae
N331B	Postmenopausal osteoporosis with pathological fracture
N331C	Pathological fracture of cervical vertebra
N331D	Collapsed vertebra NOS
N331E	Collapse of cervical vertebra
N331F	Collapse of thoracic vertebra
N331G	Collapse of lumbar vertebra
N331H	Collapse of cervical vertebra due to osteoporosis
N331J	Collapse of lumbar vertebra due to osteoporosis
N331K	Collapse of thoracic vertebra due to osteoporosis
N331L	Collapse of vertebra due to osteoporosis NOS
N331M	Fragility fracture due to unspecified osteoporosis
N331N	Fragility fracture
N331y	Other specified pathological fracture
N331z	Pathological fracture NOS
N332.	Cyst of bone
N3320	Localised bone cyst, unspecified
N3321	Solitary bone cyst
N3322	Aneurysmal bone cyst
N3323	Monostotic fibrous dysplasia
N3324	Fibrous cortical defect
N3325	Brown tumour of hyperparathyroidism
N332z	Cyst of bone NOS
N333.	Hyperostosis of skull
N3330	Hyperostosis interna frontalis
N3331	Leontiasis ossium
N333z	Hyperostosis of skull NOS
N334.	Avascular necrosis of bone
N3340	Avascular necrosis of bone, site unspecified
N3341	Avascular necrosis of the head of humerus
N3342	Avascular necrosis of the head of femur
N3343	Avascular necrosis of the medial femoral condyle
N3344	Avascular necrosis of the talus
N3345	Avascular necrosis of capitellum
N3346	Avascular necrosis of lateral femoral condyle
N3347	Avascular necrosis of other bone
N3348	Idiopathic aseptic necrosis of bone

For peer review only

N3349	Osteonecrosis due to drugs
N334A	Osteonecrosis due to previous trauma
N334B	Osteonecrosis in caisson disease
N334C	Osteonecrosis due to haemoglobinopathy
N334z	Avascular bone necrosis NOS
N335.	Osteitis condensans
N3350	Osteitis condensans ilii
N336.	Tietze's disease
N337.	Algoneurodystrophy
N3370	Disuse atrophy of bone
N3371	Sudek's atrophy
N3372	Algodystrophy of hand
N3373	Algodystrophy of knee
N3374	Algodystrophy of foot
N337z	Algoneurodystrophy NOS
N338.	Malunion and nonunion of fracture
N3380	Malunion of fracture
N3381	Pseudoarthrosis - fracture nonunion
N3382	Hypertrophic non-union of fracture
N3383	Atrophic non-union of fracture
N3384	Angular mal-union of fracture
N3385	Rotational mal-union of fracture
N3386	Delayed union of fracture
N338z	Fracture malunion or nonunion NOS
N339.	Residual foreign body in bone
N33A.	Bone pain
N33A0	Bony pelvic pain
N33A1	Clavicle pain
N33B.	Osteoradionecrosis
N33C.	Complex regional pain syndrome type I
N33z.	Other and unspecified bone or cartilage disorders
N33z0	Bone and cartilage disorders, unspecified
N33z1	Epiphyseal arrest
N33z2	Chondromalacia NOS
N33z3	Diaphysitis
N33z4	Hypertrophy of bone
N33z5	Relapsing polychondritis
N33z6	Compensatory hypertrophy of bone
N33z7	Idiopathic hypertrophy of bone
N33z8	Complete epiphyseal arrest
N33z9	Partial epiphyseal arrest
N33zA	Skeletal fluorosis
N33zB	Chondrolysis
N33zC	Pseudarthrosis after fusion or arthrodesis
N33zD	Osteolysis
N33zE	Costochondritis
N33zF	Disorder of bone, unspecified
N33zG	Disorder of cartilage, unspecified

For peer review only

N33zH	Osteolytic lesion
N33zJ	Chondritis
N33zK	Hajdu-Cheney syndrome
N33zL	Osteitis of symphysis pubis
N33zM	Adynamic bone disease
N33zz	Bone or cartilage disorders NOS
N34..	Flat foot
N340.	Pes planus - acquired
N3400	Hypermobile flat foot
N3401	Rigid flat foot
N3402	Peroneal spastic flat foot
N341.	Talipes planus - acquired
N34z.	Flat foot NOS
N35..	Acquired deformities of toe
N350.	Hallux valgus - acquired
N351.	Hallux varus - acquired
N352.	Hallux rigidus - acquired
N353.	Acquired hammer deformity of great toe
N354.	Other hammer toe - acquired
N355.	Claw toe - acquired
N356.	Clawing of great toe
N357.	Crossover toe
N358.	Mallet toe
N359.	Bunionette
N35A.	Over-riding 5th toe
N35B.	Acquired varus deformity of toe
N35C.	Acquired hammer toe, other than great toe
N35y.	Other acquired toe deformity
N35z.	Acquired toe deformity NOS
N36..	Other acquired limb deformity
N360.	Acquired forearm deformity, excluding fingers
N3600	Acquired forearm deformity, unspecified
N3601	Cubitus valgus - acquired
N3602	Cubitus varus - acquired
N3603	Acquired valgus wrist deformity
N3604	Acquired varus wrist deformity
N3605	Wrist drop - acquired
N3606	Claw hand - acquired
N3607	Club hand - acquired
N360z	Acquired forearm deformity NOS
N361.	Mallet finger
N362.	Other acquired finger deformity
N3620	Acquired finger deformity, unspecified
N3621	Boutonniere finger deformity
N3622	Swan-neck finger deformity
N3623	Flexion deformity of finger
N3624	Extension deformity of finger
N3625	Deviation of finger

For peer review only

N3626	Rotational deformity of finger
N362z	Acquired finger deformity NOS
N363.	Acquired deformities of hip
N3630	Acquired hip deformity, unspecified
N3631	Coxa valga - acquired
N3632	Coxa vara - acquired
N3633	Acquired internal femoral torsion
N3634	Persistent femoral anteversion
N3635	Acquired external femoral torsion
N363z	Acquired hip deformity NOS
N364.	Acquired genu valgum and varum
N3640	Acquired genu valgum
N3641	Acquired genu varum
N364z	Acquired genu valgum or varum NOS
N365.	Genu recurvatum - acquired
N366.	Acquired knee deformity NOS
N3660	Flexion deformity of knee
N367.	Other acquired ankle and foot deformity
N3670	Acquired ankle or foot deformity, unspecified
N3671	Acquired equinovarus - clubfoot
N3672	Acquired equinus foot deformity
N3673	Aquired cavus foot deformity
N3674	Acquired claw foot
N3675	Acquired cavovarus foot deformity
N3676	Other acquired calcaneus deformity
N3677	Acquired talipes NEC
N3678	Acquired varus heel
N3679	Acquired valgus heel
N367A	Plantar flexion of the midtarsal joint
N367B	Plantar flexion contracture of tarsometatarsal joint
N367C	Flexion contracture of metatarsophalangeal joint
N367D	Extension contracture of metatarsophalangeal joint
N367E	Flexion contracture of toe joint
N367F	Acquired plantar-flexed forefoot
N367G	Acquired plantar-flexed first ray
N367H	Acquired plantar-flexed fifth ray
N367J	Acquired dorsiflexed forefoot
N367K	Acquired dorsiflexed first ray
N367L	Acquired supinated forefoot
N367M	Acquired pronated forefoot
N367N	Acquired forefoot adductus
N367P	Acquired forefoot abductus
N367Q	Serpentine foot
N367z	Acquired ankle or foot deformity NOS
N368.	Other knee deformity
N3680	Acquired internal tibial torsion
N3681	Acquired external tibial torsion
N3682	Chronic instability of knee

For peer review only

N369.	Flexion deformity
N36A.	Foot drop
N36y.	Acquired deformity of other limb parts
N36y0	Acquired unequal leg length
N36y1	Acquired unequal arm length
N36y2	Deformity of bone
N36y3	Deformity of clavicle
N36y4	Deformity of scapula
N36y5	Deformity of humerus
N36y6	Deformity of radius
N36y7	Deformity of ulna
N36y8	Deformity of carpal bone
N36y9	Deformity of metacarpal
N36yA	Deformity of phalanx of finger or thumb
N36yB	Deformity of pelvis
N36yC	Deformity of femur
N36yD	Deformity of patella
N36yE	Deformity of tibia
N36yF	Deformity of fibula
N36yG	Deformity of calcaneum
N36yH	Deformity of talus
N36yJ	Deformity of other tarsal bone
N36yK	Deformity of metatarsal
N36yL	Deformity of phalanx of toe
N36yM	Old amputee NOS
N36yN	Acquired radial deviation of hand
N36yz	Acquired limb deformity NEC
N36z.	Acquired limb deformity NOS
N37..	Curvature of spine
N370.	Adolescent postural kyphosis
N371.	Acquired kyphosis
N3710	Acquired postural kyphosis
N3711	Radiation kyphosis
N3712	Post-laminectomy kyphosis
N3713	Kyphosis secondary to other treatment
N371z	Acquired kyphosis NOS
N372.	Acquired lordosis
N3720	Acquired postural lordosis
N3721	Post-laminectomy lordosis
N3722	Other post-surgical lordosis
N372z	Acquired lordosis NOS
N373.	Kyphoscoliosis and scoliosis
N3730	Idiopathic scoliosis
N3731	Idiopathic kyphoscoliosis
N3732	Resolving infantile idiopathic scoliosis
N3733	Progressive infantile idiopathic scoliosis
N3734	Radiation scoliosis
N3735	Thoracogenic scoliosis

For peer review only

N3736	Postural scoliosis
N3737	Adolescent idiopathic scoliosis
N3738	Post-surgical scoliosis
N3739	Scoliosis secondary to other treatment
N373z	Kyphoscoliosis or scoliosis NOS
N374.	Curvature of spine associated with other conditions
N3740	Curvature of spine, unspecified
N3741	Kyphosis associated with other condition
N3742	Lordosis associated with other condition
N3743	Scoliosis associated with other condition
N3744	Kyphosis in skeletal dysplasia
N3745	Neuromuscular kyphosis
N3746	Osteoporotic kyphosis
N3747	Lordosis in skeletal dysplasia
N3748	Lordosis in hip disease
N3749	Neuromuscular lordosis
N374A	Scoliosis in skeletal dysplasia
N374B	Neuromuscular scoliosis
N374C	Scoliosis in neurofibromatosis
N374D	Scoliosis in connective tissue anomalies
N374E	Flatback syndrome
N374W	Lordosis, unspecified
N374X	Other and unspecified kyphosis
N374z	Curvature of spine associated with other conditions NOS
N37y.	Other curvatures of spine
N37z.	Curvature of spine NOS
N37z0	Acquired hunchback
N37zz	Curvature of spine NOS
N38..	Other acquired deformity
N380.	Acquired deformity of nose
N381.	Other acquired head deformity
N382.	Acquired deformity of neck
N383.	Acquired chest and rib deformity
N3830	Acquired chest deformity, unspecified
N3831	Acquired rib deformity, unspecified
N3832	Acquired pectus carinatum
N3833	Acquired pectus excavatum
N383z	Acquired chest or rib deformity NOS
N384.	Acquired spondylolisthesis
N3840	Dysplastic spondylolisthesis
N3841	Isthmic spondylolisthesis
N3842	Degenerative spondylolisthesis
N3843	Pedicular spondylolisthesis
N385.	Acquired deformity of spine NOS
N386.	Acquired deformity of pelvis
N387.	Cauliflower ear
N388.	Spondylolysis
N38y.	Other acquired deformity

For peer review only

N38y0	Acquired clavicle deformity
N38yz	Other acquired deformity NOS
N38z.	Acquired deformity NOS
N39..	Nonallopathic lesions, NEC
N390.	Nonallopathic lesion of the head region
N391.	Nonallopathic lesion of the cervical region
N392.	Nonallopathic lesion of the thoracic region
N393.	Nonallopathic lesion of the lumbar region
N394.	Nonallopathic lesion of the sacral region
N395.	Nonallopathic lesion of the pelvic region
N396.	Nonallopathic lesion of the legs
N397.	Nonallopathic lesion of the arms
N398.	Nonallopathic lesion of the rib cage
N399.	Nonallopathic lesion of the abdomen and other regions
N39z.	Nonallopathic lesion NEC NOS
N3y..	Other specified musculoskeletal disorders
N3y0.	Biomechanical lesions, not elsewhere classified
N3y00	Segmental and somatic dysfunction
N3y01	Subluxation complex (vertebral)
N3y02	Subluxation stenosis of neural canal
N3y03	Osseous stenosis of neural canal
N3y04	Connective tissue stenosis of neural canal
N3y05	Intervertebral disc stenosis of neural canal
N3y06	Osseous and subluxation stenosis of intervertebral foramina
N3y07	Connective tissue and disc stenosis of intervertebral foramina
N3z..	Other musculoskeletal disorder NOS
Ny...	Other specified diseases of musculoskeletal or connective tissue
Ny2..	Repetitive strain injury
Ny20.	Work related upper limb disorder
NyX..	Postprocedural musculoskeletal disorder, unspecified
Nyu..	[X]Additional musculoskeletal and connective tissue disease classification terms
Nyu0.	[X]Infectious arthropathies
Nyu00	[X]Other streptococcal arthritis and polyarthritis
Nyu01	[X]Arthritis and polyarthritis due to other specified bacterial agents
Nyu02	[X]Arthritis in other infectious and parasitic diseases classified elsewhere
Nyu03	[X]Other reactive arthropathies
Nyu04	[X]Other postinfectious arthropathies in diseases classified elsewhere
Nyu05	[X]Reactive arthropathy in other diseases classified elsewhere
Nyu1.	[X]Inflammatory polyarthropathies
Nyu10	[X]Rheumatoid arthritis with involvement of other organs or systems
Nyu11	[X]Other seropositive rheumatoid arthritis
Nyu12	[X]Other specified rheumatoid arthritis
Nyu13	[X]Other psoriatic arthropathies
Nyu14	[X]Other enteropathic arthropathies
Nyu15	[X]Other juvenile arthritis
Nyu16	[X]Juvenile arthritis in other diseases classified elsewhere
Nyu17	[X]Other secondary gout
Nyu18	[X]Other chondrocalcinosis

For peer review only

Nyu19	[X]Other specified crystal arthropathies
Nyu1A	[X]Other specific arthropathies, not elsewhere classified
Nyu1B	[X]Other specified arthritis
Nyu1C	[X]Gouty arthropathy due to enzyme defects and other inherited disorders classified elsewhere
Nyu1D	[X]Crystal arthropathy in other metabolic disorders classified elsewhere
Nyu1E	[X]Arthropathies in other endocrine, nutritional and metabolic disorders
Nyu1F	[X]Arthropathies in other specified diseases classified elsewhere
Nyu1G	[X]Seropositive rheumatoid arthritis, unspecified
Nyu2.	[X]Arthrosis
Nyu20	[X]Other polyarthrosis
Nyu21	[X]Other primary coxarthrosis
Nyu22	[X]Other dysplastic coxarthrosis
Nyu23	[X]Other post-traumatic coxarthrosis
Nyu24	[X]Other secondary coxarthrosis, bilateral
Nyu25	[X]Other primary gonarthrosis
Nyu26	[X]Other post-traumatic gonarthrosis
Nyu27	[X]Other secondary gonarthrosis, bilateral
Nyu28	[X]Other secondary gonarthrosis
Nyu29	[X]Other primary arthrosis of first carpometacarpal joint
Nyu2A	[X]Other post-traumatic arthrosis of first carpometacarpal joint
Nyu2B	[X]Other secondary arthrosis of first carpometacarpal joints, bilateral
Nyu2C	[X]Other secondary arthrosis of first carpometacarpal joint
Nyu2D	[X]Other specified arthrosis
Nyu2E	[X]Other secondary coxarthrosis
Nyu2F	[X]Post-traumatic arthrosis of other joints
Nyu3.	[X]Other joint disorders
Nyu30	[X]Other deformity of hallux (acquired)
Nyu31	[X]Other hammer toe(s) (acquired)
Nyu32	[X]Other deformities of toe(s) (acquired)
Nyu33	[X]Other acquired deformities of ankle and foot
Nyu34	[X]Other specified acquired deformities of limbs
Nyu35	[X]Other derangements of patella
Nyu36	[X]Other disorders of patella
Nyu37	[X]Other meniscus derangements
Nyu38	[X]Other spontaneous disruption of ligament(s) of knee
Nyu39	[X]Other internal derangements of knee
Nyu3A	[X]Other articular cartilage disorders
Nyu3B	[X]Other specific joint derangements, not elsewhere classified
Nyu3C	[X]Other instability of joint
Nyu3D	[X]Other specified joint disorders
Nyu3E	[X]Disorder of patella, unspecified
Nyu4.	[X]Systemic connective tissue disorders
Nyu40	[X]Other conditions related to polyarteritis nodosa
Nyu41	[X]Other giant cell arteritis
Nyu42	[X]Other specified necrotizing vasculopathies
Nyu43	[X]Other forms of systemic lupus erythematosus
Nyu44	[X]Other dermatomyositis

For peer review only

Nyu45	[X]Other forms of systemic sclerosis
Nyu46	[X]Other overlap syndromes
Nyu47	[X]Other systemic diseases of connective tissue
Nyu48	[X]Dermato(poly)myositis in neoplastic disease classified elsewhere
Nyu49	[X]Arthropathy in neoplastic disease classified elsewhere
Nyu4A	[X]Arthropathy in other blood disorders classified elsewhere
Nyu4B	[X]Arthropathy in hypersensitivity reactions classified elsewhere
Nyu4C	[X]Systemic disorders of connective tissue in other diseases classified elsewhere
Nyu4D	[X]Necrotising vasculopathy, unspecified
Nyu4E	[X]Dermatopolymyositis, unspecified
Nyu4F	[X]Mixed connective tissue disease
Nyu5.	[X]Deforming dorsopathies
Nyu50	[X]Other secondary kyphosis
Nyu51	[X]Other and unspecified kyphosis
Nyu52	[X]Other lordosis
Nyu53	[X]Other idiopathic scoliosis
Nyu54	[X]Other secondary scoliosis
Nyu55	[X]Other forms of scoliosis
Nyu56	[X]Other fusion of spine
Nyu57	[X]Other recurrent atlantoaxial subluxation
Nyu58	[X]Other recurrent vertebral subluxation
Nyu59	[X]Other specified deforming dorsopathies
Nyu5A	[X]Lordosis, unspecified
Nyu5B	[X]Spinal osteochondrosis, unspecified
Nyu6.	[X]Spondylopathies
Nyu60	[X]Other infective spondylopathies
Nyu61	[X]Other specified inflammatory spondylopathies
Nyu62	[X]Other spondylosis with myelopathy
Nyu63	[X]Other spondylosis with radiculopathy
Nyu64	[X]Other spondylosis
Nyu65	[X]Other specified spondylopathies
Nyu66	[X]Spondylopathy in other infectious and parasitic diseases classified elsewhere
Nyu67	[X]Collapsed vertebra in diseases classified elsewhere
Nyu68	[X]Spondylopathy in other diseases classified elsewhere
Nyu69	[X]Spondylopathy, unspecified
Nyu7.	[X]Other dorsopathies
Nyu70	[X]Other cervical disc displacement
Nyu71	[X]Other cervical disc degeneration
Nyu72	[X]Other cervical disc disorders
Nyu73	[X]Lumbar and other intervertebral disc disorders with myelopathy
Nyu74	[X]Lumbar and other intervertebral disc disorders with radiculopathy
Nyu75	[X]Other specified intervertebral disc displacement
Nyu76	[X]Other specified intervertebral disc degeneration
Nyu77	[X]Other specified intervertebral disc disorders
Nyu78	[X]Sacrococcygeal disorders, not elsewhere classified
Nyu79	[X]Other specified dorsopathies
Nyu7A	[X]Other dorsalgia
Nyu7B	[X]Cervical disc disorder, unspecified

For peer review only

Nyu8.	[X]Disorders of muscles
Nyu80	[X]Other myositis
Nyu81	[X]Other calcification of muscle
Nyu82	[X]Other ossification of muscle
Nyu83	[X]Other rupture of muscle (nontraumatic)
Nyu84	[X]Muscle wasting and atrophy, not elsewhere classified
Nyu85	[X]Other specified disorders of muscle
Nyu86	[X]Myositis in bacterial diseases classified elsewhere
Nyu87	[X]Myositis in protozoal and parasitic infections classified elsewhere
Nyu88	[X]Myositis in other infectious diseases classified elsewhere
Nyu89	[X]Myositis in sarcoidosis classified elsewhere
Nyu8A	[X]Other disorders of muscle in diseases classified elsewhere
Nyu8B	[X]Disorder of muscle, unspecified
Nyu9.	[X]Disorders of synovium and tendon
Nyu90	[X]Other infective (teno)synovitis
Nyu91	[X]Other synovitis and tenosynovitis
Nyu92	[X]Spontaneous rupture of other tendons
Nyu93	[X]Other contracture of tendon (sheath)
Nyu94	[X]Other specified disorders of synovium and tendon
Nyu95	[X]Synovitis and tenosynovitis in bacterial diseases classified elsewhere
Nyu96	[X]Other disorders of synovium and tendon in diseases classified elsewhere
Nyu97	[X]Synovial hypertrophy, not elsewhere classified
NyuA.	[X]Other soft tissue disorders
NyuA0	[X]Other bursitis of elbow
NyuA1	[X]Other bursitis of knee
NyuA2	[X]Other bursitis of hip
NyuA3	[X]Other soft tissue disorders related to use, overuse and pressure
NyuA4	[X]Other infective bursitis
NyuA5	[X]Other bursal cyst
NyuA6	[X]Other bursitis, not elsewhere classified
NyuA7	[X]Other specified bursopathies
NyuA8	[X]Fasciitis, not elsewhere classified
NyuA9	[X]Other fibroblastic disorders
NyuAA	[X]Other soft tissue disorders in other diseases classified elsewhere
NyuAB	[X]Other shoulder lesions
NyuAC	[X]Other enthesopathies of lower limb, excluding foot
NyuAD	[X]Other enthesopathy of foot
NyuAE	[X]Other enthesopathies, not elsewhere classified
NyuAF	[X]Other specified soft tissue disorders
NyuAG	[X]Unspecified soft tissue disorder related to use, overuse and pressure
NyuAH	[X]Fibroblastic disorder, unspecified
NyuAJ	[X]Enthesopathy of lower limb, unspecified
NyuB.	[X]Disorders of bone density and structure
NyuB0	[X]Other osteoporosis with pathological fracture
NyuB1	[X]Other osteoporosis
NyuB2	[X]Osteoporosis in other disorders classified elsewhere
NyuB3	[X]Other drug-induced osteomalacia in adults
NyuB4	[X]Other adult osteomalacia

For peer review only

NyuB5	[X]Other disorders of continuity of bone
NyuB6	[X]Other cyst of bone
NyuB7	[X]Other specified disorders of bone density and structure
NyuB8	[X]Unspecified osteoporosis with pathological fracture
NyuB9	[X]Adult osteomalacia, unspecified
NyuBA	[X]Disorder of bone density and structure, unspecified
NyuBB	[X]Erosion of bone
NyuBC	[X]Osteopenia
NyuC.	[X]Other osteopathies
NyuC0	[X]Other acute osteomyelitis
NyuC1	[X]Other chronic haematogenous osteomyelitis
NyuC2	[X]Other chronic osteomyelitis
NyuC3	[X]Other osteomyelitis
NyuC4	[X]Other secondary osteonecrosis
NyuC5	[X]Other osteonecrosis
NyuC6	[X]Other disorders of bone development and growth
NyuC7	[X]Other hypertrophic osteoarthropathy
NyuC8	[X]Other specified disorders of bone
NyuC9	[X]Periostitis in other infectious diseases classified elsewhere
NyuCA	[X]Osteopathy in other infectious diseases classified elsewhere
NyuCB	[X]Osteonecrosis due to haemoglobinopathy classified elsewhere
NyuCC	[X]Osteonecrosis in other diseases classified elsewhere
NyuCD	[X]Osteitis deformans in neoplastic diseases classified elsewhere
NyuCE	[X]Fracture of bone in neoplastic diseases classified elsewhere
NyuCF	[X]Osteopathy in other diseases classified elsewhere
NyuD.	[X]Chondropathies
NyuD0	[X]Other juvenile osteochondrosis of hip and pelvis
NyuD1	[X]Other juvenile osteochondrosis of upper limb
NyuD2	[X]Other specified juvenile osteochondrosis
NyuD3	[X]Other specified osteochondropathies
NyuD4	[X]Other specified disorders of cartilage
NyuDE	[X]Disorder of cartilage, unspecified
NyuE.	[X]Other disorders of the musculoskeletal system and connective tissue
NyuE0	[X]Other specified acquired deformities of musculoskeletal system
NyuE1	[X]Fracture of bone following insertion of orthopaedic implant, joint prosthesis, or bone plate
NyuE2	[X]Other postprocedural musculoskeletal disorders
NyuE3	[X]Other biomechanical lesions
NyuE4	[X]Postprocedural musculoskeletal disorder, unspecified
Nz...	Musculoskeletal and connective tissue diseases NOS

Hospital

ALT_CODE	DESCRIPTION
M00	Pyogenic arthritis
M000	Staphylococcal arthritis and polyarthritis
M0000	Staphylococcal arthritis and polyarthritis

For peer review only

M0001	Staphylococcal arthritis and polyarthritis
M0002	Staphylococcal arthritis and polyarthritis
M0003	Staphylococcal arthritis and polyarthritis
M0004	Staphylococcal arthritis and polyarthritis
M0005	Staphylococcal arthritis and polyarthritis
M0006	Staphylococcal arthritis and polyarthritis
M0007	Staphylococcal arthritis and polyarthritis
M0008	Staphylococcal arthritis and polyarthritis
M0009	Staphylococcal arthritis and polyarthritis
M001	Pneumococcal arthritis and polyarthritis
M0010	Pneumococcal arthritis and polyarthritis
M0011	Pneumococcal arthritis and polyarthritis
M0012	Pneumococcal arthritis and polyarthritis
M0013	Pneumococcal arthritis and polyarthritis
M0014	Pneumococcal arthritis and polyarthritis
M0015	Pneumococcal arthritis and polyarthritis
M0016	Pneumococcal arthritis and polyarthritis
M0017	Pneumococcal arthritis and polyarthritis
M0018	Pneumococcal arthritis and polyarthritis
M0019	Pneumococcal arthritis and polyarthritis
M002	Other streptococcal arthritis and polyarthritis
M0020	Other streptococcal arthritis and polyarthritis
M0021	Other streptococcal arthritis and polyarthritis
M0022	Other streptococcal arthritis and polyarthritis
M0023	Other streptococcal arthritis and polyarthritis
M0024	Other streptococcal arthritis and polyarthritis
M0025	Other streptococcal arthritis and polyarthritis
M0026	Other streptococcal arthritis and polyarthritis
M0027	Other streptococcal arthritis and polyarthritis
M0028	Other streptococcal arthritis and polyarthritis
M0029	Other streptococcal arthritis and polyarthritis
M008	Arthritis and polyarthritis due to other specified bacterial agents
M0080	Arthritis and polyarthritis due to other specified bacterial agents
M0081	Arthritis and polyarthritis due to other specified bacterial agents
M0082	Arthritis and polyarthritis due to other specified bacterial agents
M0083	Arthritis and polyarthritis due to other specified bacterial agents
M0084	Arthritis and polyarthritis due to other specified bacterial agents
M0085	Arthritis and polyarthritis due to other specified bacterial agents
M0086	Arthritis and polyarthritis due to other specified bacterial agents
M0087	Arthritis and polyarthritis due to other specified bacterial agents
M0088	Arthritis and polyarthritis due to other specified bacterial agents
M0089	Arthritis and polyarthritis due to other specified bacterial agents
M009	Pyogenic arthritis, unspecified
M0090	Pyogenic arthritis, unspecified
M0091	Pyogenic arthritis, unspecified
M0092	Pyogenic arthritis, unspecified
M0093	Pyogenic arthritis, unspecified
M0094	Pyogenic arthritis, unspecified

For peer review only

M0095	Pyogenic arthritis, unspecified
M0096	Pyogenic arthritis, unspecified
M0097	Pyogenic arthritis, unspecified
M0098	Pyogenic arthritis, unspecified
M0099	Pyogenic arthritis, unspecified
M01	Direct infections of joint in infectious and parasitic diseases classified elsewhere
M010	Meningococcal arthritis
M0100	Meningococcal arthritis
M0101	Meningococcal arthritis
M0102	Meningococcal arthritis
M0103	Meningococcal arthritis
M0104	Meningococcal arthritis
M0105	Meningococcal arthritis
M0106	Meningococcal arthritis
M0107	Meningococcal arthritis
M0108	Meningococcal arthritis
M0109	Meningococcal arthritis
M011	Tuberculous arthritis
M0110	Tuberculous arthritis
M0111	Tuberculous arthritis
M0112	Tuberculous arthritis
M0113	Tuberculous arthritis
M0114	Tuberculous arthritis
M0115	Tuberculous arthritis
M0116	Tuberculous arthritis
M0117	Tuberculous arthritis
M0118	Tuberculous arthritis
M0119	Tuberculous arthritis
M012	Arthritis in Lyme disease
M0120	Arthritis in Lyme disease
M0121	Arthritis in Lyme disease
M0122	Arthritis in Lyme disease
M0123	Arthritis in Lyme disease
M0124	Arthritis in Lyme disease
M0125	Arthritis in Lyme disease
M0126	Arthritis in Lyme disease
M0127	Arthritis in Lyme disease
M0128	Arthritis in Lyme disease
M0129	Arthritis in Lyme disease
M013	Arthritis in other bacterial diseases classified elsewhere
M0130	Arthritis in other bacterial diseases classified elsewhere
M0131	Arthritis in other bacterial diseases classified elsewhere
M0132	Arthritis in other bacterial diseases classified elsewhere
M0133	Arthritis in other bacterial diseases classified elsewhere
M0134	Arthritis in other bacterial diseases classified elsewhere
M0135	Arthritis in other bacterial diseases classified elsewhere
M0136	Arthritis in other bacterial diseases classified elsewhere
M0137	Arthritis in other bacterial diseases classified elsewhere

For peer review only

M0138	Arthritis in other bacterial diseases classified elsewhere
M0139	Arthritis in other bacterial diseases classified elsewhere
M014	Rubella arthritis
M0140	Rubella arthritis
M0141	Rubella arthritis
M0142	Rubella arthritis
M0143	Rubella arthritis
M0144	Rubella arthritis
M0145	Rubella arthritis
M0146	Rubella arthritis
M0147	Rubella arthritis
M0148	Rubella arthritis
M0149	Rubella arthritis
M015	Arthritis in other viral diseases classified elsewhere
M0150	Arthritis in other viral diseases classified elsewhere
M0151	Arthritis in other viral diseases classified elsewhere
M0152	Arthritis in other viral diseases classified elsewhere
M0153	Arthritis in other viral diseases classified elsewhere
M0154	Arthritis in other viral diseases classified elsewhere
M0155	Arthritis in other viral diseases classified elsewhere
M0156	Arthritis in other viral diseases classified elsewhere
M0157	Arthritis in other viral diseases classified elsewhere
M0158	Arthritis in other viral diseases classified elsewhere
M0159	Arthritis in other viral diseases classified elsewhere
M016	Arthritis in mycoses
M0160	Arthritis in mycoses
M0161	Arthritis in mycoses
M0162	Arthritis in mycoses
M0163	Arthritis in mycoses
M0164	Arthritis in mycoses
M0165	Arthritis in mycoses
M0166	Arthritis in mycoses
M0167	Arthritis in mycoses
M0168	Arthritis in mycoses
M0169	Arthritis in mycoses
M018	Arthritis in other infectious and parasitic diseases classified elsewhere
M0180	Arthritis in other infectious and parasitic diseases classified elsewhere
M0181	Arthritis in other infectious and parasitic diseases classified elsewhere
M0182	Arthritis in other infectious and parasitic diseases classified elsewhere
M0183	Arthritis in other infectious and parasitic diseases classified elsewhere
M0184	Arthritis in other infectious and parasitic diseases classified elsewhere
M0185	Arthritis in other infectious and parasitic diseases classified elsewhere
M0186	Arthritis in other infectious and parasitic diseases classified elsewhere
M0187	Arthritis in other infectious and parasitic diseases classified elsewhere
M0188	Arthritis in other infectious and parasitic diseases classified elsewhere
M0189	Arthritis in other infectious and parasitic diseases classified elsewhere
M02	Reactive arthropathies
M020	Arthropathy following intestinal bypass

For peer review only

M0200	Arthropathy following intestinal bypass
M0201	Arthropathy following intestinal bypass
M0202	Arthropathy following intestinal bypass
M0203	Arthropathy following intestinal bypass
M0204	Arthropathy following intestinal bypass
M0205	Arthropathy following intestinal bypass
M0206	Arthropathy following intestinal bypass
M0207	Arthropathy following intestinal bypass
M0208	Arthropathy following intestinal bypass
M0209	Arthropathy following intestinal bypass
M021	Postdysenteric arthropathy
M0210	Postdysenteric arthropathy
M0211	Postdysenteric arthropathy
M0212	Postdysenteric arthropathy
M0213	Postdysenteric arthropathy
M0214	Postdysenteric arthropathy
M0215	Postdysenteric arthropathy
M0216	Postdysenteric arthropathy
M0217	Postdysenteric arthropathy
M0218	Postdysenteric arthropathy
M0219	Postdysenteric arthropathy
M022	Postimmunization arthropathy
M0220	Postimmunization arthropathy
M0221	Postimmunization arthropathy
M0222	Postimmunization arthropathy
M0223	Postimmunization arthropathy
M0224	Postimmunization arthropathy
M0225	Postimmunization arthropathy
M0226	Postimmunization arthropathy
M0227	Postimmunization arthropathy
M0228	Postimmunization arthropathy
M0229	Postimmunization arthropathy
M023	Reiter disease
M023	Reiter's disease
M0230	Reiter disease
M0230	Reiter's disease
M0231	Reiter disease
M0231	Reiter's disease
M0232	Reiter disease
M0232	Reiter's disease
M0233	Reiter disease
M0233	Reiter's disease
M0234	Reiter disease
M0234	Reiter's disease
M0235	Reiter disease
M0235	Reiter's disease
M0236	Reiter disease
M0236	Reiter's disease

For peer review only

M0237	Reiter disease
M0237	Reiter's disease
M0238	Reiter disease
M0238	Reiter's disease
M0239	Reiter disease
M0239	Reiter's disease
M028	Other reactive arthropathies
M0280	Other reactive arthropathies
M0281	Other reactive arthropathies
M0282	Other reactive arthropathies
M0283	Other reactive arthropathies
M0284	Other reactive arthropathies
M0285	Other reactive arthropathies
M0286	Other reactive arthropathies
M0287	Other reactive arthropathies
M0288	Other reactive arthropathies
M0289	Other reactive arthropathies
M029	Reactive arthropathy, unspecified
M0290	Reactive arthropathy, unspecified
M0291	Reactive arthropathy, unspecified
M0292	Reactive arthropathy, unspecified
M0293	Reactive arthropathy, unspecified
M0294	Reactive arthropathy, unspecified
M0295	Reactive arthropathy, unspecified
M0296	Reactive arthropathy, unspecified
M0297	Reactive arthropathy, unspecified
M0298	Reactive arthropathy, unspecified
M0299	Reactive arthropathy, unspecified
M03	Postinfective and reactive arthropathies in diseases classified elsewhere
M030	Postmeningococcal arthritis
M0300	Postmeningococcal arthritis
M0301	Postmeningococcal arthritis
M0302	Postmeningococcal arthritis
M0303	Postmeningococcal arthritis
M0304	Postmeningococcal arthritis
M0305	Postmeningococcal arthritis
M0306	Postmeningococcal arthritis
M0307	Postmeningococcal arthritis
M0308	Postmeningococcal arthritis
M0309	Postmeningococcal arthritis
M031	Postinfective arthropathy in syphilis
M0310	Postinfective arthropathy in syphilis
M0311	Postinfective arthropathy in syphilis
M0312	Postinfective arthropathy in syphilis
M0313	Postinfective arthropathy in syphilis
M0314	Postinfective arthropathy in syphilis
M0315	Postinfective arthropathy in syphilis
M0316	Postinfective arthropathy in syphilis

For peer review only

M0317	Postinfective arthropathy in syphilis
M0318	Postinfective arthropathy in syphilis
M0319	Postinfective arthropathy in syphilis
M032	Other postinfectious arthropathies in diseases classified elsewhere
M0320	Other postinfectious arthropathies in diseases classified elsewhere
M0321	Other postinfectious arthropathies in diseases classified elsewhere
M0322	Other postinfectious arthropathies in diseases classified elsewhere
M0323	Other postinfectious arthropathies in diseases classified elsewhere
M0324	Other postinfectious arthropathies in diseases classified elsewhere
M0325	Other postinfectious arthropathies in diseases classified elsewhere
M0326	Other postinfectious arthropathies in diseases classified elsewhere
M0327	Other postinfectious arthropathies in diseases classified elsewhere
M0328	Other postinfectious arthropathies in diseases classified elsewhere
M0329	Other postinfectious arthropathies in diseases classified elsewhere
M036	Reactive arthropathy in other diseases classified elsewhere
M0360	Reactive arthropathy in other diseases classified elsewhere
M0361	Reactive arthropathy in other diseases classified elsewhere
M0362	Reactive arthropathy in other diseases classified elsewhere
M0363	Reactive arthropathy in other diseases classified elsewhere
M0364	Reactive arthropathy in other diseases classified elsewhere
M0365	Reactive arthropathy in other diseases classified elsewhere
M0366	Reactive arthropathy in other diseases classified elsewhere
M0367	Reactive arthropathy in other diseases classified elsewhere
M0368	Reactive arthropathy in other diseases classified elsewhere
M0369	Reactive arthropathy in other diseases classified elsewhere
M05	Seropositive rheumatoid arthritis
M050	Felty syndrome
M050	Felty's syndrome
M0500	Felty syndrome
M0500	Felty's syndrome
M0501	Felty syndrome
M0501	Felty's syndrome
M0502	Felty syndrome
M0502	Felty's syndrome
M0503	Felty syndrome
M0503	Felty's syndrome
M0504	Felty syndrome
M0504	Felty's syndrome
M0505	Felty syndrome
M0505	Felty's syndrome
M0506	Felty syndrome
M0506	Felty's syndrome
M0507	Felty syndrome
M0507	Felty's syndrome
M0508	Felty syndrome
M0508	Felty's syndrome
M0509	Felty syndrome
M0509	Felty's syndrome

For peer review only

M051	Rheumatoid lung disease
M0510	Rheumatoid lung disease
M0511	Rheumatoid lung disease
M0512	Rheumatoid lung disease
M0513	Rheumatoid lung disease
M0514	Rheumatoid lung disease
M0515	Rheumatoid lung disease
M0516	Rheumatoid lung disease
M0517	Rheumatoid lung disease
M0518	Rheumatoid lung disease
M0519	Rheumatoid lung disease
M052	Rheumatoid vasculitis
M0520	Rheumatoid vasculitis
M0521	Rheumatoid vasculitis
M0522	Rheumatoid vasculitis
M0523	Rheumatoid vasculitis
M0524	Rheumatoid vasculitis
M0525	Rheumatoid vasculitis
M0526	Rheumatoid vasculitis
M0527	Rheumatoid vasculitis
M0528	Rheumatoid vasculitis
M0529	Rheumatoid vasculitis
M053	Rheumatoid arthritis with involvement of other organs and systems
M0530	Rheumatoid arthritis with involvement of other organs and systems
M0531	Rheumatoid arthritis with involvement of other organs and systems
M0532	Rheumatoid arthritis with involvement of other organs and systems
M0533	Rheumatoid arthritis with involvement of other organs and systems
M0534	Rheumatoid arthritis with involvement of other organs and systems
M0535	Rheumatoid arthritis with involvement of other organs and systems
M0536	Rheumatoid arthritis with involvement of other organs and systems
M0537	Rheumatoid arthritis with involvement of other organs and systems
M0538	Rheumatoid arthritis with involvement of other organs and systems
M0539	Rheumatoid arthritis with involvement of other organs and systems
M058	Other seropositive rheumatoid arthritis
M0580	Other seropositive rheumatoid arthritis
M0581	Other seropositive rheumatoid arthritis
M0582	Other seropositive rheumatoid arthritis
M0583	Other seropositive rheumatoid arthritis
M0584	Other seropositive rheumatoid arthritis
M0585	Other seropositive rheumatoid arthritis
M0586	Other seropositive rheumatoid arthritis
M0587	Other seropositive rheumatoid arthritis
M0588	Other seropositive rheumatoid arthritis
M0589	Other seropositive rheumatoid arthritis
M059	Seropositive rheumatoid arthritis, unspecified
M0590	Seropositive rheumatoid arthritis, unspecified
M0591	Seropositive rheumatoid arthritis, unspecified
M0592	Seropositive rheumatoid arthritis, unspecified

For peer review only

M0593	Seropositive rheumatoid arthritis, unspecified
M0594	Seropositive rheumatoid arthritis, unspecified
M0595	Seropositive rheumatoid arthritis, unspecified
M0596	Seropositive rheumatoid arthritis, unspecified
M0597	Seropositive rheumatoid arthritis, unspecified
M0598	Seropositive rheumatoid arthritis, unspecified
M0599	Seropositive rheumatoid arthritis, unspecified
M06	Other rheumatoid arthritis
M060	Seronegative rheumatoid arthritis
M0600	Seronegative rheumatoid arthritis
M0601	Seronegative rheumatoid arthritis
M0602	Seronegative rheumatoid arthritis
M0603	Seronegative rheumatoid arthritis
M0604	Seronegative rheumatoid arthritis
M0605	Seronegative rheumatoid arthritis
M0606	Seronegative rheumatoid arthritis
M0607	Seronegative rheumatoid arthritis
M0608	Seronegative rheumatoid arthritis
M0609	Seronegative rheumatoid arthritis
M061	Adult-onset Still disease
M061	Adult-onset Still's disease
M0610	Adult-onset Still disease
M0610	Adult-onset Still's disease
M0611	Adult-onset Still disease
M0611	Adult-onset Still's disease
M0612	Adult-onset Still disease
M0612	Adult-onset Still's disease
M0613	Adult-onset Still disease
M0613	Adult-onset Still's disease
M0614	Adult-onset Still disease
M0614	Adult-onset Still's disease
M0615	Adult-onset Still disease
M0615	Adult-onset Still's disease
M0616	Adult-onset Still disease
M0616	Adult-onset Still's disease
M0617	Adult-onset Still disease
M0617	Adult-onset Still's disease
M0618	Adult-onset Still disease
M0618	Adult-onset Still's disease
M0619	Adult-onset Still disease
M0619	Adult-onset Still's disease
M062	Rheumatoid bursitis
M0620	Rheumatoid bursitis
M0621	Rheumatoid bursitis
M0622	Rheumatoid bursitis
M0623	Rheumatoid bursitis
M0624	Rheumatoid bursitis
M0625	Rheumatoid bursitis

For peer review only

M0626	Rheumatoid bursitis
M0627	Rheumatoid bursitis
M0628	Rheumatoid bursitis
M0629	Rheumatoid bursitis
M063	Rheumatoid nodule
M0630	Rheumatoid nodule
M0631	Rheumatoid nodule
M0632	Rheumatoid nodule
M0633	Rheumatoid nodule
M0634	Rheumatoid nodule
M0635	Rheumatoid nodule
M0636	Rheumatoid nodule
M0637	Rheumatoid nodule
M0638	Rheumatoid nodule
M0639	Rheumatoid nodule
M064	Inflammatory polyarthropathy
M0640	Inflammatory polyarthropathy
M0641	Inflammatory polyarthropathy
M0642	Inflammatory polyarthropathy
M0643	Inflammatory polyarthropathy
M0644	Inflammatory polyarthropathy
M0645	Inflammatory polyarthropathy
M0646	Inflammatory polyarthropathy
M0647	Inflammatory polyarthropathy
M0648	Inflammatory polyarthropathy
M0649	Inflammatory polyarthropathy
M068	Other specified rheumatoid arthritis
M0680	Other specified rheumatoid arthritis
M0681	Other specified rheumatoid arthritis
M0682	Other specified rheumatoid arthritis
M0683	Other specified rheumatoid arthritis
M0684	Other specified rheumatoid arthritis
M0685	Other specified rheumatoid arthritis
M0686	Other specified rheumatoid arthritis
M0687	Other specified rheumatoid arthritis
M0688	Other specified rheumatoid arthritis
M0689	Other specified rheumatoid arthritis
M069	Rheumatoid arthritis, unspecified
M0690	Rheumatoid arthritis, unspecified
M0691	Rheumatoid arthritis, unspecified
M0692	Rheumatoid arthritis, unspecified
M0693	Rheumatoid arthritis, unspecified
M0694	Rheumatoid arthritis, unspecified
M0695	Rheumatoid arthritis, unspecified
M0696	Rheumatoid arthritis, unspecified
M0697	Rheumatoid arthritis, unspecified
M0698	Rheumatoid arthritis, unspecified
M0699	Rheumatoid arthritis, unspecified

For peer review only

M07	Psoriatic and enteropathic arthropathies
M070	Distal interphalangeal psoriatic arthropathy
M0700	Distal interphalangeal psoriatic arthropathy
M0704	Distal interphalangeal psoriatic arthropathy
M0707	Distal interphalangeal psoriatic arthropathy
M0709	Distal interphalangeal psoriatic arthropathy
M071	Arthritis mutilans
M0710	Arthritis mutilans
M0711	Arthritis mutilans
M0712	Arthritis mutilans
M0713	Arthritis mutilans
M0714	Arthritis mutilans
M0715	Arthritis mutilans
M0716	Arthritis mutilans
M0717	Arthritis mutilans
M0718	Arthritis mutilans
M0719	Arthritis mutilans
M072	Psoriatic spondylitis
M0720	Psoriatic spondylitis
M0721	Psoriatic spondylitis
M0722	Psoriatic spondylitis
M0723	Psoriatic spondylitis
M0724	Psoriatic spondylitis
M0725	Psoriatic spondylitis
M0726	Psoriatic spondylitis
M0727	Psoriatic spondylitis
M0728	Psoriatic spondylitis
M0729	Psoriatic spondylitis
M073	Other psoriatic arthropathies
M0730	Other psoriatic arthropathies
M0731	Other psoriatic arthropathies
M0732	Other psoriatic arthropathies
M0733	Other psoriatic arthropathies
M0734	Other psoriatic arthropathies
M0735	Other psoriatic arthropathies
M0736	Other psoriatic arthropathies
M0737	Other psoriatic arthropathies
M0738	Other psoriatic arthropathies
M0739	Other psoriatic arthropathies
M074	Arthropathy in Crohn disease [regional enteritis]
M074	Arthropathy in Crohn's disease [regional enteritis]
M0740	Arthropathy in Crohn disease [regional enteritis]
M0740	Arthropathy in Crohn's disease [regional enteritis]
M0741	Arthropathy in Crohn disease [regional enteritis]
M0741	Arthropathy in Crohn's disease [regional enteritis]
M0742	Arthropathy in Crohn disease [regional enteritis]
M0742	Arthropathy in Crohn's disease [regional enteritis]
M0743	Arthropathy in Crohn disease [regional enteritis]

For peer review only

M0743	Arthropathy in Crohn's disease [regional enteritis]
M0744	Arthropathy in Crohn disease [regional enteritis]
M0744	Arthropathy in Crohn's disease [regional enteritis]
M0745	Arthropathy in Crohn disease [regional enteritis]
M0745	Arthropathy in Crohn's disease [regional enteritis]
M0746	Arthropathy in Crohn disease [regional enteritis]
M0746	Arthropathy in Crohn's disease [regional enteritis]
M0747	Arthropathy in Crohn disease [regional enteritis]
M0747	Arthropathy in Crohn's disease [regional enteritis]
M0748	Arthropathy in Crohn disease [regional enteritis]
M0748	Arthropathy in Crohn's disease [regional enteritis]
M0749	Arthropathy in Crohn disease [regional enteritis]
M0749	Arthropathy in Crohn's disease [regional enteritis]
M075	Arthropathy in ulcerative colitis
M0750	Arthropathy in ulcerative colitis
M0751	Arthropathy in ulcerative colitis
M0752	Arthropathy in ulcerative colitis
M0753	Arthropathy in ulcerative colitis
M0754	Arthropathy in ulcerative colitis
M0755	Arthropathy in ulcerative colitis
M0756	Arthropathy in ulcerative colitis
M0757	Arthropathy in ulcerative colitis
M0758	Arthropathy in ulcerative colitis
M0759	Arthropathy in ulcerative colitis
M076	Other enteropathic arthropathies
M0760	Other enteropathic arthropathies
M0761	Other enteropathic arthropathies
M0762	Other enteropathic arthropathies
M0763	Other enteropathic arthropathies
M0764	Other enteropathic arthropathies
M0765	Other enteropathic arthropathies
M0766	Other enteropathic arthropathies
M0767	Other enteropathic arthropathies
M0768	Other enteropathic arthropathies
M0769	Other enteropathic arthropathies
M08	Juvenile arthritis
M080	Juvenile rheumatoid arthritis
M0800	Juvenile rheumatoid arthritis
M0801	Juvenile rheumatoid arthritis
M0802	Juvenile rheumatoid arthritis
M0803	Juvenile rheumatoid arthritis
M0804	Juvenile rheumatoid arthritis
M0805	Juvenile rheumatoid arthritis
M0806	Juvenile rheumatoid arthritis
M0807	Juvenile rheumatoid arthritis
M0808	Juvenile rheumatoid arthritis
M0809	Juvenile rheumatoid arthritis
M081	Juvenile ankylosing spondylitis

For peer review only

M0810	Juvenile ankylosing spondylitis
M0811	Juvenile ankylosing spondylitis
M0812	Juvenile ankylosing spondylitis
M0813	Juvenile ankylosing spondylitis
M0814	Juvenile ankylosing spondylitis
M0815	Juvenile ankylosing spondylitis
M0816	Juvenile ankylosing spondylitis
M0817	Juvenile ankylosing spondylitis
M0818	Juvenile ankylosing spondylitis
M0819	Juvenile ankylosing spondylitis
M082	Juvenile arthritis with systemic onset
M0820	Juvenile arthritis with systemic onset
M0821	Juvenile arthritis with systemic onset
M0822	Juvenile arthritis with systemic onset
M0823	Juvenile arthritis with systemic onset
M0824	Juvenile arthritis with systemic onset
M0825	Juvenile arthritis with systemic onset
M0826	Juvenile arthritis with systemic onset
M0827	Juvenile arthritis with systemic onset
M0828	Juvenile arthritis with systemic onset
M0829	Juvenile arthritis with systemic onset
M083	Juvenile polyarthritis (seronegative)
M0830	Juvenile polyarthritis (seronegative)
M0831	Juvenile polyarthritis (seronegative)
M0832	Juvenile polyarthritis (seronegative)
M0833	Juvenile polyarthritis (seronegative)
M0834	Juvenile polyarthritis (seronegative)
M0835	Juvenile polyarthritis (seronegative)
M0836	Juvenile polyarthritis (seronegative)
M0837	Juvenile polyarthritis (seronegative)
M0838	Juvenile polyarthritis (seronegative)
M0839	Juvenile polyarthritis (seronegative)
M084	Pauciarticular juvenile arthritis
M0840	Pauciarticular juvenile arthritis
M0841	Pauciarticular juvenile arthritis
M0842	Pauciarticular juvenile arthritis
M0843	Pauciarticular juvenile arthritis
M0844	Pauciarticular juvenile arthritis
M0845	Pauciarticular juvenile arthritis
M0846	Pauciarticular juvenile arthritis
M0847	Pauciarticular juvenile arthritis
M0848	Pauciarticular juvenile arthritis
M0849	Pauciarticular juvenile arthritis
M088	Other juvenile arthritis
M0880	Other juvenile arthritis
M0881	Other juvenile arthritis
M0882	Other juvenile arthritis
M0883	Other juvenile arthritis

For peer review only

M0884	Other juvenile arthritis
M0885	Other juvenile arthritis
M0886	Other juvenile arthritis
M0887	Other juvenile arthritis
M0888	Other juvenile arthritis
M0889	Other juvenile arthritis
M089	Juvenile arthritis, unspecified
M0890	Juvenile arthritis, unspecified
M0891	Juvenile arthritis, unspecified
M0892	Juvenile arthritis, unspecified
M0893	Juvenile arthritis, unspecified
M0894	Juvenile arthritis, unspecified
M0895	Juvenile arthritis, unspecified
M0896	Juvenile arthritis, unspecified
M0897	Juvenile arthritis, unspecified
M0898	Juvenile arthritis, unspecified
M0899	Juvenile arthritis, unspecified
M09	Juvenile arthritis in diseases classified elsewhere
M090	Juvenile arthritis in psoriasis
M0900	Juvenile arthritis in psoriasis
M0901	Juvenile arthritis in psoriasis
M0902	Juvenile arthritis in psoriasis
M0903	Juvenile arthritis in psoriasis
M0904	Juvenile arthritis in psoriasis
M0905	Juvenile arthritis in psoriasis
M0906	Juvenile arthritis in psoriasis
M0907	Juvenile arthritis in psoriasis
M0908	Juvenile arthritis in psoriasis
M0909	Juvenile arthritis in psoriasis
M091	Juvenile arthritis in Crohn disease [regional enteritis]
M091	Juvenile arthritis in Crohn's disease [regional enteritis]
M0910	Juvenile arthritis in Crohn disease [regional enteritis]
M0910	Juvenile arthritis in Crohn's disease [regional enteritis]
M0911	Juvenile arthritis in Crohn disease [regional enteritis]
M0911	Juvenile arthritis in Crohn's disease [regional enteritis]
M0912	Juvenile arthritis in Crohn disease [regional enteritis]
M0912	Juvenile arthritis in Crohn's disease [regional enteritis]
M0913	Juvenile arthritis in Crohn disease [regional enteritis]
M0913	Juvenile arthritis in Crohn's disease [regional enteritis]
M0914	Juvenile arthritis in Crohn disease [regional enteritis]
M0914	Juvenile arthritis in Crohn's disease [regional enteritis]
M0915	Juvenile arthritis in Crohn disease [regional enteritis]
M0915	Juvenile arthritis in Crohn's disease [regional enteritis]
M0916	Juvenile arthritis in Crohn disease [regional enteritis]
M0916	Juvenile arthritis in Crohn's disease [regional enteritis]
M0917	Juvenile arthritis in Crohn disease [regional enteritis]
M0917	Juvenile arthritis in Crohn's disease [regional enteritis]
M0918	Juvenile arthritis in Crohn disease [regional enteritis]

For peer review only

M0918	Juvenile arthritis in Crohn's disease [regional enteritis]
M0919	Juvenile arthritis in Crohn disease [regional enteritis]
M0919	Juvenile arthritis in Crohn's disease [regional enteritis]
M092	Juvenile arthritis in ulcerative colitis
M0920	Juvenile arthritis in ulcerative colitis
M0921	Juvenile arthritis in ulcerative colitis
M0922	Juvenile arthritis in ulcerative colitis
M0923	Juvenile arthritis in ulcerative colitis
M0924	Juvenile arthritis in ulcerative colitis
M0925	Juvenile arthritis in ulcerative colitis
M0926	Juvenile arthritis in ulcerative colitis
M0927	Juvenile arthritis in ulcerative colitis
M0928	Juvenile arthritis in ulcerative colitis
M0929	Juvenile arthritis in ulcerative colitis
M098	Juvenile arthritis in other diseases classified elsewhere
M0980	Juvenile arthritis in other diseases classified elsewhere
M0981	Juvenile arthritis in other diseases classified elsewhere
M0982	Juvenile arthritis in other diseases classified elsewhere
M0983	Juvenile arthritis in other diseases classified elsewhere
M0984	Juvenile arthritis in other diseases classified elsewhere
M0985	Juvenile arthritis in other diseases classified elsewhere
M0986	Juvenile arthritis in other diseases classified elsewhere
M0987	Juvenile arthritis in other diseases classified elsewhere
M0988	Juvenile arthritis in other diseases classified elsewhere
M0989	Juvenile arthritis in other diseases classified elsewhere
M10	Gout
M100	Idiopathic gout
M1000	Idiopathic gout
M1001	Idiopathic gout
M1002	Idiopathic gout
M1003	Idiopathic gout
M1004	Idiopathic gout
M1005	Idiopathic gout
M1006	Idiopathic gout
M1007	Idiopathic gout
M1008	Idiopathic gout
M1009	Idiopathic gout
M101	Lead-induced gout
M1010	Lead-induced gout
M1011	Lead-induced gout
M1012	Lead-induced gout
M1013	Lead-induced gout
M1014	Lead-induced gout
M1015	Lead-induced gout
M1016	Lead-induced gout
M1017	Lead-induced gout
M1018	Lead-induced gout
M1019	Lead-induced gout

For peer review only

M102	Drug-induced gout
M1020	Drug-induced gout
M1021	Drug-induced gout
M1022	Drug-induced gout
M1023	Drug-induced gout
M1024	Drug-induced gout
M1025	Drug-induced gout
M1026	Drug-induced gout
M1027	Drug-induced gout
M1028	Drug-induced gout
M1029	Drug-induced gout
M103	Gout due to impairment of renal function
M1030	Gout due to impairment of renal function
M1031	Gout due to impairment of renal function
M1032	Gout due to impairment of renal function
M1033	Gout due to impairment of renal function
M1034	Gout due to impairment of renal function
M1035	Gout due to impairment of renal function
M1036	Gout due to impairment of renal function
M1037	Gout due to impairment of renal function
M1038	Gout due to impairment of renal function
M1039	Gout due to impairment of renal function
M104	Other secondary gout
M1040	Other secondary gout
M1041	Other secondary gout
M1042	Other secondary gout
M1043	Other secondary gout
M1044	Other secondary gout
M1045	Other secondary gout
M1046	Other secondary gout
M1047	Other secondary gout
M1048	Other secondary gout
M1049	Other secondary gout
M109	Gout, unspecified
M1090	Gout, unspecified
M1091	Gout, unspecified
M1092	Gout, unspecified
M1093	Gout, unspecified
M1094	Gout, unspecified
M1095	Gout, unspecified
M1096	Gout, unspecified
M1097	Gout, unspecified
M1098	Gout, unspecified
M1099	Gout, unspecified
M11	Other crystal arthropathies
M110	Hydroxyapatite deposition disease
M1100	Hydroxyapatite deposition disease
M1101	Hydroxyapatite deposition disease

For peer review only

M1102	Hydroxyapatite deposition disease
M1103	Hydroxyapatite deposition disease
M1104	Hydroxyapatite deposition disease
M1105	Hydroxyapatite deposition disease
M1106	Hydroxyapatite deposition disease
M1107	Hydroxyapatite deposition disease
M1108	Hydroxyapatite deposition disease
M1109	Hydroxyapatite deposition disease
M111	Familial chondrocalcinosis
M1110	Familial chondrocalcinosis
M1111	Familial chondrocalcinosis
M1112	Familial chondrocalcinosis
M1113	Familial chondrocalcinosis
M1114	Familial chondrocalcinosis
M1115	Familial chondrocalcinosis
M1116	Familial chondrocalcinosis
M1117	Familial chondrocalcinosis
M1118	Familial chondrocalcinosis
M1119	Familial chondrocalcinosis
M112	Other chondrocalcinosis
M1120	Other chondrocalcinosis
M1121	Other chondrocalcinosis
M1122	Other chondrocalcinosis
M1123	Other chondrocalcinosis
M1124	Other chondrocalcinosis
M1125	Other chondrocalcinosis
M1126	Other chondrocalcinosis
M1127	Other chondrocalcinosis
M1128	Other chondrocalcinosis
M1129	Other chondrocalcinosis
M118	Other specified crystal arthropathies
M1180	Other specified crystal arthropathies
M1181	Other specified crystal arthropathies
M1182	Other specified crystal arthropathies
M1183	Other specified crystal arthropathies
M1184	Other specified crystal arthropathies
M1185	Other specified crystal arthropathies
M1186	Other specified crystal arthropathies
M1187	Other specified crystal arthropathies
M1188	Other specified crystal arthropathies
M1189	Other specified crystal arthropathies
M119	Crystal arthropathy, unspecified
M1190	Crystal arthropathy, unspecified
M1191	Crystal arthropathy, unspecified
M1192	Crystal arthropathy, unspecified
M1193	Crystal arthropathy, unspecified
M1194	Crystal arthropathy, unspecified
M1195	Crystal arthropathy, unspecified

For peer review only

M1196	Crystal arthropathy, unspecified
M1197	Crystal arthropathy, unspecified
M1198	Crystal arthropathy, unspecified
M1199	Crystal arthropathy, unspecified
M12	Other specific arthropathies
M120	Chronic postrheumatic arthropathy [Jaccoud]
M1200	Chronic postrheumatic arthropathy [Jaccoud]
M1201	Chronic postrheumatic arthropathy [Jaccoud]
M1202	Chronic postrheumatic arthropathy [Jaccoud]
M1203	Chronic postrheumatic arthropathy [Jaccoud]
M1204	Chronic postrheumatic arthropathy [Jaccoud]
M1205	Chronic postrheumatic arthropathy [Jaccoud]
M1206	Chronic postrheumatic arthropathy [Jaccoud]
M1207	Chronic postrheumatic arthropathy [Jaccoud]
M1208	Chronic postrheumatic arthropathy [Jaccoud]
M1209	Chronic postrheumatic arthropathy [Jaccoud]
M121	Kaschin-Beck disease
M1210	Kaschin-Beck disease
M1211	Kaschin-Beck disease
M1212	Kaschin-Beck disease
M1213	Kaschin-Beck disease
M1214	Kaschin-Beck disease
M1215	Kaschin-Beck disease
M1216	Kaschin-Beck disease
M1217	Kaschin-Beck disease
M1218	Kaschin-Beck disease
M1219	Kaschin-Beck disease
M122	Villonodular synovitis (pigmented)
M1220	Villonodular synovitis (pigmented)
M1221	Villonodular synovitis (pigmented)
M1222	Villonodular synovitis (pigmented)
M1223	Villonodular synovitis (pigmented)
M1224	Villonodular synovitis (pigmented)
M1225	Villonodular synovitis (pigmented)
M1226	Villonodular synovitis (pigmented)
M1227	Villonodular synovitis (pigmented)
M1228	Villonodular synovitis (pigmented)
M1229	Villonodular synovitis (pigmented)
M123	Palindromic rheumatism
M1230	Palindromic rheumatism
M1231	Palindromic rheumatism
M1232	Palindromic rheumatism
M1233	Palindromic rheumatism
M1234	Palindromic rheumatism
M1235	Palindromic rheumatism
M1236	Palindromic rheumatism
M1237	Palindromic rheumatism
M1238	Palindromic rheumatism

For peer review only

M1239	Palindromic rheumatism
M124	Intermittent hydrarthrosis
M1240	Intermittent hydrarthrosis
M1241	Intermittent hydrarthrosis
M1242	Intermittent hydrarthrosis
M1243	Intermittent hydrarthrosis
M1244	Intermittent hydrarthrosis
M1245	Intermittent hydrarthrosis
M1246	Intermittent hydrarthrosis
M1247	Intermittent hydrarthrosis
M1248	Intermittent hydrarthrosis
M1249	Intermittent hydrarthrosis
M125	Traumatic arthropathy
M1250	Traumatic arthropathy
M1251	Traumatic arthropathy
M1252	Traumatic arthropathy
M1253	Traumatic arthropathy
M1254	Traumatic arthropathy
M1255	Traumatic arthropathy
M1256	Traumatic arthropathy
M1257	Traumatic arthropathy
M1258	Traumatic arthropathy
M1259	Traumatic arthropathy
M128	Other specific arthropathies, not elsewhere classified
M1280	Other specific arthropathies, not elsewhere classified
M1281	Other specific arthropathies, not elsewhere classified
M1282	Other specific arthropathies, not elsewhere classified
M1283	Other specific arthropathies, not elsewhere classified
M1284	Other specific arthropathies, not elsewhere classified
M1285	Other specific arthropathies, not elsewhere classified
M1286	Other specific arthropathies, not elsewhere classified
M1287	Other specific arthropathies, not elsewhere classified
M1288	Other specific arthropathies, not elsewhere classified
M1289	Other specific arthropathies, not elsewhere classified
M13	Other arthritis
M130	Polyarthritis, unspecified
M1300	Polyarthritis, unspecified
M1301	Polyarthritis, unspecified
M1302	Polyarthritis, unspecified
M1303	Polyarthritis, unspecified
M1304	Polyarthritis, unspecified
M1305	Polyarthritis, unspecified
M1306	Polyarthritis, unspecified
M1307	Polyarthritis, unspecified
M1308	Polyarthritis, unspecified
M1309	Polyarthritis, unspecified
M131	Monoarthritis, not elsewhere classified
M1310	Monoarthritis, not elsewhere classified

For peer review only

M1311	Monoarthritis, not elsewhere classified
M1312	Monoarthritis, not elsewhere classified
M1313	Monoarthritis, not elsewhere classified
M1314	Monoarthritis, not elsewhere classified
M1315	Monoarthritis, not elsewhere classified
M1316	Monoarthritis, not elsewhere classified
M1317	Monoarthritis, not elsewhere classified
M1318	Monoarthritis, not elsewhere classified
M1319	Monoarthritis, not elsewhere classified
M138	Other specified arthritis
M1380	Other specified arthritis
M1381	Other specified arthritis
M1382	Other specified arthritis
M1383	Other specified arthritis
M1384	Other specified arthritis
M1385	Other specified arthritis
M1386	Other specified arthritis
M1387	Other specified arthritis
M1388	Other specified arthritis
M1389	Other specified arthritis
M139	Arthritis, unspecified
M1390	Arthritis, unspecified
M1391	Arthritis, unspecified
M1392	Arthritis, unspecified
M1393	Arthritis, unspecified
M1394	Arthritis, unspecified
M1395	Arthritis, unspecified
M1396	Arthritis, unspecified
M1397	Arthritis, unspecified
M1398	Arthritis, unspecified
M1399	Arthritis, unspecified
M14	Arthropathies in other diseases classified elsewhere
M140	Gouty arthropathy due to enzyme defects and other inherited disorders
M141	Crystal arthropathy in other metabolic disorders
M142	Diabetic arthropathy
M143	Lipoid dermatoarthritis
M144	Arthropathy in amyloidosis
M145	Arthropathies in other endocrine, nutritional and metabolic disorders
M146	Neuropathic arthropathy
M148	Arthropathies in other specified diseases classified elsewhere
M15	Polyarthrosis
M150	Primary generalized (osteo)arthrosis
M151	Heberden nodes (with arthropathy)
M151	Heberden's nodes (with arthropathy)
M152	Bouchard nodes (with arthropathy)
M152	Bouchard's nodes (with arthropathy)
M153	Secondary multiple arthrosis
M154	Erosive (osteo)arthrosis

For peer review only

M158	Other polyarthrosis
M159	Polyarthrosis, unspecified
M16	Coxarthrosis [arthrosis of hip]
M160	Primary coxarthrosis, bilateral
M161	Other primary coxarthrosis
M162	Coxarthrosis resulting from dysplasia, bilateral
M163	Other dysplastic coxarthrosis
M164	Post-traumatic coxarthrosis, bilateral
M165	Other post-traumatic coxarthrosis
M166	Other secondary coxarthrosis, bilateral
M167	Other secondary coxarthrosis
M169	Coxarthrosis, unspecified
M17	Gonarthrosis [arthrosis of knee]
M170	Primary gonarthrosis, bilateral
M171	Other primary gonarthrosis
M172	Post-traumatic gonarthrosis, bilateral
M173	Other post-traumatic gonarthrosis
M174	Other secondary gonarthrosis, bilateral
M175	Other secondary gonarthrosis
M179	Gonarthrosis, unspecified
M18	Arthrosis of first carpometacarpal joint
M180	Primary arthrosis of first carpometacarpal joints, bilateral
M181	Other primary arthrosis of first carpometacarpal joint
M182	Post-traumatic arthrosis of first carpometacarpal joints, bilateral
M183	Other post-traumatic arthrosis of first carpometacarpal joint
M184	Other secondary arthrosis of first carpometacarpal joints, bilateral
M185	Other secondary arthrosis of first carpometacarpal joint
M189	Arthrosis of first carpometacarpal joint, unspecified
M19	Other arthrosis
M190	Primary arthrosis of other joints
M1900	Primary arthrosis of other joints
M1901	Primary arthrosis of other joints
M1902	Primary arthrosis of other joints
M1903	Primary arthrosis of other joints
M1904	Primary arthrosis of other joints
M1905	Primary arthrosis of other joints
M1907	Primary arthrosis of other joints
M1908	Primary arthrosis of other joints
M1909	Primary arthrosis of other joints
M191	Post-traumatic arthrosis of other joints
M1910	Post-traumatic arthrosis of other joints
M1911	Post-traumatic arthrosis of other joints
M1912	Post-traumatic arthrosis of other joints
M1913	Post-traumatic arthrosis of other joints
M1914	Post-traumatic arthrosis of other joints
M1915	Post-traumatic arthrosis of other joints
M1917	Post-traumatic arthrosis of other joints
M1918	Post-traumatic arthrosis of other joints

For peer review only

M1919	Post-traumatic arthrosis of other joints
M192	Other secondary arthrosis
M1920	Other secondary arthrosis
M1921	Other secondary arthrosis
M1922	Other secondary arthrosis
M1923	Other secondary arthrosis
M1924	Other secondary arthrosis
M1925	Other secondary arthrosis
M1927	Other secondary arthrosis
M1928	Other secondary arthrosis
M1929	Other secondary arthrosis
M198	Other specified arthrosis
M1980	Other specified arthrosis
M1981	Other specified arthrosis
M1982	Other specified arthrosis
M1983	Other specified arthrosis
M1984	Other specified arthrosis
M1985	Other specified arthrosis
M1986	Other specified arthrosis
M1987	Other specified arthrosis
M1988	Other specified arthrosis
M1989	Other specified arthrosis
M199	Arthrosis, unspecified
M1990	Arthrosis, unspecified
M1991	Arthrosis, unspecified
M1992	Arthrosis, unspecified
M1993	Arthrosis, unspecified
M1994	Arthrosis, unspecified
M1995	Arthrosis, unspecified
M1997	Arthrosis, unspecified
M1998	Arthrosis, unspecified
M1999	Arthrosis, unspecified
M20	Acquired deformities of fingers and toes
M200	Deformity of finger(s)
M201	Hallux valgus (acquired)
M202	Hallux rigidus
M203	Other deformity of hallux (acquired)
M204	Other hammer toe(s) (acquired)
M205	Other deformities of toe(s) (acquired)
M206	Acquired deformity of toe(s), unspecified
M21	Other acquired deformities of limbs
M210	Valgus deformity, not elsewhere classified
M2100	Valgus deformity, not elsewhere classified
M2101	Valgus deformity, not elsewhere classified
M2102	Valgus deformity, not elsewhere classified
M2103	Valgus deformity, not elsewhere classified
M2104	Valgus deformity, not elsewhere classified
M2105	Valgus deformity, not elsewhere classified

For peer review only

M2106	Valgus deformity, not elsewhere classified
M2107	Valgus deformity, not elsewhere classified
M2108	Valgus deformity, not elsewhere classified
M2109	Valgus deformity, not elsewhere classified
M211	Varus deformity, not elsewhere classified
M2110	Varus deformity, not elsewhere classified
M2111	Varus deformity, not elsewhere classified
M2112	Varus deformity, not elsewhere classified
M2113	Varus deformity, not elsewhere classified
M2114	Varus deformity, not elsewhere classified
M2115	Varus deformity, not elsewhere classified
M2116	Varus deformity, not elsewhere classified
M2117	Varus deformity, not elsewhere classified
M2118	Varus deformity, not elsewhere classified
M2119	Varus deformity, not elsewhere classified
M212	Flexion deformity
M2120	Flexion deformity
M2121	Flexion deformity
M2122	Flexion deformity
M2123	Flexion deformity
M2124	Flexion deformity
M2125	Flexion deformity
M2126	Flexion deformity
M2127	Flexion deformity
M2128	Flexion deformity
M2129	Flexion deformity
M213	Wrist or foot drop (acquired)
M2130	Wrist or foot drop (acquired)
M2133	Wrist or foot drop (acquired)
M2134	Wrist or foot drop (acquired)
M2137	Wrist or foot drop (acquired)
M214	Flat foot [pes planus] (acquired)
M215	Acquired clawhand, clubhand, clawfoot and clubfoot
M2150	Acquired clawhand, clubhand, clawfoot and clubfoot
M2154	Acquired clawhand, clubhand, clawfoot and clubfoot
M2157	Acquired clawhand, clubhand, clawfoot and clubfoot
M216	Other acquired deformities of ankle and foot
M217	Unequal limb length (acquired)
M2170	Unequal limb length (acquired)
M2172	Unequal limb length (acquired)
M2173	Unequal limb length (acquired)
M2174	Unequal limb length (acquired)
M2175	Unequal limb length (acquired)
M2176	Unequal limb length (acquired)
M2177	Unequal limb length (acquired)
M2179	Unequal limb length (acquired)
M218	Other specified acquired deformities of limbs
M2180	Other specified acquired deformities of limbs

For peer review only

M2181	Other specified acquired deformities of limbs
M2182	Other specified acquired deformities of limbs
M2183	Other specified acquired deformities of limbs
M2184	Other specified acquired deformities of limbs
M2185	Other specified acquired deformities of limbs
M2186	Other specified acquired deformities of limbs
M2187	Other specified acquired deformities of limbs
M2189	Other specified acquired deformities of limbs
M219	Acquired deformity of limb, unspecified
M2190	Acquired deformity of limb, unspecified
M2191	Acquired deformity of limb, unspecified
M2192	Acquired deformity of limb, unspecified
M2193	Acquired deformity of limb, unspecified
M2194	Acquired deformity of limb, unspecified
M2195	Acquired deformity of limb, unspecified
M2196	Acquired deformity of limb, unspecified
M2197	Acquired deformity of limb, unspecified
M2199	Acquired deformity of limb, unspecified
M22	Disorders of patella
M220	Recurrent dislocation of patella
M221	Recurrent subluxation of patella
M222	Patellofemoral disorders
M223	Other derangements of patella
M224	Chondromalacia patellae
M228	Other disorders of patella
M229	Disorder of patella, unspecified
M23	Internal derangement of knee
M230	Cystic meniscus
M2300	Cystic meniscus
M2301	Cystic meniscus
M2302	Cystic meniscus
M2303	Cystic meniscus
M2304	Cystic meniscus
M2305	Cystic meniscus
M2306	Cystic meniscus
M2309	Cystic meniscus
M231	Discoid meniscus (congenital)
M2310	Discoid meniscus (congenital)
M2311	Discoid meniscus (congenital)
M2312	Discoid meniscus (congenital)
M2313	Discoid meniscus (congenital)
M2314	Discoid meniscus (congenital)
M2315	Discoid meniscus (congenital)
M2316	Discoid meniscus (congenital)
M2319	Discoid meniscus (congenital)
M232	Derangement of meniscus due to old tear or injury
M2320	Derangement of meniscus due to old tear or injury
M2321	Derangement of meniscus due to old tear or injury

For peer review only

M2322	Derangement of meniscus due to old tear or injury
M2323	Derangement of meniscus due to old tear or injury
M2324	Derangement of meniscus due to old tear or injury
M2325	Derangement of meniscus due to old tear or injury
M2326	Derangement of meniscus due to old tear or injury
M2329	Derangement of meniscus due to old tear or injury
M233	Other meniscus derangements
M2330	Other meniscus derangements
M2331	Other meniscus derangements
M2332	Other meniscus derangements
M2333	Other meniscus derangements
M2334	Other meniscus derangements
M2335	Other meniscus derangements
M2336	Other meniscus derangements
M2339	Other meniscus derangements
M234	Loose body in knee
M235	Chronic instability of knee
M2350	Chronic instability of knee
M2351	Chronic instability of knee
M2352	Chronic instability of knee
M2353	Chronic instability of knee
M2354	Chronic instability of knee
M2355	Chronic instability of knee
M2356	Chronic instability of knee
M2357	Chronic instability of knee
M2359	Chronic instability of knee
M236	Other spontaneous disruption of ligament(s) of knee
M2360	Other spontaneous disruption of ligament(s) of knee
M2361	Other spontaneous disruption of ligament(s) of knee
M2362	Other spontaneous disruption of ligament(s) of knee
M2363	Other spontaneous disruption of ligament(s) of knee
M2364	Other spontaneous disruption of ligament(s) of knee
M2367	Other spontaneous disruption of ligament(s) of knee
M2369	Other spontaneous disruption of ligament(s) of knee
M238	Other internal derangements of knee
M2380	Other internal derangements of knee
M2381	Other internal derangements of knee
M2382	Other internal derangements of knee
M2383	Other internal derangements of knee
M2384	Other internal derangements of knee
M2385	Other internal derangements of knee
M2386	Other internal derangements of knee
M2387	Other internal derangements of knee
M2389	Other internal derangements of knee
M239	Internal derangement of knee, unspecified
M2390	Internal derangement of knee, unspecified
M2391	Internal derangement of knee, unspecified
M2392	Internal derangement of knee, unspecified

For peer review only

M2393	Internal derangement of knee, unspecified
M2394	Internal derangement of knee, unspecified
M2395	Internal derangement of knee, unspecified
M2396	Internal derangement of knee, unspecified
M2397	Internal derangement of knee, unspecified
M2399	Internal derangement of knee, unspecified
M24	Other specific joint derangements
M240	Loose body in joint
M2400	Loose body in joint
M2401	Loose body in joint
M2402	Loose body in joint
M2403	Loose body in joint
M2404	Loose body in joint
M2405	Loose body in joint
M2407	Loose body in joint
M2408	Loose body in joint
M2409	Loose body in joint
M241	Other articular cartilage disorders
M2410	Other articular cartilage disorders
M2411	Other articular cartilage disorders
M2412	Other articular cartilage disorders
M2413	Other articular cartilage disorders
M2414	Other articular cartilage disorders
M2415	Other articular cartilage disorders
M2416	Other articular cartilage disorders
M2417	Other articular cartilage disorders
M2418	Other articular cartilage disorders
M2419	Other articular cartilage disorders
M242	Disorder of ligament
M2420	Disorder of ligament
M2421	Disorder of ligament
M2422	Disorder of ligament
M2423	Disorder of ligament
M2424	Disorder of ligament
M2425	Disorder of ligament
M2426	Disorder of ligament
M2427	Disorder of ligament
M2428	Disorder of ligament
M2429	Disorder of ligament
M243	Pathological dislocation and subluxation of joint, not elsewhere classified
M2430	Pathological dislocation and subluxation of joint, not elsewhere classified
M2431	Pathological dislocation and subluxation of joint, not elsewhere classified
M2432	Pathological dislocation and subluxation of joint, not elsewhere classified
M2433	Pathological dislocation and subluxation of joint, not elsewhere classified
M2434	Pathological dislocation and subluxation of joint, not elsewhere classified
M2435	Pathological dislocation and subluxation of joint, not elsewhere classified
M2436	Pathological dislocation and subluxation of joint, not elsewhere classified
M2437	Pathological dislocation and subluxation of joint, not elsewhere classified

For peer review only

M2438	Pathological dislocation and subluxation of joint, not elsewhere classified
M2439	Pathological dislocation and subluxation of joint, not elsewhere classified
M244	Recurrent dislocation and subluxation of joint
M2440	Recurrent dislocation and subluxation of joint
M2441	Recurrent dislocation and subluxation of joint
M2442	Recurrent dislocation and subluxation of joint
M2443	Recurrent dislocation and subluxation of joint
M2444	Recurrent dislocation and subluxation of joint
M2445	Recurrent dislocation and subluxation of joint
M2446	Recurrent dislocation and subluxation of joint
M2447	Recurrent dislocation and subluxation of joint
M2448	Recurrent dislocation and subluxation of joint
M2449	Recurrent dislocation and subluxation of joint
M245	Contracture of joint
M2450	Contracture of joint
M2451	Contracture of joint
M2452	Contracture of joint
M2453	Contracture of joint
M2454	Contracture of joint
M2455	Contracture of joint
M2456	Contracture of joint
M2457	Contracture of joint
M2458	Contracture of joint
M2459	Contracture of joint
M246	Ankylosis of joint
M2460	Ankylosis of joint
M2461	Ankylosis of joint
M2462	Ankylosis of joint
M2463	Ankylosis of joint
M2464	Ankylosis of joint
M2465	Ankylosis of joint
M2466	Ankylosis of joint
M2467	Ankylosis of joint
M2468	Ankylosis of joint
M2469	Ankylosis of joint
M247	Protrusio acetabuli
M248	Other specific joint derangements, not elsewhere classified
M2480	Other specific joint derangements, not elsewhere classified
M2481	Other specific joint derangements, not elsewhere classified
M2482	Other specific joint derangements, not elsewhere classified
M2483	Other specific joint derangements, not elsewhere classified
M2484	Other specific joint derangements, not elsewhere classified
M2485	Other specific joint derangements, not elsewhere classified
M2486	Other specific joint derangements, not elsewhere classified
M2487	Other specific joint derangements, not elsewhere classified
M2488	Other specific joint derangements, not elsewhere classified
M2489	Other specific joint derangements, not elsewhere classified
M249	Joint derangement, unspecified

For peer review only

M2490	Joint derangement, unspecified
M2491	Joint derangement, unspecified
M2492	Joint derangement, unspecified
M2493	Joint derangement, unspecified
M2494	Joint derangement, unspecified
M2495	Joint derangement, unspecified
M2496	Joint derangement, unspecified
M2497	Joint derangement, unspecified
M2498	Joint derangement, unspecified
M2499	Joint derangement, unspecified
M25	Other joint disorders, not elsewhere classified
M250	Haemarthrosis
M2500	Haemarthrosis
M2501	Haemarthrosis
M2502	Haemarthrosis
M2503	Haemarthrosis
M2504	Haemarthrosis
M2505	Haemarthrosis
M2506	Haemarthrosis
M2507	Haemarthrosis
M2508	Haemarthrosis
M2509	Haemarthrosis
M251	Fistula of joint
M2510	Fistula of joint
M2511	Fistula of joint
M2512	Fistula of joint
M2513	Fistula of joint
M2514	Fistula of joint
M2515	Fistula of joint
M2516	Fistula of joint
M2517	Fistula of joint
M2518	Fistula of joint
M2519	Fistula of joint
M252	Flail joint
M2520	Flail joint
M2521	Flail joint
M2522	Flail joint
M2523	Flail joint
M2524	Flail joint
M2525	Flail joint
M2526	Flail joint
M2527	Flail joint
M2528	Flail joint
M2529	Flail joint
M253	Other instability of joint
M2530	Other instability of joint
M2531	Other instability of joint
M2532	Other instability of joint

For peer review only

M2533	Other instability of joint
M2534	Other instability of joint
M2535	Other instability of joint
M2536	Other instability of joint
M2537	Other instability of joint
M2538	Other instability of joint
M2539	Other instability of joint
M254	Effusion of joint
M2540	Effusion of joint
M2541	Effusion of joint
M2542	Effusion of joint
M2543	Effusion of joint
M2544	Effusion of joint
M2545	Effusion of joint
M2546	Effusion of joint
M2547	Effusion of joint
M2548	Effusion of joint
M2549	Effusion of joint
M255	Pain in joint
M2550	Pain in joint
M2551	Pain in joint
M2552	Pain in joint
M2553	Pain in joint
M2554	Pain in joint
M2555	Pain in joint
M2556	Pain in joint
M2557	Pain in joint
M2558	Pain in joint
M2559	Pain in joint
M256	Stiffness of joint, not elsewhere classified
M2560	Stiffness of joint, not elsewhere classified
M2561	Stiffness of joint, not elsewhere classified
M2562	Stiffness of joint, not elsewhere classified
M2563	Stiffness of joint, not elsewhere classified
M2564	Stiffness of joint, not elsewhere classified
M2565	Stiffness of joint, not elsewhere classified
M2566	Stiffness of joint, not elsewhere classified
M2567	Stiffness of joint, not elsewhere classified
M2568	Stiffness of joint, not elsewhere classified
M2569	Stiffness of joint, not elsewhere classified
M257	Osteophyte
M2570	Osteophyte
M2571	Osteophyte
M2572	Osteophyte
M2573	Osteophyte
M2574	Osteophyte
M2575	Osteophyte
M2576	Osteophyte

For peer review only

M2577	Osteophyte
M2578	Osteophyte
M2579	Osteophyte
M258	Other specified joint disorders
M2580	Other specified joint disorders
M2581	Other specified joint disorders
M2582	Other specified joint disorders
M2583	Other specified joint disorders
M2584	Other specified joint disorders
M2585	Other specified joint disorders
M2586	Other specified joint disorders
M2587	Other specified joint disorders
M2588	Other specified joint disorders
M2589	Other specified joint disorders
M259	Joint disorder, unspecified
M2590	Joint disorder, unspecified
M2591	Joint disorder, unspecified
M2592	Joint disorder, unspecified
M2593	Joint disorder, unspecified
M2594	Joint disorder, unspecified
M2595	Joint disorder, unspecified
M2596	Joint disorder, unspecified
M2597	Joint disorder, unspecified
M2598	Joint disorder, unspecified
M2599	Joint disorder, unspecified
M30	Polyarteritis nodosa and related conditions
M300	Polyarteritis nodosa
M301	Polyarteritis with lung involvement [Churg-Strauss]
M302	Juvenile polyarteritis
M303	Mucocutaneous lymph node syndrome [Kawasaki]
M308	Other conditions related to polyarteritis nodosa
M31	Other necrotizing vasculopathies
M310	Hypersensitivity angiitis
M311	Thrombotic microangiopathy
M312	Lethal midline granuloma
M313	Wegener granulomatosis
M313	Wegener's granulomatosis
M314	Aortic arch syndrome [Takayasu]
M315	Giant cell arteritis with polymyalgia rheumatica
M316	Other giant cell arteritis
M317	Microscopic polyangiitis
M318	Other specified necrotizing vasculopathies
M319	Necrotizing vasculopathy, unspecified
M32	Systemic lupus erythematosus
M320	Drug-induced systemic lupus erythematosus
M321	Systemic lupus erythematosus with organ or system involvement
M328	Other forms of systemic lupus erythematosus
M329	Systemic lupus erythematosus, unspecified

For peer review only

M33	Dermatopolymyositis
M330	Juvenile dermatomyositis
M331	Other dermatomyositis
M332	Polymyositis
M339	Dermatopolymyositis, unspecified
M34	Systemic sclerosis
M340	Progressive systemic sclerosis
M341	CR(E)ST syndrome
M342	Systemic sclerosis induced by drugs and chemicals
M348	Other forms of systemic sclerosis
M349	Systemic sclerosis, unspecified
M35	Other systemic involvement of connective tissue
M350	Sicca syndrome [Sj”gren]
M351	Other overlap syndromes
M352	Beh‡et disease
M352	Beh‡et's disease
M353	Polymyalgia rheumatica
M354	Diffuse (eosinophilic) fasciitis
M355	Multifocal fibrosclerosis
M356	Relapsing panniculitis [Weber-Christian]
M357	Hypermobility syndrome
M358	Other specified systemic involvement of connective tissue
M359	Systemic involvement of connective tissue, unspecified
M36	Systemic disorders of connective tissue in diseases classified elsewhere
M360	Dermato(poly)myositis in neoplastic disease
M361	Arthropathy in neoplastic disease
M362	Haemophilic arthropathy
M363	Arthropathy in other blood disorders
M364	Arthropathy in hypersensitivity reactions classified elsewhere
M368	Systemic disorders of connective tissue in other diseases classified elsewhere
M40	Kyphosis and lordosis
M400	Postural kyphosis
M4000	Postural kyphosis
M4001	Postural kyphosis
M4002	Postural kyphosis
M4003	Postural kyphosis
M4004	Postural kyphosis
M4005	Postural kyphosis
M4006	Postural kyphosis
M4007	Postural kyphosis
M4008	Postural kyphosis
M4009	Postural kyphosis
M401	Other secondary kyphosis
M4010	Other secondary kyphosis
M4011	Other secondary kyphosis
M4012	Other secondary kyphosis
M4013	Other secondary kyphosis
M4014	Other secondary kyphosis

For peer review only

M4015	Other secondary kyphosis
M4016	Other secondary kyphosis
M4017	Other secondary kyphosis
M4018	Other secondary kyphosis
M4019	Other secondary kyphosis
M402	Other and unspecified kyphosis
M4020	Other and unspecified kyphosis
M4021	Other and unspecified kyphosis
M4022	Other and unspecified kyphosis
M4023	Other and unspecified kyphosis
M4024	Other and unspecified kyphosis
M4025	Other and unspecified kyphosis
M4026	Other and unspecified kyphosis
M4027	Other and unspecified kyphosis
M4028	Other and unspecified kyphosis
M4029	Other and unspecified kyphosis
M403	Flatback syndrome
M4030	Flatback syndrome
M4031	Flatback syndrome
M4032	Flatback syndrome
M4033	Flatback syndrome
M4034	Flatback syndrome
M4035	Flatback syndrome
M4036	Flatback syndrome
M4037	Flatback syndrome
M4038	Flatback syndrome
M4039	Flatback syndrome
M404	Other lordosis
M4040	Other lordosis
M4041	Other lordosis
M4042	Other lordosis
M4043	Other lordosis
M4044	Other lordosis
M4045	Other lordosis
M4046	Other lordosis
M4047	Other lordosis
M4048	Other lordosis
M4049	Other lordosis
M405	Lordosis, unspecified
M4050	Lordosis, unspecified
M4051	Lordosis, unspecified
M4052	Lordosis, unspecified
M4053	Lordosis, unspecified
M4054	Lordosis, unspecified
M4055	Lordosis, unspecified
M4056	Lordosis, unspecified
M4057	Lordosis, unspecified
M4058	Lordosis, unspecified

For peer review only

M4059	Lordosis, unspecified
M41	Scoliosis
M410	Infantile idiopathic scoliosis
M4100	Infantile idiopathic scoliosis
M4101	Infantile idiopathic scoliosis
M4102	Infantile idiopathic scoliosis
M4103	Infantile idiopathic scoliosis
M4104	Infantile idiopathic scoliosis
M4105	Infantile idiopathic scoliosis
M4106	Infantile idiopathic scoliosis
M4107	Infantile idiopathic scoliosis
M4108	Infantile idiopathic scoliosis
M4109	Infantile idiopathic scoliosis
M411	Juvenile idiopathic scoliosis
M4110	Juvenile idiopathic scoliosis
M4111	Juvenile idiopathic scoliosis
M4112	Juvenile idiopathic scoliosis
M4113	Juvenile idiopathic scoliosis
M4114	Juvenile idiopathic scoliosis
M4115	Juvenile idiopathic scoliosis
M4116	Juvenile idiopathic scoliosis
M4117	Juvenile idiopathic scoliosis
M4118	Juvenile idiopathic scoliosis
M4119	Juvenile idiopathic scoliosis
M412	Other idiopathic scoliosis
M4120	Other idiopathic scoliosis
M4121	Other idiopathic scoliosis
M4122	Other idiopathic scoliosis
M4123	Other idiopathic scoliosis
M4124	Other idiopathic scoliosis
M4125	Other idiopathic scoliosis
M4126	Other idiopathic scoliosis
M4127	Other idiopathic scoliosis
M4128	Other idiopathic scoliosis
M4129	Other idiopathic scoliosis
M413	Thoracogenic scoliosis
M4130	Thoracogenic scoliosis
M4133	Thoracogenic scoliosis
M4134	Thoracogenic scoliosis
M4135	Thoracogenic scoliosis
M414	Neuromuscular scoliosis
M4140	Neuromuscular scoliosis
M4141	Neuromuscular scoliosis
M4142	Neuromuscular scoliosis
M4143	Neuromuscular scoliosis
M4144	Neuromuscular scoliosis
M4145	Neuromuscular scoliosis
M4146	Neuromuscular scoliosis

For peer review only

M4147	Neuromuscular scoliosis
M4148	Neuromuscular scoliosis
M4149	Neuromuscular scoliosis
M415	Other secondary scoliosis
M4150	Other secondary scoliosis
M4151	Other secondary scoliosis
M4152	Other secondary scoliosis
M4153	Other secondary scoliosis
M4154	Other secondary scoliosis
M4155	Other secondary scoliosis
M4156	Other secondary scoliosis
M4157	Other secondary scoliosis
M4158	Other secondary scoliosis
M4159	Other secondary scoliosis
M418	Other forms of scoliosis
M4180	Other forms of scoliosis
M4181	Other forms of scoliosis
M4182	Other forms of scoliosis
M4183	Other forms of scoliosis
M4184	Other forms of scoliosis
M4185	Other forms of scoliosis
M4186	Other forms of scoliosis
M4187	Other forms of scoliosis
M4188	Other forms of scoliosis
M4189	Other forms of scoliosis
M419	Scoliosis, unspecified
M4190	Scoliosis, unspecified
M4191	Scoliosis, unspecified
M4192	Scoliosis, unspecified
M4193	Scoliosis, unspecified
M4194	Scoliosis, unspecified
M4195	Scoliosis, unspecified
M4196	Scoliosis, unspecified
M4197	Scoliosis, unspecified
M4198	Scoliosis, unspecified
M4199	Scoliosis, unspecified
M42	Spinal osteochondrosis
M420	Juvenile osteochondrosis of spine
M4200	Juvenile osteochondrosis of spine
M4201	Juvenile osteochondrosis of spine
M4202	Juvenile osteochondrosis of spine
M4203	Juvenile osteochondrosis of spine
M4204	Juvenile osteochondrosis of spine
M4205	Juvenile osteochondrosis of spine
M4206	Juvenile osteochondrosis of spine
M4207	Juvenile osteochondrosis of spine
M4208	Juvenile osteochondrosis of spine
M4209	Juvenile osteochondrosis of spine

For peer review only

M421	Adult osteochondrosis of spine
M4210	Adult osteochondrosis of spine
M4211	Adult osteochondrosis of spine
M4212	Adult osteochondrosis of spine
M4213	Adult osteochondrosis of spine
M4214	Adult osteochondrosis of spine
M4215	Adult osteochondrosis of spine
M4216	Adult osteochondrosis of spine
M4217	Adult osteochondrosis of spine
M4218	Adult osteochondrosis of spine
M4219	Adult osteochondrosis of spine
M429	Spinal osteochondrosis, unspecified
M4290	Spinal osteochondrosis, unspecified
M4291	Spinal osteochondrosis, unspecified
M4292	Spinal osteochondrosis, unspecified
M4293	Spinal osteochondrosis, unspecified
M4294	Spinal osteochondrosis, unspecified
M4295	Spinal osteochondrosis, unspecified
M4296	Spinal osteochondrosis, unspecified
M4297	Spinal osteochondrosis, unspecified
M4298	Spinal osteochondrosis, unspecified
M4299	Spinal osteochondrosis, unspecified
M43	Other deforming dorsopathies
M430	Spondylolysis
M4300	Spondylolysis
M4301	Spondylolysis
M4302	Spondylolysis
M4303	Spondylolysis
M4304	Spondylolysis
M4305	Spondylolysis
M4306	Spondylolysis
M4307	Spondylolysis
M4308	Spondylolysis
M4309	Spondylolysis
M431	Spondylolisthesis
M4310	Spondylolisthesis
M4311	Spondylolisthesis
M4312	Spondylolisthesis
M4313	Spondylolisthesis
M4314	Spondylolisthesis
M4315	Spondylolisthesis
M4316	Spondylolisthesis
M4317	Spondylolisthesis
M4318	Spondylolisthesis
M4319	Spondylolisthesis
M432	Other fusion of spine
M4320	Other fusion of spine
M4321	Other fusion of spine

For peer review only

M4322	Other fusion of spine
M4323	Other fusion of spine
M4324	Other fusion of spine
M4325	Other fusion of spine
M4326	Other fusion of spine
M4327	Other fusion of spine
M4328	Other fusion of spine
M4329	Other fusion of spine
M433	Recurrent atlantoaxial subluxation with myelopathy
M4330	Recurrent atlantoaxial subluxation with myelopathy
M4331	Recurrent atlantoaxial subluxation with myelopathy
M4332	Recurrent atlantoaxial subluxation with myelopathy
M4333	Recurrent atlantoaxial subluxation with myelopathy
M4334	Recurrent atlantoaxial subluxation with myelopathy
M4335	Recurrent atlantoaxial subluxation with myelopathy
M4336	Recurrent atlantoaxial subluxation with myelopathy
M4337	Recurrent atlantoaxial subluxation with myelopathy
M4338	Recurrent atlantoaxial subluxation with myelopathy
M4339	Recurrent atlantoaxial subluxation with myelopathy
M434	Other recurrent atlantoaxial subluxation
M435	Other recurrent vertebral subluxation
M4350	Other recurrent vertebral subluxation
M4352	Other recurrent vertebral subluxation
M4353	Other recurrent vertebral subluxation
M4354	Other recurrent vertebral subluxation
M4355	Other recurrent vertebral subluxation
M4356	Other recurrent vertebral subluxation
M4357	Other recurrent vertebral subluxation
M4358	Other recurrent vertebral subluxation
M4359	Other recurrent vertebral subluxation
M436	Torticollis
M438	Other specified deforming dorsopathies
M4380	Other specified deforming dorsopathies
M4381	Other specified deforming dorsopathies
M4382	Other specified deforming dorsopathies
M4383	Other specified deforming dorsopathies
M4384	Other specified deforming dorsopathies
M4385	Other specified deforming dorsopathies
M4386	Other specified deforming dorsopathies
M4387	Other specified deforming dorsopathies
M4388	Other specified deforming dorsopathies
M4389	Other specified deforming dorsopathies
M439	Deforming dorsopathy, unspecified
M4390	Deforming dorsopathy, unspecified
M4391	Deforming dorsopathy, unspecified
M4392	Deforming dorsopathy, unspecified
M4393	Deforming dorsopathy, unspecified
M4394	Deforming dorsopathy, unspecified

For peer review only

M4395	Deforming dorsopathy, unspecified
M4396	Deforming dorsopathy, unspecified
M4397	Deforming dorsopathy, unspecified
M4398	Deforming dorsopathy, unspecified
M4399	Deforming dorsopathy, unspecified
M45X	Ankylosing spondylitis
M45X0	Ankylosing spondylitis
M45X1	Ankylosing spondylitis
M45X2	Ankylosing spondylitis
M45X3	Ankylosing spondylitis
M45X4	Ankylosing spondylitis
M45X5	Ankylosing spondylitis
M45X6	Ankylosing spondylitis
M45X7	Ankylosing spondylitis
M45X8	Ankylosing spondylitis
M45X9	Ankylosing spondylitis
M46	Other inflammatory spondylopathies
M460	Spinal enthesopathy
M4600	Spinal enthesopathy
M4601	Spinal enthesopathy
M4602	Spinal enthesopathy
M4603	Spinal enthesopathy
M4604	Spinal enthesopathy
M4605	Spinal enthesopathy
M4606	Spinal enthesopathy
M4607	Spinal enthesopathy
M4608	Spinal enthesopathy
M4609	Spinal enthesopathy
M461	Sacroiliitis, not elsewhere classified
M462	Osteomyelitis of vertebra
M4620	Osteomyelitis of vertebra
M4621	Osteomyelitis of vertebra
M4622	Osteomyelitis of vertebra
M4623	Osteomyelitis of vertebra
M4624	Osteomyelitis of vertebra
M4625	Osteomyelitis of vertebra
M4626	Osteomyelitis of vertebra
M4627	Osteomyelitis of vertebra
M4628	Osteomyelitis of vertebra
M4629	Osteomyelitis of vertebra
M463	Infection of intervertebral disc (pyogenic)
M4630	Infection of intervertebral disc (pyogenic)
M4631	Infection of intervertebral disc (pyogenic)
M4632	Infection of intervertebral disc (pyogenic)
M4633	Infection of intervertebral disc (pyogenic)
M4634	Infection of intervertebral disc (pyogenic)
M4635	Infection of intervertebral disc (pyogenic)
M4636	Infection of intervertebral disc (pyogenic)

For peer review only

M4637	Infection of intervertebral disc (pyogenic)
M4638	Infection of intervertebral disc (pyogenic)
M4639	Infection of intervertebral disc (pyogenic)
M464	Discitis, unspecified
M4640	Discitis, unspecified
M4641	Discitis, unspecified
M4642	Discitis, unspecified
M4643	Discitis, unspecified
M4644	Discitis, unspecified
M4645	Discitis, unspecified
M4646	Discitis, unspecified
M4647	Discitis, unspecified
M4648	Discitis, unspecified
M4649	Discitis, unspecified
M465	Other infective spondylopathies
M4650	Other infective spondylopathies
M4651	Other infective spondylopathies
M4652	Other infective spondylopathies
M4653	Other infective spondylopathies
M4654	Other infective spondylopathies
M4655	Other infective spondylopathies
M4656	Other infective spondylopathies
M4657	Other infective spondylopathies
M4658	Other infective spondylopathies
M4659	Other infective spondylopathies
M468	Other specified inflammatory spondylopathies
M4680	Other specified inflammatory spondylopathies
M4681	Other specified inflammatory spondylopathies
M4682	Other specified inflammatory spondylopathies
M4683	Other specified inflammatory spondylopathies
M4684	Other specified inflammatory spondylopathies
M4685	Other specified inflammatory spondylopathies
M4686	Other specified inflammatory spondylopathies
M4687	Other specified inflammatory spondylopathies
M4688	Other specified inflammatory spondylopathies
M4689	Other specified inflammatory spondylopathies
M469	Inflammatory spondylopathy, unspecified
M4690	Inflammatory spondylopathy, unspecified
M4691	Inflammatory spondylopathy, unspecified
M4692	Inflammatory spondylopathy, unspecified
M4693	Inflammatory spondylopathy, unspecified
M4694	Inflammatory spondylopathy, unspecified
M4695	Inflammatory spondylopathy, unspecified
M4696	Inflammatory spondylopathy, unspecified
M4697	Inflammatory spondylopathy, unspecified
M4698	Inflammatory spondylopathy, unspecified
M4699	Inflammatory spondylopathy, unspecified
M47	Spondylosis

For peer review only

M470	Anterior spinal and vertebral artery compression syndromes
M4700	Anterior spinal and vertebral artery compression syndromes
M4701	Anterior spinal and vertebral artery compression syndromes
M4702	Anterior spinal and vertebral artery compression syndromes
M4703	Anterior spinal and vertebral artery compression syndromes
M4704	Anterior spinal and vertebral artery compression syndromes
M4705	Anterior spinal and vertebral artery compression syndromes
M4706	Anterior spinal and vertebral artery compression syndromes
M4707	Anterior spinal and vertebral artery compression syndromes
M4708	Anterior spinal and vertebral artery compression syndromes
M4709	Anterior spinal and vertebral artery compression syndromes
M471	Other spondylosis with myelopathy
M4710	Other spondylosis with myelopathy
M4711	Other spondylosis with myelopathy
M4712	Other spondylosis with myelopathy
M4713	Other spondylosis with myelopathy
M4714	Other spondylosis with myelopathy
M4715	Other spondylosis with myelopathy
M4716	Other spondylosis with myelopathy
M4717	Other spondylosis with myelopathy
M4718	Other spondylosis with myelopathy
M4719	Other spondylosis with myelopathy
M472	Other spondylosis with radiculopathy
M4720	Other spondylosis with radiculopathy
M4721	Other spondylosis with radiculopathy
M4722	Other spondylosis with radiculopathy
M4723	Other spondylosis with radiculopathy
M4724	Other spondylosis with radiculopathy
M4725	Other spondylosis with radiculopathy
M4726	Other spondylosis with radiculopathy
M4727	Other spondylosis with radiculopathy
M4728	Other spondylosis with radiculopathy
M4729	Other spondylosis with radiculopathy
M478	Other spondylosis
M4780	Other spondylosis
M4781	Other spondylosis
M4782	Other spondylosis
M4783	Other spondylosis
M4784	Other spondylosis
M4785	Other spondylosis
M4786	Other spondylosis
M4787	Other spondylosis
M4788	Other spondylosis
M4789	Other spondylosis
M479	Spondylosis, unspecified
M4790	Spondylosis, unspecified
M4791	Spondylosis, unspecified
M4792	Spondylosis, unspecified

For peer review only

M4793	Spondylosis, unspecified
M4794	Spondylosis, unspecified
M4795	Spondylosis, unspecified
M4796	Spondylosis, unspecified
M4797	Spondylosis, unspecified
M4798	Spondylosis, unspecified
M4799	Spondylosis, unspecified
M48	Other spondylopathies
M480	Spinal stenosis
M4800	Spinal stenosis
M4801	Spinal stenosis
M4802	Spinal stenosis
M4803	Spinal stenosis
M4804	Spinal stenosis
M4805	Spinal stenosis
M4806	Spinal stenosis
M4807	Spinal stenosis
M4808	Spinal stenosis
M4809	Spinal stenosis
M481	Ankylosing hyperostosis [Forestier]
M4810	Ankylosing hyperostosis [Forestier]
M4811	Ankylosing hyperostosis [Forestier]
M4812	Ankylosing hyperostosis [Forestier]
M4813	Ankylosing hyperostosis [Forestier]
M4814	Ankylosing hyperostosis [Forestier]
M4815	Ankylosing hyperostosis [Forestier]
M4816	Ankylosing hyperostosis [Forestier]
M4817	Ankylosing hyperostosis [Forestier]
M4818	Ankylosing hyperostosis [Forestier]
M4819	Ankylosing hyperostosis [Forestier]
M482	Kissing spine
M4820	Kissing spine
M4821	Kissing spine
M4822	Kissing spine
M4823	Kissing spine
M4824	Kissing spine
M4825	Kissing spine
M4826	Kissing spine
M4827	Kissing spine
M4828	Kissing spine
M4829	Kissing spine
M483	Traumatic spondylopathy
M4830	Traumatic spondylopathy
M4831	Traumatic spondylopathy
M4832	Traumatic spondylopathy
M4833	Traumatic spondylopathy
M4834	Traumatic spondylopathy
M4835	Traumatic spondylopathy

For peer review only

M4836	Traumatic spondylopathy
M4837	Traumatic spondylopathy
M4838	Traumatic spondylopathy
M4839	Traumatic spondylopathy
M484	Fatigue fracture of vertebra
M4840	Fatigue fracture of vertebra
M4841	Fatigue fracture of vertebra
M4842	Fatigue fracture of vertebra
M4843	Fatigue fracture of vertebra
M4844	Fatigue fracture of vertebra
M4845	Fatigue fracture of vertebra
M4846	Fatigue fracture of vertebra
M4847	Fatigue fracture of vertebra
M4848	Fatigue fracture of vertebra
M4849	Fatigue fracture of vertebra
M485	Collapsed vertebra, not elsewhere classified
M4850	Collapsed vertebra, not elsewhere classified
M4851	Collapsed vertebra, not elsewhere classified
M4852	Collapsed vertebra, not elsewhere classified
M4853	Collapsed vertebra, not elsewhere classified
M4854	Collapsed vertebra, not elsewhere classified
M4855	Collapsed vertebra, not elsewhere classified
M4856	Collapsed vertebra, not elsewhere classified
M4857	Collapsed vertebra, not elsewhere classified
M4858	Collapsed vertebra, not elsewhere classified
M4859	Collapsed vertebra, not elsewhere classified
M488	Other specified spondylopathies
M4880	Other specified spondylopathies
M4881	Other specified spondylopathies
M4882	Other specified spondylopathies
M4883	Other specified spondylopathies
M4884	Other specified spondylopathies
M4885	Other specified spondylopathies
M4886	Other specified spondylopathies
M4887	Other specified spondylopathies
M4888	Other specified spondylopathies
M4889	Other specified spondylopathies
M489	Spondylopathy, unspecified
M4890	Spondylopathy, unspecified
M4891	Spondylopathy, unspecified
M4892	Spondylopathy, unspecified
M4893	Spondylopathy, unspecified
M4894	Spondylopathy, unspecified
M4895	Spondylopathy, unspecified
M4896	Spondylopathy, unspecified
M4897	Spondylopathy, unspecified
M4898	Spondylopathy, unspecified
M4899	Spondylopathy, unspecified

For peer review only

M49	Spondylopathies in diseases classified elsewhere
M490	Tuberculosis of spine
M4900	Tuberculosis of spine
M4901	Tuberculosis of spine
M4902	Tuberculosis of spine
M4903	Tuberculosis of spine
M4904	Tuberculosis of spine
M4905	Tuberculosis of spine
M4906	Tuberculosis of spine
M4907	Tuberculosis of spine
M4908	Tuberculosis of spine
M4909	Tuberculosis of spine
M491	Brucella spondylitis
M4910	Brucella spondylitis
M4911	Brucella spondylitis
M4912	Brucella spondylitis
M4913	Brucella spondylitis
M4914	Brucella spondylitis
M4915	Brucella spondylitis
M4916	Brucella spondylitis
M4917	Brucella spondylitis
M4918	Brucella spondylitis
M4919	Brucella spondylitis
M492	Enterobacterial spondylitis
M4920	Enterobacterial spondylitis
M4921	Enterobacterial spondylitis
M4922	Enterobacterial spondylitis
M4923	Enterobacterial spondylitis
M4924	Enterobacterial spondylitis
M4925	Enterobacterial spondylitis
M4926	Enterobacterial spondylitis
M4927	Enterobacterial spondylitis
M4928	Enterobacterial spondylitis
M4929	Enterobacterial spondylitis
M493	Spondylopathy in other infectious and parasitic diseases classified elsewhere
M4930	Spondylopathy in other infectious and parasitic diseases classified elsewhere
M4931	Spondylopathy in other infectious and parasitic diseases classified elsewhere
M4932	Spondylopathy in other infectious and parasitic diseases classified elsewhere
M4933	Spondylopathy in other infectious and parasitic diseases classified elsewhere
M4934	Spondylopathy in other infectious and parasitic diseases classified elsewhere
M4935	Spondylopathy in other infectious and parasitic diseases classified elsewhere
M4936	Spondylopathy in other infectious and parasitic diseases classified elsewhere
M4937	Spondylopathy in other infectious and parasitic diseases classified elsewhere
M4938	Spondylopathy in other infectious and parasitic diseases classified elsewhere
M4939	Spondylopathy in other infectious and parasitic diseases classified elsewhere
M494	Neuropathic spondylopathy
M4940	Neuropathic spondylopathy
M4941	Neuropathic spondylopathy

For peer review only

M4942	Neuropathic spondylopathy
M4943	Neuropathic spondylopathy
M4944	Neuropathic spondylopathy
M4945	Neuropathic spondylopathy
M4946	Neuropathic spondylopathy
M4947	Neuropathic spondylopathy
M4948	Neuropathic spondylopathy
M4949	Neuropathic spondylopathy
M495	Collapsed vertebra in diseases classified elsewhere
M4950	Collapsed vertebra in diseases classified elsewhere
M4951	Collapsed vertebra in diseases classified elsewhere
M4952	Collapsed vertebra in diseases classified elsewhere
M4953	Collapsed vertebra in diseases classified elsewhere
M4954	Collapsed vertebra in diseases classified elsewhere
M4955	Collapsed vertebra in diseases classified elsewhere
M4956	Collapsed vertebra in diseases classified elsewhere
M4957	Collapsed vertebra in diseases classified elsewhere
M4958	Collapsed vertebra in diseases classified elsewhere
M4959	Collapsed vertebra in diseases classified elsewhere
M498	Spondylopathy in other diseases classified elsewhere
M4980	Spondylopathy in other diseases classified elsewhere
M4981	Spondylopathy in other diseases classified elsewhere
M4982	Spondylopathy in other diseases classified elsewhere
M4983	Spondylopathy in other diseases classified elsewhere
M4984	Spondylopathy in other diseases classified elsewhere
M4985	Spondylopathy in other diseases classified elsewhere
M4986	Spondylopathy in other diseases classified elsewhere
M4987	Spondylopathy in other diseases classified elsewhere
M4988	Spondylopathy in other diseases classified elsewhere
M4989	Spondylopathy in other diseases classified elsewhere
M50	Cervical disc disorders
M500	Cervical disc disorder with myelopathy
M501	Cervical disc disorder with radiculopathy
M502	Other cervical disc displacement
M503	Other cervical disc degeneration
M508	Other cervical disc disorders
M509	Cervical disc disorder, unspecified
M51	Other intervertebral disc disorders
M510	Lumbar and other intervertebral disc disorders with myelopathy
M511	Lumbar and other intervertebral disc disorders with radiculopathy
M512	Other specified intervertebral disc displacement
M513	Other specified intervertebral disc degeneration
M514	Schmorl nodes
M514	Schmorl's nodes
M518	Other specified intervertebral disc disorders
M519	Intervertebral disc disorder, unspecified
M53	Other dorsopathies, not elsewhere classified
M530	Cervicocranial syndrome

For peer review only

M5300	Cervicocranial syndrome
M5301	Cervicocranial syndrome
M5302	Cervicocranial syndrome
M5303	Cervicocranial syndrome
M531	Cervicobrachial syndrome
M5310	Cervicobrachial syndrome
M5311	Cervicobrachial syndrome
M5312	Cervicobrachial syndrome
M5313	Cervicobrachial syndrome
M532	Spinal instabilities
M5320	Spinal instabilities
M5321	Spinal instabilities
M5322	Spinal instabilities
M5323	Spinal instabilities
M5324	Spinal instabilities
M5325	Spinal instabilities
M5326	Spinal instabilities
M5327	Spinal instabilities
M5328	Spinal instabilities
M5329	Spinal instabilities
M533	Sacrococcygeal disorders, not elsewhere classified
M538	Other specified dorsopathies
M5380	Other specified dorsopathies
M5381	Other specified dorsopathies
M5382	Other specified dorsopathies
M5383	Other specified dorsopathies
M5384	Other specified dorsopathies
M5385	Other specified dorsopathies
M5386	Other specified dorsopathies
M5387	Other specified dorsopathies
M5388	Other specified dorsopathies
M5389	Other specified dorsopathies
M539	Dorsopathy, unspecified
M5390	Dorsopathy, unspecified
M5391	Dorsopathy, unspecified
M5392	Dorsopathy, unspecified
M5393	Dorsopathy, unspecified
M5394	Dorsopathy, unspecified
M5395	Dorsopathy, unspecified
M5396	Dorsopathy, unspecified
M5397	Dorsopathy, unspecified
M5398	Dorsopathy, unspecified
M5399	Dorsopathy, unspecified
M54	Dorsalgia
M540	Panniculitis affecting regions of neck and back
M5400	Panniculitis affecting regions of neck and back
M5401	Panniculitis affecting regions of neck and back
M5402	Panniculitis affecting regions of neck and back

For peer review only

M5403	Panniculitis affecting regions of neck and back
M5404	Panniculitis affecting regions of neck and back
M5405	Panniculitis affecting regions of neck and back
M5406	Panniculitis affecting regions of neck and back
M5407	Panniculitis affecting regions of neck and back
M5408	Panniculitis affecting regions of neck and back
M5409	Panniculitis affecting regions of neck and back
M541	Radiculopathy
M5410	Radiculopathy
M5411	Radiculopathy
M5412	Radiculopathy
M5413	Radiculopathy
M5414	Radiculopathy
M5415	Radiculopathy
M5416	Radiculopathy
M5417	Radiculopathy
M5418	Radiculopathy
M5419	Radiculopathy
M542	Cervicalgia
M5421	Cervicalgia
M5422	Cervicalgia
M5423	Cervicalgia
M543	Sciatica
M5430	Sciatica
M5435	Sciatica
M5436	Sciatica
M5437	Sciatica
M5438	Sciatica
M5439	Sciatica
M544	Lumbago with sciatica
M5440	Lumbago with sciatica
M5445	Lumbago with sciatica
M5446	Lumbago with sciatica
M5447	Lumbago with sciatica
M5448	Lumbago with sciatica
M5449	Lumbago with sciatica
M545	Low back pain
M5450	Low back pain
M5455	Low back pain
M5456	Low back pain
M5457	Low back pain
M5458	Low back pain
M5459	Low back pain
M546	Pain in thoracic spine
M5460	Pain in thoracic spine
M5463	Pain in thoracic spine
M5464	Pain in thoracic spine
M5465	Pain in thoracic spine

For peer review only

M548	Other dorsalgia
M5480	Other dorsalgia
M5481	Other dorsalgia
M5482	Other dorsalgia
M5483	Other dorsalgia
M5484	Other dorsalgia
M5485	Other dorsalgia
M5486	Other dorsalgia
M5487	Other dorsalgia
M5488	Other dorsalgia
M5489	Other dorsalgia
M549	Dorsalgia, unspecified
M5490	Dorsalgia, unspecified
M5491	Dorsalgia, unspecified
M5492	Dorsalgia, unspecified
M5493	Dorsalgia, unspecified
M5494	Dorsalgia, unspecified
M5495	Dorsalgia, unspecified
M5496	Dorsalgia, unspecified
M5497	Dorsalgia, unspecified
M5498	Dorsalgia, unspecified
M5499	Dorsalgia, unspecified
M60	Myositis
M600	Infective myositis
M6000	Infective myositis
M6001	Infective myositis
M6002	Infective myositis
M6003	Infective myositis
M6004	Infective myositis
M6005	Infective myositis
M6006	Infective myositis
M6007	Infective myositis
M6008	Infective myositis
M6009	Infective myositis
M601	Interstitial myositis
M6010	Interstitial myositis
M6011	Interstitial myositis
M6012	Interstitial myositis
M6013	Interstitial myositis
M6014	Interstitial myositis
M6015	Interstitial myositis
M6016	Interstitial myositis
M6017	Interstitial myositis
M6018	Interstitial myositis
M6019	Interstitial myositis
M602	Foreign body granuloma of soft tissue, not elsewhere classified
M6020	Foreign body granuloma of soft tissue, not elsewhere classified
M6021	Foreign body granuloma of soft tissue, not elsewhere classified

For peer review only

M6022	Foreign body granuloma of soft tissue, not elsewhere classified
M6023	Foreign body granuloma of soft tissue, not elsewhere classified
M6024	Foreign body granuloma of soft tissue, not elsewhere classified
M6025	Foreign body granuloma of soft tissue, not elsewhere classified
M6026	Foreign body granuloma of soft tissue, not elsewhere classified
M6027	Foreign body granuloma of soft tissue, not elsewhere classified
M6028	Foreign body granuloma of soft tissue, not elsewhere classified
M6029	Foreign body granuloma of soft tissue, not elsewhere classified
M608	Other myositis
M6080	Other myositis
M6081	Other myositis
M6082	Other myositis
M6083	Other myositis
M6084	Other myositis
M6085	Other myositis
M6086	Other myositis
M6087	Other myositis
M6088	Other myositis
M6089	Other myositis
M609	Myositis, unspecified
M6090	Myositis, unspecified
M6091	Myositis, unspecified
M6092	Myositis, unspecified
M6093	Myositis, unspecified
M6094	Myositis, unspecified
M6095	Myositis, unspecified
M6096	Myositis, unspecified
M6097	Myositis, unspecified
M6098	Myositis, unspecified
M6099	Myositis, unspecified
M61	Calcification and ossification of muscle
M610	Myositis ossificans traumatica
M6100	Myositis ossificans traumatica
M6101	Myositis ossificans traumatica
M6102	Myositis ossificans traumatica
M6103	Myositis ossificans traumatica
M6104	Myositis ossificans traumatica
M6105	Myositis ossificans traumatica
M6106	Myositis ossificans traumatica
M6107	Myositis ossificans traumatica
M6108	Myositis ossificans traumatica
M6109	Myositis ossificans traumatica
M611	Myositis ossificans progressiva
M6110	Myositis ossificans progressiva
M6111	Myositis ossificans progressiva
M6112	Myositis ossificans progressiva
M6113	Myositis ossificans progressiva
M6114	Myositis ossificans progressiva

For peer review only

M6115	Myositis ossificans progressiva
M6116	Myositis ossificans progressiva
M6117	Myositis ossificans progressiva
M6118	Myositis ossificans progressiva
M6119	Myositis ossificans progressiva
M612	Paralytic calcification and ossification of muscle
M6120	Paralytic calcification and ossification of muscle
M6121	Paralytic calcification and ossification of muscle
M6122	Paralytic calcification and ossification of muscle
M6123	Paralytic calcification and ossification of muscle
M6124	Paralytic calcification and ossification of muscle
M6125	Paralytic calcification and ossification of muscle
M6126	Paralytic calcification and ossification of muscle
M6127	Paralytic calcification and ossification of muscle
M6128	Paralytic calcification and ossification of muscle
M6129	Paralytic calcification and ossification of muscle
M613	Calcification and ossification of muscles associated with burns
M6130	Calcification and ossification of muscles associated with burns
M6131	Calcification and ossification of muscles associated with burns
M6132	Calcification and ossification of muscles associated with burns
M6133	Calcification and ossification of muscles associated with burns
M6134	Calcification and ossification of muscles associated with burns
M6135	Calcification and ossification of muscles associated with burns
M6136	Calcification and ossification of muscles associated with burns
M6137	Calcification and ossification of muscles associated with burns
M6138	Calcification and ossification of muscles associated with burns
M6139	Calcification and ossification of muscles associated with burns
M614	Other calcification of muscle
M6140	Other calcification of muscle
M6141	Other calcification of muscle
M6142	Other calcification of muscle
M6143	Other calcification of muscle
M6144	Other calcification of muscle
M6145	Other calcification of muscle
M6146	Other calcification of muscle
M6147	Other calcification of muscle
M6148	Other calcification of muscle
M6149	Other calcification of muscle
M615	Other ossification of muscle
M6150	Other ossification of muscle
M6151	Other ossification of muscle
M6152	Other ossification of muscle
M6153	Other ossification of muscle
M6154	Other ossification of muscle
M6155	Other ossification of muscle
M6156	Other ossification of muscle
M6157	Other ossification of muscle
M6158	Other ossification of muscle

For peer review only

M6159	Other ossification of muscle
M619	Calcification and ossification of muscle, unspecified
M6190	Calcification and ossification of muscle, unspecified
M6191	Calcification and ossification of muscle, unspecified
M6192	Calcification and ossification of muscle, unspecified
M6193	Calcification and ossification of muscle, unspecified
M6194	Calcification and ossification of muscle, unspecified
M6195	Calcification and ossification of muscle, unspecified
M6196	Calcification and ossification of muscle, unspecified
M6197	Calcification and ossification of muscle, unspecified
M6198	Calcification and ossification of muscle, unspecified
M6199	Calcification and ossification of muscle, unspecified
M62	Other disorders of muscle
M620	Diastasis of muscle
M6200	Diastasis of muscle
M6201	Diastasis of muscle
M6202	Diastasis of muscle
M6203	Diastasis of muscle
M6204	Diastasis of muscle
M6205	Diastasis of muscle
M6206	Diastasis of muscle
M6207	Diastasis of muscle
M6208	Diastasis of muscle
M6209	Diastasis of muscle
M621	Other rupture of muscle (nontraumatic)
M6210	Other rupture of muscle (nontraumatic)
M6211	Other rupture of muscle (nontraumatic)
M6212	Other rupture of muscle (nontraumatic)
M6213	Other rupture of muscle (nontraumatic)
M6214	Other rupture of muscle (nontraumatic)
M6215	Other rupture of muscle (nontraumatic)
M6216	Other rupture of muscle (nontraumatic)
M6217	Other rupture of muscle (nontraumatic)
M6218	Other rupture of muscle (nontraumatic)
M6219	Other rupture of muscle (nontraumatic)
M622	Ischaemic infarction of muscle
M6220	Ischaemic infarction of muscle
M6221	Ischaemic infarction of muscle
M6222	Ischaemic infarction of muscle
M6223	Ischaemic infarction of muscle
M6224	Ischaemic infarction of muscle
M6225	Ischaemic infarction of muscle
M6226	Ischaemic infarction of muscle
M6227	Ischaemic infarction of muscle
M6228	Ischaemic infarction of muscle
M6229	Ischaemic infarction of muscle
M623	Immobility syndrome (paraplegic)
M6230	Immobility syndrome (paraplegic)

For peer review only

M6231	Immobility syndrome (paraplegic)
M6232	Immobility syndrome (paraplegic)
M6233	Immobility syndrome (paraplegic)
M6234	Immobility syndrome (paraplegic)
M6235	Immobility syndrome (paraplegic)
M6236	Immobility syndrome (paraplegic)
M6237	Immobility syndrome (paraplegic)
M6238	Immobility syndrome (paraplegic)
M6239	Immobility syndrome (paraplegic)
M624	Contracture of muscle
M6240	Contracture of muscle
M6241	Contracture of muscle
M6242	Contracture of muscle
M6243	Contracture of muscle
M6244	Contracture of muscle
M6245	Contracture of muscle
M6246	Contracture of muscle
M6247	Contracture of muscle
M6248	Contracture of muscle
M6249	Contracture of muscle
M625	Muscle wasting and atrophy, not elsewhere classified
M6250	Muscle wasting and atrophy, not elsewhere classified
M6251	Muscle wasting and atrophy, not elsewhere classified
M6252	Muscle wasting and atrophy, not elsewhere classified
M6253	Muscle wasting and atrophy, not elsewhere classified
M6254	Muscle wasting and atrophy, not elsewhere classified
M6255	Muscle wasting and atrophy, not elsewhere classified
M6256	Muscle wasting and atrophy, not elsewhere classified
M6257	Muscle wasting and atrophy, not elsewhere classified
M6258	Muscle wasting and atrophy, not elsewhere classified
M6259	Muscle wasting and atrophy, not elsewhere classified
M626	Muscle strain
M6260	Muscle strain
M6261	Muscle strain
M6262	Muscle strain
M6263	Muscle strain
M6264	Muscle strain
M6265	Muscle strain
M6266	Muscle strain
M6267	Muscle strain
M6268	Muscle strain
M6269	Muscle strain
M628	Other specified disorders of muscle
M6280	Other specified disorders of muscle
M6281	Other specified disorders of muscle
M6282	Other specified disorders of muscle
M6283	Other specified disorders of muscle
M6284	Other specified disorders of muscle

For peer review only

M6285	Other specified disorders of muscle
M6286	Other specified disorders of muscle
M6287	Other specified disorders of muscle
M6288	Other specified disorders of muscle
M6289	Other specified disorders of muscle
M629	Disorder of muscle, unspecified
M6290	Disorder of muscle, unspecified
M6291	Disorder of muscle, unspecified
M6292	Disorder of muscle, unspecified
M6293	Disorder of muscle, unspecified
M6294	Disorder of muscle, unspecified
M6295	Disorder of muscle, unspecified
M6296	Disorder of muscle, unspecified
M6297	Disorder of muscle, unspecified
M6298	Disorder of muscle, unspecified
M6299	Disorder of muscle, unspecified
M63	Disorders of muscle in diseases classified elsewhere
M630	Myositis in bacterial diseases classified elsewhere
M631	Myositis in protozoal and parasitic infections classified elsewhere
M632	Myositis in other infectious diseases classified elsewhere
M633	Myositis in sarcoidosis
M638	Other disorders of muscle in diseases classified elsewhere
M65	Synovitis and tenosynovitis
M650	Abscess of tendon sheath
M6500	Abscess of tendon sheath
M6501	Abscess of tendon sheath
M6502	Abscess of tendon sheath
M6503	Abscess of tendon sheath
M6504	Abscess of tendon sheath
M6505	Abscess of tendon sheath
M6506	Abscess of tendon sheath
M6507	Abscess of tendon sheath
M6508	Abscess of tendon sheath
M6509	Abscess of tendon sheath
M651	Other infective (teno)synovitis
M6510	Other infective (teno)synovitis
M6511	Other infective (teno)synovitis
M6512	Other infective (teno)synovitis
M6513	Other infective (teno)synovitis
M6514	Other infective (teno)synovitis
M6515	Other infective (teno)synovitis
M6516	Other infective (teno)synovitis
M6517	Other infective (teno)synovitis
M6518	Other infective (teno)synovitis
M6519	Other infective (teno)synovitis
M652	Calcific tendinitis
M6520	Calcific tendinitis
M6521	Calcific tendinitis

For peer review only

M6522	Calcific tendinitis
M6523	Calcific tendinitis
M6524	Calcific tendinitis
M6525	Calcific tendinitis
M6526	Calcific tendinitis
M6527	Calcific tendinitis
M6528	Calcific tendinitis
M6529	Calcific tendinitis
M653	Trigger finger
M654	Radial styloid tenosynovitis [de Quervain]
M6540	Radial styloid tenosynovitis [de Quervain]
M6543	Radial styloid tenosynovitis [de Quervain]
M6544	Radial styloid tenosynovitis [de Quervain]
M658	Other synovitis and tenosynovitis
M6580	Other synovitis and tenosynovitis
M6581	Other synovitis and tenosynovitis
M6582	Other synovitis and tenosynovitis
M6583	Other synovitis and tenosynovitis
M6584	Other synovitis and tenosynovitis
M6585	Other synovitis and tenosynovitis
M6586	Other synovitis and tenosynovitis
M6587	Other synovitis and tenosynovitis
M6588	Other synovitis and tenosynovitis
M6589	Other synovitis and tenosynovitis
M659	Synovitis and tenosynovitis, unspecified
M6590	Synovitis and tenosynovitis, unspecified
M6591	Synovitis and tenosynovitis, unspecified
M6592	Synovitis and tenosynovitis, unspecified
M6593	Synovitis and tenosynovitis, unspecified
M6594	Synovitis and tenosynovitis, unspecified
M6595	Synovitis and tenosynovitis, unspecified
M6596	Synovitis and tenosynovitis, unspecified
M6597	Synovitis and tenosynovitis, unspecified
M6598	Synovitis and tenosynovitis, unspecified
M6599	Synovitis and tenosynovitis, unspecified
M66	Spontaneous rupture of synovium and tendon
M660	Rupture of popliteal cyst
M661	Rupture of synovium
M6610	Rupture of synovium
M6611	Rupture of synovium
M6612	Rupture of synovium
M6613	Rupture of synovium
M6614	Rupture of synovium
M6615	Rupture of synovium
M6616	Rupture of synovium
M6617	Rupture of synovium
M6618	Rupture of synovium
M6619	Rupture of synovium

For peer review only

M662	Spontaneous rupture of extensor tendons
M6620	Spontaneous rupture of extensor tendons
M6621	Spontaneous rupture of extensor tendons
M6622	Spontaneous rupture of extensor tendons
M6623	Spontaneous rupture of extensor tendons
M6624	Spontaneous rupture of extensor tendons
M6625	Spontaneous rupture of extensor tendons
M6626	Spontaneous rupture of extensor tendons
M6627	Spontaneous rupture of extensor tendons
M6629	Spontaneous rupture of extensor tendons
M663	Spontaneous rupture of flexor tendons
M6630	Spontaneous rupture of flexor tendons
M6631	Spontaneous rupture of flexor tendons
M6632	Spontaneous rupture of flexor tendons
M6633	Spontaneous rupture of flexor tendons
M6634	Spontaneous rupture of flexor tendons
M6635	Spontaneous rupture of flexor tendons
M6636	Spontaneous rupture of flexor tendons
M6637	Spontaneous rupture of flexor tendons
M6638	Spontaneous rupture of flexor tendons
M6639	Spontaneous rupture of flexor tendons
M664	Spontaneous rupture of other tendons
M6640	Spontaneous rupture of other tendons
M6641	Spontaneous rupture of other tendons
M6642	Spontaneous rupture of other tendons
M6643	Spontaneous rupture of other tendons
M6644	Spontaneous rupture of other tendons
M6645	Spontaneous rupture of other tendons
M6646	Spontaneous rupture of other tendons
M6647	Spontaneous rupture of other tendons
M6648	Spontaneous rupture of other tendons
M6649	Spontaneous rupture of other tendons
M665	Spontaneous rupture of unspecified tendon
M6650	Spontaneous rupture of unspecified tendon
M6651	Spontaneous rupture of unspecified tendon
M6652	Spontaneous rupture of unspecified tendon
M6653	Spontaneous rupture of unspecified tendon
M6654	Spontaneous rupture of unspecified tendon
M6655	Spontaneous rupture of unspecified tendon
M6656	Spontaneous rupture of unspecified tendon
M6657	Spontaneous rupture of unspecified tendon
M6658	Spontaneous rupture of unspecified tendon
M6659	Spontaneous rupture of unspecified tendon
M67	Other disorders of synovium and tendon
M670	Short Achilles tendon (acquired)
M671	Other contracture of tendon (sheath)
M672	Synovial hypertrophy, not elsewhere classified
M673	Transient synovitis

For peer review only

M674	Ganglion
M678	Other specified disorders of synovium and tendon
M679	Disorder of synovium and tendon, unspecified
M68	Disorders of synovium and tendon in diseases classified elsewhere
M680	Synovitis and tenosynovitis in bacterial diseases classified elsewhere
M688	Other disorders of synovium and tendon in diseases classified elsewhere
M70	Soft tissue disorders related to use, overuse and pressure
M700	Chronic crepitant synovitis of hand and wrist
M7000	Chronic crepitant synovitis of hand and wrist
M7003	Chronic crepitant synovitis of hand and wrist
M7004	Chronic crepitant synovitis of hand and wrist
M701	Bursitis of hand
M702	Olecranon bursitis
M703	Other bursitis of elbow
M704	Prepatellar bursitis
M705	Other bursitis of knee
M706	Trochanteric bursitis
M707	Other bursitis of hip
M708	Other soft tissue disorders related to use, overuse and pressure
M7080	Other soft tissue disorders related to use, overuse and pressure
M7081	Other soft tissue disorders related to use, overuse and pressure
M7082	Other soft tissue disorders related to use, overuse and pressure
M7083	Other soft tissue disorders related to use, overuse and pressure
M7084	Other soft tissue disorders related to use, overuse and pressure
M7085	Other soft tissue disorders related to use, overuse and pressure
M7086	Other soft tissue disorders related to use, overuse and pressure
M7087	Other soft tissue disorders related to use, overuse and pressure
M7088	Other soft tissue disorders related to use, overuse and pressure
M7089	Other soft tissue disorders related to use, overuse and pressure
M709	Unspecified soft tissue disorder related to use, overuse and pressure
M7090	Unspecified soft tissue disorder related to use, overuse and pressure
M7091	Unspecified soft tissue disorder related to use, overuse and pressure
M7092	Unspecified soft tissue disorder related to use, overuse and pressure
M7093	Unspecified soft tissue disorder related to use, overuse and pressure
M7094	Unspecified soft tissue disorder related to use, overuse and pressure
M7095	Unspecified soft tissue disorder related to use, overuse and pressure
M7096	Unspecified soft tissue disorder related to use, overuse and pressure
M7097	Unspecified soft tissue disorder related to use, overuse and pressure
M7098	Unspecified soft tissue disorder related to use, overuse and pressure
M7099	Unspecified soft tissue disorder related to use, overuse and pressure
M71	Other bursopathies
M710	Abscess of bursa
M7100	Abscess of bursa
M7101	Abscess of bursa
M7102	Abscess of bursa
M7103	Abscess of bursa
M7104	Abscess of bursa
M7105	Abscess of bursa

For peer review only

M7106	Abscess of bursa
M7107	Abscess of bursa
M7108	Abscess of bursa
M7109	Abscess of bursa
M711	Other infective bursitis
M7110	Other infective bursitis
M7111	Other infective bursitis
M7112	Other infective bursitis
M7113	Other infective bursitis
M7114	Other infective bursitis
M7115	Other infective bursitis
M7116	Other infective bursitis
M7117	Other infective bursitis
M7118	Other infective bursitis
M7119	Other infective bursitis
M712	Synovial cyst of popliteal space [Baker]
M713	Other bursal cyst
M7130	Other bursal cyst
M7131	Other bursal cyst
M7132	Other bursal cyst
M7133	Other bursal cyst
M7134	Other bursal cyst
M7135	Other bursal cyst
M7136	Other bursal cyst
M7137	Other bursal cyst
M7138	Other bursal cyst
M7139	Other bursal cyst
M714	Calcium deposit in bursa
M7140	Calcium deposit in bursa
M7141	Calcium deposit in bursa
M7142	Calcium deposit in bursa
M7143	Calcium deposit in bursa
M7144	Calcium deposit in bursa
M7145	Calcium deposit in bursa
M7146	Calcium deposit in bursa
M7147	Calcium deposit in bursa
M7148	Calcium deposit in bursa
M7149	Calcium deposit in bursa
M715	Other bursitis, not elsewhere classified
M7150	Other bursitis, not elsewhere classified
M7151	Other bursitis, not elsewhere classified
M7152	Other bursitis, not elsewhere classified
M7153	Other bursitis, not elsewhere classified
M7154	Other bursitis, not elsewhere classified
M7155	Other bursitis, not elsewhere classified
M7156	Other bursitis, not elsewhere classified
M7157	Other bursitis, not elsewhere classified
M7158	Other bursitis, not elsewhere classified

For peer review only

M7159	Other bursitis, not elsewhere classified
M718	Other specified bursopathies
M7180	Other specified bursopathies
M7181	Other specified bursopathies
M7182	Other specified bursopathies
M7183	Other specified bursopathies
M7184	Other specified bursopathies
M7185	Other specified bursopathies
M7186	Other specified bursopathies
M7187	Other specified bursopathies
M7188	Other specified bursopathies
M7189	Other specified bursopathies
M719	Bursopathy, unspecified
M7190	Bursopathy, unspecified
M7191	Bursopathy, unspecified
M7192	Bursopathy, unspecified
M7193	Bursopathy, unspecified
M7194	Bursopathy, unspecified
M7195	Bursopathy, unspecified
M7196	Bursopathy, unspecified
M7197	Bursopathy, unspecified
M7198	Bursopathy, unspecified
M7199	Bursopathy, unspecified
M72	Fibroblastic disorders
M720	Palmar fascial fibromatosis [Dupuytren]
M721	Knuckle pads
M7210	Knuckle pads
M7214	Knuckle pads
M7217	Knuckle pads
M7219	Knuckle pads
M722	Plantar fascial fibromatosis
M723	Nodular fasciitis
M7230	Nodular fasciitis
M7231	Nodular fasciitis
M7232	Nodular fasciitis
M7233	Nodular fasciitis
M7234	Nodular fasciitis
M7235	Nodular fasciitis
M7236	Nodular fasciitis
M7237	Nodular fasciitis
M7238	Nodular fasciitis
M724	Pseudosarcomatous fibromatosis
M7240	Pseudosarcomatous fibromatosis
M7241	Pseudosarcomatous fibromatosis
M7242	Pseudosarcomatous fibromatosis
M7243	Pseudosarcomatous fibromatosis
M7244	Pseudosarcomatous fibromatosis
M7245	Pseudosarcomatous fibromatosis

For peer review only

M7246	Pseudosarcomatous fibromatosis
M7247	Pseudosarcomatous fibromatosis
M7248	Pseudosarcomatous fibromatosis
M7249	Pseudosarcomatous fibromatosis
M725	Fasciitis, not elsewhere classified
M7250	Fasciitis, not elsewhere classified
M7251	Fasciitis, not elsewhere classified
M7252	Fasciitis, not elsewhere classified
M7253	Fasciitis, not elsewhere classified
M7254	Fasciitis, not elsewhere classified
M7255	Fasciitis, not elsewhere classified
M7256	Fasciitis, not elsewhere classified
M7257	Fasciitis, not elsewhere classified
M7258	Fasciitis, not elsewhere classified
M7259	Fasciitis, not elsewhere classified
M726	Necrotizing fasciitis
M7260	Necrotizing fasciitis
M7261	Necrotizing fasciitis
M7262	Necrotizing fasciitis
M7263	Necrotizing fasciitis
M7264	Necrotizing fasciitis
M7265	Necrotizing fasciitis
M7266	Necrotizing fasciitis
M7267	Necrotizing fasciitis
M7268	Necrotizing fasciitis
M7269	Necrotizing fasciitis
M728	Other fibroblastic disorders
M7280	Other fibroblastic disorders
M7281	Other fibroblastic disorders
M7282	Other fibroblastic disorders
M7283	Other fibroblastic disorders
M7284	Other fibroblastic disorders
M7285	Other fibroblastic disorders
M7286	Other fibroblastic disorders
M7287	Other fibroblastic disorders
M7288	Other fibroblastic disorders
M7289	Other fibroblastic disorders
M729	Fibroblastic disorder, unspecified
M7290	Fibroblastic disorder, unspecified
M7291	Fibroblastic disorder, unspecified
M7292	Fibroblastic disorder, unspecified
M7293	Fibroblastic disorder, unspecified
M7294	Fibroblastic disorder, unspecified
M7295	Fibroblastic disorder, unspecified
M7296	Fibroblastic disorder, unspecified
M7297	Fibroblastic disorder, unspecified
M7298	Fibroblastic disorder, unspecified
M7299	Fibroblastic disorder, unspecified

For peer review only

M73	Soft tissue disorders in diseases classified elsewhere
M730	Gonococcal bursitis
M7300	Gonococcal bursitis
M7301	Gonococcal bursitis
M7302	Gonococcal bursitis
M7303	Gonococcal bursitis
M7304	Gonococcal bursitis
M7305	Gonococcal bursitis
M7306	Gonococcal bursitis
M7307	Gonococcal bursitis
M7308	Gonococcal bursitis
M7309	Gonococcal bursitis
M731	Syphilitic bursitis
M7310	Syphilitic bursitis
M7311	Syphilitic bursitis
M7312	Syphilitic bursitis
M7313	Syphilitic bursitis
M7314	Syphilitic bursitis
M7315	Syphilitic bursitis
M7316	Syphilitic bursitis
M7317	Syphilitic bursitis
M7318	Syphilitic bursitis
M7319	Syphilitic bursitis
M738	Other soft tissue disorders in diseases classified elsewhere
M7380	Other soft tissue disorders in diseases classified elsewhere
M7381	Other soft tissue disorders in diseases classified elsewhere
M7382	Other soft tissue disorders in diseases classified elsewhere
M7383	Other soft tissue disorders in diseases classified elsewhere
M7384	Other soft tissue disorders in diseases classified elsewhere
M7385	Other soft tissue disorders in diseases classified elsewhere
M7386	Other soft tissue disorders in diseases classified elsewhere
M7387	Other soft tissue disorders in diseases classified elsewhere
M7388	Other soft tissue disorders in diseases classified elsewhere
M7389	Other soft tissue disorders in diseases classified elsewhere
M75	Shoulder lesions
M750	Adhesive capsulitis of shoulder
M751	Rotator cuff syndrome
M752	Bicipital tendinitis
M753	Calcific tendinitis of shoulder
M754	Impingement syndrome of shoulder
M755	Bursitis of shoulder
M758	Other shoulder lesions
M759	Shoulder lesion, unspecified
M76	Enthesopathies of lower limb, excluding foot
M760	Gluteal tendinitis
M7600	Gluteal tendinitis
M7605	Gluteal tendinitis
M761	Psoas tendinitis

For peer review only

M7610	Psoas tendinitis
M7615	Psoas tendinitis
M762	Iliac crest spur
M763	Iliotibial band syndrome
M7630	Iliotibial band syndrome
M7635	Iliotibial band syndrome
M7636	Iliotibial band syndrome
M764	Tibial collateral bursitis [Pellegrini-Stieda]
M765	Patellar tendinitis
M766	Achilles tendinitis
M7660	Achilles tendinitis
M7666	Achilles tendinitis
M7667	Achilles tendinitis
M767	Peroneal tendinitis
M7670	Peroneal tendinitis
M7676	Peroneal tendinitis
M7677	Peroneal tendinitis
M768	Other enthesopathies of lower limb, excluding foot
M7680	Other enthesopathies of lower limb, excluding foot
M7685	Other enthesopathies of lower limb, excluding foot
M7686	Other enthesopathies of lower limb, excluding foot
M7687	Other enthesopathies of lower limb, excluding foot
M769	Enthesopathy of lower limb, unspecified
M7690	Enthesopathy of lower limb, unspecified
M7695	Enthesopathy of lower limb, unspecified
M7696	Enthesopathy of lower limb, unspecified
M7697	Enthesopathy of lower limb, unspecified
M7698	Enthesopathy of lower limb, unspecified
M7699	Enthesopathy of lower limb, unspecified
M77	Other enthesopathies
M770	Medial epicondylitis
M7700	Medial epicondylitis
M7702	Medial epicondylitis
M7705	Medial epicondylitis
M7706	Medial epicondylitis
M7709	Medial epicondylitis
M771	Lateral epicondylitis
M7710	Lateral epicondylitis
M7712	Lateral epicondylitis
M7715	Lateral epicondylitis
M7716	Lateral epicondylitis
M7719	Lateral epicondylitis
M772	Periarthritis of wrist
M773	Calcaneal spur
M774	Metatarsalgia
M775	Other enthesopathy of foot
M778	Other enthesopathies, not elsewhere classified
M7780	Other enthesopathies, not elsewhere classified

For peer review only

M7781	Other enthesopathies, not elsewhere classified
M7782	Other enthesopathies, not elsewhere classified
M7783	Other enthesopathies, not elsewhere classified
M7784	Other enthesopathies, not elsewhere classified
M7785	Other enthesopathies, not elsewhere classified
M7786	Other enthesopathies, not elsewhere classified
M7787	Other enthesopathies, not elsewhere classified
M7788	Other enthesopathies, not elsewhere classified
M7789	Other enthesopathies, not elsewhere classified
M779	Enthesopathy, unspecified
M7790	Enthesopathy, unspecified
M7791	Enthesopathy, unspecified
M7792	Enthesopathy, unspecified
M7793	Enthesopathy, unspecified
M7794	Enthesopathy, unspecified
M7795	Enthesopathy, unspecified
M7796	Enthesopathy, unspecified
M7797	Enthesopathy, unspecified
M7798	Enthesopathy, unspecified
M7799	Enthesopathy, unspecified
M79	Other soft tissue disorders, not elsewhere classified
M790	Rheumatism, unspecified
M7900	Rheumatism, unspecified
M7901	Rheumatism, unspecified
M7902	Rheumatism, unspecified
M7903	Rheumatism, unspecified
M7904	Rheumatism, unspecified
M7905	Rheumatism, unspecified
M7906	Rheumatism, unspecified
M7907	Rheumatism, unspecified
M7908	Rheumatism, unspecified
M7909	Rheumatism, unspecified
M791	Myalgia
M7910	Myalgia
M7911	Myalgia
M7912	Myalgia
M7913	Myalgia
M7914	Myalgia
M7915	Myalgia
M7916	Myalgia
M7917	Myalgia
M7918	Myalgia
M7919	Myalgia
M792	Neuralgia and neuritis, unspecified
M7920	Neuralgia and neuritis, unspecified
M7921	Neuralgia and neuritis, unspecified
M7922	Neuralgia and neuritis, unspecified
M7923	Neuralgia and neuritis, unspecified

For peer review only

M7924	Neuralgia and neuritis, unspecified
M7925	Neuralgia and neuritis, unspecified
M7926	Neuralgia and neuritis, unspecified
M7927	Neuralgia and neuritis, unspecified
M7928	Neuralgia and neuritis, unspecified
M7929	Neuralgia and neuritis, unspecified
M793	Panniculitis, unspecified
M7930	Panniculitis, unspecified
M7931	Panniculitis, unspecified
M7932	Panniculitis, unspecified
M7933	Panniculitis, unspecified
M7934	Panniculitis, unspecified
M7935	Panniculitis, unspecified
M7936	Panniculitis, unspecified
M7937	Panniculitis, unspecified
M7938	Panniculitis, unspecified
M7939	Panniculitis, unspecified
M794	Hypertrophy of (infrapatellar) fat pad
M7940	Hypertrophy of (infrapatellar) fat pad
M7941	Hypertrophy of (infrapatellar) fat pad
M7942	Hypertrophy of (infrapatellar) fat pad
M7943	Hypertrophy of (infrapatellar) fat pad
M7944	Hypertrophy of (infrapatellar) fat pad
M7945	Hypertrophy of (infrapatellar) fat pad
M7946	Hypertrophy of (infrapatellar) fat pad
M7947	Hypertrophy of (infrapatellar) fat pad
M7948	Hypertrophy of (infrapatellar) fat pad
M795	Residual foreign body in soft tissue
M7950	Residual foreign body in soft tissue
M7951	Residual foreign body in soft tissue
M7952	Residual foreign body in soft tissue
M7953	Residual foreign body in soft tissue
M7954	Residual foreign body in soft tissue
M7955	Residual foreign body in soft tissue
M7956	Residual foreign body in soft tissue
M7957	Residual foreign body in soft tissue
M7958	Residual foreign body in soft tissue
M7959	Residual foreign body in soft tissue
M796	Pain in limb
M7960	Pain in limb
M7961	Pain in limb
M7962	Pain in limb
M7963	Pain in limb
M7964	Pain in limb
M7965	Pain in limb
M7966	Pain in limb
M7967	Pain in limb
M7968	Pain in limb

For peer review only

M7969	Pain in limb
M797	Fibromyalgia
M7970	Fibromyalgia
M7971	Fibromyalgia
M7972	Fibromyalgia
M7973	Fibromyalgia
M7974	Fibromyalgia
M7975	Fibromyalgia
M7976	Fibromyalgia
M7977	Fibromyalgia
M7978	Fibromyalgia
M7979	Fibromyalgia
M798	Other specified soft tissue disorders
M7980	Other specified soft tissue disorders
M7981	Other specified soft tissue disorders
M7982	Other specified soft tissue disorders
M7983	Other specified soft tissue disorders
M7984	Other specified soft tissue disorders
M7985	Other specified soft tissue disorders
M7986	Other specified soft tissue disorders
M7987	Other specified soft tissue disorders
M7988	Other specified soft tissue disorders
M7989	Other specified soft tissue disorders
M799	Soft tissue disorder, unspecified
M7990	Soft tissue disorder, unspecified
M7991	Soft tissue disorder, unspecified
M7992	Soft tissue disorder, unspecified
M7993	Soft tissue disorder, unspecified
M7994	Soft tissue disorder, unspecified
M7995	Soft tissue disorder, unspecified
M7996	Soft tissue disorder, unspecified
M7997	Soft tissue disorder, unspecified
M7998	Soft tissue disorder, unspecified
M7999	Soft tissue disorder, unspecified
M80	Osteoporosis with pathological fracture
M800	Postmenopausal osteoporosis with pathological fracture
M8000	Postmenopausal osteoporosis with pathological fracture
M8001	Postmenopausal osteoporosis with pathological fracture
M8002	Postmenopausal osteoporosis with pathological fracture
M8003	Postmenopausal osteoporosis with pathological fracture
M8004	Postmenopausal osteoporosis with pathological fracture
M8005	Postmenopausal osteoporosis with pathological fracture
M8006	Postmenopausal osteoporosis with pathological fracture
M8007	Postmenopausal osteoporosis with pathological fracture
M8008	Postmenopausal osteoporosis with pathological fracture
M8009	Postmenopausal osteoporosis with pathological fracture
M801	Postoophorectomy osteoporosis with pathological fracture
M8010	Postoophorectomy osteoporosis with pathological fracture

For peer review only

M8011	Postoophorectomy osteoporosis with pathological fracture
M8012	Postoophorectomy osteoporosis with pathological fracture
M8013	Postoophorectomy osteoporosis with pathological fracture
M8014	Postoophorectomy osteoporosis with pathological fracture
M8015	Postoophorectomy osteoporosis with pathological fracture
M8016	Postoophorectomy osteoporosis with pathological fracture
M8017	Postoophorectomy osteoporosis with pathological fracture
M8018	Postoophorectomy osteoporosis with pathological fracture
M8019	Postoophorectomy osteoporosis with pathological fracture
M802	Osteoporosis of disuse with pathological fracture
M8020	Osteoporosis of disuse with pathological fracture
M8021	Osteoporosis of disuse with pathological fracture
M8022	Osteoporosis of disuse with pathological fracture
M8023	Osteoporosis of disuse with pathological fracture
M8024	Osteoporosis of disuse with pathological fracture
M8025	Osteoporosis of disuse with pathological fracture
M8026	Osteoporosis of disuse with pathological fracture
M8027	Osteoporosis of disuse with pathological fracture
M8028	Osteoporosis of disuse with pathological fracture
M8029	Osteoporosis of disuse with pathological fracture
M803	Postsurgical malabsorption osteoporosis with pathological fracture
M8030	Postsurgical malabsorption osteoporosis with pathological fracture
M8031	Postsurgical malabsorption osteoporosis with pathological fracture
M8032	Postsurgical malabsorption osteoporosis with pathological fracture
M8033	Postsurgical malabsorption osteoporosis with pathological fracture
M8034	Postsurgical malabsorption osteoporosis with pathological fracture
M8035	Postsurgical malabsorption osteoporosis with pathological fracture
M8036	Postsurgical malabsorption osteoporosis with pathological fracture
M8037	Postsurgical malabsorption osteoporosis with pathological fracture
M8038	Postsurgical malabsorption osteoporosis with pathological fracture
M8039	Postsurgical malabsorption osteoporosis with pathological fracture
M804	Drug-induced osteoporosis with pathological fracture
M8040	Drug-induced osteoporosis with pathological fracture
M8041	Drug-induced osteoporosis with pathological fracture
M8042	Drug-induced osteoporosis with pathological fracture
M8043	Drug-induced osteoporosis with pathological fracture
M8044	Drug-induced osteoporosis with pathological fracture
M8045	Drug-induced osteoporosis with pathological fracture
M8046	Drug-induced osteoporosis with pathological fracture
M8047	Drug-induced osteoporosis with pathological fracture
M8048	Drug-induced osteoporosis with pathological fracture
M8049	Drug-induced osteoporosis with pathological fracture
M805	Idiopathic osteoporosis with pathological fracture
M8050	Idiopathic osteoporosis with pathological fracture
M8051	Idiopathic osteoporosis with pathological fracture
M8052	Idiopathic osteoporosis with pathological fracture
M8053	Idiopathic osteoporosis with pathological fracture
M8054	Idiopathic osteoporosis with pathological fracture

For peer review only

M8055	Idiopathic osteoporosis with pathological fracture
M8056	Idiopathic osteoporosis with pathological fracture
M8057	Idiopathic osteoporosis with pathological fracture
M8058	Idiopathic osteoporosis with pathological fracture
M8059	Idiopathic osteoporosis with pathological fracture
M808	Other osteoporosis with pathological fracture
M8080	Other osteoporosis with pathological fracture
M8081	Other osteoporosis with pathological fracture
M8082	Other osteoporosis with pathological fracture
M8083	Other osteoporosis with pathological fracture
M8084	Other osteoporosis with pathological fracture
M8085	Other osteoporosis with pathological fracture
M8086	Other osteoporosis with pathological fracture
M8087	Other osteoporosis with pathological fracture
M8088	Other osteoporosis with pathological fracture
M8089	Other osteoporosis with pathological fracture
M809	Unspecified osteoporosis with pathological fracture
M8090	Unspecified osteoporosis with pathological fracture
M8091	Unspecified osteoporosis with pathological fracture
M8092	Unspecified osteoporosis with pathological fracture
M8093	Unspecified osteoporosis with pathological fracture
M8094	Unspecified osteoporosis with pathological fracture
M8095	Unspecified osteoporosis with pathological fracture
M8096	Unspecified osteoporosis with pathological fracture
M8097	Unspecified osteoporosis with pathological fracture
M8098	Unspecified osteoporosis with pathological fracture
M8099	Unspecified osteoporosis with pathological fracture
M81	Osteoporosis without pathological fracture
M810	Postmenopausal osteoporosis
M8100	Postmenopausal osteoporosis
M8101	Postmenopausal osteoporosis
M8102	Postmenopausal osteoporosis
M8103	Postmenopausal osteoporosis
M8104	Postmenopausal osteoporosis
M8105	Postmenopausal osteoporosis
M8106	Postmenopausal osteoporosis
M8107	Postmenopausal osteoporosis
M8108	Postmenopausal osteoporosis
M8109	Postmenopausal osteoporosis
M811	Postoophorectomy osteoporosis
M8110	Postoophorectomy osteoporosis
M8111	Postoophorectomy osteoporosis
M8112	Postoophorectomy osteoporosis
M8113	Postoophorectomy osteoporosis
M8114	Postoophorectomy osteoporosis
M8115	Postoophorectomy osteoporosis
M8116	Postoophorectomy osteoporosis
M8117	Postoophorectomy osteoporosis

For peer review only

M8118	Postoophorectomy osteoporosis
M8119	Postoophorectomy osteoporosis
M812	Osteoporosis of disuse
M8120	Osteoporosis of disuse
M8121	Osteoporosis of disuse
M8122	Osteoporosis of disuse
M8123	Osteoporosis of disuse
M8124	Osteoporosis of disuse
M8125	Osteoporosis of disuse
M8126	Osteoporosis of disuse
M8127	Osteoporosis of disuse
M8128	Osteoporosis of disuse
M8129	Osteoporosis of disuse
M813	Postsurgical malabsorption osteoporosis
M8130	Postsurgical malabsorption osteoporosis
M8131	Postsurgical malabsorption osteoporosis
M8132	Postsurgical malabsorption osteoporosis
M8133	Postsurgical malabsorption osteoporosis
M8134	Postsurgical malabsorption osteoporosis
M8135	Postsurgical malabsorption osteoporosis
M8136	Postsurgical malabsorption osteoporosis
M8137	Postsurgical malabsorption osteoporosis
M8138	Postsurgical malabsorption osteoporosis
M8139	Postsurgical malabsorption osteoporosis
M814	Drug-induced osteoporosis
M8140	Drug-induced osteoporosis
M8141	Drug-induced osteoporosis
M8142	Drug-induced osteoporosis
M8143	Drug-induced osteoporosis
M8144	Drug-induced osteoporosis
M8145	Drug-induced osteoporosis
M8146	Drug-induced osteoporosis
M8147	Drug-induced osteoporosis
M8148	Drug-induced osteoporosis
M8149	Drug-induced osteoporosis
M815	Idiopathic osteoporosis
M8150	Idiopathic osteoporosis
M8151	Idiopathic osteoporosis
M8152	Idiopathic osteoporosis
M8153	Idiopathic osteoporosis
M8154	Idiopathic osteoporosis
M8155	Idiopathic osteoporosis
M8156	Idiopathic osteoporosis
M8157	Idiopathic osteoporosis
M8158	Idiopathic osteoporosis
M8159	Idiopathic osteoporosis
M816	Localized osteoporosis [Lequesne]
M8160	Localized osteoporosis [Lequesne]

For peer review only

M8161	Localized osteoporosis [Lequesne]
M8162	Localized osteoporosis [Lequesne]
M8163	Localized osteoporosis [Lequesne]
M8164	Localized osteoporosis [Lequesne]
M8165	Localized osteoporosis [Lequesne]
M8166	Localized osteoporosis [Lequesne]
M8167	Localized osteoporosis [Lequesne]
M8168	Localized osteoporosis [Lequesne]
M8169	Localized osteoporosis [Lequesne]
M818	Other osteoporosis
M8180	Other osteoporosis
M8181	Other osteoporosis
M8182	Other osteoporosis
M8183	Other osteoporosis
M8184	Other osteoporosis
M8185	Other osteoporosis
M8186	Other osteoporosis
M8187	Other osteoporosis
M8188	Other osteoporosis
M8189	Other osteoporosis
M819	Osteoporosis, unspecified
M8190	Osteoporosis, unspecified
M8191	Osteoporosis, unspecified
M8192	Osteoporosis, unspecified
M8193	Osteoporosis, unspecified
M8194	Osteoporosis, unspecified
M8195	Osteoporosis, unspecified
M8196	Osteoporosis, unspecified
M8197	Osteoporosis, unspecified
M8198	Osteoporosis, unspecified
M8199	Osteoporosis, unspecified
M82	Osteoporosis in diseases classified elsewhere
M820	Osteoporosis in multiple myelomatosis
M8200	Osteoporosis in multiple myelomatosis
M8201	Osteoporosis in multiple myelomatosis
M8202	Osteoporosis in multiple myelomatosis
M8203	Osteoporosis in multiple myelomatosis
M8204	Osteoporosis in multiple myelomatosis
M8205	Osteoporosis in multiple myelomatosis
M8206	Osteoporosis in multiple myelomatosis
M8207	Osteoporosis in multiple myelomatosis
M8208	Osteoporosis in multiple myelomatosis
M8209	Osteoporosis in multiple myelomatosis
M821	Osteoporosis in endocrine disorders
M8210	Osteoporosis in endocrine disorders
M8211	Osteoporosis in endocrine disorders
M8212	Osteoporosis in endocrine disorders
M8213	Osteoporosis in endocrine disorders

For peer review only

M8214	Osteoporosis in endocrine disorders
M8215	Osteoporosis in endocrine disorders
M8216	Osteoporosis in endocrine disorders
M8217	Osteoporosis in endocrine disorders
M8218	Osteoporosis in endocrine disorders
M8219	Osteoporosis in endocrine disorders
M828	Osteoporosis in other diseases classified elsewhere
M8280	Osteoporosis in other diseases classified elsewhere
M8281	Osteoporosis in other diseases classified elsewhere
M8282	Osteoporosis in other diseases classified elsewhere
M8283	Osteoporosis in other diseases classified elsewhere
M8284	Osteoporosis in other diseases classified elsewhere
M8285	Osteoporosis in other diseases classified elsewhere
M8286	Osteoporosis in other diseases classified elsewhere
M8287	Osteoporosis in other diseases classified elsewhere
M8288	Osteoporosis in other diseases classified elsewhere
M8289	Osteoporosis in other diseases classified elsewhere
M83	Adult osteomalacia
M830	Puerperal osteomalacia
M8300	Puerperal osteomalacia
M8301	Puerperal osteomalacia
M8302	Puerperal osteomalacia
M8303	Puerperal osteomalacia
M8304	Puerperal osteomalacia
M8305	Puerperal osteomalacia
M8306	Puerperal osteomalacia
M8307	Puerperal osteomalacia
M8308	Puerperal osteomalacia
M8309	Puerperal osteomalacia
M831	Senile osteomalacia
M8310	Senile osteomalacia
M8311	Senile osteomalacia
M8312	Senile osteomalacia
M8313	Senile osteomalacia
M8314	Senile osteomalacia
M8315	Senile osteomalacia
M8316	Senile osteomalacia
M8317	Senile osteomalacia
M8318	Senile osteomalacia
M8319	Senile osteomalacia
M832	Adult osteomalacia due to malabsorption
M8320	Adult osteomalacia due to malabsorption
M8321	Adult osteomalacia due to malabsorption
M8322	Adult osteomalacia due to malabsorption
M8323	Adult osteomalacia due to malabsorption
M8324	Adult osteomalacia due to malabsorption
M8325	Adult osteomalacia due to malabsorption
M8326	Adult osteomalacia due to malabsorption

For peer review only

M8327	Adult osteomalacia due to malabsorption
M8328	Adult osteomalacia due to malabsorption
M8329	Adult osteomalacia due to malabsorption
M833	Adult osteomalacia due to malnutrition
M8330	Adult osteomalacia due to malnutrition
M8331	Adult osteomalacia due to malnutrition
M8332	Adult osteomalacia due to malnutrition
M8333	Adult osteomalacia due to malnutrition
M8334	Adult osteomalacia due to malnutrition
M8335	Adult osteomalacia due to malnutrition
M8336	Adult osteomalacia due to malnutrition
M8337	Adult osteomalacia due to malnutrition
M8338	Adult osteomalacia due to malnutrition
M8339	Adult osteomalacia due to malnutrition
M834	Aluminium bone disease
M8340	Aluminium bone disease
M8341	Aluminium bone disease
M8342	Aluminium bone disease
M8343	Aluminium bone disease
M8344	Aluminium bone disease
M8345	Aluminium bone disease
M8346	Aluminium bone disease
M8347	Aluminium bone disease
M8348	Aluminium bone disease
M8349	Aluminium bone disease
M835	Other drug-induced osteomalacia in adults
M8350	Other drug-induced osteomalacia in adults
M8351	Other drug-induced osteomalacia in adults
M8352	Other drug-induced osteomalacia in adults
M8353	Other drug-induced osteomalacia in adults
M8354	Other drug-induced osteomalacia in adults
M8355	Other drug-induced osteomalacia in adults
M8356	Other drug-induced osteomalacia in adults
M8357	Other drug-induced osteomalacia in adults
M8358	Other drug-induced osteomalacia in adults
M8359	Other drug-induced osteomalacia in adults
M838	Other adult osteomalacia
M8380	Other adult osteomalacia
M8381	Other adult osteomalacia
M8382	Other adult osteomalacia
M8383	Other adult osteomalacia
M8384	Other adult osteomalacia
M8385	Other adult osteomalacia
M8386	Other adult osteomalacia
M8387	Other adult osteomalacia
M8388	Other adult osteomalacia
M8389	Other adult osteomalacia
M839	Adult osteomalacia, unspecified

For peer review only

M8390	Adult osteomalacia, unspecified
M8391	Adult osteomalacia, unspecified
M8392	Adult osteomalacia, unspecified
M8393	Adult osteomalacia, unspecified
M8394	Adult osteomalacia, unspecified
M8395	Adult osteomalacia, unspecified
M8396	Adult osteomalacia, unspecified
M8397	Adult osteomalacia, unspecified
M8398	Adult osteomalacia, unspecified
M8399	Adult osteomalacia, unspecified
M84	Disorders of continuity of bone
M840	Malunion of fracture
M8400	Malunion of fracture
M8401	Malunion of fracture
M8402	Malunion of fracture
M8403	Malunion of fracture
M8404	Malunion of fracture
M8405	Malunion of fracture
M8406	Malunion of fracture
M8407	Malunion of fracture
M8408	Malunion of fracture
M8409	Malunion of fracture
M841	Nonunion of fracture [pseudarthrosis]
M8410	Nonunion of fracture [pseudarthrosis]
M8411	Nonunion of fracture [pseudarthrosis]
M8412	Nonunion of fracture [pseudarthrosis]
M8413	Nonunion of fracture [pseudarthrosis]
M8414	Nonunion of fracture [pseudarthrosis]
M8415	Nonunion of fracture [pseudarthrosis]
M8416	Nonunion of fracture [pseudarthrosis]
M8417	Nonunion of fracture [pseudarthrosis]
M8418	Nonunion of fracture [pseudarthrosis]
M8419	Nonunion of fracture [pseudarthrosis]
M842	Delayed union of fracture
M8420	Delayed union of fracture
M8421	Delayed union of fracture
M8422	Delayed union of fracture
M8423	Delayed union of fracture
M8424	Delayed union of fracture
M8425	Delayed union of fracture
M8426	Delayed union of fracture
M8427	Delayed union of fracture
M8428	Delayed union of fracture
M8429	Delayed union of fracture
M843	Stress fracture, not elsewhere classified
M8430	Stress fracture, not elsewhere classified
M8431	Stress fracture, not elsewhere classified
M8432	Stress fracture, not elsewhere classified

For peer review only

M8433	Stress fracture, not elsewhere classified
M8434	Stress fracture, not elsewhere classified
M8435	Stress fracture, not elsewhere classified
M8436	Stress fracture, not elsewhere classified
M8437	Stress fracture, not elsewhere classified
M8438	Stress fracture, not elsewhere classified
M8439	Stress fracture, not elsewhere classified
M844	Pathological fracture, not elsewhere classified
M8440	Pathological fracture, not elsewhere classified
M8441	Pathological fracture, not elsewhere classified
M8442	Pathological fracture, not elsewhere classified
M8443	Pathological fracture, not elsewhere classified
M8444	Pathological fracture, not elsewhere classified
M8445	Pathological fracture, not elsewhere classified
M8446	Pathological fracture, not elsewhere classified
M8447	Pathological fracture, not elsewhere classified
M8448	Pathological fracture, not elsewhere classified
M8449	Pathological fracture, not elsewhere classified
M848	Other disorders of continuity of bone
M8480	Other disorders of continuity of bone
M8481	Other disorders of continuity of bone
M8482	Other disorders of continuity of bone
M8483	Other disorders of continuity of bone
M8484	Other disorders of continuity of bone
M8485	Other disorders of continuity of bone
M8486	Other disorders of continuity of bone
M8487	Other disorders of continuity of bone
M8488	Other disorders of continuity of bone
M8489	Other disorders of continuity of bone
M849	Disorder of continuity of bone, unspecified
M8490	Disorder of continuity of bone, unspecified
M8491	Disorder of continuity of bone, unspecified
M8492	Disorder of continuity of bone, unspecified
M8493	Disorder of continuity of bone, unspecified
M8494	Disorder of continuity of bone, unspecified
M8495	Disorder of continuity of bone, unspecified
M8496	Disorder of continuity of bone, unspecified
M8497	Disorder of continuity of bone, unspecified
M8498	Disorder of continuity of bone, unspecified
M8499	Disorder of continuity of bone, unspecified
M85	Other disorders of bone density and structure
M850	Fibrous dysplasia (monostotic)
M8500	Fibrous dysplasia (monostotic)
M8501	Fibrous dysplasia (monostotic)
M8502	Fibrous dysplasia (monostotic)
M8503	Fibrous dysplasia (monostotic)
M8504	Fibrous dysplasia (monostotic)
M8505	Fibrous dysplasia (monostotic)

For peer review only

M8506	Fibrous dysplasia (monostotic)
M8507	Fibrous dysplasia (monostotic)
M8508	Fibrous dysplasia (monostotic)
M8509	Fibrous dysplasia (monostotic)
M851	Skeletal fluorosis
M8510	Skeletal fluorosis
M8511	Skeletal fluorosis
M8512	Skeletal fluorosis
M8513	Skeletal fluorosis
M8514	Skeletal fluorosis
M8515	Skeletal fluorosis
M8516	Skeletal fluorosis
M8517	Skeletal fluorosis
M8518	Skeletal fluorosis
M8519	Skeletal fluorosis
M852	Hyperostosis of skull
M853	Osteitis condensans
M8530	Osteitis condensans
M8531	Osteitis condensans
M8532	Osteitis condensans
M8533	Osteitis condensans
M8534	Osteitis condensans
M8535	Osteitis condensans
M8536	Osteitis condensans
M8537	Osteitis condensans
M8538	Osteitis condensans
M8539	Osteitis condensans
M854	Solitary bone cyst
M8540	Solitary bone cyst
M8541	Solitary bone cyst
M8542	Solitary bone cyst
M8543	Solitary bone cyst
M8544	Solitary bone cyst
M8545	Solitary bone cyst
M8546	Solitary bone cyst
M8547	Solitary bone cyst
M8548	Solitary bone cyst
M8549	Solitary bone cyst
M855	Aneurysmal bone cyst
M8550	Aneurysmal bone cyst
M8551	Aneurysmal bone cyst
M8552	Aneurysmal bone cyst
M8553	Aneurysmal bone cyst
M8554	Aneurysmal bone cyst
M8555	Aneurysmal bone cyst
M8556	Aneurysmal bone cyst
M8557	Aneurysmal bone cyst
M8558	Aneurysmal bone cyst

For peer review only

M8559	Aneurysmal bone cyst
M856	Other cyst of bone
M8560	Other cyst of bone
M8561	Other cyst of bone
M8562	Other cyst of bone
M8563	Other cyst of bone
M8564	Other cyst of bone
M8565	Other cyst of bone
M8566	Other cyst of bone
M8567	Other cyst of bone
M8568	Other cyst of bone
M8569	Other cyst of bone
M858	Other specified disorders of bone density and structure
M8580	Other specified disorders of bone density and structure
M8581	Other specified disorders of bone density and structure
M8582	Other specified disorders of bone density and structure
M8583	Other specified disorders of bone density and structure
M8584	Other specified disorders of bone density and structure
M8585	Other specified disorders of bone density and structure
M8586	Other specified disorders of bone density and structure
M8587	Other specified disorders of bone density and structure
M8588	Other specified disorders of bone density and structure
M8589	Other specified disorders of bone density and structure
M859	Disorder of bone density and structure, unspecified
M8590	Disorder of bone density and structure, unspecified
M8591	Disorder of bone density and structure, unspecified
M8592	Disorder of bone density and structure, unspecified
M8593	Disorder of bone density and structure, unspecified
M8594	Disorder of bone density and structure, unspecified
M8595	Disorder of bone density and structure, unspecified
M8596	Disorder of bone density and structure, unspecified
M8597	Disorder of bone density and structure, unspecified
M8598	Disorder of bone density and structure, unspecified
M8599	Disorder of bone density and structure, unspecified
M86	Osteomyelitis
M860	Acute haematogenous osteomyelitis
M8600	Acute haematogenous osteomyelitis
M8601	Acute haematogenous osteomyelitis
M8602	Acute haematogenous osteomyelitis
M8603	Acute haematogenous osteomyelitis
M8604	Acute haematogenous osteomyelitis
M8605	Acute haematogenous osteomyelitis
M8606	Acute haematogenous osteomyelitis
M8607	Acute haematogenous osteomyelitis
M8608	Acute haematogenous osteomyelitis
M8609	Acute haematogenous osteomyelitis
M861	Other acute osteomyelitis
M8610	Other acute osteomyelitis

For peer review only

M8611	Other acute osteomyelitis
M8612	Other acute osteomyelitis
M8613	Other acute osteomyelitis
M8614	Other acute osteomyelitis
M8615	Other acute osteomyelitis
M8616	Other acute osteomyelitis
M8617	Other acute osteomyelitis
M8618	Other acute osteomyelitis
M8619	Other acute osteomyelitis
M862	Subacute osteomyelitis
M8620	Subacute osteomyelitis
M8621	Subacute osteomyelitis
M8622	Subacute osteomyelitis
M8623	Subacute osteomyelitis
M8624	Subacute osteomyelitis
M8625	Subacute osteomyelitis
M8626	Subacute osteomyelitis
M8627	Subacute osteomyelitis
M8628	Subacute osteomyelitis
M8629	Subacute osteomyelitis
M863	Chronic multifocal osteomyelitis
M8630	Chronic multifocal osteomyelitis
M8631	Chronic multifocal osteomyelitis
M8632	Chronic multifocal osteomyelitis
M8633	Chronic multifocal osteomyelitis
M8634	Chronic multifocal osteomyelitis
M8635	Chronic multifocal osteomyelitis
M8636	Chronic multifocal osteomyelitis
M8637	Chronic multifocal osteomyelitis
M8638	Chronic multifocal osteomyelitis
M8639	Chronic multifocal osteomyelitis
M864	Chronic osteomyelitis with draining sinus
M8640	Chronic osteomyelitis with draining sinus
M8641	Chronic osteomyelitis with draining sinus
M8642	Chronic osteomyelitis with draining sinus
M8643	Chronic osteomyelitis with draining sinus
M8644	Chronic osteomyelitis with draining sinus
M8645	Chronic osteomyelitis with draining sinus
M8646	Chronic osteomyelitis with draining sinus
M8647	Chronic osteomyelitis with draining sinus
M8648	Chronic osteomyelitis with draining sinus
M8649	Chronic osteomyelitis with draining sinus
M865	Other chronic haematogenous osteomyelitis
M8650	Other chronic haematogenous osteomyelitis
M8651	Other chronic haematogenous osteomyelitis
M8652	Other chronic haematogenous osteomyelitis
M8653	Other chronic haematogenous osteomyelitis
M8654	Other chronic haematogenous osteomyelitis

For peer review only

M8655	Other chronic haematogenous osteomyelitis
M8656	Other chronic haematogenous osteomyelitis
M8657	Other chronic haematogenous osteomyelitis
M8658	Other chronic haematogenous osteomyelitis
M8659	Other chronic haematogenous osteomyelitis
M866	Other chronic osteomyelitis
M8660	Other chronic osteomyelitis
M8661	Other chronic osteomyelitis
M8662	Other chronic osteomyelitis
M8663	Other chronic osteomyelitis
M8664	Other chronic osteomyelitis
M8665	Other chronic osteomyelitis
M8666	Other chronic osteomyelitis
M8667	Other chronic osteomyelitis
M8668	Other chronic osteomyelitis
M8669	Other chronic osteomyelitis
M868	Other osteomyelitis
M8680	Other osteomyelitis
M8681	Other osteomyelitis
M8682	Other osteomyelitis
M8683	Other osteomyelitis
M8684	Other osteomyelitis
M8685	Other osteomyelitis
M8686	Other osteomyelitis
M8687	Other osteomyelitis
M8688	Other osteomyelitis
M8689	Other osteomyelitis
M869	Osteomyelitis, unspecified
M8690	Osteomyelitis, unspecified
M8691	Osteomyelitis, unspecified
M8692	Osteomyelitis, unspecified
M8693	Osteomyelitis, unspecified
M8694	Osteomyelitis, unspecified
M8695	Osteomyelitis, unspecified
M8696	Osteomyelitis, unspecified
M8697	Osteomyelitis, unspecified
M8698	Osteomyelitis, unspecified
M8699	Osteomyelitis, unspecified
M87	Osteonecrosis
M870	Idiopathic aseptic necrosis of bone
M8700	Idiopathic aseptic necrosis of bone
M8701	Idiopathic aseptic necrosis of bone
M8702	Idiopathic aseptic necrosis of bone
M8703	Idiopathic aseptic necrosis of bone
M8704	Idiopathic aseptic necrosis of bone
M8705	Idiopathic aseptic necrosis of bone
M8706	Idiopathic aseptic necrosis of bone
M8707	Idiopathic aseptic necrosis of bone

For peer review only

M8708	Idiopathic aseptic necrosis of bone
M8709	Idiopathic aseptic necrosis of bone
M871	Osteonecrosis due to drugs
M8710	Osteonecrosis due to drugs
M8711	Osteonecrosis due to drugs
M8712	Osteonecrosis due to drugs
M8713	Osteonecrosis due to drugs
M8714	Osteonecrosis due to drugs
M8715	Osteonecrosis due to drugs
M8716	Osteonecrosis due to drugs
M8717	Osteonecrosis due to drugs
M8718	Osteonecrosis due to drugs
M8719	Osteonecrosis due to drugs
M872	Osteonecrosis due to previous trauma
M8720	Osteonecrosis due to previous trauma
M8721	Osteonecrosis due to previous trauma
M8722	Osteonecrosis due to previous trauma
M8723	Osteonecrosis due to previous trauma
M8724	Osteonecrosis due to previous trauma
M8725	Osteonecrosis due to previous trauma
M8726	Osteonecrosis due to previous trauma
M8727	Osteonecrosis due to previous trauma
M8728	Osteonecrosis due to previous trauma
M8729	Osteonecrosis due to previous trauma
M873	Other secondary osteonecrosis
M8730	Other secondary osteonecrosis
M8731	Other secondary osteonecrosis
M8732	Other secondary osteonecrosis
M8733	Other secondary osteonecrosis
M8734	Other secondary osteonecrosis
M8735	Other secondary osteonecrosis
M8736	Other secondary osteonecrosis
M8737	Other secondary osteonecrosis
M8738	Other secondary osteonecrosis
M8739	Other secondary osteonecrosis
M878	Other osteonecrosis
M8780	Other osteonecrosis
M8781	Other osteonecrosis
M8782	Other osteonecrosis
M8783	Other osteonecrosis
M8784	Other osteonecrosis
M8785	Other osteonecrosis
M8786	Other osteonecrosis
M8787	Other osteonecrosis
M8788	Other osteonecrosis
M8789	Other osteonecrosis
M879	Osteonecrosis, unspecified
M8790	Osteonecrosis, unspecified

For peer review only

M8791	Osteonecrosis, unspecified
M8792	Osteonecrosis, unspecified
M8793	Osteonecrosis, unspecified
M8794	Osteonecrosis, unspecified
M8795	Osteonecrosis, unspecified
M8796	Osteonecrosis, unspecified
M8797	Osteonecrosis, unspecified
M8798	Osteonecrosis, unspecified
M8799	Osteonecrosis, unspecified
M88	Paget disease of bone [osteitis deformans]
M88	Paget's disease of bone [osteitis deformans]
M880	Paget disease of skull
M880	Paget's disease of skull
M888	Paget disease of other bones
M888	Paget's disease of other bones
M8880	Paget disease of other bones
M8880	Paget's disease of other bones
M8881	Paget disease of other bones
M8881	Paget's disease of other bones
M8882	Paget disease of other bones
M8882	Paget's disease of other bones
M8883	Paget disease of other bones
M8883	Paget's disease of other bones
M8884	Paget disease of other bones
M8884	Paget's disease of other bones
M8885	Paget disease of other bones
M8885	Paget's disease of other bones
M8886	Paget disease of other bones
M8886	Paget's disease of other bones
M8887	Paget disease of other bones
M8887	Paget's disease of other bones
M8888	Paget disease of other bones
M8888	Paget's disease of other bones
M8889	Paget disease of other bones
M8889	Paget's disease of other bones
M889	Paget disease of bone, unspecified
M889	Paget's disease of bone, unspecified
M8890	Paget disease of bone, unspecified
M8890	Paget's disease of bone, unspecified
M8891	Paget disease of bone, unspecified
M8891	Paget's disease of bone, unspecified
M8892	Paget disease of bone, unspecified
M8892	Paget's disease of bone, unspecified
M8893	Paget disease of bone, unspecified
M8893	Paget's disease of bone, unspecified
M8894	Paget disease of bone, unspecified
M8894	Paget's disease of bone, unspecified
M8895	Paget disease of bone, unspecified

For peer review only

M8895	Paget's disease of bone, unspecified
M8896	Paget disease of bone, unspecified
M8896	Paget's disease of bone, unspecified
M8897	Paget disease of bone, unspecified
M8897	Paget's disease of bone, unspecified
M8898	Paget disease of bone, unspecified
M8898	Paget's disease of bone, unspecified
M8899	Paget disease of bone, unspecified
M8899	Paget's disease of bone, unspecified
M89	Other disorders of bone
M890	Algoneurodystrophy
M8900	Algoneurodystrophy
M8901	Algoneurodystrophy
M8902	Algoneurodystrophy
M8903	Algoneurodystrophy
M8904	Algoneurodystrophy
M8905	Algoneurodystrophy
M8906	Algoneurodystrophy
M8907	Algoneurodystrophy
M8908	Algoneurodystrophy
M8909	Algoneurodystrophy
M891	Epiphyseal arrest
M8910	Epiphyseal arrest
M8911	Epiphyseal arrest
M8912	Epiphyseal arrest
M8913	Epiphyseal arrest
M8914	Epiphyseal arrest
M8915	Epiphyseal arrest
M8916	Epiphyseal arrest
M8917	Epiphyseal arrest
M8918	Epiphyseal arrest
M8919	Epiphyseal arrest
M892	Other disorders of bone development and growth
M8920	Other disorders of bone development and growth
M8921	Other disorders of bone development and growth
M8922	Other disorders of bone development and growth
M8923	Other disorders of bone development and growth
M8924	Other disorders of bone development and growth
M8925	Other disorders of bone development and growth
M8926	Other disorders of bone development and growth
M8927	Other disorders of bone development and growth
M8928	Other disorders of bone development and growth
M8929	Other disorders of bone development and growth
M893	Hypertrophy of bone
M8930	Hypertrophy of bone
M8931	Hypertrophy of bone
M8932	Hypertrophy of bone
M8933	Hypertrophy of bone

For peer review only

M8934	Hypertrophy of bone
M8935	Hypertrophy of bone
M8936	Hypertrophy of bone
M8937	Hypertrophy of bone
M8938	Hypertrophy of bone
M8939	Hypertrophy of bone
M894	Other hypertrophic osteoarthropathy
M8940	Other hypertrophic osteoarthropathy
M8941	Other hypertrophic osteoarthropathy
M8942	Other hypertrophic osteoarthropathy
M8943	Other hypertrophic osteoarthropathy
M8944	Other hypertrophic osteoarthropathy
M8945	Other hypertrophic osteoarthropathy
M8946	Other hypertrophic osteoarthropathy
M8947	Other hypertrophic osteoarthropathy
M8948	Other hypertrophic osteoarthropathy
M8949	Other hypertrophic osteoarthropathy
M895	Osteolysis
M8950	Osteolysis
M8951	Osteolysis
M8952	Osteolysis
M8953	Osteolysis
M8954	Osteolysis
M8955	Osteolysis
M8956	Osteolysis
M8957	Osteolysis
M8958	Osteolysis
M8959	Osteolysis
M896	Osteopathy after poliomyelitis
M8960	Osteopathy after poliomyelitis
M8961	Osteopathy after poliomyelitis
M8962	Osteopathy after poliomyelitis
M8963	Osteopathy after poliomyelitis
M8964	Osteopathy after poliomyelitis
M8965	Osteopathy after poliomyelitis
M8966	Osteopathy after poliomyelitis
M8967	Osteopathy after poliomyelitis
M8968	Osteopathy after poliomyelitis
M8969	Osteopathy after poliomyelitis
M898	Other specified disorders of bone
M8980	Other specified disorders of bone
M8981	Other specified disorders of bone
M8982	Other specified disorders of bone
M8983	Other specified disorders of bone
M8984	Other specified disorders of bone
M8985	Other specified disorders of bone
M8986	Other specified disorders of bone
M8987	Other specified disorders of bone

For peer review only

M8988	Other specified disorders of bone
M8989	Other specified disorders of bone
M899	Disorder of bone, unspecified
M8990	Disorder of bone, unspecified
M8991	Disorder of bone, unspecified
M8992	Disorder of bone, unspecified
M8993	Disorder of bone, unspecified
M8994	Disorder of bone, unspecified
M8995	Disorder of bone, unspecified
M8996	Disorder of bone, unspecified
M8997	Disorder of bone, unspecified
M8998	Disorder of bone, unspecified
M8999	Disorder of bone, unspecified
M90	Osteopathies in diseases classified elsewhere
M900	Tuberculosis of bone
M9000	Tuberculosis of bone
M9001	Tuberculosis of bone
M9002	Tuberculosis of bone
M9003	Tuberculosis of bone
M9004	Tuberculosis of bone
M9005	Tuberculosis of bone
M9006	Tuberculosis of bone
M9007	Tuberculosis of bone
M9008	Tuberculosis of bone
M9009	Tuberculosis of bone
M901	Periostitis in other infectious diseases classified elsewhere
M9010	Periostitis in other infectious diseases classified elsewhere
M9011	Periostitis in other infectious diseases classified elsewhere
M9012	Periostitis in other infectious diseases classified elsewhere
M9013	Periostitis in other infectious diseases classified elsewhere
M9014	Periostitis in other infectious diseases classified elsewhere
M9015	Periostitis in other infectious diseases classified elsewhere
M9016	Periostitis in other infectious diseases classified elsewhere
M9017	Periostitis in other infectious diseases classified elsewhere
M9018	Periostitis in other infectious diseases classified elsewhere
M9019	Periostitis in other infectious diseases classified elsewhere
M902	Osteopathy in other infectious diseases classified elsewhere
M9020	Osteopathy in other infectious diseases classified elsewhere
M9021	Osteopathy in other infectious diseases classified elsewhere
M9022	Osteopathy in other infectious diseases classified elsewhere
M9023	Osteopathy in other infectious diseases classified elsewhere
M9024	Osteopathy in other infectious diseases classified elsewhere
M9025	Osteopathy in other infectious diseases classified elsewhere
M9026	Osteopathy in other infectious diseases classified elsewhere
M9027	Osteopathy in other infectious diseases classified elsewhere
M9028	Osteopathy in other infectious diseases classified elsewhere
M9029	Osteopathy in other infectious diseases classified elsewhere
M903	Osteonecrosis in caisson disease

For peer review only

M9030	Osteonecrosis in caisson disease
M9031	Osteonecrosis in caisson disease
M9032	Osteonecrosis in caisson disease
M9033	Osteonecrosis in caisson disease
M9034	Osteonecrosis in caisson disease
M9035	Osteonecrosis in caisson disease
M9036	Osteonecrosis in caisson disease
M9037	Osteonecrosis in caisson disease
M9038	Osteonecrosis in caisson disease
M9039	Osteonecrosis in caisson disease
M904	Osteonecrosis due to haemoglobinopathy
M9040	Osteonecrosis due to haemoglobinopathy
M9041	Osteonecrosis due to haemoglobinopathy
M9042	Osteonecrosis due to haemoglobinopathy
M9043	Osteonecrosis due to haemoglobinopathy
M9044	Osteonecrosis due to haemoglobinopathy
M9045	Osteonecrosis due to haemoglobinopathy
M9046	Osteonecrosis due to haemoglobinopathy
M9047	Osteonecrosis due to haemoglobinopathy
M9048	Osteonecrosis due to haemoglobinopathy
M9049	Osteonecrosis due to haemoglobinopathy
M905	Osteonecrosis in other diseases classified elsewhere
M9050	Osteonecrosis in other diseases classified elsewhere
M9051	Osteonecrosis in other diseases classified elsewhere
M9052	Osteonecrosis in other diseases classified elsewhere
M9053	Osteonecrosis in other diseases classified elsewhere
M9054	Osteonecrosis in other diseases classified elsewhere
M9055	Osteonecrosis in other diseases classified elsewhere
M9056	Osteonecrosis in other diseases classified elsewhere
M9057	Osteonecrosis in other diseases classified elsewhere
M9058	Osteonecrosis in other diseases classified elsewhere
M9059	Osteonecrosis in other diseases classified elsewhere
M906	Osteitis deformans in neoplastic disease
M9060	Osteitis deformans in neoplastic disease
M9061	Osteitis deformans in neoplastic disease
M9062	Osteitis deformans in neoplastic disease
M9063	Osteitis deformans in neoplastic disease
M9064	Osteitis deformans in neoplastic disease
M9065	Osteitis deformans in neoplastic disease
M9066	Osteitis deformans in neoplastic disease
M9067	Osteitis deformans in neoplastic disease
M9068	Osteitis deformans in neoplastic disease
M9069	Osteitis deformans in neoplastic disease
M907	Fracture of bone in neoplastic disease
M9070	Fracture of bone in neoplastic disease
M9071	Fracture of bone in neoplastic disease
M9072	Fracture of bone in neoplastic disease
M9073	Fracture of bone in neoplastic disease

For peer review only

M9074	Fracture of bone in neoplastic disease
M9075	Fracture of bone in neoplastic disease
M9076	Fracture of bone in neoplastic disease
M9077	Fracture of bone in neoplastic disease
M9078	Fracture of bone in neoplastic disease
M9079	Fracture of bone in neoplastic disease
M908	Osteopathy in other diseases classified elsewhere
M9080	Osteopathy in other diseases classified elsewhere
M9081	Osteopathy in other diseases classified elsewhere
M9082	Osteopathy in other diseases classified elsewhere
M9083	Osteopathy in other diseases classified elsewhere
M9084	Osteopathy in other diseases classified elsewhere
M9085	Osteopathy in other diseases classified elsewhere
M9086	Osteopathy in other diseases classified elsewhere
M9087	Osteopathy in other diseases classified elsewhere
M9088	Osteopathy in other diseases classified elsewhere
M9089	Osteopathy in other diseases classified elsewhere
M91	Juvenile osteochondrosis of hip and pelvis
M910	Juvenile osteochondrosis of pelvis
M911	Juvenile osteochondrosis of head of femur [Legg-Calv,-Perthes]
M912	Coxa plana
M913	Pseudocoxalgia
M918	Other juvenile osteochondrosis of hip and pelvis
M9180	Other juvenile osteochondrosis of hip and pelvis
M919	Juvenile osteochondrosis of hip and pelvis, unspecified
M9190	Juvenile osteochondrosis of hip and pelvis, unspecified
M92	Other juvenile osteochondrosis
M920	Juvenile osteochondrosis of humerus
M921	Juvenile osteochondrosis of radius and ulna
M922	Juvenile osteochondrosis of hand
M923	Other juvenile osteochondrosis of upper limb
M924	Juvenile osteochondrosis of patella
M925	Juvenile osteochondrosis of tibia and fibula
M926	Juvenile osteochondrosis of tarsus
M927	Juvenile osteochondrosis of metatarsus
M928	Other specified juvenile osteochondrosis
M929	Juvenile osteochondrosis, unspecified
M93	Other osteochondropathies
M930	Slipped upper femoral epiphysis (nontraumatic)
M931	Kienb”ck disease of adults
M931	Kienb”ck's disease of adults
M932	Osteochondritis dissecans
M938	Other specified osteochondropathies
M939	Osteochondropathy, unspecified
M94	Other disorders of cartilage
M940	Chondrocostal junction syndrome [Tietze]
M941	Relapsing polychondritis
M9410	Relapsing polychondritis

For peer review only

M9411	Relapsing polychondritis
M9412	Relapsing polychondritis
M9413	Relapsing polychondritis
M9414	Relapsing polychondritis
M9415	Relapsing polychondritis
M9416	Relapsing polychondritis
M9417	Relapsing polychondritis
M9418	Relapsing polychondritis
M9419	Relapsing polychondritis
M942	Chondromalacia
M9420	Chondromalacia
M9421	Chondromalacia
M9422	Chondromalacia
M9423	Chondromalacia
M9424	Chondromalacia
M9425	Chondromalacia
M9426	Chondromalacia
M9427	Chondromalacia
M9428	Chondromalacia
M9429	Chondromalacia
M943	Chondrolysis
M9430	Chondrolysis
M9431	Chondrolysis
M9432	Chondrolysis
M9433	Chondrolysis
M9434	Chondrolysis
M9435	Chondrolysis
M9436	Chondrolysis
M9437	Chondrolysis
M9438	Chondrolysis
M9439	Chondrolysis
M948	Other specified disorders of cartilage
M9480	Other specified disorders of cartilage
M9481	Other specified disorders of cartilage
M9482	Other specified disorders of cartilage
M9483	Other specified disorders of cartilage
M9484	Other specified disorders of cartilage
M9485	Other specified disorders of cartilage
M9486	Other specified disorders of cartilage
M9487	Other specified disorders of cartilage
M9488	Other specified disorders of cartilage
M9489	Other specified disorders of cartilage
M949	Disorder of cartilage, unspecified
M9490	Disorder of cartilage, unspecified
M9491	Disorder of cartilage, unspecified
M9492	Disorder of cartilage, unspecified
M9493	Disorder of cartilage, unspecified
M9494	Disorder of cartilage, unspecified

For peer review only

M9495	Disorder of cartilage, unspecified
M9496	Disorder of cartilage, unspecified
M9497	Disorder of cartilage, unspecified
M9498	Disorder of cartilage, unspecified
M9499	Disorder of cartilage, unspecified
M95	Other acquired deformities of musculoskeletal system and connective tissue
M950	Acquired deformity of nose
M951	Cauliflower ear
M952	Other acquired deformity of head
M953	Acquired deformity of neck
M954	Acquired deformity of chest and rib
M955	Acquired deformity of pelvis
M958	Other specified acquired deformities of musculoskeletal system
M959	Acquired deformity of musculoskeletal system, unspecified
M96	Postprocedural musculoskeletal disorders, not elsewhere classified
M960	Pseudarthrosis after fusion or arthrodesis
M961	Postlaminectomy syndrome, not elsewhere classified
M962	Postradiation kyphosis
M963	Postlaminectomy kyphosis
M964	Postsurgical lordosis
M965	Postradiation scoliosis
M966	Fracture of bone following insertion of orthopaedic implant, joint prosthesis, or bone plate
M968	Other postprocedural musculoskeletal disorders
M969	Postprocedural musculoskeletal disorder, unspecified
M99	Biomechanical lesions, not elsewhere classified
M990	Segmental and somatic dysfunction
M9900	Segmental and somatic dysfunction
M9901	Segmental and somatic dysfunction
M9902	Segmental and somatic dysfunction
M9903	Segmental and somatic dysfunction
M9904	Segmental and somatic dysfunction
M9905	Segmental and somatic dysfunction
M9906	Segmental and somatic dysfunction
M9907	Segmental and somatic dysfunction
M9908	Segmental and somatic dysfunction
M9909	Segmental and somatic dysfunction
M991	Subluxation complex (vertebral)
M9910	Subluxation complex (vertebral)
M9911	Subluxation complex (vertebral)
M9912	Subluxation complex (vertebral)
M9913	Subluxation complex (vertebral)
M9914	Subluxation complex (vertebral)
M9915	Subluxation complex (vertebral)
M9916	Subluxation complex (vertebral)
M9917	Subluxation complex (vertebral)
M9918	Subluxation complex (vertebral)
M9919	Subluxation complex (vertebral)

For peer review only

M992	Subluxation stenosis of neural canal
M9920	Subluxation stenosis of neural canal
M9921	Subluxation stenosis of neural canal
M9922	Subluxation stenosis of neural canal
M9923	Subluxation stenosis of neural canal
M9924	Subluxation stenosis of neural canal
M9925	Subluxation stenosis of neural canal
M993	Osseous stenosis of neural canal
M9930	Osseous stenosis of neural canal
M9931	Osseous stenosis of neural canal
M9932	Osseous stenosis of neural canal
M9933	Osseous stenosis of neural canal
M9934	Osseous stenosis of neural canal
M9935	Osseous stenosis of neural canal
M994	Connective tissue stenosis of neural canal
M9940	Connective tissue stenosis of neural canal
M9941	Connective tissue stenosis of neural canal
M9942	Connective tissue stenosis of neural canal
M9943	Connective tissue stenosis of neural canal
M9944	Connective tissue stenosis of neural canal
M9945	Connective tissue stenosis of neural canal
M995	Intervertebral disc stenosis of neural canal
M9950	Intervertebral disc stenosis of neural canal
M9951	Intervertebral disc stenosis of neural canal
M9952	Intervertebral disc stenosis of neural canal
M9953	Intervertebral disc stenosis of neural canal
M9954	Intervertebral disc stenosis of neural canal
M9955	Intervertebral disc stenosis of neural canal
M996	Osseous and subluxation stenosis of intervertebral foramina
M9960	Osseous and subluxation stenosis of intervertebral foramina
M9961	Osseous and subluxation stenosis of intervertebral foramina
M9962	Osseous and subluxation stenosis of intervertebral foramina
M9963	Osseous and subluxation stenosis of intervertebral foramina
M9964	Osseous and subluxation stenosis of intervertebral foramina
M9965	Osseous and subluxation stenosis of intervertebral foramina
M997	Connective tissue and disc stenosis of intervertebral foramina
M9970	Connective tissue and disc stenosis of intervertebral foramina
M9971	Connective tissue and disc stenosis of intervertebral foramina
M9972	Connective tissue and disc stenosis of intervertebral foramina
M9973	Connective tissue and disc stenosis of intervertebral foramina
M9974	Connective tissue and disc stenosis of intervertebral foramina
M9975	Connective tissue and disc stenosis of intervertebral foramina
M998	Other biomechanical lesions
M9980	Other biomechanical lesions
M9981	Other biomechanical lesions
M9982	Other biomechanical lesions
M9983	Other biomechanical lesions
M9984	Other biomechanical lesions

For peer review only

M9985	Other biomechanical lesions
M9986	Other biomechanical lesions
M9987	Other biomechanical lesions
M9988	Other biomechanical lesions
M9989	Other biomechanical lesions
M999	Biomechanical lesion, unspecified
M9990	Biomechanical lesion, unspecified
M9991	Biomechanical lesion, unspecified
M9992	Biomechanical lesion, unspecified
M9993	Biomechanical lesion, unspecified
M9994	Biomechanical lesion, unspecified
M9995	Biomechanical lesion, unspecified
M9996	Biomechanical lesion, unspecified
M9997	Biomechanical lesion, unspecified
M9998	Biomechanical lesion, unspecified
M9999	Biomechanical lesion, unspecified

Epilepsy (GP)

READ_CODE	READ_DESC
1O30.	Epilepsy confirmed
F25..	Epilepsy
F250.	Generalised nonconvulsive epilepsy
F2500	Petit mal (minor) epilepsy
F2501	Pykno-epilepsy
F2502	Epileptic seizures - atonic
F2503	Epileptic seizures - akinetic
F2504	Juvenile absence epilepsy
F2505	Lennox-Gastaut syndrome
F250y	Other specified generalised nonconvulsive epilepsy
F250z	Generalised nonconvulsive epilepsy NOS
F251.	Generalised convulsive epilepsy
F2510	Grand mal (major) epilepsy
F2511	Neonatal myoclonic epilepsy
F2512	Epileptic seizures - clonic
F2513	Epileptic seizures - myoclonic
F2514	Epileptic seizures - tonic
F2515	Tonic-clonic epilepsy
F2516	Grand mal seizure
F251y	Other specified generalised convulsive epilepsy
F251z	Generalised convulsive epilepsy NOS
F252.	Petit mal status
F253.	Grand mal status
F254.	Partial epilepsy with impairment of consciousness
F2540	Temporal lobe epilepsy
F2541	Psychomotor epilepsy

For peer review only

F2542	Psychosensory epilepsy
F2543	Limbic system epilepsy
F2544	Epileptic automatism
F2545	Complex partial epileptic seizure
F254z	Partial epilepsy with impairment of consciousness NOS
F255.	Partial epilepsy without mention of impairment of consciousness
F2550	Jacksonian, focal or motor epilepsy
F2551	Sensory induced epilepsy
F2552	Somatosensory epilepsy
F2553	Visceral reflex epilepsy
F2554	Visual reflex epilepsy
F2555	Unilateral epilepsy
F2556	Simple partial epileptic seizure
F255y	Other specified partial epilepsy without mention of impairment of consciousness
F255z	Partial epilepsy without mention of impairment of consciousness NOS
F256.	Infantile spasms
F2560	Hypsarrhythmia
F2561	Salaam attacks
F256z	Infantile spasms NOS
F257.	Kojevnikov's epilepsy
F258.	Post-ictal state
F259.	Early infantile epileptic encephalopathy with suppression bursts
F25A.	Juvenile myoclonic epilepsy
F25B.	Alcohol-induced epilepsy
F25C.	Drug-induced epilepsy
F25D.	Menstrual epilepsy
F25E.	Stress-induced epilepsy
F25F.	Photosensitive epilepsy
F25G.	Severe myoclonic epilepsy in infancy
F25H.	Generalised seizure
F25X.	Status epilepticus, unspecified
F25y.	Other forms of epilepsy
F25y0	Cursive (running) epilepsy
F25y1	Gelastic epilepsy
F25y2	Localization-related(focal)(partial)idiopathic epilepsy and epileptic syndromes with seizures of localised onset
F25y3	Complex partial status epilepticus
F25y4	Benign Rolandic epilepsy
F25y5	Panayiotopoulos syndrome
F25yz	Other forms of epilepsy NOS
F25z.	Epilepsy NOS
dn...	CONTROL OF EPILEPSY
dn1..	ACETAZOLAMIDE [EPILEPSY]
dn11.	DIAMOX [EP] 500mg m/r capsules
dn12.	DIAMOX [EP] 250mg tablets
dn13.	DIAMOX [EP] 500mg injection
dn1x.	ACETAZOLAMIDE [EP] 500mg m/r capsules
dn1y.	ACETAZOLAMIDE [EP] 250mg tablets

For peer review only

dn1z.	ACETAZOLAMIDE [EP] 500mg injection
dn2..	*BECLAMIDE
dn21.	*NYDRANE 500mg tablets
dn2z.	*BECLAMIDE 500mg tablets
dn3..	CARBAMAZEPINE
dn31.	CARBAMAZEPINE 100mg tablets
dn32.	CARBAMAZEPINE 200mg tablets
dn33.	CARBAMAZEPINE 400mg tablets
dn34.	TEGRETOL 100mg tablets
dn35.	TEGRETOL 200mg tablets
dn36.	TEGRETOL 400mg tablets
dn37.	TEGRETOL 100mg/5mL sugar free liquid
dn38.	TEGRETOL RETARD 200mg m/r tabs
dn39.	TEGRETOL RETARD 400mg m/r tabs
dn3A.	*EPIMAZ 100mg tablets
dn3B.	*EPIMAZ 200mg tablets
dn3C.	*EPIMAZ 400mg tablets
dn3D.	TEGRETOL 125mg suppositories
dn3E.	TEGRETOL 250mg suppositories
dn3F.	TIMONIL RETARD 200mg m/r tablets
dn3G.	TIMONIL RETARD 400mg m/r tablets
dn3H.	*TERIL CR 200mg m/r tablets
dn3I.	*TERIL CR 400mg m/r tablets
dn3J.	CARBAGEN SR 200mg m/r tablets
dn3K.	CARBAGEN SR 400mg m/r tablets
dn3a.	CARBAMAZEPINE 200mg m/r tabs
dn3b.	CARBAMAZEPINE 400mg m/r tabs
dn3c.	TEGRETOL 100mg chewable tablets
dn3c.	TEGRETOL 100mg chewable tablets
dn3d.	TEGRETOL 200mg chewable tablets
dn3d.	TEGRETOL 200mg chewable tablets
dn3e.	*ARBIL MR 200mg m/r tablets
dn3f.	*ARBIL MR 400mg m/r tablets
dn3v.	CARBAMAZEPINE 125mg suppositories
dn3w.	CARBAMAZEPINE 250mg suppositories
dn3x.	CARBAMAZEPINE 200mg chewable tablets
dn3x.	CARBAMAZEPINE 200mg chewable tablets
dn3y.	CARBAMAZEPINE 100mg chewable tablets
dn3y.	CARBAMAZEPINE 100mg chewable tablets
dn3z.	CARBAMAZEPINE 100mg/5mL sugar free liquid
dn4..	CLONAZEPAM [EPILEPSY CONTROL]
dn41.	*RIVOTRIL 500mcg tablets
dn41.	RIVOTRIL 500micrograms tablets
dn42.	RIVOTRIL 2mg tablets
dn42.	*RIVOTRIL 2mg tablets
dn4w.	CLONAZEPAM 0.5mg/5mL sugar free oral solution
dn4x.	CLONAZEPAM 2mg/5mL sugar free oral solution
dn4y.	CLONAZEPAM 500microgram tablets

For peer review only

dn4z.	CLONAZEPAM 2mg tablets
dn5..	ETHOSUXIMIDE
dn51.	*ETHOSUXIMIDE 250mg capsules
dn52.	*ETHOSUXIMIDE 250mg/5mL elixir
dn53.	*EMESIDE 250mg capsules
dn54.	EMESIDE 250mg/5mL syrup
dn55.	*ZARONTIN 250mg capsules
dn56.	ZARONTIN 250mg/5mL syrup
dn5x.	ETHOSUXIMIDE 250mg capsules
dn5y.	*ETHOSUXIMIDE 250mg/5mL elixir
dn5z.	ETHOSUXIMIDE 250mg/5mL syrup
dn6..	METHYLPHENOBARBITAL
dn61.	*PROMINAL 30mg tablets
dn62.	*PROMINAL 60mg tablets
dn63.	*PROMINAL 200mg tablets
dn6x.	METHYLPHENOBARBITONE 30mg tablets
dn6y.	METHYLPHENOBARBITONE 60mg tablets
dn6z.	METHYLPHENOBARBITONE 200mg tablets
dn7..	PHENOBARBITAL
dn71.	PHENOBARBITAL 15mg tablets
dn72.	PHENOBARBITAL 30mg tablets
dn73.	PHENOBARBITAL 60mg tablets
dn74.	PHENOBARBITAL 100mg tablets
dn75.	PHENOBARBITONE SODIUM 30mg tablets
dn76.	PHENOBARBITONE SODIUM 60mg tablets
dn77.	PHENOBARBITONE 15mg/10mL elixir
dn78.	PHENOBARBITAL 200mg/1mL injection
dn79.	*GARDENAL 200mg/1mL injection
dn7a.	*LUMINAL 15mg tablets
dn7b.	*LUMINAL 30mg tablets
dn7c.	*LUMINAL 60mg tablets
dn7d.	PHENOBARBITAL 15mg/5mL elixir
dn8..	PHENYTOIN
dn81.	EPANUTIN 30mg/5mL suspension
dn82.	EPANUTIN 50mg Infatabs
dn83.	PHENYTOIN 50mg chewable tablets
dn8y.	PHENYTOIN 30mg/5mL suspension
dn8z.	PHENYTOIN 90mg/5mL sugar free suspension
dn9..	PHENYTOIN SODIUM
dn91.	PHENYTOIN 50mg tablets
dn92.	PHENYTOIN 100mg tablets
dn93.	EPANUTIN 25mg capsules
dn93.	*EPANUTIN 25mg capsules
dn94.	EPANUTIN 50mg capsules
dn94.	*EPANUTIN 50mg capsules
dn95.	EPANUTIN 100mg capsules
dn95.	*EPANUTIN 100mg capsules
dn96.	*EPANUTIN 300mg capsules

For peer review only

dn96.	EPANUTIN 300mg capsules
dn97.	*PENTRAN 50mg tablets
dn98.	*PENTRAN 100mg tablets
dn9w.	PHENYTOIN SODIUM 300mg capsules
dn9x.	PHENYTOIN SODIUM 25mg caps
dn9y.	PHENYTOIN SODIUM 50mg capsules
dn9z.	PHENYTOIN SODIUM 100mg capsules
dna..	PRIMIDONE
dna1.	MYSOLINE 250mg tablets
dna1.	*MYSOLINE 250mg tablets
dna2.	MYSOLINE 250mg/5mL oral suspension
dna3.	*MYSOLINE 50mg tablets
dna3.	MYSOLINE 50mg tablets
dnax.	PRIMIDONE 50mg tablets
dnay.	PRIMIDONE 250mg tablets
dnaz.	PRIMIDONE 250mg/5mL oral suspension
dnb..	SODIUM VALPROATE
dnb1.	EPILIM 100mg crushable tablets
dnb2.	EPILIM 200mg e/c tablets
dnb3.	EPILIM 500mg e/c tablets
dnb4.	EPILIM 200mg/5mL sugar free liquid
dnb5.	EPILIM 200mg/5mL syrup
dnb6.	EPILIM IV 400mg/4mL injection
dnb7.	SODIUM VALPROATE 200mg e/c tablets
dnb8.	SODIUM VALPROATE 500mg e/c tablets
dnb9.	ORLEPT STARTER PACK 200mg e/c tablets x10
dnbA.	ORLEPT 200mg/5mL sugar free liquid
dnbB.	EPIVAL CR 300mg m/r tablets
dnbC.	EPIVAL CR 500mg m/r tablets
dnbD.	EPISENTA 300mg/3mL solution for injection
dnbE.	SODIUM VALPROATE 300mg/3mL solution for injection
dnbF.	EPISENTA 150mg m/r capsules
dnbG.	EPISENTA 300mg m/r capsules
dnbH.	EPISENTA 500mg/sachet m/r granules
dnbI.	EPISENTA 1g/sachet m/r granules
dnbJ.	SODIUM VALPROATE 150mg m/r capsules
dnbK.	SODIUM VALPROATE 300mg m/r capsules
dnbL.	SODIUM VALPROATE 500mg/sachet m/r granules
dnbM.	SODIUM VALPROATE 1g/sachet m/r granules
dnbN.	SODIUM VALPROATE 1g/10mL solution for injection
dnbO.	EPISENTA 1g/10mL solution for injection
dnbP.	EPILIM CHRONOSPHERE 50mg/sachet m/r granules
dnbQ.	EPILIM CHRONOSPHERE 100mg/sachet m/r granules
dnbR.	EPILIM CHRONOSPHERE 250mg/sachet m/r granules
dnbS.	EPILIM CHRONOSPHERE 500mg/sachet m/r granules
dnbT.	EPILIM CHRONOSPHERE 750mg/sachet m/r granules
dnbU.	EPILIM CHRONOSPHERE 1g/sachet m/r granules
dnba.	*ORLEPT 200mg e/c tablets

For peer review only

dnbb.	*ORLEPT 500mg e/c tablets
dnbc.	EPILIM CHRONO 200 m/r tablets
dnbd.	EPILIM CHRONO 300 m/r tablets
dnbe.	EPILIM CHRONO 500 m/r tablets
dnbn.	SODIUM VALPROATE 50mg/sachet m/r granules
dnbo.	SODIUM VALPROATE 100mg/sachet m/r granules
dnbp.	SODIUM VALPROATE 250mg/sachet m/r granules
dnbq.	SODIUM VALPROATE 750mg/sachet m/r granules
dnbr.	SODIUM VALPROATE 200mg m/r tablets
dnbs.	SODIUM VALPROATE 300mg m/r tablets
dnbt.	SODIUM VALPROATE 500mg m/r tablets
dnbu.	SODIUM VALPROATE 400mg/4mL injection
dnbv.	SODIUM VALPROATE 100mg crushable tablets
dnbw.	SODIUM VALPROATE 200mg crushable tablets
dnbx.	SODIUM VALPROATE 500mg tablets
dnby.	SODIUM VALPROATE 200mg/5mL sugar free liquid
dnbz.	SODIUM VALPROATE 200mg/5mL syrup
dnc..	CLOBAZAM [EPILEPSY ONLY]
dnc1.	*CLOBAZAM SLS 10mg capsules
dne..	VIGABATRIN
dne1.	VIGABATRIN 500mg tablets
dne2.	SABRIL 500mg tablets
dne3.	VIGABATRIN 500mg powder sachets
dne4.	SABRIL 500mg powder sachets
dnf..	LAMOTRIGINE
dnf1.	LAMOTRIGINE 50mg tablets
dnf2.	LAMOTRIGINE 100mg tablets
dnf3.	LAMICTAL 50mg tablets
dnf4.	LAMICTAL 100mg tablets
dnf5.	LAMOTRIGINE 25mg tablets
dnf6.	LAMICTAL 25mg tablets
dnf7.	LAMICTAL 5mg dispersible tablets
dnf8.	LAMICTAL 25mg dispersible tablets
dnf9.	LAMICTAL 100mg dispersible tablets
dnfA.	LAMOTRIGINE 5mg dispersible tablets
dnfB.	LAMOTRIGINE 25mg dispersible tablets
dnfC.	LAMOTRIGINE 100mg dispersible tablets
dnfD.	LAMICTAL 200mg tablets
dnfE.	LAMOTRIGINE 200mg tablets
dnfF.	LAMICTAL MONOTHERAPY 25mg starter pack
dnfG.	LAMICTAL VALPROATE ADD-ON 25mg starter pack
dnfH.	LAMICTAL NON-VALPROATE ADD-ON 50mg starter pack
dnfJ.	LAMICTAL 2mg dispersible tablets
dnfz.	LAMOTRIGINE 2mg dispersible tablets
dng..	PIRACETAM
dng1.	NOOTROPIL 800mg tablets
dng2.	NOOTROPIL 1.2g tablets
dng3.	NOOTROPIL 33% oral solution

For peer review only

dng4.	PIRACETAM 800mg tablets
dng5.	PIRACETAM 1.2g tablets
dng6.	PIRACETAM 333.3mg/mL oral solution
dnh..	VALPROIC ACID
dnh1.	CONVULEX 150mg e/c capsules
dnh2.	CONVULEX 300mg e/c capsules
dnh3.	CONVULEX 500mg e/c capsules
dnh4.	VALPROIC ACID 150mg e/c capsules
dnh5.	VALPROIC ACID 300mg e/c capsules
dnh6.	VALPROIC ACID 500mg e/c capsules
dnh7.	DEPAKOTE 250mg e/c tablets
dnh8.	DEPAKOTE 500mg e/c tablets
dnhy.	VALPROIC ACID 500mg e/c tablets
dnhz.	VALPROIC ACID 250mg e/c tablets
dni..	FOSPHENYTOIN SODIUM
dni1.	FOSPHENYTOIN SODIUM 750mg/10mL injection concentrate
dni2.	PRO-EPANUTIN 750mg/10mL injection concentrate
dnj..	GABAPENTIN
dnj1.	GABAPENTIN 100mg capsules
dnj2.	GABAPENTIN 300mg capsules
dnj3.	GABAPENTIN 400mg capsules
dnj4.	NEURONTIN 100mg capsules
dnj5.	NEURONTIN 300mg capsules
dnj6.	NEURONTIN 400mg capsules
dnj7.	NEURONTIN 600mg tablets
dnj8.	NEURONTIN 800mg tablets
dnj9.	NEURONTIN 300mg capsules/600mg tablets titration pack
dnjA.	GABAPENTIN 50mg/mL oral solution
dnjx.	GABAPENTIN 300mg capsules/600mg tablets titration pack
dnjy.	GABAPENTIN 600mg tablets
dnjz.	GABAPENTIN 800mg tablets
dnk..	TOPIRAMATE
dnk1.	TOPIRAMATE 50mg tablets
dnk2.	TOPIRAMATE 100mg tablets
dnk3.	TOPIRAMATE 200mg tablets
dnk4.	TOPAMAX 50mg tablets
dnk5.	TOPAMAX 100mg tablets
dnk6.	TOPAMAX 200mg tablets
dnk7.	TOPIRAMATE 25mg tablets
dnk8.	TOPAMAX 25mg tablets
dnk9.	TOPIRAMATE 15mg beads in capsules
dnkA.	TOPIRAMATE 25mg beads in capsules
dnkB.	TOPAMAX SPRINKLE 15mg capsules
dnkC.	TOPAMAX SPRINKLE 25mg capsules
dnkD.	TOPIRAMATE 50mg beads in capsules
dnkE.	TOPAMAX SPRINKLE 50mg capsules
dnl..	TIAGABINE
dnl1.	TIAGABINE 5mg tablets

For peer review only

dnl2.	TIAGABINE 10mg tablets
dnl3.	TIAGABINE 15mg tablets
dnl4.	GABITRIL 5mg tablets
dnl5.	GABITRIL 10mg tablets
dnl6.	GABITRIL 15mg tablets
dnm..	OXCARBAZEPINE
dnm1.	TRILEPTAL 150 tablets
dnm2.	TRILEPTAL 300 tablets
dnm3.	TRILEPTAL 600 tablets
dnm4.	TRILEPTAL 60mg/mL sugar free oral suspension
dnmw.	OXCARBAZEPINE 60mg/mL sugar free oral suspension
dnmx.	OXCARBAZEPINE 150mg tablets
dnmy.	OXCARBAZEPINE 300mg tablets
dnmz.	OXCARBAZEPINE 600mg tablets
dno..	LEVETIRACETAM
dno1.	KEPPRA 250mg tablets
dno2.	KEPPRA 500mg tablets
dno3.	KEPPRA 1g tablets
dno4.	KEPPRA 750mg tablets
dno5.	KEPPRA 100mg/mL s/f oral solution
dno6.	KEPPRA 500mg/5mL solution for injection
dno7.	DESITREND 250mg/sachet granules
dno8.	DESITREND 500mg/sachet granules
dno9.	DESITREND 1g/sachet granules
dnoA.	DESITREND 100mg/mL oral solution
dnoB.	DESITREND 500mg/5mL concentrate for solution for infusion
dnor.	LEVETIRACETAM 1g/sachet granules
dnos.	LEVETIRACETAM 500mg/sachet granules
dnot.	LEVETIRACETAM 250mg/sachet granules
dnou.	LEVETIRACETAM 500mg/5mL solution for injection
dnov.	LEVETIRACETAM 100mg/mL s/f oral solution
dnow.	LEVETIRACETAM 750mg tablets
dnox.	LEVETIRACETAM 1g tablets
dnoy.	LEVETIRACETAM 500mg tablets
dnoz.	LEVETIRACETAM 250mg tablets
dnp..	PREGABALIN
dnp1.	LYRICA 25mg capsules
dnp2.	LYRICA 50mg capsules
dnp3.	LYRICA 75mg capsules
dnp4.	LYRICA 100mg capsules
dnp5.	LYRICA 150mg capsules
dnp6.	LYRICA 200mg capsules
dnp7.	LYRICA 300mg capsules
dnp8.	LYRICA 225mg capsules
dnp9.	LYRICA 20mg/1mL oral solution
dnpA.	LECAENT 25mg capsules
dnpB.	LECAENT 50mg capsules
dnpC.	LECAENT 75mg capsules

For peer review only

dnpD.	LECAENT 100mg capsules
dnpE.	LECAENT 150mg capsules
dnpF.	LECAENT 200mg capsules
dnpG.	LECAENT 225mg capsules
dnpH.	LECAENT 300mg capsules
dnpI.	REWISCA 25mg capsules
dnpJ.	REWISCA 50mg capsules
dnpK.	REWISCA 75mg capsules
dnpL.	REWISCA 100mg capsules
dnpM.	REWISCA 150mg capsules
dnpN.	REWISCA 200mg capsules
dnpO.	REWISCA 225mg capsules
dnpP.	REWISCA 300mg capsules
dnpr.	PREGABALIN 20mg/1mL oral solution
dnps.	PREGABALIN 225mg capsules
dnpt.	PREGABALIN 300mg capsules
dnpu.	PREGABALIN 200mg capsules
dnpv.	PREGABALIN 100mg capsules
dnpw.	PREGABALIN 150mg capsules
dnpx.	PREGABALIN 75mg capsules
dnpy.	PREGABALIN 50mg capsules
dnpz.	PREGABALIN 25mg capsules
dnq..	ZONISAMIDE
dnq1.	ZONISAMIDE 25mg capsules
dnq2.	ZONISAMIDE 50mg capsules
dnq3.	ZONISAMIDE 100mg capsules
dnq4.	ZONEGRAN 25mg capsules
dnq5.	ZONEGRAN 50mg capsules
dnq6.	ZONEGRAN 100mg capsules
dnr..	RUFINAMIDE
dnr1.	INOVELON 100mg tablets
dnr2.	INOVELON 200mg tablets
dnr3.	INOVELON 400mg tablets
dnr4.	INOVELON 40mg/mL oral suspension
dnrw.	RUFINAMIDE 40mg/mL oral suspension
dnrx.	RUFINAMIDE 400mg tablets
dnry.	RUFINAMIDE 200mg tablets
dnrz.	RUFINAMIDE 100mg tablets
dns..	STIRIPENTOL
dns1.	DIACOMIT 250mg capsules
dns2.	DIACOMIT 500mg capsules
dns3.	DIACOMIT 250mg/sachet powder for oral suspension
dns4.	DIACOMIT 500mg/sachet powder for oral suspension
dnsw.	STIRIPENDOL 500mg/sachet powder for oral suspension
dnsx.	STIRIPENTOL 250mg/sachet powder for oral suspension
dnsy.	STIRIPENTOL 500mg capsules
dnsz.	STIRIPENTOL 250mg capsules
dnt..	LACOSAMIDE

For peer review only

dnt1.	VIMPAT 200mg/20mL solution for injection
dnt2.	VIMPAT 15mg/1mL sugar free liquid
dnt3.	VIMPAT 50mg tablets
dnt4.	VIMPAT 100mg tablets
dnt5.	VIMPAT 150mg tablets
dnt6.	VIMPAT 200mg tablets
dnt7.	LACOSAMIDE 200mg/20mL solution for injection
dnt8.	LACOSAMIDE 15mg/1mL sugar free liquid
dnt9.	LACOSAMIDE 50mg tablets
dntA.	LACOSAMIDE 100mg tablets
dntB.	LACOSAMIDE 150mg tablets
dntC.	LACOSAMIDE 200mg tablets
dntD.	VIMPAT 10mg/1mL syrup
dntE.	LACOSAMIDE 10mg/1mL sugar free liquid
dnu..	ESLICARBAZEPINE
dnu1.	ZEBINIX 800mg tablets
dnu2.	ESLICARBAZEPINE ACETATE 800mg tablets
dnv..	RETIGABINE
dnv1.	TROBALT 50mg tablets
dnv2.	TROBALT 100mg tablets
dnv3.	TROBALT 200mg tablets
dnv4.	TROBALT 300mg tablets
dnv5.	TROBALT 400mg tablets
dnv6.	TROBALT tablets initiation pack
dnv7.	RETIGABINE 50mg tablets
dnv8.	RETIGABINE 100mg tablets
dnv9.	RETIGABINE 200mg tablets
dnvA.	RETIGABINE 300mg tablets
dnvB.	RETIGABINE 400mg tablets
dnvC.	RETIGABINE 50mg+100mg tablets initiation pack
dnw..	PERAMPANEL
dnw1.	FYCOMPA 2mg tablets
dnw2.	FYCOMPA 4mg tablets
dnw3.	FYCOMPA 6mg tablets
dnw4.	FYCOMPA 8mg tablets
dnw5.	FYCOMPA 10mg tablets
dnw6.	FYCOMPA 12mg tablets
dnwu.	PERAMPANEL 12mg tablets
dnwv.	PERAMPANEL 10mg tablets
dnww.	PERAMPANEL 8mg tablets
dnwx.	PERAMPANEL 6mg tablets
dnwy.	PERAMPANEL 4mg tablets
dnwz.	PERAMPANEL 2mg tablets
do...	STATUS EPILEPTICUS DRUGS
do1..	DIAZEPAM [EPILEPSY USE]
do11.	DIAZEMULS [EP] 10mg/2mL injection
do12.	STESOLID [EP] 10mg/2mL injection
do13.	*STESOLID 20mg/4mL injection

For peer review only

do14.	STESOLID 5mg/2.5mL rectal solution
do15.	STESOLID 10mg/2.5mL rectal solution
do16.	VALIUM [EP] 10mg/2mL injection
do17.	VALIUM [EP] 20mg/4mL injection
do18.	DIAZEPAM 5mg/2.5mL RecTubes
do19.	DIAZEPAM 10mg/2.5mL RecTubes
do1A.	DIAZEPAM 2.5mg/1.25mL RecTubes
do1B.	*DIAZEPAM 20mg/5mL RecTubes
do1t.	DIAZEPAM 2.5mg/1.25mL rectal solution
do1u.	DIAZEPAM 20mg/5mL rectal solution
do1v.	DIAZEPAM 10mg/2mL emulsion injection
do1w.	DIAZEPAM 10mg/2mL injection
do1x.	DIAZEPAM 5mg/2.5mL rectal solution
do1y.	DIAZEPAM 10mg/2.5mL rectal solution
do1z.	*DIAZEPAM 20mg/4mL injection
do2..	CLONAZEPAM [STATUS EPILEPSY]
do21.	*RIVOTRIL 1mg/1mL injection
do21.	RIVOTRIL 1mg/1mL injection
do2z.	CLONAZEPAM 1mg/1mL injection
do2z.	*CLONAZEPAM 1mg/1mL injection
do3..	CLOMETHIAZOLE EDISYLATE [CENTRAL NERVOUS SYSTEM USE]
do31.	HEMINEVRIN [CNS] 8mg/mL intravenous infusion
do3z.	CLOMETHIAZOLE EDISYLATE 8mg/mL intravenous infusion
do4..	LORAZEPAM [EPILEPSY]
do41.	ATIVAN [EP] 4mg/mL injection
do5..	PARALDEHYDE
do51.	*PARALDEHYDE injection 5mL
do52.	*PARALDEHYDE injection 10mL
do6..	PHENYTOIN SODIUM [STATUS EPILEPSY]
do61.	EPANUTIN [EP] 250mg/5mL injection
do6z.	PHENYTOIN SODIUM 250mg/5mL injection

ALT_CODE	DESCRIPTION
G40	Epilepsy
G400	Localization-related (focal)(partial) idiopathic epilepsy and epileptic syndromes with seizures of localized onset
G401	Localization-related (focal)(partial) symptomatic epilepsy and epileptic syndromes with simple partial seizures
G402	Localization-related (focal)(partial) symptomatic epilepsy and epileptic syndromes with complex partial seizures
G403	Generalized idiopathic epilepsy and epileptic syndromes
G404	Other generalized epilepsy and epileptic syndromes
G405	Special epileptic syndromes
G406	Grand mal seizures, unspecified (with or without petit mal)
G407	Petit mal, unspecified, without grand mal seizures
G408	Other epilepsy
G409	Epilepsy, unspecified
G41	Status epilepticus

For peer review only

G410	Grand mal status epilepticus
G411	Petit mal status epilepticus
G412	Complex partial status epilepticus
G418	Other status epilepticus
G419	Status epilepticus, unspecified

For peer review only

The RECORD statement – checklist of items, extended from the STROBE statement, that should be reported in observational studies using routinely collected health data.

	Item No.	STROBE items	Location in manuscript where items are reported	RECORD items	Location in manuscript where items are reported
**Title and abstract**					
	1	(a) Indicate the study's design with a commonly used term in the title or the abstract (b) Provide in the abstract an informative and balanced summary of what was done and what was found		RECORD 1.1: The type of data used should be specified in the title or abstract. When possible, the name of the databases used should be included.<br><br>RECORD 1.2: If applicable, the geographic region and timeframe within which the study took place should be reported in the title or abstract.<br><br>RECORD 1.3: If linkage between databases was conducted for the study, this should be clearly stated in the title or abstract.	Page 1<br><br>Page 1<br><br>Page 1
**Introduction**					
Background rationale	2	Explain the scientific background and rationale for the investigation being reported			Page 3
Objectives	3	State specific objectives, including any prespecified hypotheses			Page 3
**Methods**					
Study Design	4	Present key elements of study design early in the paper			Pages 3-4
Setting	5	Describe the setting, locations, and relevant dates, including periods of recruitment, exposure, follow-up, and data collection			Page 4

For peer review

Participants	6	*(a) Cohort study* - Give the eligibility criteria, and the sources and methods of selection of participants. Describe methods of follow-up<br>*Case-control study* - Give the eligibility criteria, and the sources and methods of case ascertainment and control selection. Give the rationale for the choice of cases and controls<br>*Cross-sectional study* - Give the eligibility criteria, and the sources and methods of selection of participants<br><br>*(b) Cohort study* - For matched studies, give matching criteria and number of exposed and unexposed<br>*Case-control study* - For matched studies, give matching criteria and the number of controls per case		RECORD 6.1: The methods of study population selection (such as codes or algorithms used to identify subjects) should be listed in detail. If this is not possible, an explanation should be provided.<br><br>RECORD 6.2: Any validation studies of the codes or algorithms used to select the population should be referenced. If validation was conducted for this study and not published elsewhere, detailed methods and results should be provided.<br><br>RECORD 6.3: If the study involved linkage of databases, consider use of a flow diagram or other graphical display to demonstrate the data linkage process, including the number of individuals with linked data at each stage.	Page 4<br><br>See flow diagram (Figure 1)
Variables	7	Clearly define all outcomes, exposures, predictors, potential confounders, and effect modifiers. Give diagnostic criteria, if applicable.		RECORD 7.1: A complete list of codes and algorithms used to classify exposures, outcomes, confounders, and effect modifiers should be provided. If these cannot be reported, an explanation should be provided.	Page 4<br>Supplementary file 1
Data sources/ measurement	8	For each variable of interest, give sources of data and details of methods of assessment (measurement).<br>Describe comparability of assessment methods if there is more than one group			Page 4

For peer review only

Bias	9	Describe any efforts to address potential sources of bias			Adjusted for confounders Page 4
Study size	10	Explain how the study size was arrived at			See flow diagram (Figure 1)
Quantitative variables	11	Explain how quantitative variables were handled in the analyses. If applicable, describe which groupings were chosen, and why			Page 4
Statistical methods	12	(a) Describe all statistical methods, including those used to control for confounding<br>(b) Describe any methods used to examine subgroups and interactions<br>(c) Explain how missing data were addressed<br>(d) *Cohort study* - If applicable, explain how loss to follow-up was addressed<br>*Case-control study* - If applicable, explain how matching of cases and controls was addressed<br>*Cross-sectional study* - If applicable, describe analytical methods taking account of sampling strategy<br>(e) Describe any sensitivity analyses			Page 4
Data access and cleaning methods		..		RECORD 12.1: Authors should describe the extent to which the investigators had access to the database population used to create the study population.	Page 3

For peer review only

				RECORD 12.2: Authors should provide information on the data cleaning methods used in the study.	
Linkage		..		RECORD 12.3: State whether the study included person-level, institutional-level, or other data linkage across two or more databases. The methods of linkage and methods of linkage quality evaluation should be provided.	Page 4
**Results**					
Participants	13	(a) Report the numbers of individuals at each stage of the study (*e.g.*, numbers potentially eligible, examined for eligibility, confirmed eligible, included in the study, completing follow-up, and analysed)<br>(b) Give reasons for non-participation at each stage.<br>(c) Consider use of a flow diagram		RECORD 13.1: Describe in detail the selection of the persons included in the study (*i.e.,* study population selection) including filtering based on data quality, data availability and linkage. The selection of included persons can be described in the text and/or by means of the study flow diagram.	Flow diagram (Figure 1)
Descriptive data	14	(a) Give characteristics of study participants (*e.g.*, demographic, clinical, social) and information on exposures and potential confounders<br>(b) Indicate the number of participants with missing data for each variable of interest<br>(c) *Cohort study* - summarise follow-up time (*e.g.*, average and total amount)			Page 7
Outcome data	15	*Cohort study* - Report numbers of outcome events or summary measures over time<br>*Case-control study* - Report numbers in each exposure			Page 8-10

For peer review only

		category, or summary measures of exposure<br>*Cross-sectional study* - Report numbers of outcome events or summary measures			
Main results	16	(a) Give unadjusted estimates and, if applicable, confounder-adjusted estimates and their precision (e.g., 95% confidence interval). Make clear which confounders were adjusted for and why they were included<br>(b) Report category boundaries when continuous variables were categorized<br>(c) If relevant, consider translating estimates of relative risk into absolute risk for a meaningful time period			Page 9, Page 4 &5
Other analyses	17	Report other analyses done—e.g., analyses of subgroups and interactions, and sensitivity analyses			Page 5
**Discussion**					
Key results	18	Summarise key results with reference to study objectives			Page 5
Limitations	19	Discuss limitations of the study, taking into account sources of potential bias or imprecision. Discuss both direction and magnitude of any potential bias		RECORD 19.1: Discuss the implications of using data that were not created or collected to answer the specific research question(s). Include discussion of misclassification bias, unmeasured confounding, missing data, and changing eligibility over time, as they pertain to the study being reported.	Page 5
Interpretation	20	Give a cautious overall interpretation of results considering objectives,			Page 5

For peer review only - http://bmjopen.bmj.com/site/about/guidelines.xhtml

		limitations, multiplicity of analyses, results from similar studies, and other relevant evidence			
Generalisability	21	Discuss the generalisability (external validity) of the study results			Page 6
**Other Information**					
Funding	22	Give the source of funding and the role of the funders for the present study and, if applicable, for the original study on which the present article is based			Page 6
Accessibility of protocol, raw data, and programming code		..		RECORD 22.1: Authors should provide information on how to access any supplemental information such as the study protocol, raw data, or programming code.	Page 6

*Reference: Benchimol EI, Smeeth L, Guttmann A, Harron K, Moher D, Petersen I, Sørensen HT, von Elm E, Langan SM, the RECORD Working Committee. The REporting of studies Conducted using Observational Routinely-collected health Data (RECORD) Statement. *PLoS Medicine* 2015; in press.

*Checklist is protected under Creative Commons Attribution (CC BY) license.

For peer review only

BMJ Open

Health checks for adults with intellectual disability and association with survival rates – a linked electronic records matched cohort study in Wales, UK.

Journal:	*BMJ Open*
Manuscript ID	bmjopen-2021-049441.R2
Article Type:	Original research
Date Submitted by the Author:	29-Mar-2022
Complete List of Authors:	Kennedy, Natasha; Swansea University, National Centre for Population Health and Wellbeing<br>Kennedy, Jonathan; Swansea University; Swansea University<br>Kerr, Mike; Cardiff University School of Medicine<br>Dredge, Sam; Swansea University, School of Medicine<br>Brophy, Sinead; University of Swansea Institute of Health Research, School of Medicine
**Primary Subject Heading**:	Epidemiology
Secondary Subject Heading:	Public health, Health services research
Keywords:	Quality in health care < HEALTH SERVICES ADMINISTRATION & MANAGEMENT, EPIDEMIOLOGY, GENERAL MEDICINE (see Internal Medicine)

SCHOLARONE™
Manuscripts

I, the Submitting Author has the right to grant and does grant on behalf of all authors of the Work (as defined in the below author licence), an exclusive licence and/or a non-exclusive licence for contributions from authors who are: i) UK Crown employees; ii) where BMJ has agreed a CC-BY licence shall apply, and/or iii) in accordance with the terms applicable for US Federal Government officers or employees acting as part of their official duties; on a worldwide, perpetual, irrevocable, royalty-free basis to BMJ Publishing Group Ltd ("BMJ") its licensees and where the relevant Journal is co-owned by BMJ to the co-owners of the Journal, to publish the Work in this journal and any other BMJ products and to exploit all rights, as set out in our licence.

The Submitting Author accepts and understands that any supply made under these terms is made by BMJ to the Submitting Author unless you are acting as an employee on behalf of your employer or a postgraduate student of an affiliated institution which is paying any applicable article publishing charge ("APC") for Open Access articles. Where the Submitting Author wishes to make the Work available on an Open Access basis (and intends to pay the relevant APC), the terms of reuse of such Open Access shall be governed by a Creative Commons licence – details of these licences and which Creative Commons licence will apply to this Work are set out in our licence referred to above.

Other than as permitted in any relevant BMJ Author's Self Archiving Policies, I confirm this Work has not been accepted for publication elsewhere, is not being considered for publication elsewhere and does not duplicate material already published. I confirm all authors consent to publication of this Work and authorise the granting of this licence.

For peer review only

Health checks for adults with intellectual disability and association with survival rates – a linked electronic records matched cohort study in Wales, UK.

Natasha Kennedy PhD[a], Jonathan Kennedy EngD[a,b,] , Michael Kerr FRCPSYCH[c], Sam Dredge PGCE[a], Sinead Brophy PhD[a]

a. Swansea University Medical School, Data Science Building, Swansea University, Swansea, United Kingdom
b. Health Data Research UK Wales and Northern Ireland, Swansea University Medical School, Data Science Building, Swansea University, Swansea, United Kingdom.
c. Institute of Psychological Medicine and Clinical Neurosciences, Cardiff University School of Medicine, Cardiff, UK

Corresponding author: Professor Sinead Brophy, National Centre for Population Health and Wellbeing Research, Data Science Building, School of Medicine, Swansea University. SA2 8PP. UK. Tel 01792602058, email: s.brophy@swansea.ac.uk.

For peer review only

Abstract

Objective

To examine if mortality rates are lower in people with intellectual disability who have had a health check compared to those who have not had health checks.

Setting

General practice records of 26,954 people with an intellectual disability in Wales between 2005-2017, of which 7,650 (28.4%) with a health check were matched 1:2 with those without a health check.

Primary outcome measure:

ONS mortality data; a Cox Regression was utilised to examine time to death adjusted for co-morbidities and gender.

Results

Patients who had a health check were stratified by those who (a) had a confirmed health check i.e. Read Code for a health check (n = 7,650 (28.4 %)), and (b) had no evidence of receiving a health check in their medical record. Patients with a health check were matched for age at time of health check with two people who did not have a health check. The health check was associated with improved survival for those with autism or Down's Syndrome (Hazard Ratio (HR): 0.58 (95 % CI: 0.37 to 0.91) and HR: 0.76 (95 % CI: 0.64 to 0.91), respectively). There was no evidence of improved survival for those diagnosed with diabetes or cancer. The people who had a health check were more likely to be older, have epilepsy, and less likely to have autism or Down's Syndrome.

Conclusions

Health checks are likely to influence survival if started before a person is diagnosed with a chronic condition, especially for people with autism or Down's Syndrome.

For peer review only

Article summary: Strengths and limitations of the study:

- These findings are based on a total population cohort in one country of people with intellectual disabilities.
- Patients who have had a health check with no record in their GP data will be misclassified, consequently, the differences observed will likely be higher than those reported in this study.
- Patients who live longer are more likely to have a health check (survival bias); to account for this the cohort has been paired with age matched controls.
- However, fewer controls were available to match for older people who had a health check, this could have influenced the findings for comparisons at older ages.

peer review only

For peer review only - http://bmjopen.bmj.com/site/about/guidelines.xhtml

Introduction

People with an intellectual disability experience more health conditions such as; epilepsy [1], autism, and dental problems [2,3]. In addition, prior research [4] observed that people with an intellectual disability are at a higher risk of leading sedentary lives and becoming overweight; subsequently developing diabetes, cardiovascular disease, and respiratory disease [5]. Furthermore, this research has indicated that they are more likely to be exposed to poverty, poor housing conditions, unemployment, and other social determinates of poor health [6]. Finally, communication and ability to act on health promotion information means access to health care provision will be reduced for people with intellectual disability. Inequalities in health are apparent for people with intellectual disabilities [1]; health checks have been recommended as one component of international health policy to address the poorer health of people with intellectual disabilities [7]. Annual health checks for people with an intellectual disability and being present on the social services register were introduced into Wales in 2006 and in England in 2007. The Cardiff/Welsh annual health check for adults is aimed at early detection and treatment [8]. The 2010 Improving Health and Lives – The Learning Disabilities Public Health Observatory (IHAL) review demonstrated that the health check improves detection of unmet, potentially treatable health needs [9]. Previous studies [7] further identified that health checks lead to detection of unmet needs and targeted actions to address health needs. Additionally, a recent study indicated that General Practitioners (GP) practices with Enhanced Service (e.g. incentivised in offering the health check) had more health action plans and secondary care referrals [10]. The health check has been observed to improve detection of less serious health conditions such as ear wax obscuring one or both eardrums, and dental problems which will greatly influence a person's quality of life [7]. They have been found to improve detection of serious conditions such as cancer, and may improve the health promotion [7,8,11]. Conversely, it is not known if this translates into health gain. Few studies have evaluated the extent to which providing health checks leads to long term health benefits. In fact, a new evaluation of the impact of the Directed Enhanced Services (DES) in England found no significant difference between health check and controls (no health check) in terms of intermediate outcomes such as control of blood pressure [12]. In addition, annual health checks where not found to reduce emergency admissions, but they did appear to reduce preventable emergency admissions (e.g. those for diabetes or COPD [12]). The lack of evidence of the long-term benefits of health checks was a justification for practices not offering them, as without this evidence the time taken to organise and undertake assessments is a substantial barrier for GPs [13]. This study specifically examines if health checks are associated with better survival and lower rates of mortality compared to those who have no health check.

Methods

Study Design

The Secure Anonymised Information Linkage (SAIL) databank is a data repository which allows person-based data linkage across datasets. This databank includes Welsh GP data, hospital in- and out-patient records, as well as mortality data collected by the Office of National Statistics (ONS). SAIL comprises of over a billion anonymised records. It utilises a split-file approach to ensure anonymisation, overcome

For peer review only - http://bmjopen.bmj.com/site/about/guidelines.xhtml

issues of confidentiality, and disclosure in health-related data warehousing. Demographic data is sent to a partner organisation, NHS Wales Informatics Service, where identifiable information is removed. Clinical data are sent directly to the SAIL databank where an individual is assigned an encrypted Anonymised Linking Field (ALF). The ALF is utilised to link anonymised individuals across datasets, facilitating longitudinal analysis of an individual's journey through multiple health, education, and social datasets. Data collected by GP's are captured via Read Codes (5-digit codes related to diagnosis, medication, and process of care codes). Hospital in- and out-patient data are collected in the Patient Episode Database for Wales; this employs the International Classification of Diseases (ICD-10) clinical coding system to record clinical information regarding patients' hospital admissions, discharges, diagnoses, and operations. The ONS Mortality dataset encompasses demographic data, place of death, and underlying cause of death (also ICD-10). Anonymised routine data analysis is not subject to ethical committee approval; it is subject to Information Governance approval. This project has been approved by the IGRP (Information Governance Review Panel) which provides independent approval that the research is within the public interest and includes representatives from the National Research Ethics Service, British Medical Association, and the public.

Patient and Public Involvement: This work was discussed with the Learning Disability Ministerial Advisory Group for Wales which includes people with intellectual disabilities, and those caring for those with intellectual disabilities. Those with an intellectual disability were not involved in the design of the study or conduct of the study.

All data was selected from 2005 – 2017, patients were flagged for intellectual disability (See Supplementary file 1 and definition provided in [14]). Patients entered the study when they became eligible for a health check; at age 18 or in 2006 whichever is later. The eligible patients were stratified by (a) ever had a confirmed health check (Read Code for a health check recorded) at any time in the patients record, and (b) had no evidence of receiving a health check in their medical record.

Procedures

Statistical Analysis

STATA 15 was utilised for all analysis. Descriptive statistics for each category were generated. Patients with a health check (index case) were matched for age (+/- 5 years) at health check with 2 patients who did not have a health check, thus forming a matched cohort (Figure 1). Cox regression was employed to examine time to mortality, from date of study entry. Analysis was adjusted for gender and comorbidities (autism with co-occurring intellectual disabilities, Down's Syndrome, diabetes, epilepsy, and cancer) as these were identified as confounding variables; the cluster command in STATA was utilised to account for matching design. The incidence of death (deaths over number of person years follow up) are presented for each co-morbidity separately. However, the comparison patient was not matched on co-morbidity in this sub-analysis. People were censored if they moved out of Wales or were lost to follow up. This dataset included everyone with an intellectual disability over the age of 18 since 2006.

For peer review only - http://bmjopen.bmj.com/site/about/guidelines.xhtml

Results

There were 26,954 people with an intellectual disability between 2006 and 2017 in Wales (11-year period), of these 7650 (28.4 %) have a GP record of ever having had a health check (see Table 1). Consequently, this indicates that 71.6 % of people with intellectual disabilities have no record of having a health check in their electronic medical records (e.g., 19304/26954). The proportion of people who have a health check by year is between 1.69 % (in 2006 when health checks began) and 12.4 % in 2015 (see Figure 2); subsequently, each year approximately 87.6 % (e.g., if at most 12.4% have a health check) of eligible people do not have a health check.

Patients who received a health check were more likely to be older (see Table 1); the average cohort age was 40.5 years (health check) compared to 31.3 years (No health check), resulting in a difference of 9.2 years (95 % CI: 8.8 to 9.7). Additionally, the health check cohort were more likely to have epilepsy; 39.3 % in comparison to 28.7 % for the control cohort (difference 10.6 % (95 % CI: 9.3 % to 11.9 %)). Those with autism & ID were less likely to undergo a health check (15 % of those with autism have had a health check). There was no difference in socio-economic level of those who had a health check compared to those who did not (see Table 1).

Those receiving a health check were on average older than those not having health checks, in order to adjust for this survival bias, each index case was matched for the age (+/- 5 years) at health check with two patients who had no record of undergoing a health check (see Table 2). There was a trend for those who had a health check to have a lower rate of death compared to their matched comparisons (2.5 per 1000 per year fewer deaths, Figure 3). Results also indicated that those with autism (HR: 0.58 (95 % CI: 0.37 to 0.91)) and those with Down's Syndrome (HR: 0.76 (95 % CI: 0.64 to 0.91)) attained better survival rates when receiving a health check (Table 2). Conversely, analysis indicated no significant increase in survival rate if a patient had diabetes, epilepsy, or cancer. However, in nearly every case the diagnosis of these conditions happened before the date of the health check.

Screening for cancer was very low in both the health check and no health check groups (7.6 % and 5.3 % respectively); there was no evidence of lower death rates in those who had cancer and received a health check compared to those who did not have a health check. There was a slight trend to higher rate of death in those who have a health check and have cancer. However, it should be noted that the comparison matches would have been unlikely to also have cancer.

Discussion:

This study examined the medical records of 26,954 people with an intellectual disability and observed that having a health check was associated with reduced mortality for people with autism and those with Down's syndrome. Minimal evidence of reduced mortality rates was observed for those diagnosed with conditions such as diabetes or epilepsy; furthermore, no evidence was obtained to indicate that health check improved outcomes for people diagnosed with cancer.

For peer review only - http://bmjopen.bmj.com/site/about/guidelines.xhtml

This study only examined those who had a record of having at least one health check recorded in their medical notes with a READ code. If a person received a health check but this was not coded or recorded as such, these individuals would have been misclassified and categorised into the no health check group. Previous studies indicated that the number of people having health checks was 41 % in 2008 in Wales [15]; this number was established on GP submissions to the Community, Primary Care, and the Health Services Policy Division of the Welsh Assembly Government. However, this study observed that 28.4 % of people with an intellectual disability had ever received a health check coded in their medical records. This difference signifies that approximately 69 % of those who might have undergone a health check had it coded in their notes, thus, the health check cohort in this study could be missing a third of the people who received a health check. Consequently, the true difference between those who have health checks and those who do not may be larger than this study has detected due to a misclassification error. In addition, this study exclusively followed individuals who are registered with their GP and had a code in their medical record identifying that they had an intellectual disability. This study could not observe the level of intellectual disability. However, the results indicated that more people who received the health check also possessed a diagnosis of epilepsy. This would suggest that those with a more severe intellectual disability are most likely to be given a health check; thus are likely to have had a higher mortality rate. This assumption is supported by prior research [12] which found those who have health checks are older, have higher levels of support needs, and are more likely to be in communal living. There were fewer controls available in the older age groups, and this means the controls were often younger than the case (within 5 years), this could influence the finding for comparisons at older ages.

The number of eligible people having a health check recorded by the GP practice each year was approximately 10 - 12 %. This indicates that many people may have received one health check but not undergone the health check annually, and that the majority of people with an intellectual disability never have a health check. The study identified a lower than expected uptake of health checks in the adult population in Wales, with 71.6 % having no record of a health check. Whilst the methodology cannot identify the reason for this, this indication from the data is concerning. It appears likely that adults are not being offered health checks; either as they are not recognised as being eligible for a health check, or there is a barrier to accessing checks when offered. Further exploration of this disparity in delivery is required.

Health checks have been indicated to be valued by carers and people with an intellectual disability; they have been revealed to not increase costs, and detect health requirements earlier [1,12]. This study demonstrates that they are also associated with a long-term gain in improving survival for people with an intellectual disability, especially for those with autism or Down’s syndrome. The findings of the study suggest that health checks may be most beneficial for prevention of morbidities; it did not find evidence that a health check improves survival when a person has existing health conditions, such as diabetes or cancer. It is likely that once diagnosed, those with ID have the same care for their diabetes/epilepsy/cancer whether receiving health checks or not. Previous research has demonstrated that emergency admission for these conditions is reduced [12] when a person undergoes health checks.

For peer review only - http://bmjopen.bmj.com/site/about/guidelines.xhtml

The health check may improve prevention and therefore be associated with improved survival for those without existing co-morbidities. There is the argument that the health check itself is not associated with improved survival; instead, this relationship is confounded by the health check being provided by more engaged GP practices [8], more engaged family members, those with paid carers, and better overall care. However, the lack of association of the health check with enhanced survival when diagnosed with a chronic condition, especially those with cancer, and a lack of association with socioeconomic deprivation found in this study, would refute this argument of confounding. Barriers and facilitators for health checks for cardiometabolic disease was reported to be more associated with the individual rather than home environment/socio economics/engagement [16]. Prior research concluded that factors such as feeling healthy, and practical issues such as the type of invitation, as well as availability of an easy appointment acted as barriers; consequently, an argument that engaged families rather than a health check leads to benefit does not appear to be supported by the existing evidence [16].

Conclusions

Health checks are associated with a trend to improved survival for people with intellectual disabilities, especially for people with autism and co-occurring intellectual disabilities and Down's Syndrome. Increasing the uptake of health checks could help with prevention of morbidities and improve survival for people who do not already have chronic disease. However, there was limited evidence from this work that survival is improved when a person has existing morbidities. This study indicates benefits associated with health checks, in terms of lower rates of mortality for those with autism or Down's Syndrome.

Contributorship statement:

The concept of the study and design was by MK, SB. The dataset was prepared by JK and analysis undertaken by NK with supervision from SB and MK. The findings and interpretation was after discussion with stakeholders in a consultation which was undertaken by SD. All authors contributed to drafting the paper and all have given approval of the final submission.

Competing interests

None to declare.

Funding

This work was supported by Health and Care Research Wales (National Centre for Population Health and Well-being), and Health Data Research UK (NIWA1) which receives its funding from HDR UK Ltd. HDR UK Ltd is funded by the UK Medical Research Council, Engineering and Physical Sciences Research Council, Economic and Social Research Council, Department of Health and Social Care (England), Chief Scientist Office of the Scottish Government Health and Social Care Directorates, Health and Social Care Research and Development Division (Welsh Government), Public Health Agency (Northern Ireland), British Heart Foundation (BHF), and the Wellcome Trust.

For peer review only - http://bmjopen.bmj.com/site/about/guidelines.xhtml

For peer review only

Data sharing statement

The dataset used in this article is available via the Secure Anonymised Information Linkage (SAIL) databank, which is part of the national ehealth records infrastructure for Wales. The data is accessible by applying to SAIL. For further information on the SAIL databank and enquiries in how to access the data, please visit the SAIL website (http://www.saildatabank.com).

Acknowledgements

This study uses anonymised data provided by patients, collected by the NHS as part of their care and support and held in the Secure Anonymised Information Linkage (SAIL) Databank. We would like to acknowledge all data providers that make anonymised data available for research. All data used can be accessed by contacting the SAIL gateway (https://saildatabank.com/).

For peer review only

Figures:

Figure 1: Flow diagram of participant inclusion.

Figure 2: Number of health checks per year.

Figure 3: Survival in those who have a health check compared to no health check group (2:1 matched (for age +/- 5 years).

For peer review only - http://bmjopen.bmj.com/site/about/guidelines.xhtml

Table 1: Demographics of those who have a health check compared to those with no health check (from time eligible for health check)

	No health check	Health check	Difference (95% CI)
**Demographic Characteristics**			
**Total (n)**	19304 (71.6 %)	7650 (28.4 %)	
**Average number of years of follow-up**	8.37	3.85	
**Mean age (sdev)**	31.3 (17.7)	40.5 (16.4)	-9.2 (-9.7 to -8.8)
**Age at baseline**			
**18 to 50 years old**	16070 (83.2%)	5539 (72.4%)	10.8% (9.7 to 12.0)
**More than 50 years old**	3234 (16.8 %)	2111 (27.6 %)	-10.8% (-12.0 to -9.7)
**Male**	11655 (60.4 %)	4401 (57.5 %)	2.8% (1.5 to 4.2)
**Autism**	6290 (32.6 %)	1180 (15.4 %)	17.2% (16.1 to 18.2)
**Down's Syndrome**	3364 (17.4 %)	915 (12.0 %)	5.5 % (4.6 to 6.4)
**Diabetes**	2332 (12.1 %)	1030 (13.5 %)	-1.4% (-2.3 to -0.5)
**Epilepsy**	5536 (28.7 %)	3004 (39.3 %)	-10.6% (-11.9 to -9.3)
**Cancer Screening**	1030 (5.3 %)	582 (7.6 %)	-2.3 % (-3.0 to -1.6)
**Cancer**	3095 (16.0 %)	1040 (13.6 %)	2.4% (1.5 to 3.4)
**Died**	2527 (13.1 %)	751 (9.8 %)	3.3% (2.4 to 4.0)
**Townsend Scores**			
**1**	2515 (13.0 %)	1087 (14.2 %)	-1.2 (-2.1 to -0.3)
**2**	2978 (15.4%)	1137 (14.9%)	0.6 (-0.4 to 1.5)
**3**	3317 (17.2%)	1535 (20.1 %)	-2.9 (-3.9 to -1.9)
**4**	3778 (19.6%)	1706 (22.3 %)	-2.7 (-3.8 to -1.7)
**5 (most deprived)**	4468 (23.1%)	1829 (23.9 %)	-0.8(-1.9 to 0.4)
**N/A**	2248 (11.6%)	356 (4.7%)	7.0 (6.3 to 7.6)

For peer review only - http://bmjopen.bmj.com/site/about/guidelines.xhtml

For peer review only

Table 2: Survival in those who have a health check compared to no health check group (2:1 matched (for age +/- 5 years).

	No health check (2 matches) N=12152	Health check (Index case) N=7650
Average Number of Years of follow-up	10.39	5.74
Survival (time to death)		
Total (deaths per 1000/year)	19.6 (95 % CI: 18.8 – 20.3)	17.1 (95 % CI: 15.9 – 18.4)
Hazard ratio adjusted for co-morbidities and gender	1	0.94 (95 % CI: 0.87 – 1.01)
Males (deaths per 1000/year)	19.0 (95 % CI: 18.0 – 20.1)	16.9 (95 % CI: 15.3 – 18.6)
Hazard Ratio adjusted for co-morbidities and gender	1	0.93 (95 % CI: 0.83 – 1.03)
Females (deaths per 1000/year)	20.2 (95 % CI: 19.1 – 21.4)	17.4 (95 % CI: 15.6 – 19.4)
Hazard Ratio adjusted for co-morbidities and gender	1	0.94 (95 % CI: 0.84 - 1.07)
For those with Autism	8.4 (95 % CI: 7.4 – 9.6)	3.55 (95 % CI: 2.3 – 5.4)
Crude Hazard Ratio	1	**0.58 (95 % CI: 0.37 - 0.91)**
Adjusted for co-morbidities and gender	1	**0.56 (95 % CI: 0.36 – 0.89)**

For peer review only - http://bmjopen.bmj.com/site/about/guidelines.xhtml

For those with Down's Syndrome	35.6 (95 % CI: 32.8- 38.6)	27.2 (95 % CI: 23.2 - 31.9)
Crude Hazard Ratio	1	**0.76 (95 % CI: 0.64 – 0.91)**
Adjusted for co-morbidities and gender	1	**0.80 (95 % CI: 0.67 – 0.96)**
For those with Diabetes	31.7 (95 % CI: 29.4 – 34.2)	27.6 (95 % CI: 23.8 – 32.2)
Crude Hazard Ratio	1	0.97 (95 % CI: 0.81 – 1.15)
Adjusted for co-morbidities and gender	1	0.98 (95 % CI: 0.82 – 1.16)
For those with Epilepsy	24.3 (95 % CI: 22.9 – 25.7)	21.9 (95 % CI: 19.8 – 24.2)
Crude Hazard Ratio	1	1.03 (95 % CI: 0.91 - 1.16)
Adjusted for co-morbidities and gender	1	1.07 (95 % CI: 0.95 -1.20)
For those with Cancer Screening	25.4 (95 % CI: 22.6 – 28.6)	21.2 (95 % CI: 17.1 – 26.3)
Crude Hazard Ratio	1	1.0 (95 % CI: 0.77 – 1.30)
Adjusted for co-morbidities and gender	1	1.08 (95 % CI: 0.83 -1.40)
For those with Cancer	36.6 (95 % CI: 34.3 – 39.0)	38.5 (95 % CI: 33.7 – 43.9)
Crude Hazard Ratio	1	1.18 (95 % CI: 1.0 – 1.4)
Adjusted for co-morbidities and gender	1	1.18 (95 % CI: 1.0 - 1.4)

For peer review only - http://bmjopen.bmj.com/site/about/guidelines.xhtml

References:

[1] Kinnear D, Morrison J, Allan L, Henderson A, Smiley E, Cooper SA. Prevalence of physical conditions and multimorbidity in a cohort of adults with intellectual disabilities with and without Down syndrome: cross-sectional study. BMJ Open. 2018 Feb 5;8(2):e018292. doi: 10.1136/bmjopen-2017-018292. PMID: 29431619; PMCID: PMC5829598.

[2] van Schrojenstein Lantman-de Valk HM, Walsh PN. Managing health problems in people with intellectual disabilities. BMJ. 2008 Dec 8;337:a2507. doi: 10.1136/bmj.a2507. PMID: 19064601.

[3] Ward LM, Cooper SA, Hughes-McCormack L, Macpherson L, Kinnear D. Oral health of adults with intellectual disabilities: a systematic review. J Intellect Disabil Res. 2019 Nov;63(11):1359-1378. doi: 10.1111/jir.12632. Epub 2019 May 23. PMID: 31119825.

[4] Oeseburg, B. , Dijkstra, G. J., Groothoff, J. W., Reijneveld, S. A. , & Jansen, D. E.M.C. (2011). Prevalence of Chronic Health Conditions in Children With Intellectual Disability: A Systematic Literature Review. *Intellectual and Developmental Disabilities*, *49(2),*59-85. https://doi.org/10.1352/1934-9556-49.2.59

[5] Lhatoo SD, Sander JW. The epidemiology of epilepsy and learning disability. Epilepsia. 2001;42 Suppl 1:6-9; discussion 19-20. doi: 10.1046/j.1528-1157.2001.00502.x. PMID: 11422344.

[6] Turner S. Health Inequalities and people with learning disabilities in the UK: 2011. In: Improving Health and Lives. Learning Disabilities Oberatory;2021.

[7] Robertson J, Hatton C, Emerson E, Baines S. The impact of health checks for people with intellectual disabilities: an updated systematic review of evidence. Res Dev Disabil. 2014 Oct;35(10):2450-62. doi: 10.1016/j.ridd.2014.06.007. Epub 2014 Jun 28. PMID: 24984052.

[8] Lowe, K., Felce, D., Baxter, H., Dunstan, F., Houston, H., Jones, G., Grey, J., Felce, J., & Kerr, M. (2008). The Impact of Checking the Health of Adults with Intellectual Disabilities on Primary Care Consultation Rates, Health Promotion and Contact with Specialists. Journal of Applied Research in Intellectual Disabilities, 21(6), 597 - 602. https://doi.org/10.1111/j.1468-3148.2008.00432.x

[9] Robertson, J., Roberts, H., & Emerson, E. (2010). Health checks for people with learning disabilities: A systematic review of evidence. Improving Health & Lives: Learning Disabilities Observatory.

[10] Buszewicz M, Welch C, Horsfall L, Nazareth I, Osborn D, Hassiotis A, Glover G, Chauhan U, Hoghton M, Cooper SA, Moulster G, Hithersay R, Hunter R, Heslop P, Courtenay K, Strydom A. Assessment of an incentivised scheme to provide annual health checks in primary care for adults with intellectual disability: a longitudinal cohort study. Lancet Psychiatry. 2014 Dec;1(7):522-30. doi: 10.1016/S2215-0366(14)00079-0. Epub 2014 Dec 3. PMID: 26361311.

[11] Chauhan U, Reeve J, Kontopantelis E, Hinder S, Nelson P, Doran T. Impact of the English directly enhanced service (DES) for learning disability. In. Manchester: Manchester University; 2012.

[12] Carey, I. M., Hosking, F. J., Harris, T., DeWilde, S., Beighton, C., Shah, S. M., & Cook, D. G. (2017). Do health checks for adults with intellectual disability reduce emergency hospital admissions? Evaluation of a natural experiment. Journal of Epidemiology and Community Health, 71(1), 52-58. https://doi.org/10.1136/jech-2016-207557

For peer review only - http://bmjopen.bmj.com/site/about/guidelines.xhtml

[13] Walmsley J. If annual health checks for people with learning disabilities can address failings identified in their health care, why are more GPs not doing them? BMJ 2009;338:b1261.

[14] Brophy S, Kennedy J, Fernandez-Gutierrez F, John A, Potter R, Linehan C, Kerr M. Characteristics of Children Prescribed Antipsychotics: Analysis of Routinely Collected Data. J Child Adolesc Psychopharmacol. 2018 Apr;28(3):180-191. Doi: 10.1089/cap.2017.0003. Epub 2018 Feb 27. PMID: 29486137; PMCID: PMC5905863.

[15] Perry J, Kerr M, Felce D, Bartley S, Tomlinson J. Monitoring the public health impact of health checks for adults with learning disability in Wales. In.: Public Health Wales.; 2010

[16] de Waard AM, Wändell PE, Holzmann MJ, Korevaar JC, Hollander M, Gornitzki C, de Wit NJ, Schellevis FG, Lionis C, Søndergaard J, Seifert B, Carlsson AC; SPIMEU Research Group. Barriers and facilitators to participation in a health check for cardiometabolic diseases in primary care: A systematic review. Eur J Prev Cardiol. 2018 Aug;25(12):1326-1340. doi: 10.1177/2047487318780751. Epub 2018 Jun 19. PMID: 29916723; PMCID: PMC6097107.

only

For peer review only - http://bmjopen.bmj.com/site/about/guidelines.xhtml

Figure 1: Flow diagram of participant inclusion.

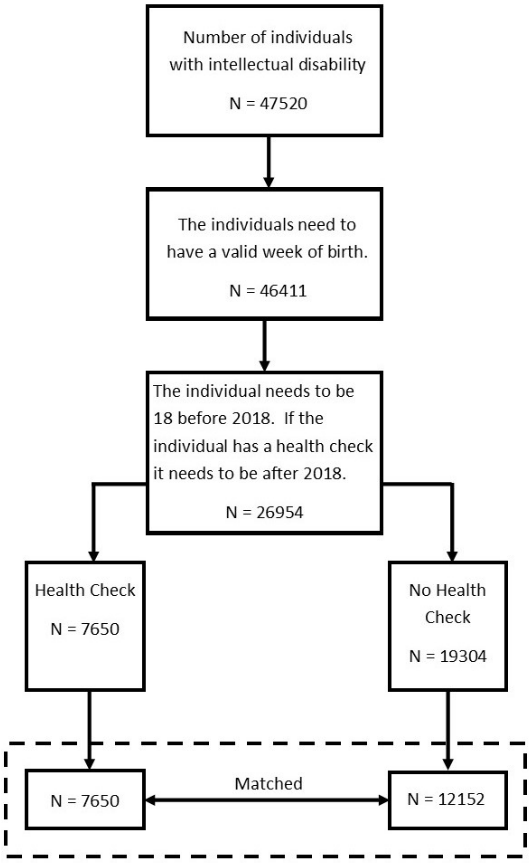

Figure 1: Flow diagram of participant inclusion

134x224mm (150 x 150 DPI)

Figure 2: Number of health checks per year.

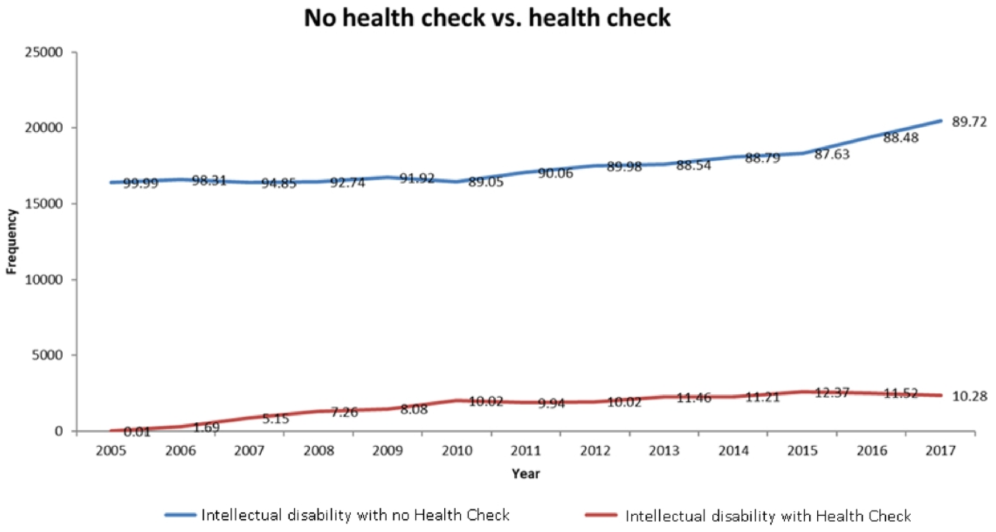

Number of health checks per year

190x124mm (300 x 300 DPI)

Figure 3: Survival in those who have a health check compared to no health check group (2:1 matched (for age +/- 5 years).

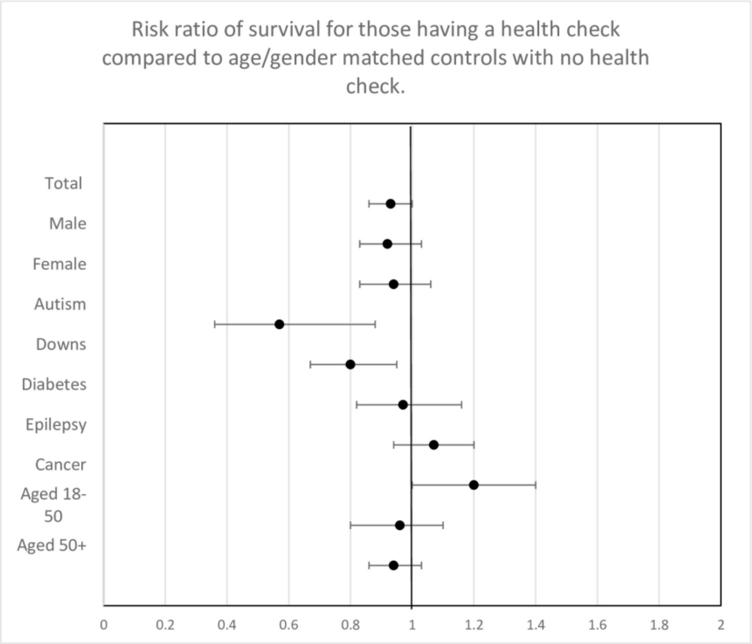

1

Survival in those who have a health check compared to no health check group (2:1 matched (for age +/- 5 years).

143x222mm (300 x 300 DPI)

Health Assessment

READ_CODE	READ_DESC
9HB5.	Learning disabilities annual health assessment

Downs (GP)

READ_CODE	READ_DESC
PJ0..	Down's syndrome - trisomy 21
PJ00.	Trisomy 21, meiotic nondisjunction
PJ0z.	Down's syndrome NOS
PJ02.	Trisomy 21, translocation
PJ01.	Trisomy 21, mosaicism

Hospital

ALT_CODE	DESCRIPTION
Q900	Trisomy 21, meiotic nondisjunction
Q901	Trisomy 21, mosaicism (mitotic nondisjunction)
Q902	Trisomy 21, translocation
Q909	Down syndrome, unspecified
Q909	Down's syndrome, unspecified

ALT_CODE	DESCRIPTION
Q90	Down syndrome

Autism (GP)

READ_CODE	READ_DESC
1J9..	Suspected autism
E1401	Residual infantile autism
E1400	Active infantile autism
E140.	Infantile autism
Eu841	[X]Atypical autism
E140z	Infantile autism NOS
Eu840	[X]Childhood autism

Hospital

ALT_CODE	DESCRIPTION
F840	Childhood autism
F841	Atypical autism

For peer review only

Learning disability (GP)

READ_CODE	READ_DESC
1J9..	Suspected autism
69DB.	Learning disability health examination
8Ce6.	Preferred place of care - learning disability unit
8H4f.	Referral to learning disabilities psychiatrist
8HHP.	Referral to learning disability team
8Hg2.	Discharge from learning disability team
918e.	On learning disability register
94Z9.	Preferred place of death: learning disability unit
9HB..	Learning disabilities administration status
9HB0.	Learning disabilities health action plan declined
9HB1.	Learning disabilities health action plan offered
9HB2.	Learning disabilities health action plan reviewed
9HB3.	Learning disabilities health assessment
9HB4.	Learning disabilities health action plan completed
9HB5.	Learning disabilities annual health assessment
9HB6.	Learning disabilities annual health assessment declined
9HB7.	Did not attend learning disabilities annual health assessment
9N0y.	Seen in learning disabilities clinic
9hL..	Exception reporting: learning disability quality indicators
9mA..	Learning disability annual health check invitation
9mA0.	Learning disability annual health check verbal invitation
9mA1.	Learning disability annual health check telephone invitation
9mA2.	Learning disability annual health check letter invitation
9mA20	Learning disability annual health check invitation first letter
9mA21	Learning disability annual health check invitation second letter
9mA22	Learning disability annual health check invitation third letter
E140.	Infantile autism
E1400	Active infantile autism
E1401	Residual infantile autism
E140z	Infantile autism NOS
E3...	Mental retardation
E30..	Mild mental retardation, IQ in range 50-70
E31..	Other specified mental retardation
E310.	Moderate mental retardation, IQ in range 35-49
E311.	Severe mental retardation, IQ in range 20-34
E312.	Profound mental retardation with IQ less than 20
E31z.	Other specified mental retardation NOS
E3y..	Other specified mental retardation
E3z..	Mental retardation NOS
Eu7..	[X]Mental retardation
Eu70.	[X]Mild mental retardation
Eu700	[X]Mild mental retardation with the statement of no, or minimal, impairment of behaviour
Eu701	[X]Mild mental retardation, significant impairment of behaviour requiring attention or treatment

For peer review only

Eu70y	[X]Mild mental retardation, other impairments of behaviour
Eu70z	[X]Mild mental retardation without mention of impairment of behaviour
Eu71.	[X]Moderate mental retardation
Eu710	[X]Moderate mental retardation with the statement of no, or minimal, impairment of behaviour
Eu711	[X]Moderate mental retardation, significant impairment of behaviour requiring attention or treatment
Eu71y	[X]Mod retard oth behav impair
Eu71z	[X]Moderate mental retardation without mention of impairment of behaviour
Eu72.	[X]Severe mental retardation
Eu720	[X]Severe mental retardation with the statement of no, or minimal, impairment of behaviour
Eu721	[X]Severe mental retardation, significant impairment of behaviour requiring attention or treatment
Eu72y	[X]Severe mental retardation, other impairments of behaviour
Eu72z	[X]Severe mental retardation without mention of impairment of behaviour
Eu73.	[X]Profound mental retardation
Eu730	[X]Profound mental retardation with the statement of no, or minimal, impairment of behaviour
Eu731	[X]Profound mental retardation, significant impairment of behaviour requiring attention or treatment
Eu73y	[X]Profound mental retardation, other impairments of behaviour
Eu73z	[X]Profound mental retardation without mention of impairment of behaviour
Eu7y.	[X]Other mental retardation
Eu7y0	[X]Other mental retardation with the statement of no, or minimal, impairment of behaviour
Eu7y1	[X]Other mental retardation, significant impairment of behaviour requiring attention or treatment
Eu7yy	[X]Other mental retardation, other impairments of behaviour
Eu7yz	[X]Other mental retardation without mention of impairment of behaviour
Eu7z.	[X]Unspecified mental retardation
Eu7z0	[X]Unspecified mental retardation with the statement of no, or minimal, impairment of behaviour
Eu7z1	[X]Unspecified mental retardation, significant impairment of behaviour requiring attention or treatment
Eu7zy	[X]Unspecified mental retardation, other impairments of behaviour
Eu7zz	[X]Unspecified mental retardation without mention of impairment of behaviour
Eu814	[X]Moderate learning disability
Eu815	[X]Severe learning disability
Eu816	[X]Mild learning disability
Eu817	[X]Profound learning disability
Eu818	[X]Specific learning disability
Eu840	[X]Childhood autism
Eu841	[X]Atypical autism
PJ0..	Down's syndrome - trisomy 21
PJ00.	Trisomy 21, meiotic nondisjunction
PJ01.	Trisomy 21, mosaicism
PJ02.	Trisomy 21, translocation
PJ0z.	Down's syndrome NOS

For peer review only

Hospital

ALT_CODE	DESCRIPTION
F79X	Unspecified mental retardation
Q18.9	Trisomy 21, meiotic nondisjunction
F84.0	Pervasive developmental disorders
Q90	Down syndrome
Q900	Trisomy 21, meiotic nondisjunction
Q901	Trisomy 21, mosaicism (mitotic nondisjunction)
Q902	Trisomy 21, translocation
Q909	Down syndrome, unspecified

Diabetes (GP)

READ_CODE	READ_DESC
C10..	Diabetes mellitus
C100.	Diabetes mellitus with no mention of complication
C1000	Diabetes mellitus, juvenile type, with no mention of complication
C1001	Diabetes mellitus, adult onset, with no mention of complication
C100z	Diabetes mellitus NOS with no mention of complication
C101.	Diabetes mellitus with ketoacidosis
C1010	Diabetes mellitus, juvenile type, with ketoacidosis
C1011	Diabetes mellitus, adult onset, with ketoacidosis
C101y	Other specified diabetes mellitus with ketoacidosis
C101z	Diabetes mellitus NOS with ketoacidosis
C102.	Diabetes mellitus with hyperosmolar coma
C1020	Diabetes mellitus, juvenile type, with hyperosmolar coma
C1021	Diabetes mellitus, adult onset, with hyperosmolar coma
C102z	Diabetes mellitus NOS with hyperosmolar coma
C103.	Diabetes mellitus with ketoacidotic coma
C1030	Diabetes mellitus, juvenile type, with ketoacidotic coma
C1031	Diabetes mellitus, adult onset, with ketoacidotic coma
C103y	Other specified diabetes mellitus with coma
C103z	Diabetes mellitus NOS with ketoacidotic coma
C104.	Diabetes mellitus with renal manifestation
C1040	Diabetes mellitus, juvenile type, with renal manifestation
C1041	Diabetes mellitus, adult onset, with renal manifestation
C104y	Other specified diabetes mellitus with renal complications
C104z	Diabetes mellitus with nephropathy NOS
C105.	Diabetes mellitus with ophthalmic manifestation
C1050	Diabetes mellitus, juvenile type, with ophthalmic manifestation
C1051	Diabetes mellitus, adult onset, with ophthalmic manifestation
C105y	Other specified diabetes mellitus with ophthalmic complications
C105z	Diabetes mellitus NOS with ophthalmic manifestation
C1060	Diabetes mellitus, juvenile type, with neurological manifestation
C1061	Diabetes mellitus, adult onset, with neurological manifestation

For peer review only

C107.	Diabetes mellitus with peripheral circulatory disorder
C1070	Diabetes mellitus, juvenile type, with peripheral circulatory disorder
C1071	Diabetes mellitus, adult onset, with peripheral circulatory disorder
C1072	Diabetes mellitus, adult with gangrene
C1073	IDDM with peripheral circulatory disorder
C1074	NIDDM with peripheral circulatory disorder
C107y	Other specified diabetes mellitus with peripheral circulatory complications
C107z	Diabetes mellitus NOS with peripheral circulatory disorder
C108.	Insulin dependent diabetes mellitus
C1080	Insulin-dependent diabetes mellitus with renal complications
C1081	Insulin-dependent diabetes mellitus with ophthalmic complications
C1082	Insulin-dependent diabetes mellitus with neurological complications
C1083	Insulin dependent diabetes mellitus with multiple complications
C1084	Unstable insulin dependent diabetes mellitus
C1085	Insulin dependent diabetes mellitus with ulcer
C1086	Insulin dependent diabetes mellitus with gangrene
C1087	Insulin dependent diabetes mellitus with retinopathy
C1088	Insulin dependent diabetes mellitus - poor control
C1089	Insulin dependent diabetes maturity onset
C108A	Insulin-dependent diabetes without complication
C108B	Insulin dependent diabetes mellitus with mononeuropathy
C108C	Insulin dependent diabetes mellitus with polyneuropathy
C108D	Insulin dependent diabetes mellitus with nephropathy
C108E	Insulin dependent diabetes mellitus with hypoglycaemic coma
C108F	Insulin dependent diabetes mellitus with diabetic cataract
C108G	Insulin dependent diabetes mellitus with peripheral angiopathy
C108H	Insulin dependent diabetes mellitus with arthropathy
C108J	Insulin dependent diabetes mellitus with neuropathic arthropathy
C108y	Other specified diabetes mellitus with multiple complications
C108z	Unspecified diabetes mellitus with multiple complications
C109.	Non-insulin dependent diabetes mellitus
C1090	Non-insulin-dependent diabetes mellitus with renal complications
C1091	Non-insulin-dependent diabetes mellitus with ophthalmic complications
C1092	Non-insulin-dependent diabetes mellitus with neurological complications
C1093	Non-insulin-dependent diabetes mellitus with multiple complications
C1094	Non-insulin dependent diabetes mellitus with ulcer
C1095	Non-insulin dependent diabetes mellitus with gangrene
C1096	Non-insulin-dependent diabetes mellitus with retinopathy
C1097	Non-insulin dependent diabetes mellitus - poor control
C1099	Non-insulin-dependent diabetes mellitus without complication
C109A	Non-insulin dependent diabetes mellitus with mononeuropathy
C109B	Non-insulin dependent diabetes mellitus with polyneuropathy
C109C	Non-insulin dependent diabetes mellitus with nephropathy
C109D	Non-insulin dependent diabetes mellitus with hypoglycaemic coma
C109E	Non-insulin dependent diabetes mellitus with diabetic cataract
C109F	Non-insulin-dependent diabetes mellitus with peripheral angiopathy
C109G	Non-insulin dependent diabetes mellitus with arthropathy
C109H	Non-insulin dependent diabetes mellitus with neuropathic arthropathy

C109J	Insulin treated Type 2 diabetes mellitus
C109K	Hyperosmolar non-ketotic state in type 2 diabetes mellitus
C10E.	Type 1 diabetes mellitus
C10E0	Type 1 diabetes mellitus with renal complications
C10E1	Type 1 diabetes mellitus with ophthalmic complications
C10E2	Type 1 diabetes mellitus with neurological complications
C10E3	Type 1 diabetes mellitus with multiple complications
C10E4	Unstable type 1 diabetes mellitus
C10E5	Type 1 diabetes mellitus with ulcer
C10E6	Type 1 diabetes mellitus with gangrene
C10E7	Type 1 diabetes mellitus with retinopathy
C10E8	Type 1 diabetes mellitus - poor control
C10E9	Type 1 diabetes mellitus maturity onset
C10EA	Type 1 diabetes mellitus without complication
C10EB	Type 1 diabetes mellitus with mononeuropathy
C10EC	Type 1 diabetes mellitus with polyneuropathy
C10ED	Type 1 diabetes mellitus with nephropathy
C10EE	Type 1 diabetes mellitus with hypoglycaemic coma
C10EF	Type 1 diabetes mellitus with diabetic cataract
C10EG	Type 1 diabetes mellitus with peripheral angiopathy
C10EH	Type 1 diabetes mellitus with arthropathy
C10EJ	Type 1 diabetes mellitus with neuropathic arthropathy
C10EK	Type 1 diabetes mellitus with persistent proteinuria
C10EL	Type 1 diabetes mellitus with persistent microalbuminuria
C10EM	Type 1 diabetes mellitus with ketoacidosis
C10EN	Type 1 diabetes mellitus with ketoacidotic coma
C10EP	Type 1 diabetes mellitus with exudative maculopathy
C10EQ	Type 1 diabetes mellitus with gastroparesis
C10ER	Latent autoimmune diabetes mellitus in adult
C10F.	Type 2 diabetes mellitus
C10F0	Type 2 diabetes mellitus with renal complications
C10F1	Type 2 diabetes mellitus with ophthalmic complications
C10F2	Type 2 diabetes mellitus with neurological complications
C10F3	Type 2 diabetes mellitus with multiple complications
C10F4	Type 2 diabetes mellitus with ulcer
C10F5	Type 2 diabetes mellitus with gangrene
C10F6	Type 2 diabetes mellitus with retinopathy
C10F7	Type 2 diabetes mellitus - poor control
C10F9	Type 2 diabetes mellitus without complication
C10FA	Type 2 diabetes mellitus with mononeuropathy
C10FB	Type 2 diabetes mellitus with polyneuropathy
C10FC	Type 2 diabetes mellitus with nephropathy
C10FD	Type 2 diabetes mellitus with hypoglycaemic coma
C10FE	Type 2 diabetes mellitus with diabetic cataract
C10FF	Type 2 diabetes mellitus with peripheral angiopathy
C10FG	Type 2 diabetes mellitus with arthropathy
C10FH	Type 2 diabetes mellitus with neuropathic arthropathy
C10FJ	Insulin treated Type 2 diabetes mellitus

For peer review only

C10FK	Hyperosmolar non-ketotic state in type 2 diabetes mellitus
C10FL	Type 2 diabetes mellitus with persistent proteinuria
C10FM	Type 2 diabetes mellitus with persistent microalbuminuria
C10FN	Type 2 diabetes mellitus with ketoacidosis
C10FQ	Type 2 diabetes mellitus with exudative maculopathy
C10FR	Type 2 diabetes mellitus with gastroparesis
C10y.	Diabetes mellitus with other specified manifestation
C10y0	Diabetes mellitus, juvenile type, with other specified manifestation
C10y1	Diabetes mellitus, adult onset, with other specified manifestation
C10yy	Other specified diabetes mellitus with other specified complications
C10yz	Diabetes mellitus NOS with other specified manifestation
C10z.	Diabetes mellitus with unspecified complication
C10z0	Diabetes mellitus, juvenile type, with unspecified complication
C10z1	Diabetes mellitus, adult onset, with unspecified complication
C10zy	Other specified diabetes mellitus with unspecified complications
C10zz	Diabetes mellitus NOS with unspecified complication
L180.	Diabetes mellitus during pregnancy, childbirth and the puerperium
L1800	Diabetes mellitus - unspecified whether during pregnancy or the puerperium
L1801	Diabetes mellitus during pregnancy - baby delivered
L1802	Diabetes mellitus in the puerperium - baby delivered during current episode of care
L1803	Diabetes mellitus during pregnancy - baby not yet delivered
L1804	Diabetes mellitus in the pueperium - baby delivered during previous episode of care
L1805	Pre-existing diabetes mellitus, insulin-dependent
L1806	Pre-existing diabetes mellitus, non-insulin-dependent
L1808	Diabetes mellitus arising in pregnancy
L1809	Gestational diabetes mellitus
L180X	Pre-existing diabetes mellitus, unspecified
L180z	Diabetes mellitus during pregnancy, childbirth or the puerperium NOS

Hospital

ALT_CODE	DESCRIPTION
E10	Insulin-dependent diabetes mellitus
E10	Type 1 diabetes mellitus
E100	Insulin-dependent diabetes mellitus
E100	Type 1 diabetes mellitus
E101	Insulin-dependent diabetes mellitus
E101	Type 1 diabetes mellitus
E102	Insulin-dependent diabetes mellitus
E102	Type 1 diabetes mellitus
E103	Insulin-dependent diabetes mellitus
E103	Type 1 diabetes mellitus
E104	Insulin-dependent diabetes mellitus
E104	Type 1 diabetes mellitus

For peer review only

E105	Insulin-dependent diabetes mellitus
E105	Type 1 diabetes mellitus
E106	Insulin-dependent diabetes mellitus
E106	Type 1 diabetes mellitus
E107	Insulin-dependent diabetes mellitus
E107	Type 1 diabetes mellitus
E108	Insulin-dependent diabetes mellitus
E108	Type 1 diabetes mellitus
E109	Insulin-dependent diabetes mellitus
E109	Type 1 diabetes mellitus
E11	Non-insulin-dependent diabetes mellitus
E11	Type 2 diabetes mellitus
E110	Non-insulin-dependent diabetes mellitus
E110	Type 2 diabetes mellitus
E111	Non-insulin-dependent diabetes mellitus
E111	Type 2 diabetes mellitus
E112	Non-insulin-dependent diabetes mellitus
E112	Type 2 diabetes mellitus
E113	Non-insulin-dependent diabetes mellitus
E113	Type 2 diabetes mellitus
E114	Non-insulin-dependent diabetes mellitus
E114	Type 2 diabetes mellitus
E115	Non-insulin-dependent diabetes mellitus
E115	Type 2 diabetes mellitus
E116	Non-insulin-dependent diabetes mellitus
E116	Type 2 diabetes mellitus
E117	Non-insulin-dependent diabetes mellitus
E117	Type 2 diabetes mellitus
E118	Non-insulin-dependent diabetes mellitus
E118	Type 2 diabetes mellitus
E119	Non-insulin-dependent diabetes mellitus
E119	Type 2 diabetes mellitus
E12	Malnutrition-related diabetes mellitus
E120	Malnutrition-related diabetes mellitus
E121	Malnutrition-related diabetes mellitus
E122	Malnutrition-related diabetes mellitus
E123	Malnutrition-related diabetes mellitus
E124	Malnutrition-related diabetes mellitus
E125	Malnutrition-related diabetes mellitus
E126	Malnutrition-related diabetes mellitus
E127	Malnutrition-related diabetes mellitus
E128	Malnutrition-related diabetes mellitus
E129	Malnutrition-related diabetes mellitus
E13	Other specified diabetes mellitus
E130	Other specified diabetes mellitus
E131	Other specified diabetes mellitus
E132	Other specified diabetes mellitus
E133	Other specified diabetes mellitus

For peer review only

E134	Other specified diabetes mellitus
E135	Other specified diabetes mellitus
E136	Other specified diabetes mellitus
E137	Other specified diabetes mellitus
E138	Other specified diabetes mellitus
E139	Other specified diabetes mellitus
E14	Unspecified diabetes mellitus
E140	Unspecified diabetes mellitus
E141	Unspecified diabetes mellitus
E142	Unspecified diabetes mellitus
E143	Unspecified diabetes mellitus
E144	Unspecified diabetes mellitus
E145	Unspecified diabetes mellitus
E146	Unspecified diabetes mellitus
E147	Unspecified diabetes mellitus
E148	Unspecified diabetes mellitus
E149	Unspecified diabetes mellitus
O240	Diabetes mellitus in pregnancy: Pre-existing diabetes mellitus, insulin-dependent
O240	Diabetes mellitus in pregnancy: Pre-existing type 1 diabetes mellitus
O241	Diabetes mellitus in pregnancy: Pre-existing diabetes mellitus, non-insulin-dependent
O241	Diabetes mellitus in pregnancy: Pre-existing type 2 diabetes mellitus
O242	Diabetes mellitus in pregnancy: Pre-existing malnutrition-related diabetes mellitus
O243	Diabetes mellitus in pregnancy: Pre-existing diabetes mellitus, unspecified
O249	Diabetes mellitus in pregnancy, unspecified

Arthritis

READ_CODE	READ_DESC
N....	Musculoskeletal and connective tissue diseases
N0...	Arthropathies and related disorders
N00..	Diffuse diseases of connective tissue
N000.	Systemic lupus erythematosus
N0000	Disseminated lupus erythematosus
N0001	Libman-Sacks disease
N0002	Drug-induced systemic lupus erythematosus
N0003	Systemic lupus erythematosus with organ or system involvement
N0004	Systemic lupus erythematosus with pericarditis
N0005	Neonatal lupus erythematosus
N0006	Cerebral lupus
N000z	Systemic lupus erythematosus NOS
N001.	Scleroderma
N0010	Progressive systemic sclerosis
N0011	CREST syndrome
N0012	Systemic sclerosis induced by drugs and chemicals
N002.	Sicca (Sjogren's) syndrome
N003.	Dermatomyositis

For peer review only

N0030	Juvenile dermatomyositis
N0031	Dermatopolymyositis in neoplastic disease
N003X	Dermatopolymyositis, unspecified
N004.	Polymyositis
N005.	Adult Still's Disease
N006.	Antiphospholipid syndrome
N00y.	Other specified diffuse collagen diseases
N00y0	Eosinophilic fasciitis
N00y1	Fibrosclerosis systemic
N00z.	Collagen disease NOS
N01..	Arthropathy associated with infections
N010.	Pyogenic arthritis
N0100	Pyogenic arthritis of unspecified site
N0101	Pyogenic arthritis of the shoulder region
N0102	Pyogenic arthritis of the upper arm
N0103	Pyogenic arthritis of the forearm
N0104	Pyogenic arthritis of the hand
N0105	Pyogenic arthritis of the pelvic region and thigh
N0106	Pyogenic arthritis of the lower leg
N0107	Pyogenic arthritis of the ankle and foot
N0108	Staphylococcal arthritis and polyarthritis
N0109	Pneumococcal arthritis and polyarthritis
N010A	Arthritis in Lyme disease
N010x	Pyogenic arthritis of multiple sites
N010y	Pyogenic arthritis of other specified sites
N010z	Pyogenic arthritis NOS
N011.	Sexually acquired reactive arthropathy
N0110	Sexually acquired reactive arthropathy of unspecified site
N0111	Sexually acquired reactive arthropathy of the shoulder region
N0112	Sexually acquired reactive arthropathy of the upper arm
N0113	Sexually acquired reactive arthropathy of the forearm
N0114	Sexually acquired reactive arthropathy of the hand
N0115	Sexually acquired reactive arthropathy of the pelvic region and thigh
N0116	Sexually acquired reactive arthropathy of the lower leg
N0117	Sexually acquired reactive arthropathy of the ankle and foot
N011x	Sexually acquired reactive arthropathy of multiple sites
N011y	Sexually acquired reactive arthropathy of other specified site
N011z	Sexually acquired reactive arthropathy NOS
N012.	Arthropathy in Behcet's syndrome
N0120	Arthropathy in Behcet's syndrome of unspecified site
N0121	Arthropathy in Behcet's syndrome of the shoulder region
N0122	Arthropathy in Behcet's syndrome of the upper arm
N0123	Arthropathy in Behcet's syndrome of the forearm
N0124	Arthropathy in Behcet's syndrome of the hand
N0125	Arthropathy in Behcet's syndrome of the pelvic region and thigh
N0126	Arthropathy in Behcet's syndrome of the lower leg
N0127	Arthropathy in Behcet's syndrome of the ankle and foot
N012x	Arthropathy in Behcet's syndrome of multiple sites

For peer review only

N012y	Arthropathy in Behcet's syndrome of other specified sites
N012z	Arthropathy in Behcet's syndrome NOS
N013.	Postdysenteric reactive arthropathy
N0130	Postdysenteric reactive arthropathy of unspecified site
N0131	Postdysenteric reactive arthropathy of the shoulder region
N0132	Postdysenteric reactive arthropathy of the upper arm
N0133	Postdysenteric reactive arthropathy of the forearm
N0134	Postdysenteric reactive arthropathy of the hand
N0135	Postdysenteric reactive arthropathy of the pelvic region and thigh
N0136	Postdysenteric reactive arthropathy of the lower leg
N0137	Postdysenteric reactive arthropathy of the ankle and foot
N013x	Postdysenteric reactive arthropathy of multiple sites
N013y	Postdysenteric reactive arthropathy of other specified sites
N013z	Postdysenteric reactive arthropathy NOS
N014.	Arthropathy associated with other bacterial diseases
N0140	Arthropathy associated with other bacterial disease, of unspecified site
N0141	Arthropathy associated with other bacterial disease, of the shoulder region
N0142	Arthropathy associated with other bacterial disease, of the upper arm
N0143	Arthropathy associated with other bacterial disease, of the forearm
N0144	Arthropathy associated with other bacterial disease, of the hand
N0145	Arthropathy associated with other bacterial disease, of the pelvic region and thigh
N0146	Arthropathy associated with other bacterial disease, of the lower leg
N0147	Arthropathy associated with other bacterial disease, of the ankle and foot
N0148	Arthropathy in Whipple's disease
N014x	Arthropathy associated with other bacterial disease, of multiple sites
N014y	Arthropathy associated with other bacterial disease, of other specified site
N014z	Arthropathy associated with other bacterial disease NOS
N015.	Arthropathy associated with other viral diseases
N0150	Arthropathy associated with other viral disease, of unspecified site
N0151	Arthropathy associated with other viral disease, of the shoulder region
N0152	Arthropathy associated with other viral disease, of the upper arm
N0153	Arthropathy associated with other viral disease, of the forearm
N0154	Arthropathy with other viral disease, of hand
N0155	Arthropathy associated with other viral disease, of the pelvic region and thigh
N0156	Arthropathy associated with other viral disease, of the lower leg
N0157	Arthropathy associated with other viral disease, of the ankle and foot
N015x	Arthropathy associated with other viral disease, of multiple sites
N015y	Arthropathy associated with other viral disease, of other specified site
N015z	Arthropathy associated with other viral disease NOS
N016.	Arthropathy associated with mycoses
N0160	Arthropathy associated with mycoses, of unspecified site
N0161	Arthropathy associated with mycoses, of the shoulder region
N0162	Arthropathy associated with mycoses, of the upper arm
N0163	Arthropathy associated with mycoses, of the forearm
N0164	Arthropathy associated with mycoses, of the hand
N0165	Arthropathy associated with mycoses, of the pelvic region and thigh
N0166	Arthropathy associated with mycoses, of the lower leg
N0167	Arthropathy associated with mycoses, of the ankle and foot

For peer review only

N016x	Arthropathy associated with mycoses, of multiple sites
N016y	Arthropathy associated with mycoses, of other specified site
N016z	Arthropathy associated with mycoses NOS
N017.	Helminthiasis with arthropathy
N0170	Helminthiasis with arthropathy of unspecified site
N0171	Helminthiasis with arthropathy of the shoulder region
N0172	Helminthiasis with arthropathy of the upper arm
N0173	Helminthiasis with arthropathy of the forearm
N0174	Helminthiasis with arthropathy of the hand
N0175	Helminthiasis with arthropathy of the pelvic region and thigh
N0176	Helminthiasis with arthropathy of the lower leg
N0177	Helminthiasis with arthropathy of the ankle and foot
N017x	Helminthiasis with arthropathy of multiple sites
N017y	Helminthiasis with arthropathy of other specified site
N017z	Helminthiasis with arthropathy NOS
N018.	Tuberculous arthritis
N01w.	Reactive arthropathy, unspecified
N01w0	Reactive arthropathy of shoulder
N01w1	Reactive arthropathy of sternoclavicular joint
N01w2	Reactive arthropathy of acromioclavicular joint
N01w3	Reactive arthropathy of elbow
N01w4	Reactive arthropathy of distal radio-ulnar joint
N01w5	Reactive arthropathy of wrist
N01w6	Reactive arthropathy of metacarpophalangeal joint
N01w7	Reactive arthropathy of proximal interphalangeal joint of finger
N01w8	Reactive arthropathy of distal interphalangeal joint of finger
N01w9	Reactive arthropathy of hip
N01wA	Reactive arthropathy of sacro-iliac joint
N01wB	Reactive arthropathy of knee
N01wC	Reactive arthropathy of tibio-fibular joint
N01wD	Reactive arthropathy of ankle
N01wE	Reactive arthropathy of subtalar joint
N01wF	Reactive arthropathy of talonavicular joint
N01wG	Reactive arthropathy of other tarsal joint
N01wH	Reactive arthropathy of 1st metatarsophalangeal joint
N01wJ	Reactive arthropathy of lesser metatarsophalangeal joint
N01wK	Reactive arthropathy of interphalangeal joint of toe
N01y.	Other infectious and parasitic diseases with arthropathy
N01y0	Other infectious and parasitic diseases with arthropathy of unspecified site
N01y1	Other infectious and parasitic diseases with arthropathy of the shoulder region
N01y2	Other infectious and parasitic diseases with arthropathy of the upper arm
N01y3	Other infectious and parasitic diseases with arthropathy of the forearm
N01y4	Other infectious and parasitic diseases with arthropathy of the hand
N01y5	Other infectious and parasitic diseases with arthropathy of the pelvic region and thigh
N01y6	Other infectious and parasitic diseases with arthropathy of the lower leg
N01y7	Other infectious and parasitic diseases with arthropathy of the ankle and foot
N01yx	Other infectious and parasitic diseases with arthropathy of multiple sites

For peer review only

N01yy	Other infectious and parasitic diseases with arthropathy of other specified sites
N01yz	Other infectious and parasitic diseases with arthropathy NOS
N01z.	Infective arthritis NOS
N01z0	Infective arthritis NOS, of unspecified site
N01z1	Infective arthritis NOS, of the shoulder region
N01z2	Infective arthritis NOS, of the upper arm
N01z3	Infective arthritis NOS, of the forearm
N01z4	Infective arthritis NOS, of the hand
N01z5	Infective arthritis NOS, of the pelvic region and thigh
N01z6	Infective arthritis NOS, of the lower leg
N01z7	Infective arthritis NOS, of the ankle and foot
N01z8	Infective arthritis NOS, of shoulder
N01z9	Infective arthritis NOS, of sternoclavicular joint
N01zA	Infective arthritis NOS, of acromioclavicular joint
N01zB	Infective arthritis NOS, of elbow
N01zC	Infective arthritis NOS, of distal radio-ulnar joint
N01zD	Infective arthritis NOS, of wrist
N01zE	Infective arthritis NOS, of metacarpophalangeal joint
N01zF	Infective arthritis NOS, of proximal interphalangeal joint of finger
N01zG	Infective arthritis NOS, of distal interphalangeal joint of finger
N01zH	Infective arthritis NOS, of hip
N01zJ	Infective arthritis NOS, of sacro-iliac joint
N01zK	Infective arthritis NOS, of knee
N01zL	Infective arthritis NOS, of tibio-fibular joint
N01zM	Infective arthritis NOS, of ankle
N01zN	Infective arthritis NOS, of subtalar joint
N01zP	Infective arthritis NOS, of talonavicular joint
N01zQ	Infective arthritis NOS, of other tarsal joint
N01zR	Infective arthritis NOS, of 1st metatarsophalangeal joint
N01zS	Infective arthritis NOS, of lesser metatarsophalangeal joint
N01zT	Infective arthritis NOS, of interphalangeal joint of toe
N01zx	Infective arthritis NOS, of multiple sites
N01zy	Infective arthritis NOS, of other specified site
N01zz	Infective arthritis NOS
N02..	Crystal arthropathies
N020.	Chondrocalcinosis due to dicalcium phosphate crystals
N0200	Chondrocalcinosis due to dicalcium phosphate crystals, of unspecified site
N0201	Chondrocalcinosis due to dicalcium phosphate crystals, of the shoulder region
N0202	Chondrocalcinosis due to dicalcium phosphate crystals, of the upper arm
N0203	Chondrocalcinosis due to dicalcium phosphate crystals, of the forearm
N0204	Chondrocalcinosis due to dicalcium phosphate crystals, of the hand
N0205	Chondrocalcinosis due to dicalcium phosphate crystals, of the pelvic region and thigh
N0206	Chondrocalcinosis due to dicalcium phosphate crystals, of the lower leg
N0207	Chondrocalcinosis due to dicalcium phosphate crystals, of the ankle and foot
N020x	Chondrocalcinosis due to dicalcium phosphate crystals, of multiple sites
N020y	Chondrocalcinosis due to dicalcium phosphate crystals, of other specified site
N020z	Chondrocalcinosis due to dicalcium phosphate crystals, NOS

For peer review only

N021.	Chondrocalcinosis due to pyrophosphate crystals
N0210	Chondrocalcinosis due to pyrophosphate crystals, of unspecified site
N0211	Chondrocalcinosis due to pyrophosphate crystals, of the shoulder region
N0212	Chondrocalcinosis due to pyrophosphate crystals, of the upper arm
N0213	Chondrocalcinosis due to pyrophosphate crystals, of the forearm
N0214	Chondrocalcinosis-pyrophosphate crystals, of the hand
N0215	Chondrocalcinosis due to pyrophosphate crystals, of the pelvic region and thigh
N0216	Chondrocalcinosis due to pyrophosphate crystals, of the lower leg
N0217	Chondrocalcinosis due to pyrophosphate crystals, of the ankle and foot
N021x	Chondrocalcinosis due to pyrophosphate crystals, of multiple sites
N021y	Chondrocalcinosis due to pyrophosphate crystals, of other specified site
N021z	Chondrocalcinosis due to pyrophosphate crystals, NOS
N022.	Chondrocalcinosis, unspecified
N0220	Chondrocalcinosis unspecified, of unspecified site
N0221	Chondrocalcinosis unspecified, of the shoulder region
N0222	Chondrocalcinosis unspecified, of the upper arm
N0223	Chondrocalcinosis unspecified, of the forearm
N0224	Chondrocalcinosis unspecified, of the hand
N0225	Chondrocalcinosis unspecified, of the pelvic region and thigh
N0226	Chondrocalcinosis unspecified, of the lower leg
N0227	Chondrocalcinosis unspecified, of the ankle and foot
N022x	Chondrocalcinosis unspecified, of multiple sites
N022y	Chondrocalcinosis unspecified, of other specified site
N022z	Chondrocalcinosis NOS
N023.	Gouty arthritis
N0230	Gouty arthritis of unspecified site
N0231	Gouty arthritis of the shoulder region
N0232	Gouty arthritis of the upper arm
N0233	Gouty arthritis of the forearm
N0234	Gouty arthritis of the hand
N0235	Gouty arthritis of the pelvic region and thigh
N0236	Gouty arthritis of the lower leg
N0237	Gouty arthritis of the ankle and foot
N0238	Gouty arthritis of toe
N023x	Gouty arthritis of multiple sites
N023y	Gouty arthritis of other specified site
N023z	Gouty arthritis NOS
N024.	Familial chondrocalcinosis
N02y.	Other crystal arthropathies
N02y0	Other crystal arthropathies of unspecified site
N02y1	Other crystal arthropathies of the shoulder
N02y2	Other crystal arthropathies of the upper arm
N02y3	Other crystal arthropathies of the forearm
N02y4	Other crystal arthropathies of the hand
N02y5	Other crystal arthropathies of the pelvic region and thigh
N02y6	Other crystal arthropathies of the lower leg
N02y7	Other crystal arthropathies of the ankle and foot
N02y8	Hydroxyapatite deposition disease

For peer review only

N02yx	Other crystal arthropathies of multiple sites
N02yy	Other crystal arthropathies of other specified sites
N02yz	Other crystal arthropathy NOS
N02z.	Crystal arthropathy NOS
N02z0	Crystal arthropathy NOS, site unspecified
N02z1	Crystal arthropathy NOS, of the shoulder region
N02z2	Crystal arthropathy NOS, of the upper arm
N02z3	Crystal arthropathy NOS, of the forearm
N02z4	Crystal arthropathy NOS, of the hand
N02z5	Crystal arthropathy NOS, of the pelvic region and thigh
N02z6	Crystal arthropathy NOS, of the lower leg
N02z7	Crystal arthropathy NOS, of the ankle and foot
N02z8	Crystal arthropathy NOS, of shoulder
N02z9	Crystal arthropathy NOS, of sternoclavicular joint
N02zA	Crystal arthropathy NOS, of acromioclavicular joint
N02zB	Crystal arthropathy NOS, of elbow
N02zC	Crystal arthropathy NOS, of distal radio-ulnar joint
N02zD	Crystal arthropathy NOS, of wrist
N02zE	Crystal arthropathy NOS, of metacarpophalangeal joint
N02zF	Crystal arthropathy NOS, of proximal interphalangeal joint of finger
N02zG	Crystal arthropathy NOS, of distal interphalangeal joint of finger
N02zH	Crystal arthropathy NOS, of hip
N02zJ	Crystal arthropathy NOS, of sacro-iliac joint
N02zK	Crystal arthropathy NOS, of knee
N02zL	Crystal arthropathy NOS, of tibio-fibular joint
N02zM	Crystal arthropathy NOS, of ankle
N02zN	Crystal arthropathy NOS, of subtalar joint
N02zP	Crystal arthropathy NOS, of talonavicular joint
N02zQ	Crystal arthropathy NOS, of other tarsal joint
N02zR	Crystal arthropathy NOS, of 1st metatarsophalangeal joint
N02zS	Crystal arthropathy NOS, of lesser metatarsophalangeal joint
N02zT	Crystal arthropathy NOS, of interphalangeal joint of toe
N02zx	Crystal arthropathy NOS, of multiple sites
N02zy	Crystal arthropathy NOS, of other specified site
N02zz	Crystal arthropathy NOS
N03..	Arthropathy associated with disorders EC
N030.	Arthropathy associated with endocrine and metabolic disorder
N0300	Diabetic cheiroarthropathy
N0301	Diabetic Charcot arthropathy
N0302	Arthropathy in amyloidosis
N031.	Arthropathy associated with non-infective gastrointestinal disorders
N0310	Arthropathy in ulcerative colitis
N0311	Arthropathy in Crohn's disease
N0312	Arthropathy in Whipple's disease
N0313	Arthropathy following intestinal bypass
N032.	Arthropathy associated with haematological disorders
N0320	Arthropathy due to haemophilia
N033.	Arthropathy associated with dermatological disorders

For peer review only

N034.	Arthropathy associated with respiratory disorders
N035.	Neuropathic arthropathy
N036.	Arthropathy due to hypersensitivity reaction
N037.	Postimmunization arthropathy
N038.	Reactive arthropathies
N0380	Postmeningococcal arthritis
N0381	Postinfective arthropathy in syphilis
N039.	Beta-2 microglobulin arthropathy
N03x.	Other general diseases with associated arthropathy
N03x0	Arthritis associated with other disease, shoulder
N03x1	Arthritis associated with other disease, sternoclavicular joint
N03x2	Arthritis associated with other disease, acromioclavicular joint
N03x3	Arthritis associated with other disease, elbow
N03x4	Arthritis associated with other disease, distal radio-ulnar joint
N03x5	Arthritis associated with other disease, wrist
N03x6	Arthritis associated with other disease, metacarpophalangeal joint
N03x7	Arthritis associated with other disease, proximal interphalangeal joint of finger
N03x8	Arthritis associated with other disease, distal interphalangeal joint of finger
N03x9	Arthritis associated with other disease, hip
N03xA	Arthritis associated with other disease, sacro-iliac joint
N03xB	Arthritis associated with other disease, knee
N03xC	Arthritis associated with other disease, tibio-fibular joint
N03xD	Arthritis associated with other disease, ankle
N03xE	Arthritis associated with other disease, subtalar joint
N03xF	Arthritis associated with other disease, talonavicular joint
N03xG	Arthritis associated with other disease, other tarsal joint
N03xH	Arthritis associated with other disease, 1st metatarsophalangeal joint
N03xJ	Arthritis associated with other disease, lesser metatarsophalangeal joint
N03xK	Arthritis associated with other disease, interphalangeal joint of toe
N03y.	Arthropathy associated with other conditions EC
N03z.	Arthropathy associated with disorders EC NOS
N04..	Rheumatoid arthritis and other inflammatory polyarthropathies
N040.	Rheumatoid arthritis
N0400	Rheumatoid arthritis of cervical spine
N0401	Other rheumatoid arthritis of spine
N0402	Rheumatoid arthritis of shoulder
N0403	Rheumatoid arthritis of sternoclavicular joint
N0404	Rheumatoid arthritis of acromioclavicular joint
N0405	Rheumatoid arthritis of elbow
N0406	Rheumatoid arthritis of distal radio-ulnar joint
N0407	Rheumatoid arthritis of wrist
N0408	Rheumatoid arthritis of metacarpophalangeal joint
N0409	Rheumatoid arthritis of proximal interphalangeal joint of finger
N040A	Rheumatoid arthritis of distal interphalangeal joint of finger
N040B	Rheumatoid arthritis of hip
N040C	Rheumatoid arthritis of sacro-iliac joint
N040D	Rheumatoid arthritis of knee
N040E	Rheumatoid arthritis of tibio-fibular joint

For peer review only

N040F	Rheumatoid arthritis of ankle
N040G	Rheumatoid arthritis of subtalar joint
N040H	Rheumatoid arthritis of talonavicular joint
N040J	Rheumatoid arthritis of other tarsal joint
N040K	Rheumatoid arthritis of 1st metatarsophalangeal joint
N040L	Rheumatoid arthritis of lesser metatarsophalangeal joint
N040M	Rheumatoid arthritis of interphalangeal joint of toe
N040N	Rheumatoid vasculitis
N040P	Seronegative rheumatoid arthritis
N040Q	Rheumatoid bursitis
N040R	Rheumatoid nodule
N040S	Rheumatoid arthritis - multiple joint
N040T	Flare of rheumatoid arthritis
N041.	Felty's syndrome
N042.	Other rheumatoid arthropathy with visceral or systemic involvement
N0420	Rheumatic carditis
N0421	Rheumatoid lung disease
N0422	Rheumatoid nodule
N042z	Rheumatoid arthropathy with visceral or systemic involvement NOS
N043.	Juvenile rheumatoid arthritis - Still's disease
N0430	Juvenile rheumatoid arthropathy unspecified
N0431	Acute polyarticular juvenile rheumatoid arthritis
N0432	Pauciarticular juvenile rheumatoid arthritis
N0433	Monarticular juvenile rheumatoid arthritis
N043z	Juvenile rheumatoid arthritis NOS
N044.	Chronic post-rheumatic arthropathy
N045.	Other juvenile arthritis
N0450	Juvenile ankylosing spondylitis
N0451	Juvenile seronegative polyarthritis
N0452	Juvenile arthritis in psoriasis
N0453	Juvenile arthritis in Crohn's disease
N0454	Juvenile arthritis in ulcerative colitis
N0455	Juvenile rheumatoid arthritis
N0456	Pauciarticular onset juvenile chronic arthritis
N047.	Seropositive errosive rheumatoid arthritis
N04X.	Seropositive rheumatoid arthritis, unspecified
N04y.	Other specified inflammatory polyarthropathy
N04y0	Rheumatoid lung
N04y1	Sero negative arthritis
N04y2	Adult-onset Still's disease
N04y3	Remitting seronegative symmetrical synovitis with pitting oedema
N04yz	Other specified inflammatory polyarthropathy NOS
N04z.	Inflammatory polyarthropathy NOS
N05..	Osteoarthritis and allied disorders
N050.	Generalised osteoarthritis - OA
N0500	Generalised osteoarthritis of unspecified site
N0501	Generalised osteoarthritis of the hand
N0502	Generalised osteoarthritis of multiple sites

For peer review only

N0503	Bouchard's nodes with arthropathy
N0504	Primary generalized osteoarthrosis
N0505	Secondary multiple arthrosis
N0506	Erosive osteoarthrosis
N0507	Heberden's nodes with arthropathy
N050z	Generalised osteoarthritis NOS
N051.	Localised, primary osteoarthritis
N0510	Localised, primary osteoarthritis of unspecified site
N0511	Localised, primary osteoarthritis of the shoulder region
N0512	Localised, primary osteoarthritis of the upper arm
N0513	Localised, primary osteoarthritis of the forearm
N0514	Localised, primary osteoarthritis of the hand
N0515	Localised, primary osteoarthritis of the pelvic region and thigh
N0516	Localised, primary osteoarthritis of the lower leg
N0517	Localised, primary osteoarthritis of the ankle and foot
N0518	Localised, primary osteoarthritis of other specified site
N0519	Primary coxarthrosis, bilateral
N051A	Coxarthrosis resulting from dysplasia, bilateral
N051B	Primary gonarthrosis, bilateral
N051C	Primary arthrosis of first carpometacarpal joints, bilateral
N051D	Localised, primary osteoarthritis of the wrist
N051E	Localised, primary osteoarthritis of toe
N051F	Localised, primary osteoarthritis of elbow
N051G	Osteoarthritis of spinal facet joint
N051z	Localised, primary osteoarthritis NOS
N052.	Localised, secondary osteoarthritis
N0520	Localised, secondary osteoarthritis of unspecified site
N0521	Localised, secondary osteoarthritis of the shoulder region
N0522	Localised, secondary osteoarthritis of the upper arm
N0523	Localised, secondary osteoarthritis of the forearm
N0524	Localised, secondary osteoarthritis of the hand
N0525	Localised, secondary osteoarthritis of the pelvic region and thigh
N0526	Localised, secondary osteoarthritis of the lower leg
N0527	Localised, secondary osteoarthritis of the ankle and foot
N0528	Localised, secondary osteoarthritis of other specified site
N0529	Post-traumatic coxarthrosis, bilateral
N052A	Post-traumatic gonarthrosis, bilateral
N052B	Post-traumatic arthrosis of first carpometacarpal joints, bilateral
N052C	Post-traumatic gonarthrosis, unilateral
N052z	Localised, secondary osteoarthritis NOS
N053.	Localised osteoarthritis, unspecified
N0530	Localised osteoarthritis, unspecified, of unspecified site
N0531	Localised osteoarthritis, unspecified, of the shoulder region
N0532	Localised osteoarthritis, unspecified, of the upper arm
N0533	Localised osteoarthritis, unspecified, of the forearm
N0534	Localised osteoarthritis, unspecified, of the hand
N0535	Localised osteoarthritis, unspecified, of the pelvic region and thigh
N0536	Localised osteoarthritis, unspecified, of the lower leg

For peer review only

N0537	Localised osteoarthritis, unspecified, of the ankle and foot
N0538	Localised osteoarthritis, unspecified, of other specified site
N0539	Arthrosis of first carpometacarpal joint, unspecified
N053z	Localised osteoarthritis, unspecified, NOS
N054.	Oligoarticular osteoarthritis, unspecified
N0540	Oligoarticular osteoarthritis, unspecified, of unspecified sites
N0541	Oligoarticular osteoarthritis, unspecified, of the shoulder region
N0542	Oligoarticular osteoarthritis, unspecified, of upper arm
N0543	Oligoarticular osteoarthritis, unspecified, of forearm
N0544	Oligoarticular osteoarthritis, unspecified, of hand
N0545	Oligoarticular osteoarthritis, unspecified, of the pelvic region and thigh
N0546	Oligoarticular osteoarthritis, unspecified, of lower leg
N0547	Oligoarticular osteoarthritis, unspecified, of ankle and foot
N0548	Oligoarticular osteoarthritis, unspecified, of other specified sites
N0549	Oligoarticular osteoarthritis, unspecified, of multiple sites
N054z	Osteoarthritis of more than one site, unspecified, NOS
N05z.	Osteoarthritis NOS
N05z0	Osteoarthritis NOS, of unspecified site
N05z1	Osteoarthritis NOS, of shoulder region
N05z2	Osteoarthritis NOS, of the upper arm
N05z3	Osteoarthritis NOS, of the forearm
N05z4	Osteoarthritis NOS, of the hand
N05z5	Osteoarthritis NOS, pelvic region/thigh
N05z6	Osteoarthritis NOS, of the lower leg
N05z7	Osteoarthritis NOS, of ankle and foot
N05z8	Osteoarthritis NOS, other specified site
N05z9	Osteoarthritis NOS, of shoulder
N05zA	Osteoarthritis NOS, of sternoclavicular joint
N05zB	Osteoarthritis NOS, of acromioclavicular joint
N05zC	Osteoarthritis NOS, of elbow
N05zD	Osteoarthritis NOS, of distal radio-ulnar joint
N05zE	Osteoarthritis NOS, of wrist
N05zF	Osteoarthritis NOS, of metacarpophalangeal joint
N05zG	Osteoarthritis NOS, of proximal interphalangeal joint of finger
N05zH	Osteoarthritis NOS, of distal interphalangeal joint of finger
N05zJ	Osteoarthritis NOS, of hip
N05zK	Osteoarthritis NOS, of sacro-iliac joint
N05zL	Osteoarthritis NOS, of knee
N05zM	Osteoarthritis NOS, of tibio-fibular joint
N05zN	Osteoarthritis NOS, of ankle
N05zP	Osteoarthritis NOS, of subtalar joint
N05zQ	Osteoarthritis NOS, of talonavicular joint
N05zR	Osteoarthritis NOS, of other tarsal joint
N05zS	Osteoarthritis NOS, of 1st metatarsophalangeal joint
N05zT	Osteoarthritis NOS, of lesser metatarsophalangeal joint
N05zU	Osteoarthritis NOS, of interphalangeal joint of toe
N05zz	Osteoarthritis NOS
N06..	Other and unspecified arthropathies

For peer review only

N060.	Kaschin - Beck disease
N0600	Kaschin-Beck disease of unspecified site
N0601	Kaschin-Beck disease of the shoulder region
N0602	Kaschin-Beck disease of the upper arm
N0603	Kaschin-Beck disease of the forearm
N0604	Kaschin-Beck disease of the hand
N0605	Kaschin-Beck disease of the pelvic region and thigh
N0606	Kaschin-Beck disease of the lower leg
N0607	Kaschin-Beck disease of the ankle and foot
N0608	Kaschin-Beck disease of other specified site
N0609	Kaschin-Beck disease of multiple sites
N060z	Kaschin-Beck disease NOS
N061.	Traumatic arthropathy
N0610	Traumatic arthropathy of unspecified site
N0611	Traumatic arthropathy of the shoulder region
N0612	Traumatic arthropathy of the upper arm
N0613	Traumatic arthropathy of the forearm
N0614	Traumatic arthropathy of the hand
N0615	Traumatic arthropathy of the pelvic region and thigh
N0616	Traumatic arthropathy of the lower leg
N0617	Traumatic arthropathy of the ankle and foot
N0618	Traumatic arthropathy of other specified site
N0619	Traumatic arthropathy of multiple sites
N061A	Traumatic arthropathy of shoulder
N061B	Traumatic arthropathy of sternoclavicular joint
N061C	Traumatic arthropathy of acromioclavicular joint
N061D	Traumatic arthropathy-elbow
N061E	Traumatic arthropathy of distal radio-ulnar joint
N061F	Traumatic arthropathy-wrist
N061G	Traumatic arthropathy of metacarpophalangeal joint
N061H	Traumatic arthropathy of proximal interphalangeal joint of finger
N061J	Traumatic arthropathy of distal interphalangeal joint of finger
N061K	Traumatic arthropathy-hip
N061L	Traumatic arthropathy of sacro-iliac joint
N061M	Traumatic arthropathy-knee
N061N	Traumatic arthropathy of tibio-fibular joint
N061P	Traumatic arthropathy-ankle
N061Q	Traumatic arthropathy of subtalar joint
N061R	Traumatic arthropathy of talonavicular joint
N061S	Traumatic arthropathy of other tarsal joint
N061T	Traumatic arthropathy of 1st metatarsophalangeal joint
N061U	Traumatic arthropathy of lesser metatarsophalangeal joint
N061V	Traumatic arthropathy of interphalangeal joint of toe
N061z	Traumatic arthropathy NOS
N062.	Allergic arthritis
N0620	Allergic arthritis of unspecified site
N0621	Allergic arthritis of the shoulder region
N0622	Allergic arthritis of the upper arm

For peer review only

N0623	Allergic arthritis of the forearm
N0624	Allergic arthritis of the hand
N0625	Allergic arthritis of the pelvic region and thigh
N0626	Allergic arthritis of the lower leg
N0627	Allergic arthritis of the ankle and foot
N0628	Allergic arthritis of other specified site
N0629	Allergic arthritis of multiple sites
N062z	Allergic arthritis NOS
N063.	Climacteric arthritis
N0630	Climacteric arthritis of unspecified site
N0631	Climacteric arthritis of the shoulder region
N0632	Climacteric arthritis of the upper arm
N0633	Climacteric arthritis of the forearm
N0634	Climacteric arthritis of the hand
N0635	Climacteric arthritis of the pelvic region and thigh
N0636	Climacteric arthritis of the lower leg
N0637	Climacteric arthritis of the ankle and foot
N0638	Climacteric arthritis of other specified site
N0639	Climacteric arthritis of multiple sites
N063z	Climacteric arthritis NOS
N064.	Transient arthropathy
N0640	Transient arthropathy of unspecified site
N0641	Transient arthropathy of the shoulder region
N0642	Transient arthropathy of the upper arm
N0643	Transient arthropathy of the forearm
N0644	Transient arthropathy of the hand
N0645	Transient arthropathy of the pelvic region and thigh
N0646	Transient arthropathy of the lower leg
N0647	Transient arthropathy of the ankle and foot
N0648	Transient arthropathy of other specified site
N0649	Transient arthropathy of multiple sites
N064A	Transient arthropathy of shoulder
N064B	Transient arthropathy of sternoclavicular joint
N064C	Transient arthropathy of acromioclavicular joint
N064D	Transient arthropathy-elbow
N064E	Transient arthropathy of distal radio-ulnar joint
N064F	Transient arthropathy-wrist
N064G	Transient arthropathy of metacarpophalangeal joint
N064H	Transient arthropathy of proximal interphalangeal joint of finger
N064J	Transient arthropathy of distal interphalangeal joint of finger
N064K	Transient arthropathy-hip
N064L	Transient arthropathy of sacro-iliac joint
N064M	Transient arthropathy-knee
N064N	Transient arthropathy of tibio-fibular joint
N064P	Transient arthropathy-ankle
N064Q	Transient arthropathy of subtalar joint
N064R	Transient arthropathy of talonavicular joint
N064S	Transient arthropathy of other tarsal joint

For peer review only

N064T	Transient arthropathy of 1st metatarsophalangeal joint
N064U	Transient arthropathy of lesser metatarsophalangeal joint
N064V	Transient arthropathy of interphalangeal joint of toe
N064z	Transient arthropathy NOS
N065.	Unspecified polyarthropathy or polyarthritis
N0650	Unspecified polyarthropathy of unspecified site
N0651	Unspecified polyarthropathy of the shoulder region
N0652	Unspecified polyarthropathy of the upper arm
N0653	Unspecified polyarthropathy of the forearm
N0654	Unspecified polyarthropathy of the hand
N0655	Unspecified polyarthropathy of the pelvic region and thigh
N0656	Unspecified polyarthropathy of the lower leg
N0657	Unspecified polyarthropathy of the ankle and foot
N0658	Unspecified polyarthropathy of other specified site
N0659	Unspecified polyarthropathy of multiple sites
N065A	Generalised arthritis
N065z	Unspecified polyarthropathy or polyarthritis NOS
N066.	Unspecified monoarthritis
N0660	Unspecified monoarthritis of unspecified site
N0661	Unspecified monoarthritis of the shoulder region
N0662	Unspecified monoarthritis of the upper arm
N0663	Unspecified monoarthritis of the forearm
N0664	Unspecified monoarthritis of the hand
N0665	Unspecified monoarthritis of the pelvic region and thigh
N0666	Unspecified monoarthritis of the lower leg
N0667	Unspecified monoarthritis of the ankle and foot
N0668	Unspecified monoarthritis of other specified site
N066z	Unspecified monoarthritis NOS
N067.	Ochronotic arthropathy
N068.	Haemophilic arthropathy
N069.	Arthropathy in neoplastic disease
N06y.	Other specified arthropathy
N06y0	Other specified arthropathy of unspecified site
N06y1	Other specified arthropathy of the shoulder region
N06y2	Other specified arthropathy of the upper arm
N06y3	Other specified arthropathy of the forearm
N06y4	Other specified arthropathy of the hand
N06y5	Other specified arthropathy of the pelvic region and thigh
N06y6	Other specified arthropathy of the lower leg
N06y7	Other specified arthropathy of the ankle and foot
N06y8	Other specified arthropathy of other specified site
N06y9	Other specified arthropathy of multiple sites
N06yz	Other specified arthropathy NOS
N06z.	Arthropathy NOS
N06z0	Arthropathy NOS, of unspecified site
N06z1	Arthropathy NOS, of the shoulder region
N06z2	Arthropathy NOS, of the upper arm
N06z3	Arthropathy NOS, of the forearm

For peer review only

N06z4	Arthropathy NOS, of the hand
N06z5	Arthropathy NOS, of the pelvic region and thigh
N06z6	Arthropathy NOS, of the lower leg
N06z7	Arthropathy NOS, of the ankle and foot
N06z8	Arthropathy NOS, of other specified site
N06z9	Arthropathy NOS, of multiple sites
N06zA	Acute arthritis
N06zB	Chronic arthritis
N06zz	Arthropathy NOS
N07..	Internal derangement of knee
N070.	Medial meniscus derangement
N0700	Medial meniscus derangement, unspecified
N0701	Old bucket handle tear of medial meniscus
N0702	Medial meniscus, anterior horn derangement
N0703	Medial meniscus, posterior horn derangement
N0704	Parrot beak tear of posterior horn of medial meniscus
N0705	Peripheral detachment of medial meniscus
N0706	Radial tear of medial meniscus
N0707	Horizontal cleavage tear of medial meniscus
N0708	Multiple tears of medial meniscus
N0709	Cyst of medial meniscus
N070A	Old tear of medial meniscus
N070B	Old tear of posterior horn of medial meniscus
N070z	Medial meniscus derangement NOS
N071.	Lateral meniscus derangement
N0710	Lateral meniscus derangement unspecified
N0711	Old bucket handle tear of lateral meniscus
N0712	Lateral meniscus, anterior horn derangement
N0713	Lateral meniscus, posterior horn derangement
N0714	Lateral meniscus derangement NOS
N0715	Parrot beak tear of posterior horn of lateral meniscus
N0716	Peripheral detachment of lateral meniscus
N0717	Radial tear of lateral meniscus
N0718	Horizontal cleavage tear of lateral meniscus
N0719	Multiple tears of lateral meniscus
N071A	Cyst of lateral meniscus
N071B	Discoid lateral meniscus
N071C	Old tear of lateral meniscus
N072.	Meniscus derangement NEC
N0720	Old torn meniscus of knee
N0721	Degenerative lesion of articular cartilage of knee
N0722	Cyst of semilunar cartilage
N073.	Loose body in knee
N074.	Chondromalacia patellae
N07y.	Other internal knee derangement
N07y0	Old lateral collateral ligament disruption
N07y1	Old medial collateral ligament disruption
N07y2	Old anterior cruciate ligament disruption

For peer review only

N07y3	Old posterior cruciate ligament disruption
N07y4	Old capsular knee ligament disruption
N07y5	Locked knee
N07y6	Patellofemoral maltracking
N07y7	Old partial tear lateral collateral ligament
N07y8	Old complete tear lateral collateral ligament
N07y9	Old posterolateral capsular complex tear
N07yA	Old partial tear medial collateral ligament
N07yB	Old complete tear medial collateral ligament
N07yC	Old medial capsular complex tear
N07yD	Old partial tear anterior cruciate ligament
N07yE	Old complete tear anterior cruciate ligament
N07yF	Old partial tear posterior cruciate ligament
N07yG	Old complete tear posterior cruciate ligament
N07yH	Locking knee
N07yJ	Adhesions of knee joint
N07yy	Other old knee ligament disruption
N07yz	Other internal knee derangement NOS
N07z.	Internal knee derangement NOS
N08..	Other derangement of joint
N080.	Articular cartilage disorder, excluding the knee
N0800	Articular cartilage disorder, of unspecified site
N0801	Articular cartilage disorder of the shoulder region
N0802	Articular cartilage disorder of the upper arm
N0803	Articular cartilage disorder of the forearm
N0804	Articular cartilage disorder of the hand
N0805	Articular cartilage disorder of the pelvic region and thigh
N0806	Articular cartilage disorder of the ankle and foot
N0807	Articular cartilage disorder of other specified site
N0808	Articular cartilage disorder of multiple sites
N0809	Hill-Sachs lesion
N080A	Reverse Hill-Sachs lesion
N080B	Articular cartilage disorder of other joints of the shoulder girdle
N080C	Chondrolysis of the femoral head
N080z	Articular cartilage disorder NOS
N081.	Loose body in joint, excluding the knee
N0810	Loose body in joint, unspecified joint
N0811	Loose body in joint of shoulder region
N0812	Loose body in joint of upper arm
N0813	Loose body in joint of forearm
N0814	Loose body in joint of hand
N0815	Loose body in joint of pelvic region and thigh
N0816	Loose body in joint of ankle and foot
N0817	Loose body in joint, other specified joint
N0818	Loose joint body in multiple joints
N0819	Loose body in shoulder joint
N081A	Loose body in other joint of shoulder girdle
N081B	Loose body in elbow joint

For peer review only

N081C	Loose body in wrist joint
N081D	Loose body in hip joint
N081E	Loose body in other joint of pelvic girdle
N081F	Loose body in ankle joint
N081G	Loose body in foot joint
N081z	Loose body in joint (excluding the knee) NOS
N082.	Pathological dislocation
N0820	Pathological dislocation of unspecified site
N0821	Pathological dislocation of the shoulder region
N0822	Pathological dislocation of the upper arm
N0823	Pathological dislocation of the forearm
N0824	Pathological dislocation of the hand
N0825	Pathological dislocation of the pelvic region and thigh
N0826	Pathological dislocation of the lower leg
N0827	Pathological dislocation of the ankle and foot
N0828	Pathological dislocation of other specified site
N0829	Pathological dislocation of multiple joints
N082A	Pathological dislocation of the shoulder joint
N082B	Pathological dislocation of other joint of shoulder girdle
N082C	Pathological dislocation of humero-ulnar joint
N082D	Pathological dislocation of superior radio-ulnar joint
N082E	Pathological dislocation of radial head
N082F	Pathological dislocation of inferior radio-ulnar joint
N082G	Pathological dislocation of wrist joint
N082H	Pathological dislocation of 1st carpometacarpal joint
N082J	Pathological dislocation of other carpometacarpal joint
N082K	Pathological dislocation of metacarpophalangeal joint
N082L	Pathological dislocation of proximal interphalangeal joint
N082M	Pathological dislocation of distal interphalangeal joint
N082N	Neuromuscular dislocation of the hip
N082P	Other acquired pathological dislocation of the hip
N082Q	Pathological dislocation of knee joint
N082R	Pathological dislocation of patellofemoral joint
N082S	Pathological dislocation of ankle joint
N082T	Pathological dislocation of subtalar joint
N082U	Pathological dislocation of midtarsal joint
N082V	Pathological dislocation of tarsometatarsal joint
N082W	Pathological dislocation of 1st metatarsophalangeal joint
N082X	Pathological dislocation of lesser metatarsophalangeal joint
N082Y	Pathological dislocation of toe interphalangeal joint
N082Z	Non-traumatic subluxation of acromioclavicular joint
N082z	Pathological dislocation NOS
N083.	Recurrent dislocation of joint
N0830	Recurrent joint dislocation, of unspecified site
N0831	Recurrent joint dislocation, of the shoulder region
N0832	Recurrent joint dislocation, of the upper arm
N0833	Recurrent joint dislocation, of the forearm
N0834	Recurrent joint dislocation, of the hand

For peer review only

N0835	Recurrent joint dislocation, of the pelvic region and thigh
N0836	Recurrent joint dislocation, of the lower leg
N0837	Recurrent joint dislocation, of the ankle and foot
N0838	Recurrent joint dislocation, of other specified site
N0839	Recurrent dislocation of multiple joints
N083A	Recurrent dislocation of shoulder - anterior
N083B	Recurrent dislocation of shoulder - posterior
N083C	Recurrent subluxation of shoulder - anterior
N083D	Recurrent subluxation of shoulder - posterior
N083E	Recurrent dislocation of shoulder - inferior
N083F	Recurrent subluxation of shoulder - inferior
N083G	Recurrent dislocation of shoulder - anterior
N083H	Recurrent subluxation of shoulder - anterior
N083J	Recurrent dislocation of shoulder - multidirectional
N083K	Recurrent subluxation of shoulder - multidirectional
N083L	Habitual dislocation of the shoulder
N083M	Habitual subluxation of the shoulder
N083N	Recurrent dislocation of elbow
N083P	Recurrent subluxation of elbow
N083Q	Recurrent dislocation of superior radio-ulnar joint
N083R	Recurrent subluxation of superior radio-ulnar joint
N083S	Recurrent dislocation of radial head
N083T	Recurrent subluxation of radial head
N083U	Recurrent dislocation of inferior radio-ulnar joint
N083V	Recurrent subluxation of inferior radio-ulnar joint
N083W	Recurrent dislocation of wrist
N083X	Carpal instability
N083Y	Recurrent subluxation of wrist
N083Z	Carpal instability, dorsal intercalated segment instability
N083a	Carpal instability, ventral intercalated segment instability
N083b	Carpal instability, ulnar translocation
N083c	Carpal instability, dorsal subluxation
N083d	Carpal instability, other
N083e	Recurrent dislocation of carpometacarpal joint
N083f	Recurrent subluxation of carpometacarpal joint
N083g	Recurrent dislocation of metacarpophalangeal joint
N083h	Recurrent subluxation of metacarpophalangeal joint
N083j	Recurrent dislocation of interphalangeal joint
N083k	Recurrent subluxation of interphalangeal joint
N083l	Recurrent dislocation of the hip
N083m	Recurrent dislocation of other pelvic joints
N083n	Recurrent dislocation of the knee
N083p	Recurrent dislocation of the patella
N083q	Recurrent subluxation of the patella
N083r	Habitual dislocation of the patella
N083s	Recurrent dislocation of the ankle
N083t	Recurrent subluxation of the ankle
N083u	Recurrent dislocation of foot joint

For peer review only

N083v	Recurrent subluxation of the subtalar joint
N083w	Recurrent subluxation of other foot joint
N083x	Recurrent subluxation of the hip joint
N083z	Recurrent joint dislocation NOS
N084.	Contracture of joint
N0840	Joint contracture of unspecified site
N0841	Joint contracture of the shoulder region
N0842	Joint contracture of the upper arm
N0843	Joint contracture of the forearm
N0844	Joint contracture of the hand
N0845	Joint contracture of the pelvic region and thigh
N0846	Joint contracture of the lower leg
N0847	Joint contracture of the ankle and foot
N0848	Joint contracture of other specified site
N0849	Contracture of multiple joints
N084A	Flexion contracture-shoulder
N084B	Extension contracture-shoulder
N084C	Abduction contracture-shoulder
N084D	Adduction contracture-shoulder
N084E	Internal rotation contracture-shoulder
N084F	External rotation contracture-shoulder
N084G	Flexion contracture - elbow
N084H	Extension contracture - elbow
N084J	Pronation contracture - forearm
N084K	Supination contracture - forearm
N084L	Flexion contracture - wrist
N084M	Extension contracture of the wrist
N084N	Ulnar deviation contracture of the wrist
N084P	Radial deviation contracture of the wrist
N084Q	Flexion contracture of metacarpophalangeal joint
N084R	Extension contracture of metacarpophalangeal joint
N084S	Flexion contracture of proximal interphalangeal joint
N084T	Flexion contracture of distal interphalangeal joint
N084U	Flexion contracture of hip
N084V	Extension contracture of hip
N084W	Abduction contracture of hip
N084X	Adduction contracture of hip
N084Y	Internal rotation contracture of hip
N084Z	External rotation contracture of hip
N084a	Flexion contracture of the knee
N084b	Equinus contracture of the ankle
N084c	Calcaneus contracture of the ankle
N084d	Flexion contracture of metatarsophalangeal joint
N084e	Extension contracture of metatarsophalangeal joint
N084f	Flexion contracture of toe interphalangeal joint
N084g	Extension contracture of toe interphalangeal joint
N084z	Contracture of joint NOS
N085.	Ankylosis of joint

For peer review only

N0850	Joint ankylosis of unspecified site
N0851	Joint ankylosis of the shoulder region
N0852	Joint ankylosis of the upper arm
N0853	Joint ankylosis of the forearm
N0854	Joint ankylosis of the hand
N0855	Joint ankylosis of the pelvic region and thigh
N0856	Joint ankylosis of the lower leg
N0857	Joint ankylosis of the ankle and foot
N0858	Joint ankylosis of other specified site
N0859	Ankylosis of multiple joints
N085A	Ankylosis of the shoulder joint
N085B	Ankylosis of other joint of the shoulder girdle
N085C	Ankylosis of the elbow joint
N085D	Ankylosis of the superior radio-ulnar joint
N085E	Ankylosis of the inferior radio-ulnar joint
N085F	Ankylosis of the wrist joint
N085G	Ankylosis of the 1st carpometacarpal joint
N085H	Ankylosis of other carpometacarpal joint
N085J	Ankylosis of metacarpophalangeal joint
N085K	Ankylosis of proximal interphalangeal joint
N085L	Ankylosis of distal interphalangeal joint
N085M	Ankylosis of the hip joint
N085N	Ankylosis of other pelvic joint
N085P	Ankylosis of the knee joint
N085Q	Ankylosis of the ankle joint
N085R	Ankylosis of the subtalar joint
N085S	Ankylosis of other tarsal joint
N085T	Ankylosis of metatarsophalangeal joint
N085U	Ankylosis of toe joint
N085z	Ankylosis of joint NOS
N086.	Unspecified intrapelvic protrusio acetabuli
N0860	Protrusio acetabuli of unspecified site
N0861	Protrusio acetabuli of the pelvic region and thigh
N086z	Protrusio acetabuli NOS
N087.	Fibrocartilage lesion of joint
N0870	Bankart lesion
N0871	Reverse Bankart lesion
N0872	Glenoid labrum detachment
N0873	Glenoid labrum tear
N0874	Triangular fibrocartilage tear
N0875	Triangular fibrocartilage detachment
N0876	Acetabular labrum detachment
N0877	Acetabular labrum tear
N0878	Snapping shoulder
N08y.	Other joint derangement NEC
N08y0	Other joint derangement NEC, of unspecified site
N08y1	Other joint derangement NEC, of the shoulder region
N08y2	Other joint derangement NEC, of the upper arm

For peer review only

N08y3	Other joint derangement NEC, of the forearm
N08y4	Other joint derangement NEC, of the hand
N08y5	Other joint derangement NEC, of the pelvic region and thigh
N08y6	Other joint derangement NEC, of the lower leg
N08y7	Other joint derangement NEC, of the ankle and foot
N08y8	Other joint derangement NEC, of other specified site
N08y9	Other joint derangement NEC, of multiple sites
N08yA	Flail joint
N08yB	Shoulder joint unstable
N08yz	Other joint derangement NEC, NOS
N08z.	Joint derangement NOS
N08z0	Joint derangement NOS, of unspecified site
N08z1	Joint derangement NOS, of the shoulder region
N08z2	Joint derangement NOS, of the upper arm
N08z3	Joint derangement NOS, of the forearm
N08z4	Joint derangement NOS, of the hand
N08z5	Joint derangement NOS, of the pelvic region and thigh
N08z6	Joint derangement NOS, of the ankle and foot
N08z7	Joint derangement NOS, of other specified site
N08z8	Joint derangement NOS, of multiple sites
N08zz	Joint derangement NOS
N09..	Other and unspecified joint disorders
N090.	Effusion of joint
N0900	Joint effusion of unspecified site
N0901	Joint effusion of the shoulder region
N0902	Joint effusion of the upper arm
N0903	Joint effusion of the forearm
N0904	Joint effusion of the hand
N0905	Joint effusion of the pelvic region and thigh
N0906	Joint effusion of the lower leg
N0907	Joint effusion of the ankle and foot
N0908	Joint effusion of other specified site
N0909	Effusion of multiple joints
N090A	Effusion of shoulder
N090B	Effusion of sternoclavicular joint
N090C	Effusion of acromioclavicular joint
N090D	Effusion of elbow
N090E	Effusion of distal radio-ulnar joint
N090F	Effusion of wrist
N090G	Effusion of metacarpophalangeal joint
N090H	Effusion of proximal interphalangeal joint of finger
N090J	Effusion of distal interphalangeal joint of finger
N090K	Effusion of hip
N090L	Effusion of sacro-iliac joint
N090M	Effusion of knee
N090N	Effusion of tibio-fibular joint
N090P	Effusion of ankle
N090Q	Effusion of subtalar joint

For peer review only

N090R	Effusion of talonavicular joint
N090S	Effusion of other tarsal joint
N090T	Effusion of 1st metatarsophalangeal joint
N090U	Effusion of lesser metatarsophalangeal joint
N090V	Effusion of interphalangeal joint of toe
N090W	Intermittent hydrarthrosis
N090X	Chronic joint effusion
N090Y	Acute joint effusion
N090z	Effusion of joint NOS
N091.	Haemarthrosis
N0910	Haemarthrosis of unspecified site
N0911	Haemarthrosis of the shoulder region
N0912	Haemarthrosis of the upper arm
N0913	Haemarthrosis of the forearm
N0914	Haemarthrosis of the hand
N0915	Haemarthrosis of the pelvic region and thigh
N0916	Haemarthrosis of the lower leg
N0917	Haemarthrosis of the ankle and foot
N0918	Haemarthrosis of other specified site
N0919	Haemarthrosis of multiple joints
N091A	Haemarthrosis of shoulder
N091B	Haemarthrosis of sternoclavicular joint
N091C	Haemarthrosis of acromioclavicular joint
N091D	Haemarthrosis of elbow
N091E	Haemarthrosis of distal radio-ulnar joint
N091F	Haemarthrosis of wrist
N091G	Haemarthrosis of MCP joint
N091H	Haemarthrosis of PIP joint of finger
N091J	Haemarthrosis of DIP joint of finger
N091K	Haemarthrosis of hip
N091L	Haemarthrosis of sacro-iliac joint
N091M	Haemarthrosis of knee
N091N	Haemarthrosis of tibio-fibular joint
N091P	Haemarthrosis of ankle
N091Q	Haemarthrosis of subtalar joint
N091R	Haemarthrosis of talonavicular joint
N091S	Haemarthrosis of other tarsal joint
N091T	Haemarthrosis-1st metatarsophalangeal joint
N091U	Haemarthrosis of lesser metatarsophalangeal joint
N091V	Haemarthrosis of interphalangeal joint of toe
N091z	Haemarthrosis NOS
N092.	Villonodular synovitis
N0920	Villonodular synovitis of unspecified site
N0921	Villonodular synovitis of the shoulder region
N0922	Villonodular synovitis of the upper arm
N0923	Villonodular synovitis of the forearm
N0924	Villonodular synovitis of the hand
N0925	Villonodular synovitis of the pelvic region and thigh

For peer review only

N0926	Villonodular synovitis of the lower leg
N0927	Villonodular synovitis of the ankle and foot
N0928	Villonodular synovitis of other specified site
N0929	Villonodular synovitis of multiple sites
N092A	Villonodular synovitis of shoulder joint
N092B	Villonodular synovitis of sternoclavicular joint
N092C	Villonodular synovitis of acromioclavicular joint
N092D	Villonodular synovitis of elbow
N092E	Villonodular synovitis of distal radio-ulnar joint
N092F	Villonodular synovitis of wrist
N092G	Villonodular synovitis of metacarpophalangeal joint
N092H	Villonodular synovitis of proximal interphalangeal joint of finger
N092J	Villonodular synovitis of distal interphalangeal joint of finger
N092K	Villonodular synovitis of hip
N092L	Villonodular synovitis of sacro-iliac joint
N092M	Villonodular synovitis of knee
N092N	Villonodular synovitis of tibio-fibular joint
N092P	Villonodular synovitis of ankle
N092Q	Villonodular synovitis of subtalar joint
N092R	Villonodular synovitis of talonavicular joint
N092S	Villonodular synovitis of other tarsal joint
N092T	Villonodular synovitis of 1st metatarsophalangeal joint
N092U	Villonodular synovitis of lesser metatarsophalangeal joint
N092V	Villonodular synovitis of interphalangeal joint of toe
N092z	Villonodular synovitis NOS
N093.	Palindromic rheumatism
N0930	Palindromic rheumatism of unspecified site
N0931	Palindromic rheumatism of the shoulder region
N0932	Palindromic rheumatism of the upper arm
N0933	Palindromic rheumatism of the forearm
N0934	Palindromic rheumatism of the hand
N0935	Palindromic rheumatism of the pelvic region and thigh
N0936	Palindromic rheumatism of the lower leg
N0937	Palindromic rheumatism of the ankle and foot
N0938	Palindromic rheumatism of other specified site
N0939	Palindromic rheumatism of multiple sites
N093z	Palindromic rheumatism NOS
N094.	Pain in joint - arthralgia
N0940	Arthralgia of unspecified site
N0941	Arthralgia of the shoulder region
N0942	Arthralgia of the upper arm
N0943	Arthralgia of the forearm
N0944	Arthralgia of the hand
N0945	Arthralgia of the pelvic region and thigh
N0946	Arthralgia of the lower leg
N0947	Arthralgia of the ankle and foot
N0948	Arthralgia of other specified site
N0949	Arthralgia of multiple joints

For peer review only

N094A	Arthralgia of shoulder
N094B	Arthralgia of sternoclavicular joint
N094C	Arthralgia of acromioclavicular joint
N094D	Arthralgia of elbow
N094E	Arthralgia of distal radio-ulnar joint
N094F	Arthralgia of wrist
N094G	Arthralgia of metacarpophalangeal joint
N094H	Arthralgia of proximal interphalangeal joint of finger
N094J	Arthralgia of distal interphalangeal joint of finger
N094K	Arthralgia of hip
N094L	Arthralgia of sacro-iliac joint
N094M	Arthralgia of knee
N094N	Arthralgia of tibio-fibular joint
N094P	Arthralgia of ankle
N094Q	Arthralgia of subtalar joint
N094R	Arthralgia of talonavicular joint
N094S	Arthralgia of other tarsal joint
N094T	Arthralgia of 1st metatarsophalangeal joint
N094U	Arthralgia of lesser metatarsophalangeal joint
N094V	Arthralgia of interphalangeal joint of toe
N094W	Anterior knee pain
N094z	Arthralgia NOS
N095.	Joint stiffness NEC
N0950	Stiff joint NEC, of unspecified site
N0951	Stiff joint NEC, of the shoulder region
N0952	Stiff joint NEC, of the upper arm
N0953	Stiff joint NEC, of the forearm
N0954	Stiff joint NEC, of the hand
N0955	Stiff joint NEC, of the pelvic region and thigh
N0956	Stiff joint NEC, of the lower leg
N0957	Stiff joint NEC, of the ankle and foot
N0958	Stiff joint NEC, of other specified site
N0959	Multiple stiff joints
N095A	Stiff shoulder NEC
N095B	Stiff sternoclavicular joint NEC
N095C	Stiff acromioclavicular joint NEC
N095D	Stiff elbow NEC
N095E	Stiff distal radio-ulnar joint NEC
N095F	Stiff wrist NEC
N095G	Stiff metacarpophalangeal joint NEC
N095H	Stiff proximal interphalangeal joint of finger NEC
N095J	Stiff distal interphalangeal joint of finger NEC
N095K	Stiff hip NEC
N095L	Stiff sacro-iliac joint NEC
N095M	Stiff knee NEC
N095N	Stiff tibio-fibular joint NEC
N095P	Stiff ankle NEC
N095Q	Stiff subtalar joint NEC

For peer review only

N095R	Stiff talonavicular joint NEC
N095S	Stiff other tarsal joint NEC
N095T	Stiff 1st metatarsophalangeal joint NEC
N095U	Stiff lesser metatarsophalangeal joint NEC
N095V	Stiff interphalangeal joint of toe NEC
N095W	Stiff finger
N095z	Joint stiffness NEC, NOS
N096.	Other joint symptoms
N0960	Other joint symptoms of unspecified site
N0961	Other joint symptoms of the shoulder region
N0962	Other joint symptoms of the upper arm
N0963	Other joint symptoms of the forearm
N0964	Other joint symptoms of the hand
N0965	Other joint symptoms of the pelvic region and thigh
N0966	Other joint symptoms of the lower leg
N0967	Other joint symptoms of the ankle and foot
N0968	Other joint symptoms of other specified site
N0969	Other joint symptoms of multiple sites
N096A	Other symptoms - shoulder
N096B	Other symptoms in sternoclavicular joint
N096C	Other symptoms in acromioclavicular joint
N096D	Other symptoms - elbow
N096E	Other symptoms in distal radio-ulnar joint
N096F	Other symptoms - wrist
N096G	Other symptoms - MCPJ
N096H	Other symptoms in proximal interphalangeal joint of finger
N096J	Other symptoms in distal interphalangeal joint of finger
N096K	Other symptoms - hip
N096L	Other symptoms in sacro-iliac joint
N096M	Other symptoms - knee
N096N	Other symptoms in tibio-fibular joint
N096P	Other symptoms - ankle
N096Q	Other symptoms in subtalar joint
N096R	Other symptoms in talonavicular joint
N096S	Other symptoms in other tarsal joint
N096T	Other symptoms in 1st metatarsophalangeal joint
N096U	Other symptoms in lesser metatarsophalangeal joint
N096V	Other symptoms in interphalangeal joint of toe
N096z	Other joint symptoms NOS
N097.	Difficulty in walking
N0970	Walking difficulty due to unspecified site
N0971	Walking difficulty due to pelvic region and thigh
N0972	Walking difficulty due to lower leg
N0973	Walking difficulty due to ankle and foot
N0974	Walking difficulty due to other specified site
N0975	Walking difficulty due to multiple sites
N097z	Difficulty in walking NOS
N098.	Synovial osteochondromatosis

For peer review only

N0980	Synovial osteochondromatosis of shoulder
N0981	Synovial osteochondromatosis of sternoclavicular joint
N0982	Synovial osteochondromatosis of acromioclavicular joint
N0983	Synovial osteochondromatosis of elbow
N0984	Synovial osteochondromatosis of distal radio-ulnar joint
N0985	Synovial osteochondromatosis of wrist
N0986	Synovial osteochondromatosis of metacarpophalangeal joint
N0987	Synovial osteochondromatosis of proximal interphalangeal joint of finger
N0988	Synovial osteochondromatosis of distal interphalangeal joint of finger
N0989	Synovial osteochondromatosis of hip
N098A	Synovial osteochondromatosis of sacro-iliac joint
N098B	Synovial osteochondromatosis of knee
N098C	Synovial osteochondromatosis of tibio-fibular joint
N098D	Synovial osteochondromatosis of ankle
N098E	Synovial osteochondromatosis of subtalar joint
N098F	Synovial osteochondromatosis of talonavicular joint
N098G	Synovial osteochondromatosis of other tarsal joint
N098H	Synovial osteochondromatosis of 1st metatarsophalangeal joint
N098J	Synovial osteochondromatosis of lesser metatarsophalangeal joint
N098K	Synovial osteochondromatosis of interphalangeal joint of toe
N099.	Clicking joint
N0990	Clicking shoulder
N0991	Clicking sternoclavicular joint
N0992	Clicking acromioclavicular joint
N0993	Clicking elbow
N0994	Clicking distal radio-ulnar joint
N0995	Clicking wrist
N0996	Clicking metacarpophalangeal joint
N0997	Clicking proximal interphalangeal joint of finger
N0998	Clicking distal interphalangeal joint of finger
N0999	Clicking hip
N099A	Multiple clicking joints
N099B	Clicking sacro-iliac joint
N099C	Clicking knee
N099D	Clicking tibio-fibular joint
N099E	Clicking ankle
N099F	Clicking subtalar joint
N099G	Clicking talonavicular joint
N099H	Clicking other tarsal joint
N099J	Clicking 1st metatarsophalangeal joint
N099K	Clicking lesser metatarsophalangeal joint
N099L	Clicking interphalangeal joint of toe
N09A.	Patellofemoral disorder
N09AX	Disorder of patella, unspecified
N09B.	Osteophyte
N09C.	Fistula of joint
N09y.	Other specified joint disorders
N09y0	Other specified joint disorders of unspecified site

For peer review only

N09y1	Other specified joint disorders of the shoulder region
N09y2	Other specified joint disorders of the upper arm
N09y3	Other specified joint disorders of the forearm
N09y4	Other specified joint disorders of the hand
N09y5	Other specified joint disorders of the pelvic region and thigh
N09y6	Other specified joint disorders of the lower leg
N09y7	Other specified joint disorders of the ankle and foot
N09y8	Other specified joint disorders of other specified site
N09y9	Other specified joint disorders of multiple sites
N09yz	Other specified joint disorders NOS
N09z.	Joint disorders NOS
N09z0	Joint disorder NOS, of unspecified site
N09z1	Joint disorder NOS, of shoulder region
N09z2	Joint disorder NOS, of the upper arm
N09z3	Joint disorder NOS, of the forearm
N09z4	Joint disorder NOS, of the hand
N09z5	Joint disorder NOS, of the pelvic region and thigh
N09z6	Joint disorder NOS, of the lower leg
N09z7	Joint disorder NOS, of ankle and foot
N09z8	Joint disorder NOS, of other specified site
N09z9	Joint disorder NOS, of multiple sites
N09zz	Joint disorders NOS
N0y..	Other specified arthropathies
N0z..	Arthropathies NOS
N1...	Vertebral column syndromes
N10..	Inflammatory spondylopathies
N100.	Ankylosing spondylitis
N101.	Spinal enthesopathy
N102.	Sacroiliitis NEC
N10y.	Other inflammatory spondylopathies
N10y0	Inflammatory spondylopathies in diseases EC
N10yz	Other inflammatory spondylopathies NOS
N10z.	Spondylitis NOS
N11..	Spondylosis and allied disorders
N110.	Cervical spondylosis without myelopathy
N1100	Single-level cervical spondylosis without myelopathy
N1101	Two-level cervical spondylosis without myelopathy
N1102	Multiple-level cervical spondylosis without myelopathy
N111.	Cervical spondylosis with myelopathy
N1110	Single-level cervical spondylosis with myelopathy
N1111	Two-level cervical spondylosis with myelopathy
N1112	Multiple-level cervical spondylosis with myelopathy
N1113	Cervical myelopathy
N112.	Thoracic spondylosis without myelopathy
N1120	Single-level thoracic spondylosis without myelopathy
N1121	Two-level thoracic spondylosis without myelopathy
N1122	Multiple-level thoracic spondylosis without myelopathy
N1123	Dorsal spondylosis without myelopathy

For peer review only

N113.	Thoracic spondylosis with myelopathy
N1130	Single-level thoracic spondylosis with myelopathy
N1131	Two-level thoracic spondylosis with myelopathy
N1132	Multiple-level thoracic spondylosis with myelopathy
N114.	Lumbosacral spondylosis without myelopathy
N1140	Single-level lumbosacral spondylosis without myelopathy
N1141	Two-level lumbosacral spondylosis without myelopathy
N1142	Multiple-level lumbosacral spondylosis without myelopathy
N115.	Lumbosacral spondylosis with myelopathy
N1150	Single-level lumbosacral spondylosis with myelopathy
N1151	Two-level lumbosacral spondylosis with myelopathy
N1152	Multiple-level lumbosacral spondylosis with myelopathy
N116.	Kissing spine
N117.	Ankylosing vertebral hyperostosis
N118.	Traumatic spondylopathy
N119.	Cervical spondylosis with radiculopathy
N1190	Single-level cervical spondylosis with radiculopathy
N1191	Two-level cervical spondylosis with radiculopathy
N1192	Multiple-level cervical spondylosis with radiculopathy
N11A.	Cervical spondylosis with vascular compression
N11B.	Thoracic spondylosis with radiculopathy
N11B0	Single-level thoracic spondylosis with radiculopathy
N11B1	Two-level thoracic spondylosis with radiculopathy
N11B2	Multiple-level thoracic spondylosis with radiculopathy
N11C.	Lumbosacral spondylosis with radiculopathy
N11C0	Single-level lumbosacral spondylosis with radiculopathy
N11C1	Two-level lumbosacral spondylosis with radiculopathy
N11C2	Multiple-level lumbosacral spondylosis with radiculopathy
N11D.	Osteoarthritis of spine
N11D0	Osteoarthritis of cervical spine
N11D1	Osteoarthritis of thoracic spine
N11D2	Osteoarthritis of lumbar spine
N11D3	Osteoarthritis of spine NOS
N11E.	Cervical spondylosis
N11F.	Axial spondyloarthritis
N11y.	Other spondyloses and allied disorders
N11y0	Brucella spondylitis
N11y1	Enterobacterial spondylitis
N11y2	Neuropathic spondylopathy
N11z.	Spondylosis NOS
N11z0	Spondylosis without myelopathy, NOS
N11z1	Spondylosis with myelopathy, NOS
N11zz	Spondylosis NOS
N12..	Intervertebral disc disorders
N120.	Cervical disc displacement without myelopathy
N121.	Thoracic disc displacement without myelopathy
N122.	Lumbar disc displacement
N123.	Disc displacement, site unspecified, without myelopathy

For peer review only

N124.	Schmorl's nodes
N1240	Schmorl's nodes of unspecified region
N1241	Schmorl's nodes of the thoracic region
N1242	Schmorl's nodes of the lumbar region
N124z	Schmorl's nodes, region NOS
N125.	Cervical disc degeneration
N126.	Thoracic disc degeneration
N127.	Lumbar disc degeneration
N128.	Degenerative disc disease NOS
N129.	Disc disorder with myelopathy
N1290	Unspecified disc disorder with myelopathy
N1291	Cervical disc disorder with myelopathy
N1292	Thoracic disc disorder with myelopathy
N1293	Lumbar disc disorder with myelopathy
N129z	Disc disorder with myelopathy NOS
N12A.	Postlaminectomy syndrome
N12A0	Postlaminectomy syndrome of unspecified site
N12A1	Cervical postlaminectomy syndrome
N12A2	Thoracic postlaminectomy syndrome
N12A3	Lumbar postlaminectomy syndrome
N12Az	Postlaminectomy syndrome NOS
N12B.	Disc prolapse with myelopathy
N12B0	Cervical disc prolapse with myelopathy
N12B1	Thoracic disc prolapse with myelopathy
N12B2	Lumbar disc prolapse with myelopathy
N12C.	Disc prolapse with radiculopathy
N12C0	Cervical disc prolapse with radiculopathy
N12C1	Thoracic disc prolapse with radiculopathy
N12C2	Lumbar disc prolapse with radiculopathy
N12C3	Lumbar disc prolapse with cauda equina compression
N12C4	Prolapsed lumbar intervertebral disc with sciatica
N12D.	Narrowing intervertebral disc space
N12z.	Other and unspecified disc disorders
N12z0	Other disc disorders of unspecified site
N12z1	Other cervical disc disorders
N12z2	Other thoracic disc disorders
N12z3	Other lumbar disc disorders
N12z4	Cervical discitis
N12z5	Annular tear of cervical disc
N12z6	Resorption of cervical disc
N12z7	Calcification of cervical disc
N12z8	Thoracic discitis
N12z9	Annular tear of thoracic disc
N12zA	Resorption of thoracic disc
N12zB	Calcification of thoracic disc
N12zC	Lumbar discitis
N12zD	Annular tear of lumbar disc
N12zE	Resorption of lumbar disc

For peer review only

N12zF	Calcification of lumbar disc
N12zG	Infection of intervertebral disc - pyogenic
N12zH	Cervical disc disorder with radiculopathy
N12zz	Disc disorders NOS
N13..	Other cervical disorders
N130.	Cervical spinal stenosis
N1300	Idiopathic cervical spinal stenosis
N1301	Degenerative cervical spinal stenosis
N1302	Iatrogenic cervical spinal stenosis
N1303	Cervical spinal stenosis secondary to other disease
N131.	Cervicalgia - pain in neck
N132.	Cervicocranial syndrome
N133.	Cervicobrachial syndrome
N134.	Brachial (cervical) neuritis
N135.	Torticollis unspecified
N1350	Intermittent torticollis
N1351	Rheumatic torticollis
N135z	Torticollis NOS
N136.	Panniculitis of neck
N137.	Cervical posterior longitudinal ligament ossification
N138.	Cervicalgia
N13y.	Other cervical syndromes
N13y0	Cervical syndrome NEC
N13y1	Klippel's disease
N13y2	Crick in neck
N13y3	Cervical root syndrome
N13yz	Other cervical syndromes NOS
N13z.	Cervical and neck disorders NOS
N14..	Other and unspecified back disorders
N140.	Spinal stenosis, excluding cervical region
N1400	Spinal stenosis of unspecified region
N1401	Thoracic spinal stenosis
N1402	Lumbar spinal stenosis
N1403	Idiopathic thoracic spinal stenosis
N1404	Degenerative thoracic spinal stenosis
N1405	Iatrogenic thoracic spinal stenosis
N1406	Thoracic spinal stenosis secondary to other disease
N1407	Idiopathic lumbar spinal stenosis
N1408	Degenerative lumbar spinal stenosis
N1409	Iatrogenic lumbar spinal stenosis
N140A	Lumbar spinal stenosis secondary to other disease
N140z	Spinal stenosis NOS
N141.	Pain in thoracic spine
N142.	Pain in lumbar spine
N1420	Lumbago with sciatica
N143.	Sciatica
N144.	Thoracic and lumbosacral neuritis
N1440	Thoracic neuritis, unspecified

For peer review only

N1441	Lumbosacral neuritis, unspecified
N144z	Thoracic and lumbosacral neuritis NOS
N145.	Backache, unspecified
N146.	Disorders of the sacrum
N1460	Lumbosacral ankylosis
N1461	Sacroiliac ankylosis
N1462	Sacral ankylosis NOS
N1463	Lumbosacral instability
N1464	Sacroiliac instability
N1465	Sacral instability NOS
N1466	Sacroiliac disorder
N146z	Disorders of the sacrum NOS
N147.	Disorders of the coccyx
N1470	Unspecified disorder of the coccyx
N1471	Hypermobility of the coccyx
N1472	Coccygodynia
N147z	Coccyx disorder NOS
N148.	Ankylosis or instability of cervical,thoracic or lumbar spine
N1480	Atlanto-occipital ankylosis
N1481	Atlanto-axial ankylosis
N1482	Cervical spine ankylosis
N1483	Cervico-thoracic ankylosis
N1484	Thoracic spine ankylosis
N1485	Thoraco-lumbar ankylosis
N1486	Lumbar spine ankylosis
N1487	Atlanto-occipital instability
N1488	Atlanto-axial instability
N1489	Cervical spine instability
N148A	Cervico-thoracic instability
N148B	Thoracic spine instability
N148C	Lumbar spine instability
N149.	Back stiffness
N14A.	Neurogenic claudication
N14X.	Sacrococcygeal disorders, not elsewhere classified
N14y.	Other back symptoms
N14z.	Back disorders NOS
N1y..	Other specified disorders of vertebral column
N1y0.	Recurrent atlantoaxial subluxation with myelopathy
N1y1.	Fatigue fracture of vertebra
N1y2.	Pars interarticularis stress fracture
N1z..	Vertebral column disorder NOS
N2...	Rheumatism, excluding the back
N20..	Polymyalgia rheumatica
N200.	Giant cell arteritis with polymyalgia rheumatica
N21..	Peripheral enthesopathies and allied syndromes
N210.	Adhesive capsulitis of the shoulder
N211.	Rotator cuff shoulder syndrome and allied disorders
N2110	Rotator cuff syndrome, unspecified

For peer review only

N2111	Calcifying tendinitis of the shoulder
N2112	Bicipital tenosynovitis
N2113	Supraspinatus tendinitis
N2114	Partial thickness rotator cuff tear
N2115	Full thickness rotator cuff tear
N2116	Subacromial bursitis
N2117	Subdeltoid bursitis
N2118	Bursitis of shoulder
N211z	Rotator cuff syndrome NOS
N212.	Other shoulder affections NEC
N2120	Periarthritis of shoulder
N2121	Scapulohumeral fibrositis
N2122	Subacromial impingement
N2123	Coracoid impingement
N2124	Impingement syndrome of shoulder
N2125	Shoulder tendonitis
N212z	Other shoulder affections NEC, NOS
N213.	Enthesopathy of the elbow region
N2130	Elbow enthesopathy unspecified
N2131	Medial epicondylitis of the elbow
N2132	Lateral epicondylitis of the elbow
N2133	Olecranon bursitis
N2134	Biceps tendinitis
N2135	Triceps tendinitis
N213z	Elbow enthesopathy NOS
N214.	Enthesopathy of the wrist and carpus
N2140	Bursitis of wrist
N2141	Bursitis of hand
N2142	Periarthritis of wrist
N2143	Carpometacarpal bossing
N214z	Wrist or carpus enthesopathy NOS
N215.	Enthesopathy of the hip region
N2150	Hip enthesopathy, unspecified
N2151	Bursitis of hip
N2152	Gluteal tendinitis
N2153	Iliac crest spur
N2154	Psoas tendinitis
N2155	Trochanteric tendinitis
N2156	Adductor tendinitis
N2157	Trochanteric bursitis
N2158	Snapping hip
N2159	Iliotibial band syndrome
N215A	Ischial bursitis
N215B	Femoroacetabular impingement
N215z	Hip enthesopathy NOS
N216.	Enthesopathy of the knee
N2160	Bursitis of the knee NOS
N2161	Pes anserinus tendinitis and bursitis

For peer review only

N2162	Tibial collateral ligament bursitis
N2163	Fibular collateral ligament bursitis
N2164	Patellar tendinitis
N2165	Prepatellar bursitis
N2166	Infrapatellar bursitis
N2167	Subpatellar bursitis
N2168	Biceps femoris tendinitis
N2169	Semimembranosus tendinitis
N216z	Knee enthesopathy NOS
N217.	Enthesopathy of the ankle and tarsus
N2170	Enthesopathy of the ankle unspecified
N2171	Enthesopathy of the tarsus unspecified
N2172	Metatarsalgia NOS
N2173	Achilles bursitis
N2174	Achilles tendinitis
N2175	Tibialis anterior tendinitis
N2176	Tibialis posterior tendinitis
N2177	Calcaneal spur
N2178	Peroneal tendinitis
N2179	Plantar fasciitis
N217A	Posterior calcaneal exostosis (pump bump)
N217B	Anterior ankle impingement
N217C	Fibular impingement
N217z	Ankle or tarsus enthesopathy NOS
N21y.	Other peripheral enthesopathies
N21y0	Anterior shin splints
N21y1	Posterior shin splints
N21z.	Enthesopathy NOS
N21z0	Capsulitis NOS
N21z1	Periarthritis NOS
N21z2	Tendinitis NOS
N21z3	Exostosis of unspecified site
N21z4	Subungual exostosis
N21z5	Subungual exostosis of great toe
N21z6	Subungual exostosis of lesser toe
N21z7	Exostosis
N21zz	Peripheral enthesopathy NOS
N22..	Other disorders of the synovium, tendon and bursa
N220.	Synovitis and tenosynovitis
N2200	Synovitis or tenosynovitis NOS
N2201	Synovitis and tenosynovitis with disorders EC
N2202	Tendon sheath giant cell tumor
N2203	Trigger finger - acquired
N2204	Radial styloid tenosynovitis
N2205	Other tenosynovitis of hand or wrist
N2206	Tenosynovitis of ankle
N2207	Tenosynovitis of foot
N2208	Villonodular synovitis of tendon sheath

For peer review only

N2209	Plant thorn synovitis
N220A	Flexor tenosynovitis of wrist
N220B	Flexor tenosynovitis of finger
N220C	Flexor tenosynovitis of thumb
N220D	Extensor tenosynovitis of wrist
N220E	Extensor tenosynovitis of finger
N220F	Extensor tenosynovitis of thumb
N220G	Acquired trigger thumb
N220H	Achilles tenosynovitis
N220J	Tibialis anterior tenosynovitis
N220K	Tibialis posterior tenosynovitis
N220L	Extensor hallucis longus tenosynovitis
N220M	Extensor digitorum longus tenosynovitis
N220N	Peroneus longus tenosynovitis
N220P	Peroneus brevis tenosynovitis
N220Q	Transient synovitis
N220R	Chronic crepitant synovitis of hand and wrist
N220S	Synovitis of hip
N220T	Synovitis NOS
N220V	Synovitis of knee
N220W	Synovitis of elbow
N220X	Synovitis of shoulder
N220Y	Irritable hip
N220z	Other synovitis and tenosynovitis
N221.	Bunion
N2210	Infected bunion
N222.	Specific bursitides
N2220	Beat elbow
N2221	Beat hand
N2222	Beat knee
N2223	Miners' elbow
N2224	Miners' knee
N2225	Housemaids' knee
N2226	Calcium deposit in bursa
N2227	Syphilitic bursitis
N222z	Specific bursitides NOS
N223.	Bursitis NOS
N224.	Ganglion and cyst of synovium, tendon and bursa
N2240	Synovial cyst unspecified
N2241	Ganglion of joint
N2242	Ganglion of tendon sheath
N2243	Ganglion unspecified
N2244	Cyst of bursa
N2245	Ganglion of wrist
N2246	Ganglion of knee
N2247	Ganglion of superior tibio-fibular joint
N2248	Ganglion of ankle
N2249	Ganglion of flexor tendon sheath of finger

For peer review only

N224A	Synovial cyst of popliteal space
N224B	Ganglion of hand
N224C	Ganglion of foot
N224D	Cyst of tendon sheath
N224E	Digital mucous cyst
N224z	Ganglion or cyst of synovium/tendon/bursa NOS
N225.	Rupture of synovium
N2250	Rupture of synovium, unspecified
N2251	Rupture of popliteal space synovial cyst
N225z	Rupture of synovium NOS
N226.	Nontraumatic tendon rupture
N2260	Nontraumatic tendon rupture, unspecified
N2261	Rotator cuff complete rupture
N2262	Biceps tendon rupture
N2263	Hand and wrist extensor tendon rupture
N2264	Hand and wrist flexor tendon rupture
N2265	Quadriceps tendon rupture
N2266	Nontraumatic rupture of patellar tendon
N2267	Nontraumatic rupture of Achilles tendon
N2268	Extensor digitorum communis rupture
N2269	Extensor pollicis longus rupture
N226A	Long head of biceps rupture
N226B	Subluxation of long head of biceps
N226C	Flexor digitorum sublimis tendon rupture
N226D	Flexor digitorum profundus tendon rupture
N226E	Flexor pollicis longus tendon rupture
N226F	Tibialis posterior rupture
N226G	Peroneus longus rupture
N226H	Subluxation of peroneal tendon
N226J	Subluxation of tendon, wrist or hand
N226K	Dislocation of tendon, wrist or hand
N226L	Bowstringing of tendon, wrist or hand
N226M	Spontaneous rupture of flexor tendons
N226N	Spontaneous rupture of extensor tendons
N226y	Other foot and ankle tendon rupture
N226z	Other nontraumatic tendon rupture
N227.	Posterior tibial tendon insufficiency
N228.	Tendinous xanthoma
N22y.	Other synovial, tendon and bursa disorders
N22y0	Contracture of tendon sheath
N22y1	Calcification of tendon NOS
N22y2	Abscess of tendon
N22y3	Abscess of bursa
N22y4	Synovial plica
N22y5	Short tendon
N22y6	Abscess of tendon-arm
N22y7	Abscess of tendon-forearm
N22y8	Abscess of tendon-hand

For peer review only

N22y9	Abscess of tendon-thigh
N22yA	Abscess of tendon-leg
N22yB	Abscess of tendon-foot
N22yC	Pyogenic infection of tendon sheath
N22yD	Tuberculous infection of tendon sheath
N22yE	Abscess of bursa-shoulder
N22yF	Abscess of bursa-elbow
N22yG	Abscess of bursa-wrist
N22yH	Abscess of bursa-hip
N22yJ	Abscess of bursa-knee
N22yK	Abscess of bursa-ankle
N22yL	Abscess of bursa-foot
N22yM	Short Achilles tendon - acquired
N22yN	Achilles degeneration
N22yz	Other synovial, tendon or bursa disorder NOS
N22z.	Synovial, tendon or bursa disorder NOS
N23..	Muscle, ligament and fascia disorders
N230.	Infective myositis
N2300	Infective myositis-neck
N2301	Infective myositis-back
N2302	Infective myositis-shoulder
N2303	Infective myositis-arm
N2304	Infective myositis-forearm
N2305	Infective myositis-hand
N2306	Infective myositis-pelvis
N2307	Infective myositis-thigh
N2308	Infective myositis-leg
N2309	Infective myositis-foot
N230A	Muscle abscess
N230B	Muscle abscess-neck
N230C	Muscle abscess-back
N230D	Muscle abscess-shoulder
N230E	Muscle abscess-arm
N230F	Muscle abscess-forearm
N230G	Muscle abscess-hand
N230H	Muscle abscess-pelvis
N230J	Muscle abscess-thigh
N230K	Muscle abscess-leg
N230L	Muscle abscess-foot
N231.	Muscle calcification and ossification
N2310	Muscular calcification and ossification, unspecified
N2311	Progressive myositis ossificans
N2312	Traumatic myositis ossificans
N2313	Post-operative heterotopic calcification
N2314	Polymyositis ossificans
N2315	Calcification and ossification of muscles associated with burns
N2316	Paralytic calcification and ossification of muscle
N231z	Muscle calcification or ossification NOS

For peer review only

N232.	Muscle wasting and disuse atrophy NEC
N2320	Amyotrophia NOS
N2321	Myofibrosis
N2322	Muscle wasting NEC
N232z	Muscle wasting/disuse atrophy NEC NOS
N233.	Other specific muscle disorder
N2330	Arthrogryposis
N2331	Immobility syndrome
N2332	Myositis in sarcoidosis
N2333	Rhabdomyolysis
N2334	Antisynthetase syndrome
N233z	Other specific muscle disorder NOS
N234.	Laxity of ligament
N235.	Hypermobility syndrome
N236.	Dupuytren's contracture
N2360	Dupuytren's disease of palm
N2361	Dupuytren's disease of palm, nodules with no contracture
N2362	Dupuytren's disease of palm, with contracture
N2363	Dupuytren's disease of finger(s)
N2364	Dupuytren's disease of finger(s), nodules with no contracture
N2365	Dupuytren's disease of finger(s), with contracture
N2366	Dupuytren's disease of palm and finger(s)
N2367	Dupuytren's disease of palm and finger(s), nodules with no contracture
N2368	Dupuytren's disease of palm and finger(s), with contracture
N237.	Other fibromatoses
N2370	Plantar fascial fibromatosis
N2371	Knuckle pads
N2372	Nodular fasciitis
N2373	Pseudosarcomatous fibromatosis
N237z	Other fibromatoses NOS
N238.	Muscle contracture
N2380	Contracture of pectoralis major
N2381	Contracture of triceps
N2382	Contracture of biceps
N2383	Contracture of wrist flexor(s)
N2384	Contracture of wrist extensor(s)
N2385	Contracture of flexor pollicis longus
N2386	Contracture of thumb extensor(s)
N2387	Contracture of flexor digitorum superficialis
N2388	Contracture of flexor digitorum profundus
N2389	Contracture of adductor pollicis
N238A	Contracture of other intrinsic muscle(s) of hand
N238B	Contracture of iliopsoas
N238C	Contracture of rectus femoris
N238D	Contracture of adductor muscle(s) of hip
N238E	Contracture of abductor muscle(s) of hip
N238F	Contracture of hamstring(s)
N238G	Contracture of quadriceps

For peer review only

N238H	Contracture of tendo achilles
N238J	Contracture of tibialis anterior
N238K	Contracture of tibialis posterior
N238L	Contracture of long toe flexor(s)
N238M	Contracture of long toe extensor(s)
N238N	Contracture of intrinsic muscle(s) of foot
N239.	Fibromyalgia
N23y.	Other muscle, ligament and fascia disorder
N23y0	Interstitial myositis
N23y1	Foreign body muscle granuloma
N23y2	Nontraumatic muscle rupture
N23y3	Diastasis recti abdominis
N23y4	Spasm of muscle
N23y5	Inappropriate firing of muscle
N23y6	Palmar space infection, thenar
N23y7	Palmar space infection, mid-palm
N23y8	Palmar space infection, hypo-thenar
N23y9	Calcific tendinitis
N23yA	Diastasis of muscle
N23yB	Ischaemic infarction of muscle
N23yC	Contracture of muscle
N23yD	Muscle strain
N23yE	Spasm of back muscles
N23yF	Muscle crush syndrome
N23yz	Other muscle, ligament or fascia disorder NOS
N23z.	Muscle, ligament or fascia disorder NOS
N24..	Other soft tissue disorders
N240.	Rheumatism and fibrositis unspecified
N2400	Rheumatism unspecified
N2401	Fibrositis unspecified
N2402	Muscular rheumatism
N2403	Rheumatic pain
N2405	Fibrositis of neck
N2406	Fibrositis arm
N2407	Hand rheumatism
N240z	Rheumatism or fibrositis NOS
N241.	Myalgia and myositis unspecified
N2410	Myalgia unspecified
N2411	Myositis unspecified
N2412	Fibromyositis NOS
N2413	Viral myalgia
N241z	Myalgia or myositis NOS
N242.	Neuralgia, neuritis and radiculitis unspecified
N2420	Neuralgia unspecified
N2421	Neuritis unspecified
N2422	Radiculitis unspecified
N2423	Neuropathic pain
N242z	Neuralgia, neuritis or radiculitis NOS

For peer review only

N243.	Panniculitis unspecified
N2430	Panniculitis of unspecified site
N2431	Hypertrophy of the knee fat pad
N2432	Weber - Christian disease
N243z	Panniculitis NOS
N244.	Fasciitis unspecified
N245.	Pain in limb
N2450	Hand pain
N2451	Foot pain
N2452	Pain in leg
N2453	Pain in arm
N2454	Calf pain
N2455	Axillary pain
N2456	Tender heel pad
N2457	Shoulder pain
N246.	Residual foreign body in soft tissue
N247.	Other musculoskeletal limb symptoms
N2470	Swelling of limb
N2471	Leg cramps
N2472	Cramp
N247z	Musculoskeletal limb symptoms NOS
N248.	Fibromyalgia
N2480	Myofascial pain syndrome
N2481	Piriformis syndrome
N24z.	Soft tissue disorders NOS
N25..	SAPHO syndrome (Synovitis Acne Pustulosis Hyperostosis Osteomyelitis)
N2y..	Other specified nonarticular rheumatism
N2z..	Nonarticular rheumatism NOS
N3...	Osteopathies, chondropathies and acquired musculoskeletal deformities
N30..	Osteomyelitis, periostitis and other infections affecting bone
N300.	Acute osteomyelitis
N3000	Acute osteomyelitis of unspecified site
N3001	Acute osteomyelitis of the shoulder region
N3002	Acute osteomyelitis of the upper arm
N3003	Acute osteomyelitis of the forearm
N3004	Acute osteomyelitis of the hand
N3005	Acute osteomyelitis of the pelvic region and thigh
N3006	Acute osteomyelitis of the lower leg
N3007	Acute osteomyelitis of the ankle and foot
N3008	Acute osteomyelitis of other specified site
N3009	Acute osteomyelitis of multiple sites
N300A	Acute osteomyelitis-cervical spine
N300B	Acute osteomyelitis-thoracic spine
N300C	Acute osteomyelitis-lumbar spine
N300D	Acute osteomyelitis-sacrum
N300E	Acute osteomyelitis-coccyx
N300F	Acute osteomyelitis-clavicle
N300G	Acute osteomyelitis-scapula

For peer review only

N300H	Acute osteomyelitis-humerus
N300J	Acute osteomyelitis-radius
N300K	Acute osteomyelitis-ulna
N300L	Acute osteomyelitis-carpal bone
N300M	Acute osteomyelitis-metacarpal
N300N	Acute osteomyelitis-phalanx of finger/thumb
N300P	Acute osteomyelitis-pelvis
N300Q	Acute osteomyelitis-femur
N300R	Acute osteomyelitis-patella
N300S	Acute osteomyelitis-tibia
N300T	Acute osteomyelitis-fibula
N300U	Acute osteomyelitis-calcaneum
N300V	Acute osteomyelitis-talus
N300W	Acute osteomyelitis-other tarsal bone
N300X	Acute osteomyelitis-metatarsal
N300Y	Acute osteomyelitis-phalanx of toe
N300Z	Acute haematogenous osteomyelitis
N300z	Acute osteomyelitis NOS
N301.	Chronic osteomyelitis
N3010	Chronic osteomyelitis of unspecified site
N3011	Chronic osteomyelitis of the shoulder region
N3012	Chronic osteomyelitis of the upper arm
N3013	Chronic osteomyelitis of the forearm
N3014	Chronic osteomyelitis of the hand
N3015	Chronic osteomyelitis of the pelvic region and thigh
N3016	Chronic osteomyelitis of the lower leg
N3017	Chronic osteomyelitis of the ankle and foot
N3018	Chronic osteomyelitis of other specified site
N3019	Chronic osteomyelitis of multiple sites
N301A	Chronic osteomyelitis-cervical spine
N301B	Chronic osteomyelitis-thoracic spine
N301C	Chronic osteomyelitis-lumbar spine
N301D	Chronic osteomyelitis-sacrum
N301E	Chronic osteomyelitis-coccyx
N301F	Brodie's abscess-cervical spine
N301G	Brodie's abscess-thoracic spine
N301H	Brodie's abscess-lumbar spine
N301J	Brodie's abscess-sacrum
N301K	Brodie's abscess-coccyx
N301L	Chronic multifocal osteomyelitis
N301M	Chronic osteomyelitis with draining sinus
N301z	Chronic osteomyelitis NOS
N302.	Unspecified osteomyelitis
N3020	Unspecified osteomyelitis of unspecified site
N3021	Unspecified osteomyelitis of the shoulder region
N3022	Unspecified osteomyelitis of the upper arm
N3023	Unspecified osteomyelitis of the forearm
N3024	Unspecified osteomyelitis of the hand

For peer review only

N3025	Unspecified osteomyelitis of the pelvic region and thigh
N3026	Unspecified osteomyelitis of the lower leg
N3027	Unspecified osteomyelitis of the ankle and foot
N3028	Unspecified osteomyelitis of other specified site
N3029	Unspecified osteomyelitis of multiple sites
N302A	Infection of cervical spine
N302B	Infection of thoracic spine
N302C	Infection of lumbar spine
N302D	Infection of sacrum
N302E	Infection of coccyx
N302F	Infection of clavicle
N302G	Infection of scapula
N302H	Infection of humerus
N302J	Infection of radius
N302K	Infection of ulna
N302L	Infection of carpal bone
N302M	Infection of metacarpal
N302N	Infection of phalanx of finger or thumb
N302P	Infection of pelvis
N302Q	Infection of femur
N302R	Infection of patella
N302S	Infection of tibia
N302T	Infection of fibula
N302U	Infection of calcaneum
N302V	Infection of talus
N302W	Infection of other tarsal bone
N302X	Infection of metatarsal
N302Y	Infection of phalanx of toe
N302Z	Infection of multiple bones
N302a	Osteomyelitis of vertebra
N302b	Brodie's abscess
N302z	Unspecified osteomyelitis NOS
N303.	Periostitis without osteomyelitis
N3030	Periostitis without mention of osteomyelitis, of unspecified site
N3031	Periostitis without mention of osteomyelitis, of the shoulder region
N3032	Periostitis without mention of osteomyelitis, of the upper arm
N3033	Periostitis without osteomyelitis, of the forearm
N3034	Periostitis without osteomyelitis, of the hand
N3035	Periostitis without osteomyelitis, of the pelvic region and thigh
N3036	Periostitis without mention of osteomyelitis, of the lower leg
N3037	Periostitis without mention of osteomyelitis, of the ankle and foot
N3038	Periostitis without mention of osteomyelitis, of other specified site
N3039	Periostitis without mention of osteomyelitis, of multiple sites
N303A	Periostitis without osteomyelitis-cervical spine
N303B	Periostitis without osteomyelitis-thoracic spine
N303C	Periostitis without osteomyelitis-lumbar spine
N303D	Periostitis without osteomyelitis-sacrum
N303E	Periostitis without osteomyelitis-coccyx

For peer review only

N303z	Periostitis without osteomyelitis NOS
N304.	Tuberculosis of spine (Pott's)
N3040	Tuberculosis of cervical spine
N3041	Tuberculosis of thoracic spine
N3042	Tuberculosis of lumbar spine
N3043	Tuberculosis of sacrum/coccyx
N305.	Tuberculosis of limb bones
N3050	Tuberculosis of unspecified limb bone
N3051	Tuberculosis of the upper arm bone
N3052	Tuberculosis of the forearm bone
N3053	Tuberculosis of the pelvic and thigh bones
N3054	Tuberculosis of the lower leg bone
N3055	Tuberculosis of other limb bones
N3056	Tuberculosis of multiple limb bones
N305z	Tuberculosis of limb bones NOS
N306.	Tuberculosis of other bones
N3060	Tuberculosis of bone, site unspecified
N3061	Tuberculosis of the bones of the shoulder region
N3062	Tuberculosis of the bones of the hand
N3063	Tuberculosis of the bones of the ankle and foot
N3064	Tuberculosis of the bones of other sites
N3065	Tuberculosis of the bones of multiple sites
N306z	Tuberculosis of bone NOS
N307.	Osteopathy from poliomyelitis
N3070	Poliomyelitis osteopathy of unspecified site
N3071	Poliomyelitis osteopathy of the shoulder region
N3072	Poliomyelitis osteopathy of the upper arm
N3073	Poliomyelitis osteopathy of the forearm
N3074	Poliomyelitis osteopathy of the hand
N3075	Poliomyelitis osteopathy of the pelvic region and thigh
N3076	Poliomyelitis osteopathy of the lower leg
N3077	Poliomyelitis osteopathy of the ankle and foot
N3078	Poliomyelitis osteopathy of other specified sites
N3079	Poliomyelitis osteopathy of multiple sites
N307z	Poliomyelitis osteopathy NOS
N308.	Subacute osteomyelitis
N3080	Subacute osteomyelitis-cervical spine
N3081	Subacute osteomyelitis-thoracic spine
N3082	Subacute osteomyelitis-lumbar spine
N3083	Subacute osteomyelitis-sacrum
N3084	Subacute osteomyelitis-coccyx
N309.	Subacute osteomyelitis
N30y.	Other infections involving bone
N30y0	Other infections involving bone, site unspecified
N30y1	Other infections involving bone, of the shoulder region
N30y2	Other infections involving bone, of the upper arm
N30y3	Other infections involving bone, of the forearm
N30y4	Other infections involving bone, of the hand

For peer review only

N30y5	Other infections involving bone, of the pelvic region and thigh
N30y6	Other infections involving bone, of the lower leg
N30y7	Other infections involving bone, of the ankle and foot
N30y8	Other infections involving bone, of other specified site
N30y9	Other infections involving bone, of multiple sites
N30yz	Other infections involving bone, NOS
N30z.	Bone infection NOS
N30z0	Bone infection NOS, of unspecified site
N30z1	Bone infection NOS, of the shoulder region
N30z2	Bone infection NOS, of the upper arm
N30z3	Bone infection NOS, of the forearm
N30z4	Bone infection NOS, of the hand
N30z5	Bone infection NOS, of the pelvic/thigh
N30z6	Bone infection NOS, of the lower leg
N30z7	Bone infection NOS, of ankle and foot
N30z8	Bone infection NOS, of other specified site
N30z9	Bone infection NOS, of multiple sites
N30zz	Bone infection NOS
N31..	Osteitis deformans and osteopathies associated with diseases EC
N310.	Osteitis deformans - Paget's disease of the bone
N3100	Paget's disease-cervical spine
N3101	Paget's disease-thoracic spine
N3102	Paget's disease-lumbar spine
N3103	Paget's disease-sacrum
N3104	Paget's disease-coccyx
N3105	Paget's disease-clavicle
N3106	Paget's disease-scapula
N3107	Paget's disease-humerus
N3108	Paget's disease-radius
N3109	Paget's disease-ulna
N310A	Paget's disease-carpal bone
N310B	Paget's disease-metacarpal
N310C	Paget's disease-phalanx of finger or thumb
N310D	Paget's disease-pelvis
N310E	Paget's disease-femur
N310F	Paget's disease-patella
N310G	Paget's disease-tibia
N310H	Paget's disease-fibula
N310J	Paget's disease-calcaneum
N310K	Paget's disease-talus
N310L	Paget's disease-other tarsal bone
N310M	Paget's disease-metatarsal
N310N	Paget's disease-phalanx of toe
N310P	Paget's disease-skull
N310x	Paget's disease-multiple sites
N310y	Paget's disease OS
N310z	Paget's disease NOS
N311.	Osteitis deformans associated with diseases EC

For peer review only

N3110	Osteitis deformans in neoplastic disease
N312.	Hypertrophic pulmonary osteoarthropathy
N31y.	Other bone involvement in diseases EC
N31z.	Bone involvement in diseases EC NOS
N32..	Osteochondropathies
N320.	Juvenile osteochondritis of the spine
N3200	Juvenile osteochondritis of the spine, unspecified
N3201	Scheuermann's disease
N3202	Calve's vertebral osteochondritis
N320z	Juvenile osteochondritis of the spine NOS
N321.	Juvenile osteochondritis of the hip and pelvis
N3210	Juvenile osteochondritis of the hip and pelvis, unspecified
N3211	Perthes' disease - osteochondritis of the femoral head
N3212	Ischiopubic synchondrosis
N3213	Juvenile osteochondritis of the acetabulum
N3214	Juvenile osteochondritis of the iliac crest
N3215	Juvenile osteochondritis of the symphysis pubis
N3216	Coxa plana
N3217	Pseudocoxalgia
N321z	Juvenile osteochondritis of the hip and pelvis NOS
N322.	Non traumatic slipped upper femoral epiphysis
N3220	Non traumatic acute slipped upper femoral epiphysis
N3221	Non traumatic acute-on-chronic slipped upper femoral epiphysis
N3222	Non traumatic chronic slipped upper femoral epiphysis
N323.	Juvenile osteochondritis of the arm and hand
N3230	Juvenile osteochondritis of the arm, unspecified
N3231	Juvenile osteochondritis of the hand, unspecified
N3232	Panner's disease - osteochondritis of capitulum of humerus
N3233	Kienbock's disease - osteochondritis of carpal lunate
N3234	Haas' disease - osteochondritis of head of humerus
N3235	Mauclaire's disease - osteochondritis of metacarpal heads
N3236	Burn's disease - osteochondritis of the lower ulna
N3237	Brailsford's disease - osteochondritis of the radial head
N323z	Juvenile osteochondritis of the arm and hand NOS
N324.	Juvenile osteochondrosis of the leg
N3240	Juvenile osteochondrosis of the leg, unspecified
N3241	Kohler's disease - osteochondrosis of primary patella centre
N3242	Blount's disease - osteochondrosis of proximal tibia
N3243	Juvenile osteochondrosis of the secondary patellar centre
N3244	Osgood-Schlatter's disease - osteochondrosis of tibial tubercle
N324z	Juvenile osteochondrosis of the leg, NOS
N325.	Juvenile osteochondrosis of the foot
N3250	Juvenile osteochondrosis of the foot, unspecified
N3251	Diaz's disease - osteochondrosis of astragalus
N3252	Sever's disease - osteochondrosis of calcaneum
N3253	Freiberg's disease - osteochondrosis of second metatarsal
N3254	Iselin's disease - osteochondrosis of fifth metatarsal
N3255	Haglund's disease - osteochondrosis of os tibiale externum

For peer review only

N3256	Kohler's disease - osteochondrosis of tarsal navicular
N325z	Juvenile osteochondrosis of the foot NOS
N326.	Other juvenile osteochondroses
N3260	Juvenile apophysitis NOS
N3261	Juvenile epiphysitis NOS
N3262	Juvenile osteochondritis NOS
N3263	Juvenile osteochondrosis NOS
N326z	Juvenile osteochondroses NOS
N327.	Osteochondritis dissecans
N3270	Osteochondritis dissecans of patella
N3271	Osteochondritis dissecans of lateral femoral condyle
N3272	Other osteochondritis dissecans of knee
N3273	Osteochondritis dissecans of the humeral head
N3274	Osteochondritis dissecans of the capitellum
N3275	Osteochondritis dissecans of the radial head
N3276	Other osteochondritis dissecans of the elbow
N3277	Osteochondritis dissecans of the wrist
N3278	Osteochondritis dissecans of the femoral head
N3279	Osteochondritis dissecans of the talus
N327y	Osteochondritis dissecans of other site
N328.	Juvenile osteochondrosis of spine
N32y.	Other specified forms of osteochondropathy
N32y0	Adult osteochondrosis of spine
N32y1	Kienbock's disease of adults
N32yz	Other specified osteochondropathy NOS
N32z.	Osteochondropathy NOS
N32z0	Apophysitis NOS
N32z1	Epiphysitis NOS
N32z2	Osteochondritis NOS
N32z3	Osteochondrosis NOS
N32zz	Osteochondropathy NOS
N33..	Other bone and cartilage disorders
N330.	Osteoporosis
N3300	Osteoporosis, unspecified
N3301	Senile osteoporosis
N3302	Postmenopausal osteoporosis
N3303	Idiopathic osteoporosis
N3304	Dissuse osteoporosis
N3305	Drug-induced osteoporosis
N3306	Postoophorectomy osteoporosis
N3307	Postsurgical malabsorption osteoporosis
N3308	Localized osteoporosis - Lequesne
N3309	Osteoporosis in multiple myelomatosis
N330A	Osteoporosis in endocrine disorders
N330B	Vertebral osteoporosis
N330C	Osteoporosis localized to spine
N330D	Osteoporosis due to corticosteroids
N330z	Osteoporosis NOS

For peer review only

N331.	Pathological fracture
N3310	Pathological fracture of thoracic vertebra
N3311	Pathological fracture of lumbar vertebra
N3312	Postoophorectomy osteoporosis with pathological fracture
N3313	Osteoporosis of disuse with pathological fracture
N3314	Postsurgical malabsorption osteoporosis with pathological fracture
N3315	Drug-induced osteoporosis with pathological fracture
N3316	Idiopathic osteoporosis with pathological fracture
N3317	Fracture of bone in neoplastic disease
N3318	Osteoporosis + pathological fracture lumbar vertebrae
N3319	Osteoporosis + pathological fracture thoracic vertebrae
N331A	Osteoporosis + pathological fracture cervical vertebrae
N331B	Postmenopausal osteoporosis with pathological fracture
N331C	Pathological fracture of cervical vertebra
N331D	Collapsed vertebra NOS
N331E	Collapse of cervical vertebra
N331F	Collapse of thoracic vertebra
N331G	Collapse of lumbar vertebra
N331H	Collapse of cervical vertebra due to osteoporosis
N331J	Collapse of lumbar vertebra due to osteoporosis
N331K	Collapse of thoracic vertebra due to osteoporosis
N331L	Collapse of vertebra due to osteoporosis NOS
N331M	Fragility fracture due to unspecified osteoporosis
N331N	Fragility fracture
N331y	Other specified pathological fracture
N331z	Pathological fracture NOS
N332.	Cyst of bone
N3320	Localised bone cyst, unspecified
N3321	Solitary bone cyst
N3322	Aneurysmal bone cyst
N3323	Monostotic fibrous dysplasia
N3324	Fibrous cortical defect
N3325	Brown tumour of hyperparathyroidism
N332z	Cyst of bone NOS
N333.	Hyperostosis of skull
N3330	Hyperostosis interna frontalis
N3331	Leontiasis ossium
N333z	Hyperostosis of skull NOS
N334.	Avascular necrosis of bone
N3340	Avascular necrosis of bone, site unspecified
N3341	Avascular necrosis of the head of humerus
N3342	Avascular necrosis of the head of femur
N3343	Avascular necrosis of the medial femoral condyle
N3344	Avascular necrosis of the talus
N3345	Avascular necrosis of capitellum
N3346	Avascular necrosis of lateral femoral condyle
N3347	Avascular necrosis of other bone
N3348	Idiopathic aseptic necrosis of bone

For peer review only

N3349	Osteonecrosis due to drugs
N334A	Osteonecrosis due to previous trauma
N334B	Osteonecrosis in caisson disease
N334C	Osteonecrosis due to haemoglobinopathy
N334z	Avascular bone necrosis NOS
N335.	Osteitis condensans
N3350	Osteitis condensans ilii
N336.	Tietze's disease
N337.	Algoneurodystrophy
N3370	Disuse atrophy of bone
N3371	Sudek's atrophy
N3372	Algodystrophy of hand
N3373	Algodystrophy of knee
N3374	Algodystrophy of foot
N337z	Algoneurodystrophy NOS
N338.	Malunion and nonunion of fracture
N3380	Malunion of fracture
N3381	Pseudoarthrosis - fracture nonunion
N3382	Hypertrophic non-union of fracture
N3383	Atrophic non-union of fracture
N3384	Angular mal-union of fracture
N3385	Rotational mal-union of fracture
N3386	Delayed union of fracture
N338z	Fracture malunion or nonunion NOS
N339.	Residual foreign body in bone
N33A.	Bone pain
N33A0	Bony pelvic pain
N33A1	Clavicle pain
N33B.	Osteoradionecrosis
N33C.	Complex regional pain syndrome type I
N33z.	Other and unspecified bone or cartilage disorders
N33z0	Bone and cartilage disorders, unspecified
N33z1	Epiphyseal arrest
N33z2	Chondromalacia NOS
N33z3	Diaphysitis
N33z4	Hypertrophy of bone
N33z5	Relapsing polychondritis
N33z6	Compensatory hypertrophy of bone
N33z7	Idiopathic hypertrophy of bone
N33z8	Complete epiphyseal arrest
N33z9	Partial epiphyseal arrest
N33zA	Skeletal fluorosis
N33zB	Chondrolysis
N33zC	Pseudarthrosis after fusion or arthrodesis
N33zD	Osteolysis
N33zE	Costochondritis
N33zF	Disorder of bone, unspecified
N33zG	Disorder of cartilage, unspecified

For peer review only

N33zH	Osteolytic lesion
N33zJ	Chondritis
N33zK	Hajdu-Cheney syndrome
N33zL	Osteitis of symphysis pubis
N33zM	Adynamic bone disease
N33zz	Bone or cartilage disorders NOS
N34..	Flat foot
N340.	Pes planus - acquired
N3400	Hypermobile flat foot
N3401	Rigid flat foot
N3402	Peroneal spastic flat foot
N341.	Talipes planus - acquired
N34z.	Flat foot NOS
N35..	Acquired deformities of toe
N350.	Hallux valgus - acquired
N351.	Hallux varus - acquired
N352.	Hallux rigidus - acquired
N353.	Acquired hammer deformity of great toe
N354.	Other hammer toe - acquired
N355.	Claw toe - acquired
N356.	Clawing of great toe
N357.	Crossover toe
N358.	Mallet toe
N359.	Bunionette
N35A.	Over-riding 5th toe
N35B.	Acquired varus deformity of toe
N35C.	Acquired hammer toe, other than great toe
N35y.	Other acquired toe deformity
N35z.	Acquired toe deformity NOS
N36..	Other acquired limb deformity
N360.	Acquired forearm deformity, excluding fingers
N3600	Acquired forearm deformity, unspecified
N3601	Cubitus valgus - acquired
N3602	Cubitus varus - acquired
N3603	Acquired valgus wrist deformity
N3604	Acquired varus wrist deformity
N3605	Wrist drop - acquired
N3606	Claw hand - acquired
N3607	Club hand - acquired
N360z	Acquired forearm deformity NOS
N361.	Mallet finger
N362.	Other acquired finger deformity
N3620	Acquired finger deformity, unspecified
N3621	Boutonniere finger deformity
N3622	Swan-neck finger deformity
N3623	Flexion deformity of finger
N3624	Extension deformity of finger
N3625	Deviation of finger

For peer review only

N3626	Rotational deformity of finger
N362z	Acquired finger deformity NOS
N363.	Acquired deformities of hip
N3630	Acquired hip deformity, unspecified
N3631	Coxa valga - acquired
N3632	Coxa vara - acquired
N3633	Acquired internal femoral torsion
N3634	Persistent femoral anteversion
N3635	Acquired external femoral torsion
N363z	Acquired hip deformity NOS
N364.	Acquired genu valgum and varum
N3640	Acquired genu valgum
N3641	Acquired genu varum
N364z	Acquired genu valgum or varum NOS
N365.	Genu recurvatum - acquired
N366.	Acquired knee deformity NOS
N3660	Flexion deformity of knee
N367.	Other acquired ankle and foot deformity
N3670	Acquired ankle or foot deformity, unspecified
N3671	Acquired equinovarus - clubfoot
N3672	Acquired equinus foot deformity
N3673	Aquired cavus foot deformity
N3674	Acquired claw foot
N3675	Acquired cavovarus foot deformity
N3676	Other acquired calcaneus deformity
N3677	Acquired talipes NEC
N3678	Acquired varus heel
N3679	Acquired valgus heel
N367A	Plantar flexion of the midtarsal joint
N367B	Plantar flexion contracture of tarsometatarsal joint
N367C	Flexion contracture of metatarsophalangeal joint
N367D	Extension contracture of metatarsophalangeal joint
N367E	Flexion contracture of toe joint
N367F	Acquired plantar-flexed forefoot
N367G	Acquired plantar-flexed first ray
N367H	Acquired plantar-flexed fifth ray
N367J	Acquired dorsiflexed forefoot
N367K	Acquired dorsiflexed first ray
N367L	Acquired supinated forefoot
N367M	Acquired pronated forefoot
N367N	Acquired forefoot adductus
N367P	Acquired forefoot abductus
N367Q	Serpentine foot
N367z	Acquired ankle or foot deformity NOS
N368.	Other knee deformity
N3680	Acquired internal tibial torsion
N3681	Acquired external tibial torsion
N3682	Chronic instability of knee

For peer review only

N369.	Flexion deformity
N36A.	Foot drop
N36y.	Acquired deformity of other limb parts
N36y0	Acquired unequal leg length
N36y1	Acquired unequal arm length
N36y2	Deformity of bone
N36y3	Deformity of clavicle
N36y4	Deformity of scapula
N36y5	Deformity of humerus
N36y6	Deformity of radius
N36y7	Deformity of ulna
N36y8	Deformity of carpal bone
N36y9	Deformity of metacarpal
N36yA	Deformity of phalanx of finger or thumb
N36yB	Deformity of pelvis
N36yC	Deformity of femur
N36yD	Deformity of patella
N36yE	Deformity of tibia
N36yF	Deformity of fibula
N36yG	Deformity of calcaneum
N36yH	Deformity of talus
N36yJ	Deformity of other tarsal bone
N36yK	Deformity of metatarsal
N36yL	Deformity of phalanx of toe
N36yM	Old amputee NOS
N36yN	Acquired radial deviation of hand
N36yz	Acquired limb deformity NEC
N36z.	Acquired limb deformity NOS
N37..	Curvature of spine
N370.	Adolescent postural kyphosis
N371.	Acquired kyphosis
N3710	Acquired postural kyphosis
N3711	Radiation kyphosis
N3712	Post-laminectomy kyphosis
N3713	Kyphosis secondary to other treatment
N371z	Acquired kyphosis NOS
N372.	Acquired lordosis
N3720	Acquired postural lordosis
N3721	Post-laminectomy lordosis
N3722	Other post-surgical lordosis
N372z	Acquired lordosis NOS
N373.	Kyphoscoliosis and scoliosis
N3730	Idiopathic scoliosis
N3731	Idiopathic kyphoscoliosis
N3732	Resolving infantile idiopathic scoliosis
N3733	Progressive infantile idiopathic scoliosis
N3734	Radiation scoliosis
N3735	Thoracogenic scoliosis

For peer review only

N3736	Postural scoliosis
N3737	Adolescent idiopathic scoliosis
N3738	Post-surgical scoliosis
N3739	Scoliosis secondary to other treatment
N373z	Kyphoscoliosis or scoliosis NOS
N374.	Curvature of spine associated with other conditions
N3740	Curvature of spine, unspecified
N3741	Kyphosis associated with other condition
N3742	Lordosis associated with other condition
N3743	Scoliosis associated with other condition
N3744	Kyphosis in skeletal dysplasia
N3745	Neuromuscular kyphosis
N3746	Osteoporotic kyphosis
N3747	Lordosis in skeletal dysplasia
N3748	Lordosis in hip disease
N3749	Neuromuscular lordosis
N374A	Scoliosis in skeletal dysplasia
N374B	Neuromuscular scoliosis
N374C	Scoliosis in neurofibromatosis
N374D	Scoliosis in connective tissue anomalies
N374E	Flatback syndrome
N374W	Lordosis, unspecified
N374X	Other and unspecified kyphosis
N374z	Curvature of spine associated with other conditions NOS
N37y.	Other curvatures of spine
N37z.	Curvature of spine NOS
N37z0	Acquired hunchback
N37zz	Curvature of spine NOS
N38..	Other acquired deformity
N380.	Acquired deformity of nose
N381.	Other acquired head deformity
N382.	Acquired deformity of neck
N383.	Acquired chest and rib deformity
N3830	Acquired chest deformity, unspecified
N3831	Acquired rib deformity, unspecified
N3832	Acquired pectus carinatum
N3833	Acquired pectus excavatum
N383z	Acquired chest or rib deformity NOS
N384.	Acquired spondylolisthesis
N3840	Dysplastic spondylolisthesis
N3841	Isthmic spondylolisthesis
N3842	Degenerative spondylolisthesis
N3843	Pedicular spondylolisthesis
N385.	Acquired deformity of spine NOS
N386.	Acquired deformity of pelvis
N387.	Cauliflower ear
N388.	Spondylolysis
N38y.	Other acquired deformity

For peer review only

N38y0	Acquired clavicle deformity
N38yz	Other acquired deformity NOS
N38z.	Acquired deformity NOS
N39..	Nonallopathic lesions, NEC
N390.	Nonallopathic lesion of the head region
N391.	Nonallopathic lesion of the cervical region
N392.	Nonallopathic lesion of the thoracic region
N393.	Nonallopathic lesion of the lumbar region
N394.	Nonallopathic lesion of the sacral region
N395.	Nonallopathic lesion of the pelvic region
N396.	Nonallopathic lesion of the legs
N397.	Nonallopathic lesion of the arms
N398.	Nonallopathic lesion of the rib cage
N399.	Nonallopathic lesion of the abdomen and other regions
N39z.	Nonallopathic lesion NEC NOS
N3y..	Other specified musculoskeletal disorders
N3y0.	Biomechanical lesions, not elsewhere classified
N3y00	Segmental and somatic dysfunction
N3y01	Subluxation complex (vertebral)
N3y02	Subluxation stenosis of neural canal
N3y03	Osseous stenosis of neural canal
N3y04	Connective tissue stenosis of neural canal
N3y05	Intervertebral disc stenosis of neural canal
N3y06	Osseous and subluxation stenosis of intervertebral foramina
N3y07	Connective tissue and disc stenosis of intervertebral foramina
N3z..	Other musculoskeletal disorder NOS
Ny...	Other specified diseases of musculoskeletal or connective tissue
Ny2..	Repetitive strain injury
Ny20.	Work related upper limb disorder
NyX..	Postprocedural musculoskeletal disorder, unspecified
Nyu..	[X]Additional musculoskeletal and connective tissue disease classification terms
Nyu0.	[X]Infectious arthropathies
Nyu00	[X]Other streptococcal arthritis and polyarthritis
Nyu01	[X]Arthritis and polyarthritis due to other specified bacterial agents
Nyu02	[X]Arthritis in other infectious and parasitic diseases classified elsewhere
Nyu03	[X]Other reactive arthropathies
Nyu04	[X]Other postinfectious arthropathies in diseases classified elsewhere
Nyu05	[X]Reactive arthropathy in other diseases classified elsewhere
Nyu1.	[X]Inflammatory polyarthropathies
Nyu10	[X]Rheumatoid arthritis with involvement of other organs or systems
Nyu11	[X]Other seropositive rheumatoid arthritis
Nyu12	[X]Other specified rheumatoid arthritis
Nyu13	[X]Other psoriatic arthropathies
Nyu14	[X]Other enteropathic arthropathies
Nyu15	[X]Other juvenile arthritis
Nyu16	[X]Juvenile arthritis in other diseases classified elsewhere
Nyu17	[X]Other secondary gout
Nyu18	[X]Other chondrocalcinosis

For peer review only

Nyu19	[X]Other specified crystal arthropathies
Nyu1A	[X]Other specific arthropathies, not elsewhere classified
Nyu1B	[X]Other specified arthritis
Nyu1C	[X]Gouty arthropathy due to enzyme defects and other inherited disorders classified elsewhere
Nyu1D	[X]Crystal arthropathy in other metabolic disorders classified elsewhere
Nyu1E	[X]Arthropathies in other endocrine, nutritional and metabolic disorders
Nyu1F	[X]Arthropathies in other specified diseases classified elsewhere
Nyu1G	[X]Seropositive rheumatoid arthritis, unspecified
Nyu2.	[X]Arthrosis
Nyu20	[X]Other polyarthrosis
Nyu21	[X]Other primary coxarthrosis
Nyu22	[X]Other dysplastic coxarthrosis
Nyu23	[X]Other post-traumatic coxarthrosis
Nyu24	[X]Other secondary coxarthrosis, bilateral
Nyu25	[X]Other primary gonarthrosis
Nyu26	[X]Other post-traumatic gonarthrosis
Nyu27	[X]Other secondary gonarthrosis, bilateral
Nyu28	[X]Other secondary gonarthrosis
Nyu29	[X]Other primary arthrosis of first carpometacarpal joint
Nyu2A	[X]Other post-traumatic arthrosis of first carpometacarpal joint
Nyu2B	[X]Other secondary arthrosis of first carpometacarpal joints, bilateral
Nyu2C	[X]Other secondary arthrosis of first carpometacarpal joint
Nyu2D	[X]Other specified arthrosis
Nyu2E	[X]Other secondary coxarthrosis
Nyu2F	[X]Post-traumatic arthrosis of other joints
Nyu3.	[X]Other joint disorders
Nyu30	[X]Other deformity of hallux (acquired)
Nyu31	[X]Other hammer toe(s) (acquired)
Nyu32	[X]Other deformities of toe(s) (acquired)
Nyu33	[X]Other acquired deformities of ankle and foot
Nyu34	[X]Other specified acquired deformities of limbs
Nyu35	[X]Other derangements of patella
Nyu36	[X]Other disorders of patella
Nyu37	[X]Other meniscus derangements
Nyu38	[X]Other spontaneous disruption of ligament(s) of knee
Nyu39	[X]Other internal derangements of knee
Nyu3A	[X]Other articular cartilage disorders
Nyu3B	[X]Other specific joint derangements, not elsewhere classified
Nyu3C	[X]Other instability of joint
Nyu3D	[X]Other specified joint disorders
Nyu3E	[X]Disorder of patella, unspecified
Nyu4.	[X]Systemic connective tissue disorders
Nyu40	[X]Other conditions related to polyarteritis nodosa
Nyu41	[X]Other giant cell arteritis
Nyu42	[X]Other specified necrotizing vasculopathies
Nyu43	[X]Other forms of systemic lupus erythematosus
Nyu44	[X]Other dermatomyositis

For peer review only

Nyu45	[X]Other forms of systemic sclerosis
Nyu46	[X]Other overlap syndromes
Nyu47	[X]Other systemic diseases of connective tissue
Nyu48	[X]Dermato(poly)myositis in neoplastic disease classified elsewhere
Nyu49	[X]Arthropathy in neoplastic disease classified elsewhere
Nyu4A	[X]Arthropathy in other blood disorders classified elsewhere
Nyu4B	[X]Arthropathy in hypersensitivity reactions classified elsewhere
Nyu4C	[X]Systemic disorders of connective tissue in other diseases classified elsewhere
Nyu4D	[X]Necrotising vasculopathy, unspecified
Nyu4E	[X]Dermatopolymyositis, unspecified
Nyu4F	[X]Mixed connective tissue disease
Nyu5.	[X]Deforming dorsopathies
Nyu50	[X]Other secondary kyphosis
Nyu51	[X]Other and unspecified kyphosis
Nyu52	[X]Other lordosis
Nyu53	[X]Other idiopathic scoliosis
Nyu54	[X]Other secondary scoliosis
Nyu55	[X]Other forms of scoliosis
Nyu56	[X]Other fusion of spine
Nyu57	[X]Other recurrent atlantoaxial subluxation
Nyu58	[X]Other recurrent vertebral subluxation
Nyu59	[X]Other specified deforming dorsopathies
Nyu5A	[X]Lordosis, unspecified
Nyu5B	[X]Spinal osteochondrosis, unspecified
Nyu6.	[X]Spondylopathies
Nyu60	[X]Other infective spondylopathies
Nyu61	[X]Other specified inflammatory spondylopathies
Nyu62	[X]Other spondylosis with myelopathy
Nyu63	[X]Other spondylosis with radiculopathy
Nyu64	[X]Other spondylosis
Nyu65	[X]Other specified spondylopathies
Nyu66	[X]Spondylopathy in other infectious and parasitic diseases classified elsewhere
Nyu67	[X]Collapsed vertebra in diseases classified elsewhere
Nyu68	[X]Spondylopathy in other diseases classified elsewhere
Nyu69	[X]Spondylopathy, unspecified
Nyu7.	[X]Other dorsopathies
Nyu70	[X]Other cervical disc displacement
Nyu71	[X]Other cervical disc degeneration
Nyu72	[X]Other cervical disc disorders
Nyu73	[X]Lumbar and other intervertebral disc disorders with myelopathy
Nyu74	[X]Lumbar and other intervertebral disc disorders with radiculopathy
Nyu75	[X]Other specified intervertebral disc displacement
Nyu76	[X]Other specified intervertebral disc degeneration
Nyu77	[X]Other specified intervertebral disc disorders
Nyu78	[X]Sacrococcygeal disorders, not elsewhere classified
Nyu79	[X]Other specified dorsopathies
Nyu7A	[X]Other dorsalgia
Nyu7B	[X]Cervical disc disorder, unspecified

For peer review only

Nyu8.	[X]Disorders of muscles
Nyu80	[X]Other myositis
Nyu81	[X]Other calcification of muscle
Nyu82	[X]Other ossification of muscle
Nyu83	[X]Other rupture of muscle (nontraumatic)
Nyu84	[X]Muscle wasting and atrophy, not elsewhere classified
Nyu85	[X]Other specified disorders of muscle
Nyu86	[X]Myositis in bacterial diseases classified elsewhere
Nyu87	[X]Myositis in protozoal and parasitic infections classified elsewhere
Nyu88	[X]Myositis in other infectious diseases classified elsewhere
Nyu89	[X]Myositis in sarcoidosis classified elsewhere
Nyu8A	[X]Other disorders of muscle in diseases classified elsewhere
Nyu8B	[X]Disorder of muscle, unspecified
Nyu9.	[X]Disorders of synovium and tendon
Nyu90	[X]Other infective (teno)synovitis
Nyu91	[X]Other synovitis and tenosynovitis
Nyu92	[X]Spontaneous rupture of other tendons
Nyu93	[X]Other contracture of tendon (sheath)
Nyu94	[X]Other specified disorders of synovium and tendon
Nyu95	[X]Synovitis and tenosynovitis in bacterial diseases classified elsewhere
Nyu96	[X]Other disorders of synovium and tendon in diseases classified elsewhere
Nyu97	[X]Synovial hypertrophy, not elsewhere classified
NyuA.	[X]Other soft tissue disorders
NyuA0	[X]Other bursitis of elbow
NyuA1	[X]Other bursitis of knee
NyuA2	[X]Other bursitis of hip
NyuA3	[X]Other soft tissue disorders related to use, overuse and pressure
NyuA4	[X]Other infective bursitis
NyuA5	[X]Other bursal cyst
NyuA6	[X]Other bursitis, not elsewhere classified
NyuA7	[X]Other specified bursopathies
NyuA8	[X]Fasciitis, not elsewhere classified
NyuA9	[X]Other fibroblastic disorders
NyuAA	[X]Other soft tissue disorders in other diseases classified elsewhere
NyuAB	[X]Other shoulder lesions
NyuAC	[X]Other enthesopathies of lower limb, excluding foot
NyuAD	[X]Other enthesopathy of foot
NyuAE	[X]Other enthesopathies, not elsewhere classified
NyuAF	[X]Other specified soft tissue disorders
NyuAG	[X]Unspecified soft tissue disorder related to use, overuse and pressure
NyuAH	[X]Fibroblastic disorder, unspecified
NyuAJ	[X]Enthesopathy of lower limb, unspecified
NyuB.	[X]Disorders of bone density and structure
NyuB0	[X]Other osteoporosis with pathological fracture
NyuB1	[X]Other osteoporosis
NyuB2	[X]Osteoporosis in other disorders classified elsewhere
NyuB3	[X]Other drug-induced osteomalacia in adults
NyuB4	[X]Other adult osteomalacia

For peer review only

NyuB5	[X]Other disorders of continuity of bone
NyuB6	[X]Other cyst of bone
NyuB7	[X]Other specified disorders of bone density and structure
NyuB8	[X]Unspecified osteoporosis with pathological fracture
NyuB9	[X]Adult osteomalacia, unspecified
NyuBA	[X]Disorder of bone density and structure, unspecified
NyuBB	[X]Erosion of bone
NyuBC	[X]Osteopenia
NyuC.	[X]Other osteopathies
NyuC0	[X]Other acute osteomyelitis
NyuC1	[X]Other chronic haematogenous osteomyelitis
NyuC2	[X]Other chronic osteomyelitis
NyuC3	[X]Other osteomyelitis
NyuC4	[X]Other secondary osteonecrosis
NyuC5	[X]Other osteonecrosis
NyuC6	[X]Other disorders of bone development and growth
NyuC7	[X]Other hypertrophic osteoarthropathy
NyuC8	[X]Other specified disorders of bone
NyuC9	[X]Periostitis in other infectious diseases classified elsewhere
NyuCA	[X]Osteopathy in other infectious diseases classified elsewhere
NyuCB	[X]Osteonecrosis due to haemoglobinopathy classified elsewhere
NyuCC	[X]Osteonecrosis in other diseases classified elsewhere
NyuCD	[X]Osteitis deformans in neoplastic diseases classified elsewhere
NyuCE	[X]Fracture of bone in neoplastic diseases classified elsewhere
NyuCF	[X]Osteopathy in other diseases classified elsewhere
NyuD.	[X]Chondropathies
NyuD0	[X]Other juvenile osteochondrosis of hip and pelvis
NyuD1	[X]Other juvenile osteochondrosis of upper limb
NyuD2	[X]Other specified juvenile osteochondrosis
NyuD3	[X]Other specified osteochondropathies
NyuD4	[X]Other specified disorders of cartilage
NyuDE	[X]Disorder of cartilage, unspecified
NyuE.	[X]Other disorders of the musculoskeletal system and connective tissue
NyuE0	[X]Other specified acquired deformities of musculoskeletal system
NyuE1	[X]Fracture of bone following insertion of orthopaedic implant, joint prosthesis, or bone plate
NyuE2	[X]Other postprocedural musculoskeletal disorders
NyuE3	[X]Other biomechanical lesions
NyuE4	[X]Postprocedural musculoskeletal disorder, unspecified
Nz...	Musculoskeletal and connective tissue diseases NOS

Hospital

ALT_CODE	DESCRIPTION
M00	Pyogenic arthritis
M000	Staphylococcal arthritis and polyarthritis
M0000	Staphylococcal arthritis and polyarthritis

For peer review only

M0001	Staphylococcal arthritis and polyarthritis
M0002	Staphylococcal arthritis and polyarthritis
M0003	Staphylococcal arthritis and polyarthritis
M0004	Staphylococcal arthritis and polyarthritis
M0005	Staphylococcal arthritis and polyarthritis
M0006	Staphylococcal arthritis and polyarthritis
M0007	Staphylococcal arthritis and polyarthritis
M0008	Staphylococcal arthritis and polyarthritis
M0009	Staphylococcal arthritis and polyarthritis
M001	Pneumococcal arthritis and polyarthritis
M0010	Pneumococcal arthritis and polyarthritis
M0011	Pneumococcal arthritis and polyarthritis
M0012	Pneumococcal arthritis and polyarthritis
M0013	Pneumococcal arthritis and polyarthritis
M0014	Pneumococcal arthritis and polyarthritis
M0015	Pneumococcal arthritis and polyarthritis
M0016	Pneumococcal arthritis and polyarthritis
M0017	Pneumococcal arthritis and polyarthritis
M0018	Pneumococcal arthritis and polyarthritis
M0019	Pneumococcal arthritis and polyarthritis
M002	Other streptococcal arthritis and polyarthritis
M0020	Other streptococcal arthritis and polyarthritis
M0021	Other streptococcal arthritis and polyarthritis
M0022	Other streptococcal arthritis and polyarthritis
M0023	Other streptococcal arthritis and polyarthritis
M0024	Other streptococcal arthritis and polyarthritis
M0025	Other streptococcal arthritis and polyarthritis
M0026	Other streptococcal arthritis and polyarthritis
M0027	Other streptococcal arthritis and polyarthritis
M0028	Other streptococcal arthritis and polyarthritis
M0029	Other streptococcal arthritis and polyarthritis
M008	Arthritis and polyarthritis due to other specified bacterial agents
M0080	Arthritis and polyarthritis due to other specified bacterial agents
M0081	Arthritis and polyarthritis due to other specified bacterial agents
M0082	Arthritis and polyarthritis due to other specified bacterial agents
M0083	Arthritis and polyarthritis due to other specified bacterial agents
M0084	Arthritis and polyarthritis due to other specified bacterial agents
M0085	Arthritis and polyarthritis due to other specified bacterial agents
M0086	Arthritis and polyarthritis due to other specified bacterial agents
M0087	Arthritis and polyarthritis due to other specified bacterial agents
M0088	Arthritis and polyarthritis due to other specified bacterial agents
M0089	Arthritis and polyarthritis due to other specified bacterial agents
M009	Pyogenic arthritis, unspecified
M0090	Pyogenic arthritis, unspecified
M0091	Pyogenic arthritis, unspecified
M0092	Pyogenic arthritis, unspecified
M0093	Pyogenic arthritis, unspecified
M0094	Pyogenic arthritis, unspecified

For peer review only

M0095	Pyogenic arthritis, unspecified
M0096	Pyogenic arthritis, unspecified
M0097	Pyogenic arthritis, unspecified
M0098	Pyogenic arthritis, unspecified
M0099	Pyogenic arthritis, unspecified
M01	Direct infections of joint in infectious and parasitic diseases classified elsewhere
M010	Meningococcal arthritis
M0100	Meningococcal arthritis
M0101	Meningococcal arthritis
M0102	Meningococcal arthritis
M0103	Meningococcal arthritis
M0104	Meningococcal arthritis
M0105	Meningococcal arthritis
M0106	Meningococcal arthritis
M0107	Meningococcal arthritis
M0108	Meningococcal arthritis
M0109	Meningococcal arthritis
M011	Tuberculous arthritis
M0110	Tuberculous arthritis
M0111	Tuberculous arthritis
M0112	Tuberculous arthritis
M0113	Tuberculous arthritis
M0114	Tuberculous arthritis
M0115	Tuberculous arthritis
M0116	Tuberculous arthritis
M0117	Tuberculous arthritis
M0118	Tuberculous arthritis
M0119	Tuberculous arthritis
M012	Arthritis in Lyme disease
M0120	Arthritis in Lyme disease
M0121	Arthritis in Lyme disease
M0122	Arthritis in Lyme disease
M0123	Arthritis in Lyme disease
M0124	Arthritis in Lyme disease
M0125	Arthritis in Lyme disease
M0126	Arthritis in Lyme disease
M0127	Arthritis in Lyme disease
M0128	Arthritis in Lyme disease
M0129	Arthritis in Lyme disease
M013	Arthritis in other bacterial diseases classified elsewhere
M0130	Arthritis in other bacterial diseases classified elsewhere
M0131	Arthritis in other bacterial diseases classified elsewhere
M0132	Arthritis in other bacterial diseases classified elsewhere
M0133	Arthritis in other bacterial diseases classified elsewhere
M0134	Arthritis in other bacterial diseases classified elsewhere
M0135	Arthritis in other bacterial diseases classified elsewhere
M0136	Arthritis in other bacterial diseases classified elsewhere
M0137	Arthritis in other bacterial diseases classified elsewhere

For peer review only

M0138	Arthritis in other bacterial diseases classified elsewhere
M0139	Arthritis in other bacterial diseases classified elsewhere
M014	Rubella arthritis
M0140	Rubella arthritis
M0141	Rubella arthritis
M0142	Rubella arthritis
M0143	Rubella arthritis
M0144	Rubella arthritis
M0145	Rubella arthritis
M0146	Rubella arthritis
M0147	Rubella arthritis
M0148	Rubella arthritis
M0149	Rubella arthritis
M015	Arthritis in other viral diseases classified elsewhere
M0150	Arthritis in other viral diseases classified elsewhere
M0151	Arthritis in other viral diseases classified elsewhere
M0152	Arthritis in other viral diseases classified elsewhere
M0153	Arthritis in other viral diseases classified elsewhere
M0154	Arthritis in other viral diseases classified elsewhere
M0155	Arthritis in other viral diseases classified elsewhere
M0156	Arthritis in other viral diseases classified elsewhere
M0157	Arthritis in other viral diseases classified elsewhere
M0158	Arthritis in other viral diseases classified elsewhere
M0159	Arthritis in other viral diseases classified elsewhere
M016	Arthritis in mycoses
M0160	Arthritis in mycoses
M0161	Arthritis in mycoses
M0162	Arthritis in mycoses
M0163	Arthritis in mycoses
M0164	Arthritis in mycoses
M0165	Arthritis in mycoses
M0166	Arthritis in mycoses
M0167	Arthritis in mycoses
M0168	Arthritis in mycoses
M0169	Arthritis in mycoses
M018	Arthritis in other infectious and parasitic diseases classified elsewhere
M0180	Arthritis in other infectious and parasitic diseases classified elsewhere
M0181	Arthritis in other infectious and parasitic diseases classified elsewhere
M0182	Arthritis in other infectious and parasitic diseases classified elsewhere
M0183	Arthritis in other infectious and parasitic diseases classified elsewhere
M0184	Arthritis in other infectious and parasitic diseases classified elsewhere
M0185	Arthritis in other infectious and parasitic diseases classified elsewhere
M0186	Arthritis in other infectious and parasitic diseases classified elsewhere
M0187	Arthritis in other infectious and parasitic diseases classified elsewhere
M0188	Arthritis in other infectious and parasitic diseases classified elsewhere
M0189	Arthritis in other infectious and parasitic diseases classified elsewhere
M02	Reactive arthropathies
M020	Arthropathy following intestinal bypass

For peer review only

M0200	Arthropathy following intestinal bypass
M0201	Arthropathy following intestinal bypass
M0202	Arthropathy following intestinal bypass
M0203	Arthropathy following intestinal bypass
M0204	Arthropathy following intestinal bypass
M0205	Arthropathy following intestinal bypass
M0206	Arthropathy following intestinal bypass
M0207	Arthropathy following intestinal bypass
M0208	Arthropathy following intestinal bypass
M0209	Arthropathy following intestinal bypass
M021	Postdysenteric arthropathy
M0210	Postdysenteric arthropathy
M0211	Postdysenteric arthropathy
M0212	Postdysenteric arthropathy
M0213	Postdysenteric arthropathy
M0214	Postdysenteric arthropathy
M0215	Postdysenteric arthropathy
M0216	Postdysenteric arthropathy
M0217	Postdysenteric arthropathy
M0218	Postdysenteric arthropathy
M0219	Postdysenteric arthropathy
M022	Postimmunization arthropathy
M0220	Postimmunization arthropathy
M0221	Postimmunization arthropathy
M0222	Postimmunization arthropathy
M0223	Postimmunization arthropathy
M0224	Postimmunization arthropathy
M0225	Postimmunization arthropathy
M0226	Postimmunization arthropathy
M0227	Postimmunization arthropathy
M0228	Postimmunization arthropathy
M0229	Postimmunization arthropathy
M023	Reiter disease
M023	Reiter's disease
M0230	Reiter disease
M0230	Reiter's disease
M0231	Reiter disease
M0231	Reiter's disease
M0232	Reiter disease
M0232	Reiter's disease
M0233	Reiter disease
M0233	Reiter's disease
M0234	Reiter disease
M0234	Reiter's disease
M0235	Reiter disease
M0235	Reiter's disease
M0236	Reiter disease
M0236	Reiter's disease

For peer review only

M0237	Reiter disease
M0237	Reiter's disease
M0238	Reiter disease
M0238	Reiter's disease
M0239	Reiter disease
M0239	Reiter's disease
M028	Other reactive arthropathies
M0280	Other reactive arthropathies
M0281	Other reactive arthropathies
M0282	Other reactive arthropathies
M0283	Other reactive arthropathies
M0284	Other reactive arthropathies
M0285	Other reactive arthropathies
M0286	Other reactive arthropathies
M0287	Other reactive arthropathies
M0288	Other reactive arthropathies
M0289	Other reactive arthropathies
M029	Reactive arthropathy, unspecified
M0290	Reactive arthropathy, unspecified
M0291	Reactive arthropathy, unspecified
M0292	Reactive arthropathy, unspecified
M0293	Reactive arthropathy, unspecified
M0294	Reactive arthropathy, unspecified
M0295	Reactive arthropathy, unspecified
M0296	Reactive arthropathy, unspecified
M0297	Reactive arthropathy, unspecified
M0298	Reactive arthropathy, unspecified
M0299	Reactive arthropathy, unspecified
M03	Postinfective and reactive arthropathies in diseases classified elsewhere
M030	Postmeningococcal arthritis
M0300	Postmeningococcal arthritis
M0301	Postmeningococcal arthritis
M0302	Postmeningococcal arthritis
M0303	Postmeningococcal arthritis
M0304	Postmeningococcal arthritis
M0305	Postmeningococcal arthritis
M0306	Postmeningococcal arthritis
M0307	Postmeningococcal arthritis
M0308	Postmeningococcal arthritis
M0309	Postmeningococcal arthritis
M031	Postinfective arthropathy in syphilis
M0310	Postinfective arthropathy in syphilis
M0311	Postinfective arthropathy in syphilis
M0312	Postinfective arthropathy in syphilis
M0313	Postinfective arthropathy in syphilis
M0314	Postinfective arthropathy in syphilis
M0315	Postinfective arthropathy in syphilis
M0316	Postinfective arthropathy in syphilis

For peer review only

M0317	Postinfective arthropathy in syphilis
M0318	Postinfective arthropathy in syphilis
M0319	Postinfective arthropathy in syphilis
M032	Other postinfectious arthropathies in diseases classified elsewhere
M0320	Other postinfectious arthropathies in diseases classified elsewhere
M0321	Other postinfectious arthropathies in diseases classified elsewhere
M0322	Other postinfectious arthropathies in diseases classified elsewhere
M0323	Other postinfectious arthropathies in diseases classified elsewhere
M0324	Other postinfectious arthropathies in diseases classified elsewhere
M0325	Other postinfectious arthropathies in diseases classified elsewhere
M0326	Other postinfectious arthropathies in diseases classified elsewhere
M0327	Other postinfectious arthropathies in diseases classified elsewhere
M0328	Other postinfectious arthropathies in diseases classified elsewhere
M0329	Other postinfectious arthropathies in diseases classified elsewhere
M036	Reactive arthropathy in other diseases classified elsewhere
M0360	Reactive arthropathy in other diseases classified elsewhere
M0361	Reactive arthropathy in other diseases classified elsewhere
M0362	Reactive arthropathy in other diseases classified elsewhere
M0363	Reactive arthropathy in other diseases classified elsewhere
M0364	Reactive arthropathy in other diseases classified elsewhere
M0365	Reactive arthropathy in other diseases classified elsewhere
M0366	Reactive arthropathy in other diseases classified elsewhere
M0367	Reactive arthropathy in other diseases classified elsewhere
M0368	Reactive arthropathy in other diseases classified elsewhere
M0369	Reactive arthropathy in other diseases classified elsewhere
M05	Seropositive rheumatoid arthritis
M050	Felty syndrome
M050	Felty's syndrome
M0500	Felty syndrome
M0500	Felty's syndrome
M0501	Felty syndrome
M0501	Felty's syndrome
M0502	Felty syndrome
M0502	Felty's syndrome
M0503	Felty syndrome
M0503	Felty's syndrome
M0504	Felty syndrome
M0504	Felty's syndrome
M0505	Felty syndrome
M0505	Felty's syndrome
M0506	Felty syndrome
M0506	Felty's syndrome
M0507	Felty syndrome
M0507	Felty's syndrome
M0508	Felty syndrome
M0508	Felty's syndrome
M0509	Felty syndrome
M0509	Felty's syndrome

For peer review only

M051	Rheumatoid lung disease
M0510	Rheumatoid lung disease
M0511	Rheumatoid lung disease
M0512	Rheumatoid lung disease
M0513	Rheumatoid lung disease
M0514	Rheumatoid lung disease
M0515	Rheumatoid lung disease
M0516	Rheumatoid lung disease
M0517	Rheumatoid lung disease
M0518	Rheumatoid lung disease
M0519	Rheumatoid lung disease
M052	Rheumatoid vasculitis
M0520	Rheumatoid vasculitis
M0521	Rheumatoid vasculitis
M0522	Rheumatoid vasculitis
M0523	Rheumatoid vasculitis
M0524	Rheumatoid vasculitis
M0525	Rheumatoid vasculitis
M0526	Rheumatoid vasculitis
M0527	Rheumatoid vasculitis
M0528	Rheumatoid vasculitis
M0529	Rheumatoid vasculitis
M053	Rheumatoid arthritis with involvement of other organs and systems
M0530	Rheumatoid arthritis with involvement of other organs and systems
M0531	Rheumatoid arthritis with involvement of other organs and systems
M0532	Rheumatoid arthritis with involvement of other organs and systems
M0533	Rheumatoid arthritis with involvement of other organs and systems
M0534	Rheumatoid arthritis with involvement of other organs and systems
M0535	Rheumatoid arthritis with involvement of other organs and systems
M0536	Rheumatoid arthritis with involvement of other organs and systems
M0537	Rheumatoid arthritis with involvement of other organs and systems
M0538	Rheumatoid arthritis with involvement of other organs and systems
M0539	Rheumatoid arthritis with involvement of other organs and systems
M058	Other seropositive rheumatoid arthritis
M0580	Other seropositive rheumatoid arthritis
M0581	Other seropositive rheumatoid arthritis
M0582	Other seropositive rheumatoid arthritis
M0583	Other seropositive rheumatoid arthritis
M0584	Other seropositive rheumatoid arthritis
M0585	Other seropositive rheumatoid arthritis
M0586	Other seropositive rheumatoid arthritis
M0587	Other seropositive rheumatoid arthritis
M0588	Other seropositive rheumatoid arthritis
M0589	Other seropositive rheumatoid arthritis
M059	Seropositive rheumatoid arthritis, unspecified
M0590	Seropositive rheumatoid arthritis, unspecified
M0591	Seropositive rheumatoid arthritis, unspecified
M0592	Seropositive rheumatoid arthritis, unspecified

For peer review only

M0593	Seropositive rheumatoid arthritis, unspecified
M0594	Seropositive rheumatoid arthritis, unspecified
M0595	Seropositive rheumatoid arthritis, unspecified
M0596	Seropositive rheumatoid arthritis, unspecified
M0597	Seropositive rheumatoid arthritis, unspecified
M0598	Seropositive rheumatoid arthritis, unspecified
M0599	Seropositive rheumatoid arthritis, unspecified
M06	Other rheumatoid arthritis
M060	Seronegative rheumatoid arthritis
M0600	Seronegative rheumatoid arthritis
M0601	Seronegative rheumatoid arthritis
M0602	Seronegative rheumatoid arthritis
M0603	Seronegative rheumatoid arthritis
M0604	Seronegative rheumatoid arthritis
M0605	Seronegative rheumatoid arthritis
M0606	Seronegative rheumatoid arthritis
M0607	Seronegative rheumatoid arthritis
M0608	Seronegative rheumatoid arthritis
M0609	Seronegative rheumatoid arthritis
M061	Adult-onset Still disease
M061	Adult-onset Still's disease
M0610	Adult-onset Still disease
M0610	Adult-onset Still's disease
M0611	Adult-onset Still disease
M0611	Adult-onset Still's disease
M0612	Adult-onset Still disease
M0612	Adult-onset Still's disease
M0613	Adult-onset Still disease
M0613	Adult-onset Still's disease
M0614	Adult-onset Still disease
M0614	Adult-onset Still's disease
M0615	Adult-onset Still disease
M0615	Adult-onset Still's disease
M0616	Adult-onset Still disease
M0616	Adult-onset Still's disease
M0617	Adult-onset Still disease
M0617	Adult-onset Still's disease
M0618	Adult-onset Still disease
M0618	Adult-onset Still's disease
M0619	Adult-onset Still disease
M0619	Adult-onset Still's disease
M062	Rheumatoid bursitis
M0620	Rheumatoid bursitis
M0621	Rheumatoid bursitis
M0622	Rheumatoid bursitis
M0623	Rheumatoid bursitis
M0624	Rheumatoid bursitis
M0625	Rheumatoid bursitis

For peer review only

M0626	Rheumatoid bursitis
M0627	Rheumatoid bursitis
M0628	Rheumatoid bursitis
M0629	Rheumatoid bursitis
M063	Rheumatoid nodule
M0630	Rheumatoid nodule
M0631	Rheumatoid nodule
M0632	Rheumatoid nodule
M0633	Rheumatoid nodule
M0634	Rheumatoid nodule
M0635	Rheumatoid nodule
M0636	Rheumatoid nodule
M0637	Rheumatoid nodule
M0638	Rheumatoid nodule
M0639	Rheumatoid nodule
M064	Inflammatory polyarthropathy
M0640	Inflammatory polyarthropathy
M0641	Inflammatory polyarthropathy
M0642	Inflammatory polyarthropathy
M0643	Inflammatory polyarthropathy
M0644	Inflammatory polyarthropathy
M0645	Inflammatory polyarthropathy
M0646	Inflammatory polyarthropathy
M0647	Inflammatory polyarthropathy
M0648	Inflammatory polyarthropathy
M0649	Inflammatory polyarthropathy
M068	Other specified rheumatoid arthritis
M0680	Other specified rheumatoid arthritis
M0681	Other specified rheumatoid arthritis
M0682	Other specified rheumatoid arthritis
M0683	Other specified rheumatoid arthritis
M0684	Other specified rheumatoid arthritis
M0685	Other specified rheumatoid arthritis
M0686	Other specified rheumatoid arthritis
M0687	Other specified rheumatoid arthritis
M0688	Other specified rheumatoid arthritis
M0689	Other specified rheumatoid arthritis
M069	Rheumatoid arthritis, unspecified
M0690	Rheumatoid arthritis, unspecified
M0691	Rheumatoid arthritis, unspecified
M0692	Rheumatoid arthritis, unspecified
M0693	Rheumatoid arthritis, unspecified
M0694	Rheumatoid arthritis, unspecified
M0695	Rheumatoid arthritis, unspecified
M0696	Rheumatoid arthritis, unspecified
M0697	Rheumatoid arthritis, unspecified
M0698	Rheumatoid arthritis, unspecified
M0699	Rheumatoid arthritis, unspecified

For peer review only

M07	Psoriatic and enteropathic arthropathies
M070	Distal interphalangeal psoriatic arthropathy
M0700	Distal interphalangeal psoriatic arthropathy
M0704	Distal interphalangeal psoriatic arthropathy
M0707	Distal interphalangeal psoriatic arthropathy
M0709	Distal interphalangeal psoriatic arthropathy
M071	Arthritis mutilans
M0710	Arthritis mutilans
M0711	Arthritis mutilans
M0712	Arthritis mutilans
M0713	Arthritis mutilans
M0714	Arthritis mutilans
M0715	Arthritis mutilans
M0716	Arthritis mutilans
M0717	Arthritis mutilans
M0718	Arthritis mutilans
M0719	Arthritis mutilans
M072	Psoriatic spondylitis
M0720	Psoriatic spondylitis
M0721	Psoriatic spondylitis
M0722	Psoriatic spondylitis
M0723	Psoriatic spondylitis
M0724	Psoriatic spondylitis
M0725	Psoriatic spondylitis
M0726	Psoriatic spondylitis
M0727	Psoriatic spondylitis
M0728	Psoriatic spondylitis
M0729	Psoriatic spondylitis
M073	Other psoriatic arthropathies
M0730	Other psoriatic arthropathies
M0731	Other psoriatic arthropathies
M0732	Other psoriatic arthropathies
M0733	Other psoriatic arthropathies
M0734	Other psoriatic arthropathies
M0735	Other psoriatic arthropathies
M0736	Other psoriatic arthropathies
M0737	Other psoriatic arthropathies
M0738	Other psoriatic arthropathies
M0739	Other psoriatic arthropathies
M074	Arthropathy in Crohn disease [regional enteritis]
M074	Arthropathy in Crohn's disease [regional enteritis]
M0740	Arthropathy in Crohn disease [regional enteritis]
M0740	Arthropathy in Crohn's disease [regional enteritis]
M0741	Arthropathy in Crohn disease [regional enteritis]
M0741	Arthropathy in Crohn's disease [regional enteritis]
M0742	Arthropathy in Crohn disease [regional enteritis]
M0742	Arthropathy in Crohn's disease [regional enteritis]
M0743	Arthropathy in Crohn disease [regional enteritis]

For peer review only

M0743	Arthropathy in Crohn's disease [regional enteritis]
M0744	Arthropathy in Crohn disease [regional enteritis]
M0744	Arthropathy in Crohn's disease [regional enteritis]
M0745	Arthropathy in Crohn disease [regional enteritis]
M0745	Arthropathy in Crohn's disease [regional enteritis]
M0746	Arthropathy in Crohn disease [regional enteritis]
M0746	Arthropathy in Crohn's disease [regional enteritis]
M0747	Arthropathy in Crohn disease [regional enteritis]
M0747	Arthropathy in Crohn's disease [regional enteritis]
M0748	Arthropathy in Crohn disease [regional enteritis]
M0748	Arthropathy in Crohn's disease [regional enteritis]
M0749	Arthropathy in Crohn disease [regional enteritis]
M0749	Arthropathy in Crohn's disease [regional enteritis]
M075	Arthropathy in ulcerative colitis
M0750	Arthropathy in ulcerative colitis
M0751	Arthropathy in ulcerative colitis
M0752	Arthropathy in ulcerative colitis
M0753	Arthropathy in ulcerative colitis
M0754	Arthropathy in ulcerative colitis
M0755	Arthropathy in ulcerative colitis
M0756	Arthropathy in ulcerative colitis
M0757	Arthropathy in ulcerative colitis
M0758	Arthropathy in ulcerative colitis
M0759	Arthropathy in ulcerative colitis
M076	Other enteropathic arthropathies
M0760	Other enteropathic arthropathies
M0761	Other enteropathic arthropathies
M0762	Other enteropathic arthropathies
M0763	Other enteropathic arthropathies
M0764	Other enteropathic arthropathies
M0765	Other enteropathic arthropathies
M0766	Other enteropathic arthropathies
M0767	Other enteropathic arthropathies
M0768	Other enteropathic arthropathies
M0769	Other enteropathic arthropathies
M08	Juvenile arthritis
M080	Juvenile rheumatoid arthritis
M0800	Juvenile rheumatoid arthritis
M0801	Juvenile rheumatoid arthritis
M0802	Juvenile rheumatoid arthritis
M0803	Juvenile rheumatoid arthritis
M0804	Juvenile rheumatoid arthritis
M0805	Juvenile rheumatoid arthritis
M0806	Juvenile rheumatoid arthritis
M0807	Juvenile rheumatoid arthritis
M0808	Juvenile rheumatoid arthritis
M0809	Juvenile rheumatoid arthritis
M081	Juvenile ankylosing spondylitis

For peer review only

M0810	Juvenile ankylosing spondylitis
M0811	Juvenile ankylosing spondylitis
M0812	Juvenile ankylosing spondylitis
M0813	Juvenile ankylosing spondylitis
M0814	Juvenile ankylosing spondylitis
M0815	Juvenile ankylosing spondylitis
M0816	Juvenile ankylosing spondylitis
M0817	Juvenile ankylosing spondylitis
M0818	Juvenile ankylosing spondylitis
M0819	Juvenile ankylosing spondylitis
M082	Juvenile arthritis with systemic onset
M0820	Juvenile arthritis with systemic onset
M0821	Juvenile arthritis with systemic onset
M0822	Juvenile arthritis with systemic onset
M0823	Juvenile arthritis with systemic onset
M0824	Juvenile arthritis with systemic onset
M0825	Juvenile arthritis with systemic onset
M0826	Juvenile arthritis with systemic onset
M0827	Juvenile arthritis with systemic onset
M0828	Juvenile arthritis with systemic onset
M0829	Juvenile arthritis with systemic onset
M083	Juvenile polyarthritis (seronegative)
M0830	Juvenile polyarthritis (seronegative)
M0831	Juvenile polyarthritis (seronegative)
M0832	Juvenile polyarthritis (seronegative)
M0833	Juvenile polyarthritis (seronegative)
M0834	Juvenile polyarthritis (seronegative)
M0835	Juvenile polyarthritis (seronegative)
M0836	Juvenile polyarthritis (seronegative)
M0837	Juvenile polyarthritis (seronegative)
M0838	Juvenile polyarthritis (seronegative)
M0839	Juvenile polyarthritis (seronegative)
M084	Pauciarticular juvenile arthritis
M0840	Pauciarticular juvenile arthritis
M0841	Pauciarticular juvenile arthritis
M0842	Pauciarticular juvenile arthritis
M0843	Pauciarticular juvenile arthritis
M0844	Pauciarticular juvenile arthritis
M0845	Pauciarticular juvenile arthritis
M0846	Pauciarticular juvenile arthritis
M0847	Pauciarticular juvenile arthritis
M0848	Pauciarticular juvenile arthritis
M0849	Pauciarticular juvenile arthritis
M088	Other juvenile arthritis
M0880	Other juvenile arthritis
M0881	Other juvenile arthritis
M0882	Other juvenile arthritis
M0883	Other juvenile arthritis

For peer review only

M0884	Other juvenile arthritis
M0885	Other juvenile arthritis
M0886	Other juvenile arthritis
M0887	Other juvenile arthritis
M0888	Other juvenile arthritis
M0889	Other juvenile arthritis
M089	Juvenile arthritis, unspecified
M0890	Juvenile arthritis, unspecified
M0891	Juvenile arthritis, unspecified
M0892	Juvenile arthritis, unspecified
M0893	Juvenile arthritis, unspecified
M0894	Juvenile arthritis, unspecified
M0895	Juvenile arthritis, unspecified
M0896	Juvenile arthritis, unspecified
M0897	Juvenile arthritis, unspecified
M0898	Juvenile arthritis, unspecified
M0899	Juvenile arthritis, unspecified
M09	Juvenile arthritis in diseases classified elsewhere
M090	Juvenile arthritis in psoriasis
M0900	Juvenile arthritis in psoriasis
M0901	Juvenile arthritis in psoriasis
M0902	Juvenile arthritis in psoriasis
M0903	Juvenile arthritis in psoriasis
M0904	Juvenile arthritis in psoriasis
M0905	Juvenile arthritis in psoriasis
M0906	Juvenile arthritis in psoriasis
M0907	Juvenile arthritis in psoriasis
M0908	Juvenile arthritis in psoriasis
M0909	Juvenile arthritis in psoriasis
M091	Juvenile arthritis in Crohn disease [regional enteritis]
M091	Juvenile arthritis in Crohn's disease [regional enteritis]
M0910	Juvenile arthritis in Crohn disease [regional enteritis]
M0910	Juvenile arthritis in Crohn's disease [regional enteritis]
M0911	Juvenile arthritis in Crohn disease [regional enteritis]
M0911	Juvenile arthritis in Crohn's disease [regional enteritis]
M0912	Juvenile arthritis in Crohn disease [regional enteritis]
M0912	Juvenile arthritis in Crohn's disease [regional enteritis]
M0913	Juvenile arthritis in Crohn disease [regional enteritis]
M0913	Juvenile arthritis in Crohn's disease [regional enteritis]
M0914	Juvenile arthritis in Crohn disease [regional enteritis]
M0914	Juvenile arthritis in Crohn's disease [regional enteritis]
M0915	Juvenile arthritis in Crohn disease [regional enteritis]
M0915	Juvenile arthritis in Crohn's disease [regional enteritis]
M0916	Juvenile arthritis in Crohn disease [regional enteritis]
M0916	Juvenile arthritis in Crohn's disease [regional enteritis]
M0917	Juvenile arthritis in Crohn disease [regional enteritis]
M0917	Juvenile arthritis in Crohn's disease [regional enteritis]
M0918	Juvenile arthritis in Crohn disease [regional enteritis]

For peer review only

M0918	Juvenile arthritis in Crohn's disease [regional enteritis]
M0919	Juvenile arthritis in Crohn disease [regional enteritis]
M0919	Juvenile arthritis in Crohn's disease [regional enteritis]
M092	Juvenile arthritis in ulcerative colitis
M0920	Juvenile arthritis in ulcerative colitis
M0921	Juvenile arthritis in ulcerative colitis
M0922	Juvenile arthritis in ulcerative colitis
M0923	Juvenile arthritis in ulcerative colitis
M0924	Juvenile arthritis in ulcerative colitis
M0925	Juvenile arthritis in ulcerative colitis
M0926	Juvenile arthritis in ulcerative colitis
M0927	Juvenile arthritis in ulcerative colitis
M0928	Juvenile arthritis in ulcerative colitis
M0929	Juvenile arthritis in ulcerative colitis
M098	Juvenile arthritis in other diseases classified elsewhere
M0980	Juvenile arthritis in other diseases classified elsewhere
M0981	Juvenile arthritis in other diseases classified elsewhere
M0982	Juvenile arthritis in other diseases classified elsewhere
M0983	Juvenile arthritis in other diseases classified elsewhere
M0984	Juvenile arthritis in other diseases classified elsewhere
M0985	Juvenile arthritis in other diseases classified elsewhere
M0986	Juvenile arthritis in other diseases classified elsewhere
M0987	Juvenile arthritis in other diseases classified elsewhere
M0988	Juvenile arthritis in other diseases classified elsewhere
M0989	Juvenile arthritis in other diseases classified elsewhere
M10	Gout
M100	Idiopathic gout
M1000	Idiopathic gout
M1001	Idiopathic gout
M1002	Idiopathic gout
M1003	Idiopathic gout
M1004	Idiopathic gout
M1005	Idiopathic gout
M1006	Idiopathic gout
M1007	Idiopathic gout
M1008	Idiopathic gout
M1009	Idiopathic gout
M101	Lead-induced gout
M1010	Lead-induced gout
M1011	Lead-induced gout
M1012	Lead-induced gout
M1013	Lead-induced gout
M1014	Lead-induced gout
M1015	Lead-induced gout
M1016	Lead-induced gout
M1017	Lead-induced gout
M1018	Lead-induced gout
M1019	Lead-induced gout

For peer review only

M102	Drug-induced gout
M1020	Drug-induced gout
M1021	Drug-induced gout
M1022	Drug-induced gout
M1023	Drug-induced gout
M1024	Drug-induced gout
M1025	Drug-induced gout
M1026	Drug-induced gout
M1027	Drug-induced gout
M1028	Drug-induced gout
M1029	Drug-induced gout
M103	Gout due to impairment of renal function
M1030	Gout due to impairment of renal function
M1031	Gout due to impairment of renal function
M1032	Gout due to impairment of renal function
M1033	Gout due to impairment of renal function
M1034	Gout due to impairment of renal function
M1035	Gout due to impairment of renal function
M1036	Gout due to impairment of renal function
M1037	Gout due to impairment of renal function
M1038	Gout due to impairment of renal function
M1039	Gout due to impairment of renal function
M104	Other secondary gout
M1040	Other secondary gout
M1041	Other secondary gout
M1042	Other secondary gout
M1043	Other secondary gout
M1044	Other secondary gout
M1045	Other secondary gout
M1046	Other secondary gout
M1047	Other secondary gout
M1048	Other secondary gout
M1049	Other secondary gout
M109	Gout, unspecified
M1090	Gout, unspecified
M1091	Gout, unspecified
M1092	Gout, unspecified
M1093	Gout, unspecified
M1094	Gout, unspecified
M1095	Gout, unspecified
M1096	Gout, unspecified
M1097	Gout, unspecified
M1098	Gout, unspecified
M1099	Gout, unspecified
M11	Other crystal arthropathies
M110	Hydroxyapatite deposition disease
M1100	Hydroxyapatite deposition disease
M1101	Hydroxyapatite deposition disease

For peer review only

M1102	Hydroxyapatite deposition disease
M1103	Hydroxyapatite deposition disease
M1104	Hydroxyapatite deposition disease
M1105	Hydroxyapatite deposition disease
M1106	Hydroxyapatite deposition disease
M1107	Hydroxyapatite deposition disease
M1108	Hydroxyapatite deposition disease
M1109	Hydroxyapatite deposition disease
M111	Familial chondrocalcinosis
M1110	Familial chondrocalcinosis
M1111	Familial chondrocalcinosis
M1112	Familial chondrocalcinosis
M1113	Familial chondrocalcinosis
M1114	Familial chondrocalcinosis
M1115	Familial chondrocalcinosis
M1116	Familial chondrocalcinosis
M1117	Familial chondrocalcinosis
M1118	Familial chondrocalcinosis
M1119	Familial chondrocalcinosis
M112	Other chondrocalcinosis
M1120	Other chondrocalcinosis
M1121	Other chondrocalcinosis
M1122	Other chondrocalcinosis
M1123	Other chondrocalcinosis
M1124	Other chondrocalcinosis
M1125	Other chondrocalcinosis
M1126	Other chondrocalcinosis
M1127	Other chondrocalcinosis
M1128	Other chondrocalcinosis
M1129	Other chondrocalcinosis
M118	Other specified crystal arthropathies
M1180	Other specified crystal arthropathies
M1181	Other specified crystal arthropathies
M1182	Other specified crystal arthropathies
M1183	Other specified crystal arthropathies
M1184	Other specified crystal arthropathies
M1185	Other specified crystal arthropathies
M1186	Other specified crystal arthropathies
M1187	Other specified crystal arthropathies
M1188	Other specified crystal arthropathies
M1189	Other specified crystal arthropathies
M119	Crystal arthropathy, unspecified
M1190	Crystal arthropathy, unspecified
M1191	Crystal arthropathy, unspecified
M1192	Crystal arthropathy, unspecified
M1193	Crystal arthropathy, unspecified
M1194	Crystal arthropathy, unspecified
M1195	Crystal arthropathy, unspecified

For peer review only

M1196	Crystal arthropathy, unspecified
M1197	Crystal arthropathy, unspecified
M1198	Crystal arthropathy, unspecified
M1199	Crystal arthropathy, unspecified
M12	Other specific arthropathies
M120	Chronic postrheumatic arthropathy [Jaccoud]
M1200	Chronic postrheumatic arthropathy [Jaccoud]
M1201	Chronic postrheumatic arthropathy [Jaccoud]
M1202	Chronic postrheumatic arthropathy [Jaccoud]
M1203	Chronic postrheumatic arthropathy [Jaccoud]
M1204	Chronic postrheumatic arthropathy [Jaccoud]
M1205	Chronic postrheumatic arthropathy [Jaccoud]
M1206	Chronic postrheumatic arthropathy [Jaccoud]
M1207	Chronic postrheumatic arthropathy [Jaccoud]
M1208	Chronic postrheumatic arthropathy [Jaccoud]
M1209	Chronic postrheumatic arthropathy [Jaccoud]
M121	Kaschin-Beck disease
M1210	Kaschin-Beck disease
M1211	Kaschin-Beck disease
M1212	Kaschin-Beck disease
M1213	Kaschin-Beck disease
M1214	Kaschin-Beck disease
M1215	Kaschin-Beck disease
M1216	Kaschin-Beck disease
M1217	Kaschin-Beck disease
M1218	Kaschin-Beck disease
M1219	Kaschin-Beck disease
M122	Villonodular synovitis (pigmented)
M1220	Villonodular synovitis (pigmented)
M1221	Villonodular synovitis (pigmented)
M1222	Villonodular synovitis (pigmented)
M1223	Villonodular synovitis (pigmented)
M1224	Villonodular synovitis (pigmented)
M1225	Villonodular synovitis (pigmented)
M1226	Villonodular synovitis (pigmented)
M1227	Villonodular synovitis (pigmented)
M1228	Villonodular synovitis (pigmented)
M1229	Villonodular synovitis (pigmented)
M123	Palindromic rheumatism
M1230	Palindromic rheumatism
M1231	Palindromic rheumatism
M1232	Palindromic rheumatism
M1233	Palindromic rheumatism
M1234	Palindromic rheumatism
M1235	Palindromic rheumatism
M1236	Palindromic rheumatism
M1237	Palindromic rheumatism
M1238	Palindromic rheumatism

For peer review only

M1239	Palindromic rheumatism
M124	Intermittent hydrarthrosis
M1240	Intermittent hydrarthrosis
M1241	Intermittent hydrarthrosis
M1242	Intermittent hydrarthrosis
M1243	Intermittent hydrarthrosis
M1244	Intermittent hydrarthrosis
M1245	Intermittent hydrarthrosis
M1246	Intermittent hydrarthrosis
M1247	Intermittent hydrarthrosis
M1248	Intermittent hydrarthrosis
M1249	Intermittent hydrarthrosis
M125	Traumatic arthropathy
M1250	Traumatic arthropathy
M1251	Traumatic arthropathy
M1252	Traumatic arthropathy
M1253	Traumatic arthropathy
M1254	Traumatic arthropathy
M1255	Traumatic arthropathy
M1256	Traumatic arthropathy
M1257	Traumatic arthropathy
M1258	Traumatic arthropathy
M1259	Traumatic arthropathy
M128	Other specific arthropathies, not elsewhere classified
M1280	Other specific arthropathies, not elsewhere classified
M1281	Other specific arthropathies, not elsewhere classified
M1282	Other specific arthropathies, not elsewhere classified
M1283	Other specific arthropathies, not elsewhere classified
M1284	Other specific arthropathies, not elsewhere classified
M1285	Other specific arthropathies, not elsewhere classified
M1286	Other specific arthropathies, not elsewhere classified
M1287	Other specific arthropathies, not elsewhere classified
M1288	Other specific arthropathies, not elsewhere classified
M1289	Other specific arthropathies, not elsewhere classified
M13	Other arthritis
M130	Polyarthritis, unspecified
M1300	Polyarthritis, unspecified
M1301	Polyarthritis, unspecified
M1302	Polyarthritis, unspecified
M1303	Polyarthritis, unspecified
M1304	Polyarthritis, unspecified
M1305	Polyarthritis, unspecified
M1306	Polyarthritis, unspecified
M1307	Polyarthritis, unspecified
M1308	Polyarthritis, unspecified
M1309	Polyarthritis, unspecified
M131	Monoarthritis, not elsewhere classified
M1310	Monoarthritis, not elsewhere classified

For peer review only

M1311	Monoarthritis, not elsewhere classified
M1312	Monoarthritis, not elsewhere classified
M1313	Monoarthritis, not elsewhere classified
M1314	Monoarthritis, not elsewhere classified
M1315	Monoarthritis, not elsewhere classified
M1316	Monoarthritis, not elsewhere classified
M1317	Monoarthritis, not elsewhere classified
M1318	Monoarthritis, not elsewhere classified
M1319	Monoarthritis, not elsewhere classified
M138	Other specified arthritis
M1380	Other specified arthritis
M1381	Other specified arthritis
M1382	Other specified arthritis
M1383	Other specified arthritis
M1384	Other specified arthritis
M1385	Other specified arthritis
M1386	Other specified arthritis
M1387	Other specified arthritis
M1388	Other specified arthritis
M1389	Other specified arthritis
M139	Arthritis, unspecified
M1390	Arthritis, unspecified
M1391	Arthritis, unspecified
M1392	Arthritis, unspecified
M1393	Arthritis, unspecified
M1394	Arthritis, unspecified
M1395	Arthritis, unspecified
M1396	Arthritis, unspecified
M1397	Arthritis, unspecified
M1398	Arthritis, unspecified
M1399	Arthritis, unspecified
M14	Arthropathies in other diseases classified elsewhere
M140	Gouty arthropathy due to enzyme defects and other inherited disorders
M141	Crystal arthropathy in other metabolic disorders
M142	Diabetic arthropathy
M143	Lipoid dermatoarthritis
M144	Arthropathy in amyloidosis
M145	Arthropathies in other endocrine, nutritional and metabolic disorders
M146	Neuropathic arthropathy
M148	Arthropathies in other specified diseases classified elsewhere
M15	Polyarthrosis
M150	Primary generalized (osteo)arthrosis
M151	Heberden nodes (with arthropathy)
M151	Heberden's nodes (with arthropathy)
M152	Bouchard nodes (with arthropathy)
M152	Bouchard's nodes (with arthropathy)
M153	Secondary multiple arthrosis
M154	Erosive (osteo)arthrosis

For peer review only

M158	Other polyarthrosis
M159	Polyarthrosis, unspecified
M16	Coxarthrosis [arthrosis of hip]
M160	Primary coxarthrosis, bilateral
M161	Other primary coxarthrosis
M162	Coxarthrosis resulting from dysplasia, bilateral
M163	Other dysplastic coxarthrosis
M164	Post-traumatic coxarthrosis, bilateral
M165	Other post-traumatic coxarthrosis
M166	Other secondary coxarthrosis, bilateral
M167	Other secondary coxarthrosis
M169	Coxarthrosis, unspecified
M17	Gonarthrosis [arthrosis of knee]
M170	Primary gonarthrosis, bilateral
M171	Other primary gonarthrosis
M172	Post-traumatic gonarthrosis, bilateral
M173	Other post-traumatic gonarthrosis
M174	Other secondary gonarthrosis, bilateral
M175	Other secondary gonarthrosis
M179	Gonarthrosis, unspecified
M18	Arthrosis of first carpometacarpal joint
M180	Primary arthrosis of first carpometacarpal joints, bilateral
M181	Other primary arthrosis of first carpometacarpal joint
M182	Post-traumatic arthrosis of first carpometacarpal joints, bilateral
M183	Other post-traumatic arthrosis of first carpometacarpal joint
M184	Other secondary arthrosis of first carpometacarpal joints, bilateral
M185	Other secondary arthrosis of first carpometacarpal joint
M189	Arthrosis of first carpometacarpal joint, unspecified
M19	Other arthrosis
M190	Primary arthrosis of other joints
M1900	Primary arthrosis of other joints
M1901	Primary arthrosis of other joints
M1902	Primary arthrosis of other joints
M1903	Primary arthrosis of other joints
M1904	Primary arthrosis of other joints
M1905	Primary arthrosis of other joints
M1907	Primary arthrosis of other joints
M1908	Primary arthrosis of other joints
M1909	Primary arthrosis of other joints
M191	Post-traumatic arthrosis of other joints
M1910	Post-traumatic arthrosis of other joints
M1911	Post-traumatic arthrosis of other joints
M1912	Post-traumatic arthrosis of other joints
M1913	Post-traumatic arthrosis of other joints
M1914	Post-traumatic arthrosis of other joints
M1915	Post-traumatic arthrosis of other joints
M1917	Post-traumatic arthrosis of other joints
M1918	Post-traumatic arthrosis of other joints

For peer review only

M1919	Post-traumatic arthrosis of other joints
M192	Other secondary arthrosis
M1920	Other secondary arthrosis
M1921	Other secondary arthrosis
M1922	Other secondary arthrosis
M1923	Other secondary arthrosis
M1924	Other secondary arthrosis
M1925	Other secondary arthrosis
M1927	Other secondary arthrosis
M1928	Other secondary arthrosis
M1929	Other secondary arthrosis
M198	Other specified arthrosis
M1980	Other specified arthrosis
M1981	Other specified arthrosis
M1982	Other specified arthrosis
M1983	Other specified arthrosis
M1984	Other specified arthrosis
M1985	Other specified arthrosis
M1986	Other specified arthrosis
M1987	Other specified arthrosis
M1988	Other specified arthrosis
M1989	Other specified arthrosis
M199	Arthrosis, unspecified
M1990	Arthrosis, unspecified
M1991	Arthrosis, unspecified
M1992	Arthrosis, unspecified
M1993	Arthrosis, unspecified
M1994	Arthrosis, unspecified
M1995	Arthrosis, unspecified
M1997	Arthrosis, unspecified
M1998	Arthrosis, unspecified
M1999	Arthrosis, unspecified
M20	Acquired deformities of fingers and toes
M200	Deformity of finger(s)
M201	Hallux valgus (acquired)
M202	Hallux rigidus
M203	Other deformity of hallux (acquired)
M204	Other hammer toe(s) (acquired)
M205	Other deformities of toe(s) (acquired)
M206	Acquired deformity of toe(s), unspecified
M21	Other acquired deformities of limbs
M210	Valgus deformity, not elsewhere classified
M2100	Valgus deformity, not elsewhere classified
M2101	Valgus deformity, not elsewhere classified
M2102	Valgus deformity, not elsewhere classified
M2103	Valgus deformity, not elsewhere classified
M2104	Valgus deformity, not elsewhere classified
M2105	Valgus deformity, not elsewhere classified

For peer review only

M2106	Valgus deformity, not elsewhere classified
M2107	Valgus deformity, not elsewhere classified
M2108	Valgus deformity, not elsewhere classified
M2109	Valgus deformity, not elsewhere classified
M211	Varus deformity, not elsewhere classified
M2110	Varus deformity, not elsewhere classified
M2111	Varus deformity, not elsewhere classified
M2112	Varus deformity, not elsewhere classified
M2113	Varus deformity, not elsewhere classified
M2114	Varus deformity, not elsewhere classified
M2115	Varus deformity, not elsewhere classified
M2116	Varus deformity, not elsewhere classified
M2117	Varus deformity, not elsewhere classified
M2118	Varus deformity, not elsewhere classified
M2119	Varus deformity, not elsewhere classified
M212	Flexion deformity
M2120	Flexion deformity
M2121	Flexion deformity
M2122	Flexion deformity
M2123	Flexion deformity
M2124	Flexion deformity
M2125	Flexion deformity
M2126	Flexion deformity
M2127	Flexion deformity
M2128	Flexion deformity
M2129	Flexion deformity
M213	Wrist or foot drop (acquired)
M2130	Wrist or foot drop (acquired)
M2133	Wrist or foot drop (acquired)
M2134	Wrist or foot drop (acquired)
M2137	Wrist or foot drop (acquired)
M214	Flat foot [pes planus] (acquired)
M215	Acquired clawhand, clubhand, clawfoot and clubfoot
M2150	Acquired clawhand, clubhand, clawfoot and clubfoot
M2154	Acquired clawhand, clubhand, clawfoot and clubfoot
M2157	Acquired clawhand, clubhand, clawfoot and clubfoot
M216	Other acquired deformities of ankle and foot
M217	Unequal limb length (acquired)
M2170	Unequal limb length (acquired)
M2172	Unequal limb length (acquired)
M2173	Unequal limb length (acquired)
M2174	Unequal limb length (acquired)
M2175	Unequal limb length (acquired)
M2176	Unequal limb length (acquired)
M2177	Unequal limb length (acquired)
M2179	Unequal limb length (acquired)
M218	Other specified acquired deformities of limbs
M2180	Other specified acquired deformities of limbs

For peer review only

M2181	Other specified acquired deformities of limbs
M2182	Other specified acquired deformities of limbs
M2183	Other specified acquired deformities of limbs
M2184	Other specified acquired deformities of limbs
M2185	Other specified acquired deformities of limbs
M2186	Other specified acquired deformities of limbs
M2187	Other specified acquired deformities of limbs
M2189	Other specified acquired deformities of limbs
M219	Acquired deformity of limb, unspecified
M2190	Acquired deformity of limb, unspecified
M2191	Acquired deformity of limb, unspecified
M2192	Acquired deformity of limb, unspecified
M2193	Acquired deformity of limb, unspecified
M2194	Acquired deformity of limb, unspecified
M2195	Acquired deformity of limb, unspecified
M2196	Acquired deformity of limb, unspecified
M2197	Acquired deformity of limb, unspecified
M2199	Acquired deformity of limb, unspecified
M22	Disorders of patella
M220	Recurrent dislocation of patella
M221	Recurrent subluxation of patella
M222	Patellofemoral disorders
M223	Other derangements of patella
M224	Chondromalacia patellae
M228	Other disorders of patella
M229	Disorder of patella, unspecified
M23	Internal derangement of knee
M230	Cystic meniscus
M2300	Cystic meniscus
M2301	Cystic meniscus
M2302	Cystic meniscus
M2303	Cystic meniscus
M2304	Cystic meniscus
M2305	Cystic meniscus
M2306	Cystic meniscus
M2309	Cystic meniscus
M231	Discoid meniscus (congenital)
M2310	Discoid meniscus (congenital)
M2311	Discoid meniscus (congenital)
M2312	Discoid meniscus (congenital)
M2313	Discoid meniscus (congenital)
M2314	Discoid meniscus (congenital)
M2315	Discoid meniscus (congenital)
M2316	Discoid meniscus (congenital)
M2319	Discoid meniscus (congenital)
M232	Derangement of meniscus due to old tear or injury
M2320	Derangement of meniscus due to old tear or injury
M2321	Derangement of meniscus due to old tear or injury

For peer review only

M2322	Derangement of meniscus due to old tear or injury
M2323	Derangement of meniscus due to old tear or injury
M2324	Derangement of meniscus due to old tear or injury
M2325	Derangement of meniscus due to old tear or injury
M2326	Derangement of meniscus due to old tear or injury
M2329	Derangement of meniscus due to old tear or injury
M233	Other meniscus derangements
M2330	Other meniscus derangements
M2331	Other meniscus derangements
M2332	Other meniscus derangements
M2333	Other meniscus derangements
M2334	Other meniscus derangements
M2335	Other meniscus derangements
M2336	Other meniscus derangements
M2339	Other meniscus derangements
M234	Loose body in knee
M235	Chronic instability of knee
M2350	Chronic instability of knee
M2351	Chronic instability of knee
M2352	Chronic instability of knee
M2353	Chronic instability of knee
M2354	Chronic instability of knee
M2355	Chronic instability of knee
M2356	Chronic instability of knee
M2357	Chronic instability of knee
M2359	Chronic instability of knee
M236	Other spontaneous disruption of ligament(s) of knee
M2360	Other spontaneous disruption of ligament(s) of knee
M2361	Other spontaneous disruption of ligament(s) of knee
M2362	Other spontaneous disruption of ligament(s) of knee
M2363	Other spontaneous disruption of ligament(s) of knee
M2364	Other spontaneous disruption of ligament(s) of knee
M2367	Other spontaneous disruption of ligament(s) of knee
M2369	Other spontaneous disruption of ligament(s) of knee
M238	Other internal derangements of knee
M2380	Other internal derangements of knee
M2381	Other internal derangements of knee
M2382	Other internal derangements of knee
M2383	Other internal derangements of knee
M2384	Other internal derangements of knee
M2385	Other internal derangements of knee
M2386	Other internal derangements of knee
M2387	Other internal derangements of knee
M2389	Other internal derangements of knee
M239	Internal derangement of knee, unspecified
M2390	Internal derangement of knee, unspecified
M2391	Internal derangement of knee, unspecified
M2392	Internal derangement of knee, unspecified

For peer review only

M2393	Internal derangement of knee, unspecified
M2394	Internal derangement of knee, unspecified
M2395	Internal derangement of knee, unspecified
M2396	Internal derangement of knee, unspecified
M2397	Internal derangement of knee, unspecified
M2399	Internal derangement of knee, unspecified
M24	Other specific joint derangements
M240	Loose body in joint
M2400	Loose body in joint
M2401	Loose body in joint
M2402	Loose body in joint
M2403	Loose body in joint
M2404	Loose body in joint
M2405	Loose body in joint
M2407	Loose body in joint
M2408	Loose body in joint
M2409	Loose body in joint
M241	Other articular cartilage disorders
M2410	Other articular cartilage disorders
M2411	Other articular cartilage disorders
M2412	Other articular cartilage disorders
M2413	Other articular cartilage disorders
M2414	Other articular cartilage disorders
M2415	Other articular cartilage disorders
M2416	Other articular cartilage disorders
M2417	Other articular cartilage disorders
M2418	Other articular cartilage disorders
M2419	Other articular cartilage disorders
M242	Disorder of ligament
M2420	Disorder of ligament
M2421	Disorder of ligament
M2422	Disorder of ligament
M2423	Disorder of ligament
M2424	Disorder of ligament
M2425	Disorder of ligament
M2426	Disorder of ligament
M2427	Disorder of ligament
M2428	Disorder of ligament
M2429	Disorder of ligament
M243	Pathological dislocation and subluxation of joint, not elsewhere classified
M2430	Pathological dislocation and subluxation of joint, not elsewhere classified
M2431	Pathological dislocation and subluxation of joint, not elsewhere classified
M2432	Pathological dislocation and subluxation of joint, not elsewhere classified
M2433	Pathological dislocation and subluxation of joint, not elsewhere classified
M2434	Pathological dislocation and subluxation of joint, not elsewhere classified
M2435	Pathological dislocation and subluxation of joint, not elsewhere classified
M2436	Pathological dislocation and subluxation of joint, not elsewhere classified
M2437	Pathological dislocation and subluxation of joint, not elsewhere classified

For peer review only

M2438	Pathological dislocation and subluxation of joint, not elsewhere classified
M2439	Pathological dislocation and subluxation of joint, not elsewhere classified
M244	Recurrent dislocation and subluxation of joint
M2440	Recurrent dislocation and subluxation of joint
M2441	Recurrent dislocation and subluxation of joint
M2442	Recurrent dislocation and subluxation of joint
M2443	Recurrent dislocation and subluxation of joint
M2444	Recurrent dislocation and subluxation of joint
M2445	Recurrent dislocation and subluxation of joint
M2446	Recurrent dislocation and subluxation of joint
M2447	Recurrent dislocation and subluxation of joint
M2448	Recurrent dislocation and subluxation of joint
M2449	Recurrent dislocation and subluxation of joint
M245	Contracture of joint
M2450	Contracture of joint
M2451	Contracture of joint
M2452	Contracture of joint
M2453	Contracture of joint
M2454	Contracture of joint
M2455	Contracture of joint
M2456	Contracture of joint
M2457	Contracture of joint
M2458	Contracture of joint
M2459	Contracture of joint
M246	Ankylosis of joint
M2460	Ankylosis of joint
M2461	Ankylosis of joint
M2462	Ankylosis of joint
M2463	Ankylosis of joint
M2464	Ankylosis of joint
M2465	Ankylosis of joint
M2466	Ankylosis of joint
M2467	Ankylosis of joint
M2468	Ankylosis of joint
M2469	Ankylosis of joint
M247	Protrusio acetabuli
M248	Other specific joint derangements, not elsewhere classified
M2480	Other specific joint derangements, not elsewhere classified
M2481	Other specific joint derangements, not elsewhere classified
M2482	Other specific joint derangements, not elsewhere classified
M2483	Other specific joint derangements, not elsewhere classified
M2484	Other specific joint derangements, not elsewhere classified
M2485	Other specific joint derangements, not elsewhere classified
M2486	Other specific joint derangements, not elsewhere classified
M2487	Other specific joint derangements, not elsewhere classified
M2488	Other specific joint derangements, not elsewhere classified
M2489	Other specific joint derangements, not elsewhere classified
M249	Joint derangement, unspecified

For peer review only

M2490	Joint derangement, unspecified
M2491	Joint derangement, unspecified
M2492	Joint derangement, unspecified
M2493	Joint derangement, unspecified
M2494	Joint derangement, unspecified
M2495	Joint derangement, unspecified
M2496	Joint derangement, unspecified
M2497	Joint derangement, unspecified
M2498	Joint derangement, unspecified
M2499	Joint derangement, unspecified
M25	Other joint disorders, not elsewhere classified
M250	Haemarthrosis
M2500	Haemarthrosis
M2501	Haemarthrosis
M2502	Haemarthrosis
M2503	Haemarthrosis
M2504	Haemarthrosis
M2505	Haemarthrosis
M2506	Haemarthrosis
M2507	Haemarthrosis
M2508	Haemarthrosis
M2509	Haemarthrosis
M251	Fistula of joint
M2510	Fistula of joint
M2511	Fistula of joint
M2512	Fistula of joint
M2513	Fistula of joint
M2514	Fistula of joint
M2515	Fistula of joint
M2516	Fistula of joint
M2517	Fistula of joint
M2518	Fistula of joint
M2519	Fistula of joint
M252	Flail joint
M2520	Flail joint
M2521	Flail joint
M2522	Flail joint
M2523	Flail joint
M2524	Flail joint
M2525	Flail joint
M2526	Flail joint
M2527	Flail joint
M2528	Flail joint
M2529	Flail joint
M253	Other instability of joint
M2530	Other instability of joint
M2531	Other instability of joint
M2532	Other instability of joint

For peer review only

M2533	Other instability of joint
M2534	Other instability of joint
M2535	Other instability of joint
M2536	Other instability of joint
M2537	Other instability of joint
M2538	Other instability of joint
M2539	Other instability of joint
M254	Effusion of joint
M2540	Effusion of joint
M2541	Effusion of joint
M2542	Effusion of joint
M2543	Effusion of joint
M2544	Effusion of joint
M2545	Effusion of joint
M2546	Effusion of joint
M2547	Effusion of joint
M2548	Effusion of joint
M2549	Effusion of joint
M255	Pain in joint
M2550	Pain in joint
M2551	Pain in joint
M2552	Pain in joint
M2553	Pain in joint
M2554	Pain in joint
M2555	Pain in joint
M2556	Pain in joint
M2557	Pain in joint
M2558	Pain in joint
M2559	Pain in joint
M256	Stiffness of joint, not elsewhere classified
M2560	Stiffness of joint, not elsewhere classified
M2561	Stiffness of joint, not elsewhere classified
M2562	Stiffness of joint, not elsewhere classified
M2563	Stiffness of joint, not elsewhere classified
M2564	Stiffness of joint, not elsewhere classified
M2565	Stiffness of joint, not elsewhere classified
M2566	Stiffness of joint, not elsewhere classified
M2567	Stiffness of joint, not elsewhere classified
M2568	Stiffness of joint, not elsewhere classified
M2569	Stiffness of joint, not elsewhere classified
M257	Osteophyte
M2570	Osteophyte
M2571	Osteophyte
M2572	Osteophyte
M2573	Osteophyte
M2574	Osteophyte
M2575	Osteophyte
M2576	Osteophyte

For peer review only

M2577	Osteophyte
M2578	Osteophyte
M2579	Osteophyte
M258	Other specified joint disorders
M2580	Other specified joint disorders
M2581	Other specified joint disorders
M2582	Other specified joint disorders
M2583	Other specified joint disorders
M2584	Other specified joint disorders
M2585	Other specified joint disorders
M2586	Other specified joint disorders
M2587	Other specified joint disorders
M2588	Other specified joint disorders
M2589	Other specified joint disorders
M259	Joint disorder, unspecified
M2590	Joint disorder, unspecified
M2591	Joint disorder, unspecified
M2592	Joint disorder, unspecified
M2593	Joint disorder, unspecified
M2594	Joint disorder, unspecified
M2595	Joint disorder, unspecified
M2596	Joint disorder, unspecified
M2597	Joint disorder, unspecified
M2598	Joint disorder, unspecified
M2599	Joint disorder, unspecified
M30	Polyarteritis nodosa and related conditions
M300	Polyarteritis nodosa
M301	Polyarteritis with lung involvement [Churg-Strauss]
M302	Juvenile polyarteritis
M303	Mucocutaneous lymph node syndrome [Kawasaki]
M308	Other conditions related to polyarteritis nodosa
M31	Other necrotizing vasculopathies
M310	Hypersensitivity angiitis
M311	Thrombotic microangiopathy
M312	Lethal midline granuloma
M313	Wegener granulomatosis
M313	Wegener's granulomatosis
M314	Aortic arch syndrome [Takayasu]
M315	Giant cell arteritis with polymyalgia rheumatica
M316	Other giant cell arteritis
M317	Microscopic polyangiitis
M318	Other specified necrotizing vasculopathies
M319	Necrotizing vasculopathy, unspecified
M32	Systemic lupus erythematosus
M320	Drug-induced systemic lupus erythematosus
M321	Systemic lupus erythematosus with organ or system involvement
M328	Other forms of systemic lupus erythematosus
M329	Systemic lupus erythematosus, unspecified

For peer review only

M33	Dermatopolymyositis
M330	Juvenile dermatomyositis
M331	Other dermatomyositis
M332	Polymyositis
M339	Dermatopolymyositis, unspecified
M34	Systemic sclerosis
M340	Progressive systemic sclerosis
M341	CR(E)ST syndrome
M342	Systemic sclerosis induced by drugs and chemicals
M348	Other forms of systemic sclerosis
M349	Systemic sclerosis, unspecified
M35	Other systemic involvement of connective tissue
M350	Sicca syndrome [Sj”gren]
M351	Other overlap syndromes
M352	Beh‡et disease
M352	Beh‡et's disease
M353	Polymyalgia rheumatica
M354	Diffuse (eosinophilic) fasciitis
M355	Multifocal fibrosclerosis
M356	Relapsing panniculitis [Weber-Christian]
M357	Hypermobility syndrome
M358	Other specified systemic involvement of connective tissue
M359	Systemic involvement of connective tissue, unspecified
M36	Systemic disorders of connective tissue in diseases classified elsewhere
M360	Dermato(poly)myositis in neoplastic disease
M361	Arthropathy in neoplastic disease
M362	Haemophilic arthropathy
M363	Arthropathy in other blood disorders
M364	Arthropathy in hypersensitivity reactions classified elsewhere
M368	Systemic disorders of connective tissue in other diseases classified elsewhere
M40	Kyphosis and lordosis
M400	Postural kyphosis
M4000	Postural kyphosis
M4001	Postural kyphosis
M4002	Postural kyphosis
M4003	Postural kyphosis
M4004	Postural kyphosis
M4005	Postural kyphosis
M4006	Postural kyphosis
M4007	Postural kyphosis
M4008	Postural kyphosis
M4009	Postural kyphosis
M401	Other secondary kyphosis
M4010	Other secondary kyphosis
M4011	Other secondary kyphosis
M4012	Other secondary kyphosis
M4013	Other secondary kyphosis
M4014	Other secondary kyphosis

For peer review only

M4015	Other secondary kyphosis
M4016	Other secondary kyphosis
M4017	Other secondary kyphosis
M4018	Other secondary kyphosis
M4019	Other secondary kyphosis
M402	Other and unspecified kyphosis
M4020	Other and unspecified kyphosis
M4021	Other and unspecified kyphosis
M4022	Other and unspecified kyphosis
M4023	Other and unspecified kyphosis
M4024	Other and unspecified kyphosis
M4025	Other and unspecified kyphosis
M4026	Other and unspecified kyphosis
M4027	Other and unspecified kyphosis
M4028	Other and unspecified kyphosis
M4029	Other and unspecified kyphosis
M403	Flatback syndrome
M4030	Flatback syndrome
M4031	Flatback syndrome
M4032	Flatback syndrome
M4033	Flatback syndrome
M4034	Flatback syndrome
M4035	Flatback syndrome
M4036	Flatback syndrome
M4037	Flatback syndrome
M4038	Flatback syndrome
M4039	Flatback syndrome
M404	Other lordosis
M4040	Other lordosis
M4041	Other lordosis
M4042	Other lordosis
M4043	Other lordosis
M4044	Other lordosis
M4045	Other lordosis
M4046	Other lordosis
M4047	Other lordosis
M4048	Other lordosis
M4049	Other lordosis
M405	Lordosis, unspecified
M4050	Lordosis, unspecified
M4051	Lordosis, unspecified
M4052	Lordosis, unspecified
M4053	Lordosis, unspecified
M4054	Lordosis, unspecified
M4055	Lordosis, unspecified
M4056	Lordosis, unspecified
M4057	Lordosis, unspecified
M4058	Lordosis, unspecified

For peer review only

M4059	Lordosis, unspecified
M41	Scoliosis
M410	Infantile idiopathic scoliosis
M4100	Infantile idiopathic scoliosis
M4101	Infantile idiopathic scoliosis
M4102	Infantile idiopathic scoliosis
M4103	Infantile idiopathic scoliosis
M4104	Infantile idiopathic scoliosis
M4105	Infantile idiopathic scoliosis
M4106	Infantile idiopathic scoliosis
M4107	Infantile idiopathic scoliosis
M4108	Infantile idiopathic scoliosis
M4109	Infantile idiopathic scoliosis
M411	Juvenile idiopathic scoliosis
M4110	Juvenile idiopathic scoliosis
M4111	Juvenile idiopathic scoliosis
M4112	Juvenile idiopathic scoliosis
M4113	Juvenile idiopathic scoliosis
M4114	Juvenile idiopathic scoliosis
M4115	Juvenile idiopathic scoliosis
M4116	Juvenile idiopathic scoliosis
M4117	Juvenile idiopathic scoliosis
M4118	Juvenile idiopathic scoliosis
M4119	Juvenile idiopathic scoliosis
M412	Other idiopathic scoliosis
M4120	Other idiopathic scoliosis
M4121	Other idiopathic scoliosis
M4122	Other idiopathic scoliosis
M4123	Other idiopathic scoliosis
M4124	Other idiopathic scoliosis
M4125	Other idiopathic scoliosis
M4126	Other idiopathic scoliosis
M4127	Other idiopathic scoliosis
M4128	Other idiopathic scoliosis
M4129	Other idiopathic scoliosis
M413	Thoracogenic scoliosis
M4130	Thoracogenic scoliosis
M4133	Thoracogenic scoliosis
M4134	Thoracogenic scoliosis
M4135	Thoracogenic scoliosis
M414	Neuromuscular scoliosis
M4140	Neuromuscular scoliosis
M4141	Neuromuscular scoliosis
M4142	Neuromuscular scoliosis
M4143	Neuromuscular scoliosis
M4144	Neuromuscular scoliosis
M4145	Neuromuscular scoliosis
M4146	Neuromuscular scoliosis

For peer review only

M4147	Neuromuscular scoliosis
M4148	Neuromuscular scoliosis
M4149	Neuromuscular scoliosis
M415	Other secondary scoliosis
M4150	Other secondary scoliosis
M4151	Other secondary scoliosis
M4152	Other secondary scoliosis
M4153	Other secondary scoliosis
M4154	Other secondary scoliosis
M4155	Other secondary scoliosis
M4156	Other secondary scoliosis
M4157	Other secondary scoliosis
M4158	Other secondary scoliosis
M4159	Other secondary scoliosis
M418	Other forms of scoliosis
M4180	Other forms of scoliosis
M4181	Other forms of scoliosis
M4182	Other forms of scoliosis
M4183	Other forms of scoliosis
M4184	Other forms of scoliosis
M4185	Other forms of scoliosis
M4186	Other forms of scoliosis
M4187	Other forms of scoliosis
M4188	Other forms of scoliosis
M4189	Other forms of scoliosis
M419	Scoliosis, unspecified
M4190	Scoliosis, unspecified
M4191	Scoliosis, unspecified
M4192	Scoliosis, unspecified
M4193	Scoliosis, unspecified
M4194	Scoliosis, unspecified
M4195	Scoliosis, unspecified
M4196	Scoliosis, unspecified
M4197	Scoliosis, unspecified
M4198	Scoliosis, unspecified
M4199	Scoliosis, unspecified
M42	Spinal osteochondrosis
M420	Juvenile osteochondrosis of spine
M4200	Juvenile osteochondrosis of spine
M4201	Juvenile osteochondrosis of spine
M4202	Juvenile osteochondrosis of spine
M4203	Juvenile osteochondrosis of spine
M4204	Juvenile osteochondrosis of spine
M4205	Juvenile osteochondrosis of spine
M4206	Juvenile osteochondrosis of spine
M4207	Juvenile osteochondrosis of spine
M4208	Juvenile osteochondrosis of spine
M4209	Juvenile osteochondrosis of spine

For peer review only

For peer review only - http://bmjopen.bmj.com/site/about/guidelines.xhtml

M421	Adult osteochondrosis of spine
M4210	Adult osteochondrosis of spine
M4211	Adult osteochondrosis of spine
M4212	Adult osteochondrosis of spine
M4213	Adult osteochondrosis of spine
M4214	Adult osteochondrosis of spine
M4215	Adult osteochondrosis of spine
M4216	Adult osteochondrosis of spine
M4217	Adult osteochondrosis of spine
M4218	Adult osteochondrosis of spine
M4219	Adult osteochondrosis of spine
M429	Spinal osteochondrosis, unspecified
M4290	Spinal osteochondrosis, unspecified
M4291	Spinal osteochondrosis, unspecified
M4292	Spinal osteochondrosis, unspecified
M4293	Spinal osteochondrosis, unspecified
M4294	Spinal osteochondrosis, unspecified
M4295	Spinal osteochondrosis, unspecified
M4296	Spinal osteochondrosis, unspecified
M4297	Spinal osteochondrosis, unspecified
M4298	Spinal osteochondrosis, unspecified
M4299	Spinal osteochondrosis, unspecified
M43	Other deforming dorsopathies
M430	Spondylolysis
M4300	Spondylolysis
M4301	Spondylolysis
M4302	Spondylolysis
M4303	Spondylolysis
M4304	Spondylolysis
M4305	Spondylolysis
M4306	Spondylolysis
M4307	Spondylolysis
M4308	Spondylolysis
M4309	Spondylolysis
M431	Spondylolisthesis
M4310	Spondylolisthesis
M4311	Spondylolisthesis
M4312	Spondylolisthesis
M4313	Spondylolisthesis
M4314	Spondylolisthesis
M4315	Spondylolisthesis
M4316	Spondylolisthesis
M4317	Spondylolisthesis
M4318	Spondylolisthesis
M4319	Spondylolisthesis
M432	Other fusion of spine
M4320	Other fusion of spine
M4321	Other fusion of spine

For peer review only

M4322	Other fusion of spine
M4323	Other fusion of spine
M4324	Other fusion of spine
M4325	Other fusion of spine
M4326	Other fusion of spine
M4327	Other fusion of spine
M4328	Other fusion of spine
M4329	Other fusion of spine
M433	Recurrent atlantoaxial subluxation with myelopathy
M4330	Recurrent atlantoaxial subluxation with myelopathy
M4331	Recurrent atlantoaxial subluxation with myelopathy
M4332	Recurrent atlantoaxial subluxation with myelopathy
M4333	Recurrent atlantoaxial subluxation with myelopathy
M4334	Recurrent atlantoaxial subluxation with myelopathy
M4335	Recurrent atlantoaxial subluxation with myelopathy
M4336	Recurrent atlantoaxial subluxation with myelopathy
M4337	Recurrent atlantoaxial subluxation with myelopathy
M4338	Recurrent atlantoaxial subluxation with myelopathy
M4339	Recurrent atlantoaxial subluxation with myelopathy
M434	Other recurrent atlantoaxial subluxation
M435	Other recurrent vertebral subluxation
M4350	Other recurrent vertebral subluxation
M4352	Other recurrent vertebral subluxation
M4353	Other recurrent vertebral subluxation
M4354	Other recurrent vertebral subluxation
M4355	Other recurrent vertebral subluxation
M4356	Other recurrent vertebral subluxation
M4357	Other recurrent vertebral subluxation
M4358	Other recurrent vertebral subluxation
M4359	Other recurrent vertebral subluxation
M436	Torticollis
M438	Other specified deforming dorsopathies
M4380	Other specified deforming dorsopathies
M4381	Other specified deforming dorsopathies
M4382	Other specified deforming dorsopathies
M4383	Other specified deforming dorsopathies
M4384	Other specified deforming dorsopathies
M4385	Other specified deforming dorsopathies
M4386	Other specified deforming dorsopathies
M4387	Other specified deforming dorsopathies
M4388	Other specified deforming dorsopathies
M4389	Other specified deforming dorsopathies
M439	Deforming dorsopathy, unspecified
M4390	Deforming dorsopathy, unspecified
M4391	Deforming dorsopathy, unspecified
M4392	Deforming dorsopathy, unspecified
M4393	Deforming dorsopathy, unspecified
M4394	Deforming dorsopathy, unspecified

For peer review only

M4395	Deforming dorsopathy, unspecified
M4396	Deforming dorsopathy, unspecified
M4397	Deforming dorsopathy, unspecified
M4398	Deforming dorsopathy, unspecified
M4399	Deforming dorsopathy, unspecified
M45X	Ankylosing spondylitis
M45X0	Ankylosing spondylitis
M45X1	Ankylosing spondylitis
M45X2	Ankylosing spondylitis
M45X3	Ankylosing spondylitis
M45X4	Ankylosing spondylitis
M45X5	Ankylosing spondylitis
M45X6	Ankylosing spondylitis
M45X7	Ankylosing spondylitis
M45X8	Ankylosing spondylitis
M45X9	Ankylosing spondylitis
M46	Other inflammatory spondylopathies
M460	Spinal enthesopathy
M4600	Spinal enthesopathy
M4601	Spinal enthesopathy
M4602	Spinal enthesopathy
M4603	Spinal enthesopathy
M4604	Spinal enthesopathy
M4605	Spinal enthesopathy
M4606	Spinal enthesopathy
M4607	Spinal enthesopathy
M4608	Spinal enthesopathy
M4609	Spinal enthesopathy
M461	Sacroiliitis, not elsewhere classified
M462	Osteomyelitis of vertebra
M4620	Osteomyelitis of vertebra
M4621	Osteomyelitis of vertebra
M4622	Osteomyelitis of vertebra
M4623	Osteomyelitis of vertebra
M4624	Osteomyelitis of vertebra
M4625	Osteomyelitis of vertebra
M4626	Osteomyelitis of vertebra
M4627	Osteomyelitis of vertebra
M4628	Osteomyelitis of vertebra
M4629	Osteomyelitis of vertebra
M463	Infection of intervertebral disc (pyogenic)
M4630	Infection of intervertebral disc (pyogenic)
M4631	Infection of intervertebral disc (pyogenic)
M4632	Infection of intervertebral disc (pyogenic)
M4633	Infection of intervertebral disc (pyogenic)
M4634	Infection of intervertebral disc (pyogenic)
M4635	Infection of intervertebral disc (pyogenic)
M4636	Infection of intervertebral disc (pyogenic)

For peer review only

M4637	Infection of intervertebral disc (pyogenic)
M4638	Infection of intervertebral disc (pyogenic)
M4639	Infection of intervertebral disc (pyogenic)
M464	Discitis, unspecified
M4640	Discitis, unspecified
M4641	Discitis, unspecified
M4642	Discitis, unspecified
M4643	Discitis, unspecified
M4644	Discitis, unspecified
M4645	Discitis, unspecified
M4646	Discitis, unspecified
M4647	Discitis, unspecified
M4648	Discitis, unspecified
M4649	Discitis, unspecified
M465	Other infective spondylopathies
M4650	Other infective spondylopathies
M4651	Other infective spondylopathies
M4652	Other infective spondylopathies
M4653	Other infective spondylopathies
M4654	Other infective spondylopathies
M4655	Other infective spondylopathies
M4656	Other infective spondylopathies
M4657	Other infective spondylopathies
M4658	Other infective spondylopathies
M4659	Other infective spondylopathies
M468	Other specified inflammatory spondylopathies
M4680	Other specified inflammatory spondylopathies
M4681	Other specified inflammatory spondylopathies
M4682	Other specified inflammatory spondylopathies
M4683	Other specified inflammatory spondylopathies
M4684	Other specified inflammatory spondylopathies
M4685	Other specified inflammatory spondylopathies
M4686	Other specified inflammatory spondylopathies
M4687	Other specified inflammatory spondylopathies
M4688	Other specified inflammatory spondylopathies
M4689	Other specified inflammatory spondylopathies
M469	Inflammatory spondylopathy, unspecified
M4690	Inflammatory spondylopathy, unspecified
M4691	Inflammatory spondylopathy, unspecified
M4692	Inflammatory spondylopathy, unspecified
M4693	Inflammatory spondylopathy, unspecified
M4694	Inflammatory spondylopathy, unspecified
M4695	Inflammatory spondylopathy, unspecified
M4696	Inflammatory spondylopathy, unspecified
M4697	Inflammatory spondylopathy, unspecified
M4698	Inflammatory spondylopathy, unspecified
M4699	Inflammatory spondylopathy, unspecified
M47	Spondylosis

For peer review only

M470	Anterior spinal and vertebral artery compression syndromes
M4700	Anterior spinal and vertebral artery compression syndromes
M4701	Anterior spinal and vertebral artery compression syndromes
M4702	Anterior spinal and vertebral artery compression syndromes
M4703	Anterior spinal and vertebral artery compression syndromes
M4704	Anterior spinal and vertebral artery compression syndromes
M4705	Anterior spinal and vertebral artery compression syndromes
M4706	Anterior spinal and vertebral artery compression syndromes
M4707	Anterior spinal and vertebral artery compression syndromes
M4708	Anterior spinal and vertebral artery compression syndromes
M4709	Anterior spinal and vertebral artery compression syndromes
M471	Other spondylosis with myelopathy
M4710	Other spondylosis with myelopathy
M4711	Other spondylosis with myelopathy
M4712	Other spondylosis with myelopathy
M4713	Other spondylosis with myelopathy
M4714	Other spondylosis with myelopathy
M4715	Other spondylosis with myelopathy
M4716	Other spondylosis with myelopathy
M4717	Other spondylosis with myelopathy
M4718	Other spondylosis with myelopathy
M4719	Other spondylosis with myelopathy
M472	Other spondylosis with radiculopathy
M4720	Other spondylosis with radiculopathy
M4721	Other spondylosis with radiculopathy
M4722	Other spondylosis with radiculopathy
M4723	Other spondylosis with radiculopathy
M4724	Other spondylosis with radiculopathy
M4725	Other spondylosis with radiculopathy
M4726	Other spondylosis with radiculopathy
M4727	Other spondylosis with radiculopathy
M4728	Other spondylosis with radiculopathy
M4729	Other spondylosis with radiculopathy
M478	Other spondylosis
M4780	Other spondylosis
M4781	Other spondylosis
M4782	Other spondylosis
M4783	Other spondylosis
M4784	Other spondylosis
M4785	Other spondylosis
M4786	Other spondylosis
M4787	Other spondylosis
M4788	Other spondylosis
M4789	Other spondylosis
M479	Spondylosis, unspecified
M4790	Spondylosis, unspecified
M4791	Spondylosis, unspecified
M4792	Spondylosis, unspecified

For peer review only

M4793	Spondylosis, unspecified
M4794	Spondylosis, unspecified
M4795	Spondylosis, unspecified
M4796	Spondylosis, unspecified
M4797	Spondylosis, unspecified
M4798	Spondylosis, unspecified
M4799	Spondylosis, unspecified
M48	Other spondylopathies
M480	Spinal stenosis
M4800	Spinal stenosis
M4801	Spinal stenosis
M4802	Spinal stenosis
M4803	Spinal stenosis
M4804	Spinal stenosis
M4805	Spinal stenosis
M4806	Spinal stenosis
M4807	Spinal stenosis
M4808	Spinal stenosis
M4809	Spinal stenosis
M481	Ankylosing hyperostosis [Forestier]
M4810	Ankylosing hyperostosis [Forestier]
M4811	Ankylosing hyperostosis [Forestier]
M4812	Ankylosing hyperostosis [Forestier]
M4813	Ankylosing hyperostosis [Forestier]
M4814	Ankylosing hyperostosis [Forestier]
M4815	Ankylosing hyperostosis [Forestier]
M4816	Ankylosing hyperostosis [Forestier]
M4817	Ankylosing hyperostosis [Forestier]
M4818	Ankylosing hyperostosis [Forestier]
M4819	Ankylosing hyperostosis [Forestier]
M482	Kissing spine
M4820	Kissing spine
M4821	Kissing spine
M4822	Kissing spine
M4823	Kissing spine
M4824	Kissing spine
M4825	Kissing spine
M4826	Kissing spine
M4827	Kissing spine
M4828	Kissing spine
M4829	Kissing spine
M483	Traumatic spondylopathy
M4830	Traumatic spondylopathy
M4831	Traumatic spondylopathy
M4832	Traumatic spondylopathy
M4833	Traumatic spondylopathy
M4834	Traumatic spondylopathy
M4835	Traumatic spondylopathy

For peer review only

M4836	Traumatic spondylopathy
M4837	Traumatic spondylopathy
M4838	Traumatic spondylopathy
M4839	Traumatic spondylopathy
M484	Fatigue fracture of vertebra
M4840	Fatigue fracture of vertebra
M4841	Fatigue fracture of vertebra
M4842	Fatigue fracture of vertebra
M4843	Fatigue fracture of vertebra
M4844	Fatigue fracture of vertebra
M4845	Fatigue fracture of vertebra
M4846	Fatigue fracture of vertebra
M4847	Fatigue fracture of vertebra
M4848	Fatigue fracture of vertebra
M4849	Fatigue fracture of vertebra
M485	Collapsed vertebra, not elsewhere classified
M4850	Collapsed vertebra, not elsewhere classified
M4851	Collapsed vertebra, not elsewhere classified
M4852	Collapsed vertebra, not elsewhere classified
M4853	Collapsed vertebra, not elsewhere classified
M4854	Collapsed vertebra, not elsewhere classified
M4855	Collapsed vertebra, not elsewhere classified
M4856	Collapsed vertebra, not elsewhere classified
M4857	Collapsed vertebra, not elsewhere classified
M4858	Collapsed vertebra, not elsewhere classified
M4859	Collapsed vertebra, not elsewhere classified
M488	Other specified spondylopathies
M4880	Other specified spondylopathies
M4881	Other specified spondylopathies
M4882	Other specified spondylopathies
M4883	Other specified spondylopathies
M4884	Other specified spondylopathies
M4885	Other specified spondylopathies
M4886	Other specified spondylopathies
M4887	Other specified spondylopathies
M4888	Other specified spondylopathies
M4889	Other specified spondylopathies
M489	Spondylopathy, unspecified
M4890	Spondylopathy, unspecified
M4891	Spondylopathy, unspecified
M4892	Spondylopathy, unspecified
M4893	Spondylopathy, unspecified
M4894	Spondylopathy, unspecified
M4895	Spondylopathy, unspecified
M4896	Spondylopathy, unspecified
M4897	Spondylopathy, unspecified
M4898	Spondylopathy, unspecified
M4899	Spondylopathy, unspecified

For peer review only

M49	Spondylopathies in diseases classified elsewhere
M490	Tuberculosis of spine
M4900	Tuberculosis of spine
M4901	Tuberculosis of spine
M4902	Tuberculosis of spine
M4903	Tuberculosis of spine
M4904	Tuberculosis of spine
M4905	Tuberculosis of spine
M4906	Tuberculosis of spine
M4907	Tuberculosis of spine
M4908	Tuberculosis of spine
M4909	Tuberculosis of spine
M491	Brucella spondylitis
M4910	Brucella spondylitis
M4911	Brucella spondylitis
M4912	Brucella spondylitis
M4913	Brucella spondylitis
M4914	Brucella spondylitis
M4915	Brucella spondylitis
M4916	Brucella spondylitis
M4917	Brucella spondylitis
M4918	Brucella spondylitis
M4919	Brucella spondylitis
M492	Enterobacterial spondylitis
M4920	Enterobacterial spondylitis
M4921	Enterobacterial spondylitis
M4922	Enterobacterial spondylitis
M4923	Enterobacterial spondylitis
M4924	Enterobacterial spondylitis
M4925	Enterobacterial spondylitis
M4926	Enterobacterial spondylitis
M4927	Enterobacterial spondylitis
M4928	Enterobacterial spondylitis
M4929	Enterobacterial spondylitis
M493	Spondylopathy in other infectious and parasitic diseases classified elsewhere
M4930	Spondylopathy in other infectious and parasitic diseases classified elsewhere
M4931	Spondylopathy in other infectious and parasitic diseases classified elsewhere
M4932	Spondylopathy in other infectious and parasitic diseases classified elsewhere
M4933	Spondylopathy in other infectious and parasitic diseases classified elsewhere
M4934	Spondylopathy in other infectious and parasitic diseases classified elsewhere
M4935	Spondylopathy in other infectious and parasitic diseases classified elsewhere
M4936	Spondylopathy in other infectious and parasitic diseases classified elsewhere
M4937	Spondylopathy in other infectious and parasitic diseases classified elsewhere
M4938	Spondylopathy in other infectious and parasitic diseases classified elsewhere
M4939	Spondylopathy in other infectious and parasitic diseases classified elsewhere
M494	Neuropathic spondylopathy
M4940	Neuropathic spondylopathy
M4941	Neuropathic spondylopathy

For peer review only

M4942	Neuropathic spondylopathy
M4943	Neuropathic spondylopathy
M4944	Neuropathic spondylopathy
M4945	Neuropathic spondylopathy
M4946	Neuropathic spondylopathy
M4947	Neuropathic spondylopathy
M4948	Neuropathic spondylopathy
M4949	Neuropathic spondylopathy
M495	Collapsed vertebra in diseases classified elsewhere
M4950	Collapsed vertebra in diseases classified elsewhere
M4951	Collapsed vertebra in diseases classified elsewhere
M4952	Collapsed vertebra in diseases classified elsewhere
M4953	Collapsed vertebra in diseases classified elsewhere
M4954	Collapsed vertebra in diseases classified elsewhere
M4955	Collapsed vertebra in diseases classified elsewhere
M4956	Collapsed vertebra in diseases classified elsewhere
M4957	Collapsed vertebra in diseases classified elsewhere
M4958	Collapsed vertebra in diseases classified elsewhere
M4959	Collapsed vertebra in diseases classified elsewhere
M498	Spondylopathy in other diseases classified elsewhere
M4980	Spondylopathy in other diseases classified elsewhere
M4981	Spondylopathy in other diseases classified elsewhere
M4982	Spondylopathy in other diseases classified elsewhere
M4983	Spondylopathy in other diseases classified elsewhere
M4984	Spondylopathy in other diseases classified elsewhere
M4985	Spondylopathy in other diseases classified elsewhere
M4986	Spondylopathy in other diseases classified elsewhere
M4987	Spondylopathy in other diseases classified elsewhere
M4988	Spondylopathy in other diseases classified elsewhere
M4989	Spondylopathy in other diseases classified elsewhere
M50	Cervical disc disorders
M500	Cervical disc disorder with myelopathy
M501	Cervical disc disorder with radiculopathy
M502	Other cervical disc displacement
M503	Other cervical disc degeneration
M508	Other cervical disc disorders
M509	Cervical disc disorder, unspecified
M51	Other intervertebral disc disorders
M510	Lumbar and other intervertebral disc disorders with myelopathy
M511	Lumbar and other intervertebral disc disorders with radiculopathy
M512	Other specified intervertebral disc displacement
M513	Other specified intervertebral disc degeneration
M514	Schmorl nodes
M514	Schmorl's nodes
M518	Other specified intervertebral disc disorders
M519	Intervertebral disc disorder, unspecified
M53	Other dorsopathies, not elsewhere classified
M530	Cervicocranial syndrome

M5300	Cervicocranial syndrome
M5301	Cervicocranial syndrome
M5302	Cervicocranial syndrome
M5303	Cervicocranial syndrome
M531	Cervicobrachial syndrome
M5310	Cervicobrachial syndrome
M5311	Cervicobrachial syndrome
M5312	Cervicobrachial syndrome
M5313	Cervicobrachial syndrome
M532	Spinal instabilities
M5320	Spinal instabilities
M5321	Spinal instabilities
M5322	Spinal instabilities
M5323	Spinal instabilities
M5324	Spinal instabilities
M5325	Spinal instabilities
M5326	Spinal instabilities
M5327	Spinal instabilities
M5328	Spinal instabilities
M5329	Spinal instabilities
M533	Sacrococcygeal disorders, not elsewhere classified
M538	Other specified dorsopathies
M5380	Other specified dorsopathies
M5381	Other specified dorsopathies
M5382	Other specified dorsopathies
M5383	Other specified dorsopathies
M5384	Other specified dorsopathies
M5385	Other specified dorsopathies
M5386	Other specified dorsopathies
M5387	Other specified dorsopathies
M5388	Other specified dorsopathies
M5389	Other specified dorsopathies
M539	Dorsopathy, unspecified
M5390	Dorsopathy, unspecified
M5391	Dorsopathy, unspecified
M5392	Dorsopathy, unspecified
M5393	Dorsopathy, unspecified
M5394	Dorsopathy, unspecified
M5395	Dorsopathy, unspecified
M5396	Dorsopathy, unspecified
M5397	Dorsopathy, unspecified
M5398	Dorsopathy, unspecified
M5399	Dorsopathy, unspecified
M54	Dorsalgia
M540	Panniculitis affecting regions of neck and back
M5400	Panniculitis affecting regions of neck and back
M5401	Panniculitis affecting regions of neck and back
M5402	Panniculitis affecting regions of neck and back

For peer review only

M5403	Panniculitis affecting regions of neck and back
M5404	Panniculitis affecting regions of neck and back
M5405	Panniculitis affecting regions of neck and back
M5406	Panniculitis affecting regions of neck and back
M5407	Panniculitis affecting regions of neck and back
M5408	Panniculitis affecting regions of neck and back
M5409	Panniculitis affecting regions of neck and back
M541	Radiculopathy
M5410	Radiculopathy
M5411	Radiculopathy
M5412	Radiculopathy
M5413	Radiculopathy
M5414	Radiculopathy
M5415	Radiculopathy
M5416	Radiculopathy
M5417	Radiculopathy
M5418	Radiculopathy
M5419	Radiculopathy
M542	Cervicalgia
M5421	Cervicalgia
M5422	Cervicalgia
M5423	Cervicalgia
M543	Sciatica
M5430	Sciatica
M5435	Sciatica
M5436	Sciatica
M5437	Sciatica
M5438	Sciatica
M5439	Sciatica
M544	Lumbago with sciatica
M5440	Lumbago with sciatica
M5445	Lumbago with sciatica
M5446	Lumbago with sciatica
M5447	Lumbago with sciatica
M5448	Lumbago with sciatica
M5449	Lumbago with sciatica
M545	Low back pain
M5450	Low back pain
M5455	Low back pain
M5456	Low back pain
M5457	Low back pain
M5458	Low back pain
M5459	Low back pain
M546	Pain in thoracic spine
M5460	Pain in thoracic spine
M5463	Pain in thoracic spine
M5464	Pain in thoracic spine
M5465	Pain in thoracic spine

For peer review only

M548	Other dorsalgia
M5480	Other dorsalgia
M5481	Other dorsalgia
M5482	Other dorsalgia
M5483	Other dorsalgia
M5484	Other dorsalgia
M5485	Other dorsalgia
M5486	Other dorsalgia
M5487	Other dorsalgia
M5488	Other dorsalgia
M5489	Other dorsalgia
M549	Dorsalgia, unspecified
M5490	Dorsalgia, unspecified
M5491	Dorsalgia, unspecified
M5492	Dorsalgia, unspecified
M5493	Dorsalgia, unspecified
M5494	Dorsalgia, unspecified
M5495	Dorsalgia, unspecified
M5496	Dorsalgia, unspecified
M5497	Dorsalgia, unspecified
M5498	Dorsalgia, unspecified
M5499	Dorsalgia, unspecified
M60	Myositis
M600	Infective myositis
M6000	Infective myositis
M6001	Infective myositis
M6002	Infective myositis
M6003	Infective myositis
M6004	Infective myositis
M6005	Infective myositis
M6006	Infective myositis
M6007	Infective myositis
M6008	Infective myositis
M6009	Infective myositis
M601	Interstitial myositis
M6010	Interstitial myositis
M6011	Interstitial myositis
M6012	Interstitial myositis
M6013	Interstitial myositis
M6014	Interstitial myositis
M6015	Interstitial myositis
M6016	Interstitial myositis
M6017	Interstitial myositis
M6018	Interstitial myositis
M6019	Interstitial myositis
M602	Foreign body granuloma of soft tissue, not elsewhere classified
M6020	Foreign body granuloma of soft tissue, not elsewhere classified
M6021	Foreign body granuloma of soft tissue, not elsewhere classified

For peer review only

M6022	Foreign body granuloma of soft tissue, not elsewhere classified
M6023	Foreign body granuloma of soft tissue, not elsewhere classified
M6024	Foreign body granuloma of soft tissue, not elsewhere classified
M6025	Foreign body granuloma of soft tissue, not elsewhere classified
M6026	Foreign body granuloma of soft tissue, not elsewhere classified
M6027	Foreign body granuloma of soft tissue, not elsewhere classified
M6028	Foreign body granuloma of soft tissue, not elsewhere classified
M6029	Foreign body granuloma of soft tissue, not elsewhere classified
M608	Other myositis
M6080	Other myositis
M6081	Other myositis
M6082	Other myositis
M6083	Other myositis
M6084	Other myositis
M6085	Other myositis
M6086	Other myositis
M6087	Other myositis
M6088	Other myositis
M6089	Other myositis
M609	Myositis, unspecified
M6090	Myositis, unspecified
M6091	Myositis, unspecified
M6092	Myositis, unspecified
M6093	Myositis, unspecified
M6094	Myositis, unspecified
M6095	Myositis, unspecified
M6096	Myositis, unspecified
M6097	Myositis, unspecified
M6098	Myositis, unspecified
M6099	Myositis, unspecified
M61	Calcification and ossification of muscle
M610	Myositis ossificans traumatica
M6100	Myositis ossificans traumatica
M6101	Myositis ossificans traumatica
M6102	Myositis ossificans traumatica
M6103	Myositis ossificans traumatica
M6104	Myositis ossificans traumatica
M6105	Myositis ossificans traumatica
M6106	Myositis ossificans traumatica
M6107	Myositis ossificans traumatica
M6108	Myositis ossificans traumatica
M6109	Myositis ossificans traumatica
M611	Myositis ossificans progressiva
M6110	Myositis ossificans progressiva
M6111	Myositis ossificans progressiva
M6112	Myositis ossificans progressiva
M6113	Myositis ossificans progressiva
M6114	Myositis ossificans progressiva

For peer review only

M6115	Myositis ossificans progressiva
M6116	Myositis ossificans progressiva
M6117	Myositis ossificans progressiva
M6118	Myositis ossificans progressiva
M6119	Myositis ossificans progressiva
M612	Paralytic calcification and ossification of muscle
M6120	Paralytic calcification and ossification of muscle
M6121	Paralytic calcification and ossification of muscle
M6122	Paralytic calcification and ossification of muscle
M6123	Paralytic calcification and ossification of muscle
M6124	Paralytic calcification and ossification of muscle
M6125	Paralytic calcification and ossification of muscle
M6126	Paralytic calcification and ossification of muscle
M6127	Paralytic calcification and ossification of muscle
M6128	Paralytic calcification and ossification of muscle
M6129	Paralytic calcification and ossification of muscle
M613	Calcification and ossification of muscles associated with burns
M6130	Calcification and ossification of muscles associated with burns
M6131	Calcification and ossification of muscles associated with burns
M6132	Calcification and ossification of muscles associated with burns
M6133	Calcification and ossification of muscles associated with burns
M6134	Calcification and ossification of muscles associated with burns
M6135	Calcification and ossification of muscles associated with burns
M6136	Calcification and ossification of muscles associated with burns
M6137	Calcification and ossification of muscles associated with burns
M6138	Calcification and ossification of muscles associated with burns
M6139	Calcification and ossification of muscles associated with burns
M614	Other calcification of muscle
M6140	Other calcification of muscle
M6141	Other calcification of muscle
M6142	Other calcification of muscle
M6143	Other calcification of muscle
M6144	Other calcification of muscle
M6145	Other calcification of muscle
M6146	Other calcification of muscle
M6147	Other calcification of muscle
M6148	Other calcification of muscle
M6149	Other calcification of muscle
M615	Other ossification of muscle
M6150	Other ossification of muscle
M6151	Other ossification of muscle
M6152	Other ossification of muscle
M6153	Other ossification of muscle
M6154	Other ossification of muscle
M6155	Other ossification of muscle
M6156	Other ossification of muscle
M6157	Other ossification of muscle
M6158	Other ossification of muscle

For peer review only

M6159	Other ossification of muscle
M619	Calcification and ossification of muscle, unspecified
M6190	Calcification and ossification of muscle, unspecified
M6191	Calcification and ossification of muscle, unspecified
M6192	Calcification and ossification of muscle, unspecified
M6193	Calcification and ossification of muscle, unspecified
M6194	Calcification and ossification of muscle, unspecified
M6195	Calcification and ossification of muscle, unspecified
M6196	Calcification and ossification of muscle, unspecified
M6197	Calcification and ossification of muscle, unspecified
M6198	Calcification and ossification of muscle, unspecified
M6199	Calcification and ossification of muscle, unspecified
M62	Other disorders of muscle
M620	Diastasis of muscle
M6200	Diastasis of muscle
M6201	Diastasis of muscle
M6202	Diastasis of muscle
M6203	Diastasis of muscle
M6204	Diastasis of muscle
M6205	Diastasis of muscle
M6206	Diastasis of muscle
M6207	Diastasis of muscle
M6208	Diastasis of muscle
M6209	Diastasis of muscle
M621	Other rupture of muscle (nontraumatic)
M6210	Other rupture of muscle (nontraumatic)
M6211	Other rupture of muscle (nontraumatic)
M6212	Other rupture of muscle (nontraumatic)
M6213	Other rupture of muscle (nontraumatic)
M6214	Other rupture of muscle (nontraumatic)
M6215	Other rupture of muscle (nontraumatic)
M6216	Other rupture of muscle (nontraumatic)
M6217	Other rupture of muscle (nontraumatic)
M6218	Other rupture of muscle (nontraumatic)
M6219	Other rupture of muscle (nontraumatic)
M622	Ischaemic infarction of muscle
M6220	Ischaemic infarction of muscle
M6221	Ischaemic infarction of muscle
M6222	Ischaemic infarction of muscle
M6223	Ischaemic infarction of muscle
M6224	Ischaemic infarction of muscle
M6225	Ischaemic infarction of muscle
M6226	Ischaemic infarction of muscle
M6227	Ischaemic infarction of muscle
M6228	Ischaemic infarction of muscle
M6229	Ischaemic infarction of muscle
M623	Immobility syndrome (paraplegic)
M6230	Immobility syndrome (paraplegic)

For peer review only

M6231	Immobility syndrome (paraplegic)
M6232	Immobility syndrome (paraplegic)
M6233	Immobility syndrome (paraplegic)
M6234	Immobility syndrome (paraplegic)
M6235	Immobility syndrome (paraplegic)
M6236	Immobility syndrome (paraplegic)
M6237	Immobility syndrome (paraplegic)
M6238	Immobility syndrome (paraplegic)
M6239	Immobility syndrome (paraplegic)
M624	Contracture of muscle
M6240	Contracture of muscle
M6241	Contracture of muscle
M6242	Contracture of muscle
M6243	Contracture of muscle
M6244	Contracture of muscle
M6245	Contracture of muscle
M6246	Contracture of muscle
M6247	Contracture of muscle
M6248	Contracture of muscle
M6249	Contracture of muscle
M625	Muscle wasting and atrophy, not elsewhere classified
M6250	Muscle wasting and atrophy, not elsewhere classified
M6251	Muscle wasting and atrophy, not elsewhere classified
M6252	Muscle wasting and atrophy, not elsewhere classified
M6253	Muscle wasting and atrophy, not elsewhere classified
M6254	Muscle wasting and atrophy, not elsewhere classified
M6255	Muscle wasting and atrophy, not elsewhere classified
M6256	Muscle wasting and atrophy, not elsewhere classified
M6257	Muscle wasting and atrophy, not elsewhere classified
M6258	Muscle wasting and atrophy, not elsewhere classified
M6259	Muscle wasting and atrophy, not elsewhere classified
M626	Muscle strain
M6260	Muscle strain
M6261	Muscle strain
M6262	Muscle strain
M6263	Muscle strain
M6264	Muscle strain
M6265	Muscle strain
M6266	Muscle strain
M6267	Muscle strain
M6268	Muscle strain
M6269	Muscle strain
M628	Other specified disorders of muscle
M6280	Other specified disorders of muscle
M6281	Other specified disorders of muscle
M6282	Other specified disorders of muscle
M6283	Other specified disorders of muscle
M6284	Other specified disorders of muscle

For peer review only

M6285	Other specified disorders of muscle
M6286	Other specified disorders of muscle
M6287	Other specified disorders of muscle
M6288	Other specified disorders of muscle
M6289	Other specified disorders of muscle
M629	Disorder of muscle, unspecified
M6290	Disorder of muscle, unspecified
M6291	Disorder of muscle, unspecified
M6292	Disorder of muscle, unspecified
M6293	Disorder of muscle, unspecified
M6294	Disorder of muscle, unspecified
M6295	Disorder of muscle, unspecified
M6296	Disorder of muscle, unspecified
M6297	Disorder of muscle, unspecified
M6298	Disorder of muscle, unspecified
M6299	Disorder of muscle, unspecified
M63	Disorders of muscle in diseases classified elsewhere
M630	Myositis in bacterial diseases classified elsewhere
M631	Myositis in protozoal and parasitic infections classified elsewhere
M632	Myositis in other infectious diseases classified elsewhere
M633	Myositis in sarcoidosis
M638	Other disorders of muscle in diseases classified elsewhere
M65	Synovitis and tenosynovitis
M650	Abscess of tendon sheath
M6500	Abscess of tendon sheath
M6501	Abscess of tendon sheath
M6502	Abscess of tendon sheath
M6503	Abscess of tendon sheath
M6504	Abscess of tendon sheath
M6505	Abscess of tendon sheath
M6506	Abscess of tendon sheath
M6507	Abscess of tendon sheath
M6508	Abscess of tendon sheath
M6509	Abscess of tendon sheath
M651	Other infective (teno)synovitis
M6510	Other infective (teno)synovitis
M6511	Other infective (teno)synovitis
M6512	Other infective (teno)synovitis
M6513	Other infective (teno)synovitis
M6514	Other infective (teno)synovitis
M6515	Other infective (teno)synovitis
M6516	Other infective (teno)synovitis
M6517	Other infective (teno)synovitis
M6518	Other infective (teno)synovitis
M6519	Other infective (teno)synovitis
M652	Calcific tendinitis
M6520	Calcific tendinitis
M6521	Calcific tendinitis

For peer review only

M6522	Calcific tendinitis
M6523	Calcific tendinitis
M6524	Calcific tendinitis
M6525	Calcific tendinitis
M6526	Calcific tendinitis
M6527	Calcific tendinitis
M6528	Calcific tendinitis
M6529	Calcific tendinitis
M653	Trigger finger
M654	Radial styloid tenosynovitis [de Quervain]
M6540	Radial styloid tenosynovitis [de Quervain]
M6543	Radial styloid tenosynovitis [de Quervain]
M6544	Radial styloid tenosynovitis [de Quervain]
M658	Other synovitis and tenosynovitis
M6580	Other synovitis and tenosynovitis
M6581	Other synovitis and tenosynovitis
M6582	Other synovitis and tenosynovitis
M6583	Other synovitis and tenosynovitis
M6584	Other synovitis and tenosynovitis
M6585	Other synovitis and tenosynovitis
M6586	Other synovitis and tenosynovitis
M6587	Other synovitis and tenosynovitis
M6588	Other synovitis and tenosynovitis
M6589	Other synovitis and tenosynovitis
M659	Synovitis and tenosynovitis, unspecified
M6590	Synovitis and tenosynovitis, unspecified
M6591	Synovitis and tenosynovitis, unspecified
M6592	Synovitis and tenosynovitis, unspecified
M6593	Synovitis and tenosynovitis, unspecified
M6594	Synovitis and tenosynovitis, unspecified
M6595	Synovitis and tenosynovitis, unspecified
M6596	Synovitis and tenosynovitis, unspecified
M6597	Synovitis and tenosynovitis, unspecified
M6598	Synovitis and tenosynovitis, unspecified
M6599	Synovitis and tenosynovitis, unspecified
M66	Spontaneous rupture of synovium and tendon
M660	Rupture of popliteal cyst
M661	Rupture of synovium
M6610	Rupture of synovium
M6611	Rupture of synovium
M6612	Rupture of synovium
M6613	Rupture of synovium
M6614	Rupture of synovium
M6615	Rupture of synovium
M6616	Rupture of synovium
M6617	Rupture of synovium
M6618	Rupture of synovium
M6619	Rupture of synovium

For peer review only

M662	Spontaneous rupture of extensor tendons
M6620	Spontaneous rupture of extensor tendons
M6621	Spontaneous rupture of extensor tendons
M6622	Spontaneous rupture of extensor tendons
M6623	Spontaneous rupture of extensor tendons
M6624	Spontaneous rupture of extensor tendons
M6625	Spontaneous rupture of extensor tendons
M6626	Spontaneous rupture of extensor tendons
M6627	Spontaneous rupture of extensor tendons
M6629	Spontaneous rupture of extensor tendons
M663	Spontaneous rupture of flexor tendons
M6630	Spontaneous rupture of flexor tendons
M6631	Spontaneous rupture of flexor tendons
M6632	Spontaneous rupture of flexor tendons
M6633	Spontaneous rupture of flexor tendons
M6634	Spontaneous rupture of flexor tendons
M6635	Spontaneous rupture of flexor tendons
M6636	Spontaneous rupture of flexor tendons
M6637	Spontaneous rupture of flexor tendons
M6638	Spontaneous rupture of flexor tendons
M6639	Spontaneous rupture of flexor tendons
M664	Spontaneous rupture of other tendons
M6640	Spontaneous rupture of other tendons
M6641	Spontaneous rupture of other tendons
M6642	Spontaneous rupture of other tendons
M6643	Spontaneous rupture of other tendons
M6644	Spontaneous rupture of other tendons
M6645	Spontaneous rupture of other tendons
M6646	Spontaneous rupture of other tendons
M6647	Spontaneous rupture of other tendons
M6648	Spontaneous rupture of other tendons
M6649	Spontaneous rupture of other tendons
M665	Spontaneous rupture of unspecified tendon
M6650	Spontaneous rupture of unspecified tendon
M6651	Spontaneous rupture of unspecified tendon
M6652	Spontaneous rupture of unspecified tendon
M6653	Spontaneous rupture of unspecified tendon
M6654	Spontaneous rupture of unspecified tendon
M6655	Spontaneous rupture of unspecified tendon
M6656	Spontaneous rupture of unspecified tendon
M6657	Spontaneous rupture of unspecified tendon
M6658	Spontaneous rupture of unspecified tendon
M6659	Spontaneous rupture of unspecified tendon
M67	Other disorders of synovium and tendon
M670	Short Achilles tendon (acquired)
M671	Other contracture of tendon (sheath)
M672	Synovial hypertrophy, not elsewhere classified
M673	Transient synovitis

For peer review only

M674	Ganglion
M678	Other specified disorders of synovium and tendon
M679	Disorder of synovium and tendon, unspecified
M68	Disorders of synovium and tendon in diseases classified elsewhere
M680	Synovitis and tenosynovitis in bacterial diseases classified elsewhere
M688	Other disorders of synovium and tendon in diseases classified elsewhere
M70	Soft tissue disorders related to use, overuse and pressure
M700	Chronic crepitant synovitis of hand and wrist
M7000	Chronic crepitant synovitis of hand and wrist
M7003	Chronic crepitant synovitis of hand and wrist
M7004	Chronic crepitant synovitis of hand and wrist
M701	Bursitis of hand
M702	Olecranon bursitis
M703	Other bursitis of elbow
M704	Prepatellar bursitis
M705	Other bursitis of knee
M706	Trochanteric bursitis
M707	Other bursitis of hip
M708	Other soft tissue disorders related to use, overuse and pressure
M7080	Other soft tissue disorders related to use, overuse and pressure
M7081	Other soft tissue disorders related to use, overuse and pressure
M7082	Other soft tissue disorders related to use, overuse and pressure
M7083	Other soft tissue disorders related to use, overuse and pressure
M7084	Other soft tissue disorders related to use, overuse and pressure
M7085	Other soft tissue disorders related to use, overuse and pressure
M7086	Other soft tissue disorders related to use, overuse and pressure
M7087	Other soft tissue disorders related to use, overuse and pressure
M7088	Other soft tissue disorders related to use, overuse and pressure
M7089	Other soft tissue disorders related to use, overuse and pressure
M709	Unspecified soft tissue disorder related to use, overuse and pressure
M7090	Unspecified soft tissue disorder related to use, overuse and pressure
M7091	Unspecified soft tissue disorder related to use, overuse and pressure
M7092	Unspecified soft tissue disorder related to use, overuse and pressure
M7093	Unspecified soft tissue disorder related to use, overuse and pressure
M7094	Unspecified soft tissue disorder related to use, overuse and pressure
M7095	Unspecified soft tissue disorder related to use, overuse and pressure
M7096	Unspecified soft tissue disorder related to use, overuse and pressure
M7097	Unspecified soft tissue disorder related to use, overuse and pressure
M7098	Unspecified soft tissue disorder related to use, overuse and pressure
M7099	Unspecified soft tissue disorder related to use, overuse and pressure
M71	Other bursopathies
M710	Abscess of bursa
M7100	Abscess of bursa
M7101	Abscess of bursa
M7102	Abscess of bursa
M7103	Abscess of bursa
M7104	Abscess of bursa
M7105	Abscess of bursa

For peer review only

M7106	Abscess of bursa
M7107	Abscess of bursa
M7108	Abscess of bursa
M7109	Abscess of bursa
M711	Other infective bursitis
M7110	Other infective bursitis
M7111	Other infective bursitis
M7112	Other infective bursitis
M7113	Other infective bursitis
M7114	Other infective bursitis
M7115	Other infective bursitis
M7116	Other infective bursitis
M7117	Other infective bursitis
M7118	Other infective bursitis
M7119	Other infective bursitis
M712	Synovial cyst of popliteal space [Baker]
M713	Other bursal cyst
M7130	Other bursal cyst
M7131	Other bursal cyst
M7132	Other bursal cyst
M7133	Other bursal cyst
M7134	Other bursal cyst
M7135	Other bursal cyst
M7136	Other bursal cyst
M7137	Other bursal cyst
M7138	Other bursal cyst
M7139	Other bursal cyst
M714	Calcium deposit in bursa
M7140	Calcium deposit in bursa
M7141	Calcium deposit in bursa
M7142	Calcium deposit in bursa
M7143	Calcium deposit in bursa
M7144	Calcium deposit in bursa
M7145	Calcium deposit in bursa
M7146	Calcium deposit in bursa
M7147	Calcium deposit in bursa
M7148	Calcium deposit in bursa
M7149	Calcium deposit in bursa
M715	Other bursitis, not elsewhere classified
M7150	Other bursitis, not elsewhere classified
M7151	Other bursitis, not elsewhere classified
M7152	Other bursitis, not elsewhere classified
M7153	Other bursitis, not elsewhere classified
M7154	Other bursitis, not elsewhere classified
M7155	Other bursitis, not elsewhere classified
M7156	Other bursitis, not elsewhere classified
M7157	Other bursitis, not elsewhere classified
M7158	Other bursitis, not elsewhere classified

For peer review only

M7159	Other bursitis, not elsewhere classified
M718	Other specified bursopathies
M7180	Other specified bursopathies
M7181	Other specified bursopathies
M7182	Other specified bursopathies
M7183	Other specified bursopathies
M7184	Other specified bursopathies
M7185	Other specified bursopathies
M7186	Other specified bursopathies
M7187	Other specified bursopathies
M7188	Other specified bursopathies
M7189	Other specified bursopathies
M719	Bursopathy, unspecified
M7190	Bursopathy, unspecified
M7191	Bursopathy, unspecified
M7192	Bursopathy, unspecified
M7193	Bursopathy, unspecified
M7194	Bursopathy, unspecified
M7195	Bursopathy, unspecified
M7196	Bursopathy, unspecified
M7197	Bursopathy, unspecified
M7198	Bursopathy, unspecified
M7199	Bursopathy, unspecified
M72	Fibroblastic disorders
M720	Palmar fascial fibromatosis [Dupuytren]
M721	Knuckle pads
M7210	Knuckle pads
M7214	Knuckle pads
M7217	Knuckle pads
M7219	Knuckle pads
M722	Plantar fascial fibromatosis
M723	Nodular fasciitis
M7230	Nodular fasciitis
M7231	Nodular fasciitis
M7232	Nodular fasciitis
M7233	Nodular fasciitis
M7234	Nodular fasciitis
M7235	Nodular fasciitis
M7236	Nodular fasciitis
M7237	Nodular fasciitis
M7238	Nodular fasciitis
M724	Pseudosarcomatous fibromatosis
M7240	Pseudosarcomatous fibromatosis
M7241	Pseudosarcomatous fibromatosis
M7242	Pseudosarcomatous fibromatosis
M7243	Pseudosarcomatous fibromatosis
M7244	Pseudosarcomatous fibromatosis
M7245	Pseudosarcomatous fibromatosis

For peer review only

M7246	Pseudosarcomatous fibromatosis
M7247	Pseudosarcomatous fibromatosis
M7248	Pseudosarcomatous fibromatosis
M7249	Pseudosarcomatous fibromatosis
M725	Fasciitis, not elsewhere classified
M7250	Fasciitis, not elsewhere classified
M7251	Fasciitis, not elsewhere classified
M7252	Fasciitis, not elsewhere classified
M7253	Fasciitis, not elsewhere classified
M7254	Fasciitis, not elsewhere classified
M7255	Fasciitis, not elsewhere classified
M7256	Fasciitis, not elsewhere classified
M7257	Fasciitis, not elsewhere classified
M7258	Fasciitis, not elsewhere classified
M7259	Fasciitis, not elsewhere classified
M726	Necrotizing fasciitis
M7260	Necrotizing fasciitis
M7261	Necrotizing fasciitis
M7262	Necrotizing fasciitis
M7263	Necrotizing fasciitis
M7264	Necrotizing fasciitis
M7265	Necrotizing fasciitis
M7266	Necrotizing fasciitis
M7267	Necrotizing fasciitis
M7268	Necrotizing fasciitis
M7269	Necrotizing fasciitis
M728	Other fibroblastic disorders
M7280	Other fibroblastic disorders
M7281	Other fibroblastic disorders
M7282	Other fibroblastic disorders
M7283	Other fibroblastic disorders
M7284	Other fibroblastic disorders
M7285	Other fibroblastic disorders
M7286	Other fibroblastic disorders
M7287	Other fibroblastic disorders
M7288	Other fibroblastic disorders
M7289	Other fibroblastic disorders
M729	Fibroblastic disorder, unspecified
M7290	Fibroblastic disorder, unspecified
M7291	Fibroblastic disorder, unspecified
M7292	Fibroblastic disorder, unspecified
M7293	Fibroblastic disorder, unspecified
M7294	Fibroblastic disorder, unspecified
M7295	Fibroblastic disorder, unspecified
M7296	Fibroblastic disorder, unspecified
M7297	Fibroblastic disorder, unspecified
M7298	Fibroblastic disorder, unspecified
M7299	Fibroblastic disorder, unspecified

For peer review only

M73	Soft tissue disorders in diseases classified elsewhere
M730	Gonococcal bursitis
M7300	Gonococcal bursitis
M7301	Gonococcal bursitis
M7302	Gonococcal bursitis
M7303	Gonococcal bursitis
M7304	Gonococcal bursitis
M7305	Gonococcal bursitis
M7306	Gonococcal bursitis
M7307	Gonococcal bursitis
M7308	Gonococcal bursitis
M7309	Gonococcal bursitis
M731	Syphilitic bursitis
M7310	Syphilitic bursitis
M7311	Syphilitic bursitis
M7312	Syphilitic bursitis
M7313	Syphilitic bursitis
M7314	Syphilitic bursitis
M7315	Syphilitic bursitis
M7316	Syphilitic bursitis
M7317	Syphilitic bursitis
M7318	Syphilitic bursitis
M7319	Syphilitic bursitis
M738	Other soft tissue disorders in diseases classified elsewhere
M7380	Other soft tissue disorders in diseases classified elsewhere
M7381	Other soft tissue disorders in diseases classified elsewhere
M7382	Other soft tissue disorders in diseases classified elsewhere
M7383	Other soft tissue disorders in diseases classified elsewhere
M7384	Other soft tissue disorders in diseases classified elsewhere
M7385	Other soft tissue disorders in diseases classified elsewhere
M7386	Other soft tissue disorders in diseases classified elsewhere
M7387	Other soft tissue disorders in diseases classified elsewhere
M7388	Other soft tissue disorders in diseases classified elsewhere
M7389	Other soft tissue disorders in diseases classified elsewhere
M75	Shoulder lesions
M750	Adhesive capsulitis of shoulder
M751	Rotator cuff syndrome
M752	Bicipital tendinitis
M753	Calcific tendinitis of shoulder
M754	Impingement syndrome of shoulder
M755	Bursitis of shoulder
M758	Other shoulder lesions
M759	Shoulder lesion, unspecified
M76	Enthesopathies of lower limb, excluding foot
M760	Gluteal tendinitis
M7600	Gluteal tendinitis
M7605	Gluteal tendinitis
M761	Psoas tendinitis

For peer review only

M7610	Psoas tendinitis
M7615	Psoas tendinitis
M762	Iliac crest spur
M763	Iliotibial band syndrome
M7630	Iliotibial band syndrome
M7635	Iliotibial band syndrome
M7636	Iliotibial band syndrome
M764	Tibial collateral bursitis [Pellegrini-Stieda]
M765	Patellar tendinitis
M766	Achilles tendinitis
M7660	Achilles tendinitis
M7666	Achilles tendinitis
M7667	Achilles tendinitis
M767	Peroneal tendinitis
M7670	Peroneal tendinitis
M7676	Peroneal tendinitis
M7677	Peroneal tendinitis
M768	Other enthesopathies of lower limb, excluding foot
M7680	Other enthesopathies of lower limb, excluding foot
M7685	Other enthesopathies of lower limb, excluding foot
M7686	Other enthesopathies of lower limb, excluding foot
M7687	Other enthesopathies of lower limb, excluding foot
M769	Enthesopathy of lower limb, unspecified
M7690	Enthesopathy of lower limb, unspecified
M7695	Enthesopathy of lower limb, unspecified
M7696	Enthesopathy of lower limb, unspecified
M7697	Enthesopathy of lower limb, unspecified
M7698	Enthesopathy of lower limb, unspecified
M7699	Enthesopathy of lower limb, unspecified
M77	Other enthesopathies
M770	Medial epicondylitis
M7700	Medial epicondylitis
M7702	Medial epicondylitis
M7705	Medial epicondylitis
M7706	Medial epicondylitis
M7709	Medial epicondylitis
M771	Lateral epicondylitis
M7710	Lateral epicondylitis
M7712	Lateral epicondylitis
M7715	Lateral epicondylitis
M7716	Lateral epicondylitis
M7719	Lateral epicondylitis
M772	Periarthritis of wrist
M773	Calcaneal spur
M774	Metatarsalgia
M775	Other enthesopathy of foot
M778	Other enthesopathies, not elsewhere classified
M7780	Other enthesopathies, not elsewhere classified

For peer review only

M7781	Other enthesopathies, not elsewhere classified
M7782	Other enthesopathies, not elsewhere classified
M7783	Other enthesopathies, not elsewhere classified
M7784	Other enthesopathies, not elsewhere classified
M7785	Other enthesopathies, not elsewhere classified
M7786	Other enthesopathies, not elsewhere classified
M7787	Other enthesopathies, not elsewhere classified
M7788	Other enthesopathies, not elsewhere classified
M7789	Other enthesopathies, not elsewhere classified
M779	Enthesopathy, unspecified
M7790	Enthesopathy, unspecified
M7791	Enthesopathy, unspecified
M7792	Enthesopathy, unspecified
M7793	Enthesopathy, unspecified
M7794	Enthesopathy, unspecified
M7795	Enthesopathy, unspecified
M7796	Enthesopathy, unspecified
M7797	Enthesopathy, unspecified
M7798	Enthesopathy, unspecified
M7799	Enthesopathy, unspecified
M79	Other soft tissue disorders, not elsewhere classified
M790	Rheumatism, unspecified
M7900	Rheumatism, unspecified
M7901	Rheumatism, unspecified
M7902	Rheumatism, unspecified
M7903	Rheumatism, unspecified
M7904	Rheumatism, unspecified
M7905	Rheumatism, unspecified
M7906	Rheumatism, unspecified
M7907	Rheumatism, unspecified
M7908	Rheumatism, unspecified
M7909	Rheumatism, unspecified
M791	Myalgia
M7910	Myalgia
M7911	Myalgia
M7912	Myalgia
M7913	Myalgia
M7914	Myalgia
M7915	Myalgia
M7916	Myalgia
M7917	Myalgia
M7918	Myalgia
M7919	Myalgia
M792	Neuralgia and neuritis, unspecified
M7920	Neuralgia and neuritis, unspecified
M7921	Neuralgia and neuritis, unspecified
M7922	Neuralgia and neuritis, unspecified
M7923	Neuralgia and neuritis, unspecified

For peer review only

M7924	Neuralgia and neuritis, unspecified
M7925	Neuralgia and neuritis, unspecified
M7926	Neuralgia and neuritis, unspecified
M7927	Neuralgia and neuritis, unspecified
M7928	Neuralgia and neuritis, unspecified
M7929	Neuralgia and neuritis, unspecified
M793	Panniculitis, unspecified
M7930	Panniculitis, unspecified
M7931	Panniculitis, unspecified
M7932	Panniculitis, unspecified
M7933	Panniculitis, unspecified
M7934	Panniculitis, unspecified
M7935	Panniculitis, unspecified
M7936	Panniculitis, unspecified
M7937	Panniculitis, unspecified
M7938	Panniculitis, unspecified
M7939	Panniculitis, unspecified
M794	Hypertrophy of (infrapatellar) fat pad
M7940	Hypertrophy of (infrapatellar) fat pad
M7941	Hypertrophy of (infrapatellar) fat pad
M7942	Hypertrophy of (infrapatellar) fat pad
M7943	Hypertrophy of (infrapatellar) fat pad
M7944	Hypertrophy of (infrapatellar) fat pad
M7945	Hypertrophy of (infrapatellar) fat pad
M7946	Hypertrophy of (infrapatellar) fat pad
M7947	Hypertrophy of (infrapatellar) fat pad
M7948	Hypertrophy of (infrapatellar) fat pad
M795	Residual foreign body in soft tissue
M7950	Residual foreign body in soft tissue
M7951	Residual foreign body in soft tissue
M7952	Residual foreign body in soft tissue
M7953	Residual foreign body in soft tissue
M7954	Residual foreign body in soft tissue
M7955	Residual foreign body in soft tissue
M7956	Residual foreign body in soft tissue
M7957	Residual foreign body in soft tissue
M7958	Residual foreign body in soft tissue
M7959	Residual foreign body in soft tissue
M796	Pain in limb
M7960	Pain in limb
M7961	Pain in limb
M7962	Pain in limb
M7963	Pain in limb
M7964	Pain in limb
M7965	Pain in limb
M7966	Pain in limb
M7967	Pain in limb
M7968	Pain in limb

For peer review only

M7969	Pain in limb
M797	Fibromyalgia
M7970	Fibromyalgia
M7971	Fibromyalgia
M7972	Fibromyalgia
M7973	Fibromyalgia
M7974	Fibromyalgia
M7975	Fibromyalgia
M7976	Fibromyalgia
M7977	Fibromyalgia
M7978	Fibromyalgia
M7979	Fibromyalgia
M798	Other specified soft tissue disorders
M7980	Other specified soft tissue disorders
M7981	Other specified soft tissue disorders
M7982	Other specified soft tissue disorders
M7983	Other specified soft tissue disorders
M7984	Other specified soft tissue disorders
M7985	Other specified soft tissue disorders
M7986	Other specified soft tissue disorders
M7987	Other specified soft tissue disorders
M7988	Other specified soft tissue disorders
M7989	Other specified soft tissue disorders
M799	Soft tissue disorder, unspecified
M7990	Soft tissue disorder, unspecified
M7991	Soft tissue disorder, unspecified
M7992	Soft tissue disorder, unspecified
M7993	Soft tissue disorder, unspecified
M7994	Soft tissue disorder, unspecified
M7995	Soft tissue disorder, unspecified
M7996	Soft tissue disorder, unspecified
M7997	Soft tissue disorder, unspecified
M7998	Soft tissue disorder, unspecified
M7999	Soft tissue disorder, unspecified
M80	Osteoporosis with pathological fracture
M800	Postmenopausal osteoporosis with pathological fracture
M8000	Postmenopausal osteoporosis with pathological fracture
M8001	Postmenopausal osteoporosis with pathological fracture
M8002	Postmenopausal osteoporosis with pathological fracture
M8003	Postmenopausal osteoporosis with pathological fracture
M8004	Postmenopausal osteoporosis with pathological fracture
M8005	Postmenopausal osteoporosis with pathological fracture
M8006	Postmenopausal osteoporosis with pathological fracture
M8007	Postmenopausal osteoporosis with pathological fracture
M8008	Postmenopausal osteoporosis with pathological fracture
M8009	Postmenopausal osteoporosis with pathological fracture
M801	Postoophorectomy osteoporosis with pathological fracture
M8010	Postoophorectomy osteoporosis with pathological fracture

For peer review only

M8011	Postoophorectomy osteoporosis with pathological fracture
M8012	Postoophorectomy osteoporosis with pathological fracture
M8013	Postoophorectomy osteoporosis with pathological fracture
M8014	Postoophorectomy osteoporosis with pathological fracture
M8015	Postoophorectomy osteoporosis with pathological fracture
M8016	Postoophorectomy osteoporosis with pathological fracture
M8017	Postoophorectomy osteoporosis with pathological fracture
M8018	Postoophorectomy osteoporosis with pathological fracture
M8019	Postoophorectomy osteoporosis with pathological fracture
M802	Osteoporosis of disuse with pathological fracture
M8020	Osteoporosis of disuse with pathological fracture
M8021	Osteoporosis of disuse with pathological fracture
M8022	Osteoporosis of disuse with pathological fracture
M8023	Osteoporosis of disuse with pathological fracture
M8024	Osteoporosis of disuse with pathological fracture
M8025	Osteoporosis of disuse with pathological fracture
M8026	Osteoporosis of disuse with pathological fracture
M8027	Osteoporosis of disuse with pathological fracture
M8028	Osteoporosis of disuse with pathological fracture
M8029	Osteoporosis of disuse with pathological fracture
M803	Postsurgical malabsorption osteoporosis with pathological fracture
M8030	Postsurgical malabsorption osteoporosis with pathological fracture
M8031	Postsurgical malabsorption osteoporosis with pathological fracture
M8032	Postsurgical malabsorption osteoporosis with pathological fracture
M8033	Postsurgical malabsorption osteoporosis with pathological fracture
M8034	Postsurgical malabsorption osteoporosis with pathological fracture
M8035	Postsurgical malabsorption osteoporosis with pathological fracture
M8036	Postsurgical malabsorption osteoporosis with pathological fracture
M8037	Postsurgical malabsorption osteoporosis with pathological fracture
M8038	Postsurgical malabsorption osteoporosis with pathological fracture
M8039	Postsurgical malabsorption osteoporosis with pathological fracture
M804	Drug-induced osteoporosis with pathological fracture
M8040	Drug-induced osteoporosis with pathological fracture
M8041	Drug-induced osteoporosis with pathological fracture
M8042	Drug-induced osteoporosis with pathological fracture
M8043	Drug-induced osteoporosis with pathological fracture
M8044	Drug-induced osteoporosis with pathological fracture
M8045	Drug-induced osteoporosis with pathological fracture
M8046	Drug-induced osteoporosis with pathological fracture
M8047	Drug-induced osteoporosis with pathological fracture
M8048	Drug-induced osteoporosis with pathological fracture
M8049	Drug-induced osteoporosis with pathological fracture
M805	Idiopathic osteoporosis with pathological fracture
M8050	Idiopathic osteoporosis with pathological fracture
M8051	Idiopathic osteoporosis with pathological fracture
M8052	Idiopathic osteoporosis with pathological fracture
M8053	Idiopathic osteoporosis with pathological fracture
M8054	Idiopathic osteoporosis with pathological fracture

For peer review only

M8055	Idiopathic osteoporosis with pathological fracture
M8056	Idiopathic osteoporosis with pathological fracture
M8057	Idiopathic osteoporosis with pathological fracture
M8058	Idiopathic osteoporosis with pathological fracture
M8059	Idiopathic osteoporosis with pathological fracture
M808	Other osteoporosis with pathological fracture
M8080	Other osteoporosis with pathological fracture
M8081	Other osteoporosis with pathological fracture
M8082	Other osteoporosis with pathological fracture
M8083	Other osteoporosis with pathological fracture
M8084	Other osteoporosis with pathological fracture
M8085	Other osteoporosis with pathological fracture
M8086	Other osteoporosis with pathological fracture
M8087	Other osteoporosis with pathological fracture
M8088	Other osteoporosis with pathological fracture
M8089	Other osteoporosis with pathological fracture
M809	Unspecified osteoporosis with pathological fracture
M8090	Unspecified osteoporosis with pathological fracture
M8091	Unspecified osteoporosis with pathological fracture
M8092	Unspecified osteoporosis with pathological fracture
M8093	Unspecified osteoporosis with pathological fracture
M8094	Unspecified osteoporosis with pathological fracture
M8095	Unspecified osteoporosis with pathological fracture
M8096	Unspecified osteoporosis with pathological fracture
M8097	Unspecified osteoporosis with pathological fracture
M8098	Unspecified osteoporosis with pathological fracture
M8099	Unspecified osteoporosis with pathological fracture
M81	Osteoporosis without pathological fracture
M810	Postmenopausal osteoporosis
M8100	Postmenopausal osteoporosis
M8101	Postmenopausal osteoporosis
M8102	Postmenopausal osteoporosis
M8103	Postmenopausal osteoporosis
M8104	Postmenopausal osteoporosis
M8105	Postmenopausal osteoporosis
M8106	Postmenopausal osteoporosis
M8107	Postmenopausal osteoporosis
M8108	Postmenopausal osteoporosis
M8109	Postmenopausal osteoporosis
M811	Postoophorectomy osteoporosis
M8110	Postoophorectomy osteoporosis
M8111	Postoophorectomy osteoporosis
M8112	Postoophorectomy osteoporosis
M8113	Postoophorectomy osteoporosis
M8114	Postoophorectomy osteoporosis
M8115	Postoophorectomy osteoporosis
M8116	Postoophorectomy osteoporosis
M8117	Postoophorectomy osteoporosis

For peer review only

M8118	Postoophorectomy osteoporosis
M8119	Postoophorectomy osteoporosis
M812	Osteoporosis of disuse
M8120	Osteoporosis of disuse
M8121	Osteoporosis of disuse
M8122	Osteoporosis of disuse
M8123	Osteoporosis of disuse
M8124	Osteoporosis of disuse
M8125	Osteoporosis of disuse
M8126	Osteoporosis of disuse
M8127	Osteoporosis of disuse
M8128	Osteoporosis of disuse
M8129	Osteoporosis of disuse
M813	Postsurgical malabsorption osteoporosis
M8130	Postsurgical malabsorption osteoporosis
M8131	Postsurgical malabsorption osteoporosis
M8132	Postsurgical malabsorption osteoporosis
M8133	Postsurgical malabsorption osteoporosis
M8134	Postsurgical malabsorption osteoporosis
M8135	Postsurgical malabsorption osteoporosis
M8136	Postsurgical malabsorption osteoporosis
M8137	Postsurgical malabsorption osteoporosis
M8138	Postsurgical malabsorption osteoporosis
M8139	Postsurgical malabsorption osteoporosis
M814	Drug-induced osteoporosis
M8140	Drug-induced osteoporosis
M8141	Drug-induced osteoporosis
M8142	Drug-induced osteoporosis
M8143	Drug-induced osteoporosis
M8144	Drug-induced osteoporosis
M8145	Drug-induced osteoporosis
M8146	Drug-induced osteoporosis
M8147	Drug-induced osteoporosis
M8148	Drug-induced osteoporosis
M8149	Drug-induced osteoporosis
M815	Idiopathic osteoporosis
M8150	Idiopathic osteoporosis
M8151	Idiopathic osteoporosis
M8152	Idiopathic osteoporosis
M8153	Idiopathic osteoporosis
M8154	Idiopathic osteoporosis
M8155	Idiopathic osteoporosis
M8156	Idiopathic osteoporosis
M8157	Idiopathic osteoporosis
M8158	Idiopathic osteoporosis
M8159	Idiopathic osteoporosis
M816	Localized osteoporosis [Lequesne]
M8160	Localized osteoporosis [Lequesne]

For peer review only

M8161	Localized osteoporosis [Lequesne]
M8162	Localized osteoporosis [Lequesne]
M8163	Localized osteoporosis [Lequesne]
M8164	Localized osteoporosis [Lequesne]
M8165	Localized osteoporosis [Lequesne]
M8166	Localized osteoporosis [Lequesne]
M8167	Localized osteoporosis [Lequesne]
M8168	Localized osteoporosis [Lequesne]
M8169	Localized osteoporosis [Lequesne]
M818	Other osteoporosis
M8180	Other osteoporosis
M8181	Other osteoporosis
M8182	Other osteoporosis
M8183	Other osteoporosis
M8184	Other osteoporosis
M8185	Other osteoporosis
M8186	Other osteoporosis
M8187	Other osteoporosis
M8188	Other osteoporosis
M8189	Other osteoporosis
M819	Osteoporosis, unspecified
M8190	Osteoporosis, unspecified
M8191	Osteoporosis, unspecified
M8192	Osteoporosis, unspecified
M8193	Osteoporosis, unspecified
M8194	Osteoporosis, unspecified
M8195	Osteoporosis, unspecified
M8196	Osteoporosis, unspecified
M8197	Osteoporosis, unspecified
M8198	Osteoporosis, unspecified
M8199	Osteoporosis, unspecified
M82	Osteoporosis in diseases classified elsewhere
M820	Osteoporosis in multiple myelomatosis
M8200	Osteoporosis in multiple myelomatosis
M8201	Osteoporosis in multiple myelomatosis
M8202	Osteoporosis in multiple myelomatosis
M8203	Osteoporosis in multiple myelomatosis
M8204	Osteoporosis in multiple myelomatosis
M8205	Osteoporosis in multiple myelomatosis
M8206	Osteoporosis in multiple myelomatosis
M8207	Osteoporosis in multiple myelomatosis
M8208	Osteoporosis in multiple myelomatosis
M8209	Osteoporosis in multiple myelomatosis
M821	Osteoporosis in endocrine disorders
M8210	Osteoporosis in endocrine disorders
M8211	Osteoporosis in endocrine disorders
M8212	Osteoporosis in endocrine disorders
M8213	Osteoporosis in endocrine disorders

For peer review only

M8214	Osteoporosis in endocrine disorders
M8215	Osteoporosis in endocrine disorders
M8216	Osteoporosis in endocrine disorders
M8217	Osteoporosis in endocrine disorders
M8218	Osteoporosis in endocrine disorders
M8219	Osteoporosis in endocrine disorders
M828	Osteoporosis in other diseases classified elsewhere
M8280	Osteoporosis in other diseases classified elsewhere
M8281	Osteoporosis in other diseases classified elsewhere
M8282	Osteoporosis in other diseases classified elsewhere
M8283	Osteoporosis in other diseases classified elsewhere
M8284	Osteoporosis in other diseases classified elsewhere
M8285	Osteoporosis in other diseases classified elsewhere
M8286	Osteoporosis in other diseases classified elsewhere
M8287	Osteoporosis in other diseases classified elsewhere
M8288	Osteoporosis in other diseases classified elsewhere
M8289	Osteoporosis in other diseases classified elsewhere
M83	Adult osteomalacia
M830	Puerperal osteomalacia
M8300	Puerperal osteomalacia
M8301	Puerperal osteomalacia
M8302	Puerperal osteomalacia
M8303	Puerperal osteomalacia
M8304	Puerperal osteomalacia
M8305	Puerperal osteomalacia
M8306	Puerperal osteomalacia
M8307	Puerperal osteomalacia
M8308	Puerperal osteomalacia
M8309	Puerperal osteomalacia
M831	Senile osteomalacia
M8310	Senile osteomalacia
M8311	Senile osteomalacia
M8312	Senile osteomalacia
M8313	Senile osteomalacia
M8314	Senile osteomalacia
M8315	Senile osteomalacia
M8316	Senile osteomalacia
M8317	Senile osteomalacia
M8318	Senile osteomalacia
M8319	Senile osteomalacia
M832	Adult osteomalacia due to malabsorption
M8320	Adult osteomalacia due to malabsorption
M8321	Adult osteomalacia due to malabsorption
M8322	Adult osteomalacia due to malabsorption
M8323	Adult osteomalacia due to malabsorption
M8324	Adult osteomalacia due to malabsorption
M8325	Adult osteomalacia due to malabsorption
M8326	Adult osteomalacia due to malabsorption

For peer review only

M8327	Adult osteomalacia due to malabsorption
M8328	Adult osteomalacia due to malabsorption
M8329	Adult osteomalacia due to malabsorption
M833	Adult osteomalacia due to malnutrition
M8330	Adult osteomalacia due to malnutrition
M8331	Adult osteomalacia due to malnutrition
M8332	Adult osteomalacia due to malnutrition
M8333	Adult osteomalacia due to malnutrition
M8334	Adult osteomalacia due to malnutrition
M8335	Adult osteomalacia due to malnutrition
M8336	Adult osteomalacia due to malnutrition
M8337	Adult osteomalacia due to malnutrition
M8338	Adult osteomalacia due to malnutrition
M8339	Adult osteomalacia due to malnutrition
M834	Aluminium bone disease
M8340	Aluminium bone disease
M8341	Aluminium bone disease
M8342	Aluminium bone disease
M8343	Aluminium bone disease
M8344	Aluminium bone disease
M8345	Aluminium bone disease
M8346	Aluminium bone disease
M8347	Aluminium bone disease
M8348	Aluminium bone disease
M8349	Aluminium bone disease
M835	Other drug-induced osteomalacia in adults
M8350	Other drug-induced osteomalacia in adults
M8351	Other drug-induced osteomalacia in adults
M8352	Other drug-induced osteomalacia in adults
M8353	Other drug-induced osteomalacia in adults
M8354	Other drug-induced osteomalacia in adults
M8355	Other drug-induced osteomalacia in adults
M8356	Other drug-induced osteomalacia in adults
M8357	Other drug-induced osteomalacia in adults
M8358	Other drug-induced osteomalacia in adults
M8359	Other drug-induced osteomalacia in adults
M838	Other adult osteomalacia
M8380	Other adult osteomalacia
M8381	Other adult osteomalacia
M8382	Other adult osteomalacia
M8383	Other adult osteomalacia
M8384	Other adult osteomalacia
M8385	Other adult osteomalacia
M8386	Other adult osteomalacia
M8387	Other adult osteomalacia
M8388	Other adult osteomalacia
M8389	Other adult osteomalacia
M839	Adult osteomalacia, unspecified

For peer review only

M8390	Adult osteomalacia, unspecified
M8391	Adult osteomalacia, unspecified
M8392	Adult osteomalacia, unspecified
M8393	Adult osteomalacia, unspecified
M8394	Adult osteomalacia, unspecified
M8395	Adult osteomalacia, unspecified
M8396	Adult osteomalacia, unspecified
M8397	Adult osteomalacia, unspecified
M8398	Adult osteomalacia, unspecified
M8399	Adult osteomalacia, unspecified
M84	Disorders of continuity of bone
M840	Malunion of fracture
M8400	Malunion of fracture
M8401	Malunion of fracture
M8402	Malunion of fracture
M8403	Malunion of fracture
M8404	Malunion of fracture
M8405	Malunion of fracture
M8406	Malunion of fracture
M8407	Malunion of fracture
M8408	Malunion of fracture
M8409	Malunion of fracture
M841	Nonunion of fracture [pseudarthrosis]
M8410	Nonunion of fracture [pseudarthrosis]
M8411	Nonunion of fracture [pseudarthrosis]
M8412	Nonunion of fracture [pseudarthrosis]
M8413	Nonunion of fracture [pseudarthrosis]
M8414	Nonunion of fracture [pseudarthrosis]
M8415	Nonunion of fracture [pseudarthrosis]
M8416	Nonunion of fracture [pseudarthrosis]
M8417	Nonunion of fracture [pseudarthrosis]
M8418	Nonunion of fracture [pseudarthrosis]
M8419	Nonunion of fracture [pseudarthrosis]
M842	Delayed union of fracture
M8420	Delayed union of fracture
M8421	Delayed union of fracture
M8422	Delayed union of fracture
M8423	Delayed union of fracture
M8424	Delayed union of fracture
M8425	Delayed union of fracture
M8426	Delayed union of fracture
M8427	Delayed union of fracture
M8428	Delayed union of fracture
M8429	Delayed union of fracture
M843	Stress fracture, not elsewhere classified
M8430	Stress fracture, not elsewhere classified
M8431	Stress fracture, not elsewhere classified
M8432	Stress fracture, not elsewhere classified

For peer review only

M8433	Stress fracture, not elsewhere classified
M8434	Stress fracture, not elsewhere classified
M8435	Stress fracture, not elsewhere classified
M8436	Stress fracture, not elsewhere classified
M8437	Stress fracture, not elsewhere classified
M8438	Stress fracture, not elsewhere classified
M8439	Stress fracture, not elsewhere classified
M844	Pathological fracture, not elsewhere classified
M8440	Pathological fracture, not elsewhere classified
M8441	Pathological fracture, not elsewhere classified
M8442	Pathological fracture, not elsewhere classified
M8443	Pathological fracture, not elsewhere classified
M8444	Pathological fracture, not elsewhere classified
M8445	Pathological fracture, not elsewhere classified
M8446	Pathological fracture, not elsewhere classified
M8447	Pathological fracture, not elsewhere classified
M8448	Pathological fracture, not elsewhere classified
M8449	Pathological fracture, not elsewhere classified
M848	Other disorders of continuity of bone
M8480	Other disorders of continuity of bone
M8481	Other disorders of continuity of bone
M8482	Other disorders of continuity of bone
M8483	Other disorders of continuity of bone
M8484	Other disorders of continuity of bone
M8485	Other disorders of continuity of bone
M8486	Other disorders of continuity of bone
M8487	Other disorders of continuity of bone
M8488	Other disorders of continuity of bone
M8489	Other disorders of continuity of bone
M849	Disorder of continuity of bone, unspecified
M8490	Disorder of continuity of bone, unspecified
M8491	Disorder of continuity of bone, unspecified
M8492	Disorder of continuity of bone, unspecified
M8493	Disorder of continuity of bone, unspecified
M8494	Disorder of continuity of bone, unspecified
M8495	Disorder of continuity of bone, unspecified
M8496	Disorder of continuity of bone, unspecified
M8497	Disorder of continuity of bone, unspecified
M8498	Disorder of continuity of bone, unspecified
M8499	Disorder of continuity of bone, unspecified
M85	Other disorders of bone density and structure
M850	Fibrous dysplasia (monostotic)
M8500	Fibrous dysplasia (monostotic)
M8501	Fibrous dysplasia (monostotic)
M8502	Fibrous dysplasia (monostotic)
M8503	Fibrous dysplasia (monostotic)
M8504	Fibrous dysplasia (monostotic)
M8505	Fibrous dysplasia (monostotic)

For peer review only

M8506	Fibrous dysplasia (monostotic)
M8507	Fibrous dysplasia (monostotic)
M8508	Fibrous dysplasia (monostotic)
M8509	Fibrous dysplasia (monostotic)
M851	Skeletal fluorosis
M8510	Skeletal fluorosis
M8511	Skeletal fluorosis
M8512	Skeletal fluorosis
M8513	Skeletal fluorosis
M8514	Skeletal fluorosis
M8515	Skeletal fluorosis
M8516	Skeletal fluorosis
M8517	Skeletal fluorosis
M8518	Skeletal fluorosis
M8519	Skeletal fluorosis
M852	Hyperostosis of skull
M853	Osteitis condensans
M8530	Osteitis condensans
M8531	Osteitis condensans
M8532	Osteitis condensans
M8533	Osteitis condensans
M8534	Osteitis condensans
M8535	Osteitis condensans
M8536	Osteitis condensans
M8537	Osteitis condensans
M8538	Osteitis condensans
M8539	Osteitis condensans
M854	Solitary bone cyst
M8540	Solitary bone cyst
M8541	Solitary bone cyst
M8542	Solitary bone cyst
M8543	Solitary bone cyst
M8544	Solitary bone cyst
M8545	Solitary bone cyst
M8546	Solitary bone cyst
M8547	Solitary bone cyst
M8548	Solitary bone cyst
M8549	Solitary bone cyst
M855	Aneurysmal bone cyst
M8550	Aneurysmal bone cyst
M8551	Aneurysmal bone cyst
M8552	Aneurysmal bone cyst
M8553	Aneurysmal bone cyst
M8554	Aneurysmal bone cyst
M8555	Aneurysmal bone cyst
M8556	Aneurysmal bone cyst
M8557	Aneurysmal bone cyst
M8558	Aneurysmal bone cyst

For peer review only

M8559	Aneurysmal bone cyst
M856	Other cyst of bone
M8560	Other cyst of bone
M8561	Other cyst of bone
M8562	Other cyst of bone
M8563	Other cyst of bone
M8564	Other cyst of bone
M8565	Other cyst of bone
M8566	Other cyst of bone
M8567	Other cyst of bone
M8568	Other cyst of bone
M8569	Other cyst of bone
M858	Other specified disorders of bone density and structure
M8580	Other specified disorders of bone density and structure
M8581	Other specified disorders of bone density and structure
M8582	Other specified disorders of bone density and structure
M8583	Other specified disorders of bone density and structure
M8584	Other specified disorders of bone density and structure
M8585	Other specified disorders of bone density and structure
M8586	Other specified disorders of bone density and structure
M8587	Other specified disorders of bone density and structure
M8588	Other specified disorders of bone density and structure
M8589	Other specified disorders of bone density and structure
M859	Disorder of bone density and structure, unspecified
M8590	Disorder of bone density and structure, unspecified
M8591	Disorder of bone density and structure, unspecified
M8592	Disorder of bone density and structure, unspecified
M8593	Disorder of bone density and structure, unspecified
M8594	Disorder of bone density and structure, unspecified
M8595	Disorder of bone density and structure, unspecified
M8596	Disorder of bone density and structure, unspecified
M8597	Disorder of bone density and structure, unspecified
M8598	Disorder of bone density and structure, unspecified
M8599	Disorder of bone density and structure, unspecified
M86	Osteomyelitis
M860	Acute haematogenous osteomyelitis
M8600	Acute haematogenous osteomyelitis
M8601	Acute haematogenous osteomyelitis
M8602	Acute haematogenous osteomyelitis
M8603	Acute haematogenous osteomyelitis
M8604	Acute haematogenous osteomyelitis
M8605	Acute haematogenous osteomyelitis
M8606	Acute haematogenous osteomyelitis
M8607	Acute haematogenous osteomyelitis
M8608	Acute haematogenous osteomyelitis
M8609	Acute haematogenous osteomyelitis
M861	Other acute osteomyelitis
M8610	Other acute osteomyelitis

For peer review only

M8611	Other acute osteomyelitis
M8612	Other acute osteomyelitis
M8613	Other acute osteomyelitis
M8614	Other acute osteomyelitis
M8615	Other acute osteomyelitis
M8616	Other acute osteomyelitis
M8617	Other acute osteomyelitis
M8618	Other acute osteomyelitis
M8619	Other acute osteomyelitis
M862	Subacute osteomyelitis
M8620	Subacute osteomyelitis
M8621	Subacute osteomyelitis
M8622	Subacute osteomyelitis
M8623	Subacute osteomyelitis
M8624	Subacute osteomyelitis
M8625	Subacute osteomyelitis
M8626	Subacute osteomyelitis
M8627	Subacute osteomyelitis
M8628	Subacute osteomyelitis
M8629	Subacute osteomyelitis
M863	Chronic multifocal osteomyelitis
M8630	Chronic multifocal osteomyelitis
M8631	Chronic multifocal osteomyelitis
M8632	Chronic multifocal osteomyelitis
M8633	Chronic multifocal osteomyelitis
M8634	Chronic multifocal osteomyelitis
M8635	Chronic multifocal osteomyelitis
M8636	Chronic multifocal osteomyelitis
M8637	Chronic multifocal osteomyelitis
M8638	Chronic multifocal osteomyelitis
M8639	Chronic multifocal osteomyelitis
M864	Chronic osteomyelitis with draining sinus
M8640	Chronic osteomyelitis with draining sinus
M8641	Chronic osteomyelitis with draining sinus
M8642	Chronic osteomyelitis with draining sinus
M8643	Chronic osteomyelitis with draining sinus
M8644	Chronic osteomyelitis with draining sinus
M8645	Chronic osteomyelitis with draining sinus
M8646	Chronic osteomyelitis with draining sinus
M8647	Chronic osteomyelitis with draining sinus
M8648	Chronic osteomyelitis with draining sinus
M8649	Chronic osteomyelitis with draining sinus
M865	Other chronic haematogenous osteomyelitis
M8650	Other chronic haematogenous osteomyelitis
M8651	Other chronic haematogenous osteomyelitis
M8652	Other chronic haematogenous osteomyelitis
M8653	Other chronic haematogenous osteomyelitis
M8654	Other chronic haematogenous osteomyelitis

For peer review only

M8655	Other chronic haematogenous osteomyelitis
M8656	Other chronic haematogenous osteomyelitis
M8657	Other chronic haematogenous osteomyelitis
M8658	Other chronic haematogenous osteomyelitis
M8659	Other chronic haematogenous osteomyelitis
M866	Other chronic osteomyelitis
M8660	Other chronic osteomyelitis
M8661	Other chronic osteomyelitis
M8662	Other chronic osteomyelitis
M8663	Other chronic osteomyelitis
M8664	Other chronic osteomyelitis
M8665	Other chronic osteomyelitis
M8666	Other chronic osteomyelitis
M8667	Other chronic osteomyelitis
M8668	Other chronic osteomyelitis
M8669	Other chronic osteomyelitis
M868	Other osteomyelitis
M8680	Other osteomyelitis
M8681	Other osteomyelitis
M8682	Other osteomyelitis
M8683	Other osteomyelitis
M8684	Other osteomyelitis
M8685	Other osteomyelitis
M8686	Other osteomyelitis
M8687	Other osteomyelitis
M8688	Other osteomyelitis
M8689	Other osteomyelitis
M869	Osteomyelitis, unspecified
M8690	Osteomyelitis, unspecified
M8691	Osteomyelitis, unspecified
M8692	Osteomyelitis, unspecified
M8693	Osteomyelitis, unspecified
M8694	Osteomyelitis, unspecified
M8695	Osteomyelitis, unspecified
M8696	Osteomyelitis, unspecified
M8697	Osteomyelitis, unspecified
M8698	Osteomyelitis, unspecified
M8699	Osteomyelitis, unspecified
M87	Osteonecrosis
M870	Idiopathic aseptic necrosis of bone
M8700	Idiopathic aseptic necrosis of bone
M8701	Idiopathic aseptic necrosis of bone
M8702	Idiopathic aseptic necrosis of bone
M8703	Idiopathic aseptic necrosis of bone
M8704	Idiopathic aseptic necrosis of bone
M8705	Idiopathic aseptic necrosis of bone
M8706	Idiopathic aseptic necrosis of bone
M8707	Idiopathic aseptic necrosis of bone

For peer review only

M8708	Idiopathic aseptic necrosis of bone
M8709	Idiopathic aseptic necrosis of bone
M871	Osteonecrosis due to drugs
M8710	Osteonecrosis due to drugs
M8711	Osteonecrosis due to drugs
M8712	Osteonecrosis due to drugs
M8713	Osteonecrosis due to drugs
M8714	Osteonecrosis due to drugs
M8715	Osteonecrosis due to drugs
M8716	Osteonecrosis due to drugs
M8717	Osteonecrosis due to drugs
M8718	Osteonecrosis due to drugs
M8719	Osteonecrosis due to drugs
M872	Osteonecrosis due to previous trauma
M8720	Osteonecrosis due to previous trauma
M8721	Osteonecrosis due to previous trauma
M8722	Osteonecrosis due to previous trauma
M8723	Osteonecrosis due to previous trauma
M8724	Osteonecrosis due to previous trauma
M8725	Osteonecrosis due to previous trauma
M8726	Osteonecrosis due to previous trauma
M8727	Osteonecrosis due to previous trauma
M8728	Osteonecrosis due to previous trauma
M8729	Osteonecrosis due to previous trauma
M873	Other secondary osteonecrosis
M8730	Other secondary osteonecrosis
M8731	Other secondary osteonecrosis
M8732	Other secondary osteonecrosis
M8733	Other secondary osteonecrosis
M8734	Other secondary osteonecrosis
M8735	Other secondary osteonecrosis
M8736	Other secondary osteonecrosis
M8737	Other secondary osteonecrosis
M8738	Other secondary osteonecrosis
M8739	Other secondary osteonecrosis
M878	Other osteonecrosis
M8780	Other osteonecrosis
M8781	Other osteonecrosis
M8782	Other osteonecrosis
M8783	Other osteonecrosis
M8784	Other osteonecrosis
M8785	Other osteonecrosis
M8786	Other osteonecrosis
M8787	Other osteonecrosis
M8788	Other osteonecrosis
M8789	Other osteonecrosis
M879	Osteonecrosis, unspecified
M8790	Osteonecrosis, unspecified

For peer review only

M8791	Osteonecrosis, unspecified
M8792	Osteonecrosis, unspecified
M8793	Osteonecrosis, unspecified
M8794	Osteonecrosis, unspecified
M8795	Osteonecrosis, unspecified
M8796	Osteonecrosis, unspecified
M8797	Osteonecrosis, unspecified
M8798	Osteonecrosis, unspecified
M8799	Osteonecrosis, unspecified
M88	Paget disease of bone [osteitis deformans]
M88	Paget's disease of bone [osteitis deformans]
M880	Paget disease of skull
M880	Paget's disease of skull
M888	Paget disease of other bones
M888	Paget's disease of other bones
M8880	Paget disease of other bones
M8880	Paget's disease of other bones
M8881	Paget disease of other bones
M8881	Paget's disease of other bones
M8882	Paget disease of other bones
M8882	Paget's disease of other bones
M8883	Paget disease of other bones
M8883	Paget's disease of other bones
M8884	Paget disease of other bones
M8884	Paget's disease of other bones
M8885	Paget disease of other bones
M8885	Paget's disease of other bones
M8886	Paget disease of other bones
M8886	Paget's disease of other bones
M8887	Paget disease of other bones
M8887	Paget's disease of other bones
M8888	Paget disease of other bones
M8888	Paget's disease of other bones
M8889	Paget disease of other bones
M8889	Paget's disease of other bones
M889	Paget disease of bone, unspecified
M889	Paget's disease of bone, unspecified
M8890	Paget disease of bone, unspecified
M8890	Paget's disease of bone, unspecified
M8891	Paget disease of bone, unspecified
M8891	Paget's disease of bone, unspecified
M8892	Paget disease of bone, unspecified
M8892	Paget's disease of bone, unspecified
M8893	Paget disease of bone, unspecified
M8893	Paget's disease of bone, unspecified
M8894	Paget disease of bone, unspecified
M8894	Paget's disease of bone, unspecified
M8895	Paget disease of bone, unspecified

For peer review only

M8895	Paget's disease of bone, unspecified
M8896	Paget disease of bone, unspecified
M8896	Paget's disease of bone, unspecified
M8897	Paget disease of bone, unspecified
M8897	Paget's disease of bone, unspecified
M8898	Paget disease of bone, unspecified
M8898	Paget's disease of bone, unspecified
M8899	Paget disease of bone, unspecified
M8899	Paget's disease of bone, unspecified
M89	Other disorders of bone
M890	Algoneurodystrophy
M8900	Algoneurodystrophy
M8901	Algoneurodystrophy
M8902	Algoneurodystrophy
M8903	Algoneurodystrophy
M8904	Algoneurodystrophy
M8905	Algoneurodystrophy
M8906	Algoneurodystrophy
M8907	Algoneurodystrophy
M8908	Algoneurodystrophy
M8909	Algoneurodystrophy
M891	Epiphyseal arrest
M8910	Epiphyseal arrest
M8911	Epiphyseal arrest
M8912	Epiphyseal arrest
M8913	Epiphyseal arrest
M8914	Epiphyseal arrest
M8915	Epiphyseal arrest
M8916	Epiphyseal arrest
M8917	Epiphyseal arrest
M8918	Epiphyseal arrest
M8919	Epiphyseal arrest
M892	Other disorders of bone development and growth
M8920	Other disorders of bone development and growth
M8921	Other disorders of bone development and growth
M8922	Other disorders of bone development and growth
M8923	Other disorders of bone development and growth
M8924	Other disorders of bone development and growth
M8925	Other disorders of bone development and growth
M8926	Other disorders of bone development and growth
M8927	Other disorders of bone development and growth
M8928	Other disorders of bone development and growth
M8929	Other disorders of bone development and growth
M893	Hypertrophy of bone
M8930	Hypertrophy of bone
M8931	Hypertrophy of bone
M8932	Hypertrophy of bone
M8933	Hypertrophy of bone

For peer review only

M8934	Hypertrophy of bone
M8935	Hypertrophy of bone
M8936	Hypertrophy of bone
M8937	Hypertrophy of bone
M8938	Hypertrophy of bone
M8939	Hypertrophy of bone
M894	Other hypertrophic osteoarthropathy
M8940	Other hypertrophic osteoarthropathy
M8941	Other hypertrophic osteoarthropathy
M8942	Other hypertrophic osteoarthropathy
M8943	Other hypertrophic osteoarthropathy
M8944	Other hypertrophic osteoarthropathy
M8945	Other hypertrophic osteoarthropathy
M8946	Other hypertrophic osteoarthropathy
M8947	Other hypertrophic osteoarthropathy
M8948	Other hypertrophic osteoarthropathy
M8949	Other hypertrophic osteoarthropathy
M895	Osteolysis
M8950	Osteolysis
M8951	Osteolysis
M8952	Osteolysis
M8953	Osteolysis
M8954	Osteolysis
M8955	Osteolysis
M8956	Osteolysis
M8957	Osteolysis
M8958	Osteolysis
M8959	Osteolysis
M896	Osteopathy after poliomyelitis
M8960	Osteopathy after poliomyelitis
M8961	Osteopathy after poliomyelitis
M8962	Osteopathy after poliomyelitis
M8963	Osteopathy after poliomyelitis
M8964	Osteopathy after poliomyelitis
M8965	Osteopathy after poliomyelitis
M8966	Osteopathy after poliomyelitis
M8967	Osteopathy after poliomyelitis
M8968	Osteopathy after poliomyelitis
M8969	Osteopathy after poliomyelitis
M898	Other specified disorders of bone
M8980	Other specified disorders of bone
M8981	Other specified disorders of bone
M8982	Other specified disorders of bone
M8983	Other specified disorders of bone
M8984	Other specified disorders of bone
M8985	Other specified disorders of bone
M8986	Other specified disorders of bone
M8987	Other specified disorders of bone

For peer review only

M8988	Other specified disorders of bone
M8989	Other specified disorders of bone
M899	Disorder of bone, unspecified
M8990	Disorder of bone, unspecified
M8991	Disorder of bone, unspecified
M8992	Disorder of bone, unspecified
M8993	Disorder of bone, unspecified
M8994	Disorder of bone, unspecified
M8995	Disorder of bone, unspecified
M8996	Disorder of bone, unspecified
M8997	Disorder of bone, unspecified
M8998	Disorder of bone, unspecified
M8999	Disorder of bone, unspecified
M90	Osteopathies in diseases classified elsewhere
M900	Tuberculosis of bone
M9000	Tuberculosis of bone
M9001	Tuberculosis of bone
M9002	Tuberculosis of bone
M9003	Tuberculosis of bone
M9004	Tuberculosis of bone
M9005	Tuberculosis of bone
M9006	Tuberculosis of bone
M9007	Tuberculosis of bone
M9008	Tuberculosis of bone
M9009	Tuberculosis of bone
M901	Periostitis in other infectious diseases classified elsewhere
M9010	Periostitis in other infectious diseases classified elsewhere
M9011	Periostitis in other infectious diseases classified elsewhere
M9012	Periostitis in other infectious diseases classified elsewhere
M9013	Periostitis in other infectious diseases classified elsewhere
M9014	Periostitis in other infectious diseases classified elsewhere
M9015	Periostitis in other infectious diseases classified elsewhere
M9016	Periostitis in other infectious diseases classified elsewhere
M9017	Periostitis in other infectious diseases classified elsewhere
M9018	Periostitis in other infectious diseases classified elsewhere
M9019	Periostitis in other infectious diseases classified elsewhere
M902	Osteopathy in other infectious diseases classified elsewhere
M9020	Osteopathy in other infectious diseases classified elsewhere
M9021	Osteopathy in other infectious diseases classified elsewhere
M9022	Osteopathy in other infectious diseases classified elsewhere
M9023	Osteopathy in other infectious diseases classified elsewhere
M9024	Osteopathy in other infectious diseases classified elsewhere
M9025	Osteopathy in other infectious diseases classified elsewhere
M9026	Osteopathy in other infectious diseases classified elsewhere
M9027	Osteopathy in other infectious diseases classified elsewhere
M9028	Osteopathy in other infectious diseases classified elsewhere
M9029	Osteopathy in other infectious diseases classified elsewhere
M903	Osteonecrosis in caisson disease

For peer review only

M9030	Osteonecrosis in caisson disease
M9031	Osteonecrosis in caisson disease
M9032	Osteonecrosis in caisson disease
M9033	Osteonecrosis in caisson disease
M9034	Osteonecrosis in caisson disease
M9035	Osteonecrosis in caisson disease
M9036	Osteonecrosis in caisson disease
M9037	Osteonecrosis in caisson disease
M9038	Osteonecrosis in caisson disease
M9039	Osteonecrosis in caisson disease
M904	Osteonecrosis due to haemoglobinopathy
M9040	Osteonecrosis due to haemoglobinopathy
M9041	Osteonecrosis due to haemoglobinopathy
M9042	Osteonecrosis due to haemoglobinopathy
M9043	Osteonecrosis due to haemoglobinopathy
M9044	Osteonecrosis due to haemoglobinopathy
M9045	Osteonecrosis due to haemoglobinopathy
M9046	Osteonecrosis due to haemoglobinopathy
M9047	Osteonecrosis due to haemoglobinopathy
M9048	Osteonecrosis due to haemoglobinopathy
M9049	Osteonecrosis due to haemoglobinopathy
M905	Osteonecrosis in other diseases classified elsewhere
M9050	Osteonecrosis in other diseases classified elsewhere
M9051	Osteonecrosis in other diseases classified elsewhere
M9052	Osteonecrosis in other diseases classified elsewhere
M9053	Osteonecrosis in other diseases classified elsewhere
M9054	Osteonecrosis in other diseases classified elsewhere
M9055	Osteonecrosis in other diseases classified elsewhere
M9056	Osteonecrosis in other diseases classified elsewhere
M9057	Osteonecrosis in other diseases classified elsewhere
M9058	Osteonecrosis in other diseases classified elsewhere
M9059	Osteonecrosis in other diseases classified elsewhere
M906	Osteitis deformans in neoplastic disease
M9060	Osteitis deformans in neoplastic disease
M9061	Osteitis deformans in neoplastic disease
M9062	Osteitis deformans in neoplastic disease
M9063	Osteitis deformans in neoplastic disease
M9064	Osteitis deformans in neoplastic disease
M9065	Osteitis deformans in neoplastic disease
M9066	Osteitis deformans in neoplastic disease
M9067	Osteitis deformans in neoplastic disease
M9068	Osteitis deformans in neoplastic disease
M9069	Osteitis deformans in neoplastic disease
M907	Fracture of bone in neoplastic disease
M9070	Fracture of bone in neoplastic disease
M9071	Fracture of bone in neoplastic disease
M9072	Fracture of bone in neoplastic disease
M9073	Fracture of bone in neoplastic disease

For peer review only

M9074	Fracture of bone in neoplastic disease
M9075	Fracture of bone in neoplastic disease
M9076	Fracture of bone in neoplastic disease
M9077	Fracture of bone in neoplastic disease
M9078	Fracture of bone in neoplastic disease
M9079	Fracture of bone in neoplastic disease
M908	Osteopathy in other diseases classified elsewhere
M9080	Osteopathy in other diseases classified elsewhere
M9081	Osteopathy in other diseases classified elsewhere
M9082	Osteopathy in other diseases classified elsewhere
M9083	Osteopathy in other diseases classified elsewhere
M9084	Osteopathy in other diseases classified elsewhere
M9085	Osteopathy in other diseases classified elsewhere
M9086	Osteopathy in other diseases classified elsewhere
M9087	Osteopathy in other diseases classified elsewhere
M9088	Osteopathy in other diseases classified elsewhere
M9089	Osteopathy in other diseases classified elsewhere
M91	Juvenile osteochondrosis of hip and pelvis
M910	Juvenile osteochondrosis of pelvis
M911	Juvenile osteochondrosis of head of femur [Legg-Calv,-Perthes]
M912	Coxa plana
M913	Pseudocoxalgia
M918	Other juvenile osteochondrosis of hip and pelvis
M9180	Other juvenile osteochondrosis of hip and pelvis
M919	Juvenile osteochondrosis of hip and pelvis, unspecified
M9190	Juvenile osteochondrosis of hip and pelvis, unspecified
M92	Other juvenile osteochondrosis
M920	Juvenile osteochondrosis of humerus
M921	Juvenile osteochondrosis of radius and ulna
M922	Juvenile osteochondrosis of hand
M923	Other juvenile osteochondrosis of upper limb
M924	Juvenile osteochondrosis of patella
M925	Juvenile osteochondrosis of tibia and fibula
M926	Juvenile osteochondrosis of tarsus
M927	Juvenile osteochondrosis of metatarsus
M928	Other specified juvenile osteochondrosis
M929	Juvenile osteochondrosis, unspecified
M93	Other osteochondropathies
M930	Slipped upper femoral epiphysis (nontraumatic)
M931	Kienb”ck disease of adults
M931	Kienb”ck's disease of adults
M932	Osteochondritis dissecans
M938	Other specified osteochondropathies
M939	Osteochondropathy, unspecified
M94	Other disorders of cartilage
M940	Chondrocostal junction syndrome [Tietze]
M941	Relapsing polychondritis
M9410	Relapsing polychondritis

For peer review only

M9411	Relapsing polychondritis
M9412	Relapsing polychondritis
M9413	Relapsing polychondritis
M9414	Relapsing polychondritis
M9415	Relapsing polychondritis
M9416	Relapsing polychondritis
M9417	Relapsing polychondritis
M9418	Relapsing polychondritis
M9419	Relapsing polychondritis
M942	Chondromalacia
M9420	Chondromalacia
M9421	Chondromalacia
M9422	Chondromalacia
M9423	Chondromalacia
M9424	Chondromalacia
M9425	Chondromalacia
M9426	Chondromalacia
M9427	Chondromalacia
M9428	Chondromalacia
M9429	Chondromalacia
M943	Chondrolysis
M9430	Chondrolysis
M9431	Chondrolysis
M9432	Chondrolysis
M9433	Chondrolysis
M9434	Chondrolysis
M9435	Chondrolysis
M9436	Chondrolysis
M9437	Chondrolysis
M9438	Chondrolysis
M9439	Chondrolysis
M948	Other specified disorders of cartilage
M9480	Other specified disorders of cartilage
M9481	Other specified disorders of cartilage
M9482	Other specified disorders of cartilage
M9483	Other specified disorders of cartilage
M9484	Other specified disorders of cartilage
M9485	Other specified disorders of cartilage
M9486	Other specified disorders of cartilage
M9487	Other specified disorders of cartilage
M9488	Other specified disorders of cartilage
M9489	Other specified disorders of cartilage
M949	Disorder of cartilage, unspecified
M9490	Disorder of cartilage, unspecified
M9491	Disorder of cartilage, unspecified
M9492	Disorder of cartilage, unspecified
M9493	Disorder of cartilage, unspecified
M9494	Disorder of cartilage, unspecified

For peer review only

M9495	Disorder of cartilage, unspecified
M9496	Disorder of cartilage, unspecified
M9497	Disorder of cartilage, unspecified
M9498	Disorder of cartilage, unspecified
M9499	Disorder of cartilage, unspecified
M95	Other acquired deformities of musculoskeletal system and connective tissue
M950	Acquired deformity of nose
M951	Cauliflower ear
M952	Other acquired deformity of head
M953	Acquired deformity of neck
M954	Acquired deformity of chest and rib
M955	Acquired deformity of pelvis
M958	Other specified acquired deformities of musculoskeletal system
M959	Acquired deformity of musculoskeletal system, unspecified
M96	Postprocedural musculoskeletal disorders, not elsewhere classified
M960	Pseudarthrosis after fusion or arthrodesis
M961	Postlaminectomy syndrome, not elsewhere classified
M962	Postradiation kyphosis
M963	Postlaminectomy kyphosis
M964	Postsurgical lordosis
M965	Postradiation scoliosis
M966	Fracture of bone following insertion of orthopaedic implant, joint prosthesis, or bone plate
M968	Other postprocedural musculoskeletal disorders
M969	Postprocedural musculoskeletal disorder, unspecified
M99	Biomechanical lesions, not elsewhere classified
M990	Segmental and somatic dysfunction
M9900	Segmental and somatic dysfunction
M9901	Segmental and somatic dysfunction
M9902	Segmental and somatic dysfunction
M9903	Segmental and somatic dysfunction
M9904	Segmental and somatic dysfunction
M9905	Segmental and somatic dysfunction
M9906	Segmental and somatic dysfunction
M9907	Segmental and somatic dysfunction
M9908	Segmental and somatic dysfunction
M9909	Segmental and somatic dysfunction
M991	Subluxation complex (vertebral)
M9910	Subluxation complex (vertebral)
M9911	Subluxation complex (vertebral)
M9912	Subluxation complex (vertebral)
M9913	Subluxation complex (vertebral)
M9914	Subluxation complex (vertebral)
M9915	Subluxation complex (vertebral)
M9916	Subluxation complex (vertebral)
M9917	Subluxation complex (vertebral)
M9918	Subluxation complex (vertebral)
M9919	Subluxation complex (vertebral)

For peer review only

M992	Subluxation stenosis of neural canal
M9920	Subluxation stenosis of neural canal
M9921	Subluxation stenosis of neural canal
M9922	Subluxation stenosis of neural canal
M9923	Subluxation stenosis of neural canal
M9924	Subluxation stenosis of neural canal
M9925	Subluxation stenosis of neural canal
M993	Osseous stenosis of neural canal
M9930	Osseous stenosis of neural canal
M9931	Osseous stenosis of neural canal
M9932	Osseous stenosis of neural canal
M9933	Osseous stenosis of neural canal
M9934	Osseous stenosis of neural canal
M9935	Osseous stenosis of neural canal
M994	Connective tissue stenosis of neural canal
M9940	Connective tissue stenosis of neural canal
M9941	Connective tissue stenosis of neural canal
M9942	Connective tissue stenosis of neural canal
M9943	Connective tissue stenosis of neural canal
M9944	Connective tissue stenosis of neural canal
M9945	Connective tissue stenosis of neural canal
M995	Intervertebral disc stenosis of neural canal
M9950	Intervertebral disc stenosis of neural canal
M9951	Intervertebral disc stenosis of neural canal
M9952	Intervertebral disc stenosis of neural canal
M9953	Intervertebral disc stenosis of neural canal
M9954	Intervertebral disc stenosis of neural canal
M9955	Intervertebral disc stenosis of neural canal
M996	Osseous and subluxation stenosis of intervertebral foramina
M9960	Osseous and subluxation stenosis of intervertebral foramina
M9961	Osseous and subluxation stenosis of intervertebral foramina
M9962	Osseous and subluxation stenosis of intervertebral foramina
M9963	Osseous and subluxation stenosis of intervertebral foramina
M9964	Osseous and subluxation stenosis of intervertebral foramina
M9965	Osseous and subluxation stenosis of intervertebral foramina
M997	Connective tissue and disc stenosis of intervertebral foramina
M9970	Connective tissue and disc stenosis of intervertebral foramina
M9971	Connective tissue and disc stenosis of intervertebral foramina
M9972	Connective tissue and disc stenosis of intervertebral foramina
M9973	Connective tissue and disc stenosis of intervertebral foramina
M9974	Connective tissue and disc stenosis of intervertebral foramina
M9975	Connective tissue and disc stenosis of intervertebral foramina
M998	Other biomechanical lesions
M9980	Other biomechanical lesions
M9981	Other biomechanical lesions
M9982	Other biomechanical lesions
M9983	Other biomechanical lesions
M9984	Other biomechanical lesions

For peer review only

M9985	Other biomechanical lesions
M9986	Other biomechanical lesions
M9987	Other biomechanical lesions
M9988	Other biomechanical lesions
M9989	Other biomechanical lesions
M999	Biomechanical lesion, unspecified
M9990	Biomechanical lesion, unspecified
M9991	Biomechanical lesion, unspecified
M9992	Biomechanical lesion, unspecified
M9993	Biomechanical lesion, unspecified
M9994	Biomechanical lesion, unspecified
M9995	Biomechanical lesion, unspecified
M9996	Biomechanical lesion, unspecified
M9997	Biomechanical lesion, unspecified
M9998	Biomechanical lesion, unspecified
M9999	Biomechanical lesion, unspecified

Epilepsy (GP)

READ_CODE	READ_DESC
1O30.	Epilepsy confirmed
F25..	Epilepsy
F250.	Generalised nonconvulsive epilepsy
F2500	Petit mal (minor) epilepsy
F2501	Pykno-epilepsy
F2502	Epileptic seizures - atonic
F2503	Epileptic seizures - akinetic
F2504	Juvenile absence epilepsy
F2505	Lennox-Gastaut syndrome
F250y	Other specified generalised nonconvulsive epilepsy
F250z	Generalised nonconvulsive epilepsy NOS
F251.	Generalised convulsive epilepsy
F2510	Grand mal (major) epilepsy
F2511	Neonatal myoclonic epilepsy
F2512	Epileptic seizures - clonic
F2513	Epileptic seizures - myoclonic
F2514	Epileptic seizures - tonic
F2515	Tonic-clonic epilepsy
F2516	Grand mal seizure
F251y	Other specified generalised convulsive epilepsy
F251z	Generalised convulsive epilepsy NOS
F252.	Petit mal status
F253.	Grand mal status
F254.	Partial epilepsy with impairment of consciousness
F2540	Temporal lobe epilepsy
F2541	Psychomotor epilepsy

For peer review only

F2542	Psychosensory epilepsy
F2543	Limbic system epilepsy
F2544	Epileptic automatism
F2545	Complex partial epileptic seizure
F254z	Partial epilepsy with impairment of consciousness NOS
F255.	Partial epilepsy without mention of impairment of consciousness
F2550	Jacksonian, focal or motor epilepsy
F2551	Sensory induced epilepsy
F2552	Somatosensory epilepsy
F2553	Visceral reflex epilepsy
F2554	Visual reflex epilepsy
F2555	Unilateral epilepsy
F2556	Simple partial epileptic seizure
F255y	Other specified partial epilepsy without mention of impairment of consciousness
F255z	Partial epilepsy without mention of impairment of consciousness NOS
F256.	Infantile spasms
F2560	Hypsarrhythmia
F2561	Salaam attacks
F256z	Infantile spasms NOS
F257.	Kojevnikov's epilepsy
F258.	Post-ictal state
F259.	Early infantile epileptic encephalopathy with suppression bursts
F25A.	Juvenile myoclonic epilepsy
F25B.	Alcohol-induced epilepsy
F25C.	Drug-induced epilepsy
F25D.	Menstrual epilepsy
F25E.	Stress-induced epilepsy
F25F.	Photosensitive epilepsy
F25G.	Severe myoclonic epilepsy in infancy
F25H.	Generalised seizure
F25X.	Status epilepticus, unspecified
F25y.	Other forms of epilepsy
F25y0	Cursive (running) epilepsy
F25y1	Gelastic epilepsy
F25y2	Localization-related(focal)(partial)idiopathic epilepsy and epileptic syndromes with seizures of localised onset
F25y3	Complex partial status epilepticus
F25y4	Benign Rolandic epilepsy
F25y5	Panayiotopoulos syndrome
F25yz	Other forms of epilepsy NOS
F25z.	Epilepsy NOS
dn...	CONTROL OF EPILEPSY
dn1..	ACETAZOLAMIDE [EPILEPSY]
dn11.	DIAMOX [EP] 500mg m/r capsules
dn12.	DIAMOX [EP] 250mg tablets
dn13.	DIAMOX [EP] 500mg injection
dn1x.	ACETAZOLAMIDE [EP] 500mg m/r capsules
dn1y.	ACETAZOLAMIDE [EP] 250mg tablets

For peer review only

dn1z.	ACETAZOLAMIDE [EP] 500mg injection
dn2..	*BECLAMIDE
dn21.	*NYDRANE 500mg tablets
dn2z.	*BECLAMIDE 500mg tablets
dn3..	CARBAMAZEPINE
dn31.	CARBAMAZEPINE 100mg tablets
dn32.	CARBAMAZEPINE 200mg tablets
dn33.	CARBAMAZEPINE 400mg tablets
dn34.	TEGRETOL 100mg tablets
dn35.	TEGRETOL 200mg tablets
dn36.	TEGRETOL 400mg tablets
dn37.	TEGRETOL 100mg/5mL sugar free liquid
dn38.	TEGRETOL RETARD 200mg m/r tabs
dn39.	TEGRETOL RETARD 400mg m/r tabs
dn3A.	*EPIMAZ 100mg tablets
dn3B.	*EPIMAZ 200mg tablets
dn3C.	*EPIMAZ 400mg tablets
dn3D.	TEGRETOL 125mg suppositories
dn3E.	TEGRETOL 250mg suppositories
dn3F.	TIMONIL RETARD 200mg m/r tablets
dn3G.	TIMONIL RETARD 400mg m/r tablets
dn3H.	*TERIL CR 200mg m/r tablets
dn3I.	*TERIL CR 400mg m/r tablets
dn3J.	CARBAGEN SR 200mg m/r tablets
dn3K.	CARBAGEN SR 400mg m/r tablets
dn3a.	CARBAMAZEPINE 200mg m/r tabs
dn3b.	CARBAMAZEPINE 400mg m/r tabs
dn3c.	TEGRETOL 100mg chewable tablets
dn3c.	TEGRETOL 100mg chewable tablets
dn3d.	TEGRETOL 200mg chewable tablets
dn3d.	TEGRETOL 200mg chewable tablets
dn3e.	*ARBIL MR 200mg m/r tablets
dn3f.	*ARBIL MR 400mg m/r tablets
dn3v.	CARBAMAZEPINE 125mg suppositories
dn3w.	CARBAMAZEPINE 250mg suppositories
dn3x.	CARBAMAZEPINE 200mg chewable tablets
dn3x.	CARBAMAZEPINE 200mg chewable tablets
dn3y.	CARBAMAZEPINE 100mg chewable tablets
dn3y.	CARBAMAZEPINE 100mg chewable tablets
dn3z.	CARBAMAZEPINE 100mg/5mL sugar free liquid
dn4..	CLONAZEPAM [EPILEPSY CONTROL]
dn41.	*RIVOTRIL 500mcg tablets
dn41.	RIVOTRIL 500micrograms tablets
dn42.	RIVOTRIL 2mg tablets
dn42.	*RIVOTRIL 2mg tablets
dn4w.	CLONAZEPAM 0.5mg/5mL sugar free oral solution
dn4x.	CLONAZEPAM 2mg/5mL sugar free oral solution
dn4y.	CLONAZEPAM 500microgram tablets

For peer review only

dn4z.	CLONAZEPAM 2mg tablets
dn5..	ETHOSUXIMIDE
dn51.	*ETHOSUXIMIDE 250mg capsules
dn52.	*ETHOSUXIMIDE 250mg/5mL elixir
dn53.	*EMESIDE 250mg capsules
dn54.	EMESIDE 250mg/5mL syrup
dn55.	*ZARONTIN 250mg capsules
dn56.	ZARONTIN 250mg/5mL syrup
dn5x.	ETHOSUXIMIDE 250mg capsules
dn5y.	*ETHOSUXIMIDE 250mg/5mL elixir
dn5z.	ETHOSUXIMIDE 250mg/5mL syrup
dn6..	METHYLPHENOBARBITAL
dn61.	*PROMINAL 30mg tablets
dn62.	*PROMINAL 60mg tablets
dn63.	*PROMINAL 200mg tablets
dn6x.	METHYLPHENOBARBITONE 30mg tablets
dn6y.	METHYLPHENOBARBITONE 60mg tablets
dn6z.	METHYLPHENOBARBITONE 200mg tablets
dn7..	PHENOBARBITAL
dn71.	PHENOBARBITAL 15mg tablets
dn72.	PHENOBARBITAL 30mg tablets
dn73.	PHENOBARBITAL 60mg tablets
dn74.	PHENOBARBITAL 100mg tablets
dn75.	PHENOBARBITONE SODIUM 30mg tablets
dn76.	PHENOBARBITONE SODIUM 60mg tablets
dn77.	PHENOBARBITONE 15mg/10mL elixir
dn78.	PHENOBARBITAL 200mg/1mL injection
dn79.	*GARDENAL 200mg/1mL injection
dn7a.	*LUMINAL 15mg tablets
dn7b.	*LUMINAL 30mg tablets
dn7c.	*LUMINAL 60mg tablets
dn7d.	PHENOBARBITAL 15mg/5mL elixir
dn8..	PHENYTOIN
dn81.	EPANUTIN 30mg/5mL suspension
dn82.	EPANUTIN 50mg Infatabs
dn83.	PHENYTOIN 50mg chewable tablets
dn8y.	PHENYTOIN 30mg/5mL suspension
dn8z.	PHENYTOIN 90mg/5mL sugar free suspension
dn9..	PHENYTOIN SODIUM
dn91.	PHENYTOIN 50mg tablets
dn92.	PHENYTOIN 100mg tablets
dn93.	EPANUTIN 25mg capsules
dn93.	*EPANUTIN 25mg capsules
dn94.	EPANUTIN 50mg capsules
dn94.	*EPANUTIN 50mg capsules
dn95.	EPANUTIN 100mg capsules
dn95.	*EPANUTIN 100mg capsules
dn96.	*EPANUTIN 300mg capsules

For peer review only

dn96.	EPANUTIN 300mg capsules
dn97.	*PENTRAN 50mg tablets
dn98.	*PENTRAN 100mg tablets
dn9w.	PHENYTOIN SODIUM 300mg capsules
dn9x.	PHENYTOIN SODIUM 25mg caps
dn9y.	PHENYTOIN SODIUM 50mg capsules
dn9z.	PHENYTOIN SODIUM 100mg capsules
dna..	PRIMIDONE
dna1.	MYSOLINE 250mg tablets
dna1.	*MYSOLINE 250mg tablets
dna2.	MYSOLINE 250mg/5mL oral suspension
dna3.	*MYSOLINE 50mg tablets
dna3.	MYSOLINE 50mg tablets
dnax.	PRIMIDONE 50mg tablets
dnay.	PRIMIDONE 250mg tablets
dnaz.	PRIMIDONE 250mg/5mL oral suspension
dnb..	SODIUM VALPROATE
dnb1.	EPILIM 100mg crushable tablets
dnb2.	EPILIM 200mg e/c tablets
dnb3.	EPILIM 500mg e/c tablets
dnb4.	EPILIM 200mg/5mL sugar free liquid
dnb5.	EPILIM 200mg/5mL syrup
dnb6.	EPILIM IV 400mg/4mL injection
dnb7.	SODIUM VALPROATE 200mg e/c tablets
dnb8.	SODIUM VALPROATE 500mg e/c tablets
dnb9.	ORLEPT STARTER PACK 200mg e/c tablets x10
dnbA.	ORLEPT 200mg/5mL sugar free liquid
dnbB.	EPIVAL CR 300mg m/r tablets
dnbC.	EPIVAL CR 500mg m/r tablets
dnbD.	EPISENTA 300mg/3mL solution for injection
dnbE.	SODIUM VALPROATE 300mg/3mL solution for injection
dnbF.	EPISENTA 150mg m/r capsules
dnbG.	EPISENTA 300mg m/r capsules
dnbH.	EPISENTA 500mg/sachet m/r granules
dnbI.	EPISENTA 1g/sachet m/r granules
dnbJ.	SODIUM VALPROATE 150mg m/r capsules
dnbK.	SODIUM VALPROATE 300mg m/r capsules
dnbL.	SODIUM VALPROATE 500mg/sachet m/r granules
dnbM.	SODIUM VALPROATE 1g/sachet m/r granules
dnbN.	SODIUM VALPROATE 1g/10mL solution for injection
dnbO.	EPISENTA 1g/10mL solution for injection
dnbP.	EPILIM CHRONOSPHERE 50mg/sachet m/r granules
dnbQ.	EPILIM CHRONOSPHERE 100mg/sachet m/r granules
dnbR.	EPILIM CHRONOSPHERE 250mg/sachet m/r granules
dnbS.	EPILIM CHRONOSPHERE 500mg/sachet m/r granules
dnbT.	EPILIM CHRONOSPHERE 750mg/sachet m/r granules
dnbU.	EPILIM CHRONOSPHERE 1g/sachet m/r granules
dnba.	*ORLEPT 200mg e/c tablets

For peer review only

dnbb.	*ORLEPT 500mg e/c tablets
dnbc.	EPILIM CHRONO 200 m/r tablets
dnbd.	EPILIM CHRONO 300 m/r tablets
dnbe.	EPILIM CHRONO 500 m/r tablets
dnbn.	SODIUM VALPROATE 50mg/sachet m/r granules
dnbo.	SODIUM VALPROATE 100mg/sachet m/r granules
dnbp.	SODIUM VALPROATE 250mg/sachet m/r granules
dnbq.	SODIUM VALPROATE 750mg/sachet m/r granules
dnbr.	SODIUM VALPROATE 200mg m/r tablets
dnbs.	SODIUM VALPROATE 300mg m/r tablets
dnbt.	SODIUM VALPROATE 500mg m/r tablets
dnbu.	SODIUM VALPROATE 400mg/4mL injection
dnbv.	SODIUM VALPROATE 100mg crushable tablets
dnbw.	SODIUM VALPROATE 200mg crushable tablets
dnbx.	SODIUM VALPROATE 500mg tablets
dnby.	SODIUM VALPROATE 200mg/5mL sugar free liquid
dnbz.	SODIUM VALPROATE 200mg/5mL syrup
dnc..	CLOBAZAM [EPILEPSY ONLY]
dnc1.	*CLOBAZAM SLS 10mg capsules
dne..	VIGABATRIN
dne1.	VIGABATRIN 500mg tablets
dne2.	SABRIL 500mg tablets
dne3.	VIGABATRIN 500mg powder sachets
dne4.	SABRIL 500mg powder sachets
dnf..	LAMOTRIGINE
dnf1.	LAMOTRIGINE 50mg tablets
dnf2.	LAMOTRIGINE 100mg tablets
dnf3.	LAMICTAL 50mg tablets
dnf4.	LAMICTAL 100mg tablets
dnf5.	LAMOTRIGINE 25mg tablets
dnf6.	LAMICTAL 25mg tablets
dnf7.	LAMICTAL 5mg dispersible tablets
dnf8.	LAMICTAL 25mg dispersible tablets
dnf9.	LAMICTAL 100mg dispersible tablets
dnfA.	LAMOTRIGINE 5mg dispersible tablets
dnfB.	LAMOTRIGINE 25mg dispersible tablets
dnfC.	LAMOTRIGINE 100mg dispersible tablets
dnfD.	LAMICTAL 200mg tablets
dnfE.	LAMOTRIGINE 200mg tablets
dnfF.	LAMICTAL MONOTHERAPY 25mg starter pack
dnfG.	LAMICTAL VALPROATE ADD-ON 25mg starter pack
dnfH.	LAMICTAL NON-VALPROATE ADD-ON 50mg starter pack
dnfJ.	LAMICTAL 2mg dispersible tablets
dnfz.	LAMOTRIGINE 2mg dispersible tablets
dng..	PIRACETAM
dng1.	NOOTROPIL 800mg tablets
dng2.	NOOTROPIL 1.2g tablets
dng3.	NOOTROPIL 33% oral solution

For peer review only

dng4.	PIRACETAM 800mg tablets
dng5.	PIRACETAM 1.2g tablets
dng6.	PIRACETAM 333.3mg/mL oral solution
dnh..	VALPROIC ACID
dnh1.	CONVULEX 150mg e/c capsules
dnh2.	CONVULEX 300mg e/c capsules
dnh3.	CONVULEX 500mg e/c capsules
dnh4.	VALPROIC ACID 150mg e/c capsules
dnh5.	VALPROIC ACID 300mg e/c capsules
dnh6.	VALPROIC ACID 500mg e/c capsules
dnh7.	DEPAKOTE 250mg e/c tablets
dnh8.	DEPAKOTE 500mg e/c tablets
dnhy.	VALPROIC ACID 500mg e/c tablets
dnhz.	VALPROIC ACID 250mg e/c tablets
dni..	FOSPHENYTOIN SODIUM
dni1.	FOSPHENYTOIN SODIUM 750mg/10mL injection concentrate
dni2.	PRO-EPANUTIN 750mg/10mL injection concentrate
dnj..	GABAPENTIN
dnj1.	GABAPENTIN 100mg capsules
dnj2.	GABAPENTIN 300mg capsules
dnj3.	GABAPENTIN 400mg capsules
dnj4.	NEURONTIN 100mg capsules
dnj5.	NEURONTIN 300mg capsules
dnj6.	NEURONTIN 400mg capsules
dnj7.	NEURONTIN 600mg tablets
dnj8.	NEURONTIN 800mg tablets
dnj9.	NEURONTIN 300mg capsules/600mg tablets titration pack
dnjA.	GABAPENTIN 50mg/mL oral solution
dnjx.	GABAPENTIN 300mg capsules/600mg tablets titration pack
dnjy.	GABAPENTIN 600mg tablets
dnjz.	GABAPENTIN 800mg tablets
dnk..	TOPIRAMATE
dnk1.	TOPIRAMATE 50mg tablets
dnk2.	TOPIRAMATE 100mg tablets
dnk3.	TOPIRAMATE 200mg tablets
dnk4.	TOPAMAX 50mg tablets
dnk5.	TOPAMAX 100mg tablets
dnk6.	TOPAMAX 200mg tablets
dnk7.	TOPIRAMATE 25mg tablets
dnk8.	TOPAMAX 25mg tablets
dnk9.	TOPIRAMATE 15mg beads in capsules
dnkA.	TOPIRAMATE 25mg beads in capsules
dnkB.	TOPAMAX SPRINKLE 15mg capsules
dnkC.	TOPAMAX SPRINKLE 25mg capsules
dnkD.	TOPIRAMATE 50mg beads in capsules
dnkE.	TOPAMAX SPRINKLE 50mg capsules
dnl..	TIAGABINE
dnl1.	TIAGABINE 5mg tablets

For peer review only

dnl2.	TIAGABINE 10mg tablets
dnl3.	TIAGABINE 15mg tablets
dnl4.	GABITRIL 5mg tablets
dnl5.	GABITRIL 10mg tablets
dnl6.	GABITRIL 15mg tablets
dnm..	OXCARBAZEPINE
dnm1.	TRILEPTAL 150 tablets
dnm2.	TRILEPTAL 300 tablets
dnm3.	TRILEPTAL 600 tablets
dnm4.	TRILEPTAL 60mg/mL sugar free oral suspension
dnmw.	OXCARBAZEPINE 60mg/mL sugar free oral suspension
dnmx.	OXCARBAZEPINE 150mg tablets
dnmy.	OXCARBAZEPINE 300mg tablets
dnmz.	OXCARBAZEPINE 600mg tablets
dno..	LEVETIRACETAM
dno1.	KEPPRA 250mg tablets
dno2.	KEPPRA 500mg tablets
dno3.	KEPPRA 1g tablets
dno4.	KEPPRA 750mg tablets
dno5.	KEPPRA 100mg/mL s/f oral solution
dno6.	KEPPRA 500mg/5mL solution for injection
dno7.	DESITREND 250mg/sachet granules
dno8.	DESITREND 500mg/sachet granules
dno9.	DESITREND 1g/sachet granules
dnoA.	DESITREND 100mg/mL oral solution
dnoB.	DESITREND 500mg/5mL concentrate for solution for infusion
dnor.	LEVETIRACETAM 1g/sachet granules
dnos.	LEVETIRACETAM 500mg/sachet granules
dnot.	LEVETIRACETAM 250mg/sachet granules
dnou.	LEVETIRACETAM 500mg/5mL solution for injection
dnov.	LEVETIRACETAM 100mg/mL s/f oral solution
dnow.	LEVETIRACETAM 750mg tablets
dnox.	LEVETIRACETAM 1g tablets
dnoy.	LEVETIRACETAM 500mg tablets
dnoz.	LEVETIRACETAM 250mg tablets
dnp..	PREGABALIN
dnp1.	LYRICA 25mg capsules
dnp2.	LYRICA 50mg capsules
dnp3.	LYRICA 75mg capsules
dnp4.	LYRICA 100mg capsules
dnp5.	LYRICA 150mg capsules
dnp6.	LYRICA 200mg capsules
dnp7.	LYRICA 300mg capsules
dnp8.	LYRICA 225mg capsules
dnp9.	LYRICA 20mg/1mL oral solution
dnpA.	LECAENT 25mg capsules
dnpB.	LECAENT 50mg capsules
dnpC.	LECAENT 75mg capsules

For peer review only

dnpD.	LECAENT 100mg capsules
dnpE.	LECAENT 150mg capsules
dnpF.	LECAENT 200mg capsules
dnpG.	LECAENT 225mg capsules
dnpH.	LECAENT 300mg capsules
dnpI.	REWISCA 25mg capsules
dnpJ.	REWISCA 50mg capsules
dnpK.	REWISCA 75mg capsules
dnpL.	REWISCA 100mg capsules
dnpM.	REWISCA 150mg capsules
dnpN.	REWISCA 200mg capsules
dnpO.	REWISCA 225mg capsules
dnpP.	REWISCA 300mg capsules
dnpr.	PREGABALIN 20mg/1mL oral solution
dnps.	PREGABALIN 225mg capsules
dnpt.	PREGABALIN 300mg capsules
dnpu.	PREGABALIN 200mg capsules
dnpv.	PREGABALIN 100mg capsules
dnpw.	PREGABALIN 150mg capsules
dnpx.	PREGABALIN 75mg capsules
dnpy.	PREGABALIN 50mg capsules
dnpz.	PREGABALIN 25mg capsules
dnq..	ZONISAMIDE
dnq1.	ZONISAMIDE 25mg capsules
dnq2.	ZONISAMIDE 50mg capsules
dnq3.	ZONISAMIDE 100mg capsules
dnq4.	ZONEGRAN 25mg capsules
dnq5.	ZONEGRAN 50mg capsules
dnq6.	ZONEGRAN 100mg capsules
dnr..	RUFINAMIDE
dnr1.	INOVELON 100mg tablets
dnr2.	INOVELON 200mg tablets
dnr3.	INOVELON 400mg tablets
dnr4.	INOVELON 40mg/mL oral suspension
dnrw.	RUFINAMIDE 40mg/mL oral suspension
dnrx.	RUFINAMIDE 400mg tablets
dnry.	RUFINAMIDE 200mg tablets
dnrz.	RUFINAMIDE 100mg tablets
dns..	STIRIPENTOL
dns1.	DIACOMIT 250mg capsules
dns2.	DIACOMIT 500mg capsules
dns3.	DIACOMIT 250mg/sachet powder for oral suspension
dns4.	DIACOMIT 500mg/sachet powder for oral suspension
dnsw.	STIRIPENDOL 500mg/sachet powder for oral suspension
dnsx.	STIRIPENTOL 250mg/sachet powder for oral suspension
dnsy.	STIRIPENTOL 500mg capsules
dnsz.	STIRIPENTOL 250mg capsules
dnt..	LACOSAMIDE

For peer review only

dnt1.	VIMPAT 200mg/20mL solution for injection
dnt2.	VIMPAT 15mg/1mL sugar free liquid
dnt3.	VIMPAT 50mg tablets
dnt4.	VIMPAT 100mg tablets
dnt5.	VIMPAT 150mg tablets
dnt6.	VIMPAT 200mg tablets
dnt7.	LACOSAMIDE 200mg/20mL solution for injection
dnt8.	LACOSAMIDE 15mg/1mL sugar free liquid
dnt9.	LACOSAMIDE 50mg tablets
dntA.	LACOSAMIDE 100mg tablets
dntB.	LACOSAMIDE 150mg tablets
dntC.	LACOSAMIDE 200mg tablets
dntD.	VIMPAT 10mg/1mL syrup
dntE.	LACOSAMIDE 10mg/1mL sugar free liquid
dnu..	ESLICARBAZEPINE
dnu1.	ZEBINIX 800mg tablets
dnu2.	ESLICARBAZEPINE ACETATE 800mg tablets
dnv..	RETIGABINE
dnv1.	TROBALT 50mg tablets
dnv2.	TROBALT 100mg tablets
dnv3.	TROBALT 200mg tablets
dnv4.	TROBALT 300mg tablets
dnv5.	TROBALT 400mg tablets
dnv6.	TROBALT tablets initiation pack
dnv7.	RETIGABINE 50mg tablets
dnv8.	RETIGABINE 100mg tablets
dnv9.	RETIGABINE 200mg tablets
dnvA.	RETIGABINE 300mg tablets
dnvB.	RETIGABINE 400mg tablets
dnvC.	RETIGABINE 50mg+100mg tablets initiation pack
dnw..	PERAMPANEL
dnw1.	FYCOMPA 2mg tablets
dnw2.	FYCOMPA 4mg tablets
dnw3.	FYCOMPA 6mg tablets
dnw4.	FYCOMPA 8mg tablets
dnw5.	FYCOMPA 10mg tablets
dnw6.	FYCOMPA 12mg tablets
dnwu.	PERAMPANEL 12mg tablets
dnwv.	PERAMPANEL 10mg tablets
dnww.	PERAMPANEL 8mg tablets
dnwx.	PERAMPANEL 6mg tablets
dnwy.	PERAMPANEL 4mg tablets
dnwz.	PERAMPANEL 2mg tablets
do...	STATUS EPILEPTICUS DRUGS
do1..	DIAZEPAM [EPILEPSY USE]
do11.	DIAZEMULS [EP] 10mg/2mL injection
do12.	STESOLID [EP] 10mg/2mL injection
do13.	*STESOLID 20mg/4mL injection

For peer review only

do14.	STESOLID 5mg/2.5mL rectal solution
do15.	STESOLID 10mg/2.5mL rectal solution
do16.	VALIUM [EP] 10mg/2mL injection
do17.	VALIUM [EP] 20mg/4mL injection
do18.	DIAZEPAM 5mg/2.5mL RecTubes
do19.	DIAZEPAM 10mg/2.5mL RecTubes
do1A.	DIAZEPAM 2.5mg/1.25mL RecTubes
do1B.	*DIAZEPAM 20mg/5mL RecTubes
do1t.	DIAZEPAM 2.5mg/1.25mL rectal solution
do1u.	DIAZEPAM 20mg/5mL rectal solution
do1v.	DIAZEPAM 10mg/2mL emulsion injection
do1w.	DIAZEPAM 10mg/2mL injection
do1x.	DIAZEPAM 5mg/2.5mL rectal solution
do1y.	DIAZEPAM 10mg/2.5mL rectal solution
do1z.	*DIAZEPAM 20mg/4mL injection
do2..	CLONAZEPAM [STATUS EPILEPSY]
do21.	*RIVOTRIL 1mg/1mL injection
do21.	RIVOTRIL 1mg/1mL injection
do2z.	CLONAZEPAM 1mg/1mL injection
do2z.	*CLONAZEPAM 1mg/1mL injection
do3..	CLOMETHIAZOLE EDISYLATE [CENTRAL NERVOUS SYSTEM USE]
do31.	HEMINEVRIN [CNS] 8mg/mL intravenous infusion
do3z.	CLOMETHIAZOLE EDISYLATE 8mg/mL intravenous infusion
do4..	LORAZEPAM [EPILEPSY]
do41.	ATIVAN [EP] 4mg/mL injection
do5..	PARALDEHYDE
do51.	*PARALDEHYDE injection 5mL
do52.	*PARALDEHYDE injection 10mL
do6..	PHENYTOIN SODIUM [STATUS EPILEPSY]
do61.	EPANUTIN [EP] 250mg/5mL injection
do6z.	PHENYTOIN SODIUM 250mg/5mL injection

ALT_CODE	DESCRIPTION
G40	Epilepsy
G400	Localization-related (focal)(partial) idiopathic epilepsy and epileptic syndromes with seizures of localized onset
G401	Localization-related (focal)(partial) symptomatic epilepsy and epileptic syndromes with simple partial seizures
G402	Localization-related (focal)(partial) symptomatic epilepsy and epileptic syndromes with complex partial seizures
G403	Generalized idiopathic epilepsy and epileptic syndromes
G404	Other generalized epilepsy and epileptic syndromes
G405	Special epileptic syndromes
G406	Grand mal seizures, unspecified (with or without petit mal)
G407	Petit mal, unspecified, without grand mal seizures
G408	Other epilepsy
G409	Epilepsy, unspecified
G41	Status epilepticus

For peer review only

G410	Grand mal status epilepticus
G411	Petit mal status epilepticus
G412	Complex partial status epilepticus
G418	Other status epilepticus
G419	Status epilepticus, unspecified

For peer review only

The RECORD statement – checklist of items, extended from the STROBE statement, that should be reported in observational studies using routinely collected health data.

	Item No.	STROBE items	Location in manuscript where items are reported	RECORD items	Location in manuscript where items are reported
**Title and abstract**					
	1	(a) Indicate the study's design with a commonly used term in the title or the abstract (b) Provide in the abstract an informative and balanced summary of what was done and what was found		RECORD 1.1: The type of data used should be specified in the title or abstract. When possible, the name of the databases used should be included.<br><br>RECORD 1.2: If applicable, the geographic region and timeframe within which the study took place should be reported in the title or abstract.<br><br>RECORD 1.3: If linkage between databases was conducted for the study, this should be clearly stated in the title or abstract.	Page 1<br><br>Page 1<br><br>Page 1
**Introduction**					
Background rationale	2	Explain the scientific background and rationale for the investigation being reported			Page 3
Objectives	3	State specific objectives, including any prespecified hypotheses			Page 3
**Methods**					
Study Design	4	Present key elements of study design early in the paper			Pages 3-4
Setting	5	Describe the setting, locations, and relevant dates, including periods of recruitment, exposure, follow-up, and data collection			Page 4

For peer review only

Participants	6	*(a) Cohort study* - Give the eligibility criteria, and the sources and methods of selection of participants. Describe methods of follow-up<br>*Case-control study* - Give the eligibility criteria, and the sources and methods of case ascertainment and control selection. Give the rationale for the choice of cases and controls<br>*Cross-sectional study* - Give the eligibility criteria, and the sources and methods of selection of participants<br><br>*(b) Cohort study* - For matched studies, give matching criteria and number of exposed and unexposed<br>*Case-control study* - For matched studies, give matching criteria and the number of controls per case		RECORD 6.1: The methods of study population selection (such as codes or algorithms used to identify subjects) should be listed in detail. If this is not possible, an explanation should be provided.<br><br>RECORD 6.2: Any validation studies of the codes or algorithms used to select the population should be referenced. If validation was conducted for this study and not published elsewhere, detailed methods and results should be provided.<br><br>RECORD 6.3: If the study involved linkage of databases, consider use of a flow diagram or other graphical display to demonstrate the data linkage process, including the number of individuals with linked data at each stage.	Page 4<br><br>See flow diagram (Figure 1)
Variables	7	Clearly define all outcomes, exposures, predictors, potential confounders, and effect modifiers. Give diagnostic criteria, if applicable.		RECORD 7.1: A complete list of codes and algorithms used to classify exposures, outcomes, confounders, and effect modifiers should be provided. If these cannot be reported, an explanation should be provided.	Page 4<br>Supplementary file 1
Data sources/ measurement	8	For each variable of interest, give sources of data and details of methods of assessment (measurement).<br>Describe comparability of assessment methods if there is more than one group			Page 4

For peer review only

Bias	9	Describe any efforts to address potential sources of bias			Adjusted for confounders Page 4
Study size	10	Explain how the study size was arrived at			See flow diagram (Figure 1)
Quantitative variables	11	Explain how quantitative variables were handled in the analyses. If applicable, describe which groupings were chosen, and why			Page 4
Statistical methods	12	(a) Describe all statistical methods, including those used to control for confounding<br>(b) Describe any methods used to examine subgroups and interactions<br>(c) Explain how missing data were addressed<br>(d) *Cohort study* - If applicable, explain how loss to follow-up was addressed<br>*Case-control study* - If applicable, explain how matching of cases and controls was addressed<br>*Cross-sectional study* - If applicable, describe analytical methods taking account of sampling strategy<br>(e) Describe any sensitivity analyses			Page 4
Data access and cleaning methods		..		RECORD 12.1: Authors should describe the extent to which the investigators had access to the database population used to create the study population.	Page 3

For peer review only

				RECORD 12.2: Authors should provide information on the data cleaning methods used in the study.	
Linkage		..		RECORD 12.3: State whether the study included person-level, institutional-level, or other data linkage across two or more databases. The methods of linkage and methods of linkage quality evaluation should be provided.	Page 4
**Results**					
Participants	13	(a) Report the numbers of individuals at each stage of the study (*e.g.*, numbers potentially eligible, examined for eligibility, confirmed eligible, included in the study, completing follow-up, and analysed)<br>(b) Give reasons for non-participation at each stage.<br>(c) Consider use of a flow diagram		RECORD 13.1: Describe in detail the selection of the persons included in the study (*i.e.,* study population selection) including filtering based on data quality, data availability and linkage. The selection of included persons can be described in the text and/or by means of the study flow diagram.	Flow diagram (Figure 1)
Descriptive data	14	(a) Give characteristics of study participants (*e.g.*, demographic, clinical, social) and information on exposures and potential confounders<br>(b) Indicate the number of participants with missing data for each variable of interest<br>(c) *Cohort study* - summarise follow-up time (*e.g.*, average and total amount)			Page 7
Outcome data	15	*Cohort study* - Report numbers of outcome events or summary measures over time<br>*Case-control study* - Report numbers in each exposure			Page 8-10

For peer review only

		category, or summary measures of exposure<br>*Cross-sectional study* - Report numbers of outcome events or summary measures			
Main results	16	(a) Give unadjusted estimates and, if applicable, confounder-adjusted estimates and their precision (e.g., 95% confidence interval). Make clear which confounders were adjusted for and why they were included<br>(b) Report category boundaries when continuous variables were categorized<br>(c) If relevant, consider translating estimates of relative risk into absolute risk for a meaningful time period			Page 9, Page 4 &5
Other analyses	17	Report other analyses done—e.g., analyses of subgroups and interactions, and sensitivity analyses			Page 5
**Discussion**					
Key results	18	Summarise key results with reference to study objectives			Page 5
Limitations	19	Discuss limitations of the study, taking into account sources of potential bias or imprecision. Discuss both direction and magnitude of any potential bias		RECORD 19.1: Discuss the implications of using data that were not created or collected to answer the specific research question(s). Include discussion of misclassification bias, unmeasured confounding, missing data, and changing eligibility over time, as they pertain to the study being reported.	Page 5
Interpretation	20	Give a cautious overall interpretation of results considering objectives,			Page 5

		limitations, multiplicity of analyses, results from similar studies, and other relevant evidence			
Generalisability	21	Discuss the generalisability (external validity) of the study results			Page 6
**Other Information**					
Funding	22	Give the source of funding and the role of the funders for the present study and, if applicable, for the original study on which the present article is based			Page 6
Accessibility of protocol, raw data, and programming code		..		RECORD 22.1: Authors should provide information on how to access any supplemental information such as the study protocol, raw data, or programming code.	Page 6

*Reference: Benchimol EI, Smeeth L, Guttmann A, Harron K, Moher D, Petersen I, Sørensen HT, von Elm E, Langan SM, the RECORD Working Committee. The REporting of studies Conducted using Observational Routinely-collected health Data (RECORD) Statement. *PLoS Medicine* 2015; in press.

*Checklist is protected under Creative Commons Attribution (CC BY) license.

For peer review only
